# Explore the active ingredients and potential mechanisms of JianPi QingRe HuaYu Methods in the treatment of gastric inflammation-cancer transformation by network pharmacology and experimental validation

**DOI:** 10.1186/s12906-023-04232-0

**Published:** 2023-11-14

**Authors:** Kechao Nie, Zhihua Zheng, Xiushen Li, Yonglong Chang, FengBin Liu, Xiaoyu Wang

**Affiliations:** 1 Department of Integrated Traditional Chinese & Western Medicine, The Second Xiangya Hospital, Central South University, Changsha, 410011 China; 2 School of Health Science, Guangdong Pharmaceutical University, Guangzhou, 510006 China; 3 The Fourth Clinical Medical College, Guangzhou University of Chinese Medicine, Shenzhen, 518033 China; 4 Department of Gastroenterology, Shenzhen Traditional Chinese Medicine Hospital, Shenzhen, 518000 China; 5 Shenzhen University General Hospital, Shenzhen, 518060 China; 6 Guangdong Key Laboratory for Biomedical Measurements and Ultrasound Imaging, School of Biomedical Engineering, Shenzhen University Health Science Center, Shenzhen, 518060 China; 7 The First Affiliated Hospital, Guangzhou University of Chinese Medicine, Guangzhou, 510405 China

**Keywords:** JianPi QingRe HuaYu Methods, Chronic atrophic gastritis, Inflammation-cancer transformation, Quercetin

## Abstract

**Background:**

JianPi QingRe HuaYu Methods (JQH) have been long used to treat chronic atrophic gastritis (CAG) and precancerous lesions of gastric cancer (PLGC). However, whether JQH can inhibit the transformation of gastritis to gastric cancer (GC) remains unclear.

**Methods:**

Herein, we first retrieved the active ingredients and targets of JQH from the TCMSP database and the targets related to the gastric inflammation-cancer transformation from public databases. Differentially expressed genes (DEGs) related to gastric inflammation-cancer transformation were identified from the Gene Expression Omnibus (GEO) database. Then, we obtained the potential therapeutic targets of JQH in treating gastric inflammation-cancer transformation by intersecting drugs and disease targets. The Gene Ontology (GO), Kyoto Encyclopedia of Genes and Genomes (KEGG), and protein–protein interaction (PPI) analyses of the potential therapeutic targets were conducted using R software. Next, we conducted molecular docking and in vitro experiments to validate our results.

**Results:**

We obtained 214 potential therapeutic targets of JQH by intersecting drugs and disease targets. We found that the potential mechanisms of JQH in treating gastric inflammation-cancer transformation might be related to JAK-STAT, Wnt, p53 and VEGF signaling pathways. The molecular docking indicated that quercetin, as the main active ingredient of JQH, might inhibit gastric inflammation-cancer transformation by binding with specific receptors. Our experimental results showed that quercetin inhibited cells proliferation (*P* < 0.001), promoted cell apoptosis (*P* < 0.001), reduced the secretion of pro-inflammatory cytokines (*P* < 0.001) and promoted the secretion of anti-inflammatory cytokines (*P* < 0.001) in MNNG-induced GES-1 cells. Furthermore, quercetin inhibited cells proliferation (*P* < 0.001) and reduced mRNA and protein level of markers of PLGC (*P* < 0.001) in CDCA-induced GES-1 cells.

**Conclusion:**

These results provide the material basis and regulatory mechanisms of JQH in treating gastric inflammation-cancer transformation.

**Supplementary Information:**

The online version contains supplementary material available at 10.1186/s12906-023-04232-0.

## Introduction

Chronic atrophic gastritis (CAG) is a common gastrointestinal disease characterized by mucosa atrophy, exposed vessels, and mucosal nodules [1]. Correa cascade proposed that CAG often develops into intestinal gastric cancer (GC) in the pathogenesis of disease evolution [2]. A recent study has suggested that *Helicobacter pylori* (Hp) infection plays a vital role in this process [3]. Hp eradication in patients with gastritis can prevent the occurrence of intestinal GC to some extent [4]. However, 34-54% of patients with atrophic gastritis develop GC even after Hp eradication [5]. Thus, finding effective drugs to inhibit gastric inflammation-cancer transformation is urgent.

Traditional Chinese Medicine (TCM) are complementary and alternative medicine in treating gastrointestinal diseases and has specific advantages compared to Western medicine, such as less drug dependence and side effects [6]. JianPi QingRe HuaYu, a frequently-used Traditional Chinese Medicine, is often used for treatment of CAG and precancerous lesions of gastric cancer (PLGC) in clinic [7]. JQH is composed of Astragalus membranaceus (Fisch.) Bunge (Huangqi), Atractylodes macrocephala Koidz (Baizhu), Citrus aurantium L. (Zhike), Hedyotis diffusa Willd. (Baihuasheshecao), Scutellaria barbata D. Don (Bazhilian), and Curcuma zedoaria (Christm.) Rosc. (Ezhu) (Table 1). Our clinical studies have shown that JQH could significantly ameliorate digestive tract symptoms and pathological conditions of gastric mucosal and inflammatory levels in patients with CAG or PLGC [7, 8]. In experimental animal studies, JQH improved the pathological condition of gastric mucosa and delayed the development of intestinal metaplasia or atypical hyperplasia by suppressing NF-κB pathway activation in CAG rats [9, 10]. However, whether JQH inhibits CAG from proceeding to GC (gastric inflammation-cancer transformation) remains unclear.

**Table 1:** Details of the ingredients of JianPi QingRe HuaYu Methods

Latin binomial nomenclature	Family	Medicinal parts	Chinese name
Astragalus membranaceus (Fisch.) Bunge	Leguminosae	root	Huangqi
Atractylodes macrocephala Koidz	Compositae	root	Baizhu
Citrus aurantium L	Rutaceae	fruit	Zhike
Hedyotis diffusa Willd	Rubiaceae	herb	Baihuasheshecao
Scutellaria barbata D. Don	Lamiaceae	herb	Bazhilian
Curcuma zedoaria (Christm.) Rosc	Zingiberaceae	root	Ezhu

**Fig. 1:**
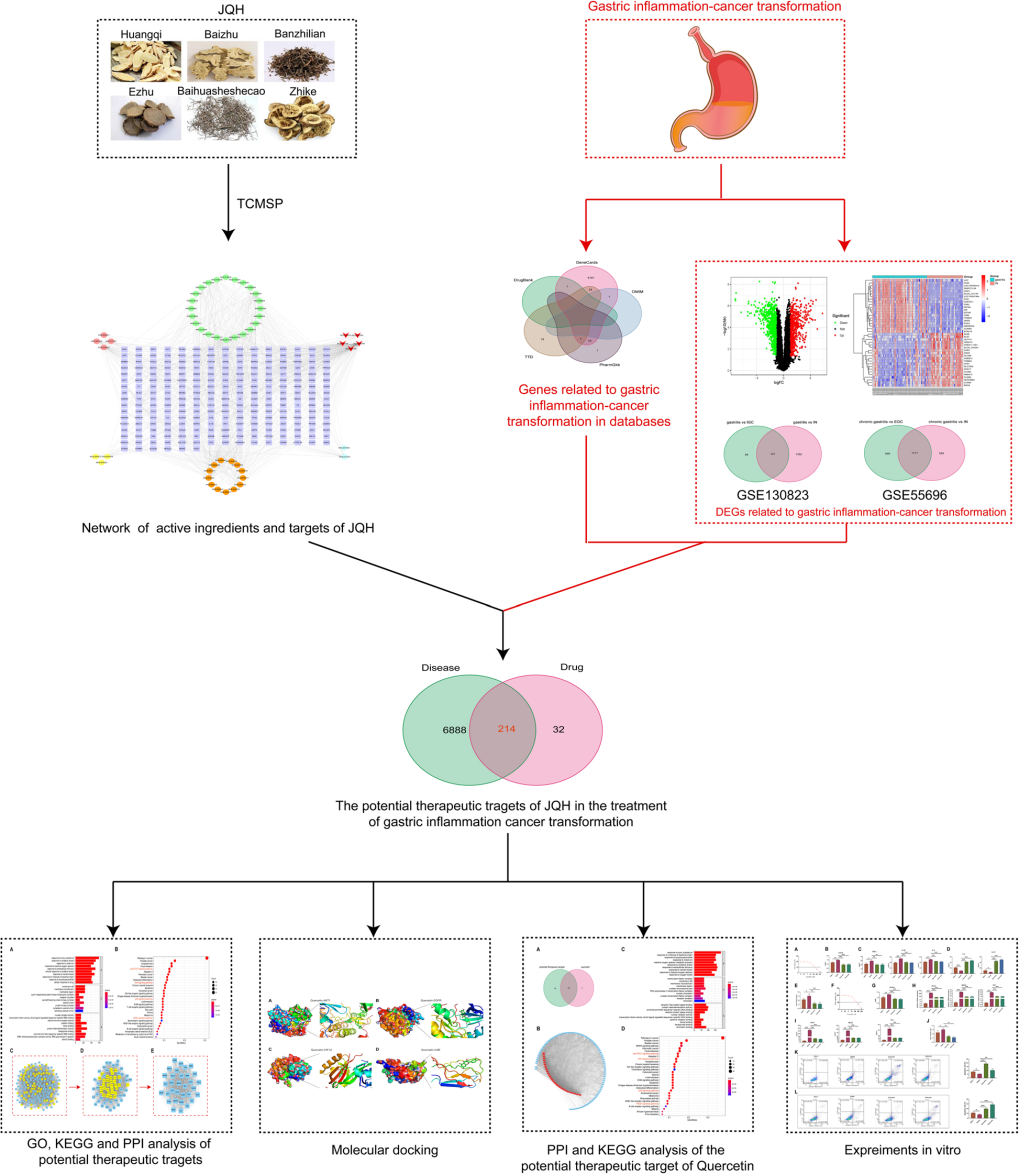
Flow chart of network pharmacology and experimental validation strategies to explore JQH in treating gastric inflammation-cancer transformation

Network pharmacology has become an effective approach to investigating the mechanisms of drug therapy, screening the active ingredients, and exploring the therapeutic targets of TCM [11]. Therefore, in the present study, we used network pharmacology, bioinformatics, molecular docking, and in vitro experiments for validation to reveal the active ingredients, targets, and potential mechanisms of JQH to treat gastric inflammation-cancer transformation. The flow chart of this study is shown in Fig. 1.

## Materials and methods

### Screen of JQH active ingredients and targets

We screened the JQH bioactive ingredients in the TCMSP database (https://old.tcmsp-e.com/tcmsp.php) according to the cut-off value of drug-like proprieties ≥ 018 and bioavailability ≥ 30%. The corresponding targets related to all these bioactive ingredients were also retrieved from the TCMSP database, and gene symbols were converted using the Uniport database (https://www.uniprot.org/).

### Identification of gastric inflammation-cancer transformation targets by GEO database

The GSE130823 and GSE55696 datasets were retrieved from the Gene Expression Omnibus (GEO) database (https://www.ncbi.nlm.nih.gov/geo/). To identify the genes related to gastric inflammation-cancer transformation, differently expressed genes (DEGs) among different subgroups were identified based on FDR < 0.05 and |logFC|> 1. Then, the DEGs in the intersection of gastritis versus (*vs*.) intraepithelial neoplasia (IN) groups and gastritis *vs*. intestinal gastric cancer (IGC) groups in the GSE130832 dataset were selected. The DEGs obtained from chronic gastritis *vs*. IN groups and chronic gastritis *vs*. early gastric cancer (EGC) groups were also intersected in GSE55696 datasets. The intersected DEGs in GSE130823 and GSE55696 datasets were regarded as the targets for gastric inflammation-cancer transformation in the GEO database.

### Collection of gastric inflammation-cancer transformation targets

We also collected targets using the keywords “atrophic gastritis”, “gastric adenocarcinoma,” and “stomach adenocarcinoma” in OMIM, TTD, DrugBank, Genecard, and PharmGkb databases. The targets obtained using the keyword “gastric adenocarcinoma” were combined with those obtained using the keyword “stomach adenocarcinoma”. Then, we intersected these combined targets with the targets identified by the keyword “atrophic gastritis” in each database. Finally, we combined the targets from the GEO and online databases using R software, and these targets were regarded as gene targets related to gastric inflammation-cancer transformation.

### Potential therapeutic targets of JQH in treating gastric inflammation-cancer transformation

The targets of gastric inflammation-cancer transformation were intersected with the targets of JQH bioactive ingredients. These genes were regarded as the potential therapeutic targets of JQH in treating gastric inflammation-cancer transformation. This procedure was conducted using R software.

### Protein–protein Interaction (PPI) network, Gene Ontology (GO), and Kyoto Encyclopedia Gene and Genomes (KEGG) analyses

The potential therapeutic targets of JQH in treating gastric inflammation-cancer transformation were subjected to PPI network analysis by Cytoscape software. The PPI network was constructed using the CytoNCA tool in Cytoscape to identify the core genes based on specific parameters (Betweenness, Closeness, Eigenvector, and LAC) ≥ median value. To investigate the molecular mechanisms of JQH in treating gastric inflammation-cancer transformation, GO and KEGG enrichment analyses were also performed using R software.

### Molecular docking

The 2D structure of active ingredients was obtained from the PubChem database. These 2D structures were converted into 3D structures using ChemBio3D software. The PDB database was used to obtain the 3D structures of core targets. The “AutoDockTool” was used to convert the 3D structure of active ingredients. The four targets were AKT1, EGFR, HIF1A and IL6R. These targets include ligand and water removal, hydrogen addition, and amino acid optimization and patching.Then, these four targets were saved as PDBQT format files and we identify the active pockets. Finally, molecular docking was conducted using “vina” software.

### Identification of the key ingredients of JQH

We intersected the targets of the active ingredients of JQH with the therapeutic targets, and the ingredients with the most therapeutic targets were regarded as core JQH ingredients in the treatment of gastric inflammation-cancer transformation. Further, bioinformatics approaches were used to investigate the molecular mechanisms of these therapeutic targets related to core active ingredients.

### Cell culture

Cells were cultured in RPMI-1640 medium with 10% fetal bovine serum and 50 mg/mL streptomycin, and 50 U/mL penicillin in an incubator at 37 °C and 5% CO_2_. Then, 20 μM N-methyl-N'-nitro-N-nitrosoguanidine (MNNG) and 100 μM chenodeoxycholic acid (CDCA) were added to GES-1 cells for 24 h to establish an inflammatory and intestinal metaplasia model of the gastric mucosa.

### Cell proliferation assay

First, cells were seeded in a 96-well microplate (6000 cells/well, 100 μL). Then, cells were routinely incubated for 24 h in a humidified incubator. After 24 h of pre-incubation, the medium was aspirated and exchanged containing celecoxib (100 μM) and/or quercetin (140 μM in MNNG-GES-1 cells, 150 μM in CDCA-GES-1 cells). Cell viability was measured using the Cell Counting Kit-8 (CCK-8) after 24 h incubation. Cells were seeded in 96-well plates, 10 μL CCK-8 solution was added to each well, and incubated at 37 °C. The optical density (OD) value of each well was measured at 450 nm.

### Enzyme-Linked Immunosorbent Assay (ELISA)

After drug intervention for 24 h, cells supernatants were collected to detect the expression of TNF-α, IL-1β, IL-6 (pro-inflammatory cytokines), IL-4 and IL-10 (anti-inflammatory cytokines) by ELISA kits from Beyotime (Shanghai, China) following the manufacturer’s instructions. Similarly, cell lysate were collected to detect the expression of KLF4, MUC2 and VIL1(biomarkers of PLGC) by ELISA kits. Each well received 100 µL prepared standard. The whole plate was sealed with a plate sealing membrane and incubated at 4 ℃ overnight. The prepared 1 × wash solution was added to the plate washer, washed four times, and 300 µL wash solution was added to each well. Moreover, 100 µL prepared detection antibody (biotin-labeled antibody) was added to each well and incubated at room temperature for 1 h. Next, 100 µL HRP-streptavidin was added to each well and incubated at room temperature for 45 min. Then, 100 µL TMB chromogenic solution was added to each well and incubated at room temperature in the dark for 30 min. Finally, 50 µL of termination solution was added to each well and immediately read at 450 nm.

### RT-qPCR

Total RNA from cells was isolated with the TRIzol reagent (Invitrogen, CA, USA) following the manufacturer’s instructions. One μg of total RNA was reverse transcribed using PrimeScript RT Master Mix Kit (TaKaRa Bio Inc., Kusatsu, Japan). Real-time PCR was performed on the cDNA using SYBR Green PCR Kit (TaKaRa Bio Inc.). GAPDH mRNA was used as the endogenous control. Primers are described in Table S1.

### Alkaline phosphatase (ALP) activity measurement

Cells were seeded in 12-well microplates at a density of 1 × 10^5^ cells/mL and treated with celecoxib (100 μM) or quercetin (140 μM in MNNG-GES-1 cells, 150 μM in CDCA-GES-1 cells) for 24 h before being assayed for ALP activity. After drug intervention for 24 h, cells were dissolved with non-denatured cell lysate to detect the ALP activity at 360/450 nm by QuantiFluoTM Alkaline Phosphatase Assay Kit (California, USA) following the manufacturer’s instructions.

### Cell apoptosis assay

Cell apoptosis kits were purchased from Beyotime (Shanghai, China), and the experiment was conducted following the manufacturer’s instructions. Cells were centrifuged at approximately 1,000 g for 3–5 min and resuspended in phosphate-buffered saline. Next, 50,000–100,000 suspended cells were centrifuged at 1,000* g* for 5 min. After discarding the supernatants, Annexin V-FITC binding solution (195 μL) was added to resuspend the cells gently. Next, Annexin V-FITC (5 μL) and propyl iodide staining solution (10 μL) were added and mixed. The solution was incubated at room temperature (20–25 ℃) for 10–20 min in the dark and analyzed using a CytoFLEX S flow cytometer (Beckman Coulter, CA, USA).

### Statistical analysis

The mean ± standard deviation was used to express all data, which were subjected to normality distribution before statistical analysis. In this study, there were at least three samples in each group. Statistical analysis was conducted using SPSS version 24.0 (IBM Corp., Armonk, NY, USA). Images were plotted using GraphPad Prism 8.0 (GraphPad Software Inc., San Diego, CA, USA). Student’s t-test and analysis of variance (ANOVA) were performed for comparisons between groups. *P*-values < 0.05 denoted statistically significant differences.

## Results

### Active ingredients and targets of JQH

**Fig. 2:**
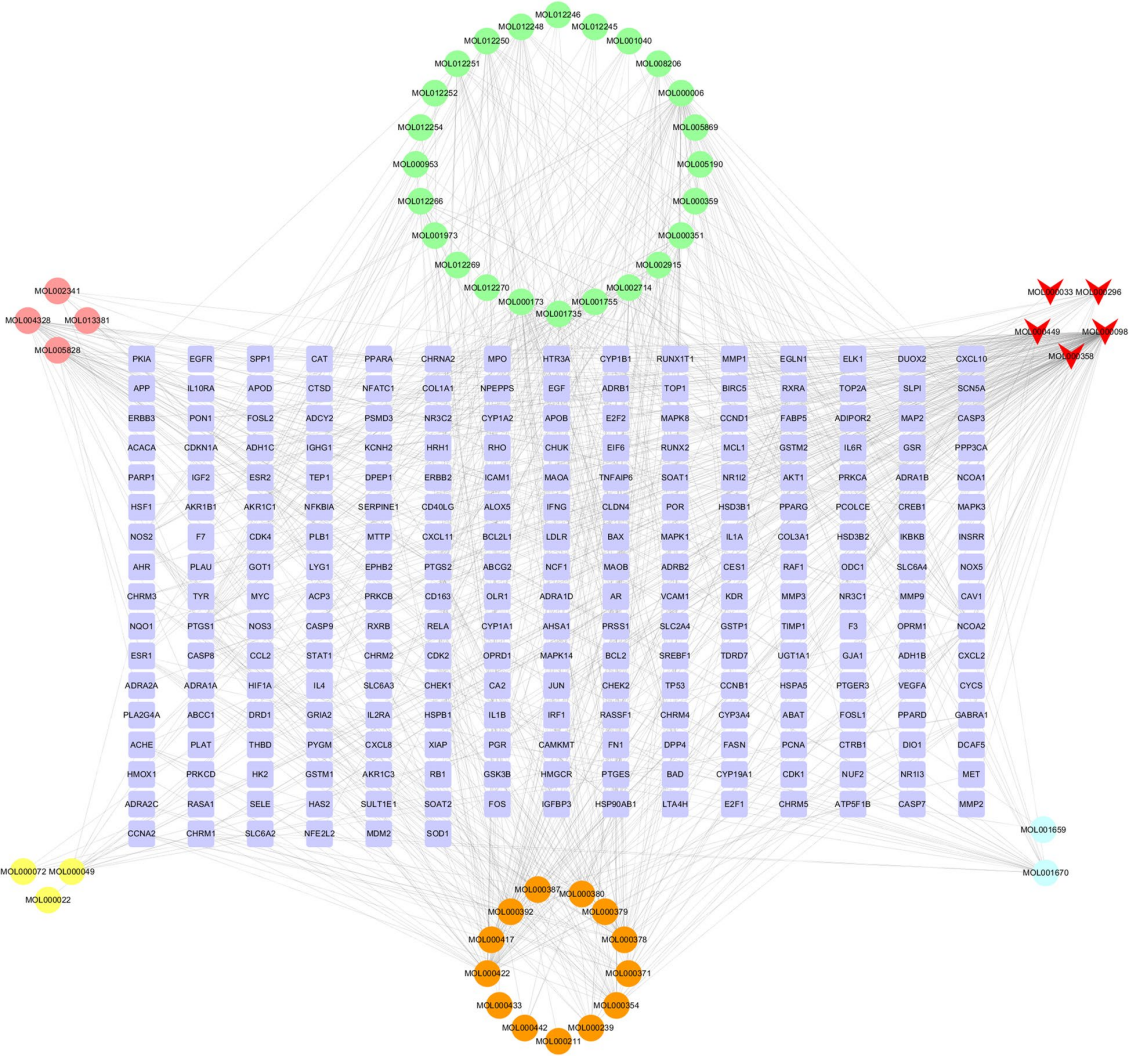
Regulatory network of active ingredients and targets of JQH

We obtained 51 active ingredients and 246 potential targets of JQH in the TCMSP database based on our filter condition (Table S2). The regulatory network between ingredients and targets of JQH was constructed using Cytoscape**.** The rectangles represent the potential targets, and the surrounding circle represents the active ingredients of JQH. Especially the green, pink, yellow, brown, and blue circles of Fig. 2 represent the active ingredients from Baizhilian, Zhike, Baizhu, Huangqi, and Baihuasheshecao, respectively. The red triangle represents the active ingredients from more than two herbs (Fig. 2).

### Potential therapeutic targets for JQH

**Fig. 3:**
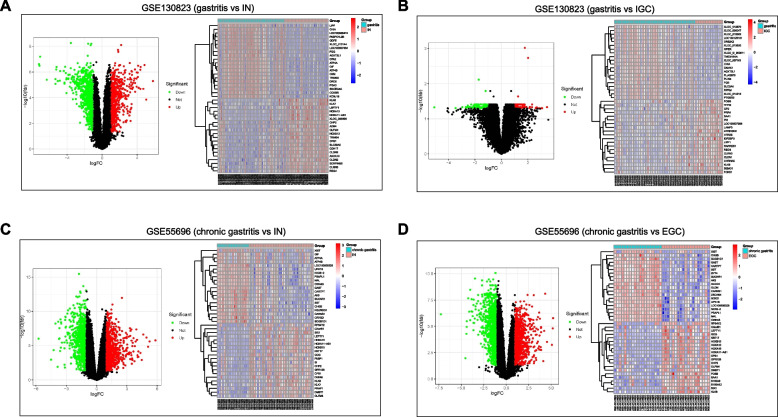
Identification of DEGs related to gastric inflammation-cancer transformation in GSE130823 and GSE55696 datasets. Volcano plot of DEGs and heatmap of the top 20 upregulated and downregulated genes in gastritis *vs*. IN groups (**A**) and gastritis *vs*. IGC groups (**B**) in the GSE130823 dataset. Volcano plot of DEGs and heatmap of the top 20 upregulated and downregulated genes in chronic gastritis *vs*. IN groups (**C**) and chronic gastritis *vs*. EGC groups (**D**) in the GSE55696 dataset

**Fig. 4:**
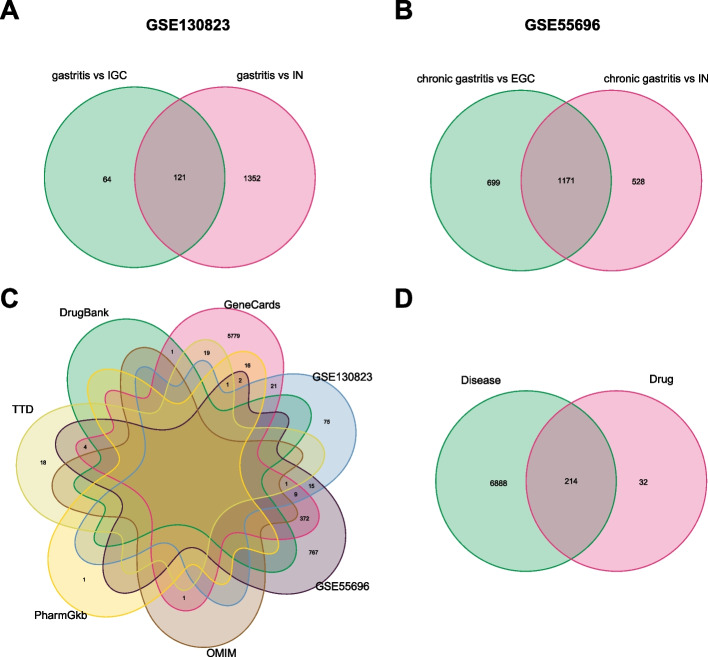
Potential therapeutic targets of JQH in treating gastric inflammation-cancer transformation. DEGs related to gastric inflammation-cancer transformation in GSE130823 (**A**) and GSE55696 (**B**) datasets. **C** Identification of gastric inflammation-cancer transformation targets in public and GEO databases. **D** Potential therapeutic targets of JQH in treating gastric inflammation-cancer transformation

**Fig. 5:**
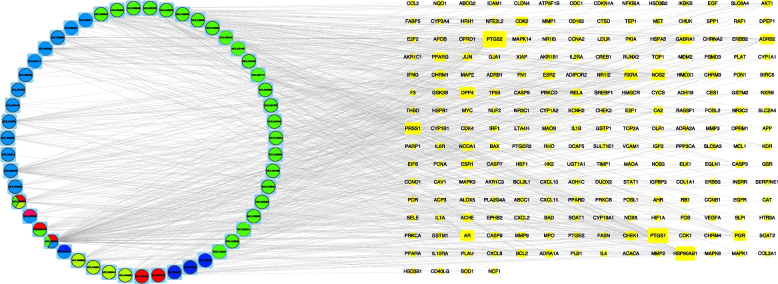
Regulatory network of 150 potential therapeutic targets and corresponding active ingredients

The GSE130823 dataset comprised 47 gastritis tissues, 31 IN samples, and 16 IGC samples. In the GSE130823 dataset, 1473 DEGs were detected in the IN group (Fig. 3A and Table S3) and 185 DEGs in the IGC group (Fig. 3B and Table S4) compared to the gastritis group. Then, 121 intersecting genes were obtained from gastritis *vs*. IN and gastritis *vs*. IGC subgroups (Fig. 4A). The GSE55696 dataset comprised 19 chronic gastritis tissues, 39 IN samples, and 19 EGC samples. In the GSE55696 dataset, 1699 DEGs were detected in the IN group (Fig. 3C and Table S5) and 1870 DEGs in the EGC group (Fig. 3D and Table S6) compared to the chronic gastritis group. Next, 1171 intersecting genes were obtained from chronic gastritis *vs*. IN and chronic gastritis *vs*. EGC subgroups (Fig. 4B). We found 1, 43, 1, 6226 and 20 therapeutic targets in OMIM, TTD, DrugBank, Genecard, and PharmGkb databases, respectively (Fig. 4C). A total of 7102 therapeutic targets were obtained by combining the targets from GEO and online databases (Fig. 4C). To identify the potential therapeutic targets for JQH, we intersected the 7102 targets with the targets of all active ingredients of JQH and obtained 214 repeated targets (Fig. 4D and Table S7). The regulatory network of 51 active ingredients and 214 therapeutic targets is presented in Fig. 5.

### GO and KEGG enrichment analysis of therapeutic targets

**Fig. 6:**
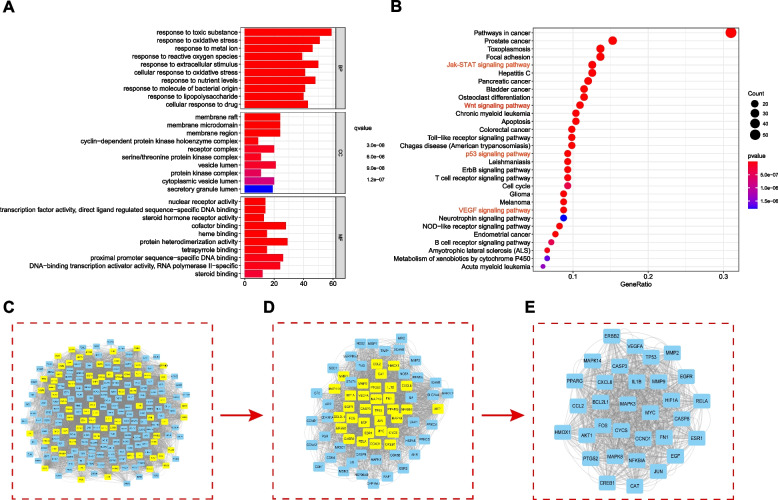
GO, KEGG, and PPI analysis of potential therapeutic targets. GO (**A**) and KEGG (**B**) enrichment analysis of potential therapeutic targets. (**C**-**E**) Core targets in the PPI network by the CytoNCA tool in Cytoscape

To explore the potential mechanisms of JQH in treating gastric inflammation-cancer transformation, we conducted GO and KEGG enrichment analysis of the 214 therapeutic targets using R software. For GO annotation, the genes were enriched in response to lipolysaccharide, cellular response to drugs, and response to oxidative stress in Biological Process (BP); receptor complex, protein kinase complex and cyclin − dependent protein kinase holoenzyme complex in Cellular Components (CC); and nuclear receptor activity, phosphatase binding, and cofactor binding in Molecular Function (MF) (Fig. 6A). The KEGG pathways showed that the targets were enriched in JAK-STAT, Wnt, p53 and VEGF signaling pathways (Fig. 6B).

### PPI network and identification of core genes

The PPI network of the 214 therapeutic targets was plotted using Cytoscape. The network has 214 nodes and 4046 edges in this regulatory network (Fig. 6C). The PPI network was further analyzed by the CytoNCA tool in Cytoscape to identify the core genes based on specific parameters (Betweenness, Closeness, Eigenvector, and LAC) ≥ median value, respectively. A sub-network was obtained from the genes with Betweenness > 71.42, Closeness > 0.51, Eigenvector > 0.05, and LAC > 38.21(Fig. 6D). Similarly, the genes with Betweenness, Closeness, Eigenvector, and LAC ≥ median value in the subnetwork were subjected to further analysis using the same methods. The core network comprised 32 genes with Betweenness > 15.66, Closeness > 0.74, Eigenvector > 0.12, and LAC > 73.58 (Fig. 6E). These 32 genes might be the potential core targets of JQH in treating gastric inflammation-cancer transformation.

### Molecular docking

**Fig. 7:**
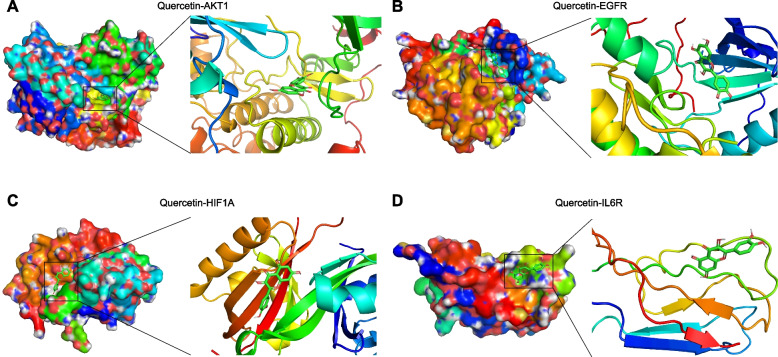
Molecular docking of core targets with quercetin: quercetin to AKT1 (**A**), quercetin to EGFR (**B**), quercetin to HIF1A (**C**), and quercetin to IL6R (**D**)

Furthermore, we conducted the molecular docking of several genes related to inflammation-cancer transformation with quercetin in JQH. Four ingredient-target pairs were analyzed: quercetin-AKT1, quercetin-EGFR, quercetin-HIF1A, and quercetin-IL6R. The lowest binding energies of these compounds with their targets are presented in Fig. 7 and Table S7. The interactions between ligand and target proteins is shown in Table S8. All these results showed quercetin mostly interacted with AKT1, EGFR, HIF1A and IL6R.

### Core JQH ingredients

**Fig. 8:**
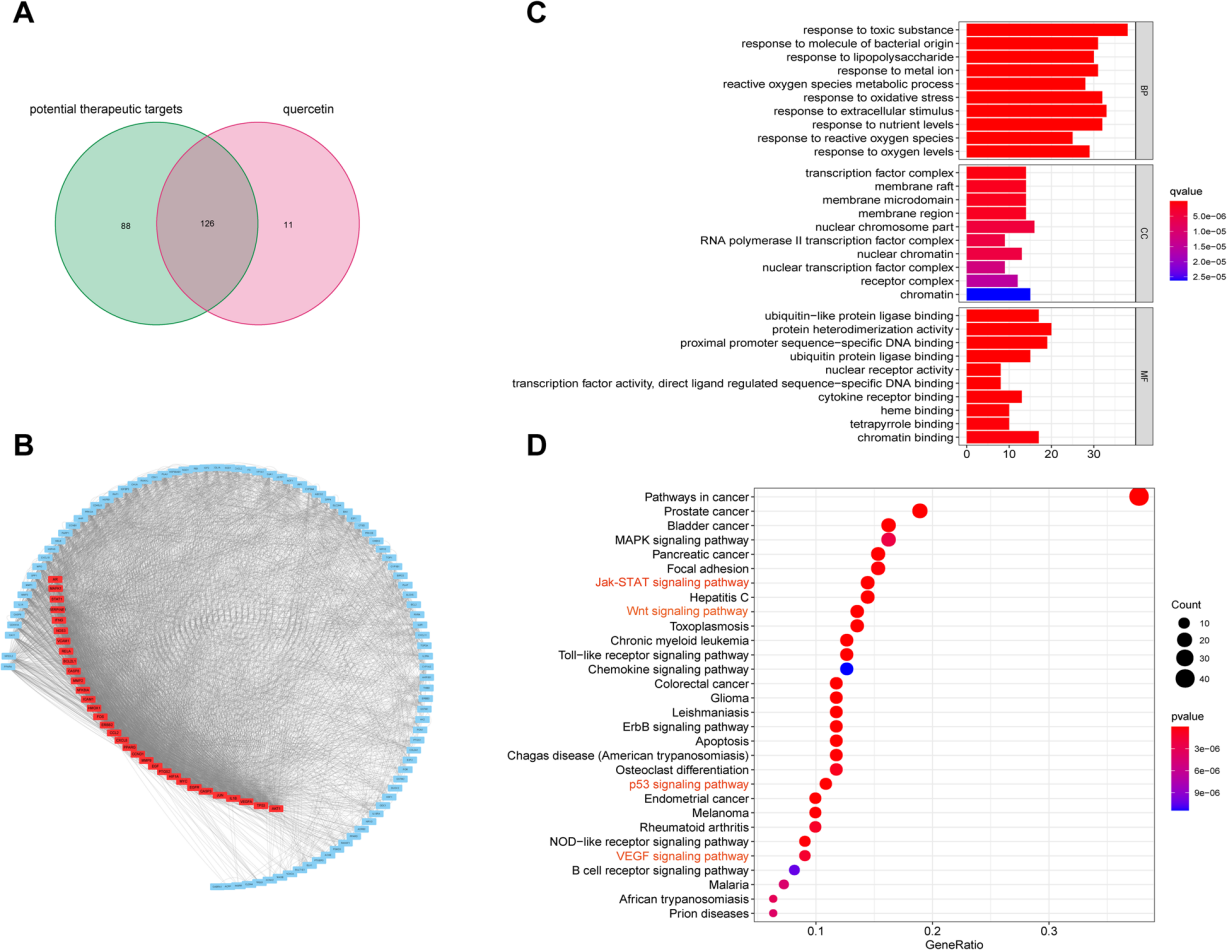
Bioinformatic analysis of quercetin’s therapeutic targets. **A** The intersection of quercetin and JQH therapeutic targets. **B** PPI analysis of 126 therapeutic targets. **C** GO annotation of 126 therapeutic targets. **D** KEGG analysis of 126 therapeutic targets

By intersecting the targets of active JQH ingredients with the 214 therapeutic targets, we determined that quercetin might be the core ingredient of JQH in the treatment of gastric inflammation-cancer transformation. We found 126 overlapped targets of quercetin and the potential therapeutic targets of gastric inflammation-cancer transformation (Fig. 8A and Table S9). The PPI analysis showed that the top 32 core genes were almost identical to those of JQH (Fig. 8B). GO annotation showed that the higher repetition rate of quercetin and JQH in BP, CC and MF items (Fig. 8C). The KEGG analysis showed that the repetition rate of quercetin and JQH pathways were nearly 90%, including JAK-STAT, Wnt, p53 and VEGF signaling pathways (Fig. 8D).

### Validation with in vitro experiments

**Fig. 9:**
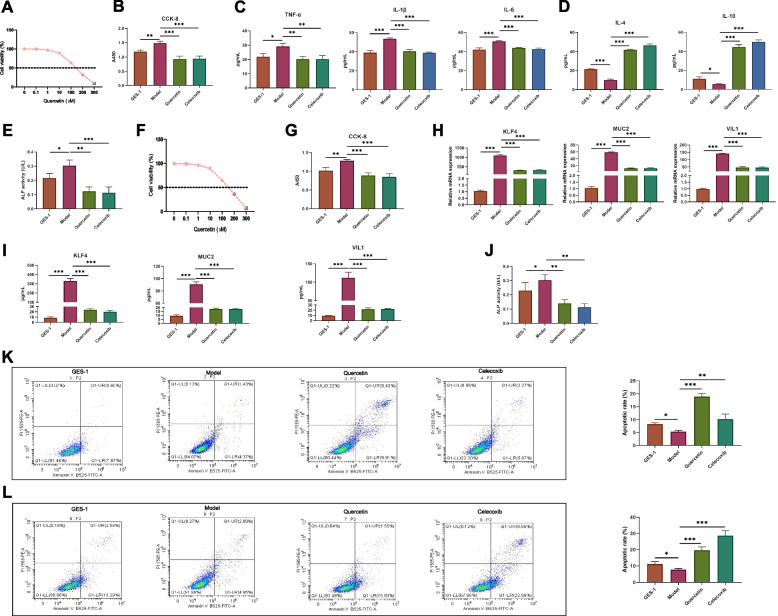
Quercetin inhibited inflammation and intestinal metaplasia levels in MNNG and CDCA-induced GES-1 cells. **A** IC_50_ of MNNG-GES-1 cells after quercetin treatment for 24 h. **B** Cell viability of MNNG-GES-1 cells after quercetin treatment for 24 h in CCK-8 assay. **C** TNF-α, IL-1β, and IL-6 expression of MNNG-GES-1 cells after quercetin treatment for 24 h by ELISA. **D** IL-4 and IL-10 expression of MNNG-GES-1 cells after quercetin treatment for 24 h by ELISA. **E** ALP activity of MNNG-GES-1 cells after quercetin treatment for 24 h. **F** IC_50_ of CDCA-GES-1 cells after quercetin treatment for 24 h. **G** Cell viability of CDCA-GES-1 cells after quercetin treatment for 24 h in CCK-8 assay. **H** KLF4, MUC2, and VIL1 expression in CDCA-GES-1 cells after quercetin treatment for 24 h by qPCR. **I** MUC2, and VIL1 expression of CDCA-GES-1 cells after quercetin treatment for 24 h by ELISA. **J** ALP activity of CDCA-GES-1 cells after quercetin treatment for 24h. Apoptosis of (**K**) MNNG-GES-1 and (**L**) CDCA-GES-1 cells after quercetin treatment for 24 h by flow cytometry. The mean ± standard deviation was used to express all data, and there were at least three samples in each group. The analysis of variance (ANOVA) methods were performed for comparisons between groups. *P*-values < 0.05 denoted statistically significant differences. *p < 0.05, **p < 0.01, ***p < 0.001

Furthermore, we found that quercetin was the active ingredient of JQH using LC-Q-TOF–MS and GC–MS [12]. Therefore, various functional experiments were conducted to validate the network pharmacology results. GES-1 cells were treated with MNNG and CDCA to construct an inflammatory [13] and intestinal metaplasia [14] cell model. Then, we detected the effect of quercetin on the proliferation of MNNG-GES-1 and CDCA-GES-1 cells in vitro. Quercetin inhibited cells proliferation with an IC_50_ of about 140 μM (Fig. 9A and B). MNNG was used to construct an inflammatory cell model, and specific inflammatory cytokines in cell supernatant were analyzed by ELISA. After quercetin treatment, TNF-α, IL-1β, and IL-6 levels significantly decreased compared to the model group in MNNG-GES-1 cells (*P*_TNF-α_ < 0.01, *P*_IL-1β_ < 0.001, *P*_IL-6_ < 0.001; Fig. 9C), and IL-4 and IL-10 levels significantly increased compared to the model group in MNNG-GES-1 cells (*P*_IL-4_ < 0.001, *P*_IL-10_ < 0.001; Fig. 9D). We also investigated whether quercetin treatment affected MNNG-GES-1 cell differentiation by detecting ALP activity. ALP is often positive in GC cells and negative in normal gastric cells [15]. ALP activity significantly decreased after quercetin treatment (*P* < 0.01; Fig. 9E), inducing the differentiation of GC cells. Moreover, we conducted flow cytometry analysis to investigate cell apoptosis after quercetin intervention. The quercetin intervention significantly promoted apoptosis of MNNG-GES-1 cells (*P* < 0.001; Fig. 9K).

Moreover, we found that quercetin inhibited CDCA-GES-1 cell proliferation with an IC_50_ of about 150 μM (Fig. 9F and G). Then, we conducted RT-qPCR to investigate the mRNA expression of several intestinal markers after quercetin intervention. The quercetin intervention significantly reduced mRNA(*P*_KLF4_ < 0.001, *P*_MUC2_ < 0.001, *P*_VIL1_ < 0.001; Fig. 9H) and protein level (*P*_KLF4_ < 0.001, *P*_MUC2_ < 0.001, *P*_VIL1_ < 0.001; Fig. 9I) of KLF4, MUC2, and VIL1 in CDCA-GES-1 cells. Similarly, after quercetin treatment, ALP activity significantly decreased in CDCA-GES-1 cells (*P* < 0.01; Fig. 9J). The flow cytometry analysis showed that quercetin intervention significantly promoted the apoptosis of CDCA-GES-1 cells (*P* < 0.001; Fig. 9K).

## Discussion

JianPi QingRe HuaYu, a frequently-used Traditional Chinese Medicine, is often used for treatment of CAG and precancerous lesions of gastric cancer (PLGC) in clinic [7]. Clinical and animal studies have been conducted to investigate the correlation between JQH and CAG or PLGC. Our clinical studies showed that JQH significantly improved mental and digestive function and reduced the inflammatory level, atrophy, and intestinal metaplasia of CAG patients [8]. JQH can also reduce the intestinal metaplasia and atypical hyperplasia of PLGC patients [16]. Our animal experiments showed that JQH could improve the ultrastructure of chief and parietal cells of the gastric mucosa to a certain extent [9]. However, examining the features of multi-ingredients and targets of TCM formulations is challenging. Nevertheless, network pharmacology allows the study of the molecular features of TCM formulations.

Herein, we obtained 214 potential therapeutic targets by intersecting JQH and gastric inflammation-cancer transformation targets. The KEGG enrichment analysis showed that the potential mechanisms of JQH in the treatment of gastric inflammation-cancer transformation might be related to JAK-STAT, Wnt, p53 and VEGF signaling pathways. STAT3, a protein composed of 770 amino acids, plays a crucial role in intracellular signal transduction [17]. STAT3 signaling pathway is a "bridge" between inflammation and cancer, and is closely related to "inflammation-cancer" transformation [17]. Zhang et al. [18] found that TRIM27 mediated the activation of STAT3 through retromer-positive structures and promoted colitis cancer. Long non-coding RNA (lncRNA) FAM64A promotes Th17 differentiation and colitis-related tumor formation by positively regulating STAT3 activity [19]. Zhang et al. found [20] that CKLF1 promotes inflammatory mediated hepatocellular carcinoma formation by activating the IL6/STAT3 signaling pathway, and can block adriamycin induced apoptosis. Anther study found [21] STAT3 signaling, mediated by TFF1 silencing, promotes gastric inflammation-cancer transformation. Activation of Wnt is highly correlated with gastric inflammation-cancer transformation. For examples, activation of Wnt/β-catenin signaling pathway promotes epithelial mesenchymal transition of MNNG-GES-1 cells and gastric precancerous lesions in rats [22]. Wnt activation also accelerate the progression of atrophic gastritis, and TCM may inhibit the these effection [23, 24]. The correlation between the P53 pathway and gastritis or GC has also been explored. P53 can mediate apoptotic and gastric carcinogenesis by targeting EPSIN3 [25]. P53 degradation, induced by USF1 defect, accelerates gastric carcinogenesis when rats are infected by *Helicobacter pylori* [26]. VEGF is a subfamily of growth factors, specifically belonging to the platelet-derived growth factor (PDGF) family of cystine node growth factors. Activation of HIF-1α/VEGF angiogenesis pathway promotes the development of MNNG-induced atrophic gastritis [27].

Subsequently, we identified 32 core genes by PPI and CytoNCA in Cytoscape. Most genes were significantly correlated with gastric inflammation-cancer transformation, such as AKT1, EGFR, and MYC. For example, Akt can promote gastric tumorigenesis by causing PTEN deficiency [28]. AKT1, suppressed by RUNX3, inhibits gastric tumorigenesis in GC [29]. EGFR methylation might promote the transformation of chronic gastritis into gastric carcinoma [30]. Additionally, exosome-delivered EGFR promoter liver metastasis by regulating the liver microenvironment in GC [31]. Another study found [32] that EGFR degradation, induced by CMTM3, inhibited GC tumorigenesis by enhancing Rab5 activity. MYC, a classic oncogene, is significantly correlated with gastric inflammation-cancer transformation. Zheng et al. [33] found that c-MYC upregulation, induced by CHAF1A and TCF4, promotes gastric carcinogenesis. Besides, c-Myc upregulation, caused by microRNA-10b/CSMD1 axis activation, promotes the inflammation-carcinogenesis of GC [34].

Finally, the molecular docking and core ingredients analysis showed that quercetin was the core ingredient of JQH and bound well with several inflammation-cancer targets, including AKT1, EGFR, HIF1A, and IL6A. Our validated experiments indicated that quercetin inhibited cell proliferation, promoted cell apoptosis, and decreased inflammation and intestinal metaplasia levels in cell models of inflammation and intestinal metaplasia in vitro. Additionally, Zhang et al. [35] found that quercetin can ameliorate gastric inflammation by regulating p38MAPK and BCL-2 expression. Hsieh et al. demonstrated that quercetin exerts anti-inflammatory effects by inhibiting the TNF/MMP9 axis in GES-1 cells [36]. Yu et al. found [37] that quercetin can inhibit the IRF8/IFN-γ axis, reduce gastric inflammation, and enhance gastric secretory function, improving CAG induced by Hp infection. Another study [38] found that quercetin induced GC cell apoptosis and exerted potential anti-gastric cancer efficacy. Moreover, quercetin enhances the efficacy of other anti-cancer drugs in vitro and in vivo for GC [39].

However, our current study also has some limitations. First, some compounds of JQH were not well investigated, and we only explored the function of quercetin on gastric inflammation-cancer transformation. Then, we did not investigate the effects of JQH and its compounds on gastric inflammation-cancer transformation in animal experiments. We will conduct further experiments to validate the above mechanisms of JQH or its compounds.

In conclusion, we elucidated the potential molecular mechanisms of JQH in treating gastric inflammation-cancer transformation using network pharmacology, bioinformatics, and in vitro experiments for validation. Moreover, quercetin might be one of the active ingredients of JQH that exerted drug efficacy in gastric inflammation-cancer transformation. We provided robust evidence for the clinical application of JQH in gastric inflammation-cancer transformation, but further in vivo experiments are needed to validate these findings.

### Supplementary Information

**Additional file 1:** **Table S1.** Sequences of primers used in the RT-qPCR.

**Additional file 2: Table S2.** The active ingredients and potential targets of JQH in TCMSP database.

**Additional file 3: Table S3.** The DEGs between IN and gastritis group In GSE130823 dataset.

**Additional file 4: Table S4.** The DEGs between IGC and gastritis group In GSE130823 dataset.

**Additional file 5:** **Table S5.** The DEGs between IN and chronic gastritis group in GSE55696 dataset.

**Additional file 6:** **Table S6.** The DEGs between EGC and chronic gastritis group in GSE55696 dataset.

**Additional file 7:** **Table S7.** Binding energy (kcal/mol) of quercetin target proteins.

**Additional file 8: Table S8.** Interaction between ligands and target proteins.

**Additional file 9:** **Table S9.** The potential therapeutic targets for JQH and Quercetin in the treatments of gastric inflammation-cancer transformation.

## Data Availability

The datasets used and/or analyzed during the current study are available from the corresponding author upon reasonable request. GSE130823 and GSE55696 datasets used and/or analyzed during the current study are available from https://www.ncbi.nlm.nih.gov/gds/.

## Abbreviations

JQH: JianPi QingRe HuaYu Methods
CAG: Chronic atrophic gastritis
PLGC: Precancerous lesions of gastric cancer
GC: Gastric cancer
DEGs: Differentially expressed genes
GEO: Gene Expression Omnibus
GO: Gene Ontology
KEGG: Kyoto Encyclopedia of Genes and Genomes
PPI: Protein–protein interaction
TCM: Traditional Chinese Medicine
IN: Intraepithelial neoplasia
IGC: Intestinal gastric cancer
EGC: Early gastric cancer
MNNG: N-methyl-N'-nitro-N-nitrosoguanidine
CDCA: Chenodeoxycholic acid
ELISA: Enzyme-Linked Immunosorbent Assay
ALP: Alkaline phosphatase
BP: Biological Process
CC: Cellular Components
MF: Molecular Function
