# Supplementary material for: Explore the active ingredients and potential mechanisms of JianPi QingRe HuaYu Methods in the treatment of gastric inflammation-cancer transformation by network pharmacology and experimental validation
Source: BMC Complement Med Ther. 2023 Nov 14;23:411. doi: 10.1186/s12906-023-04232-0 (PMC10644588; doi:10.1186/s12906-023-04232-0)
Supplement: Supplementary file 1 — Additional file 1: Table S1. Sequences of primers used in the RT-qPCR. [file 12906_2023_4232_MOESM1_ESM.docx]

**Table S1. Sequences of primers used in the RT-qPCR.**

| Primers | Forward sequence (5’→3’) | Reverse sequence (5’→3’) |
| --- | --- | --- |
| KLF4 | CCCACATGAAGCGACTTCCC | CAGGTCCAGGAGATCGTTGAA |
| MUC2 | GAGGGCAGAACCCGAAACC | GGCGAAGTTGTAGTCGCAGAG |
| VIL1 | CTGAGCGCCCAAGTCAAAG | AGCAGTCACCATCGAAGAAGC |
| GAPDH | GGACCTGACCTGCCGTCTAG | GTAGCCCAGGATGCCCTTGA |
