# Supplementary material for: Explore the active ingredients and potential mechanisms of JianPi QingRe HuaYu Methods in the treatment of gastric inflammation-cancer transformation by network pharmacology and experimental validation
Source: BMC Complement Med Ther. 2023 Nov 14;23:411. doi: 10.1186/s12906-023-04232-0 (PMC10644588; doi:10.1186/s12906-023-04232-0)
Supplement: Supplementary file 2 — Additional file 2: Table S2. The active ingredients and potential targets of JQH in TCMSP database. [file 12906_2023_4232_MOESM2_ESM.docx]

**Table S2. The active ingredients and potential targets of JQH in TCMSP database.**

| Drug | MolId | MolName | Symbol |
| --- | --- | --- | --- |
| baihuasheshecao | MOL001659 | Poriferasterol | PGR |
| baihuasheshecao | MOL001659 | Poriferasterol | NR3C2 |
| baihuasheshecao | MOL001670 | 2-methoxy-3-methyl-9,10-anthraquinone | PTGS1 |
| baihuasheshecao | MOL001670 | 2-methoxy-3-methyl-9,10-anthraquinone | DRD1 |
| baihuasheshecao | MOL001670 | 2-methoxy-3-methyl-9,10-anthraquinone | CHRM3 |
| baihuasheshecao | MOL001670 | 2-methoxy-3-methyl-9,10-anthraquinone | CHRM1 |
| baihuasheshecao | MOL001670 | 2-methoxy-3-methyl-9,10-anthraquinone | ESR1 |
| baihuasheshecao | MOL001670 | 2-methoxy-3-methyl-9,10-anthraquinone | SCN5A |
| baihuasheshecao | MOL001670 | 2-methoxy-3-methyl-9,10-anthraquinone | CHRM5 |
| baihuasheshecao | MOL001670 | 2-methoxy-3-methyl-9,10-anthraquinone | PTGS2 |
| baihuasheshecao | MOL001670 | 2-methoxy-3-methyl-9,10-anthraquinone | CHRM4 |
| baihuasheshecao | MOL001670 | 2-methoxy-3-methyl-9,10-anthraquinone | RXRA |
| baihuasheshecao | MOL001670 | 2-methoxy-3-methyl-9,10-anthraquinone | OPRD1 |
| baihuasheshecao | MOL001670 | 2-methoxy-3-methyl-9,10-anthraquinone | HRH1 |
| baihuasheshecao | MOL001670 | 2-methoxy-3-methyl-9,10-anthraquinone | SLC6A2 |
| baihuasheshecao | MOL001670 | 2-methoxy-3-methyl-9,10-anthraquinone | ADRA1A |
| baihuasheshecao | MOL001670 | 2-methoxy-3-methyl-9,10-anthraquinone | CHRM2 |
| baihuasheshecao | MOL001670 | 2-methoxy-3-methyl-9,10-anthraquinone | ADRA1B |
| baihuasheshecao | MOL001670 | 2-methoxy-3-methyl-9,10-anthraquinone | SLC6A3 |
| baihuasheshecao | MOL001670 | 2-methoxy-3-methyl-9,10-anthraquinone | ADRB2 |
| baihuasheshecao | MOL001670 | 2-methoxy-3-methyl-9,10-anthraquinone | ADRA1D |
| baihuasheshecao | MOL001670 | 2-methoxy-3-methyl-9,10-anthraquinone | SLC6A4 |
| baihuasheshecao | MOL001670 | 2-methoxy-3-methyl-9,10-anthraquinone | OPRM1 |
| baihuasheshecao | MOL001670 | 2-methoxy-3-methyl-9,10-anthraquinone | GABRA1 |
| baihuasheshecao | MOL001670 | 2-methoxy-3-methyl-9,10-anthraquinone | HSP90AB1 |
| baihuasheshecao | MOL001670 | 2-methoxy-3-methyl-9,10-anthraquinone | NCOA2 |
| baihuasheshecao | MOL001670 | 2-methoxy-3-methyl-9,10-anthraquinone | PKIA |
| baihuasheshecao | MOL000449 | Stigmasterol | PGR |
| baihuasheshecao | MOL000449 | Stigmasterol | NR3C2 |
| baihuasheshecao | MOL000449 | Stigmasterol | NCOA2 |
| baihuasheshecao | MOL000449 | Stigmasterol | ADH1C |
| baihuasheshecao | MOL000449 | Stigmasterol | IGHG1 |
| baihuasheshecao | MOL000449 | Stigmasterol | RXRA |
| baihuasheshecao | MOL000449 | Stigmasterol | NCOA1 |
| baihuasheshecao | MOL000449 | Stigmasterol | PTGS1 |
| baihuasheshecao | MOL000449 | Stigmasterol | PTGS2 |
| baihuasheshecao | MOL000449 | Stigmasterol | ADRA2A |
| baihuasheshecao | MOL000449 | Stigmasterol | SLC6A2 |
| baihuasheshecao | MOL000449 | Stigmasterol | SLC6A3 |
| baihuasheshecao | MOL000449 | Stigmasterol | ADRB2 |
| baihuasheshecao | MOL000449 | Stigmasterol | AKR1B1 |
| baihuasheshecao | MOL000449 | Stigmasterol | PLAU |
| baihuasheshecao | MOL000449 | Stigmasterol | LTA4H |
| baihuasheshecao | MOL000449 | Stigmasterol | MAOB |
| baihuasheshecao | MOL000449 | Stigmasterol | MAOA |
| baihuasheshecao | MOL000449 | Stigmasterol | CTRB1 |
| baihuasheshecao | MOL000449 | Stigmasterol | CHRM3 |
| baihuasheshecao | MOL000449 | Stigmasterol | CHRM1 |
| baihuasheshecao | MOL000449 | Stigmasterol | ADRB1 |
| baihuasheshecao | MOL000449 | Stigmasterol | SCN5A |
| baihuasheshecao | MOL000449 | Stigmasterol | ADRA1A |
| baihuasheshecao | MOL000449 | Stigmasterol | CHRM2 |
| baihuasheshecao | MOL000449 | Stigmasterol | ADRA1B |
| baihuasheshecao | MOL000449 | Stigmasterol | GABRA1 |
| baihuasheshecao | MOL000358 | beta-sitosterol | PGR |
| baihuasheshecao | MOL000358 | beta-sitosterol | NCOA2 |
| baihuasheshecao | MOL000358 | beta-sitosterol | PTGS1 |
| baihuasheshecao | MOL000358 | beta-sitosterol | PTGS2 |
| baihuasheshecao | MOL000358 | beta-sitosterol | HSP90AB1 |
| baihuasheshecao | MOL000358 | beta-sitosterol | KCNH2 |
| baihuasheshecao | MOL000358 | beta-sitosterol | DRD1 |
| baihuasheshecao | MOL000358 | beta-sitosterol | CHRM3 |
| baihuasheshecao | MOL000358 | beta-sitosterol | CHRM1 |
| baihuasheshecao | MOL000358 | beta-sitosterol | SCN5A |
| baihuasheshecao | MOL000358 | beta-sitosterol | CHRM4 |
| baihuasheshecao | MOL000358 | beta-sitosterol | ADRA1A |
| baihuasheshecao | MOL000358 | beta-sitosterol | CHRM2 |
| baihuasheshecao | MOL000358 | beta-sitosterol | ADRA1B |
| baihuasheshecao | MOL000358 | beta-sitosterol | ADRB2 |
| baihuasheshecao | MOL000358 | beta-sitosterol | CHRNA2 |
| baihuasheshecao | MOL000358 | beta-sitosterol | SLC6A4 |
| baihuasheshecao | MOL000358 | beta-sitosterol | OPRM1 |
| baihuasheshecao | MOL000358 | beta-sitosterol | GABRA1 |
| baihuasheshecao | MOL000358 | beta-sitosterol | BCL2 |
| baihuasheshecao | MOL000358 | beta-sitosterol | BAX |
| baihuasheshecao | MOL000358 | beta-sitosterol | CASP9 |
| baihuasheshecao | MOL000358 | beta-sitosterol | JUN |
| baihuasheshecao | MOL000358 | beta-sitosterol | CASP3 |
| baihuasheshecao | MOL000358 | beta-sitosterol | CASP8 |
| baihuasheshecao | MOL000358 | beta-sitosterol | PRKCA |
| baihuasheshecao | MOL000358 | beta-sitosterol | PON1 |
| baihuasheshecao | MOL000358 | beta-sitosterol | MAP2 |
| baihuasheshecao | MOL000098 | quercetin | PTGS1 |
| baihuasheshecao | MOL000098 | quercetin | AR |
| baihuasheshecao | MOL000098 | quercetin | PPARG |
| baihuasheshecao | MOL000098 | quercetin | PTGS2 |
| baihuasheshecao | MOL000098 | quercetin | HSP90AB1 |
| baihuasheshecao | MOL000098 | quercetin | NCOA2 |
| baihuasheshecao | MOL000098 | quercetin | DPP4 |
| baihuasheshecao | MOL000098 | quercetin | AKR1B1 |
| baihuasheshecao | MOL000098 | quercetin | PRSS1 |
| baihuasheshecao | MOL000098 | quercetin | KCNH2 |
| baihuasheshecao | MOL000098 | quercetin | SCN5A |
| baihuasheshecao | MOL000098 | quercetin | ADRB2 |
| baihuasheshecao | MOL000098 | quercetin | MMP3 |
| baihuasheshecao | MOL000098 | quercetin | F7 |
| baihuasheshecao | MOL000098 | quercetin | RXRA |
| baihuasheshecao | MOL000098 | quercetin | ACHE |
| baihuasheshecao | MOL000098 | quercetin | GABRA1 |
| baihuasheshecao | MOL000098 | quercetin | MAOB |
| baihuasheshecao | MOL000098 | quercetin | RELA |
| baihuasheshecao | MOL000098 | quercetin | EGFR |
| baihuasheshecao | MOL000098 | quercetin | AKT1 |
| baihuasheshecao | MOL000098 | quercetin | VEGFA |
| baihuasheshecao | MOL000098 | quercetin | CCND1 |
| baihuasheshecao | MOL000098 | quercetin | BCL2 |
| baihuasheshecao | MOL000098 | quercetin | BCL2L1 |
| baihuasheshecao | MOL000098 | quercetin | FOS |
| baihuasheshecao | MOL000098 | quercetin | CDKN1A |
| baihuasheshecao | MOL000098 | quercetin | EIF6 |
| baihuasheshecao | MOL000098 | quercetin | BAX |
| baihuasheshecao | MOL000098 | quercetin | CASP9 |
| baihuasheshecao | MOL000098 | quercetin | PLAU |
| baihuasheshecao | MOL000098 | quercetin | MMP2 |
| baihuasheshecao | MOL000098 | quercetin | MMP9 |
| baihuasheshecao | MOL000098 | quercetin | MAPK1 |
| baihuasheshecao | MOL000098 | quercetin | IL10RA |
| baihuasheshecao | MOL000098 | quercetin | EGF |
| baihuasheshecao | MOL000098 | quercetin | RB1 |
| baihuasheshecao | MOL000098 | quercetin | TNFAIP6 |
| baihuasheshecao | MOL000098 | quercetin | JUN |
| baihuasheshecao | MOL000098 | quercetin | IL6R |
| baihuasheshecao | MOL000098 | quercetin | AHSA1 |
| baihuasheshecao | MOL000098 | quercetin | CASP3 |
| baihuasheshecao | MOL000098 | quercetin | TP53 |
| baihuasheshecao | MOL000098 | quercetin | ELK1 |
| baihuasheshecao | MOL000098 | quercetin | NFKBIA |
| baihuasheshecao | MOL000098 | quercetin | POR |
| baihuasheshecao | MOL000098 | quercetin | ODC1 |
| baihuasheshecao | MOL000098 | quercetin | CASP8 |
| baihuasheshecao | MOL000098 | quercetin | TOP1 |
| baihuasheshecao | MOL000098 | quercetin | RAF1 |
| baihuasheshecao | MOL000098 | quercetin | SOD1 |
| baihuasheshecao | MOL000098 | quercetin | PRKCA |
| baihuasheshecao | MOL000098 | quercetin | MMP1 |
| baihuasheshecao | MOL000098 | quercetin | HIF1A |
| baihuasheshecao | MOL000098 | quercetin | STAT1 |
| baihuasheshecao | MOL000098 | quercetin | RUNX1T1 |
| baihuasheshecao | MOL000098 | quercetin | CDK1 |
| baihuasheshecao | MOL000098 | quercetin | HSPA5 |
| baihuasheshecao | MOL000098 | quercetin | ERBB2 |
| baihuasheshecao | MOL000098 | quercetin | PPARG |
| baihuasheshecao | MOL000098 | quercetin | ACACA |
| baihuasheshecao | MOL000098 | quercetin | HMOX1 |
| baihuasheshecao | MOL000098 | quercetin | CYP3A4 |
| baihuasheshecao | MOL000098 | quercetin | CYP1A2 |
| baihuasheshecao | MOL000098 | quercetin | CAV1 |
| baihuasheshecao | MOL000098 | quercetin | MYC |
| baihuasheshecao | MOL000098 | quercetin | F3 |
| baihuasheshecao | MOL000098 | quercetin | GJA1 |
| baihuasheshecao | MOL000098 | quercetin | CYP1A1 |
| baihuasheshecao | MOL000098 | quercetin | ICAM1 |
| baihuasheshecao | MOL000098 | quercetin | IL1B |
| baihuasheshecao | MOL000098 | quercetin | CCL2 |
| baihuasheshecao | MOL000098 | quercetin | SELE |
| baihuasheshecao | MOL000098 | quercetin | VCAM1 |
| baihuasheshecao | MOL000098 | quercetin | PTGER3 |
| baihuasheshecao | MOL000098 | quercetin | CXCL8 |
| baihuasheshecao | MOL000098 | quercetin | PRKCB |
| baihuasheshecao | MOL000098 | quercetin | BIRC5 |
| baihuasheshecao | MOL000098 | quercetin | DUOX2 |
| baihuasheshecao | MOL000098 | quercetin | NOS3 |
| baihuasheshecao | MOL000098 | quercetin | HSPB1 |
| baihuasheshecao | MOL000098 | quercetin | SULT1E1 |
| baihuasheshecao | MOL000098 | quercetin | IL2RA |
| baihuasheshecao | MOL000098 | quercetin | NR1I2 |
| baihuasheshecao | MOL000098 | quercetin | CYP1B1 |
| baihuasheshecao | MOL000098 | quercetin | CCNB1 |
| baihuasheshecao | MOL000098 | quercetin | PLAT |
| baihuasheshecao | MOL000098 | quercetin | THBD |
| baihuasheshecao | MOL000098 | quercetin | SERPINE1 |
| baihuasheshecao | MOL000098 | quercetin | COL1A1 |
| baihuasheshecao | MOL000098 | quercetin | IFNG |
| baihuasheshecao | MOL000098 | quercetin | ALOX5 |
| baihuasheshecao | MOL000098 | quercetin | IL1A |
| baihuasheshecao | MOL000098 | quercetin | MPO |
| baihuasheshecao | MOL000098 | quercetin | TOP2A |
| baihuasheshecao | MOL000098 | quercetin | NCF1 |
| baihuasheshecao | MOL000098 | quercetin | ABCG2 |
| baihuasheshecao | MOL000098 | quercetin | HAS2 |
| baihuasheshecao | MOL000098 | quercetin | GSTP1 |
| baihuasheshecao | MOL000098 | quercetin | NFE2L2 |
| baihuasheshecao | MOL000098 | quercetin | NQO1 |
| baihuasheshecao | MOL000098 | quercetin | PARP1 |
| baihuasheshecao | MOL000098 | quercetin | AHR |
| baihuasheshecao | MOL000098 | quercetin | PSMD3 |
| baihuasheshecao | MOL000098 | quercetin | SLC2A4 |
| baihuasheshecao | MOL000098 | quercetin | COL3A1 |
| baihuasheshecao | MOL000098 | quercetin | CXCL11 |
| baihuasheshecao | MOL000098 | quercetin | CXCL2 |
| baihuasheshecao | MOL000098 | quercetin | DCAF5 |
| baihuasheshecao | MOL000098 | quercetin | NR1I3 |
| baihuasheshecao | MOL000098 | quercetin | CHEK2 |
| baihuasheshecao | MOL000098 | quercetin | INSRR |
| baihuasheshecao | MOL000098 | quercetin | CLDN4 |
| baihuasheshecao | MOL000098 | quercetin | PPARA |
| baihuasheshecao | MOL000098 | quercetin | PPARD |
| baihuasheshecao | MOL000098 | quercetin | HSF1 |
| baihuasheshecao | MOL000098 | quercetin | CXCL10 |
| baihuasheshecao | MOL000098 | quercetin | CHUK |
| baihuasheshecao | MOL000098 | quercetin | SPP1 |
| baihuasheshecao | MOL000098 | quercetin | RUNX2 |
| baihuasheshecao | MOL000098 | quercetin | RASSF1 |
| baihuasheshecao | MOL000098 | quercetin | E2F1 |
| baihuasheshecao | MOL000098 | quercetin | E2F2 |
| baihuasheshecao | MOL000098 | quercetin | ACP3 |
| baihuasheshecao | MOL000098 | quercetin | CTSD |
| baihuasheshecao | MOL000098 | quercetin | IGFBP3 |
| baihuasheshecao | MOL000098 | quercetin | IGF2 |
| baihuasheshecao | MOL000098 | quercetin | CD40LG |
| baihuasheshecao | MOL000098 | quercetin | IRF1 |
| baihuasheshecao | MOL000098 | quercetin | ERBB3 |
| baihuasheshecao | MOL000098 | quercetin | PON1 |
| baihuasheshecao | MOL000098 | quercetin | DIO1 |
| baihuasheshecao | MOL000098 | quercetin | PCOLCE |
| baihuasheshecao | MOL000098 | quercetin | NPEPPS |
| baihuasheshecao | MOL000098 | quercetin | HK2 |
| baihuasheshecao | MOL000098 | quercetin | RASA1 |
| baihuasheshecao | MOL000098 | quercetin | GSTM1 |
| baihuasheshecao | MOL000098 | quercetin | GSTM2 |
| baizhu | MOL000022 | 14-acetyl-12-senecioyl-2E,8Z,10E-atractylentriol | PTGS2 |
| baizhu | MOL000033 | (3S,8S,9S,10R,13R,14S,17R)-10,13-dimethyl-17-[(2R,5S)-5-propan-2-yloctan-2-yl]-2,3,4,7,8,9,11,12,14,15,16,17-dodecahydro-1H-cyclopenta[a]phenanthren-3-ol | PGR |
| baizhu | MOL000049 | 3尾-acetoxyatractylone | CHRM3 |
| baizhu | MOL000049 | 3尾-acetoxyatractylone | CHRM1 |
| baizhu | MOL000049 | 3尾-acetoxyatractylone | AR |
| baizhu | MOL000049 | 3尾-acetoxyatractylone | SCN5A |
| baizhu | MOL000049 | 3尾-acetoxyatractylone | PTGS2 |
| baizhu | MOL000049 | 3尾-acetoxyatractylone | RXRA |
| baizhu | MOL000049 | 3尾-acetoxyatractylone | ACHE |
| baizhu | MOL000049 | 3尾-acetoxyatractylone | ADRA1A |
| baizhu | MOL000049 | 3尾-acetoxyatractylone | CHRM2 |
| baizhu | MOL000049 | 3尾-acetoxyatractylone | ADRB2 |
| baizhu | MOL000049 | 3尾-acetoxyatractylone | OPRM1 |
| baizhu | MOL000049 | 3尾-acetoxyatractylone | GABRA1 |
| baizhu | MOL000049 | 3尾-acetoxyatractylone | DPP4 |
| baizhu | MOL000072 | 8β-ethoxy atractylenolide Ⅲ | PTGS2 |
| baizhu | MOL000072 | 8β-ethoxy atractylenolide Ⅲ | GABRA1 |
| baizhu | MOL000072 | 8β-ethoxy atractylenolide Ⅲ | NCOA2 |
| baizhu | MOL000072 | 8β-ethoxy atractylenolide Ⅲ | NCOA1 |
| banzhilian | MOL001040 | (2R)-5,7-dihydroxy-2-(4-hydroxyphenyl)chroman-4-one | PTGS1 |
| banzhilian | MOL001040 | (2R)-5,7-dihydroxy-2-(4-hydroxyphenyl)chroman-4-one | ESR1 |
| banzhilian | MOL001040 | (2R)-5,7-dihydroxy-2-(4-hydroxyphenyl)chroman-4-one | PTGS2 |
| banzhilian | MOL001040 | (2R)-5,7-dihydroxy-2-(4-hydroxyphenyl)chroman-4-one | HSP90AB1 |
| banzhilian | MOL001040 | (2R)-5,7-dihydroxy-2-(4-hydroxyphenyl)chroman-4-one | DPEP1 |
| banzhilian | MOL001040 | (2R)-5,7-dihydroxy-2-(4-hydroxyphenyl)chroman-4-one | PGR |
| banzhilian | MOL001040 | (2R)-5,7-dihydroxy-2-(4-hydroxyphenyl)chroman-4-one | NR3C2 |
| banzhilian | MOL001040 | (2R)-5,7-dihydroxy-2-(4-hydroxyphenyl)chroman-4-one | NR3C1 |
| banzhilian | MOL012245 | 5,7,4'-trihydroxy-6-methoxyflavanone | PTGS1 |
| banzhilian | MOL012245 | 5,7,4'-trihydroxy-6-methoxyflavanone | PTGS2 |
| banzhilian | MOL012245 | 5,7,4'-trihydroxy-6-methoxyflavanone | CA2 |
| banzhilian | MOL012245 | 5,7,4'-trihydroxy-6-methoxyflavanone | HSP90AB1 |
| banzhilian | MOL012245 | 5,7,4'-trihydroxy-6-methoxyflavanone | CAMKMT |
| banzhilian | MOL012246 | 5,7,4'-trihydroxy-8-methoxyflavanone | PTGS1 |
| banzhilian | MOL012246 | 5,7,4'-trihydroxy-8-methoxyflavanone | PTGS2 |
| banzhilian | MOL012246 | 5,7,4'-trihydroxy-8-methoxyflavanone | CA2 |
| banzhilian | MOL012246 | 5,7,4'-trihydroxy-8-methoxyflavanone | HSP90AB1 |
| banzhilian | MOL012248 | 5-hydroxy-7,8-dimethoxy-2-(4-methoxyphenyl)chromone | NOS2 |
| banzhilian | MOL012248 | 5-hydroxy-7,8-dimethoxy-2-(4-methoxyphenyl)chromone | PTGS1 |
| banzhilian | MOL012248 | 5-hydroxy-7,8-dimethoxy-2-(4-methoxyphenyl)chromone | KCNH2 |
| banzhilian | MOL012248 | 5-hydroxy-7,8-dimethoxy-2-(4-methoxyphenyl)chromone | AR |
| banzhilian | MOL012248 | 5-hydroxy-7,8-dimethoxy-2-(4-methoxyphenyl)chromone | SCN5A |
| banzhilian | MOL012248 | 5-hydroxy-7,8-dimethoxy-2-(4-methoxyphenyl)chromone | PTGS2 |
| banzhilian | MOL012248 | 5-hydroxy-7,8-dimethoxy-2-(4-methoxyphenyl)chromone | CA2 |
| banzhilian | MOL012248 | 5-hydroxy-7,8-dimethoxy-2-(4-methoxyphenyl)chromone | ADRA1B |
| banzhilian | MOL012248 | 5-hydroxy-7,8-dimethoxy-2-(4-methoxyphenyl)chromone | ADRB2 |
| banzhilian | MOL012248 | 5-hydroxy-7,8-dimethoxy-2-(4-methoxyphenyl)chromone | ESR2 |
| banzhilian | MOL012248 | 5-hydroxy-7,8-dimethoxy-2-(4-methoxyphenyl)chromone | DPP4 |
| banzhilian | MOL012248 | 5-hydroxy-7,8-dimethoxy-2-(4-methoxyphenyl)chromone | PYGM |
| banzhilian | MOL012248 | 5-hydroxy-7,8-dimethoxy-2-(4-methoxyphenyl)chromone | GSK3B |
| banzhilian | MOL012248 | 5-hydroxy-7,8-dimethoxy-2-(4-methoxyphenyl)chromone | HSP90AB1 |
| banzhilian | MOL012248 | 5-hydroxy-7,8-dimethoxy-2-(4-methoxyphenyl)chromone | CDK2 |
| banzhilian | MOL012248 | 5-hydroxy-7,8-dimethoxy-2-(4-methoxyphenyl)chromone | CHEK1 |
| banzhilian | MOL012248 | 5-hydroxy-7,8-dimethoxy-2-(4-methoxyphenyl)chromone | IGHG1 |
| banzhilian | MOL012248 | 5-hydroxy-7,8-dimethoxy-2-(4-methoxyphenyl)chromone | PRSS1 |
| banzhilian | MOL012248 | 5-hydroxy-7,8-dimethoxy-2-(4-methoxyphenyl)chromone | NCOA2 |
| banzhilian | MOL012248 | 5-hydroxy-7,8-dimethoxy-2-(4-methoxyphenyl)chromone | NCOA1 |
| banzhilian | MOL012248 | 5-hydroxy-7,8-dimethoxy-2-(4-methoxyphenyl)chromone | CAMKMT |
| banzhilian | MOL012250 | 7-hydroxy-5,8-dimethoxy-2-phenyl-chromone | NOS2 |
| banzhilian | MOL012250 | 7-hydroxy-5,8-dimethoxy-2-phenyl-chromone | PTGS1 |
| banzhilian | MOL012250 | 7-hydroxy-5,8-dimethoxy-2-phenyl-chromone | ESR1 |
| banzhilian | MOL012250 | 7-hydroxy-5,8-dimethoxy-2-phenyl-chromone | AR |
| banzhilian | MOL012250 | 7-hydroxy-5,8-dimethoxy-2-phenyl-chromone | SCN5A |
| banzhilian | MOL012250 | 7-hydroxy-5,8-dimethoxy-2-phenyl-chromone | PPARG |
| banzhilian | MOL012250 | 7-hydroxy-5,8-dimethoxy-2-phenyl-chromone | PTGS2 |
| banzhilian | MOL012250 | 7-hydroxy-5,8-dimethoxy-2-phenyl-chromone | CA2 |
| banzhilian | MOL012250 | 7-hydroxy-5,8-dimethoxy-2-phenyl-chromone | RXRA |
| banzhilian | MOL012250 | 7-hydroxy-5,8-dimethoxy-2-phenyl-chromone | ADRA1B |
| banzhilian | MOL012250 | 7-hydroxy-5,8-dimethoxy-2-phenyl-chromone | ADRB2 |
| banzhilian | MOL012250 | 7-hydroxy-5,8-dimethoxy-2-phenyl-chromone | ADRA1D |
| banzhilian | MOL012250 | 7-hydroxy-5,8-dimethoxy-2-phenyl-chromone | ESR2 |
| banzhilian | MOL012250 | 7-hydroxy-5,8-dimethoxy-2-phenyl-chromone | DPP4 |
| banzhilian | MOL012250 | 7-hydroxy-5,8-dimethoxy-2-phenyl-chromone | MAPK14 |
| banzhilian | MOL012250 | 7-hydroxy-5,8-dimethoxy-2-phenyl-chromone | GSK3B |
| banzhilian | MOL012250 | 7-hydroxy-5,8-dimethoxy-2-phenyl-chromone | HSP90AB1 |
| banzhilian | MOL012250 | 7-hydroxy-5,8-dimethoxy-2-phenyl-chromone | CDK2 |
| banzhilian | MOL012250 | 7-hydroxy-5,8-dimethoxy-2-phenyl-chromone | CHEK1 |
| banzhilian | MOL012250 | 7-hydroxy-5,8-dimethoxy-2-phenyl-chromone | IGHG1 |
| banzhilian | MOL012250 | 7-hydroxy-5,8-dimethoxy-2-phenyl-chromone | PRSS1 |
| banzhilian | MOL012250 | 7-hydroxy-5,8-dimethoxy-2-phenyl-chromone | NCOA1 |
| banzhilian | MOL012250 | 7-hydroxy-5,8-dimethoxy-2-phenyl-chromone | CAMKMT |
| banzhilian | MOL012251 | Chrysin-5-methylether | NOS2 |
| banzhilian | MOL012251 | Chrysin-5-methylether | PTGS1 |
| banzhilian | MOL012251 | Chrysin-5-methylether | AR |
| banzhilian | MOL012251 | Chrysin-5-methylether | SCN5A |
| banzhilian | MOL012251 | Chrysin-5-methylether | PTGS2 |
| banzhilian | MOL012251 | Chrysin-5-methylether | CA2 |
| banzhilian | MOL012251 | Chrysin-5-methylether | RXRA |
| banzhilian | MOL012251 | Chrysin-5-methylether | ADRA1A |
| banzhilian | MOL012251 | Chrysin-5-methylether | ADRA1B |
| banzhilian | MOL012251 | Chrysin-5-methylether | ADRB2 |
| banzhilian | MOL012251 | Chrysin-5-methylether | ADRA1D |
| banzhilian | MOL012251 | Chrysin-5-methylether | SLC6A4 |
| banzhilian | MOL012251 | Chrysin-5-methylether | ESR2 |
| banzhilian | MOL012251 | Chrysin-5-methylether | DPP4 |
| banzhilian | MOL012251 | Chrysin-5-methylether | HSP90AB1 |
| banzhilian | MOL012251 | Chrysin-5-methylether | CDK2 |
| banzhilian | MOL012251 | Chrysin-5-methylether | MAOB |
| banzhilian | MOL012251 | Chrysin-5-methylether | CHEK1 |
| banzhilian | MOL012251 | Chrysin-5-methylether | PRSS1 |
| banzhilian | MOL012251 | Chrysin-5-methylether | PKIA |
| banzhilian | MOL012251 | Chrysin-5-methylether | CAMKMT |
| banzhilian | MOL012252 | 9,19-cyclolanost-24-en-3-ol | NR3C2 |
| banzhilian | MOL012254 | campesterol | PGR |
| banzhilian | MOL000953 | CLR | PGR |
| banzhilian | MOL000953 | CLR | NR3C2 |
| banzhilian | MOL000953 | CLR | NCOA2 |
| banzhilian | MOL000358 | beta-sitosterol | PGR |
| banzhilian | MOL000358 | beta-sitosterol | NCOA2 |
| banzhilian | MOL000358 | beta-sitosterol | PTGS1 |
| banzhilian | MOL000358 | beta-sitosterol | PTGS2 |
| banzhilian | MOL000358 | beta-sitosterol | HSP90AB1 |
| banzhilian | MOL000358 | beta-sitosterol | KCNH2 |
| banzhilian | MOL000358 | beta-sitosterol | DRD1 |
| banzhilian | MOL000358 | beta-sitosterol | CHRM3 |
| banzhilian | MOL000358 | beta-sitosterol | CHRM1 |
| banzhilian | MOL000358 | beta-sitosterol | SCN5A |
| banzhilian | MOL000358 | beta-sitosterol | CHRM4 |
| banzhilian | MOL000358 | beta-sitosterol | ADRA1A |
| banzhilian | MOL000358 | beta-sitosterol | CHRM2 |
| banzhilian | MOL000358 | beta-sitosterol | ADRA1B |
| banzhilian | MOL000358 | beta-sitosterol | ADRB2 |
| banzhilian | MOL000358 | beta-sitosterol | CHRNA2 |
| banzhilian | MOL000358 | beta-sitosterol | SLC6A4 |
| banzhilian | MOL000358 | beta-sitosterol | OPRM1 |
| banzhilian | MOL000358 | beta-sitosterol | GABRA1 |
| banzhilian | MOL000358 | beta-sitosterol | BCL2 |
| banzhilian | MOL000358 | beta-sitosterol | BAX |
| banzhilian | MOL000358 | beta-sitosterol | CASP9 |
| banzhilian | MOL000358 | beta-sitosterol | JUN |
| banzhilian | MOL000358 | beta-sitosterol | CASP3 |
| banzhilian | MOL000358 | beta-sitosterol | CASP8 |
| banzhilian | MOL000358 | beta-sitosterol | PRKCA |
| banzhilian | MOL000358 | beta-sitosterol | PON1 |
| banzhilian | MOL000358 | beta-sitosterol | MAP2 |
| banzhilian | MOL012266 | rivularin | NOS2 |
| banzhilian | MOL012266 | rivularin | PTGS1 |
| banzhilian | MOL012266 | rivularin | KCNH2 |
| banzhilian | MOL012266 | rivularin | AR |
| banzhilian | MOL012266 | rivularin | SCN5A |
| banzhilian | MOL012266 | rivularin | PTGS2 |
| banzhilian | MOL012266 | rivularin | CA2 |
| banzhilian | MOL012266 | rivularin | F7 |
| banzhilian | MOL012266 | rivularin | KDR |
| banzhilian | MOL012266 | rivularin | RXRA |
| banzhilian | MOL012266 | rivularin | ESR2 |
| banzhilian | MOL012266 | rivularin | DPP4 |
| banzhilian | MOL012266 | rivularin | HSP90AB1 |
| banzhilian | MOL012266 | rivularin | PRSS1 |
| banzhilian | MOL012266 | rivularin | NCOA2 |
| banzhilian | MOL012266 | rivularin | NCOA1 |
| banzhilian | MOL012266 | rivularin | CAMKMT |
| banzhilian | MOL001973 | Sitosteryl acetate | PGR |
| banzhilian | MOL012269 | Stigmasta-5,22-dien-3-ol-acetate | PGR |
| banzhilian | MOL012270 | Stigmastan-3,5,22-triene | PGR |
| banzhilian | MOL000449 | Stigmasterol | PGR |
| banzhilian | MOL000449 | Stigmasterol | NR3C2 |
| banzhilian | MOL000449 | Stigmasterol | NCOA2 |
| banzhilian | MOL000449 | Stigmasterol | ADH1C |
| banzhilian | MOL000449 | Stigmasterol | IGHG1 |
| banzhilian | MOL000449 | Stigmasterol | RXRA |
| banzhilian | MOL000449 | Stigmasterol | NCOA1 |
| banzhilian | MOL000449 | Stigmasterol | PTGS1 |
| banzhilian | MOL000449 | Stigmasterol | PTGS2 |
| banzhilian | MOL000449 | Stigmasterol | ADRA2A |
| banzhilian | MOL000449 | Stigmasterol | SLC6A2 |
| banzhilian | MOL000449 | Stigmasterol | SLC6A3 |
| banzhilian | MOL000449 | Stigmasterol | ADRB2 |
| banzhilian | MOL000449 | Stigmasterol | AKR1B1 |
| banzhilian | MOL000449 | Stigmasterol | PLAU |
| banzhilian | MOL000449 | Stigmasterol | LTA4H |
| banzhilian | MOL000449 | Stigmasterol | MAOB |
| banzhilian | MOL000449 | Stigmasterol | MAOA |
| banzhilian | MOL000449 | Stigmasterol | CTRB1 |
| banzhilian | MOL000449 | Stigmasterol | CHRM3 |
| banzhilian | MOL000449 | Stigmasterol | CHRM1 |
| banzhilian | MOL000449 | Stigmasterol | ADRB1 |
| banzhilian | MOL000449 | Stigmasterol | SCN5A |
| banzhilian | MOL000449 | Stigmasterol | ADRA1A |
| banzhilian | MOL000449 | Stigmasterol | CHRM2 |
| banzhilian | MOL000449 | Stigmasterol | ADRA1B |
| banzhilian | MOL000449 | Stigmasterol | GABRA1 |
| banzhilian | MOL000173 | wogonin | NOS2 |
| banzhilian | MOL000173 | wogonin | PTGS1 |
| banzhilian | MOL000173 | wogonin | ESR1 |
| banzhilian | MOL000173 | wogonin | AR |
| banzhilian | MOL000173 | wogonin | SCN5A |
| banzhilian | MOL000173 | wogonin | PPARG |
| banzhilian | MOL000173 | wogonin | PTGS2 |
| banzhilian | MOL000173 | wogonin | RXRA |
| banzhilian | MOL000173 | wogonin | DPP4 |
| banzhilian | MOL000173 | wogonin | MAPK14 |
| banzhilian | MOL000173 | wogonin | GSK3B |
| banzhilian | MOL000173 | wogonin | HSP90AB1 |
| banzhilian | MOL000173 | wogonin | CDK2 |
| banzhilian | MOL000173 | wogonin | CHEK1 |
| banzhilian | MOL000173 | wogonin | PRSS1 |
| banzhilian | MOL000173 | wogonin | CAMKMT |
| banzhilian | MOL000173 | wogonin | ADRB2 |
| banzhilian | MOL000173 | wogonin | GABRA1 |
| banzhilian | MOL000173 | wogonin | RELA |
| banzhilian | MOL000173 | wogonin | AKT1 |
| banzhilian | MOL000173 | wogonin | CCND1 |
| banzhilian | MOL000173 | wogonin | BCL2 |
| banzhilian | MOL000173 | wogonin | CDKN1A |
| banzhilian | MOL000173 | wogonin | EIF6 |
| banzhilian | MOL000173 | wogonin | BAX |
| banzhilian | MOL000173 | wogonin | CASP9 |
| banzhilian | MOL000173 | wogonin | KDR |
| banzhilian | MOL000173 | wogonin | TNFAIP6 |
| banzhilian | MOL000173 | wogonin | JUN |
| banzhilian | MOL000173 | wogonin | IL6R |
| banzhilian | MOL000173 | wogonin | AHSA1 |
| banzhilian | MOL000173 | wogonin | CASP3 |
| banzhilian | MOL000173 | wogonin | TP53 |
| banzhilian | MOL000173 | wogonin | TEP1 |
| banzhilian | MOL000173 | wogonin | MMP1 |
| banzhilian | MOL000173 | wogonin | CCL2 |
| banzhilian | MOL000173 | wogonin | PRKCD |
| banzhilian | MOL000173 | wogonin | PTGER3 |
| banzhilian | MOL000173 | wogonin | FN1 |
| banzhilian | MOL000173 | wogonin | CXCL8 |
| banzhilian | MOL000173 | wogonin | MCL1 |
| banzhilian | MOL001735 | Dinatin | NOS2 |
| banzhilian | MOL001735 | Dinatin | PTGS1 |
| banzhilian | MOL001735 | Dinatin | PTGS2 |
| banzhilian | MOL001735 | Dinatin | DPP4 |
| banzhilian | MOL001735 | Dinatin | HSP90AB1 |
| banzhilian | MOL001735 | Dinatin | PRSS1 |
| banzhilian | MOL001735 | Dinatin | NCOA2 |
| banzhilian | MOL001735 | Dinatin | NCOA1 |
| banzhilian | MOL001735 | Dinatin | CAMKMT |
| banzhilian | MOL001735 | Dinatin | ACHE |
| banzhilian | MOL001735 | Dinatin | RHO |
| banzhilian | MOL001735 | Dinatin | IGHG1 |
| banzhilian | MOL001755 | 24-Ethylcholest-4-en-3-one | PGR |
| banzhilian | MOL001755 | 24-Ethylcholest-4-en-3-one | NR3C2 |
| banzhilian | MOL002714 | baicalein | PTGS1 |
| banzhilian | MOL002714 | baicalein | AR |
| banzhilian | MOL002714 | baicalein | PTGS2 |
| banzhilian | MOL002714 | baicalein | HSP90AB1 |
| banzhilian | MOL002714 | baicalein | DPP4 |
| banzhilian | MOL002714 | baicalein | PRSS1 |
| banzhilian | MOL002714 | baicalein | NCOA2 |
| banzhilian | MOL002714 | baicalein | NCOA1 |
| banzhilian | MOL002714 | baicalein | CAMKMT |
| banzhilian | MOL002714 | baicalein | RELA |
| banzhilian | MOL002714 | baicalein | AKT1 |
| banzhilian | MOL002714 | baicalein | VEGFA |
| banzhilian | MOL002714 | baicalein | BCL2 |
| banzhilian | MOL002714 | baicalein | FOS |
| banzhilian | MOL002714 | baicalein | BAX |
| banzhilian | MOL002714 | baicalein | MMP9 |
| banzhilian | MOL002714 | baicalein | CASP3 |
| banzhilian | MOL002714 | baicalein | TP53 |
| banzhilian | MOL002714 | baicalein | HIF1A |
| banzhilian | MOL002714 | baicalein | FOSL1 |
| banzhilian | MOL002714 | baicalein | FOSL2 |
| banzhilian | MOL002714 | baicalein | CDK1 |
| banzhilian | MOL002714 | baicalein | CCNB1 |
| banzhilian | MOL002714 | baicalein | MPO |
| banzhilian | MOL002714 | baicalein | AHR |
| banzhilian | MOL002714 | baicalein | IGF2 |
| banzhilian | MOL002714 | baicalein | CYCS |
| banzhilian | MOL002714 | baicalein | NFATC1 |
| banzhilian | MOL002714 | baicalein | TDRD7 |
| banzhilian | MOL002714 | baicalein | EGLN1 |
| banzhilian | MOL002714 | baicalein | NOX5 |
| banzhilian | MOL002714 | baicalein | FABP5 |
| banzhilian | MOL002714 | baicalein | APOD |
| banzhilian | MOL002915 | Salvigenin | NOS2 |
| banzhilian | MOL002915 | Salvigenin | PTGS1 |
| banzhilian | MOL002915 | Salvigenin | SCN5A |
| banzhilian | MOL002915 | Salvigenin | PTGS2 |
| banzhilian | MOL002915 | Salvigenin | RXRA |
| banzhilian | MOL002915 | Salvigenin | ACHE |
| banzhilian | MOL002915 | Salvigenin | ADRA1B |
| banzhilian | MOL002915 | Salvigenin | ADRB2 |
| banzhilian | MOL002915 | Salvigenin | DPP4 |
| banzhilian | MOL002915 | Salvigenin | HSP90AB1 |
| banzhilian | MOL002915 | Salvigenin | IGHG1 |
| banzhilian | MOL002915 | Salvigenin | PRSS1 |
| banzhilian | MOL002915 | Salvigenin | NCOA2 |
| banzhilian | MOL002915 | Salvigenin | CAMKMT |
| banzhilian | MOL002915 | Salvigenin | F7 |
| banzhilian | MOL000351 | Rhamnazin | NOS2 |
| banzhilian | MOL000351 | Rhamnazin | PTGS1 |
| banzhilian | MOL000351 | Rhamnazin | ESR1 |
| banzhilian | MOL000351 | Rhamnazin | AR |
| banzhilian | MOL000351 | Rhamnazin | SCN5A |
| banzhilian | MOL000351 | Rhamnazin | PPARG |
| banzhilian | MOL000351 | Rhamnazin | PTGS2 |
| banzhilian | MOL000351 | Rhamnazin | ESR2 |
| banzhilian | MOL000351 | Rhamnazin | DPP4 |
| banzhilian | MOL000351 | Rhamnazin | MAPK14 |
| banzhilian | MOL000351 | Rhamnazin | GSK3B |
| banzhilian | MOL000351 | Rhamnazin | HSP90AB1 |
| banzhilian | MOL000351 | Rhamnazin | CDK2 |
| banzhilian | MOL000351 | Rhamnazin | PRSS1 |
| banzhilian | MOL000351 | Rhamnazin | CCNA2 |
| banzhilian | MOL000351 | Rhamnazin | NCOA2 |
| banzhilian | MOL000351 | Rhamnazin | CAMKMT |
| banzhilian | MOL000351 | Rhamnazin | PPARD |
| banzhilian | MOL000351 | Rhamnazin | CHEK1 |
| banzhilian | MOL000359 | sitosterol | PGR |
| banzhilian | MOL000359 | sitosterol | NCOA2 |
| banzhilian | MOL000359 | sitosterol | NR3C2 |
| banzhilian | MOL005190 | eriodictyol | PTGS1 |
| banzhilian | MOL005190 | eriodictyol | PTGS2 |
| banzhilian | MOL005190 | eriodictyol | HSP90AB1 |
| banzhilian | MOL005190 | eriodictyol | NCOA2 |
| banzhilian | MOL005190 | eriodictyol | HMOX1 |
| banzhilian | MOL005190 | eriodictyol | NFE2L2 |
| banzhilian | MOL005190 | eriodictyol | NQO1 |
| banzhilian | MOL005869 | daucostero_qt | PGR |
| banzhilian | MOL005869 | daucostero_qt | NCOA2 |
| banzhilian | MOL000006 | luteolin | PTGS1 |
| banzhilian | MOL000006 | luteolin | AR |
| banzhilian | MOL000006 | luteolin | PTGS2 |
| banzhilian | MOL000006 | luteolin | HSP90AB1 |
| banzhilian | MOL000006 | luteolin | PRSS1 |
| banzhilian | MOL000006 | luteolin | NCOA2 |
| banzhilian | MOL000006 | luteolin | DPP4 |
| banzhilian | MOL000006 | luteolin | RELA |
| banzhilian | MOL000006 | luteolin | EGFR |
| banzhilian | MOL000006 | luteolin | AKT1 |
| banzhilian | MOL000006 | luteolin | VEGFA |
| banzhilian | MOL000006 | luteolin | CCND1 |
| banzhilian | MOL000006 | luteolin | BCL2L1 |
| banzhilian | MOL000006 | luteolin | CDKN1A |
| banzhilian | MOL000006 | luteolin | CASP9 |
| banzhilian | MOL000006 | luteolin | MMP2 |
| banzhilian | MOL000006 | luteolin | MMP9 |
| banzhilian | MOL000006 | luteolin | MAPK1 |
| banzhilian | MOL000006 | luteolin | IL10RA |
| banzhilian | MOL000006 | luteolin | RB1 |
| banzhilian | MOL000006 | luteolin | CDK4 |
| banzhilian | MOL000006 | luteolin | TNFAIP6 |
| banzhilian | MOL000006 | luteolin | JUN |
| banzhilian | MOL000006 | luteolin | IL6R |
| banzhilian | MOL000006 | luteolin | CASP3 |
| banzhilian | MOL000006 | luteolin | TP53 |
| banzhilian | MOL000006 | luteolin | NFKBIA |
| banzhilian | MOL000006 | luteolin | TOP1 |
| banzhilian | MOL000006 | luteolin | MDM2 |
| banzhilian | MOL000006 | luteolin | APP |
| banzhilian | MOL000006 | luteolin | MMP1 |
| banzhilian | MOL000006 | luteolin | PCNA |
| banzhilian | MOL000006 | luteolin | ERBB2 |
| banzhilian | MOL000006 | luteolin | PPARG |
| banzhilian | MOL000006 | luteolin | HMOX1 |
| banzhilian | MOL000006 | luteolin | CASP7 |
| banzhilian | MOL000006 | luteolin | ICAM1 |
| banzhilian | MOL000006 | luteolin | MCL1 |
| banzhilian | MOL000006 | luteolin | BIRC5 |
| banzhilian | MOL000006 | luteolin | IL2RA |
| banzhilian | MOL000006 | luteolin | CCNB1 |
| banzhilian | MOL000006 | luteolin | TYR |
| banzhilian | MOL000006 | luteolin | IFNG |
| banzhilian | MOL000006 | luteolin | IL4 |
| banzhilian | MOL000006 | luteolin | TOP2A |
| banzhilian | MOL000006 | luteolin | GSTP1 |
| banzhilian | MOL000006 | luteolin | XIAP |
| banzhilian | MOL000006 | luteolin | SLC2A4 |
| banzhilian | MOL000006 | luteolin | INSRR |
| banzhilian | MOL000006 | luteolin | CD40LG |
| banzhilian | MOL000006 | luteolin | PTGES |
| banzhilian | MOL000006 | luteolin | NUF2 |
| banzhilian | MOL000006 | luteolin | ADCY2 |
| banzhilian | MOL000006 | luteolin | MET |
| banzhilian | MOL008206 | Moslosooflavone | NOS2 |
| banzhilian | MOL008206 | Moslosooflavone | PTGS1 |
| banzhilian | MOL008206 | Moslosooflavone | AR |
| banzhilian | MOL008206 | Moslosooflavone | SCN5A |
| banzhilian | MOL008206 | Moslosooflavone | PPARG |
| banzhilian | MOL008206 | Moslosooflavone | PTGS2 |
| banzhilian | MOL008206 | Moslosooflavone | RXRA |
| banzhilian | MOL008206 | Moslosooflavone | ESR2 |
| banzhilian | MOL008206 | Moslosooflavone | GABRA1 |
| banzhilian | MOL008206 | Moslosooflavone | DPP4 |
| banzhilian | MOL008206 | Moslosooflavone | MAPK14 |
| banzhilian | MOL008206 | Moslosooflavone | GSK3B |
| banzhilian | MOL008206 | Moslosooflavone | HSP90AB1 |
| banzhilian | MOL008206 | Moslosooflavone | CDK2 |
| banzhilian | MOL008206 | Moslosooflavone | CHEK1 |
| banzhilian | MOL008206 | Moslosooflavone | PRSS1 |
| banzhilian | MOL008206 | Moslosooflavone | NCOA1 |
| banzhilian | MOL008206 | Moslosooflavone | CAMKMT |
| banzhilian | MOL008206 | Moslosooflavone | ADRA1B |
| banzhilian | MOL008206 | Moslosooflavone | ADRB2 |
| banzhilian | MOL000098 | quercetin | PTGS1 |
| banzhilian | MOL000098 | quercetin | AR |
| banzhilian | MOL000098 | quercetin | PPARG |
| banzhilian | MOL000098 | quercetin | PTGS2 |
| banzhilian | MOL000098 | quercetin | HSP90AB1 |
| banzhilian | MOL000098 | quercetin | NCOA2 |
| banzhilian | MOL000098 | quercetin | DPP4 |
| banzhilian | MOL000098 | quercetin | AKR1B1 |
| banzhilian | MOL000098 | quercetin | PRSS1 |
| banzhilian | MOL000098 | quercetin | KCNH2 |
| banzhilian | MOL000098 | quercetin | SCN5A |
| banzhilian | MOL000098 | quercetin | ADRB2 |
| banzhilian | MOL000098 | quercetin | MMP3 |
| banzhilian | MOL000098 | quercetin | F7 |
| banzhilian | MOL000098 | quercetin | RXRA |
| banzhilian | MOL000098 | quercetin | ACHE |
| banzhilian | MOL000098 | quercetin | GABRA1 |
| banzhilian | MOL000098 | quercetin | MAOB |
| banzhilian | MOL000098 | quercetin | RELA |
| banzhilian | MOL000098 | quercetin | EGFR |
| banzhilian | MOL000098 | quercetin | AKT1 |
| banzhilian | MOL000098 | quercetin | VEGFA |
| banzhilian | MOL000098 | quercetin | CCND1 |
| banzhilian | MOL000098 | quercetin | BCL2 |
| banzhilian | MOL000098 | quercetin | BCL2L1 |
| banzhilian | MOL000098 | quercetin | FOS |
| banzhilian | MOL000098 | quercetin | CDKN1A |
| banzhilian | MOL000098 | quercetin | EIF6 |
| banzhilian | MOL000098 | quercetin | BAX |
| banzhilian | MOL000098 | quercetin | CASP9 |
| banzhilian | MOL000098 | quercetin | PLAU |
| banzhilian | MOL000098 | quercetin | MMP2 |
| banzhilian | MOL000098 | quercetin | MMP9 |
| banzhilian | MOL000098 | quercetin | MAPK1 |
| banzhilian | MOL000098 | quercetin | IL10RA |
| banzhilian | MOL000098 | quercetin | EGF |
| banzhilian | MOL000098 | quercetin | RB1 |
| banzhilian | MOL000098 | quercetin | TNFAIP6 |
| banzhilian | MOL000098 | quercetin | JUN |
| banzhilian | MOL000098 | quercetin | IL6R |
| banzhilian | MOL000098 | quercetin | AHSA1 |
| banzhilian | MOL000098 | quercetin | CASP3 |
| banzhilian | MOL000098 | quercetin | TP53 |
| banzhilian | MOL000098 | quercetin | ELK1 |
| banzhilian | MOL000098 | quercetin | NFKBIA |
| banzhilian | MOL000098 | quercetin | POR |
| banzhilian | MOL000098 | quercetin | ODC1 |
| banzhilian | MOL000098 | quercetin | CASP8 |
| banzhilian | MOL000098 | quercetin | TOP1 |
| banzhilian | MOL000098 | quercetin | RAF1 |
| banzhilian | MOL000098 | quercetin | SOD1 |
| banzhilian | MOL000098 | quercetin | PRKCA |
| banzhilian | MOL000098 | quercetin | MMP1 |
| banzhilian | MOL000098 | quercetin | HIF1A |
| banzhilian | MOL000098 | quercetin | STAT1 |
| banzhilian | MOL000098 | quercetin | RUNX1T1 |
| banzhilian | MOL000098 | quercetin | CDK1 |
| banzhilian | MOL000098 | quercetin | HSPA5 |
| banzhilian | MOL000098 | quercetin | ERBB2 |
| banzhilian | MOL000098 | quercetin | PPARG |
| banzhilian | MOL000098 | quercetin | ACACA |
| banzhilian | MOL000098 | quercetin | HMOX1 |
| banzhilian | MOL000098 | quercetin | CYP3A4 |
| banzhilian | MOL000098 | quercetin | CYP1A2 |
| banzhilian | MOL000098 | quercetin | CAV1 |
| banzhilian | MOL000098 | quercetin | MYC |
| banzhilian | MOL000098 | quercetin | F3 |
| banzhilian | MOL000098 | quercetin | GJA1 |
| banzhilian | MOL000098 | quercetin | CYP1A1 |
| banzhilian | MOL000098 | quercetin | ICAM1 |
| banzhilian | MOL000098 | quercetin | IL1B |
| banzhilian | MOL000098 | quercetin | CCL2 |
| banzhilian | MOL000098 | quercetin | SELE |
| banzhilian | MOL000098 | quercetin | VCAM1 |
| banzhilian | MOL000098 | quercetin | PTGER3 |
| banzhilian | MOL000098 | quercetin | CXCL8 |
| banzhilian | MOL000098 | quercetin | PRKCB |
| banzhilian | MOL000098 | quercetin | BIRC5 |
| banzhilian | MOL000098 | quercetin | DUOX2 |
| banzhilian | MOL000098 | quercetin | NOS3 |
| banzhilian | MOL000098 | quercetin | HSPB1 |
| banzhilian | MOL000098 | quercetin | SULT1E1 |
| banzhilian | MOL000098 | quercetin | IL2RA |
| banzhilian | MOL000098 | quercetin | NR1I2 |
| banzhilian | MOL000098 | quercetin | CYP1B1 |
| banzhilian | MOL000098 | quercetin | CCNB1 |
| banzhilian | MOL000098 | quercetin | PLAT |
| banzhilian | MOL000098 | quercetin | THBD |
| banzhilian | MOL000098 | quercetin | SERPINE1 |
| banzhilian | MOL000098 | quercetin | COL1A1 |
| banzhilian | MOL000098 | quercetin | IFNG |
| banzhilian | MOL000098 | quercetin | ALOX5 |
| banzhilian | MOL000098 | quercetin | IL1A |
| banzhilian | MOL000098 | quercetin | MPO |
| banzhilian | MOL000098 | quercetin | TOP2A |
| banzhilian | MOL000098 | quercetin | NCF1 |
| banzhilian | MOL000098 | quercetin | ABCG2 |
| banzhilian | MOL000098 | quercetin | HAS2 |
| banzhilian | MOL000098 | quercetin | GSTP1 |
| banzhilian | MOL000098 | quercetin | NFE2L2 |
| banzhilian | MOL000098 | quercetin | NQO1 |
| banzhilian | MOL000098 | quercetin | PARP1 |
| banzhilian | MOL000098 | quercetin | AHR |
| banzhilian | MOL000098 | quercetin | PSMD3 |
| banzhilian | MOL000098 | quercetin | SLC2A4 |
| banzhilian | MOL000098 | quercetin | COL3A1 |
| banzhilian | MOL000098 | quercetin | CXCL11 |
| banzhilian | MOL000098 | quercetin | CXCL2 |
| banzhilian | MOL000098 | quercetin | DCAF5 |
| banzhilian | MOL000098 | quercetin | NR1I3 |
| banzhilian | MOL000098 | quercetin | CHEK2 |
| banzhilian | MOL000098 | quercetin | INSRR |
| banzhilian | MOL000098 | quercetin | CLDN4 |
| banzhilian | MOL000098 | quercetin | PPARA |
| banzhilian | MOL000098 | quercetin | PPARD |
| banzhilian | MOL000098 | quercetin | HSF1 |
| banzhilian | MOL000098 | quercetin | CXCL10 |
| banzhilian | MOL000098 | quercetin | CHUK |
| banzhilian | MOL000098 | quercetin | SPP1 |
| banzhilian | MOL000098 | quercetin | RUNX2 |
| banzhilian | MOL000098 | quercetin | RASSF1 |
| banzhilian | MOL000098 | quercetin | E2F1 |
| banzhilian | MOL000098 | quercetin | E2F2 |
| banzhilian | MOL000098 | quercetin | ACP3 |
| banzhilian | MOL000098 | quercetin | CTSD |
| banzhilian | MOL000098 | quercetin | IGFBP3 |
| banzhilian | MOL000098 | quercetin | IGF2 |
| banzhilian | MOL000098 | quercetin | CD40LG |
| banzhilian | MOL000098 | quercetin | IRF1 |
| banzhilian | MOL000098 | quercetin | ERBB3 |
| banzhilian | MOL000098 | quercetin | PON1 |
| banzhilian | MOL000098 | quercetin | DIO1 |
| banzhilian | MOL000098 | quercetin | PCOLCE |
| banzhilian | MOL000098 | quercetin | NPEPPS |
| banzhilian | MOL000098 | quercetin | HK2 |
| banzhilian | MOL000098 | quercetin | RASA1 |
| banzhilian | MOL000098 | quercetin | GSTM1 |
| banzhilian | MOL000098 | quercetin | GSTM2 |
| ezhu | MOL000296 | hederagenin | PGR |
| ezhu | MOL000296 | hederagenin | NCOA2 |
| ezhu | MOL000296 | hederagenin | CHRM3 |
| ezhu | MOL000296 | hederagenin | CHRM1 |
| ezhu | MOL000296 | hederagenin | CHRM2 |
| ezhu | MOL000296 | hederagenin | ADRA1B |
| ezhu | MOL000296 | hederagenin | GABRA1 |
| ezhu | MOL000296 | hederagenin | GRIA2 |
| ezhu | MOL000296 | hederagenin | IGHG1 |
| ezhu | MOL000296 | hederagenin | ADH1B |
| ezhu | MOL000296 | hederagenin | ADH1C |
| ezhu | MOL000296 | hederagenin | LYG1 |
| ezhu | MOL000296 | hederagenin | PTGS1 |
| ezhu | MOL000296 | hederagenin | SCN5A |
| ezhu | MOL000296 | hederagenin | PTGS2 |
| ezhu | MOL000296 | hederagenin | RXRA |
| ezhu | MOL000296 | hederagenin | SLC6A2 |
| huangqi | MOL000211 | Mairin | PGR |
| huangqi | MOL000239 | Jaranol | NOS2 |
| huangqi | MOL000239 | Jaranol | PTGS1 |
| huangqi | MOL000239 | Jaranol | AR |
| huangqi | MOL000239 | Jaranol | SCN5A |
| huangqi | MOL000239 | Jaranol | PTGS2 |
| huangqi | MOL000239 | Jaranol | ESR2 |
| huangqi | MOL000239 | Jaranol | DPP4 |
| huangqi | MOL000239 | Jaranol | HSP90AB1 |
| huangqi | MOL000239 | Jaranol | CDK2 |
| huangqi | MOL000239 | Jaranol | CHEK1 |
| huangqi | MOL000239 | Jaranol | PRSS1 |
| huangqi | MOL000239 | Jaranol | NCOA2 |
| huangqi | MOL000239 | Jaranol | CAMKMT |
| huangqi | MOL000296 | hederagenin | PGR |
| huangqi | MOL000296 | hederagenin | NCOA2 |
| huangqi | MOL000296 | hederagenin | CHRM3 |
| huangqi | MOL000296 | hederagenin | CHRM1 |
| huangqi | MOL000296 | hederagenin | CHRM2 |
| huangqi | MOL000296 | hederagenin | ADRA1B |
| huangqi | MOL000296 | hederagenin | GABRA1 |
| huangqi | MOL000296 | hederagenin | GRIA2 |
| huangqi | MOL000296 | hederagenin | IGHG1 |
| huangqi | MOL000296 | hederagenin | ADH1B |
| huangqi | MOL000296 | hederagenin | ADH1C |
| huangqi | MOL000296 | hederagenin | LYG1 |
| huangqi | MOL000296 | hederagenin | PTGS1 |
| huangqi | MOL000296 | hederagenin | SCN5A |
| huangqi | MOL000296 | hederagenin | PTGS2 |
| huangqi | MOL000296 | hederagenin | RXRA |
| huangqi | MOL000296 | hederagenin | SLC6A2 |
| huangqi | MOL000033 | (3S,8S,9S,10R,13R,14S,17R)-10,13-dimethyl-17-[(2R,5S)-5-propan-2-yloctan-2-yl]-2,3,4,7,8,9,11,12,14,15,16,17-dodecahydro-1H-cyclopenta[a]phenanthren-3-ol | PGR |
| huangqi | MOL000354 | isorhamnetin | NOS2 |
| huangqi | MOL000354 | isorhamnetin | PTGS1 |
| huangqi | MOL000354 | isorhamnetin | ESR1 |
| huangqi | MOL000354 | isorhamnetin | AR |
| huangqi | MOL000354 | isorhamnetin | PPARG |
| huangqi | MOL000354 | isorhamnetin | PTGS2 |
| huangqi | MOL000354 | isorhamnetin | ESR2 |
| huangqi | MOL000354 | isorhamnetin | DPP4 |
| huangqi | MOL000354 | isorhamnetin | MAPK14 |
| huangqi | MOL000354 | isorhamnetin | GSK3B |
| huangqi | MOL000354 | isorhamnetin | HSP90AB1 |
| huangqi | MOL000354 | isorhamnetin | CDK2 |
| huangqi | MOL000354 | isorhamnetin | PRSS1 |
| huangqi | MOL000354 | isorhamnetin | CCNA2 |
| huangqi | MOL000354 | isorhamnetin | NCOA2 |
| huangqi | MOL000354 | isorhamnetin | CAMKMT |
| huangqi | MOL000354 | isorhamnetin | PYGM |
| huangqi | MOL000354 | isorhamnetin | PPARD |
| huangqi | MOL000354 | isorhamnetin | CHEK1 |
| huangqi | MOL000354 | isorhamnetin | AKR1B1 |
| huangqi | MOL000354 | isorhamnetin | NCOA1 |
| huangqi | MOL000354 | isorhamnetin | F7 |
| huangqi | MOL000354 | isorhamnetin | ACHE |
| huangqi | MOL000354 | isorhamnetin | GABRA1 |
| huangqi | MOL000354 | isorhamnetin | MAOB |
| huangqi | MOL000354 | isorhamnetin | GRIA2 |
| huangqi | MOL000354 | isorhamnetin | RELA |
| huangqi | MOL000354 | isorhamnetin | NCF1 |
| huangqi | MOL000354 | isorhamnetin | OLR1 |
| huangqi | MOL000371 | 3,9-di-O-methylnissolin | NOS2 |
| huangqi | MOL000371 | 3,9-di-O-methylnissolin | PTGS1 |
| huangqi | MOL000371 | 3,9-di-O-methylnissolin | CHRM3 |
| huangqi | MOL000371 | 3,9-di-O-methylnissolin | CHRM1 |
| huangqi | MOL000371 | 3,9-di-O-methylnissolin | ESR1 |
| huangqi | MOL000371 | 3,9-di-O-methylnissolin | ADRB1 |
| huangqi | MOL000371 | 3,9-di-O-methylnissolin | SCN5A |
| huangqi | MOL000371 | 3,9-di-O-methylnissolin | PTGS2 |
| huangqi | MOL000371 | 3,9-di-O-methylnissolin | HTR3A |
| huangqi | MOL000371 | 3,9-di-O-methylnissolin | ADRA2C |
| huangqi | MOL000371 | 3,9-di-O-methylnissolin | RXRA |
| huangqi | MOL000371 | 3,9-di-O-methylnissolin | ACHE |
| huangqi | MOL000371 | 3,9-di-O-methylnissolin | ADRA1B |
| huangqi | MOL000371 | 3,9-di-O-methylnissolin | ADRB2 |
| huangqi | MOL000371 | 3,9-di-O-methylnissolin | ADRA1D |
| huangqi | MOL000371 | 3,9-di-O-methylnissolin | OPRM1 |
| huangqi | MOL000371 | 3,9-di-O-methylnissolin | GABRA1 |
| huangqi | MOL000371 | 3,9-di-O-methylnissolin | PRSS1 |
| huangqi | MOL000371 | 3,9-di-O-methylnissolin | NCOA2 |
| huangqi | MOL000371 | 3,9-di-O-methylnissolin | CAMKMT |
| huangqi | MOL000378 | 7-O-methylisomucronulatol | NOS2 |
| huangqi | MOL000378 | 7-O-methylisomucronulatol | PTGS1 |
| huangqi | MOL000378 | 7-O-methylisomucronulatol | DRD1 |
| huangqi | MOL000378 | 7-O-methylisomucronulatol | CHRM3 |
| huangqi | MOL000378 | 7-O-methylisomucronulatol | KCNH2 |
| huangqi | MOL000378 | 7-O-methylisomucronulatol | CHRM1 |
| huangqi | MOL000378 | 7-O-methylisomucronulatol | ESR1 |
| huangqi | MOL000378 | 7-O-methylisomucronulatol | AR |
| huangqi | MOL000378 | 7-O-methylisomucronulatol | ADRB1 |
| huangqi | MOL000378 | 7-O-methylisomucronulatol | SCN5A |
| huangqi | MOL000378 | 7-O-methylisomucronulatol | PPARG |
| huangqi | MOL000378 | 7-O-methylisomucronulatol | CHRM5 |
| huangqi | MOL000378 | 7-O-methylisomucronulatol | PTGS2 |
| huangqi | MOL000378 | 7-O-methylisomucronulatol | ADRA2C |
| huangqi | MOL000378 | 7-O-methylisomucronulatol | CHRM4 |
| huangqi | MOL000378 | 7-O-methylisomucronulatol | RXRA |
| huangqi | MOL000378 | 7-O-methylisomucronulatol | OPRD1 |
| huangqi | MOL000378 | 7-O-methylisomucronulatol | ADRA1A |
| huangqi | MOL000378 | 7-O-methylisomucronulatol | CHRM2 |
| huangqi | MOL000378 | 7-O-methylisomucronulatol | ADRA1B |
| huangqi | MOL000378 | 7-O-methylisomucronulatol | SLC6A3 |
| huangqi | MOL000378 | 7-O-methylisomucronulatol | ADRB2 |
| huangqi | MOL000378 | 7-O-methylisomucronulatol | ADRA1D |
| huangqi | MOL000378 | 7-O-methylisomucronulatol | SLC6A4 |
| huangqi | MOL000378 | 7-O-methylisomucronulatol | ESR2 |
| huangqi | MOL000378 | 7-O-methylisomucronulatol | GABRA1 |
| huangqi | MOL000378 | 7-O-methylisomucronulatol | DPP4 |
| huangqi | MOL000378 | 7-O-methylisomucronulatol | MAPK14 |
| huangqi | MOL000378 | 7-O-methylisomucronulatol | GSK3B |
| huangqi | MOL000378 | 7-O-methylisomucronulatol | HSP90AB1 |
| huangqi | MOL000378 | 7-O-methylisomucronulatol | CDK2 |
| huangqi | MOL000378 | 7-O-methylisomucronulatol | CHEK1 |
| huangqi | MOL000378 | 7-O-methylisomucronulatol | RXRB |
| huangqi | MOL000378 | 7-O-methylisomucronulatol | PRSS1 |
| huangqi | MOL000378 | 7-O-methylisomucronulatol | CCNA2 |
| huangqi | MOL000378 | 7-O-methylisomucronulatol | NCOA2 |
| huangqi | MOL000378 | 7-O-methylisomucronulatol | CAMKMT |
| huangqi | MOL000379 | 9,10-dimethoxypterocarpan-3-O-尾-D-glucoside | PTGS2 |
| huangqi | MOL000379 | 9,10-dimethoxypterocarpan-3-O-尾-D-glucoside | NCOA2 |
| huangqi | MOL000380 | (6aR,11aR)-9,10-dimethoxy-6a,11a-dihydro-6H-benzofurano[3,2-c]chromen-3-ol | NOS2 |
| huangqi | MOL000380 | (6aR,11aR)-9,10-dimethoxy-6a,11a-dihydro-6H-benzofurano[3,2-c]chromen-3-ol | PTGS1 |
| huangqi | MOL000380 | (6aR,11aR)-9,10-dimethoxy-6a,11a-dihydro-6H-benzofurano[3,2-c]chromen-3-ol | CHRM3 |
| huangqi | MOL000380 | (6aR,11aR)-9,10-dimethoxy-6a,11a-dihydro-6H-benzofurano[3,2-c]chromen-3-ol | CHRM1 |
| huangqi | MOL000380 | (6aR,11aR)-9,10-dimethoxy-6a,11a-dihydro-6H-benzofurano[3,2-c]chromen-3-ol | ESR1 |
| huangqi | MOL000380 | (6aR,11aR)-9,10-dimethoxy-6a,11a-dihydro-6H-benzofurano[3,2-c]chromen-3-ol | SCN5A |
| huangqi | MOL000380 | (6aR,11aR)-9,10-dimethoxy-6a,11a-dihydro-6H-benzofurano[3,2-c]chromen-3-ol | PTGS2 |
| huangqi | MOL000380 | (6aR,11aR)-9,10-dimethoxy-6a,11a-dihydro-6H-benzofurano[3,2-c]chromen-3-ol | HTR3A |
| huangqi | MOL000380 | (6aR,11aR)-9,10-dimethoxy-6a,11a-dihydro-6H-benzofurano[3,2-c]chromen-3-ol | RXRA |
| huangqi | MOL000380 | (6aR,11aR)-9,10-dimethoxy-6a,11a-dihydro-6H-benzofurano[3,2-c]chromen-3-ol | ACHE |
| huangqi | MOL000380 | (6aR,11aR)-9,10-dimethoxy-6a,11a-dihydro-6H-benzofurano[3,2-c]chromen-3-ol | ADRA1B |
| huangqi | MOL000380 | (6aR,11aR)-9,10-dimethoxy-6a,11a-dihydro-6H-benzofurano[3,2-c]chromen-3-ol | ADRB2 |
| huangqi | MOL000380 | (6aR,11aR)-9,10-dimethoxy-6a,11a-dihydro-6H-benzofurano[3,2-c]chromen-3-ol | ADRA1D |
| huangqi | MOL000380 | (6aR,11aR)-9,10-dimethoxy-6a,11a-dihydro-6H-benzofurano[3,2-c]chromen-3-ol | GABRA1 |
| huangqi | MOL000380 | (6aR,11aR)-9,10-dimethoxy-6a,11a-dihydro-6H-benzofurano[3,2-c]chromen-3-ol | HSP90AB1 |
| huangqi | MOL000380 | (6aR,11aR)-9,10-dimethoxy-6a,11a-dihydro-6H-benzofurano[3,2-c]chromen-3-ol | PRSS1 |
| huangqi | MOL000380 | (6aR,11aR)-9,10-dimethoxy-6a,11a-dihydro-6H-benzofurano[3,2-c]chromen-3-ol | NCOA2 |
| huangqi | MOL000380 | (6aR,11aR)-9,10-dimethoxy-6a,11a-dihydro-6H-benzofurano[3,2-c]chromen-3-ol | NCOA1 |
| huangqi | MOL000380 | (6aR,11aR)-9,10-dimethoxy-6a,11a-dihydro-6H-benzofurano[3,2-c]chromen-3-ol | CAMKMT |
| huangqi | MOL000380 | (6aR,11aR)-9,10-dimethoxy-6a,11a-dihydro-6H-benzofurano[3,2-c]chromen-3-ol | CHRM4 |
| huangqi | MOL000387 | Bifendate | PTGS2 |
| huangqi | MOL000387 | Bifendate | KDR |
| huangqi | MOL000387 | Bifendate | MET |
| huangqi | MOL000387 | Bifendate | HSP90AB1 |
| huangqi | MOL000387 | Bifendate | PTGS1 |
| huangqi | MOL000392 | formononetin | NOS2 |
| huangqi | MOL000392 | formononetin | PTGS1 |
| huangqi | MOL000392 | formononetin | CHRM1 |
| huangqi | MOL000392 | formononetin | ESR1 |
| huangqi | MOL000392 | formononetin | AR |
| huangqi | MOL000392 | formononetin | PPARG |
| huangqi | MOL000392 | formononetin | PTGS2 |
| huangqi | MOL000392 | formononetin | RXRA |
| huangqi | MOL000392 | formononetin | ADRA1A |
| huangqi | MOL000392 | formononetin | SLC6A3 |
| huangqi | MOL000392 | formononetin | ADRB2 |
| huangqi | MOL000392 | formononetin | SLC6A4 |
| huangqi | MOL000392 | formononetin | ESR2 |
| huangqi | MOL000392 | formononetin | DPP4 |
| huangqi | MOL000392 | formononetin | MAPK14 |
| huangqi | MOL000392 | formononetin | GSK3B |
| huangqi | MOL000392 | formononetin | HSP90AB1 |
| huangqi | MOL000392 | formononetin | CDK2 |
| huangqi | MOL000392 | formononetin | MAOB |
| huangqi | MOL000392 | formononetin | CHEK1 |
| huangqi | MOL000392 | formononetin | PRSS1 |
| huangqi | MOL000392 | formononetin | CCNA2 |
| huangqi | MOL000392 | formononetin | CAMKMT |
| huangqi | MOL000392 | formononetin | PKIA |
| huangqi | MOL000392 | formononetin | ACHE |
| huangqi | MOL000392 | formononetin | DPEP1 |
| huangqi | MOL000392 | formononetin | JUN |
| huangqi | MOL000392 | formononetin | PPARG |
| huangqi | MOL000392 | formononetin | IL4 |
| huangqi | MOL000392 | formononetin | ATP5F1B |
| huangqi | MOL000392 | formononetin | HSD3B2 |
| huangqi | MOL000392 | formononetin | HSD3B1 |
| huangqi | MOL000417 | Calycosin | NOS2 |
| huangqi | MOL000417 | Calycosin | PTGS1 |
| huangqi | MOL000417 | Calycosin | ESR1 |
| huangqi | MOL000417 | Calycosin | AR |
| huangqi | MOL000417 | Calycosin | PPARG |
| huangqi | MOL000417 | Calycosin | PTGS2 |
| huangqi | MOL000417 | Calycosin | RXRA |
| huangqi | MOL000417 | Calycosin | ESR2 |
| huangqi | MOL000417 | Calycosin | DPP4 |
| huangqi | MOL000417 | Calycosin | MAPK14 |
| huangqi | MOL000417 | Calycosin | GSK3B |
| huangqi | MOL000417 | Calycosin | HSP90AB1 |
| huangqi | MOL000417 | Calycosin | CDK2 |
| huangqi | MOL000417 | Calycosin | CHEK1 |
| huangqi | MOL000417 | Calycosin | PRSS1 |
| huangqi | MOL000417 | Calycosin | CCNA2 |
| huangqi | MOL000417 | Calycosin | NCOA2 |
| huangqi | MOL000417 | Calycosin | CAMKMT |
| huangqi | MOL000417 | Calycosin | ADRB2 |
| huangqi | MOL000422 | kaempferol | NOS2 |
| huangqi | MOL000422 | kaempferol | PTGS1 |
| huangqi | MOL000422 | kaempferol | AR |
| huangqi | MOL000422 | kaempferol | PPARG |
| huangqi | MOL000422 | kaempferol | PTGS2 |
| huangqi | MOL000422 | kaempferol | HSP90AB1 |
| huangqi | MOL000422 | kaempferol | NCOA2 |
| huangqi | MOL000422 | kaempferol | DPP4 |
| huangqi | MOL000422 | kaempferol | PRSS1 |
| huangqi | MOL000422 | kaempferol | PGR |
| huangqi | MOL000422 | kaempferol | CHRM1 |
| huangqi | MOL000422 | kaempferol | ACHE |
| huangqi | MOL000422 | kaempferol | SLC6A2 |
| huangqi | MOL000422 | kaempferol | CHRM2 |
| huangqi | MOL000422 | kaempferol | ADRA1B |
| huangqi | MOL000422 | kaempferol | GABRA1 |
| huangqi | MOL000422 | kaempferol | F7 |
| huangqi | MOL000422 | kaempferol | CAMKMT |
| huangqi | MOL000422 | kaempferol | RELA |
| huangqi | MOL000422 | kaempferol | IKBKB |
| huangqi | MOL000422 | kaempferol | AKT1 |
| huangqi | MOL000422 | kaempferol | BCL2 |
| huangqi | MOL000422 | kaempferol | BAX |
| huangqi | MOL000422 | kaempferol | TNFAIP6 |
| huangqi | MOL000422 | kaempferol | JUN |
| huangqi | MOL000422 | kaempferol | AHSA1 |
| huangqi | MOL000422 | kaempferol | CASP3 |
| huangqi | MOL000422 | kaempferol | MAPK8 |
| huangqi | MOL000422 | kaempferol | MMP1 |
| huangqi | MOL000422 | kaempferol | STAT1 |
| huangqi | MOL000422 | kaempferol | CDK1 |
| huangqi | MOL000422 | kaempferol | PPARG |
| huangqi | MOL000422 | kaempferol | HMOX1 |
| huangqi | MOL000422 | kaempferol | CYP3A4 |
| huangqi | MOL000422 | kaempferol | CYP1A2 |
| huangqi | MOL000422 | kaempferol | CYP1A1 |
| huangqi | MOL000422 | kaempferol | ICAM1 |
| huangqi | MOL000422 | kaempferol | SELE |
| huangqi | MOL000422 | kaempferol | VCAM1 |
| huangqi | MOL000422 | kaempferol | NR1I2 |
| huangqi | MOL000422 | kaempferol | CYP1B1 |
| huangqi | MOL000422 | kaempferol | ALOX5 |
| huangqi | MOL000422 | kaempferol | HAS2 |
| huangqi | MOL000422 | kaempferol | GSTP1 |
| huangqi | MOL000422 | kaempferol | AHR |
| huangqi | MOL000422 | kaempferol | PSMD3 |
| huangqi | MOL000422 | kaempferol | SLC2A4 |
| huangqi | MOL000422 | kaempferol | NR1I3 |
| huangqi | MOL000422 | kaempferol | INSRR |
| huangqi | MOL000422 | kaempferol | DIO1 |
| huangqi | MOL000422 | kaempferol | PPP3CA |
| huangqi | MOL000422 | kaempferol | GSTM1 |
| huangqi | MOL000422 | kaempferol | GSTM2 |
| huangqi | MOL000422 | kaempferol | AKR1C3 |
| huangqi | MOL000422 | kaempferol | SLPI |
| huangqi | MOL000433 | FA | CDK2 |
| huangqi | MOL000433 | FA | GSK3B |
| huangqi | MOL000442 | 1,7-Dihydroxy-3,9-dimethoxy pterocarpene | PTGS2 |
| huangqi | MOL000442 | 1,7-Dihydroxy-3,9-dimethoxy pterocarpene | RXRA |
| huangqi | MOL000442 | 1,7-Dihydroxy-3,9-dimethoxy pterocarpene | HSP90AB1 |
| huangqi | MOL000442 | 1,7-Dihydroxy-3,9-dimethoxy pterocarpene | PRSS1 |
| huangqi | MOL000098 | quercetin | PTGS1 |
| huangqi | MOL000098 | quercetin | AR |
| huangqi | MOL000098 | quercetin | PPARG |
| huangqi | MOL000098 | quercetin | PTGS2 |
| huangqi | MOL000098 | quercetin | HSP90AB1 |
| huangqi | MOL000098 | quercetin | NCOA2 |
| huangqi | MOL000098 | quercetin | DPP4 |
| huangqi | MOL000098 | quercetin | AKR1B1 |
| huangqi | MOL000098 | quercetin | PRSS1 |
| huangqi | MOL000098 | quercetin | KCNH2 |
| huangqi | MOL000098 | quercetin | SCN5A |
| huangqi | MOL000098 | quercetin | ADRB2 |
| huangqi | MOL000098 | quercetin | MMP3 |
| huangqi | MOL000098 | quercetin | F7 |
| huangqi | MOL000098 | quercetin | RXRA |
| huangqi | MOL000098 | quercetin | ACHE |
| huangqi | MOL000098 | quercetin | GABRA1 |
| huangqi | MOL000098 | quercetin | MAOB |
| huangqi | MOL000098 | quercetin | RELA |
| huangqi | MOL000098 | quercetin | EGFR |
| huangqi | MOL000098 | quercetin | AKT1 |
| huangqi | MOL000098 | quercetin | VEGFA |
| huangqi | MOL000098 | quercetin | CCND1 |
| huangqi | MOL000098 | quercetin | BCL2 |
| huangqi | MOL000098 | quercetin | BCL2L1 |
| huangqi | MOL000098 | quercetin | FOS |
| huangqi | MOL000098 | quercetin | CDKN1A |
| huangqi | MOL000098 | quercetin | EIF6 |
| huangqi | MOL000098 | quercetin | BAX |
| huangqi | MOL000098 | quercetin | CASP9 |
| huangqi | MOL000098 | quercetin | PLAU |
| huangqi | MOL000098 | quercetin | MMP2 |
| huangqi | MOL000098 | quercetin | MMP9 |
| huangqi | MOL000098 | quercetin | MAPK1 |
| huangqi | MOL000098 | quercetin | IL10RA |
| huangqi | MOL000098 | quercetin | EGF |
| huangqi | MOL000098 | quercetin | RB1 |
| huangqi | MOL000098 | quercetin | TNFAIP6 |
| huangqi | MOL000098 | quercetin | JUN |
| huangqi | MOL000098 | quercetin | IL6R |
| huangqi | MOL000098 | quercetin | AHSA1 |
| huangqi | MOL000098 | quercetin | CASP3 |
| huangqi | MOL000098 | quercetin | TP53 |
| huangqi | MOL000098 | quercetin | ELK1 |
| huangqi | MOL000098 | quercetin | NFKBIA |
| huangqi | MOL000098 | quercetin | POR |
| huangqi | MOL000098 | quercetin | ODC1 |
| huangqi | MOL000098 | quercetin | CASP8 |
| huangqi | MOL000098 | quercetin | TOP1 |
| huangqi | MOL000098 | quercetin | RAF1 |
| huangqi | MOL000098 | quercetin | SOD1 |
| huangqi | MOL000098 | quercetin | PRKCA |
| huangqi | MOL000098 | quercetin | MMP1 |
| huangqi | MOL000098 | quercetin | HIF1A |
| huangqi | MOL000098 | quercetin | STAT1 |
| huangqi | MOL000098 | quercetin | RUNX1T1 |
| huangqi | MOL000098 | quercetin | CDK1 |
| huangqi | MOL000098 | quercetin | HSPA5 |
| huangqi | MOL000098 | quercetin | ERBB2 |
| huangqi | MOL000098 | quercetin | PPARG |
| huangqi | MOL000098 | quercetin | ACACA |
| huangqi | MOL000098 | quercetin | HMOX1 |
| huangqi | MOL000098 | quercetin | CYP3A4 |
| huangqi | MOL000098 | quercetin | CYP1A2 |
| huangqi | MOL000098 | quercetin | CAV1 |
| huangqi | MOL000098 | quercetin | MYC |
| huangqi | MOL000098 | quercetin | F3 |
| huangqi | MOL000098 | quercetin | GJA1 |
| huangqi | MOL000098 | quercetin | CYP1A1 |
| huangqi | MOL000098 | quercetin | ICAM1 |
| huangqi | MOL000098 | quercetin | IL1B |
| huangqi | MOL000098 | quercetin | CCL2 |
| huangqi | MOL000098 | quercetin | SELE |
| huangqi | MOL000098 | quercetin | VCAM1 |
| huangqi | MOL000098 | quercetin | PTGER3 |
| huangqi | MOL000098 | quercetin | CXCL8 |
| huangqi | MOL000098 | quercetin | PRKCB |
| huangqi | MOL000098 | quercetin | BIRC5 |
| huangqi | MOL000098 | quercetin | DUOX2 |
| huangqi | MOL000098 | quercetin | NOS3 |
| huangqi | MOL000098 | quercetin | HSPB1 |
| huangqi | MOL000098 | quercetin | SULT1E1 |
| huangqi | MOL000098 | quercetin | IL2RA |
| huangqi | MOL000098 | quercetin | NR1I2 |
| huangqi | MOL000098 | quercetin | CYP1B1 |
| huangqi | MOL000098 | quercetin | CCNB1 |
| huangqi | MOL000098 | quercetin | PLAT |
| huangqi | MOL000098 | quercetin | THBD |
| huangqi | MOL000098 | quercetin | SERPINE1 |
| huangqi | MOL000098 | quercetin | COL1A1 |
| huangqi | MOL000098 | quercetin | IFNG |
| huangqi | MOL000098 | quercetin | ALOX5 |
| huangqi | MOL000098 | quercetin | IL1A |
| huangqi | MOL000098 | quercetin | MPO |
| huangqi | MOL000098 | quercetin | TOP2A |
| huangqi | MOL000098 | quercetin | NCF1 |
| huangqi | MOL000098 | quercetin | ABCG2 |
| huangqi | MOL000098 | quercetin | HAS2 |
| huangqi | MOL000098 | quercetin | GSTP1 |
| huangqi | MOL000098 | quercetin | NFE2L2 |
| huangqi | MOL000098 | quercetin | NQO1 |
| huangqi | MOL000098 | quercetin | PARP1 |
| huangqi | MOL000098 | quercetin | AHR |
| huangqi | MOL000098 | quercetin | PSMD3 |
| huangqi | MOL000098 | quercetin | SLC2A4 |
| huangqi | MOL000098 | quercetin | COL3A1 |
| huangqi | MOL000098 | quercetin | CXCL11 |
| huangqi | MOL000098 | quercetin | CXCL2 |
| huangqi | MOL000098 | quercetin | DCAF5 |
| huangqi | MOL000098 | quercetin | NR1I3 |
| huangqi | MOL000098 | quercetin | CHEK2 |
| huangqi | MOL000098 | quercetin | INSRR |
| huangqi | MOL000098 | quercetin | CLDN4 |
| huangqi | MOL000098 | quercetin | PPARA |
| huangqi | MOL000098 | quercetin | PPARD |
| huangqi | MOL000098 | quercetin | HSF1 |
| huangqi | MOL000098 | quercetin | CXCL10 |
| huangqi | MOL000098 | quercetin | CHUK |
| huangqi | MOL000098 | quercetin | SPP1 |
| huangqi | MOL000098 | quercetin | RUNX2 |
| huangqi | MOL000098 | quercetin | RASSF1 |
| huangqi | MOL000098 | quercetin | E2F1 |
| huangqi | MOL000098 | quercetin | E2F2 |
| huangqi | MOL000098 | quercetin | ACP3 |
| huangqi | MOL000098 | quercetin | CTSD |
| huangqi | MOL000098 | quercetin | IGFBP3 |
| huangqi | MOL000098 | quercetin | IGF2 |
| huangqi | MOL000098 | quercetin | CD40LG |
| huangqi | MOL000098 | quercetin | IRF1 |
| huangqi | MOL000098 | quercetin | ERBB3 |
| huangqi | MOL000098 | quercetin | PON1 |
| huangqi | MOL000098 | quercetin | DIO1 |
| huangqi | MOL000098 | quercetin | PCOLCE |
| huangqi | MOL000098 | quercetin | NPEPPS |
| huangqi | MOL000098 | quercetin | HK2 |
| huangqi | MOL000098 | quercetin | RASA1 |
| huangqi | MOL000098 | quercetin | GSTM1 |
| huangqi | MOL000098 | quercetin | GSTM2 |
| zhike | MOL013381 | Marmin | PTGS2 |
| zhike | MOL013381 | Marmin | CA2 |
| zhike | MOL013381 | Marmin | ADRB2 |
| zhike | MOL002341 | Hesperetin | PTGS1 |
| zhike | MOL002341 | Hesperetin | SCN5A |
| zhike | MOL002341 | Hesperetin | PTGS2 |
| zhike | MOL002341 | Hesperetin | HSP90AB1 |
| zhike | MOL002341 | Hesperetin | NCOA2 |
| zhike | MOL002341 | Hesperetin | NCOA1 |
| zhike | MOL002341 | Hesperetin | CAMKMT |
| zhike | MOL000358 | beta-sitosterol | PGR |
| zhike | MOL000358 | beta-sitosterol | NCOA2 |
| zhike | MOL000358 | beta-sitosterol | PTGS1 |
| zhike | MOL000358 | beta-sitosterol | PTGS2 |
| zhike | MOL000358 | beta-sitosterol | HSP90AB1 |
| zhike | MOL000358 | beta-sitosterol | KCNH2 |
| zhike | MOL000358 | beta-sitosterol | DRD1 |
| zhike | MOL000358 | beta-sitosterol | CHRM3 |
| zhike | MOL000358 | beta-sitosterol | CHRM1 |
| zhike | MOL000358 | beta-sitosterol | SCN5A |
| zhike | MOL000358 | beta-sitosterol | CHRM4 |
| zhike | MOL000358 | beta-sitosterol | ADRA1A |
| zhike | MOL000358 | beta-sitosterol | CHRM2 |
| zhike | MOL000358 | beta-sitosterol | ADRA1B |
| zhike | MOL000358 | beta-sitosterol | ADRB2 |
| zhike | MOL000358 | beta-sitosterol | CHRNA2 |
| zhike | MOL000358 | beta-sitosterol | SLC6A4 |
| zhike | MOL000358 | beta-sitosterol | OPRM1 |
| zhike | MOL000358 | beta-sitosterol | GABRA1 |
| zhike | MOL000358 | beta-sitosterol | BCL2 |
| zhike | MOL000358 | beta-sitosterol | BAX |
| zhike | MOL000358 | beta-sitosterol | CASP9 |
| zhike | MOL000358 | beta-sitosterol | JUN |
| zhike | MOL000358 | beta-sitosterol | CASP3 |
| zhike | MOL000358 | beta-sitosterol | CASP8 |
| zhike | MOL000358 | beta-sitosterol | PRKCA |
| zhike | MOL000358 | beta-sitosterol | PON1 |
| zhike | MOL000358 | beta-sitosterol | MAP2 |
| zhike | MOL004328 | naringenin | PTGS1 |
| zhike | MOL004328 | naringenin | ESR1 |
| zhike | MOL004328 | naringenin | PTGS2 |
| zhike | MOL004328 | naringenin | HSP90AB1 |
| zhike | MOL004328 | naringenin | DPEP1 |
| zhike | MOL004328 | naringenin | RELA |
| zhike | MOL004328 | naringenin | AKT1 |
| zhike | MOL004328 | naringenin | BCL2 |
| zhike | MOL004328 | naringenin | MAPK3 |
| zhike | MOL004328 | naringenin | MAPK1 |
| zhike | MOL004328 | naringenin | CASP3 |
| zhike | MOL004328 | naringenin | FASN |
| zhike | MOL004328 | naringenin | LDLR |
| zhike | MOL004328 | naringenin | BAD |
| zhike | MOL004328 | naringenin | SOD1 |
| zhike | MOL004328 | naringenin | CAT |
| zhike | MOL004328 | naringenin | PPARG |
| zhike | MOL004328 | naringenin | MTTP |
| zhike | MOL004328 | naringenin | APOB |
| zhike | MOL004328 | naringenin | PLB1 |
| zhike | MOL004328 | naringenin | HMGCR |
| zhike | MOL004328 | naringenin | CYP19A1 |
| zhike | MOL004328 | naringenin | GSTP1 |
| zhike | MOL004328 | naringenin | UGT1A1 |
| zhike | MOL004328 | naringenin | PPARA |
| zhike | MOL004328 | naringenin | SREBF1 |
| zhike | MOL004328 | naringenin | GSR |
| zhike | MOL004328 | naringenin | ABCC1 |
| zhike | MOL004328 | naringenin | ADIPOR2 |
| zhike | MOL004328 | naringenin | SOAT2 |
| zhike | MOL004328 | naringenin | AKR1C1 |
| zhike | MOL004328 | naringenin | GOT1 |
| zhike | MOL004328 | naringenin | ABAT |
| zhike | MOL004328 | naringenin | CES1 |
| zhike | MOL004328 | naringenin | SOAT1 |
| zhike | MOL005828 | nobiletin | NOS2 |
| zhike | MOL005828 | nobiletin | PTGS1 |
| zhike | MOL005828 | nobiletin | KCNH2 |
| zhike | MOL005828 | nobiletin | ESR1 |
| zhike | MOL005828 | nobiletin | AR |
| zhike | MOL005828 | nobiletin | PPARG |
| zhike | MOL005828 | nobiletin | PTGS2 |
| zhike | MOL005828 | nobiletin | F7 |
| zhike | MOL005828 | nobiletin | ESR2 |
| zhike | MOL005828 | nobiletin | DPP4 |
| zhike | MOL005828 | nobiletin | HSP90AB1 |
| zhike | MOL005828 | nobiletin | CHEK1 |
| zhike | MOL005828 | nobiletin | PRSS1 |
| zhike | MOL005828 | nobiletin | NCOA2 |
| zhike | MOL005828 | nobiletin | CAMKMT |
| zhike | MOL005828 | nobiletin | GSK3B |
| zhike | MOL005828 | nobiletin | SCN5A |
| zhike | MOL005828 | nobiletin | BCL2 |
| zhike | MOL005828 | nobiletin | BAX |
| zhike | MOL005828 | nobiletin | CASP9 |
| zhike | MOL005828 | nobiletin | MMP9 |
| zhike | MOL005828 | nobiletin | JUN |
| zhike | MOL005828 | nobiletin | TP53 |
| zhike | MOL005828 | nobiletin | MAPK8 |
| zhike | MOL005828 | nobiletin | TIMP1 |
| zhike | MOL005828 | nobiletin | PPARG |
| zhike | MOL005828 | nobiletin | CREB1 |
| zhike | MOL005828 | nobiletin | PLA2G4A |
| zhike | MOL005828 | nobiletin | CD163 |
| zhike | MOL005828 | nobiletin | EPHB2 |
