# Supplementary material for: Explore the active ingredients and potential mechanisms of JianPi QingRe HuaYu Methods in the treatment of gastric inflammation-cancer transformation by network pharmacology and experimental validation
Source: BMC Complement Med Ther. 2023 Nov 14;23:411. doi: 10.1186/s12906-023-04232-0 (PMC10644588; doi:10.1186/s12906-023-04232-0)
Supplement: Supplementary file 3 — Additional file 3: Table S3. The DEGs between IN and gastritis group In GSE130823 dataset. [file 12906_2023_4232_MOESM3_ESM.docx]

**Table S3. The DEGs between IN and gastritis group In GSE130823 dataset.**

| Genes | logFC | AveExpr | t | P.Value | adj.P.Val | B |
| --- | --- | --- | --- | --- | --- | --- |
| APOBEC2 | -3.57223 | 0.025164 | -8.87872 | 1.77E-13 | 5.67E-09 | 20.18962 |
| EVL | -1.58688 | -0.04761 | -8.61749 | 5.71E-13 | 6.40E-09 | 19.07662 |
| PREX1 | -1.22726 | -0.12411 | -8.60691 | 5.98E-13 | 6.40E-09 | 19.03151 |
| LOC100506219 | 2.178574 | 0.356405 | 8.488377 | 1.02E-12 | 8.16E-09 | 18.52622 |
| RORA | -1.29672 | -0.11155 | -8.43024 | 1.32E-12 | 8.48E-09 | 18.27834 |
| XLOC_014161 | 1.818038 | -0.29478 | 8.200623 | 3.70E-12 | 1.98E-08 | 17.29961 |
| UBXN10 | 1.85255 | 0.368296 | 8.094496 | 5.94E-12 | 2.72E-08 | 16.84749 |
| SYTL3 | -1.63229 | 0.184743 | -7.91064 | 1.35E-11 | 5.42E-08 | 16.06498 |
| GIF | -7.0511 | -0.65848 | -7.84612 | 1.80E-11 | 6.43E-08 | 15.79062 |
| PPP1R1B | 3.170639 | -0.64046 | 7.546852 | 6.85E-11 | 2.00E-07 | 14.52095 |
| CSF1R | -1.31197 | -0.05438 | -7.52179 | 7.65E-11 | 2.03E-07 | 14.41488 |
| MEST | 1.49367 | -0.11854 | 7.505136 | 8.24E-11 | 2.03E-07 | 14.3444 |
| SNRPN | -1.70512 | -0.39579 | -7.47276 | 9.52E-11 | 2.04E-07 | 14.20745 |
| GPR155 | -2.78468 | 0.705197 | -7.47267 | 9.52E-11 | 2.04E-07 | 14.20708 |
| ATP4B | -7.18113 | -0.08549 | -7.42123 | 1.20E-10 | 2.40E-07 | 13.98969 |
| HOXB13 | 4.850134 | 0.755146 | 7.349086 | 1.65E-10 | 2.86E-07 | 13.68512 |
| XLOC_005220 | 2.064263 | -0.50986 | 7.342952 | 1.69E-10 | 2.86E-07 | 13.65924 |
| SNURF | -1.77901 | -0.11757 | -7.33011 | 1.79E-10 | 2.87E-07 | 13.60511 |
| C19orf45 | 1.98234 | 0.365208 | 7.305856 | 1.99E-10 | 2.88E-07 | 13.50284 |
| PKDCC | 1.460722 | -0.20668 | 7.269302 | 2.34E-10 | 3.13E-07 | 13.34884 |
| ATP4A | -7.12842 | -1.24782 | -7.25166 | 2.53E-10 | 3.23E-07 | 13.27455 |
| ZNF703 | 1.931358 | -0.32895 | 7.236578 | 2.71E-10 | 3.23E-07 | 13.21107 |
| SERPINB5 | 4.11605 | -1.35968 | 7.235953 | 2.72E-10 | 3.23E-07 | 13.20844 |
| DOCK2 | -1.29362 | -0.00361 | -7.2117 | 3.02E-10 | 3.46E-07 | 13.10641 |
| LOC157860 | 2.491056 | 1.004864 | 7.195061 | 3.25E-10 | 3.60E-07 | 13.03645 |
| TRIM50 | -4.21781 | 0.615085 | -7.16729 | 3.68E-10 | 3.93E-07 | 12.91974 |
| HOXA11-AS1 | 4.409701 | 2.142476 | 7.155081 | 3.88E-10 | 4.02E-07 | 12.86845 |
| LOC100506412 | -5.17853 | 2.182143 | -7.1466 | 4.03E-10 | 4.04E-07 | 12.83282 |
| PTF1A | -2.57011 | 0.456681 | -7.12297 | 4.47E-10 | 4.19E-07 | 12.73365 |
| GADD45B | -1.53835 | 0.132993 | -7.11777 | 4.58E-10 | 4.19E-07 | 12.71179 |
| ST3GAL1 | -2.03949 | 0.074679 | -7.08229 | 5.35E-10 | 4.75E-07 | 12.56299 |
| CMAHP | -1.40283 | -0.00159 | -7.07697 | 5.48E-10 | 4.75E-07 | 12.54071 |
| LOC400043 | -2.19713 | -0.2747 | -7.0496 | 6.18E-10 | 5.06E-07 | 12.426 |
| KCNE2 | -3.58203 | -0.04052 | -7.04766 | 6.23E-10 | 5.06E-07 | 12.41789 |
| KDM1B | 1.263766 | -0.12744 | 7.044758 | 6.31E-10 | 5.06E-07 | 12.40574 |
| TRIM54 | 3.757584 | 0.577438 | 6.983692 | 8.25E-10 | 6.30E-07 | 12.15021 |
| ATP2C2 | 1.813736 | -0.25036 | 6.971376 | 8.71E-10 | 6.37E-07 | 12.09872 |
| PGA3 | -5.21319 | -0.20375 | -6.97051 | 8.74E-10 | 6.37E-07 | 12.09511 |
| CLDN3 | 4.370703 | 0.166181 | 6.956705 | 9.29E-10 | 6.62E-07 | 12.03742 |
| HOXC4 | -1.78689 | -0.6017 | -6.93966 | 1.00E-09 | 6.72E-07 | 11.96622 |
| XLOC_011893 | 1.630584 | -0.13375 | 6.93282 | 1.03E-09 | 6.75E-07 | 11.93768 |
| LAMA2 | -2.20342 | 0.145111 | -6.92496 | 1.07E-09 | 6.85E-07 | 11.90486 |
| MFSD4 | -3.15972 | 0.594137 | -6.92041 | 1.09E-09 | 6.85E-07 | 11.88588 |
| F12 | 2.671064 | 0.104166 | 6.894224 | 1.22E-09 | 7.17E-07 | 11.77665 |
| LOC150622 | -2.33177 | 0.369183 | -6.89288 | 1.23E-09 | 7.17E-07 | 11.77106 |
| 3-3月 | 1.542626 | -0.3008 | 6.889915 | 1.24E-09 | 7.17E-07 | 11.75869 |
| LOC100506965 | -1.72238 | -0.08986 | -6.88873 | 1.25E-09 | 7.17E-07 | 11.75374 |
| PAR-SN | -1.88113 | -0.20053 | -6.88353 | 1.28E-09 | 7.20E-07 | 11.73206 |
| RASAL3 | -1.10603 | -0.01127 | -6.87378 | 1.34E-09 | 7.39E-07 | 11.69142 |
| SYNGR1 | -1.63839 | 0.084096 | -6.86704 | 1.38E-09 | 7.48E-07 | 11.66335 |
| XLOC_l2_000080 | -2.20947 | 0.897322 | -6.84879 | 1.49E-09 | 7.85E-07 | 11.58734 |
| CAMK2N1 | 2.583736 | -0.17586 | 6.84829 | 1.49E-09 | 7.85E-07 | 11.58526 |
| ARHGEF38 | 1.007933 | 0.130413 | 6.839306 | 1.55E-09 | 8.04E-07 | 11.54786 |
| DRD5 | -4.35941 | 1.452457 | -6.83112 | 1.61E-09 | 8.20E-07 | 11.5138 |
| MYRIP | -2.35894 | 0.404975 | -6.8244 | 1.66E-09 | 8.31E-07 | 11.48585 |
| PLCL1 | -2.02921 | 0.162319 | -6.81532 | 1.72E-09 | 8.39E-07 | 11.44806 |
| CPA2 | -4.75843 | 0.24237 | -6.81381 | 1.74E-09 | 8.39E-07 | 11.44177 |
| XLOC_006025 | 3.359172 | 1.160564 | 6.811446 | 1.75E-09 | 8.39E-07 | 11.43195 |
| CD3E | -1.95412 | 0.306619 | -6.80712 | 1.79E-09 | 8.39E-07 | 11.41397 |
| SNAI3 | -1.84411 | 0.160505 | -6.79506 | 1.88E-09 | 8.51E-07 | 11.36383 |
| HSD11B2 | 1.416585 | -0.11965 | 6.789556 | 1.93E-09 | 8.60E-07 | 11.34096 |
| BLK | -2.31558 | -0.20871 | -6.77527 | 2.05E-09 | 8.87E-07 | 11.28161 |
| ZMIZ1 | -1.27512 | 0.53654 | -6.77444 | 2.06E-09 | 8.87E-07 | 11.27818 |
| KCNJ15 | -2.66947 | -0.26122 | -6.77306 | 2.07E-09 | 8.87E-07 | 11.27243 |
| LOC100506452 | -2.37932 | 0.339826 | -6.76763 | 2.12E-09 | 8.90E-07 | 11.24988 |
| TFF3 | 3.408701 | -0.35754 | 6.752422 | 2.27E-09 | 9.33E-07 | 11.18674 |
| GPR68 | -1.01766 | -0.01573 | -6.7427 | 2.37E-09 | 9.61E-07 | 11.14639 |
| SLC9A7P1 | -1.2674 | -0.02385 | -6.73303 | 2.47E-09 | 9.90E-07 | 11.1063 |
| CTSL2 | 1.472106 | 0.063916 | 6.719469 | 2.62E-09 | 1.04E-06 | 11.05006 |
| DGKD | -1.32065 | -0.28752 | -6.71469 | 2.67E-09 | 1.05E-06 | 11.03024 |
| SIGLEC11 | -1.76471 | 0.338225 | -6.70131 | 2.84E-09 | 1.08E-06 | 10.9748 |
| FAM20A | -2.28838 | -0.57913 | -6.7001 | 2.85E-09 | 1.08E-06 | 10.9698 |
| KLK8 | 3.952893 | 1.751887 | 6.698298 | 2.87E-09 | 1.08E-06 | 10.96234 |
| GAMT | -2.24459 | -0.13312 | -6.69325 | 2.94E-09 | 1.10E-06 | 10.94142 |
| LOC100507511 | 2.623778 | 0.461967 | 6.689709 | 2.98E-09 | 1.10E-06 | 10.92676 |
| SLC9A7 | -1.24757 | -0.15725 | -6.68502 | 3.04E-09 | 1.10E-06 | 10.90733 |
| SNX22 | -1.45249 | -0.11345 | -6.67873 | 3.13E-09 | 1.11E-06 | 10.88132 |
| CLDN2 | 4.067356 | -0.91996 | 6.677178 | 3.15E-09 | 1.11E-06 | 10.87489 |
| LOC100507507 | 1.235502 | -0.03506 | 6.675339 | 3.17E-09 | 1.11E-06 | 10.86727 |
| LIFR | -2.13895 | 0.186422 | -6.67105 | 3.23E-09 | 1.12E-06 | 10.84953 |
| FLJ42875 | -2.72187 | 0.285303 | -6.66537 | 3.31E-09 | 1.13E-06 | 10.82601 |
| LOC100240734 | -2.32999 | -0.2743 | -6.66375 | 3.34E-09 | 1.13E-06 | 10.81932 |
| TIMP4 | 1.91934 | -0.13625 | 6.661595 | 3.37E-09 | 1.13E-06 | 10.81041 |
| KIAA1257 | 2.172514 | 0.298169 | 6.652015 | 3.51E-09 | 1.16E-06 | 10.77079 |
| CHRNA5 | 2.577766 | -0.52476 | 6.647429 | 3.58E-09 | 1.16E-06 | 10.75182 |
| KIF12 | 1.01867 | -0.11675 | 6.640475 | 3.69E-09 | 1.18E-06 | 10.72308 |
| TMEM37 | -1.87354 | 0.678204 | -6.63889 | 3.72E-09 | 1.18E-06 | 10.71653 |
| GDF5 | -4.13834 | 2.042706 | -6.63137 | 3.84E-09 | 1.21E-06 | 10.68543 |
| GSTM2 | -1.40749 | -0.05494 | -6.61565 | 4.11E-09 | 1.28E-06 | 10.62053 |
| LOC389332 | 3.138881 | -0.93477 | 6.596488 | 4.47E-09 | 1.38E-06 | 10.54142 |
| HENMT1 | 1.42248 | -0.08517 | 6.591291 | 4.57E-09 | 1.39E-06 | 10.51998 |
| FZD4 | -1.45004 | 0.173798 | -6.58658 | 4.67E-09 | 1.40E-06 | 10.50055 |
| RCOR2 | 1.282609 | 0.19484 | 6.583936 | 4.72E-09 | 1.40E-06 | 10.48963 |
| CDH2 | -2.85501 | 1.189095 | -6.56596 | 5.10E-09 | 1.49E-06 | 10.41554 |
| EFEMP1 | -1.72048 | -0.1505 | -6.56517 | 5.12E-09 | 1.49E-06 | 10.41228 |
| PTPN13 | -1.58814 | -0.25247 | -6.56127 | 5.21E-09 | 1.50E-06 | 10.39618 |
| ONECUT3 | 2.4121 | 0.075137 | 6.555374 | 5.34E-09 | 1.52E-06 | 10.37191 |
| HDC | -3.07311 | 0.485962 | -6.5547 | 5.36E-09 | 1.52E-06 | 10.36911 |
| LOC100505974 | -2.87443 | 0.550787 | -6.54879 | 5.50E-09 | 1.55E-06 | 10.34478 |
| ECHDC3 | -1.8906 | -0.19876 | -6.54237 | 5.65E-09 | 1.58E-06 | 10.31836 |
| ARHGEF17 | -1.43897 | 0.068216 | -6.54025 | 5.70E-09 | 1.58E-06 | 10.3096 |
| CKMT2 | -3.69642 | 0.336864 | -6.53325 | 5.88E-09 | 1.61E-06 | 10.28082 |
| MICALL1 | -1.22374 | -0.18759 | -6.51992 | 6.23E-09 | 1.68E-06 | 10.22594 |
| SKAP2 | 1.231401 | -0.3794 | 6.519664 | 6.23E-09 | 1.68E-06 | 10.2249 |
| CCKBR | -3.85942 | -0.15999 | -6.51451 | 6.37E-09 | 1.68E-06 | 10.2037 |
| ONECUT2 | 3.414292 | 0.224745 | 6.51165 | 6.45E-09 | 1.68E-06 | 10.19194 |
| CHST11 | -1.72773 | -0.43285 | -6.51085 | 6.48E-09 | 1.68E-06 | 10.18864 |
| CYB5R1 | -1.35965 | 0.275396 | -6.5106 | 6.48E-09 | 1.68E-06 | 10.18762 |
| FAM46C | -1.09956 | 0.017614 | -6.47874 | 7.44E-09 | 1.88E-06 | 10.05671 |
| ITGB8 | 1.423794 | -0.06427 | 6.478129 | 7.46E-09 | 1.88E-06 | 10.05419 |
| PPP3CC | -1.32059 | -0.1875 | -6.47173 | 7.67E-09 | 1.92E-06 | 10.0279 |
| CDK14 | -1.57452 | -0.00659 | -6.45665 | 8.18E-09 | 2.03E-06 | 9.96601 |
| ZNF793 | -1.89745 | -0.49121 | -6.45532 | 8.23E-09 | 2.03E-06 | 9.960581 |
| C5 | -1.72741 | 0.252725 | -6.44123 | 8.74E-09 | 2.11E-06 | 9.902805 |
| SH3GL2 | -3.37637 | 0.972675 | -6.43367 | 9.03E-09 | 2.13E-06 | 9.87178 |
| BEX4 | -1.47436 | -0.37888 | -6.43114 | 9.13E-09 | 2.14E-06 | 9.861448 |
| BHLHE41 | 1.694218 | 0.089362 | 6.428723 | 9.23E-09 | 2.15E-06 | 9.851526 |
| CLIC6 | -3.36656 | 0.845024 | -6.42581 | 9.34E-09 | 2.16E-06 | 9.839584 |
| HS3ST4 | -1.46008 | 0.84014 | -6.42215 | 9.49E-09 | 2.16E-06 | 9.824602 |
| SMARCD3 | -1.99243 | 0.302106 | -6.42207 | 9.50E-09 | 2.16E-06 | 9.824248 |
| DNAJC12 | 1.392559 | 0.098606 | 6.415742 | 9.76E-09 | 2.19E-06 | 9.798348 |
| TMEM184A | -2.52002 | 1.397106 | -6.41526 | 9.78E-09 | 2.19E-06 | 9.796394 |
| XLOC_l2_008546 | 1.184463 | -0.22756 | 6.412885 | 9.88E-09 | 2.20E-06 | 9.786648 |
| IGFALS | -2.78095 | 0.170056 | -6.41091 | 9.96E-09 | 2.20E-06 | 9.778575 |
| C2orf82 | 1.826516 | 0.006379 | 6.390312 | 1.09E-08 | 2.38E-06 | 9.694263 |
| RBPMS2 | -2.70774 | 0.038509 | -6.38572 | 1.11E-08 | 2.39E-06 | 9.675495 |
| LOC100506948 | -1.61643 | -0.36334 | -6.38482 | 1.11E-08 | 2.39E-06 | 9.671798 |
| LOC100505908 | 1.910298 | 0.564518 | 6.381339 | 1.13E-08 | 2.40E-06 | 9.657565 |
| BEX2 | -2.17014 | -0.46252 | -6.37337 | 1.17E-08 | 2.45E-06 | 9.624993 |
| AQP4 | -3.23648 | 1.142358 | -6.37257 | 1.17E-08 | 2.45E-06 | 9.621734 |
| XLOC_004339 | -3.19694 | 2.256258 | -6.3717 | 1.18E-08 | 2.45E-06 | 9.61817 |
| HOXA13 | 4.089826 | 1.825422 | 6.371166 | 1.18E-08 | 2.45E-06 | 9.615977 |
| ARMCX1 | -1.37397 | -0.19506 | -6.36404 | 1.22E-08 | 2.49E-06 | 9.586879 |
| MYB | 1.165538 | 0.22309 | 6.349655 | 1.30E-08 | 2.63E-06 | 9.528107 |
| DPP4 | 2.591043 | 0.088004 | 6.341484 | 1.34E-08 | 2.69E-06 | 9.494749 |
| PLBD1 | 1.013046 | -0.19684 | 6.338456 | 1.36E-08 | 2.69E-06 | 9.48239 |
| PTPRM | -1.36791 | 0.026214 | -6.33637 | 1.37E-08 | 2.70E-06 | 9.473867 |
| XLOC_l2_002611 | -3.40013 | 1.721211 | -6.33263 | 1.39E-08 | 2.72E-06 | 9.458613 |
| PRR19 | 1.273021 | -0.10279 | 6.331845 | 1.40E-08 | 2.72E-06 | 9.455416 |
| XLOC_006544 | 1.186201 | 0.191563 | 6.328804 | 1.42E-08 | 2.74E-06 | 9.443012 |
| SLC1A2 | -2.1545 | 0.452735 | -6.3235 | 1.45E-08 | 2.79E-06 | 9.42139 |
| XLOC_009075 | -1.53708 | 0.643242 | -6.32153 | 1.46E-08 | 2.79E-06 | 9.413344 |
| ACADL | -1.99813 | -0.17754 | -6.31814 | 1.48E-08 | 2.80E-06 | 9.399512 |
| GRIN2D | 3.218064 | -0.10722 | 6.313313 | 1.52E-08 | 2.84E-06 | 9.379844 |
| PLIN5 | -3.47094 | 0.166623 | -6.3049 | 1.57E-08 | 2.93E-06 | 9.345545 |
| KCNJ16 | -4.05526 | 0.063328 | -6.29706 | 1.62E-08 | 3.01E-06 | 9.313627 |
| XLOC_004638 | -3.83877 | 2.518839 | -6.28483 | 1.71E-08 | 3.16E-06 | 9.263833 |
| XLOC_006390 | 3.69698 | 2.27039 | 6.27914 | 1.75E-08 | 3.21E-06 | 9.24067 |
| FOXD2 | 1.44786 | -0.20574 | 6.276992 | 1.77E-08 | 3.22E-06 | 9.231927 |
| CLRN3 | 3.922857 | -0.91098 | 6.275942 | 1.78E-08 | 3.22E-06 | 9.227657 |
| FMO2 | -1.68799 | -0.1855 | -6.26656 | 1.85E-08 | 3.32E-06 | 9.189484 |
| XLOC_013835 | -2.82879 | 1.323652 | -6.26599 | 1.86E-08 | 3.32E-06 | 9.18719 |
| CLCNKA | -2.86808 | 0.805037 | -6.25937 | 1.91E-08 | 3.38E-06 | 9.160276 |
| ESRRG | -2.20945 | 0.601443 | -6.25817 | 1.92E-08 | 3.38E-06 | 9.155397 |
| SYN2 | -1.81731 | -0.20987 | -6.24253 | 2.05E-08 | 3.52E-06 | 9.091831 |
| CEACAM7 | 2.158918 | -0.19302 | 6.239624 | 2.08E-08 | 3.52E-06 | 9.080019 |
| GNMT | -2.24882 | -0.07042 | -6.23857 | 2.09E-08 | 3.52E-06 | 9.075746 |
| PALM3 | -2.03912 | 0.189355 | -6.23604 | 2.11E-08 | 3.52E-06 | 9.065483 |
| B3GAT1 | -3.85208 | 0.179544 | -6.2352 | 2.12E-08 | 3.52E-06 | 9.06204 |
| HOXA10 | 1.692854 | 0.520177 | 6.233808 | 2.13E-08 | 3.52E-06 | 9.056401 |
| XLOC_006599 | -3.47746 | 1.726159 | -6.23037 | 2.16E-08 | 3.55E-06 | 9.042459 |
| XLOC_006277 | 3.234338 | -0.01642 | 6.227307 | 2.19E-08 | 3.57E-06 | 9.03001 |
| LINGO2 | -2.48604 | 0.604686 | -6.22678 | 2.19E-08 | 3.57E-06 | 9.02789 |
| GLB1L2 | 1.499832 | -0.29909 | 6.224926 | 2.21E-08 | 3.57E-06 | 9.020345 |
| PNMT | 1.610323 | 0.311633 | 6.22446 | 2.22E-08 | 3.57E-06 | 9.018454 |
| ABHD11 | 1.221779 | 0.012931 | 6.220708 | 2.25E-08 | 3.60E-06 | 9.003229 |
| XLOC_010144 | -5.305 | 3.5826 | -6.22052 | 2.25E-08 | 3.60E-06 | 9.002469 |
| PTGER3 | -3.20784 | 1.484995 | -6.2167 | 2.29E-08 | 3.62E-06 | 8.986975 |
| ZNF385B | -2.39187 | 1.458565 | -6.21399 | 2.32E-08 | 3.64E-06 | 8.975993 |
| XLOC_014216 | -2.82363 | 1.974163 | -6.21251 | 2.33E-08 | 3.64E-06 | 8.96998 |
| KLK6 | 2.940964 | 1.18331 | 6.207529 | 2.38E-08 | 3.69E-06 | 8.949774 |
| GPER | -2.84087 | 0.624598 | -6.20418 | 2.42E-08 | 3.71E-06 | 8.936205 |
| PABPC1L2B | -4.08715 | 2.02216 | -6.19977 | 2.46E-08 | 3.76E-06 | 8.918327 |
| KCNH6 | 1.643331 | 0.263263 | 6.198895 | 2.47E-08 | 3.76E-06 | 8.914772 |
| ZNF132 | -1.19949 | -0.29736 | -6.19656 | 2.50E-08 | 3.78E-06 | 8.905311 |
| XLOC_002647 | -1.36469 | 0.561739 | -6.19484 | 2.51E-08 | 3.78E-06 | 8.898338 |
| HOTTIP | 2.038623 | 1.145524 | 6.192525 | 2.54E-08 | 3.78E-06 | 8.888956 |
| TRIM29 | 3.199943 | -0.8368 | 6.191649 | 2.55E-08 | 3.78E-06 | 8.885405 |
| LOC100128893 | -1.935 | -0.21836 | -6.19062 | 2.56E-08 | 3.78E-06 | 8.881223 |
| NMUR2 | 2.314037 | 0.53692 | 6.186105 | 2.61E-08 | 3.82E-06 | 8.86295 |
| GBGT1 | -1.14646 | 0.14688 | -6.18315 | 2.64E-08 | 3.85E-06 | 8.850999 |
| ETS2 | 1.277071 | 0.098861 | 6.180946 | 2.67E-08 | 3.86E-06 | 8.842055 |
| GUCY2C | 3.362984 | 0.222969 | 6.180437 | 2.67E-08 | 3.86E-06 | 8.839992 |
| TPMT | 1.106466 | 0.164211 | 6.178224 | 2.70E-08 | 3.88E-06 | 8.831034 |
| XLOC_007054 | 1.863979 | 0.293913 | 6.173587 | 2.75E-08 | 3.94E-06 | 8.812261 |
| LOC100507372 | -1.95278 | -0.47839 | -6.17201 | 2.77E-08 | 3.95E-06 | 8.805875 |
| PAQR5 | -2.69126 | 0.42908 | -6.17037 | 2.79E-08 | 3.96E-06 | 8.799259 |
| CHIA | -6.29552 | 3.467427 | -6.16764 | 2.82E-08 | 3.99E-06 | 8.788193 |
| ENPP5 | -2.49754 | -0.11319 | -6.15846 | 2.93E-08 | 4.13E-06 | 8.751037 |
| RASSF2 | -1.0572 | 0.249386 | -6.15689 | 2.95E-08 | 4.14E-06 | 8.744693 |
| XLOC_003441 | 1.465886 | 0.747433 | 6.154708 | 2.98E-08 | 4.16E-06 | 8.735879 |
| IGLL1 | -1.22456 | -0.15604 | -6.152 | 3.02E-08 | 4.17E-06 | 8.724933 |
| SETBP1 | -1.19125 | 0.121594 | -6.14774 | 3.07E-08 | 4.23E-06 | 8.707716 |
| RNF217 | -1.85037 | -0.1734 | -6.14462 | 3.11E-08 | 4.25E-06 | 8.695078 |
| TIMP1 | 1.037198 | -0.26907 | 6.137375 | 3.21E-08 | 4.36E-06 | 8.66582 |
| EGFL6 | -2.51996 | 0.061824 | -6.1312 | 3.30E-08 | 4.46E-06 | 8.640896 |
| PP7080 | -2.5476 | 0.315814 | -6.12948 | 3.32E-08 | 4.46E-06 | 8.633943 |
| NPHS1 | -2.23518 | 0.283664 | -6.12926 | 3.32E-08 | 4.46E-06 | 8.633048 |
| LINC00346 | 1.611065 | 0.131984 | 6.117761 | 3.49E-08 | 4.64E-06 | 8.586622 |
| GRIA4 | -2.29311 | 0.692078 | -6.11541 | 3.52E-08 | 4.64E-06 | 8.577137 |
| SERPING1 | -1.03363 | -0.01907 | -6.11499 | 3.53E-08 | 4.64E-06 | 8.57542 |
| GLUL | -1.67207 | -0.06292 | -6.1136 | 3.55E-08 | 4.64E-06 | 8.569836 |
| ADHFE1 | -2.4934 | -0.10327 | -6.11303 | 3.56E-08 | 4.64E-06 | 8.567524 |
| KLK7 | 3.641014 | 0.597746 | 6.110258 | 3.60E-08 | 4.66E-06 | 8.556344 |
| KIRREL2 | -1.15696 | -0.08658 | -6.10798 | 3.64E-08 | 4.68E-06 | 8.547144 |
| CKM | -4.80344 | 0.846461 | -6.10376 | 3.70E-08 | 4.73E-06 | 8.530143 |
| KCNJ13 | -1.69055 | 0.63492 | -6.10156 | 3.74E-08 | 4.76E-06 | 8.521279 |
| SLC16A7 | -2.2548 | 0.316832 | -6.09758 | 3.80E-08 | 4.82E-06 | 8.505229 |
| XLOC_012829 | -2.14122 | 1.056177 | -6.09632 | 3.82E-08 | 4.83E-06 | 8.500154 |
| CDX1 | 2.574234 | 0.045052 | 6.094428 | 3.85E-08 | 4.85E-06 | 8.492511 |
| S100A2 | 1.918022 | -0.28617 | 6.078052 | 4.13E-08 | 5.10E-06 | 8.426532 |
| MRGPRX3 | -2.34836 | 1.37827 | -6.07773 | 4.13E-08 | 5.10E-06 | 8.425227 |
| CHP2 | 5.815487 | -1.01983 | 6.075761 | 4.17E-08 | 5.11E-06 | 8.417309 |
| LOC100507464 | -5.03736 | 3.006996 | -6.07478 | 4.19E-08 | 5.11E-06 | 8.413353 |
| ASCL2 | 2.219099 | 0.207814 | 6.072223 | 4.23E-08 | 5.12E-06 | 8.403063 |
| GABARAPL1 | -1.33873 | 0.179566 | -6.07125 | 4.25E-08 | 5.12E-06 | 8.399133 |
| PTPRZ1 | -2.22967 | -0.50204 | -6.06984 | 4.28E-08 | 5.14E-06 | 8.393471 |
| CNTNAP2 | 1.851027 | 0.550718 | 6.068454 | 4.30E-08 | 5.14E-06 | 8.387891 |
| XYLT2 | -1.66326 | 0.654207 | -6.06778 | 4.31E-08 | 5.14E-06 | 8.385163 |
| SULT2B1 | 1.827271 | 0.071447 | 6.065361 | 4.36E-08 | 5.15E-06 | 8.375442 |
| BNIP3 | -1.83179 | -0.22349 | -6.06479 | 4.37E-08 | 5.15E-06 | 8.37314 |
| LOC100128131 | -2.95774 | 1.739855 | -6.06363 | 4.39E-08 | 5.16E-06 | 8.368472 |
| NR2F2 | -1.42965 | 0.502012 | -6.06277 | 4.40E-08 | 5.16E-06 | 8.36502 |
| XLOC_001575 | -3.32938 | 1.027738 | -6.06029 | 4.45E-08 | 5.17E-06 | 8.355038 |
| WASF3 | -2.01098 | -0.04769 | -6.05674 | 4.52E-08 | 5.21E-06 | 8.340751 |
| FNDC5 | -3.20208 | 0.692189 | -6.05258 | 4.60E-08 | 5.29E-06 | 8.324035 |
| SERPINA4 | -2.25592 | -0.23366 | -6.04223 | 4.80E-08 | 5.49E-06 | 8.282417 |
| CGNL1 | -2.0652 | 0.392567 | -6.04003 | 4.85E-08 | 5.52E-06 | 8.273557 |
| C10orf81 | 2.03622 | -0.53977 | 6.032907 | 5.00E-08 | 5.59E-06 | 8.244959 |
| DUOX1 | -1.88552 | 0.047898 | -6.02643 | 5.14E-08 | 5.71E-06 | 8.218954 |
| PHLDA2 | 1.452642 | -0.23168 | 6.026109 | 5.14E-08 | 5.71E-06 | 8.217655 |
| TTC7B | -1.75352 | 0.143738 | -6.02507 | 5.17E-08 | 5.71E-06 | 8.21348 |
| XLOC_009183 | -1.63591 | 0.891162 | -6.02454 | 5.18E-08 | 5.71E-06 | 8.211363 |
| FMO6P | -2.9909 | 0.333866 | -6.02251 | 5.22E-08 | 5.74E-06 | 8.20321 |
| SLC41A1 | -1.45492 | 0.112 | -6.02036 | 5.27E-08 | 5.77E-06 | 8.194572 |
| PDILT | -3.25391 | 0.629 | -6.01443 | 5.40E-08 | 5.90E-06 | 8.170766 |
| ELL2 | -1.34528 | 0.344767 | -6.01291 | 5.44E-08 | 5.91E-06 | 8.164678 |
| CACNA2D3 | -1.35819 | 0.03915 | -6.0113 | 5.48E-08 | 5.92E-06 | 8.158223 |
| MAGEH1 | -1.34915 | -0.23391 | -5.99855 | 5.78E-08 | 6.18E-06 | 8.107091 |
| XLOC_000642 | 1.474179 | 0.109483 | 5.994505 | 5.88E-08 | 6.26E-06 | 8.090869 |
| NMU | 2.287909 | -0.26398 | 5.993029 | 5.91E-08 | 6.28E-06 | 8.084953 |
| MARVELD3 | 2.121028 | 0.44425 | 5.991435 | 5.95E-08 | 6.30E-06 | 8.078563 |
| COBLL1 | -1.00421 | 0.299442 | -5.98903 | 6.02E-08 | 6.33E-06 | 8.068936 |
| MYO7B | 3.31135 | 0.225027 | 5.988241 | 6.04E-08 | 6.33E-06 | 8.065766 |
| ZNF462 | 1.128383 | -0.19512 | 5.984447 | 6.13E-08 | 6.41E-06 | 8.050565 |
| POPDC3 | -2.10712 | 0.467925 | -5.97851 | 6.29E-08 | 6.55E-06 | 8.026802 |
| JPH1 | 1.947292 | -0.14195 | 5.977579 | 6.31E-08 | 6.55E-06 | 8.02306 |
| XLOC_002258 | -3.61106 | 1.627117 | -5.97521 | 6.38E-08 | 6.58E-06 | 8.01356 |
| CALML4 | 1.461144 | -0.26856 | 5.974748 | 6.39E-08 | 6.58E-06 | 8.011724 |
| FGG | -4.29968 | 1.929094 | -5.97298 | 6.44E-08 | 6.58E-06 | 8.004654 |
| FUT5 | 1.916582 | -0.24088 | 5.97275 | 6.44E-08 | 6.58E-06 | 8.003725 |
| FABP3 | -2.81413 | 0.537516 | -5.9719 | 6.47E-08 | 6.58E-06 | 8.000308 |
| XLOC_002302 | -2.55653 | 1.835544 | -5.96792 | 6.57E-08 | 6.65E-06 | 7.984408 |
| XLOC_010491 | -1.27684 | 0.535205 | -5.96574 | 6.64E-08 | 6.69E-06 | 7.975686 |
| SSC5D | -1.73977 | 0.845528 | -5.96375 | 6.69E-08 | 6.73E-06 | 7.967715 |
| C21orf30 | -3.0155 | 1.137177 | -5.96124 | 6.76E-08 | 6.77E-06 | 7.957672 |
| KIF17 | -3.06432 | 1.318534 | -5.95795 | 6.86E-08 | 6.79E-06 | 7.944508 |
| EPOR | -1.34549 | 0.127851 | -5.95609 | 6.91E-08 | 6.80E-06 | 7.937083 |
| C13orf15 | -1.48754 | 0.147472 | -5.95494 | 6.94E-08 | 6.81E-06 | 7.932475 |
| EPHB2 | 1.508435 | 0.211928 | 5.952305 | 7.02E-08 | 6.87E-06 | 7.921934 |
| LOC100288092 | 2.36321 | -0.14853 | 5.946809 | 7.19E-08 | 6.99E-06 | 7.899963 |
| CEACAM6 | 3.539734 | -0.85413 | 5.946612 | 7.19E-08 | 6.99E-06 | 7.899176 |
| PPP2R3A | -1.76893 | -0.03625 | -5.94055 | 7.38E-08 | 7.15E-06 | 7.874962 |
| KLK1 | 2.006606 | 0.195511 | 5.938006 | 7.46E-08 | 7.21E-06 | 7.864787 |
| SLC39A5 | 3.770933 | -0.41775 | 5.933792 | 7.59E-08 | 7.27E-06 | 7.847959 |
| CXXC4 | 1.446629 | 0.084024 | 5.933753 | 7.59E-08 | 7.27E-06 | 7.847801 |
| BCL11B | 1.09437 | -0.05127 | 5.930864 | 7.68E-08 | 7.30E-06 | 7.836265 |
| FAM20C | -1.11865 | -0.09477 | -5.93076 | 7.69E-08 | 7.30E-06 | 7.835855 |
| XLOC_l2_015752 | -1.17185 | 0.023896 | -5.92825 | 7.77E-08 | 7.35E-06 | 7.825846 |
| LOC389641 | 1.236104 | -0.26996 | 5.926566 | 7.82E-08 | 7.38E-06 | 7.819105 |
| KLHDC8A | -2.28348 | 0.369782 | -5.92569 | 7.85E-08 | 7.39E-06 | 7.815611 |
| SLC27A2 | 1.691197 | -0.28659 | 5.924762 | 7.88E-08 | 7.39E-06 | 7.811903 |
| C8orf84 | -2.66842 | 0.219323 | -5.92339 | 7.93E-08 | 7.41E-06 | 7.806439 |
| LOC386758 | -1.3134 | 0.030331 | -5.92302 | 7.94E-08 | 7.41E-06 | 7.804966 |
| IYD | 1.349965 | 0.02743 | 5.921154 | 8.00E-08 | 7.41E-06 | 7.797506 |
| KRT14 | 1.641692 | 0.368926 | 5.920834 | 8.02E-08 | 7.41E-06 | 7.796229 |
| XLOC_009021 | 1.26044 | 0.010163 | 5.920801 | 8.02E-08 | 7.41E-06 | 7.796094 |
| CECR7 | -1.15939 | 0.411652 | -5.90761 | 8.47E-08 | 7.74E-06 | 7.743486 |
| IL11RA | -1.33088 | 0.309843 | -5.90245 | 8.66E-08 | 7.80E-06 | 7.722906 |
| XLOC_012662 | -2.17997 | 0.903761 | -5.90156 | 8.69E-08 | 7.81E-06 | 7.71935 |
| PSTPIP1 | -1.03387 | 0.005318 | -5.8969 | 8.86E-08 | 7.94E-06 | 7.700788 |
| BCL2L11 | -1.73872 | 0.719027 | -5.89431 | 8.96E-08 | 7.98E-06 | 7.690472 |
| XLOC_l2_014101 | 1.134891 | -0.10076 | 5.892609 | 9.02E-08 | 8.02E-06 | 7.68369 |
| SEMA4A | -1.01304 | 0.04614 | -5.88813 | 9.19E-08 | 8.09E-06 | 7.665846 |
| ABP1 | 2.480813 | -0.73433 | 5.887912 | 9.20E-08 | 8.09E-06 | 7.664979 |
| DNER | -3.10699 | 2.272407 | -5.88786 | 9.20E-08 | 8.09E-06 | 7.664788 |
| ALDH1A1 | -1.21852 | -0.06168 | -5.88588 | 9.28E-08 | 8.11E-06 | 7.656881 |
| HPN | -2.73818 | -0.36427 | -5.88488 | 9.32E-08 | 8.11E-06 | 7.652918 |
| AGXT2L1 | -4.29048 | 0.808489 | -5.8844 | 9.34E-08 | 8.11E-06 | 7.651012 |
| XLOC_005429 | -2.38367 | 1.200581 | -5.88413 | 9.35E-08 | 8.11E-06 | 7.649906 |
| SLC16A2 | 1.528116 | 0.159192 | 5.881013 | 9.47E-08 | 8.19E-06 | 7.637509 |
| OXCT1 | -1.06808 | 0.230143 | -5.88024 | 9.50E-08 | 8.19E-06 | 7.634453 |
| TTLL6 | 1.4922 | 0.907679 | 5.876299 | 9.66E-08 | 8.31E-06 | 7.618746 |
| SNORA5A | -5.3024 | 2.110917 | -5.87388 | 9.76E-08 | 8.37E-06 | 7.609107 |
| ORM2 | -2.3645 | -0.58458 | -5.87342 | 9.78E-08 | 8.37E-06 | 7.60728 |
| XLOC_002997 | -1.62724 | 0.662634 | -5.86784 | 1.00E-07 | 8.51E-06 | 7.585077 |
| LOC644192 | -1.19278 | 0.169085 | -5.85971 | 1.04E-07 | 8.77E-06 | 7.552751 |
| NEU4 | 2.522553 | 1.375574 | 5.858788 | 1.04E-07 | 8.78E-06 | 7.549097 |
| CDH16 | 2.373052 | 0.731516 | 5.85354 | 1.06E-07 | 8.92E-06 | 7.528239 |
| CD9 | 1.392214 | -0.08143 | 5.852498 | 1.07E-07 | 8.92E-06 | 7.524096 |
| GUCA1C | -2.39763 | 1.803712 | -5.85232 | 1.07E-07 | 8.92E-06 | 7.523392 |
| XLOC_l2_006574 | -1.65316 | 0.127278 | -5.84581 | 1.10E-07 | 9.15E-06 | 7.497538 |
| ABLIM2 | 1.067853 | 0.010002 | 5.842558 | 1.11E-07 | 9.22E-06 | 7.484613 |
| G0S2 | -2.87743 | 0.661201 | -5.84172 | 1.12E-07 | 9.22E-06 | 7.481273 |
| HOXA11 | 3.148607 | 0.805563 | 5.839721 | 1.13E-07 | 9.23E-06 | 7.473345 |
| ITM2A | -1.49582 | 0.015345 | -5.83969 | 1.13E-07 | 9.23E-06 | 7.473213 |
| LOC730236 | -1.29999 | 0.774403 | -5.83931 | 1.13E-07 | 9.23E-06 | 7.47173 |
| AGR3 | 1.294944 | -0.21293 | 5.835688 | 1.15E-07 | 9.33E-06 | 7.457335 |
| MLXIPL | 2.255446 | -0.02778 | 5.835072 | 1.15E-07 | 9.33E-06 | 7.45489 |
| XLOC_004726 | -1.28933 | 0.61953 | -5.83459 | 1.15E-07 | 9.33E-06 | 7.452963 |
| CDX2 | 3.449571 | -0.75649 | 5.834385 | 1.15E-07 | 9.33E-06 | 7.452164 |
| ODZ3 | -2.62539 | 1.011774 | -5.82514 | 1.20E-07 | 9.57E-06 | 7.415476 |
| HOMER2 | -1.69581 | -0.07848 | -5.82075 | 1.22E-07 | 9.73E-06 | 7.398069 |
| PTPRS | -1.31722 | -0.01323 | -5.81802 | 1.23E-07 | 9.81E-06 | 7.387255 |
| XLOC_006948 | 1.727662 | 0.199496 | 5.815051 | 1.25E-07 | 9.89E-06 | 7.37547 |
| KIT | -1.3981 | 0.249966 | -5.81315 | 1.26E-07 | 9.92E-06 | 7.367926 |
| SKP2 | 1.214565 | -0.06558 | 5.809825 | 1.28E-07 | 1.00E-05 | 7.354758 |
| GJB4 | 1.735169 | -0.23156 | 5.804764 | 1.30E-07 | 1.02E-05 | 7.334704 |
| TP73-AS1 | -1.42501 | -0.21565 | -5.80289 | 1.31E-07 | 1.03E-05 | 7.327263 |
| ZC3H12C | -1.26152 | -0.11966 | -5.80012 | 1.33E-07 | 1.04E-05 | 7.316316 |
| SHD | 2.195467 | 0.522193 | 5.799006 | 1.33E-07 | 1.04E-05 | 7.311896 |
| FGB | -2.882 | 1.846853 | -5.79209 | 1.37E-07 | 1.06E-05 | 7.284524 |
| XLOC_012210 | -2.66926 | 1.625255 | -5.78583 | 1.41E-07 | 1.08E-05 | 7.259756 |
| XLOC_l2_005553 | 2.066791 | 0.101265 | 5.779627 | 1.45E-07 | 1.11E-05 | 7.235201 |
| CYFIP2 | -1.36998 | 0.136519 | -5.77952 | 1.45E-07 | 1.11E-05 | 7.234785 |
| FAM81A | 1.769423 | -0.32121 | 5.778498 | 1.45E-07 | 1.11E-05 | 7.230736 |
| ANKRD65 | -1.61189 | -0.29851 | -5.77736 | 1.46E-07 | 1.11E-05 | 7.226225 |
| XLOC_002678 | -1.24295 | 0.76029 | -5.77411 | 1.48E-07 | 1.12E-05 | 7.213372 |
| RBPJL | -1.33396 | 0.611687 | -5.7685 | 1.52E-07 | 1.15E-05 | 7.191219 |
| RIMBP3 | -1.14943 | -0.15711 | -5.75978 | 1.57E-07 | 1.18E-05 | 7.156744 |
| XLOC_008183 | -1.01918 | 0.197439 | -5.75951 | 1.57E-07 | 1.18E-05 | 7.1557 |
| CD79A | -1.26261 | 0.095804 | -5.75937 | 1.57E-07 | 1.18E-05 | 7.155155 |
| NEK11 | 1.105344 | -0.10517 | 5.755917 | 1.60E-07 | 1.19E-05 | 7.141499 |
| FAM84A | 1.79674 | 0.457578 | 5.754512 | 1.61E-07 | 1.20E-05 | 7.135954 |
| MACC1 | 1.278501 | -0.09759 | 5.749849 | 1.64E-07 | 1.22E-05 | 7.117544 |
| PDZK1 | 2.837068 | 0.169937 | 5.748187 | 1.65E-07 | 1.22E-05 | 7.110982 |
| XLOC_013925 | -2.06566 | 1.265453 | -5.74633 | 1.66E-07 | 1.23E-05 | 7.10366 |
| MUC17 | 3.311057 | -1.0157 | 5.744825 | 1.67E-07 | 1.23E-05 | 7.097717 |
| GJB3 | 1.99163 | -0.38139 | 5.741999 | 1.69E-07 | 1.25E-05 | 7.086568 |
| TPPP3 | 1.761114 | 0.162226 | 5.73545 | 1.74E-07 | 1.27E-05 | 7.060733 |
| CADPS | 1.453946 | 0.432445 | 5.730644 | 1.77E-07 | 1.28E-05 | 7.041785 |
| TCEAL2 | -2.18467 | 0.085704 | -5.7304 | 1.78E-07 | 1.28E-05 | 7.040811 |
| MYBPC3 | -1.54425 | 0.499655 | -5.72735 | 1.80E-07 | 1.29E-05 | 7.028786 |
| IRX3 | -1.85679 | -0.18056 | -5.72669 | 1.80E-07 | 1.29E-05 | 7.026218 |
| ARMCX2 | -1.076 | -0.16099 | -5.72153 | 1.84E-07 | 1.31E-05 | 7.005852 |
| XLOC_005894 | -2.80366 | 1.283124 | -5.71569 | 1.89E-07 | 1.33E-05 | 6.982868 |
| ANXA9 | 1.96533 | 0.534578 | 5.71189 | 1.92E-07 | 1.35E-05 | 6.9679 |
| NR1I2 | 2.168834 | -0.58256 | 5.711061 | 1.92E-07 | 1.35E-05 | 6.964638 |
| GALM | 1.010905 | 0.009443 | 5.708929 | 1.94E-07 | 1.36E-05 | 6.956244 |
| LIF | 1.04369 | 0.10416 | 5.705604 | 1.97E-07 | 1.37E-05 | 6.943157 |
| SCUBE2 | -2.24137 | 0.371718 | -5.70557 | 1.97E-07 | 1.37E-05 | 6.943018 |
| XLOC_003780 | -2.61536 | 1.683046 | -5.7034 | 1.99E-07 | 1.38E-05 | 6.934489 |
| TMEM150B | 1.709682 | -0.11199 | 5.703089 | 1.99E-07 | 1.38E-05 | 6.933261 |
| TFAP2C | 1.146876 | 0.154043 | 5.701836 | 2.00E-07 | 1.38E-05 | 6.928331 |
| SALL2 | -1.04126 | 0.058974 | -5.69901 | 2.02E-07 | 1.39E-05 | 6.917204 |
| NDN | -1.1727 | -0.15893 | -5.69768 | 2.03E-07 | 1.39E-05 | 6.911989 |
| PHYHD1 | -1.70597 | -0.39353 | -5.69734 | 2.04E-07 | 1.39E-05 | 6.910635 |
| PACSIN1 | -2.53264 | 0.049344 | -5.69702 | 2.04E-07 | 1.39E-05 | 6.909388 |
| SULT2A1 | -3.69312 | -0.09658 | -5.69691 | 2.04E-07 | 1.39E-05 | 6.908945 |
| ANKS4B | 1.792129 | -0.74224 | 5.696072 | 2.05E-07 | 1.39E-05 | 6.905656 |
| RAB11FIP2 | -1.38123 | 0.522632 | -5.69517 | 2.06E-07 | 1.39E-05 | 6.902092 |
| CDH17 | 3.94696 | 0.022546 | 5.693436 | 2.07E-07 | 1.40E-05 | 6.895293 |
| ENTPD2 | 1.153793 | -0.06763 | 5.693019 | 2.07E-07 | 1.40E-05 | 6.893652 |
| LDOC1 | -1.47621 | -0.32351 | -5.69272 | 2.08E-07 | 1.40E-05 | 6.892486 |
| PDE3B | 1.433988 | -0.16103 | 5.692456 | 2.08E-07 | 1.40E-05 | 6.891438 |
| CXCL17 | -1.77379 | -0.64758 | -5.69004 | 2.10E-07 | 1.41E-05 | 6.881922 |
| KRT17 | 3.073796 | 0.741778 | 5.688927 | 2.11E-07 | 1.41E-05 | 6.877562 |
| XLOC_000095 | 1.461893 | 0.102916 | 5.679911 | 2.19E-07 | 1.46E-05 | 6.842134 |
| XLOC_012005 | -1.91765 | 0.802186 | -5.67786 | 2.21E-07 | 1.47E-05 | 6.834061 |
| HYMAI | -1.88956 | 0.037967 | -5.67648 | 2.22E-07 | 1.47E-05 | 6.828673 |
| ROBO3 | -2.31187 | 0.934329 | -5.67624 | 2.22E-07 | 1.47E-05 | 6.827696 |
| KRT15 | 1.200731 | 0.037609 | 5.675148 | 2.23E-07 | 1.47E-05 | 6.823427 |
| NPY | -1.99389 | -0.28965 | -5.66352 | 2.34E-07 | 1.53E-05 | 6.777785 |
| BEND5 | -1.61816 | -0.13799 | -5.66337 | 2.34E-07 | 1.53E-05 | 6.777189 |
| TNFRSF12A | 1.809899 | 0.063178 | 5.66034 | 2.37E-07 | 1.54E-05 | 6.765302 |
| TRPM2 | 1.602336 | 0.460856 | 5.65704 | 2.41E-07 | 1.56E-05 | 6.75236 |
| LOC100124692 | 1.594435 | 0.860245 | 5.654012 | 2.44E-07 | 1.58E-05 | 6.740484 |
| RGL3 | -1.50869 | 0.023777 | -5.65103 | 2.47E-07 | 1.59E-05 | 6.728784 |
| CXorf61 | 3.531433 | 1.664214 | 5.650807 | 2.47E-07 | 1.59E-05 | 6.727919 |
| STOX2 | -1.11191 | -0.21219 | -5.64721 | 2.51E-07 | 1.60E-05 | 6.71383 |
| CRYM | 1.361495 | -0.11276 | 5.646899 | 2.51E-07 | 1.60E-05 | 6.712597 |
| HES2 | 1.642722 | 0.592508 | 5.645269 | 2.53E-07 | 1.61E-05 | 6.706212 |
| EPCAM | 1.007388 | -0.04591 | 5.643805 | 2.54E-07 | 1.62E-05 | 6.700474 |
| XLOC_007161 | -3.0341 | 1.683218 | -5.6374 | 2.61E-07 | 1.65E-05 | 6.675388 |
| REG4 | 4.193504 | -0.52127 | 5.632828 | 2.66E-07 | 1.68E-05 | 6.657477 |
| NR6A1 | 1.155959 | 0.327026 | 5.627911 | 2.71E-07 | 1.71E-05 | 6.638229 |
| EIF5A2 | -1.6581 | 0.453263 | -5.62475 | 2.75E-07 | 1.73E-05 | 6.625859 |
| XLOC_009894 | -3.0022 | 2.214739 | -5.62452 | 2.75E-07 | 1.73E-05 | 6.624974 |
| APLP1 | -1.79595 | 0.575765 | -5.62079 | 2.80E-07 | 1.74E-05 | 6.610358 |
| XLOC_006757 | -1.29759 | 0.34194 | -5.62065 | 2.80E-07 | 1.74E-05 | 6.60983 |
| ANXA13 | 4.170603 | -0.24064 | 5.620184 | 2.80E-07 | 1.74E-05 | 6.607994 |
| XLOC_l2_010759 | -1.8415 | 0.190056 | -5.62014 | 2.80E-07 | 1.74E-05 | 6.607812 |
| P2RX2 | -2.63298 | 1.55164 | -5.61945 | 2.81E-07 | 1.74E-05 | 6.60511 |
| KLF12 | -1.23292 | 0.075197 | -5.61773 | 2.83E-07 | 1.75E-05 | 6.598401 |
| C20orf194 | -1.07611 | -0.28356 | -5.61531 | 2.86E-07 | 1.76E-05 | 6.58895 |
| XLOC_014356 | -3.4927 | 1.854322 | -5.61424 | 2.87E-07 | 1.77E-05 | 6.584734 |
| SPIRE1 | -1.10469 | -0.19124 | -5.6087 | 2.94E-07 | 1.81E-05 | 6.563083 |
| MGC12982 | 1.267134 | -0.20858 | 5.605037 | 2.98E-07 | 1.83E-05 | 6.548775 |
| CCL15 | 2.380211 | -0.10977 | 5.603677 | 3.00E-07 | 1.83E-05 | 6.543461 |
| XLOC_008152 | -1.19672 | -0.28203 | -5.60316 | 3.01E-07 | 1.83E-05 | 6.541431 |
| ENOX1 | -1.72779 | 0.387226 | -5.60207 | 3.02E-07 | 1.83E-05 | 6.537194 |
| TEX11 | 1.807688 | 0.071116 | 5.59825 | 3.07E-07 | 1.85E-05 | 6.522261 |
| GLIPR2 | -1.05715 | -0.18444 | -5.59391 | 3.12E-07 | 1.88E-05 | 6.505326 |
| MSLN | 3.231674 | 0.248237 | 5.592024 | 3.15E-07 | 1.89E-05 | 6.497949 |
| EDN3 | 2.0987 | -0.61438 | 5.583687 | 3.26E-07 | 1.94E-05 | 6.465413 |
| FGA | -2.58178 | 1.360527 | -5.58154 | 3.29E-07 | 1.95E-05 | 6.457031 |
| OR52K2 | -2.53358 | 0.967342 | -5.58127 | 3.29E-07 | 1.95E-05 | 6.455972 |
| OTC | 3.325951 | -0.39171 | 5.579933 | 3.31E-07 | 1.95E-05 | 6.450767 |
| RAP1GAP | -1.00901 | 0.435565 | -5.57574 | 3.37E-07 | 1.98E-05 | 6.434424 |
| MAST4 | -1.03048 | 0.19462 | -5.57544 | 3.37E-07 | 1.98E-05 | 6.43326 |
| JMJD5 | -1.13615 | 0.30187 | -5.57225 | 3.41E-07 | 2.00E-05 | 6.420828 |
| EEF1A2 | -2.71515 | -0.58038 | -5.56754 | 3.48E-07 | 2.03E-05 | 6.402452 |
| DRD2 | 1.403379 | 0.784986 | 5.565392 | 3.51E-07 | 2.04E-05 | 6.394083 |
| SLC25A4 | -1.27768 | 0.426496 | -5.5652 | 3.51E-07 | 2.04E-05 | 6.393352 |
| CPS1 | 4.713907 | -0.61632 | 5.563324 | 3.54E-07 | 2.05E-05 | 6.386026 |
| AIFM3 | 1.36556 | 0.048528 | 5.563164 | 3.54E-07 | 2.05E-05 | 6.385405 |
| ADH4 | 4.377629 | 0.660873 | 5.55992 | 3.59E-07 | 2.07E-05 | 6.372768 |
| TMEM45B | 1.185871 | 0.049751 | 5.558339 | 3.62E-07 | 2.08E-05 | 6.366611 |
| S100A6 | 1.095363 | -0.24504 | 5.558005 | 3.62E-07 | 2.08E-05 | 6.365312 |
| GPA33 | 3.457242 | -0.57075 | 5.557575 | 3.63E-07 | 2.08E-05 | 6.363635 |
| KRT73 | -1.30268 | 0.466078 | -5.55382 | 3.68E-07 | 2.11E-05 | 6.349021 |
| LOC100505839 | 2.294159 | -0.36612 | 5.549975 | 3.74E-07 | 2.13E-05 | 6.334053 |
| SLC26A9 | -3.09754 | -0.85254 | -5.54531 | 3.81E-07 | 2.17E-05 | 6.315905 |
| XLOC_012786 | -1.83127 | -0.03574 | -5.54105 | 3.88E-07 | 2.19E-05 | 6.299341 |
| PDE1B | -2.31392 | 0.850395 | -5.54073 | 3.89E-07 | 2.19E-05 | 6.298074 |
| C10orf11 | -1.36452 | -0.11156 | -5.54044 | 3.89E-07 | 2.19E-05 | 6.296951 |
| AGMAT | 1.979384 | -0.75955 | 5.537805 | 3.93E-07 | 2.21E-05 | 6.286712 |
| CAPN13 | -1.09543 | -0.35192 | -5.53405 | 3.99E-07 | 2.24E-05 | 6.272132 |
| SLC22A18AS | 1.581694 | 0.006311 | 5.528406 | 4.09E-07 | 2.28E-05 | 6.250181 |
| C3orf18 | -1.56509 | 0.4256 | -5.52627 | 4.12E-07 | 2.29E-05 | 6.241875 |
| METTL7A | -1.05533 | -0.00895 | -5.5261 | 4.13E-07 | 2.29E-05 | 6.241226 |
| CLDN15 | 2.554093 | 0.697148 | 5.520971 | 4.21E-07 | 2.33E-05 | 6.221299 |
| LOC439990 | 1.404891 | -0.18526 | 5.520037 | 4.23E-07 | 2.34E-05 | 6.217673 |
| RARRES1 | 2.565887 | -0.04367 | 5.51837 | 4.26E-07 | 2.35E-05 | 6.211202 |
| EFS | -1.19216 | -0.13737 | -5.51767 | 4.27E-07 | 2.35E-05 | 6.208501 |
| CHODL | -1.52226 | 0.140983 | -5.51267 | 4.36E-07 | 2.38E-05 | 6.189074 |
| TLX1 | 1.245307 | 0.792011 | 5.512316 | 4.37E-07 | 2.38E-05 | 6.187703 |
| ABCA6 | -1.11427 | -0.28434 | -5.50872 | 4.43E-07 | 2.41E-05 | 6.17376 |
| MICAL3 | 1.027903 | -0.05839 | 5.507344 | 4.46E-07 | 2.42E-05 | 6.16841 |
| GPT2 | -1.19295 | 0.021898 | -5.50067 | 4.58E-07 | 2.48E-05 | 6.142526 |
| LOC401127 | 1.176873 | 0.025923 | 5.499742 | 4.60E-07 | 2.48E-05 | 6.13893 |
| SLC6A16 | -1.02259 | 0.166958 | -5.49949 | 4.60E-07 | 2.48E-05 | 6.137952 |
| CDH3 | 2.672082 | 0.220092 | 5.497968 | 4.63E-07 | 2.49E-05 | 6.132052 |
| BVES | -1.85881 | 0.557174 | -5.49691 | 4.65E-07 | 2.49E-05 | 6.127953 |
| ORM1 | -2.20386 | -0.3546 | -5.49644 | 4.66E-07 | 2.50E-05 | 6.126125 |
| LOC100506983 | -1.73282 | 0.863593 | -5.49489 | 4.69E-07 | 2.50E-05 | 6.120138 |
| RPRM | -2.35204 | 1.764236 | -5.49398 | 4.71E-07 | 2.50E-05 | 6.116611 |
| ERC2 | -1.1146 | 0.40463 | -5.49389 | 4.71E-07 | 2.50E-05 | 6.116265 |
| AR | -1.54994 | 0.453546 | -5.49239 | 4.74E-07 | 2.52E-05 | 6.110455 |
| LINGO4 | -1.57427 | 0.270687 | -5.48814 | 4.82E-07 | 2.56E-05 | 6.093984 |
| COL2A1 | -3.40943 | 1.141158 | -5.47782 | 5.03E-07 | 2.65E-05 | 6.054034 |
| LDLRAD1 | 1.37266 | 0.017575 | 5.477041 | 5.04E-07 | 2.65E-05 | 6.050999 |
| KCNH8 | 1.165395 | -0.06278 | 5.474283 | 5.10E-07 | 2.67E-05 | 6.040327 |
| GHR | -1.55804 | 0.602062 | -5.47116 | 5.17E-07 | 2.69E-05 | 6.028228 |
| ARHGAP24 | -1.15683 | -0.04715 | -5.47036 | 5.18E-07 | 2.69E-05 | 6.025152 |
| FAM110B | -1.42184 | -0.15873 | -5.46819 | 5.23E-07 | 2.71E-05 | 6.016773 |
| C14orf37 | -1.79822 | 0.451167 | -5.46559 | 5.28E-07 | 2.73E-05 | 6.006696 |
| PPP1R3C | -1.22958 | 0.027531 | -5.46518 | 5.29E-07 | 2.73E-05 | 6.005107 |
| PLA1A | -1.44359 | -0.18526 | -5.46221 | 5.36E-07 | 2.74E-05 | 5.99364 |
| XLOC_l2_010461 | -2.38507 | -0.08372 | -5.45828 | 5.44E-07 | 2.77E-05 | 5.978443 |
| ZNF512B | -1.0761 | -0.32707 | -5.45776 | 5.46E-07 | 2.77E-05 | 5.976419 |
| GPR98 | -1.67563 | -0.16061 | -5.45748 | 5.46E-07 | 2.77E-05 | 5.975345 |
| XLOC_012170 | 1.092257 | 0.047475 | 5.457292 | 5.47E-07 | 2.77E-05 | 5.97463 |
| DUSP10 | 1.008031 | -0.05706 | 5.456061 | 5.49E-07 | 2.78E-05 | 5.969874 |
| LOC100128501 | -2.29295 | 1.575249 | -5.45179 | 5.59E-07 | 2.82E-05 | 5.953363 |
| FAM165B | -1.076 | 0.41596 | -5.45014 | 5.63E-07 | 2.83E-05 | 5.946992 |
| LOC100130093 | -1.11953 | 0.021546 | -5.44816 | 5.67E-07 | 2.85E-05 | 5.939352 |
| ITIH4 | -1.10014 | -0.01183 | -5.44556 | 5.73E-07 | 2.87E-05 | 5.92931 |
| LINC00317 | -1.8554 | 0.422687 | -5.44481 | 5.75E-07 | 2.87E-05 | 5.926425 |
| SUN5 | -1.53256 | 0.758264 | -5.44437 | 5.76E-07 | 2.87E-05 | 5.924726 |
| FAM105A | 1.028347 | 0.143627 | 5.440691 | 5.85E-07 | 2.90E-05 | 5.910526 |
| XLOC_002094 | -1.93257 | 0.687956 | -5.43784 | 5.92E-07 | 2.93E-05 | 5.899525 |
| MSX2P1 | 1.056269 | -0.21641 | 5.432214 | 6.06E-07 | 2.98E-05 | 5.877823 |
| DUOXA1 | -1.47194 | -0.04259 | -5.4317 | 6.07E-07 | 2.98E-05 | 5.875849 |
| XLOC_013965 | -1.04395 | 0.179383 | -5.43079 | 6.09E-07 | 2.99E-05 | 5.872347 |
| WDR86 | -2.43033 | 0.383378 | -5.42894 | 6.14E-07 | 3.01E-05 | 5.865207 |
| AIM1L | 1.548784 | -0.24882 | 5.426592 | 6.20E-07 | 3.03E-05 | 5.856149 |
| OVOL1 | 1.975948 | 0.382918 | 5.425604 | 6.22E-07 | 3.03E-05 | 5.852338 |
| CBS | -1.76094 | -0.05953 | -5.42243 | 6.30E-07 | 3.06E-05 | 5.84009 |
| CNDP1 | 2.887881 | 0.526536 | 5.420168 | 6.36E-07 | 3.08E-05 | 5.83139 |
| XLOC_002867 | 1.277186 | 0.007895 | 5.419182 | 6.38E-07 | 3.09E-05 | 5.827592 |
| FLJ45248 | 1.206327 | 0.012746 | 5.412225 | 6.57E-07 | 3.16E-05 | 5.8008 |
| XLOC_011755 | 1.087399 | 0.106922 | 5.410662 | 6.61E-07 | 3.17E-05 | 5.794784 |
| DHFR | 1.14229 | -0.40233 | 5.405489 | 6.75E-07 | 3.23E-05 | 5.77487 |
| LOC100505535 | 1.697881 | -0.37953 | 5.405136 | 6.76E-07 | 3.23E-05 | 5.773515 |
| VEGFB | -1.25986 | 0.122634 | -5.40495 | 6.76E-07 | 3.23E-05 | 5.772817 |
| SLC6A7 | 2.115091 | 0.728646 | 5.402532 | 6.83E-07 | 3.26E-05 | 5.763494 |
| LOC654342 | -2.06909 | 0.63764 | -5.40092 | 6.88E-07 | 3.27E-05 | 5.757295 |
| RNLS | -1.83641 | 0.220994 | -5.39929 | 6.92E-07 | 3.28E-05 | 5.751034 |
| XLOC_005540 | 1.399732 | 0.739816 | 5.39903 | 6.93E-07 | 3.28E-05 | 5.750022 |
| HSD17B11 | 1.289795 | -0.40069 | 5.395711 | 7.02E-07 | 3.32E-05 | 5.737261 |
| PLCXD3 | -2.17356 | 0.486516 | -5.39446 | 7.06E-07 | 3.33E-05 | 5.732466 |
| CLDN4 | 1.524641 | -0.16962 | 5.394042 | 7.07E-07 | 3.33E-05 | 5.730842 |
| LDHB | -1.45003 | 0.036873 | -5.39402 | 7.07E-07 | 3.33E-05 | 5.730753 |
| COL4A4 | -1.56311 | 0.041062 | -5.39077 | 7.17E-07 | 3.37E-05 | 5.71828 |
| C19orf21 | 1.554554 | -0.42742 | 5.388498 | 7.23E-07 | 3.38E-05 | 5.709535 |
| ACAA2 | 1.05111 | -0.00451 | 5.388149 | 7.24E-07 | 3.38E-05 | 5.708193 |
| LEFTY1 | 3.775456 | 0.620863 | 5.379032 | 7.52E-07 | 3.48E-05 | 5.673172 |
| KRT7 | 1.434996 | 0.544169 | 5.377555 | 7.56E-07 | 3.49E-05 | 5.667502 |
| C8orf78 | -1.34679 | 0.587029 | -5.37587 | 7.61E-07 | 3.51E-05 | 5.661025 |
| TNNC2 | 1.891217 | 0.401201 | 5.369092 | 7.82E-07 | 3.60E-05 | 5.635019 |
| PAIP2B | -1.26541 | 0.329036 | -5.36758 | 7.87E-07 | 3.61E-05 | 5.629226 |
| IGFBP2 | -1.0194 | 0.029045 | -5.36715 | 7.89E-07 | 3.61E-05 | 5.627579 |
| THOC6 | -2.95869 | 1.645314 | -5.36655 | 7.91E-07 | 3.61E-05 | 5.625268 |
| THSD4 | -1.56361 | 0.754585 | -5.3661 | 7.92E-07 | 3.61E-05 | 5.623545 |
| SLC9A9 | -1.02669 | -0.18984 | -5.36601 | 7.92E-07 | 3.61E-05 | 5.623195 |
| CITED1 | 1.512229 | 0.241574 | 5.364491 | 7.97E-07 | 3.62E-05 | 5.617373 |
| TBC1D10C | -1.02201 | 0.016828 | -5.36384 | 7.99E-07 | 3.62E-05 | 5.61489 |
| B3GNT8 | 1.349785 | 0.221095 | 5.362551 | 8.03E-07 | 3.63E-05 | 5.609934 |
| XLOC_001982 | -1.73733 | 1.181046 | -5.36251 | 8.04E-07 | 3.63E-05 | 5.609764 |
| NYX | -1.10159 | 0.011969 | -5.36162 | 8.07E-07 | 3.63E-05 | 5.606349 |
| LOC400464 | -1.18551 | 0.409901 | -5.36122 | 8.08E-07 | 3.63E-05 | 5.604847 |
| PCSK5 | 1.689035 | 0.03397 | 5.359175 | 8.15E-07 | 3.66E-05 | 5.596987 |
| C8orf46 | -1.16164 | 0.529387 | -5.35778 | 8.19E-07 | 3.67E-05 | 5.591649 |
| INA | -1.27861 | 0.119695 | -5.35551 | 8.27E-07 | 3.70E-05 | 5.582943 |
| ANGPTL3 | -1.62589 | 1.103096 | -5.35204 | 8.38E-07 | 3.74E-05 | 5.569647 |
| BTBD17 | 1.775968 | 0.025829 | 5.350587 | 8.43E-07 | 3.74E-05 | 5.56408 |
| FSD2 | -2.01564 | 1.389647 | -5.34434 | 8.65E-07 | 3.82E-05 | 5.540163 |
| VIL1 | 3.208814 | -1.48048 | 5.344266 | 8.65E-07 | 3.82E-05 | 5.539871 |
| CRYBA2 | 1.390451 | 0.041836 | 5.344061 | 8.66E-07 | 3.82E-05 | 5.539084 |
| TMPRSS4 | 1.14976 | -0.33546 | 5.343117 | 8.69E-07 | 3.82E-05 | 5.535473 |
| CHRNE | -1.64172 | 0.244936 | -5.34305 | 8.69E-07 | 3.82E-05 | 5.535226 |
| PDCD6 | -1.17392 | 0.547519 | -5.34267 | 8.71E-07 | 3.82E-05 | 5.533764 |
| ADH6 | 1.697817 | 0.501322 | 5.33447 | 9.00E-07 | 3.91E-05 | 5.502381 |
| XLOC_l2_006789 | -1.15327 | 0.703228 | -5.33192 | 9.09E-07 | 3.94E-05 | 5.492632 |
| LRRC6 | 1.244759 | 0.054161 | 5.330565 | 9.14E-07 | 3.95E-05 | 5.487442 |
| CEACAM3 | 2.146497 | -0.35286 | 5.330072 | 9.16E-07 | 3.95E-05 | 5.485557 |
| CIB2 | 1.518097 | -0.04919 | 5.327145 | 9.27E-07 | 3.99E-05 | 5.474365 |
| HMGB3 | 1.001557 | -0.05452 | 5.32375 | 9.40E-07 | 4.04E-05 | 5.461387 |
| HOXB5 | 1.690876 | 0.134317 | 5.322614 | 9.44E-07 | 4.05E-05 | 5.457047 |
| EPHA1 | 1.116263 | -0.14975 | 5.318674 | 9.59E-07 | 4.11E-05 | 5.441992 |
| XLOC_002921 | 1.253182 | -0.0878 | 5.318433 | 9.60E-07 | 4.11E-05 | 5.44107 |
| LOC100506110 | 1.012352 | -0.09271 | 5.315803 | 9.71E-07 | 4.15E-05 | 5.431026 |
| ASXL3 | -1.72534 | 0.697038 | -5.31468 | 9.75E-07 | 4.16E-05 | 5.426753 |
| CTSF | -1.42091 | -0.04158 | -5.31381 | 9.78E-07 | 4.16E-05 | 5.423395 |
| WTIP | -1.00792 | -0.08869 | -5.31168 | 9.87E-07 | 4.19E-05 | 5.415272 |
| HOXA9 | 1.365729 | 0.400168 | 5.310844 | 9.90E-07 | 4.20E-05 | 5.412087 |
| CLDN7 | 2.240375 | -0.31711 | 5.309631 | 9.95E-07 | 4.21E-05 | 5.407458 |
| C11orf92 | -1.34013 | -0.15059 | -5.30862 | 9.99E-07 | 4.22E-05 | 5.403595 |
| FBXL13 | -2.05946 | -0.18769 | -5.30489 | 1.01E-06 | 4.25E-05 | 5.389345 |
| FUT1 | -1.5947 | -0.31988 | -5.30152 | 1.03E-06 | 4.30E-05 | 5.376497 |
| ABHD11-AS1 | 1.807519 | -0.33883 | 5.30148 | 1.03E-06 | 4.30E-05 | 5.37635 |
| XLOC_003695 | -1.881 | 0.896796 | -5.2984 | 1.04E-06 | 4.33E-05 | 5.364617 |
| XLOC_001728 | 1.205849 | -0.37145 | 5.297681 | 1.04E-06 | 4.34E-05 | 5.361862 |
| STK31 | 1.59238 | -0.03629 | 5.296372 | 1.05E-06 | 4.35E-05 | 5.35687 |
| XLOC_l2_004168 | -1.4551 | 0.447242 | -5.29609 | 1.05E-06 | 4.35E-05 | 5.355801 |
| SLC5A2 | -1.24179 | 0.523924 | -5.29535 | 1.05E-06 | 4.36E-05 | 5.35296 |
| LIPF | -4.06974 | -0.09087 | -5.29344 | 1.06E-06 | 4.39E-05 | 5.345688 |
| XLOC_014103 | 1.914871 | -0.59214 | 5.290397 | 1.08E-06 | 4.42E-05 | 5.334091 |
| KCNK2 | -3.2948 | 2.231964 | -5.28882 | 1.08E-06 | 4.44E-05 | 5.328088 |
| NPR3 | -1.08738 | 0.604224 | -5.28173 | 1.11E-06 | 4.56E-05 | 5.301081 |
| SMAD5 | -1.12491 | 0.20585 | -5.28119 | 1.12E-06 | 4.56E-05 | 5.298998 |
| CLDN1 | 2.501841 | 0.257897 | 5.279994 | 1.12E-06 | 4.58E-05 | 5.29446 |
| ETHE1 | 1.195949 | 0.462826 | 5.274271 | 1.15E-06 | 4.66E-05 | 5.272674 |
| APOC4 | -1.36768 | 0.54864 | -5.27203 | 1.16E-06 | 4.70E-05 | 5.264139 |
| TMEM121 | -1.03634 | -0.25616 | -5.27011 | 1.17E-06 | 4.73E-05 | 5.256843 |
| SMAD9 | -1.08903 | -0.07328 | -5.26781 | 1.18E-06 | 4.76E-05 | 5.248081 |
| THBS2 | 1.476917 | 0.554696 | 5.264694 | 1.19E-06 | 4.81E-05 | 5.23624 |
| XLOC_011849 | -1.90379 | 0.805179 | -5.26187 | 1.21E-06 | 4.85E-05 | 5.225504 |
| LMTK3 | 1.099774 | 0.053935 | 5.259825 | 1.22E-06 | 4.88E-05 | 5.217726 |
| LOC283731 | -1.23229 | 0.55281 | -5.25857 | 1.22E-06 | 4.89E-05 | 5.212972 |
| MRAS | -1.0914 | 0.007762 | -5.25518 | 1.24E-06 | 4.94E-05 | 5.200077 |
| VIPR1 | 1.113208 | 0.109077 | 5.253571 | 1.25E-06 | 4.96E-05 | 5.193961 |
| TRIM15 | 2.087372 | -0.69763 | 5.253344 | 1.25E-06 | 4.96E-05 | 5.193101 |
| NOXO1 | 1.458277 | -0.01472 | 5.248561 | 1.27E-06 | 5.03E-05 | 5.174935 |
| C10orf125 | 1.336985 | 0.310851 | 5.245243 | 1.29E-06 | 5.07E-05 | 5.162337 |
| C2orf89 | 2.049978 | 0.366171 | 5.242717 | 1.30E-06 | 5.11E-05 | 5.152747 |
| MLK7-AS1 | 2.096503 | 1.013198 | 5.241648 | 1.31E-06 | 5.12E-05 | 5.148689 |
| XLOC_010769 | -2.12297 | 0.585323 | -5.23759 | 1.33E-06 | 5.19E-05 | 5.133303 |
| SNORD116-19 | -1.36137 | -0.07966 | -5.23653 | 1.34E-06 | 5.20E-05 | 5.129254 |
| LMAN1L | -1.63287 | 0.658201 | -5.23389 | 1.35E-06 | 5.25E-05 | 5.119274 |
| XLOC_000682 | -1.20319 | 0.184852 | -5.23041 | 1.37E-06 | 5.31E-05 | 5.10607 |
| XLOC_l2_003401 | -1.88954 | 0.341623 | -5.22949 | 1.37E-06 | 5.33E-05 | 5.102576 |
| LARP6 | -1.75117 | -0.07329 | -5.22659 | 1.39E-06 | 5.38E-05 | 5.091589 |
| LOC100507656 | -2.32089 | 0.988917 | -5.22634 | 1.39E-06 | 5.38E-05 | 5.090646 |
| AKNAD1 | -1.03111 | 0.53538 | -5.22339 | 1.41E-06 | 5.43E-05 | 5.079458 |
| IPO11 | -1.3664 | 0.68124 | -5.22056 | 1.42E-06 | 5.48E-05 | 5.068716 |
| MYEF2 | -1.22597 | -0.41759 | -5.21354 | 1.46E-06 | 5.61E-05 | 5.04217 |
| SATB2 | 1.380404 | 0.19946 | 5.212366 | 1.47E-06 | 5.63E-05 | 5.037709 |
| ANXA1 | 1.67506 | 0.158967 | 5.210964 | 1.48E-06 | 5.63E-05 | 5.032402 |
| GREM2 | -2.92989 | -0.66463 | -5.21096 | 1.48E-06 | 5.63E-05 | 5.032379 |
| METTL7B | 1.461323 | -0.07175 | 5.206712 | 1.50E-06 | 5.72E-05 | 5.016312 |
| COLEC12 | -1.45288 | -0.21238 | -5.2062 | 1.51E-06 | 5.72E-05 | 5.014387 |
| UPP1 | 2.019608 | -0.46756 | 5.203047 | 1.53E-06 | 5.78E-05 | 5.00245 |
| TNFRSF11A | 1.866804 | -0.565 | 5.2021 | 1.53E-06 | 5.80E-05 | 4.998867 |
| GLYATL1 | -1.0724 | 0.131466 | -5.20109 | 1.54E-06 | 5.81E-05 | 4.995049 |
| LOC100192426 | -1.24497 | 0.538437 | -5.19733 | 1.56E-06 | 5.87E-05 | 4.980831 |
| LEPR | -1.16477 | 0.390851 | -5.1955 | 1.57E-06 | 5.90E-05 | 4.973907 |
| A1CF | 2.212161 | 0.111932 | 5.19469 | 1.58E-06 | 5.90E-05 | 4.970855 |
| PPP1R14C | 1.941503 | -0.23615 | 5.194024 | 1.58E-06 | 5.91E-05 | 4.968335 |
| BASP1 | -1.30807 | -0.19714 | -5.19389 | 1.58E-06 | 5.91E-05 | 4.967843 |
| WNT11 | 1.500449 | 0.808759 | 5.184742 | 1.64E-06 | 6.08E-05 | 4.933277 |
| C20orf118 | 2.663323 | -0.65295 | 5.183558 | 1.65E-06 | 6.09E-05 | 4.928805 |
| XLOC_007374 | -1.77555 | 0.579062 | -5.18177 | 1.66E-06 | 6.12E-05 | 4.922047 |
| HOXB9 | 3.02569 | 0.001222 | 5.181118 | 1.67E-06 | 6.12E-05 | 4.919594 |
| CLCN2 | 1.181635 | 0.161297 | 5.180888 | 1.67E-06 | 6.12E-05 | 4.918727 |
| MFSD2A | 2.23501 | -0.63468 | 5.17621 | 1.70E-06 | 6.22E-05 | 4.901073 |
| TSPYL5 | -1.23115 | -0.21304 | -5.16786 | 1.76E-06 | 6.39E-05 | 4.869572 |
| RDH12 | -2.21556 | -0.08591 | -5.16396 | 1.78E-06 | 6.47E-05 | 4.854883 |
| XLOC_l2_010149 | 1.548255 | -0.16907 | 5.159021 | 1.82E-06 | 6.58E-05 | 4.836273 |
| XLOC_011754 | -1.9379 | 1.00975 | -5.15052 | 1.88E-06 | 6.76E-05 | 4.804272 |
| LOC284244 | -1.44389 | 0.165266 | -5.15028 | 1.88E-06 | 6.76E-05 | 4.80336 |
| GPRC5A | 1.56999 | -0.51712 | 5.149128 | 1.89E-06 | 6.79E-05 | 4.799028 |
| CRIP1 | 1.075789 | -0.00329 | 5.1487 | 1.90E-06 | 6.79E-05 | 4.797415 |
| XLOC_l2_015894 | -1.04547 | 0.445599 | -5.14763 | 1.90E-06 | 6.81E-05 | 4.793379 |
| GLIS2 | -1.11809 | 0.073389 | -5.1437 | 1.93E-06 | 6.90E-05 | 4.778599 |
| CKB | -2.44431 | 1.034767 | -5.14295 | 1.94E-06 | 6.90E-05 | 4.775772 |
| CLEC12B | -1.06267 | 0.573492 | -5.14161 | 1.95E-06 | 6.92E-05 | 4.770747 |
| PELI2 | -1.13345 | -0.32208 | -5.14054 | 1.96E-06 | 6.93E-05 | 4.76672 |
| XLOC_003483 | -2.05435 | 0.965119 | -5.13951 | 1.97E-06 | 6.93E-05 | 4.762845 |
| CFTR | 1.941289 | -0.04008 | 5.136561 | 1.99E-06 | 6.97E-05 | 4.751758 |
| PVRL1 | 1.11275 | -0.12108 | 5.136523 | 1.99E-06 | 6.97E-05 | 4.751615 |
| ETV4 | 1.525835 | 0.278718 | 5.135235 | 2.00E-06 | 7.00E-05 | 4.746775 |
| FUT3 | 1.4176 | -0.64623 | 5.126826 | 2.07E-06 | 7.19E-05 | 4.715184 |
| XLOC_001952 | -1.59244 | 0.396625 | -5.12629 | 2.07E-06 | 7.19E-05 | 4.713155 |
| PDGFD | -1.52774 | 0.29541 | -5.12601 | 2.08E-06 | 7.19E-05 | 4.712131 |
| KCNIP3 | -2.02874 | -0.2666 | -5.12591 | 2.08E-06 | 7.19E-05 | 4.711739 |
| XLOC_013005 | -1.9454 | 1.333611 | -5.12553 | 2.08E-06 | 7.19E-05 | 4.710305 |
| LOC388588 | -1.49013 | -0.13888 | -5.1243 | 2.09E-06 | 7.22E-05 | 4.705711 |
| CD55 | 1.850658 | -0.14102 | 5.123732 | 2.09E-06 | 7.22E-05 | 4.703564 |
| XLOC_012240 | -2.46035 | 0.97406 | -5.12271 | 2.10E-06 | 7.25E-05 | 4.699721 |
| XLOC_009144 | -2.14421 | 0.693721 | -5.11919 | 2.13E-06 | 7.32E-05 | 4.686516 |
| ANKRD36BP2 | -1.09419 | 0.175752 | -5.11753 | 2.15E-06 | 7.35E-05 | 4.680286 |
| NUP62CL | 1.278615 | -0.45995 | 5.117347 | 2.15E-06 | 7.35E-05 | 4.679599 |
| XLOC_003123 | -1.94365 | 0.462248 | -5.11712 | 2.15E-06 | 7.35E-05 | 4.678756 |
| ADORA2B | 2.519424 | -0.6457 | 5.113262 | 2.18E-06 | 7.42E-05 | 4.664273 |
| XLOC_004446 | -1.94854 | 0.966849 | -5.11315 | 2.18E-06 | 7.42E-05 | 4.663844 |
| XLOC_001589 | -1.25551 | 0.526268 | -5.11099 | 2.20E-06 | 7.46E-05 | 4.655754 |
| AFAP1L2 | 1.249696 | -0.22095 | 5.110566 | 2.21E-06 | 7.46E-05 | 4.654162 |
| NOS2 | 2.965988 | 1.140917 | 5.110157 | 2.21E-06 | 7.46E-05 | 4.65263 |
| FGFBP1 | 2.749457 | -0.58818 | 5.109059 | 2.22E-06 | 7.47E-05 | 4.648513 |
| NANOS3 | 2.182723 | 0.173527 | 5.108932 | 2.22E-06 | 7.47E-05 | 4.648038 |
| TBX3 | 1.312213 | -0.06623 | 5.108587 | 2.22E-06 | 7.47E-05 | 4.646743 |
| SLC26A7 | -2.08461 | 0.536126 | -5.10834 | 2.23E-06 | 7.47E-05 | 4.645832 |
| GALNT14 | -2.15288 | -0.27279 | -5.10802 | 2.23E-06 | 7.47E-05 | 4.6446 |
| CECR1 | -1.21027 | 0.146948 | -5.10669 | 2.24E-06 | 7.50E-05 | 4.63965 |
| SERPINI1 | -1.6358 | -0.00457 | -5.10247 | 2.28E-06 | 7.62E-05 | 4.62382 |
| TNFSF9 | 1.920414 | 0.484627 | 5.101954 | 2.28E-06 | 7.62E-05 | 4.621883 |
| XLOC_005851 | -1.52297 | 0.848225 | -5.09652 | 2.33E-06 | 7.75E-05 | 4.601515 |
| XLOC_000247 | -2.11145 | 0.86685 | -5.09073 | 2.39E-06 | 7.90E-05 | 4.579845 |
| EPPK1 | 1.303227 | 0.03542 | 5.087173 | 2.42E-06 | 7.98E-05 | 4.56654 |
| XLOC_011858 | 1.593539 | -0.01245 | 5.083615 | 2.46E-06 | 8.08E-05 | 4.55323 |
| CA9 | -1.4322 | -0.14302 | -5.08087 | 2.48E-06 | 8.14E-05 | 4.542958 |
| CCND2 | 1.859198 | 0.601844 | 5.080301 | 2.49E-06 | 8.15E-05 | 4.540838 |
| LOC100289134 | -1.64113 | 0.425938 | -5.07933 | 2.50E-06 | 8.17E-05 | 4.537223 |
| SLC4A2 | -1.09273 | 0.19946 | -5.07931 | 2.50E-06 | 8.17E-05 | 4.537145 |
| ZSCAN22 | -1.29332 | 0.541112 | -5.07785 | 2.51E-06 | 8.21E-05 | 4.531674 |
| XLOC_010500 | -1.04802 | 0.957781 | -5.07661 | 2.52E-06 | 8.22E-05 | 4.527057 |
| HIST3H2A | 1.578049 | -0.06301 | 5.069897 | 2.59E-06 | 8.40E-05 | 4.501958 |
| XLOC_000348 | 1.329039 | 0.031962 | 5.068831 | 2.60E-06 | 8.43E-05 | 4.497977 |
| TPPP | 1.4272 | -0.05493 | 5.064583 | 2.65E-06 | 8.55E-05 | 4.482112 |
| GJB5 | 2.347664 | 0.889829 | 5.063063 | 2.66E-06 | 8.60E-05 | 4.476438 |
| SIPA1L2 | 1.367308 | -0.07568 | 5.058684 | 2.71E-06 | 8.70E-05 | 4.460097 |
| LYPD2 | -2.27142 | 0.18126 | -5.05691 | 2.73E-06 | 8.74E-05 | 4.453459 |
| LOC642648 | -1.66282 | 0.275747 | -5.05507 | 2.75E-06 | 8.78E-05 | 4.446624 |
| ASS1 | 1.159996 | 0.296088 | 5.054419 | 2.76E-06 | 8.80E-05 | 4.444186 |
| GSTT2 | -1.56357 | 0.015174 | -5.05421 | 2.76E-06 | 8.80E-05 | 4.443393 |
| LOC100506090 | -1.72913 | 0.633238 | -5.05108 | 2.79E-06 | 8.89E-05 | 4.431723 |
| KCNJ3 | 2.147038 | 0.012579 | 5.048516 | 2.82E-06 | 8.97E-05 | 4.422176 |
| CYP7B1 | -1.34314 | 0.077338 | -5.04821 | 2.82E-06 | 8.97E-05 | 4.421038 |
| XLOC_005924 | 1.043682 | 0.396957 | 5.045938 | 2.85E-06 | 9.01E-05 | 4.412567 |
| SP6 | 1.299472 | -0.09841 | 5.045913 | 2.85E-06 | 9.01E-05 | 4.412474 |
| UBE2QL1 | -1.45034 | 1.057124 | -5.04576 | 2.85E-06 | 9.01E-05 | 4.411888 |
| CHST5 | 1.989362 | 0.195981 | 5.045756 | 2.85E-06 | 9.01E-05 | 4.411887 |
| LOC100131034 | -1.21906 | 0.748915 | -5.04386 | 2.87E-06 | 9.06E-05 | 4.404835 |
| XLOC_003355 | 1.121025 | 0.617007 | 5.042898 | 2.88E-06 | 9.06E-05 | 4.40124 |
| SLC6A2 | -1.11144 | 0.677508 | -5.04286 | 2.89E-06 | 9.06E-05 | 4.4011 |
| PGCP1 | -2.36931 | 0.002483 | -5.0401 | 2.92E-06 | 9.15E-05 | 4.390826 |
| LOC100505644 | 1.178889 | 0.680428 | 5.039868 | 2.92E-06 | 9.15E-05 | 4.389954 |
| PRSS3 | 1.365379 | -0.23103 | 5.039027 | 2.93E-06 | 9.16E-05 | 4.386822 |
| LOC100505754 | -1.61041 | 0.645124 | -5.03889 | 2.93E-06 | 9.16E-05 | 4.386317 |
| CTIF | -1.20962 | 0.418451 | -5.03381 | 2.99E-06 | 9.31E-05 | 4.367413 |
| FAM161A | -1.1705 | 0.192131 | -5.03202 | 3.01E-06 | 9.37E-05 | 4.36074 |
| ACRBP | 1.119493 | 0.18417 | 5.031595 | 3.02E-06 | 9.38E-05 | 4.359154 |
| FERD3L | -1.38897 | 0.594616 | -5.02743 | 3.07E-06 | 9.48E-05 | 4.343661 |
| CCDC110 | -1.32409 | 0.689788 | -5.02642 | 3.08E-06 | 9.50E-05 | 4.339908 |
| XLOC_012893 | -1.17124 | 0.733807 | -5.02267 | 3.12E-06 | 9.61E-05 | 4.32594 |
| KRT42P | 1.257979 | 0.219804 | 5.021113 | 3.14E-06 | 9.66E-05 | 4.320167 |
| INSM1 | 1.998261 | -0.26627 | 5.020653 | 3.15E-06 | 9.67E-05 | 4.318456 |
| LINC00319 | -1.43856 | -0.02448 | -5.01752 | 3.19E-06 | 9.76E-05 | 4.3068 |
| LOC100292909 | -1.10859 | 0.05143 | -5.01742 | 3.19E-06 | 9.76E-05 | 4.306457 |
| LOC100507056 | 2.174129 | -0.55555 | 5.014187 | 3.23E-06 | 9.86E-05 | 4.294429 |
| GPR97 | -1.05744 | 0.35603 | -5.01226 | 3.25E-06 | 9.91E-05 | 4.287284 |
| LOC389634 | 1.005121 | -0.10991 | 5.009419 | 3.29E-06 | 9.99E-05 | 4.27672 |
| SNTB1 | -1.2785 | -0.12273 | -5.00729 | 3.32E-06 | 0.000101 | 4.268832 |
| DIRC3 | -2.43416 | 1.089914 | -5.00709 | 3.32E-06 | 0.000101 | 4.268087 |
| XLOC_005749 | 1.316011 | 0.496781 | 5.00693 | 3.32E-06 | 0.000101 | 4.26748 |
| S100A10 | 1.442631 | -0.26204 | 5.00362 | 3.37E-06 | 0.000102 | 4.255193 |
| LOC100505882 | 1.416327 | 0.213761 | 5.000764 | 3.41E-06 | 0.000103 | 4.244594 |
| XLOC_l2_008289 | 1.063499 | -0.03389 | 5.000694 | 3.41E-06 | 0.000103 | 4.244338 |
| PITX1 | 1.045059 | 0.019879 | 4.998689 | 3.43E-06 | 0.000103 | 4.236897 |
| XLOC_003160 | -2.0667 | 0.73048 | -4.99724 | 3.45E-06 | 0.000104 | 4.231541 |
| TM4SF4 | 2.676566 | 0.266911 | 4.996295 | 3.47E-06 | 0.000104 | 4.22802 |
| LOC100507309 | -1.05902 | 0.108822 | -4.99607 | 3.47E-06 | 0.000104 | 4.2272 |
| LOC201651 | 1.843782 | -0.14721 | 4.991727 | 3.53E-06 | 0.000105 | 4.211082 |
| HSPB7 | -1.19411 | 0.720084 | -4.98875 | 3.57E-06 | 0.000106 | 4.200053 |
| NME9 | -1.2599 | 0.952632 | -4.9884 | 3.57E-06 | 0.000106 | 4.198742 |
| RIC3 | -1.18031 | 0.017356 | -4.98802 | 3.58E-06 | 0.000106 | 4.197351 |
| ALDH3A1 | -1.4247 | -0.25082 | -4.98671 | 3.60E-06 | 0.000106 | 4.192488 |
| MSMP | 1.001231 | -0.01838 | 4.985006 | 3.62E-06 | 0.000107 | 4.186182 |
| PTPLA | -1.96975 | 0.147778 | -4.98249 | 3.66E-06 | 0.000108 | 4.176876 |
| MAP7D2 | -1.57747 | -0.40965 | -4.97964 | 3.70E-06 | 0.000109 | 4.166315 |
| PM20D1 | -1.61514 | 0.628415 | -4.97941 | 3.70E-06 | 0.000109 | 4.165458 |
| CSRP2 | -1.3401 | -0.20382 | -4.9786 | 3.72E-06 | 0.000109 | 4.162455 |
| CELSR3 | 1.257136 | 0.203469 | 4.970457 | 3.84E-06 | 0.000112 | 4.132331 |
| CNTD1 | -1.23306 | 0.61528 | -4.96951 | 3.85E-06 | 0.000112 | 4.128831 |
| MUC21 | -1.63055 | 0.893475 | -4.96214 | 3.96E-06 | 0.000115 | 4.101578 |
| KCTD8 | -1.83438 | 0.652366 | -4.96025 | 3.99E-06 | 0.000115 | 4.094583 |
| XLOC_012670 | -2.21786 | 0.997267 | -4.9596 | 4.00E-06 | 0.000115 | 4.09221 |
| REEP6 | 1.323347 | 0.378743 | 4.956002 | 4.06E-06 | 0.000117 | 4.078905 |
| REC8 | -1.78289 | -0.11147 | -4.95592 | 4.06E-06 | 0.000117 | 4.078613 |
| UCA1 | 2.633732 | -0.01763 | 4.95309 | 4.11E-06 | 0.000118 | 4.068152 |
| OR3A3 | -2.13194 | 0.551415 | -4.94822 | 4.18E-06 | 0.00012 | 4.050173 |
| PADI2 | 1.754072 | 0.446888 | 4.946319 | 4.22E-06 | 0.00012 | 4.043161 |
| RBP4 | 2.044996 | -0.27957 | 4.939219 | 4.33E-06 | 0.000123 | 4.016976 |
| CHAD | -1.9968 | 0.543772 | -4.93898 | 4.34E-06 | 0.000123 | 4.016107 |
| CELF4 | -1.76884 | 0.273874 | -4.93619 | 4.39E-06 | 0.000124 | 4.005815 |
| TPD52L1 | -1.60096 | 0.362755 | -4.93445 | 4.42E-06 | 0.000124 | 3.999383 |
| TRIM10 | 1.182337 | -0.52251 | 4.931525 | 4.47E-06 | 0.000125 | 3.988619 |
| XLOC_000978 | -1.07967 | -0.02484 | -4.93085 | 4.48E-06 | 0.000126 | 3.986115 |
| XLOC_002411 | -1.11816 | 0.736243 | -4.93034 | 4.49E-06 | 0.000126 | 3.984247 |
| XLOC_009343 | -1.95506 | 0.68486 | -4.92952 | 4.50E-06 | 0.000126 | 3.981228 |
| CERS4 | -1.22104 | 0.149872 | -4.92936 | 4.50E-06 | 0.000126 | 3.980628 |
| TAC4 | -1.48855 | 0.826471 | -4.92926 | 4.51E-06 | 0.000126 | 3.98028 |
| ALB | -2.99249 | 1.041767 | -4.92473 | 4.59E-06 | 0.000128 | 3.963582 |
| TM4SF20 | 2.790972 | 0.959331 | 4.923468 | 4.61E-06 | 0.000128 | 3.958951 |
| CYP27B1 | 1.060028 | -0.14524 | 4.922808 | 4.62E-06 | 0.000129 | 3.956519 |
| SLC3A1 | 1.442621 | 0.437098 | 4.916149 | 4.74E-06 | 0.000131 | 3.932022 |
| C11orf80 | 1.152563 | -0.25745 | 4.913704 | 4.79E-06 | 0.000132 | 3.923028 |
| CXCR4 | -1.33647 | -0.28701 | -4.91183 | 4.82E-06 | 0.000133 | 3.916133 |
| TM4SF1 | 1.10481 | -0.04914 | 4.91133 | 4.83E-06 | 0.000133 | 3.9143 |
| GHRL | -3.34271 | 0.075688 | -4.91078 | 4.84E-06 | 0.000133 | 3.912281 |
| RIMS4 | -1.63603 | 0.383627 | -4.90997 | 4.86E-06 | 0.000133 | 3.909311 |
| CEACAM20 | 1.068997 | -0.00886 | 4.909612 | 4.87E-06 | 0.000133 | 3.907984 |
| XLOC_l2_013480 | 1.045082 | -0.30901 | 4.903597 | 4.98E-06 | 0.000136 | 3.885884 |
| CDHR5 | 1.724188 | 0.018291 | 4.902032 | 5.01E-06 | 0.000136 | 3.880137 |
| STK32B | -1.57339 | -0.23301 | -4.90111 | 5.03E-06 | 0.000137 | 3.876733 |
| LOC100507235 | 1.288848 | -0.00143 | 4.897437 | 5.10E-06 | 0.000138 | 3.863263 |
| FOLR1 | -2.18374 | 0.069393 | -4.89538 | 5.14E-06 | 0.000139 | 3.855704 |
| SLC35D3 | 1.365463 | 0.080554 | 4.895243 | 5.15E-06 | 0.000139 | 3.85521 |
| XLOC_000572 | -1.49578 | 0.464164 | -4.89426 | 5.17E-06 | 0.000139 | 3.851608 |
| XLOC_l2_012552 | -1.7046 | -0.10597 | -4.89367 | 5.18E-06 | 0.00014 | 3.849424 |
| ANKRD24 | -2.02132 | 0.829675 | -4.88992 | 5.25E-06 | 0.000141 | 3.835689 |
| OLFM4 | 5.051074 | -1.63677 | 4.889303 | 5.27E-06 | 0.000141 | 3.833419 |
| XLOC_l2_008140 | -1.11415 | 0.51703 | -4.8866 | 5.32E-06 | 0.000143 | 3.823512 |
| HOXB7 | 1.366982 | 0.19884 | 4.885867 | 5.34E-06 | 0.000143 | 3.820819 |
| HP | 1.459803 | 0.345997 | 4.885723 | 5.34E-06 | 0.000143 | 3.820291 |
| IGLL5 | -1.75266 | -0.36807 | -4.88211 | 5.42E-06 | 0.000144 | 3.807034 |
| CCNJL | -1.7222 | -0.09194 | -4.88094 | 5.44E-06 | 0.000144 | 3.802742 |
| APOE | -1.19599 | -0.02965 | -4.88057 | 5.45E-06 | 0.000144 | 3.801395 |
| ULBP2 | 1.425219 | 0.14902 | 4.87697 | 5.52E-06 | 0.000146 | 3.788216 |
| KCNE3 | 1.144164 | -0.44489 | 4.873679 | 5.60E-06 | 0.000147 | 3.776162 |
| ODZ2 | 1.271986 | 0.103441 | 4.873676 | 5.60E-06 | 0.000147 | 3.776152 |
| NOD1 | 1.060454 | -0.22476 | 4.87177 | 5.64E-06 | 0.000148 | 3.769173 |
| CD27 | -1.16554 | 0.094494 | -4.8717 | 5.64E-06 | 0.000148 | 3.768914 |
| LOC170425 | 1.379832 | 0.374956 | 4.868854 | 5.70E-06 | 0.000149 | 3.758498 |
| PRR21 | -1.58407 | 0.275663 | -4.86767 | 5.73E-06 | 0.00015 | 3.754158 |
| FAS | 1.159126 | -0.17143 | 4.866982 | 5.74E-06 | 0.00015 | 3.751648 |
| TMEM211 | -1.66786 | 0.394465 | -4.85765 | 5.95E-06 | 0.000155 | 3.71751 |
| KBTBD11 | 1.448111 | 0.23737 | 4.856065 | 5.99E-06 | 0.000155 | 3.711728 |
| NPNT | 1.37193 | 0.212499 | 4.853156 | 6.06E-06 | 0.000157 | 3.701099 |
| KLK10 | 1.627434 | 1.027819 | 4.851801 | 6.09E-06 | 0.000157 | 3.696147 |
| NUPR1 | -1.46246 | 0.167944 | -4.85121 | 6.11E-06 | 0.000157 | 3.693997 |
| GPRC5B | -1.21971 | -0.35804 | -4.84818 | 6.18E-06 | 0.000159 | 3.682935 |
| CCDC106 | -1.07319 | -0.32202 | -4.84741 | 6.20E-06 | 0.000159 | 3.6801 |
| MSX1 | 1.589165 | 0.108662 | 4.845837 | 6.23E-06 | 0.00016 | 3.674369 |
| PFKFB4 | 1.337963 | 0.202277 | 4.843423 | 6.29E-06 | 0.000161 | 3.665555 |
| FAM167A | -1.40848 | 0.596306 | -4.83855 | 6.41E-06 | 0.000164 | 3.647771 |
| PPP1R14D | 1.940755 | -0.02105 | 4.836966 | 6.45E-06 | 0.000164 | 3.641997 |
| FCRL1 | -1.25251 | 0.179571 | -4.83689 | 6.45E-06 | 0.000164 | 3.64172 |
| HTR1D | 1.510048 | 0.469448 | 4.836719 | 6.46E-06 | 0.000164 | 3.641099 |
| NGFRAP1 | -1.12997 | -0.44279 | -4.83626 | 6.47E-06 | 0.000164 | 3.639421 |
| KRTAP2-1 | -1.57659 | 0.675775 | -4.83454 | 6.51E-06 | 0.000165 | 3.633147 |
| XLOC_008013 | -1.79319 | 0.841968 | -4.83205 | 6.58E-06 | 0.000167 | 3.624067 |
| PYDC1 | 1.254722 | -0.06388 | 4.831489 | 6.59E-06 | 0.000167 | 3.622029 |
| LOC100128593 | 1.085296 | 0.053147 | 4.827712 | 6.69E-06 | 0.000169 | 3.608266 |
| LOC100506995 | 1.866675 | 0.481727 | 4.825959 | 6.73E-06 | 0.00017 | 3.601878 |
| ZP3 | 1.383839 | 0.170886 | 4.825802 | 6.74E-06 | 0.00017 | 3.601305 |
| DDC | 1.03941 | -0.29716 | 4.825767 | 6.74E-06 | 0.00017 | 3.601177 |
| OR7A17 | -1.60415 | 1.098804 | -4.82041 | 6.88E-06 | 0.000172 | 3.58166 |
| P2RY8 | -1.13853 | -0.05774 | -4.82 | 6.89E-06 | 0.000172 | 3.580164 |
| PADI3 | -1.62522 | 1.167947 | -4.81606 | 6.99E-06 | 0.000174 | 3.565829 |
| ZNF415 | -1.25434 | -0.28837 | -4.81267 | 7.09E-06 | 0.000176 | 3.553508 |
| ZNF488 | 1.698181 | -0.25623 | 4.809353 | 7.18E-06 | 0.000177 | 3.541442 |
| LOC284570 | 1.187872 | 0.081866 | 4.808446 | 7.20E-06 | 0.000178 | 3.538142 |
| TM7SF2 | -1.00811 | 0.349685 | -4.8071 | 7.24E-06 | 0.000178 | 3.533259 |
| PRSS2 | 1.133888 | -0.1222 | 4.806242 | 7.27E-06 | 0.000178 | 3.53013 |
| PROM1 | 1.019328 | -0.15722 | 4.803693 | 7.34E-06 | 0.000179 | 3.520866 |
| RANBP3L | -1.91152 | 1.720665 | -4.80292 | 7.36E-06 | 0.00018 | 3.51805 |
| EGR4 | 1.223799 | 0.742934 | 4.797286 | 7.52E-06 | 0.000183 | 3.497593 |
| LOC100505920 | -1.7693 | 0.435895 | -4.7961 | 7.55E-06 | 0.000184 | 3.493301 |
| HIST1H3J | 1.028881 | -0.0583 | 4.793809 | 7.62E-06 | 0.000185 | 3.48497 |
| XLOC_001485 | -1.11348 | 0.396527 | -4.7926 | 7.66E-06 | 0.000185 | 3.480568 |
| TGFB1I1 | -1.16072 | 0.331389 | -4.78858 | 7.78E-06 | 0.000188 | 3.466011 |
| AREG | 1.454374 | -0.37869 | 4.788535 | 7.78E-06 | 0.000188 | 3.465832 |
| LYPD6B | -1.28395 | -0.24729 | -4.78523 | 7.88E-06 | 0.000189 | 3.453846 |
| PRR5L | 1.093786 | -0.1695 | 4.784872 | 7.89E-06 | 0.00019 | 3.452545 |
| TMC7 | 1.22255 | -0.48987 | 4.783302 | 7.94E-06 | 0.000191 | 3.446852 |
| XLOC_l2_013863 | -1.05507 | 0.445452 | -4.78268 | 7.96E-06 | 0.000191 | 3.444598 |
| SLC2A4 | -1.98348 | 0.89539 | -4.78082 | 8.01E-06 | 0.000192 | 3.437857 |
| TNFRSF11B | 1.638416 | 0.198843 | 4.778325 | 8.09E-06 | 0.000193 | 3.428812 |
| GABRG2 | -1.59331 | 0.530899 | -4.77412 | 8.22E-06 | 0.000196 | 3.413561 |
| DYX1C1 | 1.067233 | -0.2536 | 4.770589 | 8.33E-06 | 0.000197 | 3.400791 |
| HAPLN3 | -1.20689 | -0.20189 | -4.7693 | 8.38E-06 | 0.000198 | 3.396115 |
| XLOC_001263 | -1.5123 | 0.202099 | -4.76386 | 8.55E-06 | 0.000202 | 3.376433 |
| LOC648987 | 1.307144 | 0.092304 | 4.763583 | 8.56E-06 | 0.000202 | 3.375438 |
| SIGLEC1 | -1.07791 | -0.09549 | -4.76197 | 8.62E-06 | 0.000203 | 3.369601 |
| ST3GAL6 | -1.07315 | -0.14006 | -4.75406 | 8.88E-06 | 0.000208 | 3.340998 |
| XLOC_l2_006745 | 1.260513 | -0.21493 | 4.753861 | 8.89E-06 | 0.000208 | 3.340283 |
| GPR37L1 | 1.420161 | -0.1865 | 4.753749 | 8.89E-06 | 0.000208 | 3.339879 |
| INPP1 | 1.052212 | -0.32029 | 4.749319 | 9.04E-06 | 0.00021 | 3.323875 |
| HIST1H2AI | 1.399528 | -0.15836 | 4.741827 | 9.31E-06 | 0.000216 | 3.296827 |
| PKIB | -1.16678 | -0.26167 | -4.74155 | 9.32E-06 | 0.000216 | 3.295835 |
| PLEKHM3 | -1.06136 | 0.539686 | -4.73938 | 9.40E-06 | 0.000217 | 3.28801 |
| ZDBF2 | -1.18523 | -0.3516 | -4.73905 | 9.41E-06 | 0.000217 | 3.286807 |
| FEV | 1.540404 | -0.14371 | 4.728262 | 9.80E-06 | 0.000224 | 3.247912 |
| ABCA8 | -1.07885 | 0.047439 | -4.72685 | 9.86E-06 | 0.000225 | 3.242825 |
| P2RY4 | -1.73077 | 0.863981 | -4.72579 | 9.90E-06 | 0.000226 | 3.238989 |
| LOC100131864 | -1.5993 | 0.193015 | -4.72531 | 9.92E-06 | 0.000226 | 3.23726 |
| ATP1B2 | -1.10844 | -0.0114 | -4.72446 | 9.95E-06 | 0.000226 | 3.234202 |
| Q6ILE4 | -1.09096 | 0.365867 | -4.72208 | 1.00E-05 | 0.000228 | 3.225636 |
| PPFIA3 | 1.139058 | 0.056095 | 4.72038 | 1.01E-05 | 0.000229 | 3.219523 |
| SERPINA5 | -2.39895 | 0.016597 | -4.71839 | 1.02E-05 | 0.000231 | 3.212378 |
| PLCB3 | 1.034321 | -0.18475 | 4.714456 | 1.03E-05 | 0.000233 | 3.198204 |
| ZNF542 | -1.038 | -0.23077 | -4.71434 | 1.03E-05 | 0.000233 | 3.197773 |
| MZB1 | -1.54727 | 0.220486 | -4.71337 | 1.04E-05 | 0.000233 | 3.19429 |
| IL22RA1 | 1.083903 | -0.05466 | 4.707964 | 1.06E-05 | 0.000238 | 3.174858 |
| KANK4 | -1.79908 | -0.67907 | -4.70657 | 1.07E-05 | 0.000238 | 3.169852 |
| NT5E | 1.120087 | 0.172639 | 4.705857 | 1.07E-05 | 0.000239 | 3.167288 |
| XLOC_005180 | -1.26598 | 0.644081 | -4.70374 | 1.08E-05 | 0.000241 | 3.159692 |
| C8orf12 | -1.65545 | 0.680801 | -4.70267 | 1.08E-05 | 0.000241 | 3.155838 |
| PRKAA2 | -1.39568 | 0.590907 | -4.70128 | 1.09E-05 | 0.000242 | 3.150838 |
| HKDC1 | 1.90119 | -0.17616 | 4.700672 | 1.09E-05 | 0.000242 | 3.148658 |
| C2orf74 | -1.26141 | -0.18298 | -4.70032 | 1.09E-05 | 0.000243 | 3.147385 |
| FAM124B | -1.01401 | 0.275379 | -4.69744 | 1.10E-05 | 0.000245 | 3.13705 |
| KCNJ18 | -1.62505 | -0.41 | -4.69413 | 1.12E-05 | 0.000248 | 3.125161 |
| XLOC_003296 | -2.18233 | 0.76437 | -4.6913 | 1.13E-05 | 0.00025 | 3.115006 |
| RGN | -1.71767 | -0.0116 | -4.69128 | 1.13E-05 | 0.00025 | 3.114931 |
| FAM65B | -1.14719 | -0.27406 | -4.67865 | 1.19E-05 | 0.000259 | 3.069672 |
| CST1 | 1.207519 | 0.376387 | 4.676956 | 1.19E-05 | 0.00026 | 3.063592 |
| EMID2 | -1.21217 | 0.353543 | -4.67074 | 1.22E-05 | 0.000265 | 3.041336 |
| LOC100128398 | -1.16069 | -0.29164 | -4.66913 | 1.23E-05 | 0.000266 | 3.035575 |
| MESP2 | 1.117484 | 0.078755 | 4.668296 | 1.23E-05 | 0.000267 | 3.03259 |
| XLOC_000712 | -1.04607 | 0.363393 | -4.66616 | 1.24E-05 | 0.000269 | 3.024933 |
| FAM151B | 1.085765 | -0.21359 | 4.66277 | 1.26E-05 | 0.000271 | 3.012824 |
| LOC393078 | -1.62994 | 0.474416 | -4.6607 | 1.27E-05 | 0.000272 | 3.005411 |
| XLOC_l2_006027 | -1.749 | 0.717708 | -4.65886 | 1.28E-05 | 0.000274 | 2.998832 |
| RTDR1 | -1.34228 | 0.721958 | -4.65711 | 1.29E-05 | 0.000275 | 2.992599 |
| XLOC_l2_007986 | 1.618695 | 0.842142 | 4.654371 | 1.30E-05 | 0.000278 | 2.982807 |
| LRRC17 | -1.97853 | 0.003818 | -4.65343 | 1.30E-05 | 0.000278 | 2.979444 |
| LOC100507554 | -1.57094 | 0.360007 | -4.65206 | 1.31E-05 | 0.000279 | 2.974558 |
| SCTR | -1.52031 | -0.1646 | -4.65194 | 1.31E-05 | 0.000279 | 2.974118 |
| GPR116 | -1.32401 | 0.378192 | -4.65181 | 1.31E-05 | 0.000279 | 2.973649 |
| DACH1 | 1.525441 | 0.41192 | 4.651469 | 1.31E-05 | 0.000279 | 2.97244 |
| OR10G4 | -1.88257 | 1.186373 | -4.65133 | 1.31E-05 | 0.000279 | 2.971955 |
| TMEM139 | 1.038256 | 0.296757 | 4.648545 | 1.33E-05 | 0.000281 | 2.962 |
| NAT2 | 2.354718 | 0.06993 | 4.64741 | 1.33E-05 | 0.000282 | 2.95795 |
| LOC100507417 | -1.60433 | 0.683376 | -4.63859 | 1.38E-05 | 0.000291 | 2.92648 |
| PTGDR2 | -1.24962 | 0.050094 | -4.63633 | 1.39E-05 | 0.000292 | 2.918418 |
| XLOC_l2_001134 | 1.511297 | -0.04587 | 4.634425 | 1.40E-05 | 0.000294 | 2.91164 |
| GPC3 | -1.26284 | 0.291468 | -4.63244 | 1.41E-05 | 0.000295 | 2.904579 |
| NEAT1 | -1.25874 | -0.0379 | -4.63053 | 1.42E-05 | 0.000297 | 2.897773 |
| C18orf56 | 1.182163 | -0.25492 | 4.627368 | 1.44E-05 | 0.000299 | 2.886501 |
| DCAF12L1 | -1.32854 | 1.057252 | -4.62724 | 1.44E-05 | 0.000299 | 2.886062 |
| XLOC_l2_005415 | -1.06836 | 0.289371 | -4.6268 | 1.44E-05 | 0.000299 | 2.884469 |
| FAM150B | -2.04729 | 0.258924 | -4.62665 | 1.44E-05 | 0.000299 | 2.883954 |
| EFHA2 | -1.55284 | 0.197022 | -4.62613 | 1.45E-05 | 0.0003 | 2.882075 |
| MALL | 2.266452 | -0.57088 | 4.626109 | 1.45E-05 | 0.0003 | 2.882017 |
| LGALS4 | 1.007841 | -0.06095 | 4.625913 | 1.45E-05 | 0.0003 | 2.881318 |
| CABP1 | -1.33775 | -0.15553 | -4.62421 | 1.46E-05 | 0.000301 | 2.875249 |
| KIF13A | 1.195511 | -0.23865 | 4.624058 | 1.46E-05 | 0.000301 | 2.874717 |
| IRX2 | -2.89787 | -1.17931 | -4.6232 | 1.46E-05 | 0.000302 | 2.87168 |
| XLOC_l2_009947 | 1.707467 | 0.046919 | 4.62315 | 1.46E-05 | 0.000302 | 2.871484 |
| C20orf202 | 1.021667 | 0.040881 | 4.618386 | 1.49E-05 | 0.000306 | 2.854537 |
| HTR1E | -1.32216 | 1.079329 | -4.61096 | 1.53E-05 | 0.000313 | 2.828143 |
| RIMKLA | 1.168264 | 0.014227 | 4.60954 | 1.54E-05 | 0.000314 | 2.823089 |
| FLJ22763 | 3.370436 | 0.414634 | 4.607503 | 1.55E-05 | 0.000316 | 2.81585 |
| GPRC5C | -1.0224 | 0.238069 | -4.60737 | 1.55E-05 | 0.000316 | 2.815364 |
| SLC18A2 | -1.17492 | -0.15264 | -4.60686 | 1.56E-05 | 0.000316 | 2.813574 |
| CHST3 | -1.80407 | 0.259578 | -4.60684 | 1.56E-05 | 0.000316 | 2.81351 |
| FAR2 | 1.548303 | -0.60328 | 4.605664 | 1.56E-05 | 0.000316 | 2.809321 |
| GJB2 | 1.195934 | -0.52173 | 4.604483 | 1.57E-05 | 0.000317 | 2.805126 |
| CSH1 | -1.34789 | 0.984776 | -4.60067 | 1.59E-05 | 0.00032 | 2.791607 |
| SLC2A12 | -2.39288 | 0.669952 | -4.5991 | 1.60E-05 | 0.000322 | 2.786035 |
| PRKD1 | -1.01949 | 0.057913 | -4.5991 | 1.60E-05 | 0.000322 | 2.786015 |
| GSTT2B | -1.33222 | -0.10009 | -4.59892 | 1.60E-05 | 0.000322 | 2.785379 |
| LOC100507333 | -1.0505 | 0.009064 | -4.59836 | 1.61E-05 | 0.000322 | 2.783385 |
| KIAA0125 | -1.74667 | 0.041162 | -4.59589 | 1.62E-05 | 0.000324 | 2.774614 |
| EPHB6 | -1.60081 | -0.24491 | -4.59583 | 1.62E-05 | 0.000324 | 2.774412 |
| SLC5A1 | 1.741362 | -0.24757 | 4.592485 | 1.64E-05 | 0.000328 | 2.762553 |
| PLVAP | -1.00856 | 0.05125 | -4.58932 | 1.66E-05 | 0.000331 | 2.751346 |
| LOC284578 | -1.25483 | -0.16142 | -4.58329 | 1.70E-05 | 0.000337 | 2.72998 |
| TFPI2 | 1.760883 | -0.09366 | 4.579094 | 1.73E-05 | 0.000341 | 2.715113 |
| RAPGEF4 | -1.28352 | 0.139832 | -4.5773 | 1.74E-05 | 0.000343 | 2.708749 |
| MPV17L | 1.06187 | 0.136571 | 4.570458 | 1.79E-05 | 0.00035 | 2.684557 |
| KRT16P2 | 1.7616 | 0.240841 | 4.570123 | 1.79E-05 | 0.000351 | 2.683373 |
| FRMD1 | -1.21351 | 0.159942 | -4.56666 | 1.81E-05 | 0.000353 | 2.671144 |
| SPINK2 | -2.05899 | -0.06385 | -4.564 | 1.83E-05 | 0.000356 | 2.661732 |
| PRAP1 | 1.973676 | 0.910475 | 4.56369 | 1.83E-05 | 0.000356 | 2.660636 |
| ACOX2 | 1.101598 | -0.0468 | 4.562679 | 1.84E-05 | 0.000357 | 2.657066 |
| SMPX | 1.383299 | 0.643137 | 4.560016 | 1.86E-05 | 0.00036 | 2.64766 |
| XLOC_011486 | -1.83241 | 1.307633 | -4.55928 | 1.86E-05 | 0.000361 | 2.645075 |
| MUM1L1 | -1.70278 | 0.286572 | -4.55849 | 1.87E-05 | 0.000361 | 2.642271 |
| ZNF300P1 | -1.37314 | -0.12621 | -4.55618 | 1.88E-05 | 0.000363 | 2.634101 |
| SLC14A2 | -1.40744 | 0.673254 | -4.55347 | 1.90E-05 | 0.000366 | 2.624543 |
| ADAM28 | -1.08806 | -0.16309 | -4.55273 | 1.91E-05 | 0.000367 | 2.621935 |
| LOC100653210 | -1.89615 | -0.04886 | -4.55127 | 1.92E-05 | 0.000368 | 2.616798 |
| ABCC5 | -1.1335 | -0.06589 | -4.54825 | 1.94E-05 | 0.000372 | 2.60614 |
| ESCO2 | 1.081109 | -0.34125 | 4.547012 | 1.95E-05 | 0.000373 | 2.601774 |
| PDSS1 | 1.134462 | -0.06175 | 4.546937 | 1.95E-05 | 0.000373 | 2.601507 |
| XLOC_014403 | -1.45652 | 0.400171 | -4.54551 | 1.96E-05 | 0.000374 | 2.596479 |
| CCKAR | -1.51524 | 1.281081 | -4.54025 | 2.00E-05 | 0.000381 | 2.57793 |
| XLOC_l2_006021 | 1.947559 | 0.557551 | 4.539885 | 2.00E-05 | 0.000381 | 2.576655 |
| FOXD1 | 2.359262 | 0.744648 | 4.53515 | 2.04E-05 | 0.000388 | 2.559981 |
| LOC100287865 | -1.38929 | 0.244629 | -4.53469 | 2.04E-05 | 0.000388 | 2.558345 |
| SOSTDC1 | -1.58376 | -0.20344 | -4.53426 | 2.05E-05 | 0.000388 | 2.556859 |
| XLOC_009023 | -1.02459 | 0.225247 | -4.53372 | 2.05E-05 | 0.000389 | 2.554953 |
| ISX | 2.512401 | 1.548389 | 4.532562 | 2.06E-05 | 0.00039 | 2.55087 |
| CHRM3 | -1.50716 | -0.27958 | -4.53231 | 2.06E-05 | 0.00039 | 2.549981 |
| KCNJ12 | -1.68041 | -0.3926 | -4.52774 | 2.10E-05 | 0.000396 | 2.53391 |
| GNAZ | -1.01702 | 0.104813 | -4.52756 | 2.10E-05 | 0.000396 | 2.533284 |
| XLOC_001755 | -1.61283 | 0.593525 | -4.5252 | 2.12E-05 | 0.000399 | 2.52499 |
| NLRP14 | -1.54221 | 1.105892 | -4.52508 | 2.12E-05 | 0.000399 | 2.524543 |
| LOC93432 | 1.216602 | 0.897638 | 4.524102 | 2.12E-05 | 0.0004 | 2.521113 |
| ERP27 | -2.00561 | 0.231735 | -4.52392 | 2.13E-05 | 0.0004 | 2.52046 |
| MST1P9 | -1.71611 | -0.3715 | -4.51995 | 2.16E-05 | 0.000404 | 2.506508 |
| LOC645202 | -1.56756 | 0.710878 | -4.51962 | 2.16E-05 | 0.000405 | 2.505351 |
| XLOC_007604 | -1.5434 | 0.13037 | -4.51634 | 2.19E-05 | 0.000409 | 2.493825 |
| ANKRD29 | -1.14232 | -0.33248 | -4.51576 | 2.19E-05 | 0.00041 | 2.491785 |
| TF | -1.48886 | 0.59718 | -4.51268 | 2.22E-05 | 0.000413 | 2.480987 |
| TTC9B | 1.552044 | 0.410572 | 4.510391 | 2.24E-05 | 0.000414 | 2.472953 |
| POLE2 | 1.051391 | -0.384 | 4.50759 | 2.26E-05 | 0.000418 | 2.463125 |
| EYA2 | -1.54418 | -0.39494 | -4.50757 | 2.26E-05 | 0.000418 | 2.463052 |
| XLOC_008945 | -2.1493 | 0.07977 | -4.50724 | 2.26E-05 | 0.000418 | 2.461907 |
| CWH43 | -2.33061 | -0.66988 | -4.50658 | 2.27E-05 | 0.000419 | 2.459586 |
| GP2 | 2.374259 | -0.37225 | 4.500913 | 2.32E-05 | 0.000426 | 2.439708 |
| LOC100507069 | -1.47624 | 1.007555 | -4.50026 | 2.32E-05 | 0.000427 | 2.437423 |
| LOC100505601 | -1.36145 | 0.428754 | -4.49533 | 2.37E-05 | 0.000433 | 2.420141 |
| LOC100132593 | -1.51764 | 0.627753 | -4.49448 | 2.37E-05 | 0.000434 | 2.417165 |
| LOC100131015 | -1.20424 | 0.176159 | -4.49229 | 2.39E-05 | 0.000437 | 2.409505 |
| LOC728606 | -1.34096 | -0.27645 | -4.49224 | 2.39E-05 | 0.000437 | 2.409315 |
| ARX | 1.072076 | 0.044091 | 4.492059 | 2.40E-05 | 0.000437 | 2.408693 |
| LOC100288911 | 1.00725 | -0.23211 | 4.490636 | 2.41E-05 | 0.000439 | 2.40371 |
| GDA | 1.344152 | -0.19383 | 4.487712 | 2.43E-05 | 0.000442 | 2.393477 |
| C6orf97 | 1.2873 | -0.1731 | 4.485818 | 2.45E-05 | 0.000445 | 2.386848 |
| ZNF662 | -1.55977 | -0.38587 | -4.48015 | 2.50E-05 | 0.000453 | 2.367024 |
| TPRXL | 1.503018 | 0.84856 | 4.476572 | 2.54E-05 | 0.000457 | 2.354523 |
| XLOC_l2_000356 | -1.57363 | 0.299874 | -4.47336 | 2.57E-05 | 0.000462 | 2.343309 |
| POLR3G | 1.337152 | -0.06186 | 4.470973 | 2.59E-05 | 0.000465 | 2.334966 |
| GABRB3 | -1.13716 | -0.35813 | -4.46784 | 2.62E-05 | 0.00047 | 2.324045 |
| XLOC_007700 | -1.12544 | 0.17361 | -4.46644 | 2.64E-05 | 0.000471 | 2.319142 |
| CRIP3 | 1.333037 | 0.328833 | 4.465869 | 2.64E-05 | 0.000471 | 2.317148 |
| DUSP19 | -1.31035 | 0.557057 | -4.46516 | 2.65E-05 | 0.000472 | 2.314679 |
| SMR3A | -1.37503 | 0.235707 | -4.4651 | 2.65E-05 | 0.000472 | 2.314458 |
| FXYD4 | -2.62519 | 0.46064 | -4.46451 | 2.65E-05 | 0.000473 | 2.312423 |
| TFAP2A | 1.254573 | 0.698641 | 4.460534 | 2.69E-05 | 0.000478 | 2.29854 |
| IP6K3 | -1.26829 | -0.01805 | -4.45834 | 2.72E-05 | 0.000481 | 2.290903 |
| C11orf86 | 2.886865 | -1.23161 | 4.457945 | 2.72E-05 | 0.000481 | 2.289512 |
| ADA | -1.43563 | 0.154571 | -4.45644 | 2.74E-05 | 0.000483 | 2.284252 |
| KLHL5 | -1.00425 | -0.34089 | -4.45547 | 2.75E-05 | 0.000484 | 2.280887 |
| GSTA3 | -2.53372 | 1.344758 | -4.45378 | 2.76E-05 | 0.000487 | 2.274983 |
| LOC100130345 | -1.05523 | 0.542946 | -4.44472 | 2.86E-05 | 0.000502 | 2.243461 |
| XLOC_012145 | -1.09521 | 0.568576 | -4.44444 | 2.86E-05 | 0.000502 | 2.242482 |
| DIRAS1 | -1.21365 | -0.0119 | -4.44245 | 2.88E-05 | 0.000504 | 2.235542 |
| COL6A5 | -1.43765 | 1.219458 | -4.43399 | 2.97E-05 | 0.000517 | 2.206126 |
| LOC389493 | -1.36609 | -0.12427 | -4.42888 | 3.03E-05 | 0.000525 | 2.188376 |
| C10orf129 | -1.6161 | 0.382311 | -4.42594 | 3.06E-05 | 0.00053 | 2.178172 |
| NXF3 | 1.490582 | 0.398482 | 4.422452 | 3.10E-05 | 0.000534 | 2.166081 |
| MID2 | -1.0682 | -0.1371 | -4.42223 | 3.11E-05 | 0.000534 | 2.165322 |
| MYOM3 | 1.25407 | 0.84492 | 4.421389 | 3.11E-05 | 0.000535 | 2.162392 |
| SNORA48 | -1.09095 | 0.304461 | -4.42027 | 3.13E-05 | 0.000536 | 2.158498 |
| LOC100507520 | -1.06195 | 0.213373 | -4.41789 | 3.16E-05 | 0.00054 | 2.150253 |
| GLTPD2 | 1.415514 | 0.323364 | 4.416247 | 3.17E-05 | 0.000542 | 2.14456 |
| SLAMF7 | -1.24512 | -0.01114 | -4.41517 | 3.19E-05 | 0.000544 | 2.140842 |
| LOC100144602 | -1.38707 | 0.976555 | -4.41341 | 3.21E-05 | 0.000546 | 2.134719 |
| DSG3 | 2.186856 | 1.700593 | 4.410497 | 3.24E-05 | 0.000551 | 2.124634 |
| EPHX4 | 1.809299 | 0.814626 | 4.405851 | 3.30E-05 | 0.00056 | 2.108546 |
| FLJ26086 | 1.923785 | -0.13243 | 4.405488 | 3.30E-05 | 0.00056 | 2.107287 |
| IGJ | -1.19756 | -0.20004 | -4.40367 | 3.33E-05 | 0.000563 | 2.101 |
| CDKN3 | 1.123429 | -0.06858 | 4.403442 | 3.33E-05 | 0.000563 | 2.100207 |
| CCDC169 | -1.02684 | -0.22537 | -4.39865 | 3.39E-05 | 0.00057 | 2.08362 |
| MS4A8B | 1.156924 | 0.134283 | 4.397808 | 3.40E-05 | 0.000571 | 2.080715 |
| TMED6 | -2.18592 | 0.123558 | -4.39549 | 3.43E-05 | 0.000574 | 2.072688 |
| LRRC19 | 2.590043 | 0.100464 | 4.393684 | 3.45E-05 | 0.000576 | 2.06646 |
| DKFZP761C1711 | -1.15261 | 0.449689 | -4.39222 | 3.47E-05 | 0.000578 | 2.061391 |
| XLOC_010065 | -1.13267 | 0.362221 | -4.39208 | 3.47E-05 | 0.000578 | 2.060911 |
| NLGN4X | -1.36965 | -0.14414 | -4.39103 | 3.48E-05 | 0.000579 | 2.057288 |
| PGC | -2.26552 | -0.88563 | -4.38985 | 3.50E-05 | 0.000581 | 2.053211 |
| DHRS9 | 1.818311 | -0.56594 | 4.389553 | 3.50E-05 | 0.000581 | 2.052186 |
| IGFL2 | 1.01651 | 0.758251 | 4.387533 | 3.53E-05 | 0.000585 | 2.045208 |
| RBP7 | -1.09787 | 0.094566 | -4.38634 | 3.55E-05 | 0.000586 | 2.041079 |
| IRAK2 | 1.1437 | -0.26655 | 4.385138 | 3.56E-05 | 0.000588 | 2.036941 |
| DAPL1 | 1.273071 | 0.077903 | 4.384674 | 3.57E-05 | 0.000589 | 2.03534 |
| C1orf190 | -1.05541 | -0.04845 | -4.38206 | 3.60E-05 | 0.000593 | 2.026317 |
| GUCY1B2 | 1.445584 | 0.83499 | 4.381093 | 3.61E-05 | 0.000595 | 2.02298 |
| LOC100131043 | -1.21068 | 0.194263 | -4.37435 | 3.71E-05 | 0.000609 | 1.999722 |
| OR12D2 | -1.53264 | 1.077643 | -4.3721 | 3.74E-05 | 0.000612 | 1.991964 |
| SPINK4 | 3.347833 | -0.26944 | 4.372004 | 3.74E-05 | 0.000612 | 1.99164 |
| XLOC_l2_011291 | 1.060772 | 0.551431 | 4.364849 | 3.84E-05 | 0.000628 | 1.966996 |
| KCNE4 | -1.5729 | -0.25171 | -4.35895 | 3.92E-05 | 0.00064 | 1.946705 |
| APC2 | -1.54339 | 0.096417 | -4.35844 | 3.93E-05 | 0.00064 | 1.944935 |
| XLOC_007928 | -1.32479 | 0.304119 | -4.35739 | 3.94E-05 | 0.000642 | 1.941313 |
| CENPH | 1.139508 | -0.13324 | 4.354257 | 3.99E-05 | 0.000648 | 1.930558 |
| TINAG | 1.784427 | 0.715878 | 4.352108 | 4.02E-05 | 0.000651 | 1.92317 |
| XLOC_011327 | -1.24916 | 0.10877 | -4.35144 | 4.03E-05 | 0.000651 | 1.920887 |
| LOC100129931 | 1.345176 | 0.173195 | 4.347612 | 4.09E-05 | 0.000657 | 1.907723 |
| PSAPL1 | -2.32571 | -0.70296 | -4.3457 | 4.12E-05 | 0.000661 | 1.901161 |
| XLOC_013301 | -1.59942 | 0.419728 | -4.34544 | 4.12E-05 | 0.000661 | 1.900253 |
| AGXT | 1.147267 | 0.071485 | 4.341911 | 4.17E-05 | 0.000669 | 1.888152 |
| ARL9 | 1.450132 | -0.0582 | 4.341576 | 4.18E-05 | 0.000669 | 1.887 |
| XLOC_010356 | -1.84291 | -0.16566 | -4.3406 | 4.19E-05 | 0.000671 | 1.88366 |
| XLOC_006705 | 1.165889 | 0.301119 | 4.340041 | 4.20E-05 | 0.000672 | 1.881732 |
| MDFI | 1.388736 | -0.09845 | 4.33623 | 4.26E-05 | 0.00068 | 1.86866 |
| LOC401317 | -1.1393 | 0.666996 | -4.33565 | 4.27E-05 | 0.000681 | 1.866671 |
| LINC00336 | 1.055154 | 0.599637 | 4.335108 | 4.28E-05 | 0.000681 | 1.864813 |
| RAD51AP1 | 1.310108 | -0.28806 | 4.330965 | 4.35E-05 | 0.000688 | 1.850613 |
| PCDH7 | -1.66759 | -0.24553 | -4.33072 | 4.35E-05 | 0.000689 | 1.849765 |
| TRPV6 | -1.15045 | -0.23779 | -4.3305 | 4.35E-05 | 0.000689 | 1.849023 |
| BIRC3 | -1.0097 | -0.02628 | -4.32087 | 4.51E-05 | 0.000708 | 1.816051 |
| SYPL2 | -1.12802 | 0.379175 | -4.32084 | 4.51E-05 | 0.000708 | 1.815928 |
| XLOC_l2_004840 | -1.33627 | -0.20745 | -4.31929 | 4.54E-05 | 0.000711 | 1.810644 |
| IL7R | -1.06083 | -0.26577 | -4.31516 | 4.60E-05 | 0.00072 | 1.796512 |
| FAM64A | 1.250826 | -0.05016 | 4.31347 | 4.63E-05 | 0.000724 | 1.790739 |
| IGFBP5 | -1.43262 | 0.041364 | -4.30985 | 4.69E-05 | 0.000732 | 1.778383 |
| SKA3 | 1.150779 | -0.16969 | 4.30861 | 4.72E-05 | 0.000733 | 1.774129 |
| XLOC_003156 | -1.54185 | 1.178794 | -4.30626 | 4.76E-05 | 0.000738 | 1.766118 |
| XLOC_010998 | -1.17819 | 0.033098 | -4.30626 | 4.76E-05 | 0.000738 | 1.766111 |
| LOC100507149 | -1.19852 | 0.245747 | -4.29983 | 4.87E-05 | 0.000751 | 1.74415 |
| GNRH2 | -1.32705 | 0.67505 | -4.29888 | 4.89E-05 | 0.000753 | 1.74091 |
| XLOC_005777 | 1.69372 | 0.442485 | 4.297572 | 4.91E-05 | 0.000755 | 1.736453 |
| XLOC_000709 | -1.68903 | 1.439062 | -4.29717 | 4.92E-05 | 0.000756 | 1.735097 |
| XLOC_011088 | -1.10036 | 0.715943 | -4.29653 | 4.93E-05 | 0.000757 | 1.732884 |
| XLOC_005101 | -1.01053 | 0.230479 | -4.29447 | 4.97E-05 | 0.000762 | 1.725883 |
| INE1 | -1.10286 | 0.032147 | -4.29345 | 4.98E-05 | 0.000763 | 1.722416 |
| LOC100505853 | -1.51184 | 0.611659 | -4.29182 | 5.01E-05 | 0.000767 | 1.716855 |
| SYNPR | 1.68337 | -0.3454 | 4.28982 | 5.05E-05 | 0.000771 | 1.710029 |
| RXFP4 | -1.93354 | 0.543833 | -4.28852 | 5.07E-05 | 0.000773 | 1.7056 |
| KRT80 | 1.794763 | -0.02578 | 4.288205 | 5.08E-05 | 0.000774 | 1.704527 |
| TMEM132A | -1.33501 | 0.059199 | -4.28721 | 5.10E-05 | 0.000775 | 1.701152 |
| GPR128 | 2.825854 | 0.172061 | 4.286656 | 5.11E-05 | 0.000775 | 1.699253 |
| CES2 | 1.14617 | -0.15214 | 4.28531 | 5.13E-05 | 0.000778 | 1.694668 |
| C14orf176 | 1.59605 | -0.01143 | 4.283631 | 5.17E-05 | 0.000781 | 1.688954 |
| PCDHB9 | 1.322277 | 0.031618 | 4.282159 | 5.19E-05 | 0.000783 | 1.683943 |
| ABCG8 | 2.023078 | 0.089841 | 4.280676 | 5.22E-05 | 0.000787 | 1.678896 |
| LOC100132790 | -1.04792 | 0.495362 | -4.27964 | 5.24E-05 | 0.000788 | 1.675383 |
| PKNOX2 | -1.15382 | 0.753394 | -4.27813 | 5.27E-05 | 0.000791 | 1.670239 |
| XLOC_002243 | -1.4779 | 0.33605 | -4.27419 | 5.35E-05 | 0.000801 | 1.656833 |
| EFNA3 | 1.108578 | 0.166504 | 4.27402 | 5.35E-05 | 0.000801 | 1.656261 |
| TMCO5A | -1.39726 | 0.393374 | -4.27306 | 5.37E-05 | 0.000802 | 1.653013 |
| SLC9A3 | -1.09744 | 0.709391 | -4.27305 | 5.37E-05 | 0.000802 | 1.652952 |
| ITGBL1 | -1.33321 | 0.412114 | -4.26491 | 5.53E-05 | 0.000817 | 1.625311 |
| LGR5 | 1.28514 | 0.583547 | 4.264714 | 5.53E-05 | 0.000818 | 1.624646 |
| LRRIQ4 | 1.256289 | 0.151556 | 4.26264 | 5.58E-05 | 0.000822 | 1.617607 |
| XLOC_013754 | 1.074382 | 0.559699 | 4.262031 | 5.59E-05 | 0.000823 | 1.615542 |
| MLN | 3.264386 | 1.591814 | 4.260939 | 5.61E-05 | 0.000825 | 1.611837 |
| LOC100505592 | 1.422507 | -0.1804 | 4.260727 | 5.61E-05 | 0.000826 | 1.611117 |
| TFPI | 1.123097 | -0.15416 | 4.260082 | 5.63E-05 | 0.000827 | 1.608928 |
| FER1L4 | -1.39629 | -0.07196 | -4.25871 | 5.66E-05 | 0.00083 | 1.604262 |
| XLOC_l2_012855 | -1.07032 | 0.260381 | -4.25843 | 5.66E-05 | 0.000831 | 1.603337 |
| ADRA2C | 1.01776 | 0.047168 | 4.256049 | 5.71E-05 | 0.000836 | 1.595251 |
| FBLN1 | -1.2887 | -0.24708 | -4.25592 | 5.71E-05 | 0.000836 | 1.594828 |
| SLC22A12 | -1.04292 | 0.344257 | -4.2548 | 5.74E-05 | 0.000838 | 1.591025 |
| NEK2 | 1.132805 | -0.21525 | 4.254185 | 5.75E-05 | 0.000839 | 1.588932 |
| XLOC_009934 | 1.369029 | 0.576035 | 4.251482 | 5.81E-05 | 0.000844 | 1.579771 |
| XLOC_l2_013080 | -1.00647 | 0.202874 | -4.24915 | 5.86E-05 | 0.00085 | 1.571863 |
| C1orf135 | 1.219944 | -0.1125 | 4.246897 | 5.90E-05 | 0.000856 | 1.56424 |
| FOXC1 | 1.194882 | 0.251676 | 4.243046 | 5.99E-05 | 0.000863 | 1.551203 |
| OR2A25 | -1.85174 | 1.770076 | -4.23228 | 6.22E-05 | 0.000894 | 1.514794 |
| ACER3 | 1.024531 | -0.10519 | 4.230123 | 6.27E-05 | 0.000899 | 1.507512 |
| ANXA2P3 | 1.243761 | -0.13587 | 4.225123 | 6.39E-05 | 0.000913 | 1.490628 |
| ZIC5 | 1.096939 | 0.619026 | 4.222212 | 6.45E-05 | 0.000922 | 1.480805 |
| LOC100506059 | -1.24009 | 0.864929 | -4.22049 | 6.49E-05 | 0.000926 | 1.475005 |
| OR7E5P | 1.240268 | -0.41545 | 4.218517 | 6.54E-05 | 0.000931 | 1.468344 |
| XLOC_005548 | -1.05306 | 0.169504 | -4.21774 | 6.56E-05 | 0.000933 | 1.465731 |
| XLOC_002487 | -1.29823 | 0.40896 | -4.21758 | 6.56E-05 | 0.000933 | 1.465194 |
| SLC6A19 | 2.406796 | 0.625428 | 4.215529 | 6.61E-05 | 0.000939 | 1.458268 |
| XLOC_002953 | -1.01848 | 0.468881 | -4.21483 | 6.63E-05 | 0.000941 | 1.455898 |
| FAM72D | 1.094545 | -0.21827 | 4.211139 | 6.72E-05 | 0.000952 | 1.443477 |
| HOXB3 | 1.023565 | -0.10779 | 4.203593 | 6.90E-05 | 0.000971 | 1.418073 |
| DEPDC1B | 1.276279 | -0.31811 | 4.202782 | 6.92E-05 | 0.000973 | 1.415345 |
| CTTNBP2 | 1.220211 | -0.03729 | 4.200617 | 6.98E-05 | 0.000979 | 1.408063 |
| LOC100507673 | -1.03643 | 0.58755 | -4.1994 | 7.01E-05 | 0.000983 | 1.403966 |
| CARNS1 | -1.76164 | -0.2127 | -4.19242 | 7.19E-05 | 0.001003 | 1.380511 |
| HOXB2 | 1.056762 | -0.03479 | 4.191832 | 7.20E-05 | 0.001004 | 1.378537 |
| LYPD5 | 1.290812 | -0.27327 | 4.190137 | 7.25E-05 | 0.001008 | 1.372844 |
| CDCA7 | 1.140668 | -0.3119 | 4.189594 | 7.26E-05 | 0.001009 | 1.371019 |
| AZGP1 | -1.17419 | 0.26973 | -4.18305 | 7.43E-05 | 0.001027 | 1.349044 |
| FOXJ1 | 1.636136 | 0.120687 | 4.175782 | 7.63E-05 | 0.00105 | 1.324692 |
| PPARGC1A | -1.32896 | -0.14449 | -4.17543 | 7.64E-05 | 0.00105 | 1.323511 |
| FLJ39095 | 1.074059 | 0.686288 | 4.174888 | 7.65E-05 | 0.001052 | 1.321695 |
| TUBB3 | 1.448233 | -0.05925 | 4.170942 | 7.76E-05 | 0.001064 | 1.308477 |
| PLK4 | 1.087409 | -0.214 | 4.167935 | 7.85E-05 | 0.001073 | 1.298411 |
| ZNF831 | -1.06973 | -0.21061 | -4.16684 | 7.88E-05 | 0.001076 | 1.294752 |
| HSPA4L | 1.362394 | 0.090334 | 4.164048 | 7.96E-05 | 0.001084 | 1.285407 |
| C5orf38 | -1.24789 | 0.020956 | -4.1607 | 8.05E-05 | 0.001095 | 1.274207 |
| LOC100652948 | -1.0329 | 0.328192 | -4.1602 | 8.07E-05 | 0.001097 | 1.27254 |
| FABP1 | 2.929 | 0.082851 | 4.159604 | 8.09E-05 | 0.001098 | 1.270547 |
| LOC728723 | -1.08654 | 0.217094 | -4.1549 | 8.22E-05 | 0.001114 | 1.254828 |
| LOC728503 | -1.09554 | 0.19516 | -4.15199 | 8.31E-05 | 0.001124 | 1.245116 |
| RAB3B | 1.64465 | 0.035428 | 4.151345 | 8.33E-05 | 0.001125 | 1.242957 |
| MTMR11 | 1.035765 | -0.20944 | 4.149404 | 8.39E-05 | 0.001132 | 1.23648 |
| LOC93444 | -1.07956 | 0.66372 | -4.14537 | 8.51E-05 | 0.001145 | 1.223023 |
| CD19 | -2.08307 | -0.06489 | -4.13981 | 8.68E-05 | 0.001162 | 1.204486 |
| LOC388242 | 1.132317 | 0.126034 | 4.136885 | 8.77E-05 | 0.00117 | 1.194736 |
| MOCOS | 1.005944 | -0.27918 | 4.135845 | 8.80E-05 | 0.001172 | 1.191273 |
| ATXN7L2 | -1.18532 | 0.653689 | -4.13584 | 8.80E-05 | 0.001172 | 1.19126 |
| NTS | 1.831291 | 0.668582 | 4.135118 | 8.83E-05 | 0.001174 | 1.188851 |
| RDM1 | 1.024184 | -0.05538 | 4.131512 | 8.94E-05 | 0.001185 | 1.176845 |
| UGT2B7 | 1.118923 | -0.21857 | 4.127294 | 9.08E-05 | 0.001199 | 1.16281 |
| WISP3 | 1.876529 | 0.522389 | 4.123716 | 9.19E-05 | 0.001212 | 1.150912 |
| DMBT1 | 3.154665 | -1.03764 | 4.118295 | 9.37E-05 | 0.001231 | 1.132898 |
| PLA2G2A | 2.481289 | 0.490507 | 4.117978 | 9.38E-05 | 0.001231 | 1.131844 |
| XLOC_001341 | 1.241768 | 0.588457 | 4.116839 | 9.42E-05 | 0.001235 | 1.128062 |
| UNC93A | 1.377571 | 0.633233 | 4.114815 | 9.49E-05 | 0.001242 | 1.12134 |
| S1PR3 | -1.10687 | -0.21879 | -4.11442 | 9.50E-05 | 0.001243 | 1.12003 |
| KL | -1.05883 | -0.07198 | -4.11067 | 9.63E-05 | 0.001258 | 1.107583 |
| ADCY1 | -1.17822 | -0.3092 | -4.11009 | 9.65E-05 | 0.001259 | 1.105653 |
| GCNT3 | 1.47748 | -0.3285 | 4.100563 | 9.98E-05 | 0.001295 | 1.074076 |
| XLOC_007653 | -1.47742 | 0.896729 | -4.09897 | 0.0001 | 0.001301 | 1.068797 |
| BDKRB1 | 1.057134 | -0.15105 | 4.096089 | 0.000101 | 0.001311 | 1.059263 |
| FIGF | -1.2853 | -0.02881 | -4.09499 | 0.000102 | 0.001315 | 1.05563 |
| HPR | 1.373959 | 0.256793 | 4.092898 | 0.000103 | 0.001323 | 1.0487 |
| XLOC_003062 | -1.20018 | 0.791943 | -4.09224 | 0.000103 | 0.001325 | 1.046532 |
| PHOSPHO2-KLHL23 | 1.192625 | -0.00287 | 4.091988 | 0.000103 | 0.001325 | 1.045691 |
| XLOC_002664 | -1.33865 | 0.723502 | -4.08872 | 0.000104 | 0.001335 | 1.034871 |
| CDSN | 1.304453 | 0.243059 | 4.086432 | 0.000105 | 0.001344 | 1.027319 |
| IL33 | -1.24455 | 0.064762 | -4.0838 | 0.000106 | 0.001353 | 1.018604 |
| DPT | -1.4896 | -0.0218 | -4.08348 | 0.000106 | 0.001354 | 1.017559 |
| CLCA1 | 3.20206 | 0.837295 | 4.082205 | 0.000107 | 0.001358 | 1.01335 |
| PRR4 | 1.992221 | 0.530827 | 4.082018 | 0.000107 | 0.001358 | 1.012732 |
| CCDC101 | -1.09158 | 0.408496 | -4.08015 | 0.000107 | 0.001365 | 1.006566 |
| HIST1H2AG | 1.017328 | -0.16745 | 4.077865 | 0.000108 | 0.001372 | 0.999019 |
| AQP2 | 1.435594 | 0.863161 | 4.076397 | 0.000109 | 0.001377 | 0.994173 |
| ZBTB16 | -1.19621 | 0.281684 | -4.07051 | 0.000111 | 0.001401 | 0.974739 |
| KLK11 | -1.46904 | -0.19608 | -4.06823 | 0.000112 | 0.001411 | 0.967232 |
| MS4A15 | 1.543878 | 0.311973 | 4.064271 | 0.000114 | 0.001427 | 0.954196 |
| LOC100287482 | 1.181824 | 0.034931 | 4.064174 | 0.000114 | 0.001427 | 0.953876 |
| LINC00483 | 1.176939 | 0.030648 | 4.063908 | 0.000114 | 0.001427 | 0.952999 |
| ADH7 | -1.88045 | 0.09909 | -4.0631 | 0.000114 | 0.00143 | 0.950343 |
| SNORD86 | 1.026877 | -0.01908 | 4.05926 | 0.000116 | 0.001443 | 0.937697 |
| LOC100507218 | 1.558412 | 0.426344 | 4.057962 | 0.000116 | 0.001448 | 0.933425 |
| SLC17A4 | 1.311561 | 0.125429 | 4.054701 | 0.000117 | 0.001464 | 0.922696 |
| ALDH1L1 | -1.40565 | 0.315182 | -4.05354 | 0.000118 | 0.001468 | 0.918881 |
| CNTN3 | -1.13786 | -0.09445 | -4.05152 | 0.000119 | 0.001478 | 0.912235 |
| GCKR | -1.00676 | 0.018741 | -4.051 | 0.000119 | 0.001479 | 0.910517 |
| CLSPN | 1.115611 | -0.20062 | 4.049211 | 0.00012 | 0.001486 | 0.90465 |
| CEACAM5 | 1.60712 | -0.45492 | 4.047233 | 0.000121 | 0.001495 | 0.89815 |
| PPBP | 1.613541 | 0.853767 | 4.045721 | 0.000121 | 0.001498 | 0.893183 |
| NKX6-3 | 1.532948 | -0.33548 | 4.045306 | 0.000121 | 0.0015 | 0.891822 |
| HIST1H2BF | 1.08518 | -0.22802 | 4.042271 | 0.000123 | 0.00151 | 0.881858 |
| XLOC_008005 | 1.154834 | 0.019984 | 4.037138 | 0.000125 | 0.001532 | 0.865014 |
| ARSE | 1.105048 | 0.166552 | 4.036543 | 0.000125 | 0.001534 | 0.863064 |
| BAIAP2L2 | 1.301489 | -0.18009 | 4.033035 | 0.000127 | 0.001548 | 0.851562 |
| KRTAP5-7 | -1.46457 | 0.355601 | -4.03303 | 0.000127 | 0.001548 | 0.851538 |
| ARSJ | 1.004502 | 0.24758 | 4.031388 | 0.000128 | 0.001555 | 0.846165 |
| ACSM2B | -1.46112 | 0.132537 | -4.03117 | 0.000128 | 0.001556 | 0.845447 |
| NIM1 | -1.32886 | -0.46293 | -4.03075 | 0.000128 | 0.001558 | 0.844071 |
| RAD51 | 1.026668 | -0.12445 | 4.02231 | 0.000132 | 0.001601 | 0.816442 |
| FAM54A | 1.046482 | 0.020823 | 4.021759 | 0.000132 | 0.001603 | 0.814639 |
| MAF | -1.05424 | -0.30983 | -4.02037 | 0.000133 | 0.001609 | 0.810084 |
| TESC | -1.0809 | -0.33842 | -4.01932 | 0.000133 | 0.001612 | 0.806669 |
| PCDHA7 | -1.79523 | 0.993491 | -4.01865 | 0.000133 | 0.001615 | 0.804471 |
| KIAA1211 | 1.277848 | -0.07047 | 4.017934 | 0.000134 | 0.001618 | 0.802128 |
| MEP1A | 2.920485 | 1.069563 | 4.013065 | 0.000136 | 0.001641 | 0.786215 |
| FGF13 | -1.19067 | 0.086927 | -4.0008 | 0.000142 | 0.001691 | 0.746183 |
| CYP2C8 | -1.42927 | 0.835147 | -4.00011 | 0.000142 | 0.001693 | 0.743932 |
| PLTP | -1.0769 | 0.063915 | -3.99725 | 0.000144 | 0.001703 | 0.734612 |
| CSMD3 | -1.15925 | 0.851221 | -3.99184 | 0.000147 | 0.001731 | 0.716993 |
| PP12613 | -1.22279 | 0.942336 | -3.98604 | 0.00015 | 0.00176 | 0.69812 |
| PSPN | -1.01365 | 0.462953 | -3.9858 | 0.00015 | 0.001761 | 0.697347 |
| SBK2 | -1.16403 | 0.441824 | -3.98206 | 0.000152 | 0.001776 | 0.685169 |
| ABTB2 | 1.045419 | -0.24849 | 3.981465 | 0.000152 | 0.001779 | 0.683241 |
| BANK1 | 1.501061 | -0.52707 | 3.977524 | 0.000154 | 0.001799 | 0.670437 |
| IRAK3 | -1.16365 | -0.32782 | -3.97741 | 0.000154 | 0.001799 | 0.670053 |
| RNASE1 | -1.03835 | -0.19812 | -3.9767 | 0.000154 | 0.001801 | 0.667769 |
| FAM131B | 1.057294 | 0.618358 | 3.976066 | 0.000155 | 0.001803 | 0.665703 |
| WNT6 | -1.11986 | 0.170483 | -3.9694 | 0.000158 | 0.001836 | 0.644071 |
| IRF4 | -1.70719 | -0.19326 | -3.96486 | 0.000161 | 0.001862 | 0.629352 |
| RAD54L | 1.056268 | -0.32085 | 3.96312 | 0.000162 | 0.001872 | 0.623706 |
| E2F2 | 1.017199 | -0.41903 | 3.960282 | 0.000164 | 0.001885 | 0.614511 |
| C12orf28 | 1.408717 | 0.301622 | 3.958508 | 0.000165 | 0.001894 | 0.608767 |
| SKA1 | 1.22222 | -0.21913 | 3.955793 | 0.000166 | 0.001911 | 0.599979 |
| ADRA2A | 1.560753 | -0.37286 | 3.955414 | 0.000166 | 0.001912 | 0.59875 |
| KLHL6 | -1.1528 | -0.20406 | -3.95481 | 0.000167 | 0.001916 | 0.596805 |
| SNORA5B | -1.65235 | 0.644097 | -3.95238 | 0.000168 | 0.001929 | 0.588934 |
| MNS1 | 1.167691 | -0.17223 | 3.951253 | 0.000169 | 0.001935 | 0.58529 |
| NUP210 | -1.10334 | -0.357 | -3.94631 | 0.000172 | 0.001962 | 0.569297 |
| FAM127C | -1.04995 | -0.32493 | -3.93786 | 0.000177 | 0.002007 | 0.542017 |
| RAPSN | -1.17456 | 0.611644 | -3.93265 | 0.00018 | 0.002033 | 0.525224 |
| ST8SIA5 | -1.13914 | 0.324262 | -3.93133 | 0.000181 | 0.002039 | 0.520948 |
| HOXB6 | 1.359734 | 0.040869 | 3.928498 | 0.000183 | 0.002055 | 0.511839 |
| XLOC_004607 | -1.57495 | 0.127854 | -3.92653 | 0.000184 | 0.002068 | 0.505504 |
| AGMO | 2.434164 | -0.00382 | 3.923125 | 0.000186 | 0.002088 | 0.494535 |
| LOC100506591 | 1.467255 | 0.995136 | 3.919788 | 0.000188 | 0.002106 | 0.483797 |
| XLOC_004600 | -1.45443 | 0.165115 | -3.91775 | 0.00019 | 0.002117 | 0.477255 |
| BEX1 | -1.70996 | -0.53094 | -3.91696 | 0.00019 | 0.002121 | 0.474687 |
| XLOC_l2_011908 | -1.04111 | 0.674639 | -3.91659 | 0.00019 | 0.002122 | 0.473498 |
| GFRA4 | -1.39106 | 0.513341 | -3.91361 | 0.000192 | 0.002138 | 0.463943 |
| SYNDIG1 | -1.50999 | -0.51358 | -3.91245 | 0.000193 | 0.002143 | 0.460211 |
| LOC100129840 | -1.26474 | 0.846082 | -3.91195 | 0.000193 | 0.002146 | 0.458608 |
| DUOX2 | -1.56585 | -0.25285 | -3.91029 | 0.000195 | 0.002158 | 0.45326 |
| SLC4A7 | 1.324953 | 0.054643 | 3.908895 | 0.000196 | 0.002165 | 0.448785 |
| SNORA74A | 1.35245 | 0.11646 | 3.906858 | 0.000197 | 0.002176 | 0.442244 |
| SH3D21 | 1.242109 | 0.115941 | 3.905256 | 0.000198 | 0.002184 | 0.437103 |
| ADRB2 | -1.29819 | -0.22255 | -3.90196 | 0.0002 | 0.002205 | 0.426528 |
| FABP6 | 1.618724 | 0.321162 | 3.893949 | 0.000206 | 0.002256 | 0.400855 |
| GRB14 | 1.16076 | 0.260294 | 3.893639 | 0.000206 | 0.002257 | 0.399861 |
| PDIA2 | -1.66772 | 0.016199 | -3.89131 | 0.000208 | 0.00227 | 0.392409 |
| OPN4 | -1.12965 | 0.314637 | -3.89002 | 0.000209 | 0.002277 | 0.388287 |
| TMEM158 | 1.147312 | -0.18888 | 3.886406 | 0.000211 | 0.0023 | 0.376709 |
| LOC200261 | -1.10659 | 0.42491 | -3.88572 | 0.000212 | 0.002303 | 0.37452 |
| CENPA | 1.214575 | -0.30084 | 3.884279 | 0.000213 | 0.00231 | 0.369908 |
| ABCG5 | 1.756616 | -0.09748 | 3.8828 | 0.000214 | 0.002318 | 0.365178 |
| MUC12 | 1.015579 | 0.589517 | 3.882405 | 0.000214 | 0.002319 | 0.363916 |
| SGK1 | -1.03725 | 0.085555 | -3.87845 | 0.000217 | 0.002341 | 0.351278 |
| OIP5 | 1.014863 | -0.1976 | 3.875437 | 0.000219 | 0.002362 | 0.341656 |
| LOC100506662 | -1.09921 | -0.05116 | -3.87498 | 0.00022 | 0.002365 | 0.340187 |
| SPOCK1 | -1.05089 | 0.431278 | -3.87459 | 0.00022 | 0.002367 | 0.338948 |
| MAD2L1 | 1.054738 | -0.17242 | 3.872425 | 0.000222 | 0.002379 | 0.332039 |
| SNAR-A3 | 1.401197 | 0.261451 | 3.871646 | 0.000222 | 0.002384 | 0.329554 |
| KIFC1 | 1.057664 | -0.24344 | 3.869869 | 0.000224 | 0.002395 | 0.323887 |
| XLOC_010411 | -1.39884 | 0.402149 | -3.86963 | 0.000224 | 0.002396 | 0.32311 |
| SNAR-H | 1.319566 | 0.213405 | 3.863673 | 0.000229 | 0.002433 | 0.304133 |
| CDC6 | 1.104159 | -0.12533 | 3.862076 | 0.00023 | 0.002441 | 0.299045 |
| SI | 3.094411 | 0.28084 | 3.861832 | 0.00023 | 0.002442 | 0.29827 |
| ANPEP | 3.14436 | 1.022396 | 3.861769 | 0.00023 | 0.002442 | 0.298067 |
| CEP55 | 1.081765 | -0.0565 | 3.860167 | 0.000231 | 0.002451 | 0.292965 |
| XLOC_007535 | -1.31374 | 0.913929 | -3.85945 | 0.000232 | 0.002455 | 0.290689 |
| GRIA3 | -1.00477 | 0.372147 | -3.85597 | 0.000235 | 0.002478 | 0.279614 |
| CCR10 | -1.11884 | 0.052124 | -3.85379 | 0.000236 | 0.002493 | 0.272682 |
| XLOC_004283 | -1.08732 | 0.422294 | -3.85102 | 0.000239 | 0.002511 | 0.263866 |
| LOC283663 | 1.104113 | -0.11768 | 3.84852 | 0.000241 | 0.002531 | 0.255916 |
| XLOC_007123 | 1.467636 | 0.015153 | 3.838211 | 0.000249 | 0.002604 | 0.223185 |
| ATP13A4 | -1.02827 | -0.19831 | -3.83656 | 0.000251 | 0.002613 | 0.217939 |
| LDLRAD2 | -1.13402 | -0.17444 | -3.836 | 0.000251 | 0.002617 | 0.216186 |
| LOC283075 | -1.07091 | 0.33256 | -3.82738 | 0.000259 | 0.002682 | 0.188858 |
| AQP12A | 1.020844 | 0.826924 | 3.823057 | 0.000263 | 0.002708 | 0.175178 |
| HPX-2 | -1.20244 | 0.210726 | -3.81989 | 0.000266 | 0.00273 | 0.165153 |
| TNFRSF13C | -1.08933 | 0.12865 | -3.81762 | 0.000268 | 0.002747 | 0.157997 |
| PRKCB | -1.03868 | 0.306682 | -3.80305 | 0.000281 | 0.002858 | 0.112001 |
| SEMG1 | 1.429767 | -0.01034 | 3.797428 | 0.000287 | 0.0029 | 0.094281 |
| GZMK | -1.04794 | -0.05005 | -3.79366 | 0.00029 | 0.002931 | 0.082425 |
| MGC16025 | -1.38012 | 0.02711 | -3.79312 | 0.000291 | 0.002936 | 0.080718 |
| PBK | 1.13836 | -0.11663 | 3.791276 | 0.000293 | 0.002951 | 0.074916 |
| CFC1 | -1.16958 | 0.653539 | -3.79058 | 0.000293 | 0.002955 | 0.072719 |
| TNS4 | 1.527032 | 0.047089 | 3.786989 | 0.000297 | 0.002987 | 0.061434 |
| SPINK13 | -2.03169 | -0.13544 | -3.7831 | 0.000301 | 0.003018 | 0.049209 |
| CCNA2 | 1.092726 | -0.13149 | 3.780977 | 0.000303 | 0.003033 | 0.042547 |
| MEI1 | -1.06153 | 0.180131 | -3.77584 | 0.000309 | 0.00308 | 0.026422 |
| PNOC | -1.64653 | -0.07167 | -3.77386 | 0.000311 | 0.003099 | 0.020214 |
| SLC22A10 | -1.08231 | 0.192905 | -3.76501 | 0.00032 | 0.00317 | -0.00753 |
| LOC254057 | 1.624888 | 0.508953 | 3.758814 | 0.000327 | 0.003227 | -0.0269 |
| HTR2B | 1.419127 | -0.08658 | 3.758484 | 0.000327 | 0.003229 | -0.02794 |
| PF4 | 1.481706 | 0.067743 | 3.75834 | 0.000327 | 0.00323 | -0.02839 |
| TRIM31 | 1.086678 | -0.21481 | 3.757832 | 0.000328 | 0.00323 | -0.02998 |
| MLIP | 1.296848 | 0.892396 | 3.757732 | 0.000328 | 0.00323 | -0.03029 |
| LRP8 | 1.403528 | 0.039884 | 3.756503 | 0.000329 | 0.003239 | -0.03413 |
| DMRTC1 | -1.08994 | -0.15918 | -3.75526 | 0.000331 | 0.003251 | -0.038 |
| GAS1 | -1.05494 | -0.1748 | -3.75307 | 0.000333 | 0.003269 | -0.04487 |
| XLOC_006193 | -1.36257 | 0.469118 | -3.7499 | 0.000337 | 0.0033 | -0.05477 |
| ANLN | 1.191835 | -0.16189 | 3.74587 | 0.000341 | 0.003332 | -0.06734 |
| HSD17B2 | 1.03963 | 0.1816 | 3.741995 | 0.000346 | 0.003367 | -0.07942 |
| SERPINB8 | 1.039774 | -0.40695 | 3.741828 | 0.000346 | 0.003368 | -0.07994 |
| TNFRSF8 | -1.03056 | -0.13627 | -3.74169 | 0.000346 | 0.003368 | -0.08036 |
| LOC100505727 | 1.151282 | -0.04444 | 3.740118 | 0.000348 | 0.003382 | -0.08527 |
| QPCT | 1.069694 | -0.23001 | 3.73556 | 0.000354 | 0.003423 | -0.09947 |
| C11orf85 | -1.48911 | 0.845569 | -3.7344 | 0.000355 | 0.003432 | -0.10308 |
| CHGA | -1.17573 | -0.2614 | -3.73024 | 0.00036 | 0.00347 | -0.11602 |
| XLOC_009945 | -1.00387 | 0.249854 | -3.72208 | 0.00037 | 0.003551 | -0.14141 |
| FOSB | 1.807226 | 0.218071 | 3.721581 | 0.000371 | 0.003553 | -0.14295 |
| TNFRSF17 | -1.77055 | -0.20271 | -3.71341 | 0.000381 | 0.00363 | -0.1683 |
| XDH | 1.697349 | -0.69872 | 3.712494 | 0.000382 | 0.003634 | -0.17115 |
| XLOC_012977 | 1.063969 | -0.17079 | 3.708509 | 0.000387 | 0.003671 | -0.1835 |
| SLC35F3 | -1.38763 | -0.25392 | -3.70831 | 0.000387 | 0.003673 | -0.18412 |
| LOC100507334 | -1.14146 | 0.062829 | -3.68844 | 0.000414 | 0.003868 | -0.24557 |
| LOC100287415 | 1.070307 | -0.14983 | 3.685584 | 0.000418 | 0.0039 | -0.25438 |
| HLA-DOB | -1.20239 | 0.038333 | -3.68079 | 0.000425 | 0.003949 | -0.26916 |
| XLOC_002870 | 1.190161 | 0.175654 | 3.676648 | 0.000431 | 0.00399 | -0.28193 |
| TUBAL3 | 1.693473 | 0.111393 | 3.676553 | 0.000431 | 0.00399 | -0.28222 |
| LOC100506123 | -1.21524 | 0.151543 | -3.67506 | 0.000433 | 0.004001 | -0.28681 |
| GATA5 | -1.30962 | -0.51439 | -3.67294 | 0.000436 | 0.00402 | -0.29335 |
| XLOC_014068 | -1.00942 | -0.05584 | -3.67218 | 0.000437 | 0.004026 | -0.29568 |
| CA4 | -1.14904 | -0.14445 | -3.67095 | 0.000439 | 0.00404 | -0.29945 |
| DBN1 | -1.01484 | 0.165541 | -3.66705 | 0.000445 | 0.004086 | -0.31147 |
| SLITRK4 | -1.13826 | 0.092357 | -3.66266 | 0.000451 | 0.004132 | -0.32495 |
| CALHM3 | 1.724448 | 0.093807 | 3.661085 | 0.000454 | 0.004149 | -0.32979 |
| C1orf130 | -1.10883 | 0.061676 | -3.66015 | 0.000455 | 0.004158 | -0.33265 |
| SNAR-D | 1.266218 | 0.323627 | 3.657458 | 0.000459 | 0.004188 | -0.34092 |
| JAKMIP1 | -1.0131 | -0.01546 | -3.6555 | 0.000462 | 0.004212 | -0.34694 |
| SPC25 | 1.139547 | -0.15405 | 3.654838 | 0.000463 | 0.004218 | -0.34896 |
| CLECL1 | -1.22162 | -0.16173 | -3.65336 | 0.000466 | 0.004235 | -0.35349 |
| CENPK | 1.021736 | -0.15421 | 3.644373 | 0.00048 | 0.004329 | -0.38103 |
| TCN1 | -1.70797 | -0.1577 | -3.64099 | 0.000485 | 0.004368 | -0.39137 |
| XLOC_008102 | -1.32711 | 0.458096 | -3.63756 | 0.000491 | 0.004409 | -0.40187 |
| FAM72A | 1.123682 | -0.15635 | 3.637309 | 0.000491 | 0.004411 | -0.40264 |
| RRM2 | 1.288662 | -0.4785 | 3.635985 | 0.000493 | 0.004428 | -0.40668 |
| GPD1 | 1.176894 | 0.190117 | 3.634584 | 0.000495 | 0.004441 | -0.41096 |
| FOLH1 | 1.43793 | 0.460352 | 3.633925 | 0.000497 | 0.004445 | -0.41298 |
| CKAP2L | 1.071427 | -0.25849 | 3.628986 | 0.000505 | 0.004499 | -0.42806 |
| SNAR-G2 | 1.262942 | 0.103022 | 3.628858 | 0.000505 | 0.004499 | -0.42845 |
| MYBL2 | 1.062381 | -0.10815 | 3.625464 | 0.000511 | 0.004539 | -0.4388 |
| GPX3 | -1.07384 | 0.019167 | -3.61818 | 0.000523 | 0.004623 | -0.46099 |
| S100A3 | 1.270018 | 0.401996 | 3.610815 | 0.000536 | 0.004709 | -0.48342 |
| AFAP1-AS1 | 1.859023 | -0.60918 | 3.607942 | 0.000541 | 0.004745 | -0.49215 |
| SOX21 | -1.57292 | -0.50383 | -3.60297 | 0.00055 | 0.004809 | -0.50726 |
| KLHL13 | 1.141583 | -0.05929 | 3.598245 | 0.000559 | 0.00487 | -0.5216 |
| GKN2 | -1.32236 | -0.54759 | -3.58589 | 0.000582 | 0.005014 | -0.55903 |
| TRIM7 | 1.115197 | -0.1596 | 3.583783 | 0.000586 | 0.005042 | -0.56541 |
| MAL | -1.75191 | -0.78332 | -3.58078 | 0.000592 | 0.005088 | -0.57448 |
| RNF186 | 1.394442 | 0.397815 | 3.579737 | 0.000594 | 0.005102 | -0.57764 |
| PLIN4 | -1.46883 | -0.08523 | -3.57138 | 0.00061 | 0.005226 | -0.60286 |
| AMHR2 | -1.03281 | 0.17828 | -3.56473 | 0.000624 | 0.00531 | -0.62294 |
| BST1 | 1.139579 | -0.13891 | 3.564141 | 0.000625 | 0.005316 | -0.62471 |
| NOL4 | 1.136207 | 0.952133 | 3.553531 | 0.000647 | 0.00547 | -0.65664 |
| C16orf89 | -1.48601 | -0.28765 | -3.54923 | 0.000656 | 0.005532 | -0.66956 |
| PRAC | 1.631902 | 0.690194 | 3.543411 | 0.000669 | 0.005613 | -0.68704 |
| XLOC_011023 | -1.08634 | 1.132912 | -3.54311 | 0.000669 | 0.005616 | -0.68794 |
| FGF20 | -1.16787 | 0.373735 | -3.53907 | 0.000678 | 0.005669 | -0.70007 |
| XLOC_002073 | -1.2672 | 0.565592 | -3.53232 | 0.000693 | 0.005763 | -0.72028 |
| XLOC_003528 | 1.471949 | 0.447157 | 3.530328 | 0.000698 | 0.005791 | -0.72625 |
| OR56B1 | -1.12488 | 0.831736 | -3.52672 | 0.000706 | 0.005842 | -0.73704 |
| REG1B | 2.037177 | 1.449361 | 3.525351 | 0.000709 | 0.005859 | -0.74113 |
| ZG16 | 2.821468 | 0.828791 | 3.520249 | 0.000721 | 0.005934 | -0.75638 |
| CNIH3 | -1.27368 | 0.257468 | -3.51546 | 0.000733 | 0.006005 | -0.77068 |
| P2RX6P | 1.022813 | 0.875176 | 3.511171 | 0.000743 | 0.006083 | -0.78346 |
| FAM25A | 1.378339 | 0.358619 | 3.507587 | 0.000752 | 0.006142 | -0.79415 |
| C3 | -1.47029 | -0.20246 | -3.50535 | 0.000757 | 0.006171 | -0.80082 |
| CELA2A | -1.67613 | 1.12946 | -3.50345 | 0.000762 | 0.006198 | -0.80646 |
| CCDC68 | 1.033803 | 0.037395 | 3.502652 | 0.000764 | 0.006209 | -0.80884 |
| FCRLA | -1.14215 | 0.376449 | -3.49699 | 0.000778 | 0.006308 | -0.82568 |
| DKK1 | -1.35191 | -0.34555 | -3.49462 | 0.000784 | 0.006349 | -0.83271 |
| CD79B | -1.12316 | 0.128632 | -3.49398 | 0.000785 | 0.006361 | -0.83463 |
| APOA5 | -1.18889 | 0.280971 | -3.4876 | 0.000802 | 0.006465 | -0.85355 |
| KLK12 | 1.634915 | 0.3007 | 3.485282 | 0.000808 | 0.006508 | -0.86044 |
| LY6D | 2.699891 | 1.403355 | 3.484503 | 0.00081 | 0.006519 | -0.86275 |
| BTNL3 | 3.261827 | -1.53452 | 3.483753 | 0.000812 | 0.006527 | -0.86497 |
| KREMEN2 | 1.042343 | 0.031487 | 3.479674 | 0.000823 | 0.006598 | -0.87706 |
| SHISA3 | 1.529001 | 0.67895 | 3.470592 | 0.000847 | 0.006761 | -0.90393 |
| ANXA8L2 | 1.06875 | -0.00142 | 3.468629 | 0.000852 | 0.006797 | -0.90973 |
| SLC5A5 | -1.1985 | -0.0684 | -3.46739 | 0.000856 | 0.006819 | -0.9134 |
| LOC284865 | 1.316453 | 1.036027 | 3.466866 | 0.000857 | 0.006827 | -0.91494 |
| RGS11 | -1.00092 | 0.24477 | -3.46192 | 0.000871 | 0.006923 | -0.92953 |
| ADAMTS19 | -1.0185 | 0.310975 | -3.46077 | 0.000874 | 0.006945 | -0.93295 |
| NLRP7 | -1.70007 | 0.254512 | -3.45965 | 0.000877 | 0.006963 | -0.93623 |
| WBSCR27 | 1.581287 | -0.38318 | 3.455495 | 0.000889 | 0.007036 | -0.94849 |
| MUC13 | 1.328174 | -0.28628 | 3.454459 | 0.000892 | 0.007054 | -0.95154 |
| TM6SF2 | 1.094681 | 0.108721 | 3.452345 | 0.000898 | 0.007084 | -0.95776 |
| NEFL | -1.01059 | 0.276041 | -3.44871 | 0.000909 | 0.007154 | -0.96846 |
| CDA | 1.261637 | 0.077287 | 3.447631 | 0.000912 | 0.007173 | -0.97164 |
| MS4A1 | -1.38062 | 0.796236 | -3.43958 | 0.000936 | 0.007317 | -0.99529 |
| WISP2 | -1.21285 | 0.04057 | -3.43862 | 0.000939 | 0.007337 | -0.99813 |
| TCTN2 | -1.20215 | 0.240187 | -3.43549 | 0.000948 | 0.007386 | -1.00731 |
| F5 | 1.292576 | -0.29622 | 3.434516 | 0.000951 | 0.007402 | -1.01017 |
| GAL | 1.027879 | -0.02232 | 3.43084 | 0.000962 | 0.007475 | -1.02095 |
| INSL5 | 1.235801 | 0.708629 | 3.428276 | 0.00097 | 0.00753 | -1.02846 |
| LIPG | 1.07021 | -0.12263 | 3.421485 | 0.000992 | 0.007665 | -1.04834 |
| XLOC_001990 | -1.60175 | 1.254575 | -3.41915 | 0.000999 | 0.007706 | -1.05518 |
| DEFA6 | 2.733669 | 2.911372 | 3.407873 | 0.001036 | 0.007927 | -1.08811 |
| GSTA5 | -1.03876 | -0.07286 | -3.40466 | 0.001046 | 0.007993 | -1.09746 |
| BTBD16 | 1.531333 | -0.10271 | 3.402259 | 0.001054 | 0.008041 | -1.10447 |
| CA1 | 2.452435 | 1.000312 | 3.397843 | 0.001069 | 0.008128 | -1.11733 |
| SNAR-B2 | 1.173919 | 0.231272 | 3.387786 | 0.001104 | 0.008337 | -1.14657 |
| CYP3A4 | 1.617004 | 0.311929 | 3.384098 | 0.001117 | 0.00842 | -1.15728 |
| OPN1MW | -1.12113 | 0.425071 | -3.38251 | 0.001123 | 0.008456 | -1.16189 |
| NRTN | -1.1335 | 0.084067 | -3.38046 | 0.00113 | 0.008496 | -1.16784 |
| GSG2 | 1.005747 | 0.133782 | 3.379877 | 0.001132 | 0.008505 | -1.16952 |
| CDHR2 | 1.088044 | 0.264672 | 3.367916 | 0.001176 | 0.008762 | -1.20416 |
| NEUROD2 | 1.295274 | 0.386127 | 3.363975 | 0.001191 | 0.008851 | -1.21555 |
| P2RY2 | 1.007921 | 0.003049 | 3.363587 | 0.001192 | 0.008856 | -1.21667 |
| C4orf7 | -2.52056 | 1.068996 | -3.35889 | 0.00121 | 0.008968 | -1.23023 |
| PITX2 | 1.118535 | 0.675451 | 3.354043 | 0.001229 | 0.009081 | -1.24421 |
| XLOC_014263 | 1.030322 | 0.378244 | 3.352738 | 0.001234 | 0.00911 | -1.24797 |
| LDHC | 1.046055 | 0.206719 | 3.3396 | 0.001286 | 0.00943 | -1.28578 |
| BCMO1 | 1.027733 | -0.00172 | 3.338743 | 0.001289 | 0.009437 | -1.28824 |
| SLC6A20 | 1.118316 | -0.09605 | 3.335641 | 0.001302 | 0.009509 | -1.29716 |
| CR2 | -1.32833 | 0.694652 | -3.33409 | 0.001308 | 0.00954 | -1.3016 |
| GALNT8 | 1.12708 | 0.528823 | 3.332118 | 0.001316 | 0.009586 | -1.30726 |
| CELA2B | -1.35475 | 0.531354 | -3.32707 | 0.001337 | 0.009711 | -1.32174 |
| C3orf32 | 1.198104 | 0.413538 | 3.3166 | 0.001382 | 0.009968 | -1.35171 |
| OR51E1 | 1.169645 | 0.371189 | 3.315995 | 0.001385 | 0.009984 | -1.35344 |
| GPR153 | 1.054378 | -0.20202 | 3.314916 | 0.001389 | 0.010014 | -1.35652 |
| LOC401847 | -1.07949 | -0.02815 | -3.3148 | 0.00139 | 0.010014 | -1.35685 |
| MCM10 | 1.029512 | -0.02287 | 3.310389 | 0.001409 | 0.010127 | -1.36945 |
| IL11 | 1.035613 | 0.598806 | 3.303577 | 0.00144 | 0.010301 | -1.38888 |
| C20orf85 | 1.442833 | 1.086786 | 3.297944 | 0.001465 | 0.010454 | -1.40493 |
| C2CD4A | 1.147424 | -0.13584 | 3.293044 | 0.001488 | 0.01058 | -1.41887 |
| VPREB3 | -1.16158 | 0.401187 | -3.28443 | 0.001528 | 0.010802 | -1.44334 |
| XLOC_l2_006138 | -1.127 | 1.134334 | -3.28147 | 0.001542 | 0.010889 | -1.45175 |
| FCRL2 | -1.02277 | 0.213032 | -3.27269 | 0.001585 | 0.011097 | -1.4766 |
| SLC7A4 | 1.165096 | -0.5249 | 3.26815 | 0.001608 | 0.011223 | -1.48946 |
| SCNN1G | -1.57882 | 0.771991 | -3.26654 | 0.001616 | 0.011264 | -1.49401 |
| OR4D11 | -1.05665 | 0.866365 | -3.26543 | 0.001621 | 0.011284 | -1.49716 |
| KHK | 1.021261 | 0.166978 | 3.262714 | 0.001635 | 0.01135 | -1.50482 |
| GC | -1.28458 | 0.059336 | -3.26156 | 0.001641 | 0.011376 | -1.50807 |
| XLOC_006689 | -1.25685 | 0.294785 | -3.25925 | 0.001653 | 0.011438 | -1.51461 |
| GCG | 1.850769 | 0.47999 | 3.255166 | 0.001674 | 0.011559 | -1.52612 |
| CXCR5 | -1.44459 | 0.319374 | -3.25467 | 0.001676 | 0.011572 | -1.52752 |
| C11orf82 | 1.020722 | -0.2297 | 3.254103 | 0.001679 | 0.011584 | -1.52911 |
| MMP3 | 1.754115 | 0.165718 | 3.249719 | 0.001702 | 0.011718 | -1.54147 |
| NPSR1 | 1.160846 | 0.588596 | 3.248538 | 0.001708 | 0.01175 | -1.54479 |
| FLVCR2 | 1.060567 | 0.990167 | 3.243985 | 0.001733 | 0.011876 | -1.5576 |
| LOC100288273 | -1.58513 | -0.39636 | -3.22201 | 0.001854 | 0.012495 | -1.61921 |
| GDPD2 | 1.043288 | 0.307748 | 3.219544 | 0.001868 | 0.01257 | -1.62612 |
| CYP4F22 | -1.25105 | 0.745457 | -3.21824 | 0.001876 | 0.01261 | -1.62978 |
| SHCBP1 | 1.081885 | -0.27587 | 3.214222 | 0.001899 | 0.012737 | -1.64099 |
| YBX2 | 1.084267 | 0.042658 | 3.211236 | 0.001917 | 0.012836 | -1.64933 |
| CXCL13 | -1.77339 | 0.575124 | -3.2076 | 0.001938 | 0.012947 | -1.65948 |
| SNAR-F | 1.121556 | -0.02354 | 3.203222 | 0.001964 | 0.013073 | -1.67166 |
| LTF | -1.83614 | 0.044514 | -3.18553 | 0.002074 | 0.013617 | -1.72082 |
| FOLH1B | 1.698063 | 0.230023 | 3.182941 | 0.00209 | 0.013678 | -1.72801 |
| CCK | 1.418273 | -0.2093 | 3.178416 | 0.002119 | 0.013823 | -1.74054 |
| AIM2 | -1.19317 | -0.21627 | -3.17803 | 0.002122 | 0.013837 | -1.74162 |
| XLOC_000371 | 1.414601 | 1.181714 | 3.177543 | 0.002125 | 0.013854 | -1.74296 |
| PCK1 | 1.941678 | 0.203584 | 3.176957 | 0.002129 | 0.013871 | -1.74458 |
| DAND5 | -1.09247 | 0.252237 | -3.17391 | 0.002149 | 0.013977 | -1.75301 |
| OTOP3 | 1.391833 | 0.818358 | 3.170463 | 0.002171 | 0.014099 | -1.76254 |
| XLOC_001066 | -1.02388 | 0.44631 | -3.1686 | 0.002184 | 0.014165 | -1.76768 |
| C21orf90 | 1.367323 | -0.2533 | 3.150174 | 0.002309 | 0.014813 | -1.81846 |
| APOBEC1 | 1.38144 | -0.53657 | 3.136337 | 0.002408 | 0.015296 | -1.85644 |
| SLC6A14 | 1.651828 | -0.55529 | 3.11938 | 0.002535 | 0.01591 | -1.90281 |
| XLOC_012083 | -1.03088 | 0.496669 | -3.11038 | 0.002605 | 0.016226 | -1.92734 |
| KIF1A | -1.23369 | -0.40865 | -3.09722 | 0.00271 | 0.016754 | -1.9631 |
| TNNC1 | 1.160866 | 0.100879 | 3.087122 | 0.002793 | 0.017186 | -1.99048 |
| SPAG17 | 1.354242 | 1.267081 | 3.073564 | 0.002909 | 0.017738 | -2.02711 |
| LOC388780 | 1.117922 | -0.10514 | 3.069751 | 0.002942 | 0.017885 | -2.03739 |
| LOC100506957 | 1.109755 | 0.167011 | 3.054396 | 0.00308 | 0.018527 | -2.07869 |
| GUCA2A | 1.468225 | 0.721332 | 3.04491 | 0.003168 | 0.01895 | -2.10413 |
| CCL19 | -1.44352 | 0.173161 | -3.04283 | 0.003187 | 0.019022 | -2.10971 |
| SLC26A3 | 2.082894 | 1.932916 | 3.038363 | 0.00323 | 0.019247 | -2.12165 |
| GSTA2 | -1.00904 | -0.13279 | -3.03381 | 0.003274 | 0.019449 | -2.1338 |
| CASP14L | 1.285262 | 1.27879 | 3.020651 | 0.003404 | 0.020029 | -2.16889 |
| LPL | -1.04013 | -0.21731 | -3.014 | 0.003471 | 0.0203 | -2.18656 |
| CCL25 | 2.126058 | 2.063623 | 3.002175 | 0.003594 | 0.020871 | -2.21794 |
| BEST4 | 1.408067 | 0.70171 | 2.98865 | 0.00374 | 0.021565 | -2.2537 |
| FST | -1.15173 | -0.06877 | -2.97342 | 0.00391 | 0.02234 | -2.2938 |
| LOC651536 | -1.01027 | 0.02924 | -2.97153 | 0.003932 | 0.022428 | -2.29878 |
| CHI3L2 | -1.02653 | 0.733135 | -2.96829 | 0.003969 | 0.022557 | -2.30729 |
| PTPRVP | 1.241162 | 0.332017 | 2.964986 | 0.004008 | 0.022711 | -2.31595 |
| KCNMB2 | -1.16623 | -0.24159 | -2.96129 | 0.004051 | 0.022916 | -2.32563 |
| ACE2 | 1.36998 | 0.306094 | 2.930099 | 0.004436 | 0.024608 | -2.40701 |
| VGF | 1.191503 | 0.180049 | 2.903728 | 0.004787 | 0.026149 | -2.47527 |
| FABP2 | 1.406497 | 1.307368 | 2.895949 | 0.004895 | 0.026619 | -2.49531 |
| CYP1A1 | 1.267546 | 0.300042 | 2.890778 | 0.004968 | 0.026854 | -2.50861 |
| FAM155B | -1.10624 | -0.40705 | -2.88759 | 0.005014 | 0.027036 | -2.51679 |
| XLOC_008925 | -1.11969 | 0.999689 | -2.88585 | 0.005039 | 0.027121 | -2.52127 |
| RNF152 | -1.16759 | -0.35744 | -2.87464 | 0.005204 | 0.027806 | -2.55 |
| TDRD9 | -1.19988 | 0.323878 | -2.8708 | 0.005261 | 0.027991 | -2.5598 |
| SCNN1B | -1.53557 | -0.25471 | -2.86807 | 0.005302 | 0.028145 | -2.56678 |
| APOH | -1.32324 | -0.03403 | -2.86736 | 0.005313 | 0.028188 | -2.5686 |
| KRT16 | 1.050108 | 0.136563 | 2.860584 | 0.005417 | 0.028658 | -2.58588 |
| SCG2 | 1.07631 | -0.0725 | 2.860492 | 0.005418 | 0.028661 | -2.58611 |
| LCN15 | 1.065489 | 0.971209 | 2.859654 | 0.005431 | 0.02872 | -2.58825 |
| PCSK9 | 1.492418 | 0.09954 | 2.85238 | 0.005545 | 0.029177 | -2.60676 |
| XLOC_008207 | -1.37861 | 0.856913 | -2.84765 | 0.00562 | 0.029495 | -2.61879 |
| GPR172B | 1.014146 | 0.354199 | 2.84247 | 0.005703 | 0.029849 | -2.63192 |
| MESP1 | 1.356321 | -0.30899 | 2.823229 | 0.006023 | 0.031171 | -2.68057 |
| XLOC_006793 | -1.10479 | 0.478821 | -2.8229 | 0.006028 | 0.031195 | -2.68141 |
| XLOC_010319 | -1.158 | 0.244746 | -2.79869 | 0.006454 | 0.032915 | -2.74224 |
| SLC35G1 | 1.011323 | 0.168023 | 2.787506 | 0.00666 | 0.033718 | -2.77018 |
| UTS2 | 1.056922 | -0.43549 | 2.780395 | 0.006794 | 0.034266 | -2.78791 |
| XLOC_005341 | 1.267287 | 0.016539 | 2.769734 | 0.006999 | 0.035066 | -2.81442 |
| CXCL1 | 1.4511 | -0.68074 | 2.754786 | 0.007297 | 0.036263 | -2.85144 |
| CREB3L3 | 1.260033 | -0.13824 | 2.726102 | 0.007901 | 0.038542 | -2.92203 |
| KRT23 | 1.04092 | -0.00574 | 2.724275 | 0.007941 | 0.038702 | -2.92651 |
| CYP4F3 | 1.023503 | -0.54524 | 2.713471 | 0.008181 | 0.039632 | -2.95292 |
| GPR64 | -1.04184 | -0.27279 | -2.71266 | 0.0082 | 0.03969 | -2.95489 |
| SLC7A9 | 1.665836 | 0.78696 | 2.699308 | 0.008506 | 0.040774 | -2.98742 |
| MTTP | 1.997702 | 1.45862 | 2.673447 | 0.00913 | 0.043029 | -3.05003 |
| MMP7 | 1.677872 | -0.87393 | 2.670952 | 0.009192 | 0.043231 | -3.05604 |
| XLOC_l2_015800 | 1.225414 | -0.40203 | 2.670417 | 0.009206 | 0.043274 | -3.05733 |
| HLA-DQA1 | -1.08502 | -0.17298 | -2.64222 | 0.009938 | 0.045871 | -3.12495 |
| C17orf78 | 1.718356 | 2.442689 | 2.641571 | 0.009956 | 0.045939 | -3.12651 |
| PTPRO | 1.030499 | 0.858568 | 2.623015 | 0.010467 | 0.047818 | -3.17068 |
