# Supplementary material for: Explore the active ingredients and potential mechanisms of JianPi QingRe HuaYu Methods in the treatment of gastric inflammation-cancer transformation by network pharmacology and experimental validation
Source: BMC Complement Med Ther. 2023 Nov 14;23:411. doi: 10.1186/s12906-023-04232-0 (PMC10644588; doi:10.1186/s12906-023-04232-0)
Supplement: Supplementary file 4 — Additional file 4: Table S4. The DEGs between IGC and gastritis group In GSE130823 dataset. [file 12906_2023_4232_MOESM4_ESM.docx]

**Table S4. The DEGs between IGC and gastritis group In GSE130823 dataset.**

| Genes | logFC | AveExpr | t | P.Value | adj.P.Val | B |
| --- | --- | --- | --- | --- | --- | --- |
| ATP6V0D2 | 1.775882 | 0.746743 | 6.316079 | 3.01E-08 | 0.000965 | 8.366881 |
| IGF2BP3 | 2.019562 | 0.443242 | 5.977153 | 1.14E-07 | 0.001836 | 7.189181 |
| LOC100507520 | -1.70241 | 0.219285 | -5.50427 | 7.17E-07 | 0.007664 | 5.569317 |
| PIK3C2G | -1.18373 | 0.043361 | -5.15952 | 2.65E-06 | 0.016145 | 4.412369 |
| SNX10 | 1.264003 | 0.078602 | 4.957866 | 5.63E-06 | 0.023121 | 3.747639 |
| PRR19 | 1.16691 | -0.30969 | 4.705967 | 1.42E-05 | 0.038658 | 2.931973 |
| PIK3AP1 | 1.265968 | -0.29037 | 4.703669 | 1.43E-05 | 0.038658 | 2.924616 |
| KRTAP4-2 | -1.5578 | -0.10211 | -4.69457 | 1.48E-05 | 0.038658 | 2.895503 |
| LOC338799 | -1.27707 | 0.172442 | -4.63021 | 1.87E-05 | 0.038658 | 2.690188 |
| VMO1 | 1.413219 | -0.31675 | 4.61429 | 1.98E-05 | 0.038658 | 2.639616 |
| ANO8 | -1.26245 | -0.20409 | -4.54465 | 2.54E-05 | 0.038658 | 2.419261 |
| THSD4 | -1.7572 | 0.947012 | -4.53553 | 2.62E-05 | 0.038658 | 2.390529 |
| NINJ2 | 1.062281 | -0.1203 | 4.459735 | 3.43E-05 | 0.038658 | 2.152684 |
| LOC100507149 | -1.52419 | 0.344863 | -4.44175 | 3.66E-05 | 0.038658 | 2.096527 |
| SEC31B | -1.18199 | 0.105465 | -4.4393 | 3.69E-05 | 0.038658 | 2.088887 |
| CELA2B | -1.7742 | 0.637301 | -4.4263 | 3.86E-05 | 0.038658 | 2.048385 |
| HS3ST4 | -1.35346 | 1.093632 | -4.37335 | 4.65E-05 | 0.038658 | 1.883966 |
| XLOC_005220 | 1.490208 | -0.92778 | 4.366495 | 4.77E-05 | 0.038658 | 1.862752 |
| GPR19 | 1.443586 | 0.00333 | 4.350941 | 5.04E-05 | 0.038658 | 1.814679 |
| SNCA | 1.323372 | 0.159021 | 4.331518 | 5.39E-05 | 0.038658 | 1.754768 |
| XLOC_000682 | -1.1928 | 0.374642 | -4.3177 | 5.66E-05 | 0.038658 | 1.712229 |
| LOC100287415 | 1.494125 | -0.18518 | 4.305516 | 5.90E-05 | 0.038658 | 1.674773 |
| HENMT1 | 1.036699 | -0.38319 | 4.304779 | 5.92E-05 | 0.038658 | 1.67251 |
| LOC100506307 | -1.16127 | 0.171762 | -4.2966 | 6.09E-05 | 0.038658 | 1.647406 |
| LOC100128893 | -2.01562 | 0.058433 | -4.28978 | 6.24E-05 | 0.038658 | 1.626478 |
| GLTPD2 | 1.673339 | 0.190955 | 4.286673 | 6.30E-05 | 0.038658 | 1.616957 |
| LOC646976 | 1.007112 | -0.21161 | 4.275187 | 6.56E-05 | 0.038658 | 1.581776 |
| BATF3 | 1.291341 | -0.0479 | 4.246822 | 7.24E-05 | 0.038826 | 1.495107 |
| SKAP2 | 1.07549 | -0.58677 | 4.246024 | 7.26E-05 | 0.038826 | 1.492671 |
| PF4V1 | 1.19473 | 0.522464 | 4.234853 | 7.55E-05 | 0.038826 | 1.458624 |
| XLOC_012786 | -1.71296 | 0.274297 | -4.23249 | 7.61E-05 | 0.038826 | 1.45142 |
| ASXL3 | -1.86819 | 0.920197 | -4.20792 | 8.28E-05 | 0.038826 | 1.376712 |
| PTF1A | -1.96778 | 0.996707 | -4.18539 | 8.95E-05 | 0.038826 | 1.308431 |
| LOC100505592 | 1.552201 | -0.33909 | 4.171165 | 9.40E-05 | 0.038988 | 1.265401 |
| C10orf81 | 1.689091 | -0.89176 | 4.167491 | 9.52E-05 | 0.038988 | 1.2543 |
| FLJ42875 | -2.27352 | 0.805435 | -4.15471 | 9.95E-05 | 0.038988 | 1.215727 |
| SEMG1 | 1.759093 | -0.12156 | 4.137865 | 0.000105 | 0.038988 | 1.164989 |
| CNTN3 | -1.28268 | 0.045958 | -4.12316 | 0.000111 | 0.038988 | 1.120779 |
| DUOX1 | -1.79374 | 0.362191 | -4.11543 | 0.000114 | 0.038988 | 1.097568 |
| XLOC_001365 | -1.15463 | 0.50756 | -4.11162 | 0.000115 | 0.038988 | 1.086145 |
| SIPA1L2 | 1.324876 | -0.27251 | 4.10709 | 0.000117 | 0.038988 | 1.072576 |
| RARRES1 | 2.322913 | -0.44521 | 4.105098 | 0.000118 | 0.038988 | 1.066607 |
| S100A2 | 1.471283 | -0.65537 | 4.090421 | 0.000124 | 0.039065 | 1.022677 |
| EGFL8 | -1.04343 | 0.146446 | -4.08991 | 0.000124 | 0.039065 | 1.021143 |
| LINC00152 | 1.055703 | -0.18265 | 4.089774 | 0.000124 | 0.039065 | 1.020742 |
| PALM3 | -1.71431 | 0.585182 | -4.07424 | 0.000131 | 0.040392 | 0.974357 |
| LOC100507286 | 1.746197 | 0.268369 | 4.058025 | 0.000138 | 0.04113 | 0.926015 |
| LILRA4 | 1.217685 | 0.118084 | 4.041095 | 0.000147 | 0.041574 | 0.875667 |
| KCNIP4 | -1.25309 | -0.04138 | -4.03285 | 0.000151 | 0.041574 | 0.851203 |
| LOC400043 | -1.33377 | 0.278952 | -4.02832 | 0.000153 | 0.041574 | 0.837749 |
| RASGEF1A | 1.167624 | -0.01543 | 4.025993 | 0.000154 | 0.041574 | 0.830853 |
| ACADL | -1.69847 | 0.209495 | -4.01305 | 0.000161 | 0.041801 | 0.792522 |
| XLOC_012662 | -2.09249 | 1.246064 | -4.01157 | 0.000162 | 0.041801 | 0.788146 |
| LOC284581 | -1.055 | 0.269284 | -4.00641 | 0.000165 | 0.041801 | 0.772874 |
| LOC100505754 | -1.85001 | 0.824645 | -4.00135 | 0.000168 | 0.041801 | 0.757939 |
| XLOC_012670 | -2.52861 | 1.249015 | -3.99106 | 0.000174 | 0.041833 | 0.727542 |
| LOC150622 | -1.79304 | 0.857867 | -3.98535 | 0.000177 | 0.041833 | 0.710707 |
| TMEM178 | -1.13355 | 0.276887 | -3.98531 | 0.000177 | 0.041833 | 0.710608 |
| TPRXL | 1.603381 | 0.654873 | 3.98029 | 0.00018 | 0.041833 | 0.695811 |
| RANBP3L | -2.01757 | 1.967463 | -3.97542 | 0.000183 | 0.041833 | 0.681461 |
| TNFSF13B | 1.196389 | 0.099735 | 3.970868 | 0.000186 | 0.041833 | 0.668078 |
| LOC100129794 | -1.12556 | -0.13007 | -3.96752 | 0.000188 | 0.041833 | 0.658219 |
| XLOC_009075 | -1.35058 | 0.927007 | -3.94787 | 0.000201 | 0.042917 | 0.600557 |
| PPP2R3A | -1.34277 | 0.34541 | -3.93159 | 0.000212 | 0.043101 | 0.552859 |
| ITGAX | 1.043445 | 0.565471 | 3.921809 | 0.000219 | 0.043869 | 0.524282 |
| FBP2 | -1.23128 | -0.13141 | -3.91878 | 0.000221 | 0.04404 | 0.515425 |
| RGL3 | -1.3369 | 0.303601 | -3.90754 | 0.000229 | 0.044882 | 0.482664 |
| ZMIZ1 | -1.00346 | 0.807672 | -3.90313 | 0.000233 | 0.044998 | 0.469797 |
| LOC100507568 | 1.017436 | -0.35828 | 3.880481 | 0.000251 | 0.045497 | 0.403945 |
| PKDCC | 1.020581 | -0.52229 | 3.878236 | 0.000253 | 0.045497 | 0.39743 |
| CLDN1 | 2.150579 | -0.17119 | 3.864593 | 0.000265 | 0.045732 | 0.357881 |
| RGS1 | 1.619836 | -0.01682 | 3.864438 | 0.000265 | 0.045732 | 0.357431 |
| PLIN4 | -1.72641 | 0.082508 | -3.86137 | 0.000267 | 0.045732 | 0.348548 |
| XLOC_014216 | -2.39011 | 2.494268 | -3.85604 | 0.000272 | 0.045732 | 0.33314 |
| LOC100127909 | -1.22488 | 0.255626 | -3.84893 | 0.000279 | 0.045732 | 0.312573 |
| TFPI2 | 1.891952 | -0.29335 | 3.847222 | 0.00028 | 0.045732 | 0.307643 |
| RBPJL | -1.20565 | 0.851083 | -3.8446 | 0.000283 | 0.045732 | 0.300073 |
| ESRRG | -1.84402 | 1.027798 | -3.84279 | 0.000284 | 0.045732 | 0.294841 |
| KREMEN2 | 1.43308 | -0.01497 | 3.841131 | 0.000286 | 0.045732 | 0.29006 |
| CLEC4A | 1.211127 | 0.271723 | 3.83046 | 0.000296 | 0.045732 | 0.259293 |
| FGB | -2.68415 | 2.313354 | -3.80188 | 0.000325 | 0.046369 | 0.177135 |
| SLC2A4 | -2.13028 | 1.151904 | -3.79906 | 0.000328 | 0.046369 | 0.169044 |
| CA9 | -1.61773 | 0.033454 | -3.79695 | 0.000331 | 0.046369 | 0.163019 |
| REG4 | 3.448982 | -1.22002 | 3.795653 | 0.000332 | 0.046369 | 0.15929 |
| XLOC_007161 | -2.94183 | 2.15195 | -3.79533 | 0.000332 | 0.046369 | 0.158351 |
| IL2RA | 1.157825 | 0.341442 | 3.793205 | 0.000335 | 0.046382 | 0.152277 |
| LOC401037 | -1.13198 | 0.301246 | -3.7926 | 0.000335 | 0.046382 | 0.150535 |
| BST2 | 1.279802 | 0.069938 | 3.7897 | 0.000339 | 0.046484 | 0.142242 |
| FCAMR | 1.030245 | 0.017374 | 3.783265 | 0.000346 | 0.046484 | 0.123831 |
| CD55 | 1.645486 | -0.43998 | 3.781525 | 0.000348 | 0.046484 | 0.118856 |
| LOC100506452 | -1.74607 | 0.861574 | -3.78132 | 0.000348 | 0.046484 | 0.118255 |
| FOSB | 2.31831 | 0.104251 | 3.78041 | 0.000349 | 0.046484 | 0.115669 |
| HTR2B | 1.821806 | -0.17138 | 3.777789 | 0.000352 | 0.046484 | 0.108178 |
| RIMKLA | 1.090133 | -0.16798 | 3.777401 | 0.000353 | 0.046484 | 0.10707 |
| FAM20A | -1.25717 | 0.022565 | -3.77601 | 0.000354 | 0.046484 | 0.103102 |
| RGS18 | 1.223925 | -0.08198 | 3.775134 | 0.000355 | 0.046484 | 0.100594 |
| 3-3月 | 1.119766 | -0.61761 | 3.774269 | 0.000356 | 0.046484 | 0.098123 |
| XLOC_009021 | 1.249257 | -0.16893 | 3.772783 | 0.000358 | 0.046484 | 0.093882 |
| CEACAM7 | 1.721619 | -0.58964 | 3.758269 | 0.000375 | 0.046628 | 0.052491 |
| KCNJ13 | -1.46177 | 0.95102 | -3.75295 | 0.000382 | 0.046628 | 0.037357 |
| LOC100508226 | -1.29395 | 0.13814 | -3.75237 | 0.000383 | 0.046628 | 0.035692 |
| RPRM | -2.16416 | 2.149121 | -3.75058 | 0.000385 | 0.046628 | 0.030619 |
| IRX3 | -1.39539 | 0.223753 | -3.7468 | 0.00039 | 0.046628 | 0.019866 |
| HAVCR2 | 1.33549 | -0.10953 | 3.742645 | 0.000395 | 0.046628 | 0.008048 |
| LAMP3 | 1.756568 | -0.29182 | 3.741928 | 0.000396 | 0.046628 | 0.006011 |
| LOC728606 | -1.44135 | -0.09649 | -3.74138 | 0.000397 | 0.046628 | 0.004463 |
| RAPSN | -1.54498 | 0.694848 | -3.73452 | 0.000405 | 0.046628 | -0.01503 |
| PRAC | 1.264125 | 0.359502 | 3.73157 | 0.000409 | 0.046628 | -0.02339 |
| INPP4A | 1.06019 | -0.30683 | 3.730055 | 0.000411 | 0.046628 | -0.02769 |
| ODAM | -1.60836 | 0.011355 | -3.72749 | 0.000415 | 0.046628 | -0.03497 |
| CHRNA5 | 1.876946 | -1.03352 | 3.726923 | 0.000416 | 0.046628 | -0.03656 |
| UPP1 | 1.750534 | -0.79609 | 3.726669 | 0.000416 | 0.046628 | -0.03728 |
| LOC100128131 | -2.5117 | 2.284189 | -3.72229 | 0.000422 | 0.046781 | -0.04968 |
| ERBB4 | -1.02747 | 0.851648 | -3.71559 | 0.000431 | 0.046781 | -0.06864 |
| LOC283075 | -1.42174 | 0.405371 | -3.71419 | 0.000433 | 0.046781 | -0.07261 |
| CD274 | 1.118345 | 0.5362 | 3.713009 | 0.000435 | 0.046781 | -0.07595 |
| CKMT2 | -2.61265 | 1.152963 | -3.71219 | 0.000436 | 0.046781 | -0.07826 |
| CARD9 | 1.029165 | 0.205751 | 3.71213 | 0.000436 | 0.046781 | -0.07843 |
| PLIN5 | -2.64062 | 0.89174 | -3.70966 | 0.00044 | 0.046843 | -0.08541 |
| GPER | -2.291 | 1.179972 | -3.7077 | 0.000442 | 0.046882 | -0.09094 |
| CHIA | -5.0674 | 4.714213 | -3.70624 | 0.000444 | 0.046882 | -0.09507 |
| LOC283731 | -1.24532 | 0.744227 | -3.70488 | 0.000446 | 0.046882 | -0.09891 |
| PSPN | -1.34769 | 0.535043 | -3.70447 | 0.000447 | 0.046882 | -0.10008 |
| LINC00346 | 1.277023 | -0.17808 | 3.695271 | 0.000461 | 0.046882 | -0.12603 |
| LOC100130345 | -1.20648 | 0.667911 | -3.68999 | 0.000468 | 0.046882 | -0.1409 |
| APOBEC2 | -1.95438 | 0.964672 | -3.68988 | 0.000469 | 0.046882 | -0.14122 |
| AGMAT | 1.53587 | -1.12539 | 3.685016 | 0.000476 | 0.047014 | -0.15491 |
| LOC100130654 | -1.08727 | 0.467292 | -3.68332 | 0.000479 | 0.047014 | -0.15968 |
| CGNL1 | -1.63586 | 0.815707 | -3.67797 | 0.000487 | 0.047014 | -0.17474 |
| MRGPRX3 | -2.00579 | 1.808514 | -3.67362 | 0.000494 | 0.047014 | -0.18696 |
| GP2 | 2.672889 | -0.59879 | 3.671432 | 0.000497 | 0.047014 | -0.1931 |
| PRR4 | 2.660609 | 0.425245 | 3.670845 | 0.000498 | 0.047014 | -0.19475 |
| LOC100128714 | -1.01219 | 0.361269 | -3.67048 | 0.000499 | 0.047014 | -0.19578 |
| RRAGD | 1.40847 | 0.139539 | 3.66953 | 0.0005 | 0.047014 | -0.19844 |
| CALML4 | 1.119081 | -0.55303 | 3.666524 | 0.000505 | 0.047014 | -0.20688 |
| XLOC_000247 | -2.16368 | 1.162908 | -3.66538 | 0.000507 | 0.047014 | -0.21007 |
| Q6ILE4 | -1.10816 | 0.535055 | -3.66282 | 0.000511 | 0.047081 | -0.21725 |
| LOC100132593 | -1.74304 | 0.798155 | -3.66226 | 0.000512 | 0.047081 | -0.21883 |
| PTGER3 | -2.61514 | 2.102566 | -3.65759 | 0.00052 | 0.047626 | -0.23193 |
| EPOR | -1.09842 | 0.40092 | -3.65602 | 0.000523 | 0.047626 | -0.23632 |
| SDS | 1.429046 | 0.284854 | 3.645687 | 0.00054 | 0.048358 | -0.26523 |
| XLOC_004283 | -1.42099 | 0.502677 | -3.64103 | 0.000548 | 0.048358 | -0.27824 |
| IFI6 | 1.301776 | 0.292856 | 3.638044 | 0.000554 | 0.048358 | -0.28659 |
| LYPD5 | 1.425579 | -0.40584 | 3.635864 | 0.000558 | 0.048358 | -0.29267 |
| LOC100506983 | -1.61503 | 1.148119 | -3.63426 | 0.00056 | 0.048358 | -0.29715 |
| CD1B | 1.042198 | 0.105041 | 3.632067 | 0.000564 | 0.048358 | -0.30326 |
| XLOC_l2_011365 | -1.00187 | 0.262189 | -3.62994 | 0.000568 | 0.048358 | -0.30919 |
| C4BPA | 1.356413 | 0.173242 | 3.625991 | 0.000575 | 0.048358 | -0.3202 |
| TMEM184A | -2.04608 | 1.879673 | -3.62483 | 0.000578 | 0.048358 | -0.32344 |
| GNRH2 | -1.55105 | 0.817619 | -3.62471 | 0.000578 | 0.048358 | -0.32377 |
| FOS | 1.278086 | -0.01081 | 3.624132 | 0.000579 | 0.048358 | -0.32538 |
| FER1L4 | -1.31184 | 0.158325 | -3.61844 | 0.00059 | 0.049118 | -0.34123 |
| OR52K2 | -2.25862 | 1.406878 | -3.61385 | 0.000598 | 0.049295 | -0.35398 |
| MYBPC3 | -1.27535 | 0.808474 | -3.60711 | 0.000611 | 0.049295 | -0.37272 |
| LOC221442 | -1.07342 | -0.12834 | -3.60656 | 0.000612 | 0.049295 | -0.37424 |
| XLOC_012977 | 1.238247 | -0.2677 | 3.605564 | 0.000614 | 0.049295 | -0.37702 |
| XLOC_009934 | 1.367022 | 0.378543 | 3.602661 | 0.00062 | 0.049295 | -0.38507 |
| C8orf12 | -1.79016 | 0.89653 | -3.60254 | 0.00062 | 0.049295 | -0.38541 |
| LDHB | -1.21451 | 0.323783 | -3.60187 | 0.000622 | 0.049295 | -0.38727 |
| SLC7A7 | 1.256684 | -0.15771 | 3.601326 | 0.000623 | 0.049295 | -0.38878 |
| ELL2 | -1.09809 | 0.619719 | -3.59613 | 0.000633 | 0.049416 | -0.40318 |
| XLOC_l2_000080 | -1.55761 | 1.391349 | -3.59247 | 0.00064 | 0.04944 | -0.41334 |
| SLC1A2 | -1.67511 | 0.897731 | -3.58908 | 0.000647 | 0.04944 | -0.42271 |
| HSD17B11 | 1.055529 | -0.6332 | 3.585876 | 0.000654 | 0.04944 | -0.43158 |
| MUC13 | 1.654244 | -0.36895 | 3.582234 | 0.000662 | 0.04944 | -0.44166 |
| NFE2 | 1.386446 | -0.05924 | 3.581547 | 0.000663 | 0.04944 | -0.44356 |
| SAA1 | 3.041039 | 0.387996 | 3.580603 | 0.000665 | 0.04944 | -0.44616 |
| AGXT2L1 | -3.45527 | 1.656223 | -3.5766 | 0.000674 | 0.04944 | -0.45722 |
| P2RY4 | -1.81208 | 1.096832 | -3.57548 | 0.000676 | 0.04944 | -0.46031 |
| XLOC_013835 | -2.20217 | 1.895703 | -3.57516 | 0.000677 | 0.04944 | -0.4612 |
| FOXD1 | 2.118167 | 0.349749 | 3.573303 | 0.000681 | 0.04944 | -0.46633 |
| HTR1E | -1.38867 | 1.261536 | -3.57274 | 0.000682 | 0.04944 | -0.46787 |
| PDPN | 1.488326 | -0.1322 | 3.571296 | 0.000685 | 0.04944 | -0.47187 |
| TPD52L1 | -1.46906 | 0.645097 | -3.57103 | 0.000686 | 0.04944 | -0.4726 |
| ZSCAN22 | -1.34177 | 0.724616 | -3.5705 | 0.000687 | 0.04944 | -0.47408 |
| KCNJ15 | -1.54537 | 0.429987 | -3.56939 | 0.000689 | 0.049457 | -0.47712 |
| PLCXD3 | -1.9192 | 0.878892 | -3.56315 | 0.000703 | 0.049619 | -0.49433 |
| XLOC_l2_002611 | -2.65771 | 2.406479 | -3.55889 | 0.000713 | 0.049619 | -0.50608 |
| GLUL | -1.13247 | 0.333548 | -3.55772 | 0.000715 | 0.049619 | -0.50929 |
| CLRN3 | 2.85136 | -1.65537 | 3.557634 | 0.000715 | 0.049619 | -0.50952 |
| KLK8 | 2.594332 | 0.839984 | 3.557166 | 0.000716 | 0.049619 | -0.51082 |
| PI3 | 3.084968 | -0.54661 | 3.555228 | 0.000721 | 0.049619 | -0.51615 |
| TCTE1 | -1.06252 | 0.257349 | -3.55432 | 0.000723 | 0.049632 | -0.51866 |
| XLOC_011849 | -1.84417 | 1.10061 | -3.55379 | 0.000724 | 0.049632 | -0.52011 |
| XLOC_014418 | 1.338991 | -0.29137 | 3.54837 | 0.000737 | 0.049982 | -0.53501 |
