# Supplementary material for: Explore the active ingredients and potential mechanisms of JianPi QingRe HuaYu Methods in the treatment of gastric inflammation-cancer transformation by network pharmacology and experimental validation
Source: BMC Complement Med Ther. 2023 Nov 14;23:411. doi: 10.1186/s12906-023-04232-0 (PMC10644588; doi:10.1186/s12906-023-04232-0)
Supplement: Supplementary file 5 — Additional file 5: Table S5. The DEGs between IN and chronic gastritis group in GSE55696 dataset. [file 12906_2023_4232_MOESM5_ESM.docx]

**Table S5. The DEGs between IN and chronic gastritis group in GSE55696 dataset.**

| Genes | logFC | AveExpr | t | P.Value | adj.P.Val | B |
| --- | --- | --- | --- | --- | --- | --- |
| CLASRP | -1.74305 | 0.214487 | -14.0241 | 1.85E-20 | 3.40E-16 | 35.95037 |
| C15orf17 | -1.32123 | 0.288808 | -12.5892 | 2.26E-18 | 2.08E-14 | 31.34502 |
| ZBTB48 | -1.04128 | 0.163502 | -12.0442 | 1.50E-17 | 9.19E-14 | 29.52105 |
| SUN1 | -1.61626 | 0.194773 | -11.811 | 3.42E-17 | 1.25E-13 | 28.72819 |
| BCAR1 | -1.58723 | 0.134963 | -11.6236 | 6.65E-17 | 2.03E-13 | 28.0858 |
| SLC9A1 | -2.00586 | 0.244757 | -11.2138 | 2.89E-16 | 6.84E-13 | 26.66438 |
| GOLGA6L9 | -1.71169 | 0.347029 | -11.2054 | 2.98E-16 | 6.84E-13 | 26.63493 |
| RGNEF | -1.87713 | 0.316219 | -11.1057 | 4.28E-16 | 8.72E-13 | 26.28569 |
| MSX1 | 2.512485 | -0.47032 | 11.01578 | 5.93E-16 | 1.09E-12 | 25.96972 |
| ULK3 | -1.02277 | 0.12501 | -10.7482 | 1.57E-15 | 2.41E-12 | 25.02313 |
| FLJ45340 | -1.26836 | 0.171774 | -10.681 | 2.02E-15 | 2.72E-12 | 24.78412 |
| BCL2L2 | -1.10087 | 0.175766 | -10.6727 | 2.08E-15 | 2.72E-12 | 24.75442 |
| FLNB | -1.89986 | 0.15264 | -10.3839 | 6.04E-15 | 7.38E-12 | 23.72004 |
| SCAMP4 | -1.17119 | 0.131187 | -10.3371 | 7.18E-15 | 8.24E-12 | 23.55159 |
| AGSK1 | -1.6747 | 0.324114 | -10.3036 | 8.13E-15 | 8.78E-12 | 23.43089 |
| LOC653581 | 1.57108 | -0.06735 | 10.24431 | 1.01E-14 | 1.01E-11 | 23.21675 |
| CIRBP | -1.37483 | 0.09793 | -10.2353 | 1.05E-14 | 1.01E-11 | 23.18407 |
| LOC653075 | -1.5335 | 0.273271 | -10.1649 | 1.36E-14 | 1.25E-11 | 22.92948 |
| LTK | -2.28847 | 0.376862 | -10.1446 | 1.47E-14 | 1.28E-11 | 22.85592 |
| HOOK2 | -1.10155 | 0.197701 | -10.0273 | 2.28E-14 | 1.90E-11 | 22.42961 |
| ZNF211 | -1.73396 | 0.29466 | -9.92783 | 3.31E-14 | 2.64E-11 | 22.06708 |
| KIAA0664L3 | -1.68202 | 0.211848 | -9.90317 | 3.63E-14 | 2.70E-11 | 21.97704 |
| PPP1R3E | -1.49736 | 0.172396 | -9.89472 | 3.75E-14 | 2.70E-11 | 21.94619 |
| CYTH1 | -1.22195 | 0.207078 | -9.88933 | 3.83E-14 | 2.70E-11 | 21.9265 |
| KIAA0195 | -1.14477 | 0.201765 | -9.83623 | 4.68E-14 | 3.18E-11 | 21.7323 |
| RALGPS1 | -1.05148 | 0.150046 | -9.8157 | 5.05E-14 | 3.31E-11 | 21.65713 |
| FRMD4A | -2.16622 | 0.281091 | -9.79515 | 5.46E-14 | 3.45E-11 | 21.58187 |
| NAGPA | -1.17575 | 0.18554 | -9.7647 | 6.12E-14 | 3.74E-11 | 21.47023 |
| PER3 | -2.64574 | 0.369814 | -9.73501 | 6.85E-14 | 4.05E-11 | 21.3613 |
| LOC100505648 | -1.63685 | 0.127207 | -9.65482 | 9.27E-14 | 5.21E-11 | 21.06662 |
| CST2 | 1.464172 | -0.20392 | 9.611958 | 1.09E-13 | 5.72E-11 | 20.90885 |
| ANO7 | -2.82732 | 0.365625 | -9.53862 | 1.44E-13 | 7.34E-11 | 20.63847 |
| STBD1 | -1.62577 | 0.18346 | -9.40858 | 2.36E-13 | 1.08E-10 | 20.15775 |
| CCDC89 | -2.3965 | 0.398728 | -9.35278 | 2.92E-13 | 1.31E-10 | 19.951 |
| RPS7P5 | 1.095891 | -0.12971 | 9.339003 | 3.08E-13 | 1.34E-10 | 19.8999 |
| MIRLET7BHG | -2.25263 | 0.352915 | -9.21877 | 4.87E-13 | 1.82E-10 | 19.45324 |
| LOC100131564 | -1.54348 | 0.113977 | -9.19824 | 5.27E-13 | 1.93E-10 | 19.37686 |
| CSH2 | 1.063768 | -0.09574 | 9.182262 | 5.60E-13 | 2.02E-10 | 19.31738 |
| ALDOC | -2.51785 | 0.57512 | -9.16364 | 6.01E-13 | 2.06E-10 | 19.24804 |
| STS | -2.12122 | 0.56515 | -9.15906 | 6.12E-13 | 2.06E-10 | 19.23095 |
| C8orf71 | 1.485854 | 0.038369 | 9.157309 | 6.16E-13 | 2.06E-10 | 19.22445 |
| SGSM3 | -1.51254 | 0.160722 | -9.14097 | 6.56E-13 | 2.14E-10 | 19.16355 |
| PRDM16 | -1.93547 | 0.299612 | -9.13762 | 6.64E-13 | 2.14E-10 | 19.15109 |
| KIAA0319 | -2.89749 | 0.646427 | -9.1247 | 6.98E-13 | 2.19E-10 | 19.10291 |
| LOC388152 | -1.31185 | 0.246383 | -9.12044 | 7.10E-13 | 2.19E-10 | 19.08704 |
| CEACAM20 | 1.308271 | -0.05766 | 9.118252 | 7.16E-13 | 2.19E-10 | 19.07887 |
| SPSB4 | 1.142354 | 0.002005 | 9.02369 | 1.03E-12 | 2.90E-10 | 18.7259 |
| PRRT2 | -2.24936 | 0.119239 | -9.01684 | 1.06E-12 | 2.92E-10 | 18.70029 |
| RIPK4 | -1.33863 | 0.221965 | -8.99678 | 1.14E-12 | 3.08E-10 | 18.62532 |
| B4GALNT3 | -1.89861 | 0.217879 | -8.98119 | 1.21E-12 | 3.19E-10 | 18.56703 |
| IGSF9 | -1.62797 | 0.036715 | -8.97963 | 1.22E-12 | 3.19E-10 | 18.56116 |
| AHSG | 1.133793 | -0.14114 | 8.939387 | 1.42E-12 | 3.60E-10 | 18.4106 |
| CDC42SE1 | -1.05739 | 0.195116 | -8.93687 | 1.44E-12 | 3.60E-10 | 18.40118 |
| ADCK4 | -1.0459 | 0.037898 | -8.93358 | 1.45E-12 | 3.60E-10 | 18.38887 |
| MTMR3 | -1.14372 | 0.183104 | -8.93031 | 1.47E-12 | 3.60E-10 | 18.37663 |
| FAM83E | -1.58641 | 0.148223 | -8.92615 | 1.50E-12 | 3.61E-10 | 18.36103 |
| SPIRE2 | -1.03055 | 0.046764 | -8.904 | 1.63E-12 | 3.88E-10 | 18.27807 |
| LOC727849 | -1.62698 | 0.20721 | -8.89215 | 1.71E-12 | 3.98E-10 | 18.2337 |
| MICALL2 | -1.23933 | 0.110099 | -8.89124 | 1.71E-12 | 3.98E-10 | 18.23028 |
| SGSM1 | -1.83054 | 0.513489 | -8.85663 | 1.96E-12 | 4.49E-10 | 18.10057 |
| MAT2A | -1.00585 | 0.180208 | -8.81991 | 2.25E-12 | 5.04E-10 | 17.96283 |
| LCN15 | 1.047278 | -0.03811 | 8.792034 | 2.51E-12 | 5.53E-10 | 17.85818 |
| NPAS1 | -2.39874 | 0.34344 | -8.78958 | 2.53E-12 | 5.53E-10 | 17.84896 |
| LOC100292909 | -3.09637 | 0.162457 | -8.7529 | 2.92E-12 | 6.22E-10 | 17.7112 |
| CEACAM19 | -1.56238 | 0.14551 | -8.74964 | 2.95E-12 | 6.23E-10 | 17.69895 |
| ZSCAN18 | -2.12896 | -0.18236 | -8.73563 | 3.12E-12 | 6.43E-10 | 17.64631 |
| LIF | 1.437482 | -0.20518 | 8.705802 | 3.50E-12 | 6.99E-10 | 17.53417 |
| LOC100132363 | -2.16897 | 0.478101 | -8.69355 | 3.67E-12 | 7.04E-10 | 17.48809 |
| FER1L4 | -3.07391 | 0.026386 | -8.68469 | 3.80E-12 | 7.04E-10 | 17.45476 |
| CHKB | -1.11663 | 0.144431 | -8.68374 | 3.81E-12 | 7.04E-10 | 17.45121 |
| CCDC88B | -1.42081 | 0.004736 | -8.68198 | 3.84E-12 | 7.04E-10 | 17.44458 |
| USP34 | -1.01437 | 0.055947 | -8.631 | 4.67E-12 | 8.40E-10 | 17.25269 |
| ABCC5 | -2.25162 | 0.322684 | -8.60667 | 5.13E-12 | 9.00E-10 | 17.16108 |
| CSH1 | 1.123125 | -0.04446 | 8.605778 | 5.15E-12 | 9.00E-10 | 17.1577 |
| SLC17A4 | 1.338302 | -0.09442 | 8.601004 | 5.24E-12 | 9.08E-10 | 17.13972 |
| LRRC72 | 1.145828 | -0.10093 | 8.59276 | 5.41E-12 | 9.23E-10 | 17.10866 |
| ERVK13-1 | -1.46895 | 0.005861 | -8.59207 | 5.43E-12 | 9.23E-10 | 17.10608 |
| GCM1 | 1.247335 | -0.05317 | 8.582361 | 5.64E-12 | 9.49E-10 | 17.06947 |
| KAZN | -1.06323 | 0.043053 | -8.56704 | 5.98E-12 | 9.89E-10 | 17.01174 |
| DRP2 | 1.416471 | -0.23622 | 8.558428 | 6.18E-12 | 1.00E-09 | 16.97926 |
| OSBPL7 | -1.68237 | 0.247087 | -8.52398 | 7.07E-12 | 1.13E-09 | 16.84937 |
| AKNA | -1.25514 | -0.09581 | -8.52192 | 7.12E-12 | 1.13E-09 | 16.84159 |
| PBX4 | -1.39628 | 0.089171 | -8.51042 | 7.45E-12 | 1.15E-09 | 16.79821 |
| UGDH | -1.42432 | 0.333187 | -8.49557 | 7.89E-12 | 1.21E-09 | 16.74218 |
| OR1E1 | 1.946213 | -0.29455 | 8.457982 | 9.13E-12 | 1.37E-09 | 16.60028 |
| RIC3 | -1.566 | -0.07495 | -8.43316 | 1.00E-11 | 1.50E-09 | 16.50655 |
| CARTPT | -3.56142 | 0.479055 | -8.42774 | 1.03E-11 | 1.51E-09 | 16.48605 |
| EVPLL | -2.68396 | 0.1832 | -8.36754 | 1.30E-11 | 1.84E-09 | 16.25855 |
| IFT140 | -1.13743 | 0.210995 | -8.36695 | 1.30E-11 | 1.84E-09 | 16.2563 |
| CAHM | -1.50302 | 0.138786 | -8.36583 | 1.30E-11 | 1.84E-09 | 16.2521 |
| KIAA1683 | -1.62423 | 0.29527 | -8.35765 | 1.35E-11 | 1.88E-09 | 16.22116 |
| CRYGD | -3.11522 | 0.7449 | -8.35619 | 1.35E-11 | 1.88E-09 | 16.21564 |
| DGCR8 | -1.20018 | 0.04052 | -8.35244 | 1.37E-11 | 1.89E-09 | 16.20144 |
| CACNA1E | 1.304036 | -0.11719 | 8.346455 | 1.41E-11 | 1.91E-09 | 16.17881 |
| LOC100129407 | 1.218077 | -0.11477 | 8.342045 | 1.43E-11 | 1.93E-09 | 16.16214 |
| DBP | -2.29286 | 0.425378 | -8.3171 | 1.58E-11 | 2.08E-09 | 16.06776 |
| PCDHGA2 | -1.2437 | 0.006455 | -8.304 | 1.66E-11 | 2.17E-09 | 16.01821 |
| LOC399900 | -1.57191 | 0.350439 | -8.28484 | 1.79E-11 | 2.26E-09 | 15.94568 |
| RASAL1 | -1.39709 | 0.180592 | -8.28428 | 1.79E-11 | 2.26E-09 | 15.94359 |
| SERTAD2 | -1.11205 | 0.066445 | -8.28373 | 1.79E-11 | 2.26E-09 | 15.94147 |
| PPFIBP1 | -1.08509 | 0.124016 | -8.27822 | 1.83E-11 | 2.29E-09 | 15.92063 |
| MGC50722 | -1.74561 | -0.22629 | -8.26659 | 1.92E-11 | 2.38E-09 | 15.87659 |
| CD3G | -1.42158 | -0.05562 | -8.2646 | 1.93E-11 | 2.38E-09 | 15.86909 |
| FLJ38576 | 1.264497 | -0.17569 | 8.238637 | 2.14E-11 | 2.60E-09 | 15.77076 |
| EVL | -1.36565 | -0.17075 | -8.19744 | 2.51E-11 | 2.90E-09 | 15.61471 |
| FOSL2 | -1.30436 | 0.199016 | -8.19555 | 2.53E-11 | 2.90E-09 | 15.60753 |
| PDZD3 | -2.5599 | 0.547145 | -8.16659 | 2.83E-11 | 3.17E-09 | 15.49776 |
| NOXA1 | -1.0902 | -0.06472 | -8.16625 | 2.83E-11 | 3.17E-09 | 15.49648 |
| CARNS1 | -3.78179 | 0.356124 | -8.1512 | 3.00E-11 | 3.34E-09 | 15.43945 |
| CCDC42B | -1.75817 | 0.368713 | -8.13874 | 3.15E-11 | 3.49E-09 | 15.39219 |
| ZNF767 | -1.43736 | 0.024636 | -8.13708 | 3.17E-11 | 3.49E-09 | 15.38591 |
| ATP13A4 | -2.26357 | -0.01497 | -8.12722 | 3.30E-11 | 3.59E-09 | 15.3485 |
| NR4A2 | 2.179029 | -0.18527 | 8.118866 | 3.41E-11 | 3.68E-09 | 15.31684 |
| PAN2 | -1.00189 | 0.122536 | -8.10439 | 3.61E-11 | 3.85E-09 | 15.26193 |
| SLC22A12 | 1.035864 | -0.16113 | 8.102978 | 3.63E-11 | 3.85E-09 | 15.25658 |
| GUSBP1 | -1.03124 | 0.082857 | -8.10146 | 3.65E-11 | 3.85E-09 | 15.25081 |
| SCGB1D1 | -3.5005 | 0.819778 | -8.08481 | 3.89E-11 | 4.08E-09 | 15.18768 |
| ANKRD50 | 1.228966 | -0.17011 | 8.067995 | 4.15E-11 | 4.28E-09 | 15.12386 |
| LOC100289079 | 1.503769 | 0.05953 | 8.064912 | 4.20E-11 | 4.31E-09 | 15.11216 |
| C14orf132 | -1.92176 | 0.208435 | -8.0601 | 4.28E-11 | 4.34E-09 | 15.09392 |
| UNC5CL | -1.43591 | 0.058135 | -8.05901 | 4.30E-11 | 4.34E-09 | 15.08978 |
| GALNT12 | -1.24603 | 0.059265 | -8.05879 | 4.31E-11 | 4.34E-09 | 15.08893 |
| TBC1D3F | -1.36332 | 0.041045 | -8.05497 | 4.37E-11 | 4.38E-09 | 15.07443 |
| AQP2 | 1.534167 | -0.05708 | 8.05233 | 4.42E-11 | 4.39E-09 | 15.06442 |
| LOC100505576 | -1.58847 | -0.01579 | -8.05168 | 4.43E-11 | 4.39E-09 | 15.06195 |
| C17orf108 | -1.27987 | 0.088894 | -8.0232 | 4.95E-11 | 4.81E-09 | 14.95385 |
| CYP4F8 | -1.16878 | 0.070443 | -8.02295 | 4.95E-11 | 4.81E-09 | 14.95293 |
| CRIPAK | -1.06522 | 0.307614 | -8.01749 | 5.06E-11 | 4.88E-09 | 14.93217 |
| BZRAP1 | -1.60345 | -0.01821 | -7.98874 | 5.66E-11 | 5.37E-09 | 14.82304 |
| NET1 | -1.03031 | 0.132316 | -7.98776 | 5.68E-11 | 5.37E-09 | 14.81932 |
| KCNK10 | -1.55466 | 0.325307 | -7.96344 | 6.24E-11 | 5.88E-09 | 14.72695 |
| CHRNE | 1.154688 | -0.1142 | 7.955572 | 6.44E-11 | 6.03E-09 | 14.69708 |
| LOC728739 | 1.311767 | 0.013781 | 7.948529 | 6.62E-11 | 6.13E-09 | 14.67032 |
| EIF2C4 | -1.18169 | 0.070215 | -7.94624 | 6.68E-11 | 6.14E-09 | 14.66162 |
| ANKFY1 | -1.01387 | 0.022771 | -7.94444 | 6.72E-11 | 6.14E-09 | 14.65481 |
| TFAP2C | 1.260688 | -0.01557 | 7.943901 | 6.74E-11 | 6.14E-09 | 14.65275 |
| SYNGR4 | 1.100557 | 0.003333 | 7.942972 | 6.76E-11 | 6.14E-09 | 14.64922 |
| PRKCD | -1.15965 | 0.233449 | -7.94108 | 6.81E-11 | 6.16E-09 | 14.64205 |
| AK5 | -4.06103 | 0.985697 | -7.93889 | 6.87E-11 | 6.16E-09 | 14.63371 |
| SUCNR1 | -3.48555 | 0.917624 | -7.93183 | 7.06E-11 | 6.29E-09 | 14.60688 |
| ANXA2 | 1.7583 | -0.04821 | 7.928189 | 7.16E-11 | 6.35E-09 | 14.59306 |
| LOC729234 | -1.87273 | 0.117791 | -7.90796 | 7.75E-11 | 6.77E-09 | 14.5162 |
| PELI2 | -1.92465 | 0.221238 | -7.90667 | 7.79E-11 | 6.78E-09 | 14.5113 |
| KIAA0556 | -1.09801 | 0.364962 | -7.90069 | 7.97E-11 | 6.90E-09 | 14.48859 |
| IL1A | 1.564447 | -0.23302 | 7.891744 | 8.26E-11 | 7.08E-09 | 14.45459 |
| PLXNA2 | -1.21543 | 0.211655 | -7.88352 | 8.53E-11 | 7.25E-09 | 14.42336 |
| HIST1H2AA | 1.848512 | -0.3495 | 7.882907 | 8.55E-11 | 7.25E-09 | 14.42101 |
| CAPN8 | -1.92119 | 0.272878 | -7.88221 | 8.57E-11 | 7.25E-09 | 14.41838 |
| OXT | 1.078355 | 0.051422 | 7.87587 | 8.78E-11 | 7.39E-09 | 14.39427 |
| GABRA3 | 1.164754 | -0.05526 | 7.869914 | 8.99E-11 | 7.53E-09 | 14.37164 |
| NPVF | 1.2768 | -0.12416 | 7.867444 | 9.08E-11 | 7.57E-09 | 14.36225 |
| ADCYAP1R1 | 1.036277 | -0.07954 | 7.859153 | 9.38E-11 | 7.74E-09 | 14.33074 |
| GOLM1 | -1.16844 | 0.091703 | -7.85402 | 9.57E-11 | 7.80E-09 | 14.31121 |
| NR1D2 | -1.58621 | 0.213101 | -7.79814 | 1.19E-10 | 9.53E-09 | 14.09879 |
| C21orf2 | -1.38551 | 0.119162 | -7.78967 | 1.23E-10 | 9.81E-09 | 14.06659 |
| HHLA1 | 1.278834 | -0.10131 | 7.769076 | 1.33E-10 | 1.05E-08 | 13.98829 |
| LOC202181 | -1.69186 | 0.136154 | -7.76142 | 1.37E-10 | 1.07E-08 | 13.95919 |
| SLC27A3 | -1.29903 | 0.261032 | -7.74709 | 1.45E-10 | 1.12E-08 | 13.90468 |
| FCN3 | 1.013608 | -0.14212 | 7.746146 | 1.46E-10 | 1.12E-08 | 13.90109 |
| FLJ39653 | -1.31248 | 0.077251 | -7.73398 | 1.53E-10 | 1.16E-08 | 13.85484 |
| COX6B2 | 1.254043 | -0.13835 | 7.726846 | 1.57E-10 | 1.19E-08 | 13.82769 |
| ENPP7 | 1.044365 | -0.11222 | 7.722151 | 1.60E-10 | 1.21E-08 | 13.80984 |
| RASEF | -1.39533 | 0.180638 | -7.71184 | 1.67E-10 | 1.25E-08 | 13.77062 |
| SENP7 | -1.15136 | 0.168236 | -7.7046 | 1.71E-10 | 1.26E-08 | 13.74309 |
| PNPLA7 | -1.3248 | 0.083162 | -7.69958 | 1.75E-10 | 1.28E-08 | 13.724 |
| FZD5 | -1.24909 | 0.163862 | -7.69726 | 1.76E-10 | 1.29E-08 | 13.71517 |
| LOC643201 | -2.38227 | -0.09486 | -7.69582 | 1.77E-10 | 1.29E-08 | 13.70968 |
| EVX1 | 1.23145 | 0.054129 | 7.6953 | 1.78E-10 | 1.29E-08 | 13.70771 |
| KIAA1530 | -1.15354 | 0.299824 | -7.69495 | 1.78E-10 | 1.29E-08 | 13.70638 |
| ALPPL2 | 1.051812 | 0.014555 | 7.690871 | 1.81E-10 | 1.30E-08 | 13.69086 |
| TMEM80 | -1.20882 | 0.219737 | -7.68251 | 1.87E-10 | 1.33E-08 | 13.65906 |
| ZNF500 | -1.0261 | 0.041268 | -7.66881 | 1.97E-10 | 1.39E-08 | 13.60695 |
| GNRHR | 1.059268 | 0.033408 | 7.663829 | 2.01E-10 | 1.41E-08 | 13.58799 |
| MTMR9LP | -1.18006 | 0.033745 | -7.65506 | 2.08E-10 | 1.45E-08 | 13.55463 |
| TMEM131 | -1.02673 | 0.217445 | -7.6515 | 2.11E-10 | 1.46E-08 | 13.54107 |
| FOXJ2 | -1.06998 | 0.274544 | -7.64964 | 2.12E-10 | 1.47E-08 | 13.53402 |
| ZNF540 | -1.70076 | 0.134911 | -7.64017 | 2.20E-10 | 1.51E-08 | 13.49797 |
| FLJ25694 | 1.191231 | 0.005678 | 7.632237 | 2.27E-10 | 1.55E-08 | 13.4678 |
| LOC100128398 | -2.42696 | -0.22638 | -7.62637 | 2.33E-10 | 1.57E-08 | 13.44549 |
| SLCO2A1 | -1.56095 | 0.188971 | -7.62499 | 2.34E-10 | 1.57E-08 | 13.44024 |
| AHNAK | -1.28797 | 0.111618 | -7.62094 | 2.38E-10 | 1.59E-08 | 13.42483 |
| C5orf25 | -1.24083 | 0.1037 | -7.61876 | 2.40E-10 | 1.59E-08 | 13.41653 |
| HES2 | 1.947456 | -0.09025 | 7.617672 | 2.41E-10 | 1.59E-08 | 13.41239 |
| LOC100507520 | -2.57956 | -0.07238 | -7.61589 | 2.42E-10 | 1.59E-08 | 13.40562 |
| C1orf63 | -1.14767 | 0.088364 | -7.61571 | 2.43E-10 | 1.59E-08 | 13.40493 |
| NOTUM | 1.206462 | 0.004614 | 7.615545 | 2.43E-10 | 1.59E-08 | 13.40429 |
| CCND1 | -1.00514 | 0.033231 | -7.61137 | 2.47E-10 | 1.60E-08 | 13.38841 |
| LOC339352 | 1.473132 | -0.07611 | 7.599054 | 2.59E-10 | 1.66E-08 | 13.34155 |
| SGK494 | -1.40162 | -0.01285 | -7.59705 | 2.61E-10 | 1.66E-08 | 13.33394 |
| FAM156A | -1.02072 | 0.081806 | -7.58987 | 2.68E-10 | 1.70E-08 | 13.30659 |
| NEUROD2 | 1.720374 | -0.03309 | 7.585787 | 2.73E-10 | 1.72E-08 | 13.29107 |
| DGKD | -1.49791 | 0.22036 | -7.5843 | 2.74E-10 | 1.72E-08 | 13.28542 |
| ARHGEF4 | -3.03441 | 0.164323 | -7.57994 | 2.79E-10 | 1.75E-08 | 13.26881 |
| CATSPER2 | -1.48775 | -0.02498 | -7.57183 | 2.88E-10 | 1.79E-08 | 13.23797 |
| LINC00346 | 1.697101 | -0.2021 | 7.571738 | 2.88E-10 | 1.79E-08 | 13.23761 |
| LOC100272216 | -1.74053 | 0.142521 | -7.55502 | 3.07E-10 | 1.88E-08 | 13.17399 |
| KIAA1652 | -1.8947 | -0.02428 | -7.54519 | 3.19E-10 | 1.93E-08 | 13.13661 |
| SRGAP2 | -1.05449 | 0.095796 | -7.53716 | 3.30E-10 | 1.98E-08 | 13.10603 |
| SRRM2 | -1.09004 | 0.065735 | -7.53618 | 3.31E-10 | 1.98E-08 | 13.1023 |
| IL17RE | -1.19476 | 0.01392 | -7.53475 | 3.33E-10 | 1.98E-08 | 13.09686 |
| CHRNB2 | 1.209532 | 0.031309 | 7.527023 | 3.43E-10 | 2.02E-08 | 13.06747 |
| CCL1 | 1.428081 | -0.06618 | 7.522964 | 3.48E-10 | 2.05E-08 | 13.05202 |
| GGT7 | -1.05664 | -0.04792 | -7.52111 | 3.51E-10 | 2.06E-08 | 13.04497 |
| SIM2 | 2.177142 | -0.73868 | 7.511703 | 3.64E-10 | 2.13E-08 | 13.00917 |
| TMEM27 | -2.6467 | 0.470312 | -7.49822 | 3.84E-10 | 2.22E-08 | 12.95787 |
| CKLF | 1.117404 | -0.13344 | 7.491741 | 3.94E-10 | 2.26E-08 | 12.93321 |
| ZSCAN4 | -3.07603 | 0.294914 | -7.48542 | 4.03E-10 | 2.31E-08 | 12.90917 |
| LOC100507094 | -1.20364 | 0.059061 | -7.46601 | 4.35E-10 | 2.45E-08 | 12.8353 |
| KATNAL1 | -1.05236 | -0.04257 | -7.46081 | 4.44E-10 | 2.49E-08 | 12.81552 |
| OTUD1 | -1.79236 | 0.245867 | -7.46051 | 4.45E-10 | 2.49E-08 | 12.81439 |
| MRO | -2.02345 | 0.461849 | -7.45568 | 4.53E-10 | 2.53E-08 | 12.79599 |
| SLC39A11 | -1.33586 | 0.265544 | -7.44663 | 4.69E-10 | 2.61E-08 | 12.76157 |
| ARHGAP29 | -1.43948 | 0.14757 | -7.44354 | 4.75E-10 | 2.63E-08 | 12.7498 |
| FOXA3 | -1.20219 | 0.077269 | -7.44048 | 4.81E-10 | 2.66E-08 | 12.73816 |
| LOC100129917 | -1.22339 | 0.048109 | -7.43605 | 4.89E-10 | 2.69E-08 | 12.72132 |
| CASP14 | 1.029342 | -0.0399 | 7.43522 | 4.91E-10 | 2.69E-08 | 12.71814 |
| KCNQ1OT1 | -1.6943 | 0.039923 | -7.43491 | 4.91E-10 | 2.69E-08 | 12.71696 |
| OR7C2 | 2.282745 | -0.15751 | 7.431628 | 4.98E-10 | 2.71E-08 | 12.70447 |
| SLC30A2 | 1.157116 | -0.03509 | 7.429676 | 5.02E-10 | 2.72E-08 | 12.69704 |
| LOC100506328 | -3.66536 | 0.080203 | -7.40956 | 5.43E-10 | 2.90E-08 | 12.62049 |
| PCDHGA12 | -1.08673 | 0.064958 | -7.40761 | 5.47E-10 | 2.92E-08 | 12.6131 |
| C21orf90 | 3.313678 | -0.45921 | 7.402379 | 5.58E-10 | 2.95E-08 | 12.59318 |
| SCN8A | -1.8528 | 0.155967 | -7.40129 | 5.60E-10 | 2.96E-08 | 12.58902 |
| ALDH3A2 | -1.53695 | 0.300552 | -7.39577 | 5.73E-10 | 3.01E-08 | 12.56804 |
| KDM4C | -1.13313 | 0.078323 | -7.38902 | 5.88E-10 | 3.08E-08 | 12.54234 |
| TSHB | 1.049023 | 0.029464 | 7.381232 | 6.06E-10 | 3.15E-08 | 12.51272 |
| CLK1 | -1.13148 | 0.084901 | -7.37999 | 6.09E-10 | 3.16E-08 | 12.50798 |
| FAM110C | -1.08646 | -0.04197 | -7.37832 | 6.13E-10 | 3.17E-08 | 12.50163 |
| SLC35F3 | -2.14414 | -0.0422 | -7.37415 | 6.23E-10 | 3.21E-08 | 12.48578 |
| C16orf11 | 1.152882 | 0.065411 | 7.367874 | 6.39E-10 | 3.27E-08 | 12.4619 |
| C4orf38 | -1.5536 | -0.08188 | -7.36696 | 6.41E-10 | 3.27E-08 | 12.45843 |
| LOC100131581 | 1.179873 | 0.004385 | 7.366138 | 6.43E-10 | 3.27E-08 | 12.45529 |
| RBM6 | -1.05074 | -0.02703 | -7.36159 | 6.54E-10 | 3.32E-08 | 12.43798 |
| KLHL3 | -1.60429 | 0.279443 | -7.35392 | 6.74E-10 | 3.40E-08 | 12.40879 |
| TMEM178 | -1.12493 | 0.24335 | -7.34522 | 6.98E-10 | 3.49E-08 | 12.37571 |
| SLC45A4 | -1.44827 | 0.303028 | -7.34507 | 6.98E-10 | 3.49E-08 | 12.37512 |
| USP17 | 1.152515 | -0.16271 | 7.340806 | 7.10E-10 | 3.53E-08 | 12.35891 |
| CYP2C19 | -1.4802 | 0.049171 | -7.3365 | 7.22E-10 | 3.57E-08 | 12.34252 |
| TLCD2 | -1.27553 | 0.021744 | -7.33498 | 7.26E-10 | 3.58E-08 | 12.33674 |
| ATG16L2 | -1.34629 | -0.04321 | -7.33152 | 7.36E-10 | 3.62E-08 | 12.32357 |
| TET3 | -1.04032 | 0.139269 | -7.32815 | 7.46E-10 | 3.64E-08 | 12.31076 |
| RENBP | 1.076273 | -0.18235 | 7.327749 | 7.47E-10 | 3.64E-08 | 12.30924 |
| CPS1-IT1 | 1.675907 | -0.10703 | 7.327563 | 7.48E-10 | 3.64E-08 | 12.30853 |
| CST1 | 1.815805 | -0.13007 | 7.326335 | 7.51E-10 | 3.64E-08 | 12.30386 |
| SCARNA17 | -1.67035 | 0.192915 | -7.32597 | 7.52E-10 | 3.64E-08 | 12.30246 |
| CYP4F12 | -1.28573 | 0.170942 | -7.32588 | 7.52E-10 | 3.64E-08 | 12.30212 |
| HDAC10 | -1.07962 | -0.01112 | -7.30865 | 8.05E-10 | 3.85E-08 | 12.23659 |
| FOXA2 | -1.4124 | 0.182271 | -7.30783 | 8.07E-10 | 3.85E-08 | 12.23346 |
| LOC100147773 | -1.39647 | -0.10658 | -7.30472 | 8.17E-10 | 3.89E-08 | 12.22164 |
| CLIP2 | -1.2629 | 0.282261 | -7.30387 | 8.20E-10 | 3.89E-08 | 12.21839 |
| FKSG2 | 1.865405 | -0.09799 | 7.300874 | 8.30E-10 | 3.92E-08 | 12.20701 |
| HOTTIP | 1.530158 | -0.02724 | 7.297962 | 8.39E-10 | 3.95E-08 | 12.19593 |
| ANO1 | -1.49101 | 0.253264 | -7.28595 | 8.79E-10 | 4.12E-08 | 12.15023 |
| ENO3 | -1.08174 | 0.010742 | -7.28457 | 8.84E-10 | 4.13E-08 | 12.145 |
| MAGI3 | -1.37366 | 0.219569 | -7.28303 | 8.90E-10 | 4.14E-08 | 12.13914 |
| HOXB13 | 3.946453 | -0.71824 | 7.264516 | 9.56E-10 | 4.39E-08 | 12.06872 |
| CAPN9 | -2.31415 | 0.156987 | -7.263 | 9.62E-10 | 4.40E-08 | 12.06295 |
| ATG16L1 | -1.06787 | 0.075708 | -7.26 | 9.73E-10 | 4.44E-08 | 12.05153 |
| CHRNA6 | 1.156925 | -0.18602 | 7.257576 | 9.83E-10 | 4.47E-08 | 12.04232 |
| WISP3 | 3.452695 | 0.055981 | 7.256987 | 9.85E-10 | 4.47E-08 | 12.04008 |
| GPRIN2 | -1.22577 | 0.049697 | -7.2489 | 1.02E-09 | 4.57E-08 | 12.00933 |
| FAM86A | 1.124966 | -0.20493 | 7.243218 | 1.04E-09 | 4.64E-08 | 11.98772 |
| TNFRSF10C | 1.47206 | -0.3772 | 7.242685 | 1.04E-09 | 4.64E-08 | 11.98569 |
| ADCY4 | -1.24848 | -0.05323 | -7.2319 | 1.09E-09 | 4.78E-08 | 11.94468 |
| USP54 | -1.06045 | 0.163781 | -7.23171 | 1.09E-09 | 4.78E-08 | 11.94395 |
| LRRC56 | -1.14579 | 0.041772 | -7.23134 | 1.09E-09 | 4.78E-08 | 11.94254 |
| MOP-1 | 1.046795 | 0.054456 | 7.230797 | 1.09E-09 | 4.78E-08 | 11.94049 |
| SLC4A9 | 1.644696 | -0.178 | 7.220589 | 1.14E-09 | 4.95E-08 | 11.90167 |
| CCNL2 | -1.05079 | 0.062452 | -7.21145 | 1.18E-09 | 5.09E-08 | 11.86692 |
| ARNTL2 | -1.14135 | 0.133407 | -7.21019 | 1.18E-09 | 5.10E-08 | 11.86213 |
| LOC100272228 | -2.44545 | 0.195161 | -7.20959 | 1.19E-09 | 5.10E-08 | 11.85985 |
| LOC100130428 | -1.89682 | 0.276848 | -7.18844 | 1.29E-09 | 5.50E-08 | 11.77944 |
| CDA | 2.285761 | -0.20286 | 7.188038 | 1.29E-09 | 5.50E-08 | 11.7779 |
| SMA4 | -1.10113 | 0.175782 | -7.17929 | 1.33E-09 | 5.67E-08 | 11.74463 |
| CLDN19 | 1.124785 | -0.05369 | 7.168416 | 1.39E-09 | 5.87E-08 | 11.70331 |
| ELOVL7 | 1.648518 | -0.30124 | 7.151149 | 1.49E-09 | 6.22E-08 | 11.63768 |
| KLHDC1 | -1.11371 | 0.131509 | -7.14821 | 1.51E-09 | 6.28E-08 | 11.62652 |
| COL22A1 | -1.76947 | 0.044778 | -7.14744 | 1.51E-09 | 6.29E-08 | 11.62357 |
| ANXA2P3 | 1.886702 | -0.04607 | 7.146575 | 1.52E-09 | 6.29E-08 | 11.62029 |
| FLCN | -1.05699 | 0.070351 | -7.14583 | 1.52E-09 | 6.30E-08 | 11.61745 |
| MIR7-3HG | -1.66206 | 0.207167 | -7.13214 | 1.60E-09 | 6.58E-08 | 11.56543 |
| RGL3 | -1.72166 | 0.038563 | -7.13127 | 1.61E-09 | 6.59E-08 | 11.56214 |
| SLC34A2 | 1.398809 | -0.1362 | 7.127153 | 1.63E-09 | 6.67E-08 | 11.54648 |
| LOC100652953 | 1.465245 | -0.04724 | 7.116119 | 1.71E-09 | 6.90E-08 | 11.50455 |
| CRYGS | -1.48236 | 0.029283 | -7.10768 | 1.76E-09 | 7.07E-08 | 11.4725 |
| SLC9A3R2 | -1.68672 | 0.18654 | -7.10381 | 1.79E-09 | 7.11E-08 | 11.45776 |
| FOSB | 3.366814 | -0.01439 | 7.092753 | 1.87E-09 | 7.33E-08 | 11.41577 |
| APBB3 | -1.11446 | -0.00931 | -7.08016 | 1.96E-09 | 7.69E-08 | 11.36791 |
| LOC554207 | 1.000901 | -0.16798 | 7.076926 | 1.99E-09 | 7.76E-08 | 11.35564 |
| RRN3P2 | -1.28208 | -0.01645 | -7.07666 | 1.99E-09 | 7.76E-08 | 11.35464 |
| C2orf89 | 2.528229 | -0.35572 | 7.073086 | 2.02E-09 | 7.81E-08 | 11.34105 |
| SRGAP1 | -1.07882 | 0.048692 | -7.06931 | 2.05E-09 | 7.90E-08 | 11.32672 |
| LOC100506242 | -1.66414 | 0.388232 | -7.06146 | 2.11E-09 | 8.11E-08 | 11.29691 |
| RNF112 | -1.50544 | -0.09638 | -7.05734 | 2.15E-09 | 8.22E-08 | 11.28123 |
| LGALS9C | -1.18104 | 0.169645 | -7.05251 | 2.19E-09 | 8.33E-08 | 11.2629 |
| HEMK1 | -1.03871 | -0.01026 | -7.05104 | 2.20E-09 | 8.34E-08 | 11.25732 |
| SSTR2 | -2.06941 | 0.162817 | -7.04725 | 2.23E-09 | 8.45E-08 | 11.24291 |
| ZNF671 | -1.73807 | -0.17117 | -7.04591 | 2.24E-09 | 8.48E-08 | 11.23782 |
| C16orf79 | -1.14898 | 0.046898 | -7.04069 | 2.29E-09 | 8.60E-08 | 11.218 |
| SNRPC | 1.094251 | 0.173227 | 7.038057 | 2.31E-09 | 8.67E-08 | 11.20801 |
| CRHR2 | 1.023702 | -0.09361 | 7.037627 | 2.32E-09 | 8.67E-08 | 11.20638 |
| SYTL2 | -1.51684 | 0.014936 | -7.03338 | 2.36E-09 | 8.77E-08 | 11.19026 |
| DAPK2 | -1.12651 | -0.01371 | -7.0322 | 2.37E-09 | 8.80E-08 | 11.18577 |
| CHAT | 1.126593 | -0.03307 | 7.027933 | 2.41E-09 | 8.89E-08 | 11.16957 |
| MEIS2 | -1.36329 | 0.125309 | -7.02522 | 2.43E-09 | 8.95E-08 | 11.15927 |
| BAIAP2 | -1.26085 | -0.00723 | -7.01827 | 2.50E-09 | 9.14E-08 | 11.13287 |
| GRIP2 | -1.50868 | 0.058363 | -7.01476 | 2.53E-09 | 9.23E-08 | 11.11957 |
| RPS10P7 | 1.30442 | -0.08692 | 7.008245 | 2.60E-09 | 9.41E-08 | 11.09483 |
| ATP1B3 | 2.052274 | -0.561 | 7.008144 | 2.60E-09 | 9.41E-08 | 11.09444 |
| SEMA3B | -1.10903 | 0.074699 | -7.00374 | 2.65E-09 | 9.55E-08 | 11.07772 |
| CD300E | 1.193584 | -0.23721 | 7.0005 | 2.68E-09 | 9.62E-08 | 11.06542 |
| RIMKLB | -1.31471 | -0.15001 | -6.99311 | 2.76E-09 | 9.87E-08 | 11.03738 |
| TP73-AS1 | -2.12252 | -0.42393 | -6.98996 | 2.79E-09 | 9.97E-08 | 11.02541 |
| OR1L3 | 1.323491 | 0.011301 | 6.986097 | 2.83E-09 | 1.01E-07 | 11.01075 |
| CAPN13 | -1.65503 | 0.050203 | -6.98309 | 2.87E-09 | 1.02E-07 | 10.99934 |
| SYT8 | -1.59268 | 0.146868 | -6.98189 | 2.88E-09 | 1.02E-07 | 10.99479 |
| ASAP3 | -1.07876 | 0.153084 | -6.97418 | 2.97E-09 | 1.05E-07 | 10.96553 |
| C6orf141 | -1.32111 | 0.182288 | -6.97004 | 3.02E-09 | 1.06E-07 | 10.94983 |
| LOC100170939 | -1.03016 | 0.084352 | -6.9641 | 3.09E-09 | 1.09E-07 | 10.92726 |
| LOC728392 | -1.80103 | 0.197823 | -6.96237 | 3.11E-09 | 1.09E-07 | 10.92072 |
| CBX7 | -1.08736 | 0.126837 | -6.9587 | 3.15E-09 | 1.10E-07 | 10.90678 |
| MPP3 | -1.24338 | 0.111172 | -6.94448 | 3.33E-09 | 1.15E-07 | 10.85284 |
| LOC729164 | 1.164863 | -0.20076 | 6.939311 | 3.40E-09 | 1.17E-07 | 10.83322 |
| OR51A7 | 1.592325 | -0.32629 | 6.938058 | 3.42E-09 | 1.17E-07 | 10.82847 |
| RNU12 | 2.000688 | -0.11315 | 6.930214 | 3.52E-09 | 1.21E-07 | 10.79872 |
| LOC100288144 | -1.51653 | 0.386792 | -6.92938 | 3.54E-09 | 1.21E-07 | 10.79555 |
| THBD | -1.98626 | 0.021259 | -6.91872 | 3.69E-09 | 1.24E-07 | 10.75512 |
| ID4 | -2.11514 | -0.1165 | -6.91179 | 3.79E-09 | 1.27E-07 | 10.72885 |
| FLJ23867 | -1.03818 | 0.248469 | -6.90863 | 3.83E-09 | 1.28E-07 | 10.71687 |
| SCGB1D2 | -2.7986 | 0.552287 | -6.90192 | 3.94E-09 | 1.31E-07 | 10.69143 |
| PRPH | -1.71774 | 0.327038 | -6.89813 | 3.99E-09 | 1.33E-07 | 10.67703 |
| LOC100506630 | -2.89652 | 0.443916 | -6.89633 | 4.02E-09 | 1.33E-07 | 10.67021 |
| SH2D6 | -1.85059 | -0.02414 | -6.89051 | 4.11E-09 | 1.35E-07 | 10.64814 |
| RAPGEFL1 | -1.0892 | 0.092358 | -6.88969 | 4.13E-09 | 1.35E-07 | 10.64504 |
| FLJ43390 | -2.26571 | 0.8458 | -6.88418 | 4.22E-09 | 1.38E-07 | 10.62417 |
| EPM2AIP1 | -1.35794 | -0.15745 | -6.8838 | 4.22E-09 | 1.38E-07 | 10.62272 |
| DNAJC5G | 1.345679 | -0.08212 | 6.881078 | 4.27E-09 | 1.39E-07 | 10.6124 |
| HOXB7 | 1.726557 | 0.2524 | 6.878485 | 4.31E-09 | 1.39E-07 | 10.60257 |
| CYP2C9 | -1.83239 | -0.05328 | -6.87745 | 4.33E-09 | 1.39E-07 | 10.59863 |
| TFAP2A | 2.912843 | -0.45586 | 6.872894 | 4.41E-09 | 1.41E-07 | 10.58137 |
| CPNE5 | -1.14253 | -0.02386 | -6.86211 | 4.60E-09 | 1.47E-07 | 10.54048 |
| NRBP2 | -1.05564 | 0.00352 | -6.85983 | 4.64E-09 | 1.48E-07 | 10.53184 |
| LOC400752 | 1.290405 | -0.13778 | 6.856247 | 4.70E-09 | 1.50E-07 | 10.51828 |
| FSD1L | 1.065604 | -0.03456 | 6.843232 | 4.95E-09 | 1.55E-07 | 10.46896 |
| FRY | -1.52968 | 0.159854 | -6.83704 | 5.07E-09 | 1.59E-07 | 10.44551 |
| DISP2 | -1.20261 | 0.018936 | -6.82586 | 5.29E-09 | 1.64E-07 | 10.40314 |
| FLJ31713 | 1.039969 | -0.07483 | 6.824918 | 5.31E-09 | 1.64E-07 | 10.39958 |
| LOC90246 | 1.238221 | 0.019984 | 6.820706 | 5.40E-09 | 1.66E-07 | 10.38363 |
| MLPH | -1.07052 | 0.140927 | -6.81952 | 5.42E-09 | 1.66E-07 | 10.37914 |
| MEIS1 | -2.06473 | 0.110015 | -6.81882 | 5.44E-09 | 1.66E-07 | 10.37648 |
| TULP1 | 1.646827 | -0.14881 | 6.818622 | 5.44E-09 | 1.66E-07 | 10.37574 |
| HSPB1 | -1.05969 | 0.120398 | -6.81714 | 5.48E-09 | 1.67E-07 | 10.37011 |
| MAP6 | -1.45559 | 0.11197 | -6.81339 | 5.56E-09 | 1.69E-07 | 10.35594 |
| YWHAH | -1.11069 | 0.123657 | -6.80621 | 5.71E-09 | 1.74E-07 | 10.32874 |
| SYTL4 | -1.44803 | 0.221447 | -6.80236 | 5.80E-09 | 1.76E-07 | 10.31416 |
| C1orf51 | -2.68812 | 0.335667 | -6.79347 | 6.00E-09 | 1.81E-07 | 10.2805 |
| ESYT3 | -1.74083 | 0.175952 | -6.79113 | 6.06E-09 | 1.82E-07 | 10.27164 |
| TTYH1 | 1.541852 | -0.05949 | 6.782322 | 6.27E-09 | 1.87E-07 | 10.23828 |
| TCP10L2 | 1.267283 | -0.01664 | 6.781097 | 6.30E-09 | 1.88E-07 | 10.23365 |
| PER2 | -1.03794 | 0.116087 | -6.78076 | 6.31E-09 | 1.88E-07 | 10.23238 |
| CASR | -2.82163 | 0.810668 | -6.76511 | 6.70E-09 | 1.98E-07 | 10.17313 |
| LINC00086 | -2.3489 | -0.26352 | -6.76058 | 6.82E-09 | 2.01E-07 | 10.156 |
| CHRNB3 | 1.671059 | -0.01167 | 6.756756 | 6.93E-09 | 2.03E-07 | 10.14152 |
| SIDT2 | -1.34621 | 0.032289 | -6.7549 | 6.98E-09 | 2.04E-07 | 10.13448 |
| KANK2 | -1.04906 | 0.100421 | -6.75351 | 7.01E-09 | 2.05E-07 | 10.12924 |
| CGB2 | 1.014365 | -0.02815 | 6.751311 | 7.07E-09 | 2.06E-07 | 10.12092 |
| ARHGAP39 | 1.069158 | -0.07902 | 6.720173 | 7.98E-09 | 2.30E-07 | 10.00313 |
| CDKN2A | 1.80583 | -0.20684 | 6.719961 | 7.99E-09 | 2.30E-07 | 10.00233 |
| OR10J1 | 1.037366 | -0.0419 | 6.717904 | 8.05E-09 | 2.31E-07 | 9.994553 |
| KIAA1147 | -1.45564 | 0.253377 | -6.71141 | 8.26E-09 | 2.36E-07 | 9.97 |
| TMEM106A | -1.26725 | 0.231104 | -6.70619 | 8.43E-09 | 2.40E-07 | 9.950248 |
| RBM20 | -1.44669 | 0.255768 | -6.70293 | 8.54E-09 | 2.43E-07 | 9.937923 |
| LOC401320 | -1.12171 | 0.135511 | -6.70164 | 8.58E-09 | 2.43E-07 | 9.933052 |
| PSD3 | -1.54488 | 0.068961 | -6.7011 | 8.60E-09 | 2.44E-07 | 9.931011 |
| CCDC84 | -1.13078 | -0.01156 | -6.69556 | 8.79E-09 | 2.48E-07 | 9.910068 |
| FSTL4 | -1.70027 | -0.07351 | -6.69469 | 8.82E-09 | 2.49E-07 | 9.90678 |
| HSPA4L | 1.82408 | -0.16596 | 6.694679 | 8.82E-09 | 2.49E-07 | 9.906741 |
| CDH3 | 3.025386 | -0.54344 | 6.692058 | 8.91E-09 | 2.50E-07 | 9.896834 |
| DMBT1 | 4.632404 | -0.76891 | 6.691948 | 8.91E-09 | 2.50E-07 | 9.896418 |
| C4BPA | 1.52956 | -0.14948 | 6.690287 | 8.97E-09 | 2.52E-07 | 9.890141 |
| KRT14 | 2.24405 | -0.71821 | 6.689812 | 8.98E-09 | 2.52E-07 | 9.888346 |
| KRTDAP | -1.97051 | 0.227284 | -6.68892 | 9.02E-09 | 2.52E-07 | 9.884964 |
| ZNF454 | -1.79384 | 0.282956 | -6.68475 | 9.16E-09 | 2.54E-07 | 9.869228 |
| VILL | -1.3468 | 0.032049 | -6.68434 | 9.18E-09 | 2.54E-07 | 9.867654 |
| LOC728978 | -2.86008 | 0.065401 | -6.68296 | 9.23E-09 | 2.55E-07 | 9.862455 |
| DEPTOR | -1.27553 | 0.189234 | -6.67855 | 9.39E-09 | 2.59E-07 | 9.845764 |
| HIST1H3J | 1.09628 | -0.00748 | 6.677326 | 9.43E-09 | 2.60E-07 | 9.841156 |
| CHAD | -1.8356 | 0.448872 | -6.6772 | 9.44E-09 | 2.60E-07 | 9.840684 |
| VSIG2 | -1.46222 | -0.10226 | -6.67223 | 9.62E-09 | 2.63E-07 | 9.821906 |
| HCG27 | -1.87233 | 0.1189 | -6.6695 | 9.72E-09 | 2.64E-07 | 9.811567 |
| CCL24 | 1.041785 | 0.004988 | 6.662018 | 1.00E-08 | 2.71E-07 | 9.783319 |
| AGAP8 | -1.00183 | 0.106091 | -6.66087 | 1.01E-08 | 2.72E-07 | 9.778985 |
| KRT13 | 1.100881 | 0.133658 | 6.654435 | 1.03E-08 | 2.77E-07 | 9.75467 |
| OTX1 | 1.939132 | -0.46024 | 6.652797 | 1.04E-08 | 2.78E-07 | 9.748483 |
| KCNK17 | -1.90486 | 0.291864 | -6.65252 | 1.04E-08 | 2.78E-07 | 9.747427 |
| NEU4 | 2.333614 | -0.33095 | 6.645242 | 1.07E-08 | 2.84E-07 | 9.719947 |
| SAMHD1 | 1.106046 | -0.21727 | 6.637504 | 1.10E-08 | 2.92E-07 | 9.690728 |
| TSPAN5 | -1.89853 | 0.163755 | -6.63689 | 1.10E-08 | 2.92E-07 | 9.688407 |
| ITPKA | -1.41926 | -0.00406 | -6.63596 | 1.11E-08 | 2.92E-07 | 9.684888 |
| MYO15A | -1.05572 | 0.026188 | -6.63585 | 1.11E-08 | 2.92E-07 | 9.684477 |
| ATG9B | 1.193555 | -0.18593 | 6.635162 | 1.11E-08 | 2.92E-07 | 9.681885 |
| RTN1 | -1.8505 | 0.226578 | -6.63181 | 1.13E-08 | 2.95E-07 | 9.669225 |
| PAX6 | -2.3959 | 0.160689 | -6.62888 | 1.14E-08 | 2.98E-07 | 9.658152 |
| KIAA0895L | -1.1069 | -0.05647 | -6.62764 | 1.14E-08 | 2.99E-07 | 9.653493 |
| DUSP26 | -1.80431 | 0.078649 | -6.62333 | 1.16E-08 | 3.04E-07 | 9.637219 |
| TAGLN2 | -1.35017 | 0.185361 | -6.62271 | 1.17E-08 | 3.04E-07 | 9.634861 |
| FAM101A | -1.57637 | -0.00361 | -6.61899 | 1.18E-08 | 3.07E-07 | 9.620823 |
| ZIK1 | -2.01481 | 0.087376 | -6.61438 | 1.20E-08 | 3.12E-07 | 9.603424 |
| KCNJ6 | -2.27233 | 0.542258 | -6.6076 | 1.24E-08 | 3.19E-07 | 9.577825 |
| C17orf77 | 1.774879 | 0.050503 | 6.594277 | 1.30E-08 | 3.34E-07 | 9.527564 |
| STON2 | -1.22103 | 0.163391 | -6.59355 | 1.31E-08 | 3.34E-07 | 9.524808 |
| ARHGAP24 | -1.25848 | 0.099154 | -6.58739 | 1.34E-08 | 3.40E-07 | 9.50159 |
| ABLIM1 | -1.0835 | 0.258164 | -6.58735 | 1.34E-08 | 3.40E-07 | 9.501432 |
| GABRG2 | -1.35101 | 0.368711 | -6.58266 | 1.36E-08 | 3.43E-07 | 9.483728 |
| CCDC153 | -1.03948 | 0.058574 | -6.58036 | 1.37E-08 | 3.45E-07 | 9.475063 |
| BAIAP3 | -1.36825 | 0.133169 | -6.57879 | 1.38E-08 | 3.47E-07 | 9.469134 |
| LOC100129034 | -1.04406 | 0.199761 | -6.57434 | 1.41E-08 | 3.52E-07 | 9.452365 |
| SCNN1A | -1.06199 | 0.105713 | -6.57274 | 1.42E-08 | 3.54E-07 | 9.446306 |
| LOC643650 | -1.67219 | 0.058309 | -6.56175 | 1.48E-08 | 3.67E-07 | 9.404896 |
| KLHL20 | -1.01983 | 0.014769 | -6.55442 | 1.52E-08 | 3.76E-07 | 9.377226 |
| NS3BP | -1.45816 | -0.07667 | -6.55236 | 1.53E-08 | 3.79E-07 | 9.369488 |
| SLC5A10 | -1.64375 | 0.42091 | -6.54946 | 1.55E-08 | 3.83E-07 | 9.358555 |
| SDS | 1.127669 | -0.15142 | 6.547437 | 1.56E-08 | 3.85E-07 | 9.350923 |
| MYO7B | 3.341129 | -0.85461 | 6.544574 | 1.58E-08 | 3.88E-07 | 9.340129 |
| KIRREL3-AS3 | 1.333025 | -0.15001 | 6.543551 | 1.59E-08 | 3.89E-07 | 9.336273 |
| PKN1 | -1.35997 | 0.009137 | -6.54157 | 1.60E-08 | 3.91E-07 | 9.328798 |
| CTNNBL1 | -1.45154 | 0.339765 | -6.5369 | 1.63E-08 | 3.98E-07 | 9.311192 |
| SLC2A10 | -1.49462 | 0.068787 | -6.5342 | 1.64E-08 | 4.01E-07 | 9.301036 |
| LINC00479 | -2.12665 | 0.033703 | -6.53109 | 1.66E-08 | 4.05E-07 | 9.28933 |
| LOC100507487 | -1.46262 | 0.180244 | -6.52267 | 1.72E-08 | 4.16E-07 | 9.257605 |
| PIK3R3 | 1.006144 | -0.23146 | 6.517898 | 1.75E-08 | 4.23E-07 | 9.239613 |
| C15orf27 | -2.64022 | 0.028596 | -6.51458 | 1.77E-08 | 4.27E-07 | 9.227129 |
| NUP210 | -1.62914 | -0.29288 | -6.51271 | 1.79E-08 | 4.29E-07 | 9.220079 |
| SORL1 | -1.0784 | 0.204348 | -6.5079 | 1.82E-08 | 4.37E-07 | 9.201956 |
| SENP3 | -1.1601 | 0.016263 | -6.50687 | 1.83E-08 | 4.38E-07 | 9.198073 |
| MICALL1 | -1.33374 | 0.13549 | -6.50542 | 1.84E-08 | 4.39E-07 | 9.192612 |
| ITGA2 | 1.788065 | 0.06656 | 6.504967 | 1.84E-08 | 4.39E-07 | 9.190911 |
| XKR6 | -1.24589 | -0.15554 | -6.49701 | 1.90E-08 | 4.51E-07 | 9.160932 |
| TPRXL | 1.885332 | -0.11265 | 6.495871 | 1.91E-08 | 4.53E-07 | 9.156662 |
| LOC729059 | 1.117793 | -0.12474 | 6.492742 | 1.93E-08 | 4.57E-07 | 9.144883 |
| SLC44A2 | -1.24162 | 0.202303 | -6.48901 | 1.96E-08 | 4.62E-07 | 9.130838 |
| SEC14L5 | -2.20475 | 0.312957 | -6.48604 | 1.98E-08 | 4.67E-07 | 9.119656 |
| HOXA2 | -1.84494 | 0.15139 | -6.46834 | 2.12E-08 | 4.91E-07 | 9.053042 |
| SYT4 | -3.09374 | 0.974127 | -6.46762 | 2.13E-08 | 4.92E-07 | 9.050314 |
| SHANK1 | 1.991929 | -0.18969 | 6.464772 | 2.15E-08 | 4.96E-07 | 9.039616 |
| LOC142937 | -1.65904 | -0.06857 | -6.46148 | 2.18E-08 | 5.02E-07 | 9.027236 |
| RAB37 | -2.29294 | 0.249413 | -6.45458 | 2.24E-08 | 5.13E-07 | 9.001261 |
| KLK6 | 3.19083 | 0.636978 | 6.453764 | 2.24E-08 | 5.14E-07 | 8.998205 |
| GDF2 | 1.26753 | -0.25921 | 6.449928 | 2.28E-08 | 5.21E-07 | 8.983777 |
| NR3C1 | -1.27984 | -0.22777 | -6.4452 | 2.32E-08 | 5.28E-07 | 8.965983 |
| TNFSF9 | 1.050673 | -0.01795 | 6.443193 | 2.34E-08 | 5.31E-07 | 8.958446 |
| KCNJ5 | -1.85622 | 0.029848 | -6.42627 | 2.50E-08 | 5.61E-07 | 8.894837 |
| RPL24 | 1.068637 | -0.02508 | 6.425926 | 2.50E-08 | 5.61E-07 | 8.89353 |
| NEAT1 | -2.13693 | -0.0638 | -6.42578 | 2.50E-08 | 5.61E-07 | 8.892984 |
| ACSF2 | -1.37066 | 0.098654 | -6.42168 | 2.54E-08 | 5.67E-07 | 8.877565 |
| NPW | -2.61831 | 0.439574 | -6.41999 | 2.56E-08 | 5.69E-07 | 8.871205 |
| AFAP1-AS1 | 2.98116 | -0.82716 | 6.416995 | 2.59E-08 | 5.74E-07 | 8.859965 |
| TRIM45 | -1.0346 | 0.071275 | -6.41438 | 2.61E-08 | 5.77E-07 | 8.85015 |
| MAPK3 | -1.07261 | 0.071961 | -6.39865 | 2.78E-08 | 6.10E-07 | 8.791055 |
| CBWD6 | 1.44399 | 0.065077 | 6.393071 | 2.84E-08 | 6.21E-07 | 8.770088 |
| CYP17A1 | 1.499622 | -0.06947 | 6.387492 | 2.90E-08 | 6.33E-07 | 8.749135 |
| GPR84 | 1.264373 | -0.39727 | 6.383494 | 2.95E-08 | 6.41E-07 | 8.734121 |
| KCNK16 | -1.74205 | 0.12153 | -6.37702 | 3.02E-08 | 6.56E-07 | 8.709831 |
| SYTL5 | -1.66073 | -0.11831 | -6.37503 | 3.04E-08 | 6.59E-07 | 8.702363 |
| GPRASP1 | -1.34236 | -0.03785 | -6.37453 | 3.05E-08 | 6.60E-07 | 8.700479 |
| ISM2 | 1.259631 | -0.10501 | 6.373653 | 3.06E-08 | 6.61E-07 | 8.697177 |
| FAM174B | -1.17091 | 0.10924 | -6.36997 | 3.10E-08 | 6.67E-07 | 8.683339 |
| NR6A1 | 1.165925 | 0.030681 | 6.36134 | 3.21E-08 | 6.86E-07 | 8.650962 |
| PDXDC2P | -1.0285 | 0.076278 | -6.36119 | 3.21E-08 | 6.86E-07 | 8.650384 |
| HOXA13 | 4.94853 | -1.20583 | 6.35984 | 3.23E-08 | 6.87E-07 | 8.645335 |
| IL6R | -1.55628 | 0.135684 | -6.3598 | 3.23E-08 | 6.87E-07 | 8.645182 |
| HMP19 | -1.91125 | 0.39158 | -6.35481 | 3.29E-08 | 6.99E-07 | 8.626472 |
| CACNA2D2 | -1.73594 | 0.160359 | -6.35105 | 3.34E-08 | 7.07E-07 | 8.61237 |
| IGSF3 | -1.09761 | 0.008928 | -6.34707 | 3.39E-08 | 7.16E-07 | 8.597441 |
| CPE | -2.03104 | -0.04451 | -6.3306 | 3.61E-08 | 7.54E-07 | 8.535641 |
| TNNC1 | 2.806091 | 0.107391 | 6.327658 | 3.65E-08 | 7.61E-07 | 8.524625 |
| C6orf204 | -1.7383 | -0.23089 | -6.31285 | 3.87E-08 | 8.02E-07 | 8.469115 |
| ANKRD18B | -1.35386 | 0.205376 | -6.31217 | 3.88E-08 | 8.02E-07 | 8.466588 |
| C3orf14 | -1.56635 | -0.16071 | -6.30946 | 3.92E-08 | 8.10E-07 | 8.456418 |
| CNTD2 | 1.586442 | -0.10544 | 6.306866 | 3.96E-08 | 8.16E-07 | 8.446692 |
| C5orf38 | 1.567732 | -0.17485 | 6.304194 | 4.00E-08 | 8.20E-07 | 8.436682 |
| SEZ6L | -2.0003 | 0.380991 | -6.3019 | 4.04E-08 | 8.26E-07 | 8.42808 |
| RNASE7 | 1.012035 | -0.21965 | 6.298955 | 4.08E-08 | 8.32E-07 | 8.417055 |
| SIX1 | 3.667815 | -0.85426 | 6.298909 | 4.08E-08 | 8.32E-07 | 8.416882 |
| FAM20A | -2.66727 | -0.153 | -6.29361 | 4.17E-08 | 8.49E-07 | 8.39703 |
| BPNT1 | 1.261072 | 0.145935 | 6.290509 | 4.22E-08 | 8.57E-07 | 8.385417 |
| TRPV1 | -1.08152 | -0.06435 | -6.28903 | 4.24E-08 | 8.58E-07 | 8.379876 |
| FABP6 | 2.988556 | -0.20432 | 6.288987 | 4.24E-08 | 8.58E-07 | 8.379717 |
| WFDC5 | 1.095778 | -0.02061 | 6.288203 | 4.25E-08 | 8.60E-07 | 8.376782 |
| PILRB | -1.60882 | -0.07351 | -6.28726 | 4.27E-08 | 8.62E-07 | 8.373264 |
| SBK1 | 1.318278 | -0.17224 | 6.279709 | 4.40E-08 | 8.86E-07 | 8.344976 |
| LOC400756 | -1.15884 | 0.102862 | -6.27906 | 4.41E-08 | 8.87E-07 | 8.342548 |
| RARRES1 | 2.645343 | -0.4482 | 6.277925 | 4.43E-08 | 8.90E-07 | 8.338295 |
| SLC7A8 | -1.74313 | 0.257702 | -6.27716 | 4.44E-08 | 8.91E-07 | 8.335437 |
| CPAMD8 | -1.67282 | 0.06624 | -6.27694 | 4.44E-08 | 8.91E-07 | 8.334613 |
| CTSZ | 1.258339 | -0.373 | 6.270406 | 4.56E-08 | 9.09E-07 | 8.310147 |
| SNAP25 | -1.911 | 0.130198 | -6.26685 | 4.62E-08 | 9.19E-07 | 8.29684 |
| MMEL1 | -1.15306 | 0.04606 | -6.26378 | 4.67E-08 | 9.28E-07 | 8.285358 |
| ACRBP | 1.261682 | -0.02969 | 6.263615 | 4.68E-08 | 9.28E-07 | 8.284731 |
| GGA1 | -1.04248 | 0.007215 | -6.26287 | 4.69E-08 | 9.30E-07 | 8.281951 |
| SST | -3.66109 | -0.03251 | -6.26216 | 4.70E-08 | 9.31E-07 | 8.279287 |
| KCP | 1.818336 | -0.25429 | 6.261376 | 4.72E-08 | 9.31E-07 | 8.276353 |
| LYPD3 | 1.6486 | 0.041754 | 6.260956 | 4.73E-08 | 9.31E-07 | 8.274781 |
| ADAM28 | -1.59402 | -0.04353 | -6.25494 | 4.84E-08 | 9.49E-07 | 8.252273 |
| CA11 | -1.33741 | 0.163658 | -6.25455 | 4.84E-08 | 9.50E-07 | 8.250799 |
| GDF15 | 1.885793 | -0.08491 | 6.252109 | 4.89E-08 | 9.57E-07 | 8.241679 |
| FAM83A | 1.748713 | -0.03222 | 6.248946 | 4.95E-08 | 9.66E-07 | 8.229845 |
| SYT12 | 1.509068 | -0.28296 | 6.245147 | 5.02E-08 | 9.76E-07 | 8.215635 |
| WNT9B | 1.034103 | -0.08079 | 6.243058 | 5.06E-08 | 9.82E-07 | 8.207824 |
| GAST | -5.73516 | -0.20367 | -6.23919 | 5.14E-08 | 9.96E-07 | 8.193373 |
| CYTIP | -1.26256 | 0.013482 | -6.23662 | 5.19E-08 | 1.00E-06 | 8.183765 |
| FLJ36840 | -1.54385 | 0.035157 | -6.23658 | 5.19E-08 | 1.00E-06 | 8.18359 |
| ANTXR2 | 1.622643 | -0.33076 | 6.235572 | 5.21E-08 | 1.00E-06 | 8.179827 |
| PKDREJ | -1.68221 | 0.04534 | -6.22736 | 5.38E-08 | 1.03E-06 | 8.149107 |
| LOC100506478 | 1.227362 | -0.23117 | 6.218477 | 5.57E-08 | 1.06E-06 | 8.115922 |
| ETNK1 | -1.27244 | -0.04882 | -6.21795 | 5.58E-08 | 1.06E-06 | 8.113963 |
| ARL4C | -1.60744 | -0.23597 | -6.21766 | 5.58E-08 | 1.06E-06 | 8.112885 |
| CD59 | -1.07633 | 0.025547 | -6.2145 | 5.65E-08 | 1.08E-06 | 8.101066 |
| ZNF259P1 | 1.484995 | -0.11884 | 6.214182 | 5.66E-08 | 1.08E-06 | 8.09987 |
| FOXI3 | 1.074666 | -0.09433 | 6.21297 | 5.68E-08 | 1.08E-06 | 8.095343 |
| CHGB | -3.7101 | 0.420855 | -6.20763 | 5.80E-08 | 1.10E-06 | 8.075396 |
| TMEM92 | -1.1795 | 0.13626 | -6.20231 | 5.92E-08 | 1.11E-06 | 8.055523 |
| CLIC3 | -1.93864 | -0.01037 | -6.20204 | 5.93E-08 | 1.11E-06 | 8.054504 |
| FANK1 | -1.00789 | 0.023774 | -6.19759 | 6.03E-08 | 1.13E-06 | 8.037886 |
| ATPAF1-AS1 | -1.39019 | -0.00515 | -6.18649 | 6.29E-08 | 1.17E-06 | 7.996444 |
| ADK | 1.060957 | -0.02629 | 6.180925 | 6.43E-08 | 1.19E-06 | 7.97566 |
| C4BPB | 1.472995 | -0.06348 | 6.172726 | 6.64E-08 | 1.23E-06 | 7.94506 |
| JMJD7-PLA2G4B | -1.12027 | -0.02931 | -6.16896 | 6.73E-08 | 1.24E-06 | 7.931011 |
| CYP2S1 | -1.3289 | 0.030809 | -6.16822 | 6.75E-08 | 1.24E-06 | 7.928261 |
| LDHC | 1.780521 | -0.04628 | 6.16629 | 6.80E-08 | 1.25E-06 | 7.921043 |
| ZNF542 | -1.23263 | -0.13611 | -6.1526 | 7.17E-08 | 1.31E-06 | 7.869958 |
| TFPI | 1.400524 | -0.12488 | 6.152339 | 7.18E-08 | 1.31E-06 | 7.869 |
| EGR2 | 1.537368 | -0.33806 | 6.152033 | 7.19E-08 | 1.31E-06 | 7.86786 |
| GLIPR2 | -1.5382 | -0.19271 | -6.15169 | 7.19E-08 | 1.31E-06 | 7.86657 |
| SPP2 | 1.339437 | 0.001095 | 6.150796 | 7.22E-08 | 1.32E-06 | 7.863247 |
| C11orf67 | -1.10189 | 0.128117 | -6.1491 | 7.27E-08 | 1.32E-06 | 7.85694 |
| LEF1 | 1.373804 | -0.22884 | 6.148634 | 7.28E-08 | 1.32E-06 | 7.855187 |
| BAHCC1 | -1.20296 | -0.01465 | -6.14734 | 7.32E-08 | 1.33E-06 | 7.850376 |
| PTPRZ1 | -2.20702 | 0.117118 | -6.14546 | 7.37E-08 | 1.34E-06 | 7.843335 |
| PPIF | -1.01166 | 0.218855 | -6.13865 | 7.56E-08 | 1.37E-06 | 7.817963 |
| KAZALD1 | -1.10796 | 0.026378 | -6.13578 | 7.65E-08 | 1.38E-06 | 7.807255 |
| GALNT6 | -1.30845 | 0.152056 | -6.13387 | 7.70E-08 | 1.39E-06 | 7.80015 |
| MYO15B | -1.17005 | 0.081197 | -6.1332 | 7.72E-08 | 1.39E-06 | 7.797657 |
| GALE | -1.18005 | 0.12153 | -6.12933 | 7.84E-08 | 1.40E-06 | 7.783227 |
| MAGEH1 | -1.54386 | -0.08971 | -6.12833 | 7.87E-08 | 1.41E-06 | 7.779479 |
| BNIPL | -1.57383 | 0.139122 | -6.12634 | 7.93E-08 | 1.41E-06 | 7.77208 |
| UTRN | -1.14025 | -0.07321 | -6.12619 | 7.93E-08 | 1.41E-06 | 7.77153 |
| LOC100652974 | 1.381461 | 0.08111 | 6.124861 | 7.98E-08 | 1.42E-06 | 7.766571 |
| CHRM5 | 1.218205 | 0.072811 | 6.124665 | 7.98E-08 | 1.42E-06 | 7.765842 |
| TMEM130 | -2.17707 | 0.389121 | -6.11493 | 8.29E-08 | 1.46E-06 | 7.729584 |
| MUC5AC | -2.28673 | -0.02668 | -6.11318 | 8.34E-08 | 1.47E-06 | 7.723068 |
| KLK8 | 4.012498 | 0.170639 | 6.110443 | 8.43E-08 | 1.48E-06 | 7.712863 |
| LOC100192204 | 1.300507 | -0.10558 | 6.107124 | 8.54E-08 | 1.50E-06 | 7.700503 |
| FLJ36116 | 1.443062 | -0.09459 | 6.102507 | 8.69E-08 | 1.52E-06 | 7.683312 |
| TAGLN3 | -2.2192 | 0.389186 | -6.1013 | 8.73E-08 | 1.52E-06 | 7.67881 |
| ZNF175 | -1.08219 | -0.07667 | -6.10083 | 8.75E-08 | 1.52E-06 | 7.677059 |
| ADRB2 | -2.3276 | -0.21588 | -6.09786 | 8.85E-08 | 1.54E-06 | 7.666022 |
| LOC100507153 | -1.59827 | -0.03993 | -6.09466 | 8.96E-08 | 1.55E-06 | 7.654085 |
| C6orf132 | -1.0749 | 0.1541 | -6.09447 | 8.96E-08 | 1.55E-06 | 7.65339 |
| GATA6 | -1.23943 | 0.2108 | -6.0938 | 8.99E-08 | 1.55E-06 | 7.650896 |
| KRT16 | 2.404389 | -0.21147 | 6.090737 | 9.09E-08 | 1.57E-06 | 7.639498 |
| ZNF134 | -1.71291 | -0.10413 | -6.08661 | 9.24E-08 | 1.59E-06 | 7.624148 |
| DPP4 | 2.38002 | -0.35588 | 6.085617 | 9.27E-08 | 1.59E-06 | 7.620444 |
| EBLN2 | -1.10104 | -0.12666 | -6.07479 | 9.66E-08 | 1.65E-06 | 7.580179 |
| GABRA1 | -1.44578 | 0.313311 | -6.07458 | 9.67E-08 | 1.65E-06 | 7.579401 |
| LOC100133331 | -1.06794 | -0.06917 | -6.07183 | 9.77E-08 | 1.66E-06 | 7.569163 |
| RHOBTB3 | 2.078279 | -0.10907 | 6.071679 | 9.78E-08 | 1.66E-06 | 7.568593 |
| TLE4 | -1.07752 | -0.01799 | -6.06877 | 9.89E-08 | 1.68E-06 | 7.557778 |
| C5orf56 | -1.07032 | -0.11735 | -6.06596 | 1.00E-07 | 1.69E-06 | 7.547313 |
| EFNA2 | 1.468721 | -0.37252 | 6.064231 | 1.01E-07 | 1.70E-06 | 7.540895 |
| PFN3 | 1.249661 | -0.18184 | 6.062898 | 1.01E-07 | 1.71E-06 | 7.535938 |
| ADHFE1 | -2.54315 | 0.104906 | -6.0611 | 1.02E-07 | 1.72E-06 | 7.529272 |
| SPATA6 | -1.20214 | 0.22904 | -6.05464 | 1.04E-07 | 1.76E-06 | 7.505236 |
| LOC202025 | -1.84169 | -0.04821 | -6.05134 | 1.06E-07 | 1.77E-06 | 7.492958 |
| HSPA2 | -1.12224 | -0.02946 | -6.05074 | 1.06E-07 | 1.78E-06 | 7.490747 |
| GLDN | -2.98681 | 0.423106 | -6.04992 | 1.06E-07 | 1.78E-06 | 7.487701 |
| H1F0 | -1.21426 | 0.107776 | -6.04877 | 1.07E-07 | 1.78E-06 | 7.483441 |
| CHP2 | 5.839682 | -1.47517 | 6.04703 | 1.07E-07 | 1.79E-06 | 7.47696 |
| ANG | -1.23914 | 0.00373 | -6.04016 | 1.10E-07 | 1.84E-06 | 7.451423 |
| ZDHHC11 | -1.86004 | 0.185848 | -6.03949 | 1.11E-07 | 1.84E-06 | 7.448945 |
| PI16 | -1.52739 | -0.00559 | -6.03512 | 1.13E-07 | 1.86E-06 | 7.432728 |
| SMPD3 | -1.06297 | -0.03558 | -6.03381 | 1.13E-07 | 1.87E-06 | 7.427857 |
| MARCO | 1.3557 | -0.237 | 6.030411 | 1.15E-07 | 1.89E-06 | 7.415224 |
| HOXA10 | 3.473742 | -1.15646 | 6.029407 | 1.15E-07 | 1.90E-06 | 7.411493 |
| BCAS1 | -1.57489 | 0.0775 | -6.02921 | 1.15E-07 | 1.90E-06 | 7.41078 |
| CLDN3 | 2.689669 | -0.74151 | 6.02495 | 1.17E-07 | 1.92E-06 | 7.394944 |
| LOC644656 | -1.04999 | -0.06857 | -6.02447 | 1.17E-07 | 1.92E-06 | 7.393167 |
| CRYGC | -2.07504 | 0.513312 | -6.0242 | 1.17E-07 | 1.92E-06 | 7.392152 |
| LONRF2 | -1.82835 | -0.21722 | -6.02236 | 1.18E-07 | 1.94E-06 | 7.385341 |
| CITED1 | 1.414926 | 0.150746 | 6.021589 | 1.18E-07 | 1.94E-06 | 7.382466 |
| KLF7 | -1.0688 | -0.00874 | -6.02155 | 1.19E-07 | 1.94E-06 | 7.382324 |
| RGS1 | 1.845798 | -0.34493 | 6.018421 | 1.20E-07 | 1.96E-06 | 7.370707 |
| KCTD19 | -1.92176 | 0.154124 | -6.01632 | 1.21E-07 | 1.97E-06 | 7.362914 |
| RDH16 | 1.052349 | 0.003817 | 6.013375 | 1.22E-07 | 1.99E-06 | 7.351977 |
| CRYBB3 | 1.201489 | 0.014402 | 6.012265 | 1.23E-07 | 2.00E-06 | 7.347856 |
| TMEM38A | -1.03804 | 0.03341 | -6.01019 | 1.24E-07 | 2.01E-06 | 7.340141 |
| SHD | 2.034291 | 0.1991 | 6.006778 | 1.25E-07 | 2.03E-06 | 7.327494 |
| TEF | -1.02906 | 0.312637 | -5.99861 | 1.29E-07 | 2.08E-06 | 7.297189 |
| KLB | -1.37832 | 0.162735 | -5.9962 | 1.31E-07 | 2.10E-06 | 7.288255 |
| ZNF285 | -1.97226 | -0.25082 | -5.9931 | 1.32E-07 | 2.12E-06 | 7.27677 |
| C7orf54 | -1.71638 | 0.07363 | -5.98998 | 1.34E-07 | 2.13E-06 | 7.2652 |
| LOC100507218 | -2.13277 | 0.075425 | -5.98878 | 1.34E-07 | 2.14E-06 | 7.260741 |
| RPTN | -2.10918 | 0.753122 | -5.98391 | 1.37E-07 | 2.18E-06 | 7.2427 |
| LILRA4 | 1.120683 | -0.3979 | 5.979088 | 1.39E-07 | 2.21E-06 | 7.224808 |
| NANOG | -1.9796 | 0.189035 | -5.97854 | 1.40E-07 | 2.21E-06 | 7.222772 |
| HEY2 | -1.564 | 0.052859 | -5.97629 | 1.41E-07 | 2.23E-06 | 7.214448 |
| DLK2 | 1.228447 | -0.02586 | 5.968464 | 1.45E-07 | 2.28E-06 | 7.185439 |
| NLRP14 | 1.565554 | -0.27684 | 5.966832 | 1.46E-07 | 2.29E-06 | 7.179393 |
| CNTF | 1.370394 | 0.252947 | 5.965349 | 1.47E-07 | 2.30E-06 | 7.173899 |
| IL17C | 1.545061 | -0.17838 | 5.964543 | 1.47E-07 | 2.31E-06 | 7.170912 |
| ZNF682 | -1.06491 | -0.08172 | -5.96404 | 1.48E-07 | 2.31E-06 | 7.169045 |
| CRMP1 | -1.37539 | 0.201779 | -5.95746 | 1.51E-07 | 2.36E-06 | 7.14467 |
| RGAG4 | -1.61902 | -0.07075 | -5.95186 | 1.55E-07 | 2.39E-06 | 7.123939 |
| TNNI2 | -1.22365 | 0.015731 | -5.93992 | 1.62E-07 | 2.49E-06 | 7.079757 |
| NRG4 | -1.59821 | 0.024066 | -5.93853 | 1.63E-07 | 2.50E-06 | 7.074604 |
| FHDC1 | -1.21267 | 0.047554 | -5.93717 | 1.64E-07 | 2.50E-06 | 7.069549 |
| TFCP2L1 | -1.55343 | 0.251797 | -5.93714 | 1.64E-07 | 2.50E-06 | 7.069441 |
| HOXA11-AS1 | 5.260389 | -1.21741 | 5.935559 | 1.65E-07 | 2.51E-06 | 7.063605 |
| MAP7D2 | -2.10938 | -0.14483 | -5.93102 | 1.67E-07 | 2.55E-06 | 7.046828 |
| NANOS3 | 2.688297 | 0.001523 | 5.929053 | 1.69E-07 | 2.57E-06 | 7.039534 |
| KCTD13 | -1.00601 | 0.273908 | -5.92765 | 1.70E-07 | 2.58E-06 | 7.034325 |
| KLF8 | -1.10233 | -0.08792 | -5.92501 | 1.71E-07 | 2.59E-06 | 7.024573 |
| LOC100505974 | 1.17699 | -0.08663 | 5.924741 | 1.72E-07 | 2.60E-06 | 7.023582 |
| KRT17 | 3.878676 | -1.54414 | 5.924399 | 1.72E-07 | 2.60E-06 | 7.022318 |
| CHRNA2 | 1.202065 | -0.22158 | 5.923556 | 1.72E-07 | 2.60E-06 | 7.019199 |
| NR1D1 | -1.91803 | 0.235571 | -5.9227 | 1.73E-07 | 2.60E-06 | 7.016045 |
| ZNF404 | -1.99422 | 0.03446 | -5.91959 | 1.75E-07 | 2.63E-06 | 7.004528 |
| NRXN2 | 1.115233 | -0.08715 | 5.916976 | 1.77E-07 | 2.65E-06 | 6.994867 |
| UGT2B28 | 1.335274 | -0.06458 | 5.91521 | 1.78E-07 | 2.66E-06 | 6.988338 |
| AKAP8L | -1.14315 | -0.07015 | -5.90576 | 1.84E-07 | 2.75E-06 | 6.953426 |
| UGCG | -1.03458 | -0.14678 | -5.89988 | 1.89E-07 | 2.80E-06 | 6.931674 |
| KHDC1 | -1.39603 | -0.18767 | -5.89864 | 1.89E-07 | 2.81E-06 | 6.927114 |
| MUC1 | -1.3532 | -0.09845 | -5.89824 | 1.90E-07 | 2.82E-06 | 6.92561 |
| KLF2 | -1.31653 | -0.08543 | -5.89588 | 1.92E-07 | 2.84E-06 | 6.916888 |
| SLC15A1 | -1.24759 | 0.132807 | -5.89373 | 1.93E-07 | 2.85E-06 | 6.908976 |
| SPATA5 | -1.45835 | 0.102223 | -5.89176 | 1.95E-07 | 2.87E-06 | 6.901677 |
| SFTPD | -1.34691 | 0.140059 | -5.88738 | 1.98E-07 | 2.91E-06 | 6.885506 |
| NR0B1 | -2.16724 | 0.308383 | -5.88725 | 1.98E-07 | 2.91E-06 | 6.885038 |
| SPAG16 | -1.26359 | 0.032049 | -5.88468 | 2.00E-07 | 2.94E-06 | 6.875543 |
| LOC100505633 | -1.61386 | -0.15096 | -5.87861 | 2.05E-07 | 2.99E-06 | 6.853125 |
| SPATA12 | 1.253051 | -0.03768 | 5.878156 | 2.05E-07 | 2.99E-06 | 6.851449 |
| WNT9A | 1.058224 | -0.19728 | 5.870004 | 2.11E-07 | 3.08E-06 | 6.821361 |
| ADCY2 | -1.07237 | -0.00209 | -5.86881 | 2.12E-07 | 3.09E-06 | 6.816954 |
| NIM1 | -1.76273 | 0.189613 | -5.86744 | 2.13E-07 | 3.10E-06 | 6.811898 |
| HAMP | 1.361912 | -0.25992 | 5.866989 | 2.14E-07 | 3.10E-06 | 6.810233 |
| FMN1 | -1.49445 | 0.244688 | -5.86688 | 2.14E-07 | 3.10E-06 | 6.809821 |
| FAM110B | -1.72259 | -0.08489 | -5.86172 | 2.18E-07 | 3.15E-06 | 6.790782 |
| GALR2 | 1.094233 | -0.0295 | 5.860543 | 2.19E-07 | 3.16E-06 | 6.786455 |
| PKD1P1 | -1.24155 | 0.046326 | -5.85473 | 2.24E-07 | 3.21E-06 | 6.765006 |
| TCEA2 | -1.43994 | -0.38493 | -5.85181 | 2.26E-07 | 3.24E-06 | 6.754235 |
| PRICKLE1 | -1.81782 | -0.10733 | -5.85152 | 2.27E-07 | 3.24E-06 | 6.753174 |
| CYP46A1 | 1.217608 | -0.21907 | 5.851121 | 2.27E-07 | 3.24E-06 | 6.751705 |
| ANK2 | 1.33726 | 0.196314 | 5.845227 | 2.32E-07 | 3.30E-06 | 6.729973 |
| TEP1 | -1.02973 | 0.140035 | -5.84011 | 2.37E-07 | 3.35E-06 | 6.711124 |
| RPL23AP32 | 1.277256 | 0.046028 | 5.830049 | 2.46E-07 | 3.46E-06 | 6.674042 |
| FZD8 | -2.02315 | 0.081053 | -5.82836 | 2.48E-07 | 3.48E-06 | 6.667832 |
| ANKRD29 | -1.80267 | -0.27496 | -5.82424 | 2.52E-07 | 3.54E-06 | 6.652653 |
| TMEM196 | -1.76782 | 0.608098 | -5.81929 | 2.56E-07 | 3.59E-06 | 6.634414 |
| NEUROD1 | -3.17444 | -0.47579 | -5.81919 | 2.56E-07 | 3.59E-06 | 6.634041 |
| L3MBTL1 | -1.02624 | -0.03676 | -5.8174 | 2.58E-07 | 3.61E-06 | 6.627475 |
| HERC2P2 | -1.3206 | -0.04593 | -5.81406 | 2.61E-07 | 3.65E-06 | 6.61517 |
| TSKU | -1.12319 | 0.068216 | -5.81355 | 2.62E-07 | 3.66E-06 | 6.613288 |
| IL22RA2 | 1.207855 | 0.051867 | 5.80556 | 2.70E-07 | 3.75E-06 | 6.583873 |
| SIGLEC15 | 1.000098 | -0.0388 | 5.805436 | 2.70E-07 | 3.75E-06 | 6.583416 |
| GABRB2 | 1.076133 | -0.23564 | 5.804783 | 2.71E-07 | 3.76E-06 | 6.581015 |
| SATB2 | 1.085768 | -0.33993 | 5.804497 | 2.71E-07 | 3.76E-06 | 6.579962 |
| C2orf19 | 1.027856 | -0.12522 | 5.804433 | 2.71E-07 | 3.76E-06 | 6.579728 |
| LOC100507056 | 2.19115 | -0.52892 | 5.802904 | 2.73E-07 | 3.77E-06 | 6.5741 |
| PSAPL1 | -3.41036 | -0.5348 | -5.80257 | 2.73E-07 | 3.77E-06 | 6.572886 |
| HEPH | 2.375113 | -0.55531 | 5.797561 | 2.78E-07 | 3.83E-06 | 6.554443 |
| CCL3L3 | 1.378907 | -0.39469 | 5.79727 | 2.79E-07 | 3.83E-06 | 6.553375 |
| ZNF471 | -1.27333 | 0.194104 | -5.79564 | 2.80E-07 | 3.85E-06 | 6.547389 |
| LOC100506898 | -1.45235 | 0.214512 | -5.79441 | 2.82E-07 | 3.86E-06 | 6.542865 |
| LINC00087 | -2.07901 | -0.40043 | -5.79307 | 2.83E-07 | 3.87E-06 | 6.537907 |
| LOC100508226 | -1.8243 | 0.259739 | -5.79261 | 2.84E-07 | 3.88E-06 | 6.536229 |
| C12orf36 | -1.56502 | -0.05131 | -5.79143 | 2.85E-07 | 3.88E-06 | 6.531895 |
| CAMK2B | -1.57843 | 0.008611 | -5.78398 | 2.93E-07 | 3.98E-06 | 6.504494 |
| HKDC1 | 2.685639 | -0.82194 | 5.783065 | 2.94E-07 | 3.99E-06 | 6.501137 |
| PRAP1 | 3.585972 | -1.10192 | 5.779551 | 2.98E-07 | 4.04E-06 | 6.488216 |
| TMPRSS6 | -1.33393 | 0.243299 | -5.77852 | 2.99E-07 | 4.05E-06 | 6.484413 |
| OR10A2 | 1.61502 | -0.24459 | 5.772389 | 3.06E-07 | 4.13E-06 | 6.461897 |
| SOX21 | -2.90865 | -0.49055 | -5.77205 | 3.07E-07 | 4.13E-06 | 6.460664 |
| PON2 | 1.15472 | -0.06293 | 5.771775 | 3.07E-07 | 4.14E-06 | 6.45964 |
| ZNF132 | -1.51844 | -0.10024 | -5.77135 | 3.07E-07 | 4.14E-06 | 6.458088 |
| PLLP | -1.33962 | 0.161751 | -5.76823 | 3.11E-07 | 4.18E-06 | 6.446619 |
| ELOVL6 | -1.09868 | 0.168511 | -5.76145 | 3.19E-07 | 4.26E-06 | 6.421701 |
| KANK4 | -2.61607 | -0.89278 | -5.75974 | 3.21E-07 | 4.28E-06 | 6.415447 |
| LOC100132356 | -1.33647 | -0.13178 | -5.75664 | 3.25E-07 | 4.32E-06 | 6.404057 |
| OR8J1 | 1.098317 | -0.09755 | 5.753444 | 3.29E-07 | 4.36E-06 | 6.392315 |
| ERO1LB | -1.46762 | 0.187471 | -5.7511 | 3.32E-07 | 4.40E-06 | 6.383697 |
| GPR173 | 1.002008 | -0.1377 | 5.739859 | 3.46E-07 | 4.55E-06 | 6.342458 |
| FNDC1 | -2.42316 | 0.521639 | -5.73874 | 3.48E-07 | 4.57E-06 | 6.338357 |
| PILRA | -1.24607 | -0.06367 | -5.73851 | 3.48E-07 | 4.57E-06 | 6.337513 |
| PYDC1 | 1.372405 | 0.056018 | 5.738318 | 3.48E-07 | 4.57E-06 | 6.336802 |
| SCRT2 | 1.664354 | 0.02503 | 5.737929 | 3.49E-07 | 4.57E-06 | 6.335374 |
| LOC100131053 | -1.10029 | 0.058003 | -5.72809 | 3.62E-07 | 4.72E-06 | 6.299283 |
| SIX3 | 1.085224 | -0.17851 | 5.726818 | 3.64E-07 | 4.74E-06 | 6.294626 |
| ROPN1L | 1.107426 | 0.07719 | 5.723742 | 3.68E-07 | 4.79E-06 | 6.283345 |
| LY6G6F | 1.365487 | -0.21096 | 5.718116 | 3.76E-07 | 4.88E-06 | 6.262724 |
| CARD14 | 1.774817 | -0.37388 | 5.714801 | 3.81E-07 | 4.94E-06 | 6.250573 |
| CXorf48 | 1.296394 | -0.162 | 5.702942 | 3.98E-07 | 5.15E-06 | 6.207127 |
| IL6 | 1.336312 | -0.13518 | 5.700975 | 4.01E-07 | 5.18E-06 | 6.199924 |
| WFIKKN1 | -1.34172 | 0.286877 | -5.69901 | 4.04E-07 | 5.21E-06 | 6.192739 |
| RAB6B | -1.41673 | 0.099506 | -5.69892 | 4.05E-07 | 5.21E-06 | 6.192388 |
| SH3TC1 | -1.16272 | 0.055687 | -5.69302 | 4.14E-07 | 5.31E-06 | 6.170808 |
| SOX30 | 1.033196 | -0.25317 | 5.692576 | 4.14E-07 | 5.31E-06 | 6.169171 |
| TGFA | -1.09744 | 0.087947 | -5.6851 | 4.26E-07 | 5.45E-06 | 6.141798 |
| LINC00260 | -1.27417 | -0.11219 | -5.68404 | 4.28E-07 | 5.46E-06 | 6.137919 |
| HCN3 | -1.06256 | 0.027601 | -5.67911 | 4.36E-07 | 5.55E-06 | 6.119878 |
| SORCS1 | -1.49678 | 0.048753 | -5.67868 | 4.37E-07 | 5.55E-06 | 6.118321 |
| LRRN4 | 1.209227 | 0.028063 | 5.678598 | 4.37E-07 | 5.55E-06 | 6.11802 |
| NXF3 | 2.049225 | 0.175657 | 5.677057 | 4.39E-07 | 5.58E-06 | 6.112385 |
| CD300LG | 1.14235 | -0.05687 | 5.664261 | 4.61E-07 | 5.83E-06 | 6.065592 |
| C17orf110 | -1.52742 | -0.08679 | -5.66009 | 4.68E-07 | 5.91E-06 | 6.050331 |
| PTPRN | -1.20747 | 0.218073 | -5.6552 | 4.77E-07 | 6.00E-06 | 6.032478 |
| SLC22A15 | -1.21694 | -0.03105 | -5.6543 | 4.79E-07 | 6.01E-06 | 6.02919 |
| FAM153B | -2.33642 | -0.20233 | -5.6541 | 4.79E-07 | 6.01E-06 | 6.028453 |
| ATP2A3 | -1.13117 | -0.00134 | -5.65215 | 4.83E-07 | 6.03E-06 | 6.021345 |
| HOXA4 | -1.47071 | 0.010489 | -5.65214 | 4.83E-07 | 6.03E-06 | 6.021295 |
| EPS8L1 | -1.07043 | 0.205936 | -5.6488 | 4.89E-07 | 6.09E-06 | 6.0091 |
| TMCO5A | -1.44856 | 0.116996 | -5.64485 | 4.96E-07 | 6.16E-06 | 5.994667 |
| GP1BB | -1.25137 | -0.01971 | -5.64428 | 4.97E-07 | 6.17E-06 | 5.992598 |
| MARVELD3 | 1.944021 | -0.36263 | 5.644189 | 4.97E-07 | 6.17E-06 | 5.992256 |
| FUT9 | -1.42126 | -0.17129 | -5.63985 | 5.06E-07 | 6.25E-06 | 5.976418 |
| GNRH1 | -1.14364 | -0.06958 | -5.63983 | 5.06E-07 | 6.25E-06 | 5.976324 |
| SLC47A2 | -1.25563 | -0.06124 | -5.63698 | 5.11E-07 | 6.31E-06 | 5.965927 |
| HS3ST5 | -2.00701 | 0.354301 | -5.63666 | 5.12E-07 | 6.31E-06 | 5.964771 |
| LOC100288432 | 1.64316 | -0.56237 | 5.634678 | 5.16E-07 | 6.34E-06 | 5.957528 |
| PER1 | -1.17137 | 0.097792 | -5.63439 | 5.16E-07 | 6.34E-06 | 5.956463 |
| TBX3 | 1.906937 | -0.25212 | 5.632207 | 5.20E-07 | 6.39E-06 | 5.94851 |
| CHFR | -1.32549 | -0.10114 | -5.63198 | 5.21E-07 | 6.39E-06 | 5.9477 |
| FAM134B | 1.303358 | -0.07004 | 5.630769 | 5.23E-07 | 6.41E-06 | 5.943263 |
| ZNF415 | -1.96997 | -0.39272 | -5.6303 | 5.24E-07 | 6.42E-06 | 5.941546 |
| BMS1P1 | -1.22688 | -0.00073 | -5.62841 | 5.28E-07 | 6.46E-06 | 5.934652 |
| TAS2R9 | 1.060137 | 0.041047 | 5.62101 | 5.43E-07 | 6.60E-06 | 5.907657 |
| GCNT4 | -1.33079 | -0.24116 | -5.61684 | 5.51E-07 | 6.68E-06 | 5.892444 |
| FLJ12825 | 1.159622 | -0.06023 | 5.616574 | 5.52E-07 | 6.68E-06 | 5.89148 |
| KRT35 | 1.109843 | 0.157822 | 5.614919 | 5.55E-07 | 6.71E-06 | 5.885444 |
| FLJ42875 | -1.0863 | -0.15853 | -5.61409 | 5.57E-07 | 6.72E-06 | 5.882423 |
| SLC27A6 | -1.43197 | 0.339765 | -5.61086 | 5.64E-07 | 6.79E-06 | 5.87063 |
| ZNF595 | -1.26783 | -0.29173 | -5.60945 | 5.67E-07 | 6.81E-06 | 5.865499 |
| FOXD4 | 1.358246 | -0.17927 | 5.607499 | 5.71E-07 | 6.85E-06 | 5.858391 |
| SNORA70 | -1.26039 | -0.01598 | -5.60497 | 5.77E-07 | 6.90E-06 | 5.849169 |
| LRRC31 | 1.725729 | -0.24802 | 5.602375 | 5.82E-07 | 6.95E-06 | 5.839719 |
| CNDP1 | 3.035341 | 0.40929 | 5.597455 | 5.93E-07 | 7.06E-06 | 5.821789 |
| PAQR6 | -1.10418 | 0.037842 | -5.59327 | 6.03E-07 | 7.15E-06 | 5.806545 |
| BTN1A1 | 1.330341 | -0.04014 | 5.59116 | 6.07E-07 | 7.19E-06 | 5.798861 |
| C19orf59 | 1.797615 | -0.4799 | 5.588043 | 6.15E-07 | 7.27E-06 | 5.787511 |
| CLEC18B | -1.35507 | -0.26782 | -5.5849 | 6.22E-07 | 7.34E-06 | 5.776049 |
| GRIA3 | -1.24073 | 0.077344 | -5.58421 | 6.24E-07 | 7.36E-06 | 5.773571 |
| ASPG | -1.80035 | 0.105407 | -5.58371 | 6.25E-07 | 7.37E-06 | 5.771745 |
| KIF1A | -1.12231 | 0.24936 | -5.58361 | 6.25E-07 | 7.37E-06 | 5.77136 |
| METTL7A | -1.02811 | 0.1034 | -5.58284 | 6.27E-07 | 7.38E-06 | 5.768561 |
| MUC3 | 1.074916 | -0.08727 | 5.579887 | 6.34E-07 | 7.44E-06 | 5.757816 |
| ENTPD5 | -1.23387 | 0.074897 | -5.57068 | 6.56E-07 | 7.68E-06 | 5.724299 |
| NACAP1 | 1.903588 | 0.166309 | 5.570379 | 6.57E-07 | 7.69E-06 | 5.72322 |
| ALDH3A1 | -1.9179 | -0.10322 | -5.56406 | 6.73E-07 | 7.84E-06 | 5.700239 |
| STOX2 | -1.19622 | -0.18988 | -5.5631 | 6.75E-07 | 7.86E-06 | 5.696736 |
| ZNF683 | -1.36869 | 0.277278 | -5.55786 | 6.88E-07 | 8.00E-06 | 5.677705 |
| KCTD14 | -1.06706 | 0.060742 | -5.55399 | 6.99E-07 | 8.11E-06 | 5.663616 |
| DPCR1 | -2.45789 | -0.11225 | -5.55141 | 7.05E-07 | 8.16E-06 | 5.654258 |
| DSCAML1 | -1.72051 | -0.04544 | -5.54318 | 7.28E-07 | 8.39E-06 | 5.624351 |
| MUSTN1 | -1.19697 | 0.008361 | -5.54001 | 7.36E-07 | 8.47E-06 | 5.612833 |
| NKD2 | 1.011503 | 0.052401 | 5.534442 | 7.52E-07 | 8.62E-06 | 5.592609 |
| HOXA6 | 1.008249 | -0.01093 | 5.53341 | 7.55E-07 | 8.65E-06 | 5.588863 |
| C15orf26 | 1.154684 | -0.14208 | 5.530113 | 7.64E-07 | 8.73E-06 | 5.576892 |
| NKAIN2 | 1.039267 | -0.09764 | 5.529342 | 7.66E-07 | 8.75E-06 | 5.574096 |
| CTSE | -1.30188 | -0.04358 | -5.52797 | 7.70E-07 | 8.79E-06 | 5.569103 |
| SPAG5-AS1 | -1.46536 | 0.209664 | -5.52033 | 7.93E-07 | 9.02E-06 | 5.541402 |
| GPRC5B | -1.565 | -0.29384 | -5.51722 | 8.02E-07 | 9.11E-06 | 5.530099 |
| NPY6R | 1.951054 | 0.288569 | 5.516992 | 8.03E-07 | 9.11E-06 | 5.529279 |
| NR4A1 | 1.893398 | -0.21297 | 5.505805 | 8.37E-07 | 9.45E-06 | 5.488714 |
| MYOM3 | 1.545061 | -0.07892 | 5.504517 | 8.41E-07 | 9.49E-06 | 5.484047 |
| OR52K2 | 1.078409 | -0.01724 | 5.497737 | 8.63E-07 | 9.71E-06 | 5.459474 |
| BTNL9 | 1.034557 | 0.084615 | 5.486918 | 8.98E-07 | 1.01E-05 | 5.420282 |
| SCG3 | -1.86234 | 0.109302 | -5.48668 | 8.99E-07 | 1.01E-05 | 5.419436 |
| SHISA6 | -2.67725 | 0.044519 | -5.48664 | 8.99E-07 | 1.01E-05 | 5.419293 |
| CALY | -1.47795 | 0.07712 | -5.48621 | 9.01E-07 | 1.01E-05 | 5.417702 |
| FLJ35390 | 1.190065 | 0.160568 | 5.479046 | 9.25E-07 | 1.03E-05 | 5.391783 |
| EDN1 | 1.617917 | -0.28304 | 5.47504 | 9.39E-07 | 1.04E-05 | 5.377285 |
| OSR2 | 2.351474 | -0.36367 | 5.474275 | 9.42E-07 | 1.05E-05 | 5.374517 |
| GPR64 | -1.60279 | 0.107838 | -5.47221 | 9.49E-07 | 1.05E-05 | 5.367037 |
| STRC | -1.11722 | 0.036601 | -5.47178 | 9.51E-07 | 1.05E-05 | 5.365491 |
| RAB27A | -1.52936 | 0.061795 | -5.4682 | 9.64E-07 | 1.06E-05 | 5.35255 |
| PEG3-AS1 | -1.54637 | 0.074451 | -5.46681 | 9.69E-07 | 1.07E-05 | 5.347525 |
| THBS2 | 2.503783 | 0.469432 | 5.462892 | 9.83E-07 | 1.08E-05 | 5.333339 |
| GALC | 1.376847 | 0.238177 | 5.462762 | 9.83E-07 | 1.08E-05 | 5.33287 |
| PDE6B | -1.16494 | -0.26248 | -5.46145 | 9.88E-07 | 1.08E-05 | 5.328134 |
| THEM4 | 1.100268 | -0.02938 | 5.452803 | 1.02E-06 | 1.11E-05 | 5.296863 |
| KIAA0408 | -1.83874 | -0.12331 | -5.4526 | 1.02E-06 | 1.11E-05 | 5.296136 |
| P2RY2 | 1.659698 | -0.31693 | 5.449614 | 1.03E-06 | 1.13E-05 | 5.285338 |
| ZNF512B | -1.20504 | -0.34163 | -5.44496 | 1.05E-06 | 1.14E-05 | 5.268534 |
| SLCO4A1 | -1.28676 | -0.04117 | -5.44203 | 1.06E-06 | 1.15E-05 | 5.257938 |
| OR1S2 | 1.180525 | -0.19736 | 5.436616 | 1.08E-06 | 1.17E-05 | 5.238391 |
| NUBPL | 1.286595 | 0.132189 | 5.433501 | 1.10E-06 | 1.19E-05 | 5.227143 |
| STK32B | -1.59331 | 0.067182 | -5.42949 | 1.11E-06 | 1.20E-05 | 5.212679 |
| COL11A2 | -1.97561 | 0.231185 | -5.42946 | 1.11E-06 | 1.20E-05 | 5.21255 |
| MALAT1 | -1.07843 | -0.19408 | -5.42888 | 1.12E-06 | 1.20E-05 | 5.210456 |
| NOS1AP | -1.14284 | 0.08365 | -5.42747 | 1.12E-06 | 1.21E-05 | 5.205363 |
| ASS1 | 1.57518 | -0.08976 | 5.424763 | 1.13E-06 | 1.22E-05 | 5.195608 |
| NOS2 | 3.544233 | 0.15203 | 5.423358 | 1.14E-06 | 1.22E-05 | 5.190535 |
| HPSE | 1.167346 | -0.1012 | 5.421463 | 1.15E-06 | 1.23E-05 | 5.183701 |
| TFF3 | 2.316644 | -0.47483 | 5.420396 | 1.15E-06 | 1.24E-05 | 5.179849 |
| GPR128 | 3.792799 | -0.72801 | 5.416082 | 1.17E-06 | 1.25E-05 | 5.164291 |
| FRMPD3 | 1.042945 | -0.12129 | 5.415407 | 1.17E-06 | 1.26E-05 | 5.161858 |
| ITGB8 | 1.057754 | -0.14506 | 5.411198 | 1.19E-06 | 1.27E-05 | 5.146679 |
| SYCP2 | -1.77971 | -0.10664 | -5.41012 | 1.20E-06 | 1.28E-05 | 5.142808 |
| LOC645249 | 1.546428 | -0.03132 | 5.409352 | 1.20E-06 | 1.28E-05 | 5.140024 |
| ZNF345 | -1.12487 | -0.13106 | -5.40492 | 1.22E-06 | 1.30E-05 | 5.124044 |
| S100A5 | 1.513542 | 0.049286 | 5.400188 | 1.24E-06 | 1.32E-05 | 5.106998 |
| ZNF334 | -1.98841 | -0.33767 | -5.40004 | 1.24E-06 | 1.32E-05 | 5.10647 |
| SLC6A19 | 1.303418 | -0.11097 | 5.397657 | 1.25E-06 | 1.33E-05 | 5.097882 |
| SVOP | -2.08056 | -0.07146 | -5.39481 | 1.27E-06 | 1.34E-05 | 5.087643 |
| WNT11 | 1.110564 | 0.151343 | 5.391301 | 1.28E-06 | 1.35E-05 | 5.074988 |
| LOC100506310 | -1.37342 | -0.11209 | -5.39037 | 1.29E-06 | 1.35E-05 | 5.071644 |
| DISP1 | -1.28161 | 0.164211 | -5.38901 | 1.30E-06 | 1.36E-05 | 5.066722 |
| NHS | 1.040328 | -0.14476 | 5.386557 | 1.31E-06 | 1.37E-05 | 5.057907 |
| C4orf34 | -1.22859 | 0.019567 | -5.38556 | 1.31E-06 | 1.37E-05 | 5.054317 |
| GATA4 | -1.25344 | 0.07731 | -5.38373 | 1.32E-06 | 1.38E-05 | 5.04773 |
| PHYHD1 | -1.59796 | -0.30909 | -5.38339 | 1.32E-06 | 1.38E-05 | 5.046517 |
| SLC38A1 | 1.512488 | -0.46578 | 5.38329 | 1.32E-06 | 1.38E-05 | 5.046147 |
| EPDR1 | -2.20443 | -0.0412 | -5.38052 | 1.34E-06 | 1.39E-05 | 5.036179 |
| C16orf73 | 1.156343 | 0.131209 | 5.37418 | 1.37E-06 | 1.42E-05 | 5.01337 |
| AQPEP | 1.370685 | -0.0784 | 5.371225 | 1.38E-06 | 1.43E-05 | 5.002738 |
| ANKRD7 | 1.078382 | -0.34345 | 5.36813 | 1.40E-06 | 1.44E-05 | 4.991608 |
| LPO | 1.369931 | -0.12144 | 5.367677 | 1.40E-06 | 1.44E-05 | 4.989981 |
| GPBAR1 | -1.39283 | 0.252093 | -5.35958 | 1.45E-06 | 1.48E-05 | 4.960877 |
| TSHZ2 | 1.004555 | -0.01269 | 5.357534 | 1.46E-06 | 1.49E-05 | 4.953521 |
| PYCARD | 1.062392 | -0.07095 | 5.355768 | 1.47E-06 | 1.50E-05 | 4.947175 |
| ANKRD60 | 1.451316 | 0.090704 | 5.349791 | 1.50E-06 | 1.53E-05 | 4.925701 |
| SPAM1 | 1.266444 | 0.070849 | 5.349579 | 1.50E-06 | 1.53E-05 | 4.924939 |
| OLFM4 | 5.109415 | -1.37959 | 5.348809 | 1.50E-06 | 1.53E-05 | 4.922174 |
| RPGR | -1.11977 | 0.05071 | -5.34816 | 1.51E-06 | 1.54E-05 | 4.919837 |
| TMEM206 | 1.036067 | -0.05612 | 5.346945 | 1.51E-06 | 1.54E-05 | 4.915479 |
| NLRP12 | 1.102419 | -0.32275 | 5.346214 | 1.52E-06 | 1.54E-05 | 4.912854 |
| BAAT | 1.526177 | -0.09404 | 5.343515 | 1.53E-06 | 1.56E-05 | 4.903163 |
| OR10A5 | 1.150305 | -0.02468 | 5.343099 | 1.54E-06 | 1.56E-05 | 4.901669 |
| ZNF347 | -1.74163 | -0.59493 | -5.34024 | 1.55E-06 | 1.57E-05 | 4.891421 |
| OBSL1 | -1.16174 | -0.1518 | -5.34016 | 1.55E-06 | 1.57E-05 | 4.891119 |
| KIFC2 | -1.19333 | -0.14316 | -5.33299 | 1.60E-06 | 1.61E-05 | 4.8654 |
| EEF1A2 | -2.39524 | 0.116897 | -5.32043 | 1.67E-06 | 1.67E-05 | 4.820324 |
| CD52 | -1.04186 | -0.1252 | -5.31325 | 1.72E-06 | 1.71E-05 | 4.794591 |
| VLDLR | -1.40349 | -0.1939 | -5.3114 | 1.73E-06 | 1.72E-05 | 4.787984 |
| GABRD | 1.06556 | -0.12084 | 5.310037 | 1.74E-06 | 1.73E-05 | 4.783091 |
| SLC32A1 | 1.160758 | 0.030285 | 5.301315 | 1.79E-06 | 1.78E-05 | 4.751851 |
| MUC6 | -1.4526 | 0.096093 | -5.29953 | 1.81E-06 | 1.79E-05 | 4.745455 |
| LIPG | 1.693842 | -0.07794 | 5.296366 | 1.83E-06 | 1.81E-05 | 4.734135 |
| HOXB5 | 1.907462 | -0.14419 | 5.291876 | 1.86E-06 | 1.83E-05 | 4.718063 |
| RAX | 1.64049 | -0.07286 | 5.286342 | 1.90E-06 | 1.86E-05 | 4.698264 |
| AVPR1B | 1.038739 | -0.08006 | 5.285683 | 1.90E-06 | 1.87E-05 | 4.695908 |
| FJX1 | 1.07213 | -0.24261 | 5.284969 | 1.91E-06 | 1.87E-05 | 4.693352 |
| ZNF256 | -1.19414 | -0.34918 | -5.28396 | 1.91E-06 | 1.87E-05 | 4.689749 |
| SPNS1 | -1.48286 | -0.22297 | -5.28341 | 1.92E-06 | 1.88E-05 | 4.687762 |
| APOBEC3G | -1.05174 | -0.15233 | -5.28291 | 1.92E-06 | 1.88E-05 | 4.685979 |
| CSTA | -2.04056 | -0.22676 | -5.28254 | 1.92E-06 | 1.88E-05 | 4.684658 |
| MB21D2 | -1.35328 | 0.145775 | -5.27483 | 1.98E-06 | 1.93E-05 | 4.6571 |
| MYO3A | 1.46246 | -0.01211 | 5.27326 | 1.99E-06 | 1.94E-05 | 4.651487 |
| ZNF479 | 1.149844 | 0.183831 | 5.269021 | 2.02E-06 | 1.96E-05 | 4.636339 |
| BHMT | -2.13444 | -0.11064 | -5.26778 | 2.03E-06 | 1.97E-05 | 4.631899 |
| TAS2R40 | 1.493373 | 0.226266 | 5.267341 | 2.04E-06 | 1.97E-05 | 4.630333 |
| CLDN1 | 2.277276 | -0.6278 | 5.259105 | 2.10E-06 | 2.02E-05 | 4.600917 |
| RNASE4 | -1.31472 | 0.218408 | -5.25283 | 2.15E-06 | 2.06E-05 | 4.578516 |
| MAL | -3.52707 | -0.79569 | -5.25155 | 2.16E-06 | 2.07E-05 | 4.573933 |
| ITLN2 | 3.065027 | 0.973678 | 5.247566 | 2.19E-06 | 2.10E-05 | 4.55973 |
| HAGHL | 1.268512 | 0.055499 | 5.245549 | 2.21E-06 | 2.11E-05 | 4.552533 |
| SULT1E1 | 3.027189 | -0.22451 | 5.235438 | 2.29E-06 | 2.18E-05 | 4.516472 |
| LOC730227 | 1.581842 | 0.11196 | 5.232422 | 2.32E-06 | 2.20E-05 | 4.50572 |
| DERL3 | -1.47757 | 0.182374 | -5.23212 | 2.32E-06 | 2.20E-05 | 4.504654 |
| OR10H1 | 1.280396 | 0.186157 | 5.228901 | 2.35E-06 | 2.22E-05 | 4.493172 |
| PAK3 | -1.37282 | 0.471185 | -5.22626 | 2.37E-06 | 2.24E-05 | 4.483763 |
| LOC221442 | -1.34055 | -0.16268 | -5.22546 | 2.38E-06 | 2.25E-05 | 4.480909 |
| PABPC3 | 2.06682 | -0.30863 | 5.224637 | 2.38E-06 | 2.25E-05 | 4.477978 |
| SNRPN | -1.28203 | -0.34996 | -5.22318 | 2.40E-06 | 2.26E-05 | 4.472806 |
| LCE3A | 1.050551 | 0.032412 | 5.218717 | 2.44E-06 | 2.29E-05 | 4.456893 |
| ONECUT2 | 2.596044 | -0.56958 | 5.217951 | 2.44E-06 | 2.29E-05 | 4.454167 |
| RAB24 | -1.168 | -0.20512 | -5.20708 | 2.54E-06 | 2.37E-05 | 4.41547 |
| TUSC5 | 1.091448 | -0.01809 | 5.206349 | 2.55E-06 | 2.38E-05 | 4.412868 |
| ST6GALNAC5 | 1.101222 | -0.06559 | 5.205432 | 2.56E-06 | 2.38E-05 | 4.409606 |
| POU2AF1 | -1.304 | -0.43332 | -5.20497 | 2.56E-06 | 2.39E-05 | 4.407971 |
| PPP1R32 | -1.11539 | 0.08499 | -5.19704 | 2.64E-06 | 2.45E-05 | 4.37975 |
| TAAR1 | -1.40284 | 0.372555 | -5.1964 | 2.65E-06 | 2.45E-05 | 4.377476 |
| CSPG4 | -1.49045 | -0.18259 | -5.19582 | 2.65E-06 | 2.45E-05 | 4.3754 |
| REEP6 | 1.369648 | 0.272047 | 5.192696 | 2.68E-06 | 2.48E-05 | 4.36431 |
| ACTL7A | 1.009217 | -0.18738 | 5.191635 | 2.69E-06 | 2.49E-05 | 4.360539 |
| FTHL17 | 1.048202 | -0.19675 | 5.190969 | 2.70E-06 | 2.49E-05 | 4.358173 |
| PTPRN2 | -1.10167 | -0.03356 | -5.19012 | 2.71E-06 | 2.50E-05 | 4.355161 |
| UPK1B | -3.18167 | -0.54265 | -5.18381 | 2.77E-06 | 2.55E-05 | 4.332732 |
| TMEM176B | 1.294427 | -0.21231 | 5.177274 | 2.84E-06 | 2.60E-05 | 4.309519 |
| LINC00261 | -1.19773 | -0.00827 | -5.17674 | 2.84E-06 | 2.61E-05 | 4.307624 |
| C6orf123 | 1.172483 | -0.07227 | 5.175113 | 2.86E-06 | 2.62E-05 | 4.301847 |
| KLK10 | 2.991134 | 0.458804 | 5.174213 | 2.87E-06 | 2.63E-05 | 4.298652 |
| LRMP | -1.53935 | 0.2595 | -5.16976 | 2.92E-06 | 2.67E-05 | 4.282843 |
| CSTL1 | 1.175472 | 0.08987 | 5.1672 | 2.95E-06 | 2.69E-05 | 4.273759 |
| TMEM176A | 1.377264 | -0.22911 | 5.167181 | 2.95E-06 | 2.69E-05 | 4.273693 |
| CLDN14 | 1.662579 | -0.20859 | 5.166481 | 2.95E-06 | 2.69E-05 | 4.271208 |
| VAV3 | 2.005389 | -0.36662 | 5.1635 | 2.99E-06 | 2.72E-05 | 4.260634 |
| FAM84A | 1.762464 | 0.300666 | 5.163361 | 2.99E-06 | 2.72E-05 | 4.260139 |
| PCDHB8 | 1.130563 | -0.03586 | 5.162253 | 3.00E-06 | 2.73E-05 | 4.256211 |
| SPAG17 | -2.6687 | 0.635104 | -5.15875 | 3.04E-06 | 2.76E-05 | 4.243785 |
| FOSL1 | 2.009776 | -0.08491 | 5.156892 | 3.06E-06 | 2.77E-05 | 4.237197 |
| PIWIL2 | -1.29652 | 0.103835 | -5.15541 | 3.08E-06 | 2.78E-05 | 4.231955 |
| FHAD1 | -1.43455 | 0.310385 | -5.15345 | 3.10E-06 | 2.80E-05 | 4.224998 |
| ZNF300 | -1.31621 | -0.32586 | -5.15298 | 3.10E-06 | 2.80E-05 | 4.223333 |
| CLDN18 | -1.33043 | -0.30272 | -5.14303 | 3.22E-06 | 2.89E-05 | 4.188073 |
| SERPINE1 | 1.047355 | -0.23583 | 5.142132 | 3.23E-06 | 2.90E-05 | 4.184889 |
| SUSD4 | -1.54768 | -0.19053 | -5.13882 | 3.27E-06 | 2.93E-05 | 4.173166 |
| KRT28 | 1.01556 | 0.014778 | 5.136503 | 3.30E-06 | 2.95E-05 | 4.164954 |
| LRRC19 | 2.608019 | 0.46095 | 5.123089 | 3.46E-06 | 3.08E-05 | 4.117482 |
| BTBD19 | 1.063141 | -0.0625 | 5.122684 | 3.47E-06 | 3.09E-05 | 4.11605 |
| LOC439990 | 1.233892 | -0.07187 | 5.114554 | 3.57E-06 | 3.16E-05 | 4.0873 |
| EPHB6 | -1.71188 | -0.18637 | -5.11214 | 3.61E-06 | 3.18E-05 | 4.078772 |
| MST1P2 | -1.62616 | 0.107415 | -5.10746 | 3.67E-06 | 3.23E-05 | 4.062231 |
| GLI2 | -1.41244 | -0.01146 | -5.10627 | 3.68E-06 | 3.24E-05 | 4.058028 |
| HOXB6 | 1.915562 | -0.24366 | 5.102629 | 3.73E-06 | 3.27E-05 | 4.045164 |
| CLCA3P | 1.30723 | -0.03503 | 5.100362 | 3.77E-06 | 3.29E-05 | 4.037158 |
| HNRNPCL1 | 1.105145 | 0.378936 | 5.097429 | 3.81E-06 | 3.32E-05 | 4.026802 |
| PIP | 1.589779 | -0.3966 | 5.093798 | 3.86E-06 | 3.35E-05 | 4.013984 |
| MIR22HG | -1.11332 | -0.00301 | -5.09344 | 3.86E-06 | 3.35E-05 | 4.012712 |
| AKAP14 | 1.420114 | 0.153997 | 5.092855 | 3.87E-06 | 3.36E-05 | 4.010656 |
| C8orf47 | -1.26317 | -0.00722 | -5.09154 | 3.89E-06 | 3.37E-05 | 4.006006 |
| ST6GALNAC4 | 1.061355 | -0.15264 | 5.08589 | 3.97E-06 | 3.42E-05 | 3.986081 |
| LOC100289058 | -1.15536 | 0.066354 | -5.08535 | 3.98E-06 | 3.42E-05 | 3.984166 |
| CEACAM4 | 1.163941 | -0.27599 | 5.082482 | 4.02E-06 | 3.45E-05 | 3.974063 |
| CADM1 | -1.15524 | -0.24166 | -5.07454 | 4.14E-06 | 3.55E-05 | 3.946059 |
| PPP2R3A | -1.4748 | 0.059329 | -5.07181 | 4.18E-06 | 3.57E-05 | 3.936456 |
| CRYGB | -1.41436 | 0.398481 | -5.06971 | 4.21E-06 | 3.60E-05 | 3.929029 |
| GREB1L | -1.31161 | 0.133604 | -5.06918 | 4.22E-06 | 3.60E-05 | 3.927176 |
| LOC100290566 | 1.10669 | -0.0961 | 5.067325 | 4.25E-06 | 3.62E-05 | 3.92064 |
| LOC100128180 | -1.15937 | -0.08606 | -5.05625 | 4.42E-06 | 3.75E-05 | 3.881656 |
| EPHB1 | 2.192989 | 0.402681 | 5.055628 | 4.44E-06 | 3.76E-05 | 3.879452 |
| ANXA9 | 1.744382 | 0.174537 | 5.053892 | 4.46E-06 | 3.77E-05 | 3.873345 |
| ARSF | 1.043692 | -0.09711 | 5.052815 | 4.48E-06 | 3.78E-05 | 3.869556 |
| CDH15 | 2.181769 | 0.072234 | 5.051648 | 4.50E-06 | 3.79E-05 | 3.865449 |
| NLRP5 | 1.532341 | 0.178121 | 5.050994 | 4.51E-06 | 3.80E-05 | 3.863146 |
| ZMAT1 | -1.38824 | -0.13911 | -5.04206 | 4.66E-06 | 3.91E-05 | 3.831723 |
| OBP2B | 1.32459 | 0.095689 | 5.041414 | 4.67E-06 | 3.92E-05 | 3.829456 |
| MFSD7 | -1.15509 | -0.2452 | -5.03861 | 4.72E-06 | 3.95E-05 | 3.819601 |
| CDH9 | 1.106989 | -0.17297 | 5.030443 | 4.86E-06 | 4.05E-05 | 3.7909 |
| SLC30A10 | 1.521456 | 0.075713 | 5.030301 | 4.86E-06 | 4.05E-05 | 3.790402 |
| ATOH1 | 1.145481 | 0.122433 | 5.028672 | 4.89E-06 | 4.07E-05 | 3.784679 |
| CCND2 | 2.059544 | 0.200053 | 5.024253 | 4.97E-06 | 4.14E-05 | 3.769161 |
| RNASE1 | -1.2511 | -0.17968 | -5.02329 | 4.99E-06 | 4.15E-05 | 3.76577 |
| AHSA2 | -1.41293 | -0.06476 | -5.0222 | 5.01E-06 | 4.16E-05 | 3.761967 |
| SLC16A2 | 1.404468 | 0.157543 | 5.018786 | 5.07E-06 | 4.21E-05 | 3.749972 |
| ADH6 | 2.226229 | 0.122913 | 5.015482 | 5.13E-06 | 4.25E-05 | 3.738376 |
| CXCL1 | 2.926134 | -0.54306 | 5.001708 | 5.40E-06 | 4.45E-05 | 3.690076 |
| EPHB2 | 2.299406 | -0.13753 | 5.001141 | 5.41E-06 | 4.45E-05 | 3.688087 |
| LOC100505657 | -1.28486 | -0.03467 | -5.00027 | 5.43E-06 | 4.46E-05 | 3.685021 |
| TIMP4 | 1.060936 | 0.024537 | 4.993717 | 5.56E-06 | 4.56E-05 | 3.662078 |
| SCG5 | -1.78385 | 0.033465 | -4.99325 | 5.57E-06 | 4.56E-05 | 3.660437 |
| ST3GAL5 | -1.06997 | -0.16888 | -4.99021 | 5.63E-06 | 4.61E-05 | 3.649797 |
| PAX9 | 2.015761 | 0.555286 | 4.989955 | 5.64E-06 | 4.61E-05 | 3.648901 |
| NPHS2 | 1.516196 | -0.11597 | 4.983862 | 5.76E-06 | 4.70E-05 | 3.627573 |
| GPR78 | 1.097724 | -0.05581 | 4.983202 | 5.78E-06 | 4.71E-05 | 3.625262 |
| C5orf62 | 1.260715 | -0.19405 | 4.982645 | 5.79E-06 | 4.72E-05 | 3.623314 |
| CWH43 | -4.23605 | -1.39414 | -4.98202 | 5.80E-06 | 4.72E-05 | 3.621141 |
| ABCG1 | -1.08003 | -0.06956 | -4.97729 | 5.90E-06 | 4.79E-05 | 3.604561 |
| GPR27 | -1.49483 | 0.007831 | -4.97507 | 5.95E-06 | 4.82E-05 | 3.59683 |
| C2 | 1.505989 | -0.28331 | 4.974437 | 5.96E-06 | 4.83E-05 | 3.594598 |
| MT1M | 1.526705 | 0.134241 | 4.97164 | 6.02E-06 | 4.88E-05 | 3.584819 |
| PRAME | 1.266134 | 0.133784 | 4.96783 | 6.11E-06 | 4.94E-05 | 3.571498 |
| SPTSSB | -2.23503 | -0.32097 | -4.96022 | 6.28E-06 | 5.05E-05 | 3.544915 |
| FADS6 | 1.318611 | -0.04632 | 4.959895 | 6.29E-06 | 5.05E-05 | 3.543772 |
| SPRR2G | 1.077475 | 0.201671 | 4.958451 | 6.32E-06 | 5.07E-05 | 3.538726 |
| SLC18A3 | 1.19249 | 0.101872 | 4.956956 | 6.35E-06 | 5.10E-05 | 3.533505 |
| LOC727820 | -1.03763 | -0.17332 | -4.95581 | 6.38E-06 | 5.11E-05 | 3.529505 |
| LOC595101 | -1.35966 | -0.06603 | -4.95399 | 6.42E-06 | 5.14E-05 | 3.523152 |
| CA9 | -1.61135 | 0.259861 | -4.95182 | 6.47E-06 | 5.18E-05 | 3.515589 |
| POLR3G | 1.214069 | -0.01554 | 4.949338 | 6.53E-06 | 5.21E-05 | 3.506909 |
| RAB7B | -1.14834 | -0.04486 | -4.94497 | 6.64E-06 | 5.27E-05 | 3.491651 |
| GCKR | -1.26721 | 0.040051 | -4.93821 | 6.80E-06 | 5.38E-05 | 3.468095 |
| IL36G | 1.148688 | -0.28612 | 4.936752 | 6.84E-06 | 5.40E-05 | 3.463007 |
| SCGB2A1 | -2.09552 | -0.35612 | -4.93362 | 6.91E-06 | 5.45E-05 | 3.45207 |
| ZNF577 | -1.13151 | -0.06418 | -4.93296 | 6.93E-06 | 5.46E-05 | 3.449793 |
| HDGFRP3 | -1.04179 | -0.22573 | -4.92727 | 7.08E-06 | 5.56E-05 | 3.429972 |
| FAM105A | 1.154717 | -0.09432 | 4.926802 | 7.09E-06 | 5.56E-05 | 3.428327 |
| LOC100507280 | -1.19124 | -0.14046 | -4.92657 | 7.09E-06 | 5.56E-05 | 3.427516 |
| ITIH4 | -1.01662 | -0.07538 | -4.92541 | 7.12E-06 | 5.58E-05 | 3.423481 |
| SOSTDC1 | -2.1923 | -0.16526 | -4.92309 | 7.18E-06 | 5.62E-05 | 3.415407 |
| LCAT | -1.12014 | -0.07586 | -4.92204 | 7.21E-06 | 5.64E-05 | 3.411744 |
| GJD2 | 1.125141 | -0.04383 | 4.920332 | 7.26E-06 | 5.66E-05 | 3.405796 |
| EMR1 | 1.36804 | -0.21012 | 4.917505 | 7.33E-06 | 5.70E-05 | 3.395953 |
| MPPED2 | -1.13812 | 0.234413 | -4.91625 | 7.36E-06 | 5.73E-05 | 3.391588 |
| LRRC25 | 1.008228 | -0.32501 | 4.915699 | 7.38E-06 | 5.73E-05 | 3.389667 |
| PLA2G7 | 1.810346 | -0.69638 | 4.914596 | 7.41E-06 | 5.75E-05 | 3.385826 |
| ZNF528 | -1.59662 | -0.20168 | -4.9127 | 7.46E-06 | 5.79E-05 | 3.379223 |
| CAPN3 | -1.03032 | -0.0139 | -4.91165 | 7.49E-06 | 5.81E-05 | 3.375561 |
| SPRN | 1.209371 | -0.16188 | 4.910916 | 7.51E-06 | 5.82E-05 | 3.37302 |
| CDC42EP2 | 1.086829 | -0.22595 | 4.906797 | 7.62E-06 | 5.90E-05 | 3.358694 |
| NCKAP5 | -1.32573 | -0.14377 | -4.89889 | 7.84E-06 | 6.04E-05 | 3.331212 |
| PPY2 | 1.271531 | -0.1264 | 4.896953 | 7.90E-06 | 6.07E-05 | 3.324471 |
| NFE4 | 1.313393 | -0.14621 | 4.893807 | 7.99E-06 | 6.13E-05 | 3.313538 |
| LOC100652730 | 2.975825 | -1.00196 | 4.8912 | 8.06E-06 | 6.18E-05 | 3.304482 |
| KCNJ15 | -2.16853 | -0.25174 | -4.89019 | 8.09E-06 | 6.20E-05 | 3.300973 |
| SLCO4C1 | -1.58178 | 0.205859 | -4.88821 | 8.15E-06 | 6.23E-05 | 3.294114 |
| CLU | -1.45188 | -0.06244 | -4.88227 | 8.33E-06 | 6.36E-05 | 3.273466 |
| NKX6-2 | -3.58918 | -1.14234 | -4.87908 | 8.42E-06 | 6.41E-05 | 3.262395 |
| PIAS4 | 1.029391 | 0.397296 | 4.878663 | 8.43E-06 | 6.42E-05 | 3.260962 |
| JPH1 | 1.232067 | -0.05824 | 4.876092 | 8.51E-06 | 6.47E-05 | 3.252039 |
| TPSD1 | 1.360352 | -0.42695 | 4.875078 | 8.54E-06 | 6.49E-05 | 3.248524 |
| LOC100505592 | 1.608426 | -0.36501 | 4.874108 | 8.57E-06 | 6.51E-05 | 3.245159 |
| FOXC1 | 1.816816 | -0.14984 | 4.866452 | 8.81E-06 | 6.66E-05 | 3.218614 |
| CDH16 | 2.772378 | -0.26938 | 4.865561 | 8.84E-06 | 6.67E-05 | 3.215525 |
| KRT80 | 1.537684 | -0.23604 | 4.864941 | 8.86E-06 | 6.68E-05 | 3.213379 |
| COMP | 1.060655 | -0.20385 | 4.864466 | 8.88E-06 | 6.69E-05 | 3.21173 |
| DAGLA | 1.773345 | -0.2274 | 4.863098 | 8.92E-06 | 6.72E-05 | 3.206992 |
| ARSJ | -1.20471 | -0.12142 | -4.85747 | 9.10E-06 | 6.82E-05 | 3.187503 |
| LOC344887 | -1.43612 | 0.155427 | -4.84838 | 9.41E-06 | 7.01E-05 | 3.156025 |
| SLC3A1 | 2.669226 | -0.19879 | 4.8451 | 9.52E-06 | 7.08E-05 | 3.144672 |
| FGF4 | 1.3456 | -0.26023 | 4.844718 | 9.53E-06 | 7.09E-05 | 3.143351 |
| LOC643037 | 1.91422 | 0.04946 | 4.842783 | 9.60E-06 | 7.12E-05 | 3.136658 |
| KCNH7 | -1.14224 | 0.320883 | -4.83968 | 9.71E-06 | 7.19E-05 | 3.125941 |
| SLC5A5 | -1.67024 | -0.26064 | -4.83577 | 9.85E-06 | 7.28E-05 | 3.112413 |
| LEFTY1 | 4.142595 | 0.331359 | 4.83569 | 9.85E-06 | 7.28E-05 | 3.112131 |
| KLK7 | 3.658962 | -0.21937 | 4.832186 | 9.97E-06 | 7.35E-05 | 3.10002 |
| MEX3A | 1.622391 | 0.20619 | 4.818957 | 1.05E-05 | 7.65E-05 | 3.05433 |
| DOK5 | 1.43365 | -0.12475 | 4.812701 | 1.07E-05 | 7.81E-05 | 3.03274 |
| FAM13A-AS1 | -1.15543 | -0.06387 | -4.8113 | 1.08E-05 | 7.84E-05 | 3.027919 |
| SHBG | 1.075712 | -0.00175 | 4.810401 | 1.08E-05 | 7.87E-05 | 3.024805 |
| LOC728537 | -1.02466 | 0.009145 | -4.80316 | 1.11E-05 | 8.04E-05 | 2.999836 |
| FLJ43489 | -1.3286 | -0.10846 | -4.80077 | 1.12E-05 | 8.10E-05 | 2.991599 |
| SCGN | -1.61049 | -0.00465 | -4.79956 | 1.12E-05 | 8.14E-05 | 2.987417 |
| ARG2 | 1.21315 | -0.18115 | 4.796504 | 1.13E-05 | 8.22E-05 | 2.976901 |
| C19orf6 | -1.41629 | -0.03087 | -4.79215 | 1.15E-05 | 8.33E-05 | 2.961909 |
| LGALS7 | 1.401311 | 0.29307 | 4.790723 | 1.16E-05 | 8.36E-05 | 2.956993 |
| ZNF549 | -1.33597 | -0.27928 | -4.78803 | 1.17E-05 | 8.44E-05 | 2.94773 |
| ACSM1 | -1.17307 | 0.036217 | -4.78543 | 1.18E-05 | 8.50E-05 | 2.93878 |
| SLC17A3 | 1.180061 | -0.09054 | 4.779056 | 1.21E-05 | 8.69E-05 | 2.916843 |
| PRDM5 | -1.31935 | -0.24201 | -4.7789 | 1.21E-05 | 8.69E-05 | 2.916295 |
| IL36B | 1.063702 | 0.025106 | 4.773335 | 1.23E-05 | 8.85E-05 | 2.897171 |
| MIR143HG | -1.90372 | -0.12178 | -4.77233 | 1.24E-05 | 8.87E-05 | 2.893707 |
| KIF5C | -1.54306 | -0.22601 | -4.77082 | 1.24E-05 | 8.91E-05 | 2.88853 |
| IFNE | -1.35678 | 0.134038 | -4.75718 | 1.31E-05 | 9.27E-05 | 2.841667 |
| RPS6KA6 | -1.40554 | -0.19188 | -4.75033 | 1.34E-05 | 9.45E-05 | 2.818166 |
| MYH3 | -1.0467 | -0.09553 | -4.75026 | 1.34E-05 | 9.45E-05 | 2.817921 |
| MIAT | -1.23475 | -0.12354 | -4.74949 | 1.34E-05 | 9.47E-05 | 2.815296 |
| APOC2 | 1.971474 | -0.41438 | 4.74839 | 1.35E-05 | 9.50E-05 | 2.811513 |
| LGR6 | 1.913877 | 0.336236 | 4.747367 | 1.35E-05 | 9.52E-05 | 2.808004 |
| HOXA11 | 2.172602 | 0.579284 | 4.746229 | 1.36E-05 | 9.55E-05 | 2.804101 |
| KRT6A | 2.002894 | -0.1167 | 4.73822 | 1.40E-05 | 9.76E-05 | 2.776648 |
| PTPN13 | -1.22303 | -0.33156 | -4.73807 | 1.40E-05 | 9.76E-05 | 2.77613 |
| HMOX1 | 1.333366 | -0.16669 | 4.736612 | 1.40E-05 | 9.80E-05 | 2.771138 |
| NKX2-2 | -1.75214 | 0.065501 | -4.7355 | 1.41E-05 | 9.83E-05 | 2.767321 |
| ZNF439 | -1.10562 | -0.35399 | -4.7352 | 1.41E-05 | 9.83E-05 | 2.766305 |
| PDSS1 | 1.081616 | -0.25331 | 4.731177 | 1.43E-05 | 9.96E-05 | 2.752521 |
| AQP1 | 1.910571 | -0.20577 | 4.73059 | 1.44E-05 | 9.97E-05 | 2.75051 |
| TMEM151A | -1.39193 | 0.476503 | -4.72644 | 1.46E-05 | 0.000101 | 2.736291 |
| CGNL1 | -1.23652 | -0.13693 | -4.72632 | 1.46E-05 | 0.000101 | 2.735908 |
| VSIG1 | -1.66294 | -0.31412 | -4.72504 | 1.46E-05 | 0.000101 | 2.731499 |
| FCGBP | -1.33575 | -0.21683 | -4.71657 | 1.51E-05 | 0.000104 | 2.702529 |
| TSPYL5 | -1.7877 | -0.64027 | -4.71402 | 1.52E-05 | 0.000105 | 2.693823 |
| HTR3D | 1.344251 | 0.08811 | 4.712688 | 1.53E-05 | 0.000105 | 2.689262 |
| TESC | -1.47148 | -0.22255 | -4.71198 | 1.53E-05 | 0.000105 | 2.68684 |
| LECT1 | 1.358358 | -0.00595 | 4.711972 | 1.53E-05 | 0.000105 | 2.686812 |
| ISX | 3.274506 | 0.203678 | 4.711721 | 1.54E-05 | 0.000106 | 2.685957 |
| FLJ40606 | 1.465772 | -0.01478 | 4.707522 | 1.56E-05 | 0.000107 | 2.671603 |
| ZNF829 | -1.05533 | -0.1378 | -4.70729 | 1.56E-05 | 0.000107 | 2.670808 |
| F12 | 1.712182 | 0.006466 | 4.70437 | 1.58E-05 | 0.000108 | 2.660836 |
| SPINK5 | -1.68458 | -0.1617 | -4.70394 | 1.58E-05 | 0.000108 | 2.659379 |
| GPD1 | 1.254593 | -0.10777 | 4.699528 | 1.60E-05 | 0.000109 | 2.644302 |
| SEMA6A | 1.276189 | 0.005958 | 4.699261 | 1.60E-05 | 0.000109 | 2.643389 |
| PID1 | -1.15552 | -0.11561 | -4.69876 | 1.61E-05 | 0.00011 | 2.64169 |
| FLVCR2 | 1.837942 | -0.06251 | 4.697922 | 1.61E-05 | 0.00011 | 2.638818 |
| FLJ38379 | -1.53111 | 0.466823 | -4.69711 | 1.62E-05 | 0.00011 | 2.636048 |
| CDO1 | -1.85526 | -0.2548 | -4.69523 | 1.63E-05 | 0.000111 | 2.629633 |
| TEKT2 | -1.46298 | 0.115554 | -4.69364 | 1.64E-05 | 0.000111 | 2.624189 |
| SSTR1 | -1.12773 | -0.09767 | -4.69032 | 1.66E-05 | 0.000112 | 2.612868 |
| KLHL17 | -1.11402 | -0.17157 | -4.6895 | 1.66E-05 | 0.000112 | 2.61009 |
| CPM | 1.353238 | -0.16945 | 4.68941 | 1.66E-05 | 0.000112 | 2.609772 |
| ITLN1 | 3.291708 | -1.27953 | 4.687135 | 1.68E-05 | 0.000113 | 2.602012 |
| CLDN7 | 2.358128 | -0.77996 | 4.683005 | 1.70E-05 | 0.000115 | 2.58793 |
| MAGEL2 | 1.175634 | -0.28633 | 4.681057 | 1.71E-05 | 0.000115 | 2.581289 |
| GPR115 | 1.03348 | -0.06875 | 4.680221 | 1.72E-05 | 0.000115 | 2.578441 |
| GUCA2B | -1.6139 | 0.053709 | -4.673 | 1.76E-05 | 0.000118 | 2.553829 |
| BASP1 | -1.63354 | -0.1462 | -4.6728 | 1.76E-05 | 0.000118 | 2.553152 |
| SPG20 | -1.30485 | -0.34544 | -4.67162 | 1.77E-05 | 0.000119 | 2.549138 |
| LYPD6B | -1.74061 | -0.27732 | -4.67052 | 1.78E-05 | 0.000119 | 2.545395 |
| C5AR1 | 1.424428 | -0.46774 | 4.668877 | 1.79E-05 | 0.000119 | 2.539802 |
| CCL25 | 3.409523 | 1.237096 | 4.66843 | 1.79E-05 | 0.000119 | 2.538282 |
| GAD1 | 1.085043 | 0.065669 | 4.667188 | 1.80E-05 | 0.00012 | 2.534053 |
| GJB5 | 3.186476 | 0.370278 | 4.665065 | 1.81E-05 | 0.000121 | 2.526828 |
| FGL1 | 1.317848 | -0.33356 | 4.66189 | 1.83E-05 | 0.000122 | 2.516025 |
| CPS1 | 4.352567 | -1.68217 | 4.660399 | 1.84E-05 | 0.000122 | 2.510954 |
| MST1P9 | -2.12752 | -0.2193 | -4.65792 | 1.86E-05 | 0.000123 | 2.502507 |
| OLIG3 | 1.207772 | 0.008079 | 4.653436 | 1.89E-05 | 0.000125 | 2.487277 |
| NAT2 | 2.109305 | 0.528548 | 4.650454 | 1.91E-05 | 0.000126 | 2.477141 |
| PTPRR | -1.31883 | -0.08984 | -4.65012 | 1.91E-05 | 0.000126 | 2.476006 |
| GML | 1.028813 | -0.07256 | 4.647732 | 1.93E-05 | 0.000127 | 2.467893 |
| PPM1J | 1.093243 | -0.23108 | 4.646946 | 1.93E-05 | 0.000127 | 2.465224 |
| LANCL3 | -1.01668 | 0.026359 | -4.64487 | 1.95E-05 | 0.000128 | 2.458157 |
| C6orf15 | 1.00637 | 0.009107 | 4.640851 | 1.97E-05 | 0.00013 | 2.444525 |
| GNA14 | 1.251043 | -0.33266 | 4.636188 | 2.01E-05 | 0.000131 | 2.428697 |
| MFSD4 | -1.52565 | 0.132208 | -4.63019 | 2.05E-05 | 0.000134 | 2.408343 |
| PDPN | 1.254199 | -0.51804 | 4.628618 | 2.06E-05 | 0.000134 | 2.403017 |
| DPT | -1.80162 | 0.156591 | -4.62561 | 2.08E-05 | 0.000136 | 2.39281 |
| IZUMO1 | -1.37338 | 0.457043 | -4.62554 | 2.08E-05 | 0.000136 | 2.392585 |
| CCDC27 | 1.160182 | -0.06348 | 4.621557 | 2.11E-05 | 0.000137 | 2.379084 |
| SETBP1 | -1.04311 | -0.16542 | -4.61808 | 2.14E-05 | 0.000138 | 2.36729 |
| NFASC | -1.4739 | -0.14965 | -4.61801 | 2.14E-05 | 0.000138 | 2.367056 |
| RASSF4 | 1.166337 | -0.46591 | 4.614421 | 2.17E-05 | 0.00014 | 2.35491 |
| LOC100130193 | -1.14921 | 0.318723 | -4.61217 | 2.19E-05 | 0.000141 | 2.347277 |
| ABCG5 | 1.744003 | -0.23156 | 4.612012 | 2.19E-05 | 0.000141 | 2.346756 |
| PHEX | 1.083667 | -0.22481 | 4.608127 | 2.22E-05 | 0.000142 | 2.333606 |
| LOC100505702 | 1.133211 | -0.37173 | 4.606926 | 2.23E-05 | 0.000143 | 2.32954 |
| GUCA2A | 2.36646 | -0.06228 | 4.606906 | 2.23E-05 | 0.000143 | 2.329472 |
| NTSR1 | 1.13507 | -0.05421 | 4.604941 | 2.24E-05 | 0.000144 | 2.322827 |
| TPST1 | -1.07064 | -0.19676 | -4.59813 | 2.30E-05 | 0.000146 | 2.29978 |
| CLDN10 | -2.20086 | -0.2771 | -4.5981 | 2.30E-05 | 0.000146 | 2.299694 |
| EGR4 | 1.773832 | 0.045514 | 4.586712 | 2.39E-05 | 0.000152 | 2.261215 |
| HERC2P4 | -1.38295 | -0.04237 | -4.57841 | 2.46E-05 | 0.000155 | 2.233181 |
| TCEAL2 | -1.44873 | -0.33988 | -4.57469 | 2.49E-05 | 0.000157 | 2.220655 |
| CNTNAP2 | 1.113577 | 0.049097 | 4.565731 | 2.57E-05 | 0.000161 | 2.190444 |
| HOXA9 | 3.153947 | -0.47019 | 4.555819 | 2.67E-05 | 0.000167 | 2.157061 |
| SLC6A4 | -1.17184 | 0.222287 | -4.55206 | 2.70E-05 | 0.000168 | 2.144396 |
| DSG3 | 3.041623 | 0.510217 | 4.550131 | 2.72E-05 | 0.000169 | 2.137922 |
| DPEP1 | 2.234011 | 0.287874 | 4.549489 | 2.73E-05 | 0.000169 | 2.135763 |
| MYADML2 | 1.914489 | 0.335602 | 4.549269 | 2.73E-05 | 0.000169 | 2.135023 |
| PPP1R1C | -1.26295 | -0.08615 | -4.54616 | 2.76E-05 | 0.000171 | 2.124559 |
| TGM2 | 1.218737 | -0.30189 | 4.543563 | 2.78E-05 | 0.000172 | 2.115835 |
| QPRT | 1.516714 | 0.017186 | 4.541597 | 2.80E-05 | 0.000173 | 2.109226 |
| ZNF645 | 1.067919 | -0.02982 | 4.540278 | 2.82E-05 | 0.000174 | 2.104793 |
| LIPC | 1.795038 | 0.289046 | 4.539995 | 2.82E-05 | 0.000174 | 2.103841 |
| RXFP4 | 1.303767 | -0.60312 | 4.537517 | 2.84E-05 | 0.000175 | 2.095517 |
| C16orf89 | -2.24283 | -0.37234 | -4.52977 | 2.92E-05 | 0.000179 | 2.069498 |
| ZNF367 | 1.224972 | 0.164668 | 4.526141 | 2.96E-05 | 0.000181 | 2.057321 |
| SAMD5 | 1.108227 | -0.00459 | 4.523866 | 2.98E-05 | 0.000182 | 2.049685 |
| MEGF6 | -1.00243 | -0.10578 | -4.52262 | 3.00E-05 | 0.000183 | 2.045499 |
| CD55 | 1.632686 | -0.30858 | 4.519803 | 3.03E-05 | 0.000185 | 2.036058 |
| TAS2R50 | 1.02785 | 0.038746 | 4.518076 | 3.04E-05 | 0.000185 | 2.030269 |
| DNAH2 | 1.114177 | 0.098231 | 4.509375 | 3.14E-05 | 0.00019 | 2.001107 |
| ANXA13 | 3.2202 | -0.50829 | 4.508082 | 3.15E-05 | 0.000191 | 1.996776 |
| OR7A17 | 1.008189 | -0.0547 | 4.507718 | 3.16E-05 | 0.000191 | 1.995558 |
| PON3 | 1.253376 | 0.01183 | 4.506826 | 3.17E-05 | 0.000192 | 1.992572 |
| LOC100131929 | -1.15923 | -0.05509 | -4.50252 | 3.21E-05 | 0.000194 | 1.978152 |
| CPVL | 1.236139 | -0.22187 | 4.501209 | 3.23E-05 | 0.000195 | 1.973768 |
| CALHM3 | 2.110625 | -0.10424 | 4.50111 | 3.23E-05 | 0.000195 | 1.973436 |
| SLC16A10 | 1.167662 | -0.09123 | 4.500465 | 3.24E-05 | 0.000195 | 1.971275 |
| CILP | -1.34659 | -0.05598 | -4.49654 | 3.28E-05 | 0.000197 | 1.95816 |
| ABCA6 | -1.13514 | 0.037691 | -4.49444 | 3.31E-05 | 0.000199 | 1.95111 |
| FAM25A | 1.898412 | 0.017675 | 4.493347 | 3.32E-05 | 0.000199 | 1.947464 |
| CAND2 | -1.2399 | -0.15476 | -4.49306 | 3.32E-05 | 0.000199 | 1.946506 |
| DKFZP586B0319 | -1.35772 | -0.09216 | -4.48726 | 3.39E-05 | 0.000203 | 1.927127 |
| MST1 | -1.20432 | 0.125992 | -4.48725 | 3.39E-05 | 0.000203 | 1.927091 |
| RGS9 | -1.02189 | -0.05781 | -4.48528 | 3.41E-05 | 0.000204 | 1.920485 |
| SVOPL | 1.104173 | -0.25003 | 4.480944 | 3.47E-05 | 0.000206 | 1.906017 |
| C2orf88 | 1.886666 | 0.065542 | 4.478942 | 3.49E-05 | 0.000207 | 1.89933 |
| MUC20 | 1.278711 | 0.020901 | 4.477943 | 3.50E-05 | 0.000208 | 1.895997 |
| KCTD15 | -1.12651 | -0.15177 | -4.47768 | 3.51E-05 | 0.000208 | 1.895117 |
| WDR69 | 1.153136 | 0.313747 | 4.475875 | 3.53E-05 | 0.000209 | 1.889094 |
| KIAA1199 | 1.416301 | 0.319481 | 4.473833 | 3.55E-05 | 0.00021 | 1.88228 |
| OR52A1 | 1.067544 | -0.10771 | 4.466058 | 3.65E-05 | 0.000215 | 1.856343 |
| DMRTC1 | -1.29335 | -0.27682 | -4.46397 | 3.68E-05 | 0.000217 | 1.84937 |
| CUBN | 1.20739 | -0.00039 | 4.458907 | 3.74E-05 | 0.00022 | 1.832512 |
| C3orf24 | 1.016556 | -0.02523 | 4.454911 | 3.80E-05 | 0.000223 | 1.8192 |
| HPN | -2.17733 | -0.76678 | -4.45443 | 3.80E-05 | 0.000223 | 1.81761 |
| AGPAT4-IT1 | -1.42537 | -0.13501 | -4.45242 | 3.83E-05 | 0.000224 | 1.810892 |
| SAA1 | 2.590124 | -0.8553 | 4.451863 | 3.84E-05 | 0.000224 | 1.809052 |
| C6orf165 | 1.045475 | 0.075062 | 4.451852 | 3.84E-05 | 0.000224 | 1.809016 |
| ETHE1 | 1.115734 | 0.294732 | 4.449233 | 3.87E-05 | 0.000226 | 1.800299 |
| TKTL1 | 1.091611 | -0.07339 | 4.448112 | 3.89E-05 | 0.000227 | 1.79657 |
| EPHX4 | 2.086536 | 0.371238 | 4.447233 | 3.90E-05 | 0.000227 | 1.793643 |
| LGALS7B | 1.248231 | 0.264946 | 4.445722 | 3.92E-05 | 0.000228 | 1.788619 |
| MTMR10 | -1.17387 | -0.04945 | -4.4415 | 3.98E-05 | 0.000231 | 1.774577 |
| SLC23A3 | -1.24172 | -0.12823 | -4.43297 | 4.10E-05 | 0.000236 | 1.746238 |
| LOC100652973 | -1.09453 | -0.08238 | -4.42852 | 4.16E-05 | 0.00024 | 1.731446 |
| TPO | -1.02397 | -0.11131 | -4.42337 | 4.24E-05 | 0.000243 | 1.714352 |
| MAGEA12 | 2.017998 | 0.085049 | 4.42315 | 4.24E-05 | 0.000243 | 1.713619 |
| GOLT1A | 1.104949 | -0.17495 | 4.42301 | 4.24E-05 | 0.000243 | 1.713156 |
| MEP1B | 2.930748 | -0.05503 | 4.422709 | 4.25E-05 | 0.000243 | 1.712156 |
| CYP2C18 | -1.06064 | -0.07198 | -4.42118 | 4.27E-05 | 0.000244 | 1.707068 |
| KRT7 | 2.204933 | -0.19215 | 4.420986 | 4.27E-05 | 0.000244 | 1.706439 |
| SH3D21 | 1.126894 | -0.06938 | 4.408784 | 4.46E-05 | 0.000253 | 1.665985 |
| ALX1 | 1.021809 | 0.150138 | 4.405434 | 4.51E-05 | 0.000255 | 1.654889 |
| C8orf4 | 1.287276 | -0.26115 | 4.405369 | 4.51E-05 | 0.000255 | 1.654675 |
| CHST15 | -1.04824 | -0.22382 | -4.40448 | 4.52E-05 | 0.000256 | 1.651743 |
| LOC100506236 | 1.219224 | -0.29395 | 4.403443 | 4.54E-05 | 0.000257 | 1.648296 |
| TWIST2 | -1.03681 | 0.117175 | -4.40267 | 4.55E-05 | 0.000257 | 1.645726 |
| CDHR3 | -1.04336 | 0.216864 | -4.39948 | 4.60E-05 | 0.00026 | 1.635172 |
| CIDEC | -1.18909 | -0.03498 | -4.39624 | 4.65E-05 | 0.000262 | 1.624471 |
| DACT2 | -2.12699 | -0.09184 | -4.39181 | 4.73E-05 | 0.000266 | 1.609811 |
| CSF2 | -1.50633 | 0.154589 | -4.3914 | 4.73E-05 | 0.000266 | 1.608464 |
| MEP1A | 3.45679 | -0.19891 | 4.390496 | 4.75E-05 | 0.000267 | 1.605459 |
| DDX10 | 1.342158 | -0.21544 | 4.389513 | 4.76E-05 | 0.000267 | 1.602209 |
| AFAP1L2 | 1.036731 | -0.1553 | 4.387464 | 4.80E-05 | 0.000269 | 1.595434 |
| EGR1 | 1.445973 | -0.35331 | 4.386579 | 4.81E-05 | 0.000269 | 1.592513 |
| GCG | 4.058575 | 0.281293 | 4.386508 | 4.81E-05 | 0.000269 | 1.592276 |
| MYPN | -1.1416 | -0.0572 | -4.38366 | 4.86E-05 | 0.000272 | 1.582859 |
| LYPD6 | -1.23926 | -0.0127 | -4.38146 | 4.90E-05 | 0.000273 | 1.575593 |
| ACTL8 | 1.991236 | 0.439085 | 4.378316 | 4.95E-05 | 0.000276 | 1.565216 |
| SERPINA4 | -1.73303 | -0.59351 | -4.37692 | 4.98E-05 | 0.000277 | 1.560598 |
| VNN1 | 2.65757 | -1.01075 | 4.376194 | 4.99E-05 | 0.000278 | 1.558212 |
| LOC100129406 | -1.16312 | -0.00248 | -4.37543 | 5.00E-05 | 0.000278 | 1.555679 |
| C2orf40 | -2.26546 | 0.22635 | -4.37264 | 5.05E-05 | 0.00028 | 1.546482 |
| FLJ32756 | 1.311502 | 0.087804 | 4.367064 | 5.15E-05 | 0.000285 | 1.528091 |
| CELF3 | -1.03666 | 0.109653 | -4.36675 | 5.15E-05 | 0.000285 | 1.527059 |
| ARMCX2 | -1.00984 | -0.27929 | -4.36637 | 5.16E-05 | 0.000286 | 1.525814 |
| ATP8B5P | 1.05133 | 0.08328 | 4.357292 | 5.33E-05 | 0.000293 | 1.495885 |
| PADI2 | 1.286955 | 0.199063 | 4.352018 | 5.42E-05 | 0.000298 | 1.478521 |
| SAP30 | -1.03343 | 0.213151 | -4.35154 | 5.43E-05 | 0.000298 | 1.476962 |
| GPRIN3 | 1.402394 | -0.36126 | 4.351227 | 5.44E-05 | 0.000298 | 1.475918 |
| CSAG1 | 1.402991 | -0.09309 | 4.350577 | 5.45E-05 | 0.000299 | 1.473778 |
| B3GNT8 | 1.090573 | -0.09754 | 4.349111 | 5.48E-05 | 0.0003 | 1.468954 |
| JAKMIP3 | -1.38236 | 0.394764 | -4.34835 | 5.49E-05 | 0.000301 | 1.466445 |
| PTGS2 | 1.749583 | -0.34126 | 4.345561 | 5.55E-05 | 0.000303 | 1.457277 |
| TRIM23 | 1.171391 | 0.105634 | 4.342567 | 5.60E-05 | 0.000306 | 1.447428 |
| ASPHD2 | -1.05695 | 0.037195 | -4.3414 | 5.63E-05 | 0.000307 | 1.443589 |
| FLJ45248 | 1.327569 | -0.39185 | 4.337219 | 5.71E-05 | 0.00031 | 1.429851 |
| LOC157860 | 2.049097 | 0.191723 | 4.337175 | 5.71E-05 | 0.00031 | 1.429707 |
| VTCN1 | -1.31569 | 0.166146 | -4.33611 | 5.73E-05 | 0.000311 | 1.42622 |
| NBPF22P | 1.230024 | -0.05659 | 4.33054 | 5.84E-05 | 0.000316 | 1.407913 |
| RIMS3 | -1.09705 | 0.137038 | -4.33026 | 5.85E-05 | 0.000317 | 1.406989 |
| ODF3L1 | -1.37951 | -0.06969 | -4.32762 | 5.90E-05 | 0.000319 | 1.39834 |
| BCL2L10 | 1.089941 | -0.02061 | 4.32554 | 5.94E-05 | 0.00032 | 1.391501 |
| DACH1 | 1.587932 | 0.302412 | 4.325519 | 5.94E-05 | 0.00032 | 1.391433 |
| SLC25A21 | 1.537472 | -0.22301 | 4.32477 | 5.96E-05 | 0.000321 | 1.388972 |
| NECAB2 | -1.46292 | -0.02101 | -4.32413 | 5.97E-05 | 0.000322 | 1.38687 |
| SLC14A2 | 1.054257 | 0.223793 | 4.322824 | 6.00E-05 | 0.000323 | 1.382589 |
| TTC9 | -1.02428 | -0.12205 | -4.3225 | 6.00E-05 | 0.000323 | 1.381536 |
| FAM3B | -1.17154 | -0.28882 | -4.32099 | 6.04E-05 | 0.000325 | 1.376564 |
| LIN28A | 1.102735 | -0.00106 | 4.316144 | 6.14E-05 | 0.000329 | 1.360684 |
| OASL | -1.16993 | -0.15746 | -4.31466 | 6.17E-05 | 0.000331 | 1.355816 |
| FOXJ1 | 1.268073 | 0.107346 | 4.313586 | 6.19E-05 | 0.000332 | 1.352302 |
| ZNF606 | -1.46071 | -0.60735 | -4.3133 | 6.20E-05 | 0.000332 | 1.351351 |
| INHBA | 1.17023 | -0.0818 | 4.309931 | 6.27E-05 | 0.000335 | 1.340327 |
| EIF1AY | 2.059968 | -1.23188 | 4.307615 | 6.32E-05 | 0.000337 | 1.332744 |
| TINAG | 1.428751 | -0.08616 | 4.305173 | 6.37E-05 | 0.00034 | 1.324749 |
| CCL20 | 1.989044 | -0.43123 | 4.301836 | 6.45E-05 | 0.000343 | 1.313826 |
| RIPPLY2 | -1.31618 | 0.062508 | -4.29589 | 6.58E-05 | 0.000349 | 1.294392 |
| COLEC12 | -1.71458 | -0.25332 | -4.28853 | 6.75E-05 | 0.000357 | 1.270313 |
| SEC31B | -1.42754 | -0.1905 | -4.28818 | 6.76E-05 | 0.000357 | 1.269173 |
| SLC5A11 | 1.111666 | -0.37697 | 4.286764 | 6.79E-05 | 0.000359 | 1.264551 |
| C7orf13 | -1.24167 | -0.03977 | -4.28645 | 6.80E-05 | 0.000359 | 1.26351 |
| WISP2 | -1.46175 | 0.100624 | -4.28629 | 6.80E-05 | 0.000359 | 1.263001 |
| LOC400043 | -1.85466 | -0.62804 | -4.28605 | 6.81E-05 | 0.000359 | 1.262232 |
| GAMT | -1.40459 | -0.59084 | -4.28485 | 6.83E-05 | 0.00036 | 1.258298 |
| DDX60 | -1.41554 | -0.02094 | -4.28462 | 6.84E-05 | 0.00036 | 1.257546 |
| PCK1 | 2.011056 | -0.34084 | 4.282224 | 6.89E-05 | 0.000363 | 1.249725 |
| APOA2 | 1.175518 | -0.03113 | 4.281274 | 6.92E-05 | 0.000364 | 1.246625 |
| OR2W1 | 1.067736 | -0.14428 | 4.279357 | 6.96E-05 | 0.000366 | 1.240371 |
| TNFRSF8 | -1.37033 | -0.08716 | -4.27858 | 6.98E-05 | 0.000367 | 1.23782 |
| B3GAT1 | -2.06285 | -0.47705 | -4.2782 | 6.99E-05 | 0.000367 | 1.236604 |
| ENTPD3 | -1.21138 | -0.31953 | -4.27507 | 7.07E-05 | 0.00037 | 1.22638 |
| MAGED4B | -1.14764 | -0.23338 | -4.2727 | 7.12E-05 | 0.000372 | 1.218642 |
| CLDN15 | 2.203906 | 0.590118 | 4.272033 | 7.14E-05 | 0.000373 | 1.21648 |
| BEND5 | -1.37535 | -0.34802 | -4.27005 | 7.19E-05 | 0.000375 | 1.210013 |
| ZNF876P | -1.17668 | -0.21066 | -4.26831 | 7.23E-05 | 0.000377 | 1.204329 |
| UNC93A | 1.551839 | -0.26186 | 4.264669 | 7.32E-05 | 0.000381 | 1.19248 |
| PHOSPHO2-KLHL23 | 1.390549 | -0.54799 | 4.261158 | 7.41E-05 | 0.000384 | 1.181046 |
| CPT1B | -1.12732 | 0.101448 | -4.25783 | 7.50E-05 | 0.000388 | 1.170205 |
| KCNK9 | 1.190951 | -0.19432 | 4.253378 | 7.61E-05 | 0.000394 | 1.155724 |
| OVOL1 | 1.185393 | -0.11703 | 4.252974 | 7.62E-05 | 0.000394 | 1.15441 |
| CACNG3 | 1.060727 | -0.15303 | 4.252197 | 7.64E-05 | 0.000395 | 1.151883 |
| GPR15 | 1.09436 | -0.12706 | 4.246051 | 7.80E-05 | 0.000403 | 1.131901 |
| ANKRD36BP2 | -1.02872 | -0.23449 | -4.24463 | 7.84E-05 | 0.000404 | 1.127286 |
| UNC5B | -1.11958 | 0.001102 | -4.24279 | 7.89E-05 | 0.000406 | 1.121312 |
| KL | -1.1033 | 0.085651 | -4.23654 | 8.06E-05 | 0.000413 | 1.101017 |
| CA1 | 2.901821 | 0.542067 | 4.236109 | 8.07E-05 | 0.000414 | 1.099606 |
| ZDBF2 | -1.13798 | -0.16958 | -4.23383 | 8.14E-05 | 0.000416 | 1.092213 |
| SLC1A1 | 1.000153 | -0.01679 | 4.231481 | 8.20E-05 | 0.000419 | 1.084588 |
| CCL15 | 1.029582 | 0.183696 | 4.230195 | 8.24E-05 | 0.00042 | 1.080415 |
| HENMT1 | 1.047925 | -0.07498 | 4.228062 | 8.30E-05 | 0.000423 | 1.0735 |
| SERPINB7 | 1.284223 | 0.043734 | 4.227018 | 8.33E-05 | 0.000424 | 1.070114 |
| ZNF818P | -1.31262 | -0.25289 | -4.22514 | 8.38E-05 | 0.000426 | 1.064016 |
| LOC389023 | -2.06329 | -0.08947 | -4.22494 | 8.39E-05 | 0.000427 | 1.06338 |
| CYP27B1 | 1.002326 | 0.077368 | 4.224186 | 8.41E-05 | 0.000428 | 1.060932 |
| GSDMA | 1.213176 | -0.14871 | 4.222399 | 8.46E-05 | 0.000429 | 1.055142 |
| PSG5 | 1.01237 | -0.02272 | 4.222077 | 8.47E-05 | 0.000429 | 1.054099 |
| RASL11A | 1.266152 | 0.005685 | 4.217891 | 8.59E-05 | 0.000435 | 1.040536 |
| ATP6V0A4 | 1.226747 | -0.13387 | 4.216844 | 8.62E-05 | 0.000436 | 1.037147 |
| IL2RA | 1.236368 | -0.23059 | 4.215436 | 8.66E-05 | 0.000438 | 1.032587 |
| KLHL1 | -1.46461 | 0.523195 | -4.21432 | 8.69E-05 | 0.00044 | 1.028974 |
| LOC442459 | 1.119878 | -0.20459 | 4.209805 | 8.83E-05 | 0.000445 | 1.014361 |
| RFPL3-AS1 | -1.36268 | 0.118137 | -4.20426 | 9.00E-05 | 0.000452 | 0.996423 |
| PRR4 | -2.26074 | -0.21542 | -4.20232 | 9.06E-05 | 0.000455 | 0.990144 |
| PPP1R14D | 1.405206 | -0.25431 | 4.200706 | 9.11E-05 | 0.000457 | 0.984939 |
| SI | 4.147438 | -0.3079 | 4.20063 | 9.11E-05 | 0.000457 | 0.984694 |
| SERPINA1 | 1.301155 | -0.21866 | 4.198049 | 9.19E-05 | 0.000461 | 0.976353 |
| CCL26 | 2.060716 | 0.03621 | 4.196578 | 9.23E-05 | 0.000463 | 0.971603 |
| CELA1 | 1.160042 | 0.013324 | 4.194179 | 9.31E-05 | 0.000466 | 0.963855 |
| LOC100132790 | -1.27087 | 0.089905 | -4.19023 | 9.44E-05 | 0.000471 | 0.95111 |
| GRM3 | -1.35065 | 0.577768 | -4.18722 | 9.53E-05 | 0.000475 | 0.941397 |
| DEPDC7 | 1.690959 | -0.19908 | 4.18693 | 9.54E-05 | 0.000476 | 0.940457 |
| EMR2 | 1.452404 | -0.5692 | 4.181758 | 9.71E-05 | 0.000483 | 0.923777 |
| KCNE2 | -2.01251 | -0.37583 | -4.17928 | 9.79E-05 | 0.000486 | 0.915804 |
| DEFA6 | 4.084433 | 1.90887 | 4.178431 | 9.82E-05 | 0.000487 | 0.913056 |
| SOX14 | 1.354738 | 0.314195 | 4.177456 | 9.85E-05 | 0.000489 | 0.909912 |
| EYA2 | -1.75509 | -0.64012 | -4.17678 | 9.88E-05 | 0.00049 | 0.907748 |
| SLC26A7 | -1.49495 | -0.28285 | -4.17567 | 9.91E-05 | 0.000491 | 0.904146 |
| CRX | 1.188057 | -0.02514 | 4.17499 | 9.94E-05 | 0.000492 | 0.901968 |
| ALDOB | 2.427523 | 0.151535 | 4.174494 | 9.95E-05 | 0.000493 | 0.900371 |
| S100A3 | 1.365388 | -0.22253 | 4.171662 | 0.0001 | 0.000497 | 0.89125 |
| WNT5A | 1.122703 | -0.1623 | 4.167105 | 0.000102 | 0.000503 | 0.876582 |
| HLA-DOB | 1.180156 | -0.22501 | 4.166874 | 0.000102 | 0.000504 | 0.875838 |
| PCSK1 | -2.13258 | 0.394681 | -4.16661 | 0.000102 | 0.000504 | 0.875003 |
| DMRTA1 | -1.492 | -0.47752 | -4.16651 | 0.000102 | 0.000504 | 0.874661 |
| SULT4A1 | 1.188008 | 0.095045 | 4.160961 | 0.000104 | 0.000512 | 0.856818 |
| PROCR | 1.019783 | 0.061206 | 4.158593 | 0.000105 | 0.000515 | 0.849208 |
| SCIN | -1.21713 | -0.03352 | -4.1571 | 0.000106 | 0.000517 | 0.844398 |
| GABRP | 1.820167 | 0.222725 | 4.152802 | 0.000107 | 0.000524 | 0.830599 |
| KBTBD12 | -1.65852 | -0.43474 | -4.15208 | 0.000107 | 0.000525 | 0.828295 |
| ELOVL4 | 1.132797 | 0.023366 | 4.150792 | 0.000108 | 0.000527 | 0.824143 |
| SOX2 | -1.53619 | -0.46679 | -4.14946 | 0.000108 | 0.000529 | 0.819873 |
| DIRAS3 | -1.56589 | 0.033467 | -4.14622 | 0.00011 | 0.000533 | 0.809477 |
| AXIN2 | -1.09568 | -0.01812 | -4.14484 | 0.00011 | 0.000535 | 0.80504 |
| LOC100506253 | 1.572277 | -0.23545 | 4.142874 | 0.000111 | 0.000538 | 0.798731 |
| PLCXD1 | -1.16509 | 0.252762 | -4.13823 | 0.000113 | 0.000546 | 0.783834 |
| CCKBR | -2.50577 | -1.14359 | -4.13697 | 0.000113 | 0.000548 | 0.779797 |
| TDRD9 | -2.52481 | 0.620791 | -4.13501 | 0.000114 | 0.00055 | 0.773526 |
| MYB | 1.126827 | 0.070513 | 4.134064 | 0.000114 | 0.000551 | 0.770488 |
| LOC100507165 | 1.000492 | -0.10535 | 4.126952 | 0.000117 | 0.000563 | 0.747711 |
| AGMAT | 1.08525 | -0.23822 | 4.121902 | 0.000119 | 0.000571 | 0.731551 |
| DUOX1 | -1.49541 | 0.09473 | -4.1191 | 0.00012 | 0.000575 | 0.722585 |
| HTRA4 | 1.53929 | -0.22743 | 4.117564 | 0.000121 | 0.000578 | 0.717678 |
| ACCS | -1.06511 | -0.0761 | -4.11575 | 0.000121 | 0.000581 | 0.711885 |
| DEFB1 | -1.70191 | 0.248806 | -4.11129 | 0.000123 | 0.000588 | 0.697613 |
| GREM2 | -2.49574 | -0.41657 | -4.11067 | 0.000123 | 0.000589 | 0.695652 |
| LOC145837 | -1.13122 | -0.12331 | -4.10817 | 0.000124 | 0.000593 | 0.687667 |
| FABP2 | 2.632461 | 0.40804 | 4.106055 | 0.000125 | 0.000596 | 0.680912 |
| FAM150A | 1.519103 | -0.15298 | 4.100893 | 0.000128 | 0.000605 | 0.664439 |
| HERC2P7 | -1.49159 | -0.48803 | -4.09904 | 0.000128 | 0.000608 | 0.658537 |
| BEX2 | -1.79021 | -0.81263 | -4.0963 | 0.00013 | 0.000613 | 0.649793 |
| MB | 1.177626 | 0.300368 | 4.091561 | 0.000132 | 0.000621 | 0.63469 |
| SLC35G1 | 1.207027 | 0.230018 | 4.091513 | 0.000132 | 0.000621 | 0.634536 |
| SLC46A3 | 1.649815 | -0.40818 | 4.089997 | 0.000132 | 0.000624 | 0.629708 |
| GPR32 | 1.139003 | -0.27638 | 4.08709 | 0.000134 | 0.000629 | 0.620449 |
| FGFBP2 | -1.21952 | -0.05939 | -4.08702 | 0.000134 | 0.000629 | 0.62024 |
| KCNMB2 | -1.46857 | 0.031349 | -4.08602 | 0.000134 | 0.000631 | 0.617052 |
| PKIB | -1.51218 | -0.22566 | -4.0848 | 0.000135 | 0.000633 | 0.613174 |
| ZNF772 | -1.25139 | -0.17121 | -4.0737 | 0.00014 | 0.000654 | 0.577861 |
| ORM2 | -2.02659 | -0.57856 | -4.07322 | 0.00014 | 0.000655 | 0.576313 |
| PEX3 | 1.078824 | 0.097883 | 4.071488 | 0.000141 | 0.000658 | 0.570823 |
| FOXD1 | 1.565127 | -0.16665 | 4.071341 | 0.000141 | 0.000658 | 0.570358 |
| RDH12 | -1.69375 | -0.03723 | -4.06871 | 0.000142 | 0.000663 | 0.561999 |
| PRSS1 | 2.218579 | 0.276007 | 4.066278 | 0.000143 | 0.000668 | 0.554277 |
| CSNK1A1L | 1.082148 | 0.398317 | 4.065017 | 0.000144 | 0.00067 | 0.550274 |
| LEPREL1 | -1.78619 | -0.2864 | -4.06471 | 0.000144 | 0.000671 | 0.549293 |
| BEX1 | -2.07187 | -0.05546 | -4.06178 | 0.000145 | 0.000676 | 0.540011 |
| NKX3-2 | -1.71522 | -0.13096 | -4.05969 | 0.000146 | 0.00068 | 0.533352 |
| HOMER2 | -1.0047 | -0.27094 | -4.05953 | 0.000147 | 0.00068 | 0.532846 |
| WSCD2 | -1.99372 | -0.03093 | -4.05725 | 0.000148 | 0.000684 | 0.525624 |
| CLDN4 | 1.968309 | -0.8754 | 4.056644 | 0.000148 | 0.000685 | 0.523707 |
| TNFRSF12A | 1.384254 | -0.29413 | 4.054471 | 0.000149 | 0.000689 | 0.516818 |
| ATF3 | 1.373959 | 0.000391 | 4.053208 | 0.00015 | 0.000692 | 0.512815 |
| GNG4 | -1.05568 | 0.048005 | -4.05059 | 0.000151 | 0.000697 | 0.504505 |
| CELSR3 | 1.016342 | 0.167554 | 4.04948 | 0.000152 | 0.000699 | 0.501001 |
| ADAMTS4 | 1.242959 | -0.2051 | 4.048274 | 0.000152 | 0.000702 | 0.497183 |
| PLAC1 | 1.537461 | -0.30022 | 4.045343 | 0.000154 | 0.000708 | 0.487899 |
| PEBP4 | -2.11854 | 0.349399 | -4.03972 | 0.000157 | 0.000719 | 0.470097 |
| KRT24 | 1.078786 | 0.090662 | 4.036514 | 0.000158 | 0.000725 | 0.459965 |
| TREM1 | 1.648363 | -0.44392 | 4.035121 | 0.000159 | 0.000728 | 0.455562 |
| DYNC2H1 | -1.34539 | 0.18023 | -4.03306 | 0.00016 | 0.000731 | 0.449053 |
| C1orf64 | -1.12488 | 0.046988 | -4.03303 | 0.00016 | 0.000731 | 0.448939 |
| FLJ43663 | -1.03081 | -0.32508 | -4.0325 | 0.00016 | 0.000732 | 0.447291 |
| LGALS2 | 1.355956 | -0.16322 | 4.030406 | 0.000162 | 0.000736 | 0.440658 |
| ZNF610 | -1.41748 | -0.2085 | -4.0262 | 0.000164 | 0.000744 | 0.427365 |
| ADAM7 | 1.23278 | 0.212908 | 4.025365 | 0.000164 | 0.000746 | 0.424737 |
| CHRDL1 | -1.29911 | -0.21121 | -4.02278 | 0.000166 | 0.000751 | 0.416593 |
| THBS4 | -1.27376 | -0.03336 | -4.01829 | 0.000168 | 0.000761 | 0.402423 |
| AOX1 | -1.00908 | -0.05647 | -4.01531 | 0.00017 | 0.000767 | 0.393003 |
| BEX4 | -1.06435 | -0.39389 | -4.0146 | 0.00017 | 0.000769 | 0.39077 |
| TXLNB | -1.09002 | -0.23617 | -4.01194 | 0.000172 | 0.000775 | 0.382407 |
| ADH4 | 3.52805 | 0.188506 | 4.011811 | 0.000172 | 0.000775 | 0.381985 |
| C2orf74 | -1.37326 | 0.156092 | -4.01134 | 0.000172 | 0.000776 | 0.380514 |
| FAM40B | 1.160889 | -0.20416 | 4.008281 | 0.000174 | 0.000783 | 0.370864 |
| SIGLEC10 | 1.153507 | -0.45546 | 3.997694 | 0.00018 | 0.000808 | 0.337545 |
| CYP2W1 | 1.044292 | 0.02682 | 3.99643 | 0.000181 | 0.00081 | 0.333571 |
| AKR1C4 | 1.072041 | 0.027771 | 3.995147 | 0.000182 | 0.000813 | 0.329537 |
| LINC00319 | -1.01361 | -0.18422 | -3.99287 | 0.000183 | 0.000817 | 0.322384 |
| C18orf34 | 1.236001 | -0.59836 | 3.983698 | 0.000189 | 0.00084 | 0.293572 |
| PSG6 | 1.126886 | -0.04798 | 3.983156 | 0.000189 | 0.000841 | 0.29187 |
| LINC00238 | 1.438931 | 0.11794 | 3.976025 | 0.000193 | 0.000858 | 0.269501 |
| C10orf125 | 1.004494 | 0.079927 | 3.97425 | 0.000195 | 0.000862 | 0.263937 |
| MAMDC2 | -1.33292 | -0.02627 | -3.96335 | 0.000202 | 0.00089 | 0.229808 |
| LOC283663 | 1.175185 | -0.15856 | 3.962612 | 0.000202 | 0.000891 | 0.227491 |
| LOC100505697 | 1.029703 | -0.02901 | 3.958696 | 0.000205 | 0.000901 | 0.215239 |
| CCL3 | 1.350169 | -0.40302 | 3.955998 | 0.000207 | 0.000906 | 0.206803 |
| CTH | 1.034541 | -0.33662 | 3.955878 | 0.000207 | 0.000906 | 0.206428 |
| C8orf48 | -1.70501 | 0.701366 | -3.95202 | 0.000209 | 0.000916 | 0.194377 |
| OSTalpha | 2.755231 | -0.48094 | 3.949291 | 0.000211 | 0.000924 | 0.185848 |
| AJAP1 | -1.27342 | -0.11327 | -3.94876 | 0.000212 | 0.000924 | 0.184202 |
| LY6D | 2.396212 | 0.533309 | 3.947453 | 0.000213 | 0.000928 | 0.180107 |
| MMP12 | 2.098948 | -0.54771 | 3.943219 | 0.000216 | 0.000938 | 0.166892 |
| ARSE | 1.13712 | -0.12413 | 3.942837 | 0.000216 | 0.000939 | 0.1657 |
| LOC100505915 | -1.25469 | 0.077824 | -3.94141 | 0.000217 | 0.000942 | 0.161244 |
| SLC18A1 | 1.555327 | 0.348451 | 3.940463 | 0.000218 | 0.000944 | 0.158294 |
| LOC100271840 | 1.077718 | 0.024702 | 3.9396 | 0.000218 | 0.000947 | 0.155601 |
| KCNE4 | -1.69993 | -0.45224 | -3.93416 | 0.000222 | 0.000962 | 0.138656 |
| MLN | 3.332021 | 0.873843 | 3.933562 | 0.000223 | 0.000964 | 0.136779 |
| SECTM1 | -1.14152 | -0.09121 | -3.93022 | 0.000225 | 0.000973 | 0.126373 |
| PRODH2 | 1.013033 | 0.145587 | 3.929888 | 0.000225 | 0.000974 | 0.125336 |
| NOL4 | -1.74001 | -0.24408 | -3.92955 | 0.000226 | 0.000975 | 0.12428 |
| ADAMDEC1 | 1.348311 | -0.41195 | 3.917741 | 0.000235 | 0.001008 | 0.087541 |
| ANPEP | 3.448106 | -0.18633 | 3.916397 | 0.000236 | 0.00101 | 0.083362 |
| KATNAL2 | 1.070366 | 0.174005 | 3.915067 | 0.000237 | 0.001014 | 0.079228 |
| LEMD1 | 1.675142 | 0.440981 | 3.910517 | 0.00024 | 0.001028 | 0.065095 |
| PTGDS | 1.070428 | -0.35604 | 3.903397 | 0.000246 | 0.001049 | 0.042996 |
| RNF217 | -1.16441 | -0.17974 | -3.90318 | 0.000246 | 0.00105 | 0.042311 |
| MUC13 | 1.102376 | 0.024221 | 3.898444 | 0.00025 | 0.001065 | 0.027637 |
| KCNS3 | -1.4441 | 0.005564 | -3.8965 | 0.000251 | 0.001071 | 0.021623 |
| PSCA | -1.79598 | -0.07664 | -3.89542 | 0.000252 | 0.001074 | 0.018256 |
| IRAK3 | -1.25331 | -0.38958 | -3.88562 | 0.000261 | 0.001102 | -0.01208 |
| ORM1 | -1.98234 | -0.49953 | -3.88164 | 0.000264 | 0.001115 | -0.02439 |
| C20orf85 | 1.760426 | 0.456426 | 3.877142 | 0.000268 | 0.001128 | -0.03829 |
| LOC100506165 | 2.56138 | 0.135517 | 3.872817 | 0.000272 | 0.001143 | -0.05165 |
| PDE8B | -1.20153 | -0.10397 | -3.86943 | 0.000275 | 0.001154 | -0.06211 |
| VGLL1 | 1.093346 | -0.10678 | 3.868081 | 0.000276 | 0.001158 | -0.06627 |
| SFRP5 | -1.6518 | 0.11582 | -3.86757 | 0.000276 | 0.001159 | -0.06783 |
| XIST | -4.49283 | 3.306873 | -3.8639 | 0.00028 | 0.001171 | -0.07917 |
| HSDL2 | 1.151718 | 0.342128 | 3.862278 | 0.000281 | 0.001176 | -0.08416 |
| LOC155060 | -1.14377 | -0.39821 | -3.86079 | 0.000283 | 0.00118 | -0.08875 |
| DLEC1 | -1.21607 | 0.020965 | -3.85822 | 0.000285 | 0.001188 | -0.09668 |
| APOA4 | 2.470105 | 0.333611 | 3.858165 | 0.000285 | 0.001188 | -0.09684 |
| LDLRAD1 | -1.01188 | 0.117587 | -3.85458 | 0.000288 | 0.0012 | -0.10787 |
| LOC100287852 | 1.117617 | 0.162872 | 3.854215 | 0.000289 | 0.001201 | -0.109 |
| TRIM15 | 1.136846 | -0.28065 | 3.85291 | 0.00029 | 0.001205 | -0.11302 |
| MUC2 | 1.03997 | 0.177839 | 3.852764 | 0.00029 | 0.001206 | -0.11347 |
| RLBP1 | 1.0866 | -0.17823 | 3.852322 | 0.000291 | 0.001206 | -0.11483 |
| GAL | 2.197853 | -0.00318 | 3.849715 | 0.000293 | 0.001215 | -0.12286 |
| WNK4 | 1.458777 | -0.11012 | 3.847617 | 0.000295 | 0.001223 | -0.12931 |
| CA2 | -1.06999 | -0.40495 | -3.84174 | 0.000301 | 0.001242 | -0.14737 |
| LOC729080 | 1.829156 | 0.179512 | 3.840067 | 0.000302 | 0.001248 | -0.15252 |
| RPS4Y2 | 4.480403 | -2.88489 | 3.828367 | 0.000314 | 0.001288 | -0.18843 |
| LOC254057 | 1.864117 | 0.431419 | 3.827232 | 0.000315 | 0.001291 | -0.19191 |
| SIPA1L2 | 1.087454 | -0.12248 | 3.826697 | 0.000316 | 0.001293 | -0.19355 |
| RNF150 | -1.34662 | -0.27002 | -3.82428 | 0.000318 | 0.001301 | -0.20096 |
| CXorf61 | 3.55899 | -0.19814 | 3.822775 | 0.00032 | 0.001306 | -0.20557 |
| NAP1L2 | -1.11461 | 0.00923 | -3.81559 | 0.000327 | 0.001333 | -0.22759 |
| CXXC4 | -1.03764 | 0.085481 | -3.81437 | 0.000329 | 0.001337 | -0.23131 |
| NOX1 | 1.543279 | 0.400906 | 3.81318 | 0.00033 | 0.001341 | -0.23495 |
| CPNE8 | -1.0897 | 0.068575 | -3.80971 | 0.000334 | 0.001354 | -0.24555 |
| CD86 | 1.169621 | -0.17686 | 3.807695 | 0.000336 | 0.001363 | -0.25173 |
| OBP2A | 1.26704 | 0.061326 | 3.806853 | 0.000337 | 0.001366 | -0.2543 |
| REG1B | 2.230409 | 0.358609 | 3.805638 | 0.000338 | 0.00137 | -0.25801 |
| AOC3 | -1.13441 | -0.04737 | -3.80561 | 0.000338 | 0.00137 | -0.25809 |
| SERPINB5 | 1.201123 | 0.032687 | 3.801372 | 0.000343 | 0.001386 | -0.27105 |
| OGDHL | -1.63004 | -0.50731 | -3.77945 | 0.000368 | 0.001476 | -0.33788 |
| UST | -1.11656 | -0.05709 | -3.77865 | 0.000369 | 0.001478 | -0.3403 |
| IL1R2 | -1.32977 | 0.018924 | -3.76538 | 0.000385 | 0.001533 | -0.38063 |
| HOTAIR | 1.923292 | 0.633922 | 3.764354 | 0.000386 | 0.001536 | -0.38376 |
| LOC100506123 | -1.2447 | 0.025752 | -3.76408 | 0.000387 | 0.001537 | -0.38458 |
| KRT12 | -1.30591 | -0.21279 | -3.75867 | 0.000394 | 0.001559 | -0.40102 |
| NANOS1 | -1.03252 | 0.148514 | -3.75486 | 0.000398 | 0.001576 | -0.41257 |
| HNRNPA1P27 | 1.236177 | 0.027233 | 3.754443 | 0.000399 | 0.001578 | -0.41382 |
| CLRN3 | 1.690577 | -0.11174 | 3.751318 | 0.000403 | 0.00159 | -0.42329 |
| TNNC2 | 1.470622 | 0.341363 | 3.750545 | 0.000404 | 0.001593 | -0.42564 |
| FLJ32255 | 1.110363 | -0.26043 | 3.742229 | 0.000415 | 0.00163 | -0.45081 |
| KIAA0485 | -1.05274 | -0.11508 | -3.72866 | 0.000433 | 0.001693 | -0.49182 |
| MOGAT2 | 1.299989 | -0.08903 | 3.728604 | 0.000433 | 0.001693 | -0.49198 |
| C2orf54 | 1.704367 | -0.32922 | 3.718739 | 0.000447 | 0.00174 | -0.52173 |
| KIF24 | -1.17199 | 0.019288 | -3.71858 | 0.000448 | 0.001741 | -0.52221 |
| PLCXD3 | -1.68177 | -0.09028 | -3.71645 | 0.000451 | 0.00175 | -0.52863 |
| ARNT2 | -1.02471 | -0.20959 | -3.71559 | 0.000452 | 0.001753 | -0.53122 |
| PRB1 | -1.59358 | 0.735705 | -3.71453 | 0.000453 | 0.001759 | -0.53441 |
| DNAH10 | 1.102709 | 0.345624 | 3.713433 | 0.000455 | 0.001765 | -0.53771 |
| C17orf78 | 2.527755 | 0.842721 | 3.711732 | 0.000458 | 0.001774 | -0.54283 |
| PPP4R1L | -1.04839 | -0.02365 | -3.70743 | 0.000464 | 0.001794 | -0.55578 |
| LOC100240734 | -1.53761 | -0.30355 | -3.70381 | 0.000469 | 0.001811 | -0.56666 |
| SNX22 | 1.005845 | -0.02965 | 3.695943 | 0.000481 | 0.001849 | -0.59029 |
| NTM | -1.12615 | 0.421874 | -3.69567 | 0.000482 | 0.001849 | -0.59111 |
| IL8 | 1.374795 | 0.173215 | 3.685074 | 0.000498 | 0.001901 | -0.62289 |
| KLRK1 | -1.10801 | -0.11513 | -3.68452 | 0.000499 | 0.001904 | -0.62453 |
| EFEMP1 | -1.07264 | 0.059532 | -3.67551 | 0.000514 | 0.001952 | -0.65152 |
| SNHG7 | -1.02747 | -0.15117 | -3.67221 | 0.000519 | 0.001971 | -0.66138 |
| MUM1L1 | -1.98654 | 0.689393 | -3.66834 | 0.000525 | 0.001992 | -0.67296 |
| CRTAM | -1.00017 | -0.29036 | -3.66561 | 0.00053 | 0.002007 | -0.68112 |
| FABP1 | 3.764032 | -1.62932 | 3.664864 | 0.000531 | 0.002011 | -0.68334 |
| GALNT8 | 1.712922 | 0.101057 | 3.664538 | 0.000532 | 0.002013 | -0.68432 |
| SLC19A3 | 1.0392 | 0.314068 | 3.662339 | 0.000536 | 0.002024 | -0.69088 |
| FRMPD2 | -1.1146 | 0.060648 | -3.66113 | 0.000538 | 0.002032 | -0.69448 |
| TM7SF4 | 1.001182 | -0.10873 | 3.660342 | 0.000539 | 0.002036 | -0.69684 |
| C19orf69 | 2.231707 | 1.104423 | 3.644101 | 0.000567 | 0.002128 | -0.74523 |
| FCGR1B | 1.425567 | -0.78073 | 3.641518 | 0.000572 | 0.002143 | -0.75292 |
| ATP6V1C2 | 1.127658 | 0.067137 | 3.635026 | 0.000584 | 0.002184 | -0.77222 |
| S100A12 | 2.085228 | -0.56539 | 3.634419 | 0.000585 | 0.002186 | -0.77402 |
| ENAM | -1.25756 | 0.386309 | -3.6295 | 0.000594 | 0.002212 | -0.78861 |
| FUT1 | -1.13184 | -0.43528 | -3.62563 | 0.000602 | 0.002235 | -0.80012 |
| EFHA2 | -1.13766 | -0.37547 | -3.61945 | 0.000613 | 0.002271 | -0.81842 |
| NKX6-3 | -1.36341 | -0.24228 | -3.61901 | 0.000614 | 0.002273 | -0.81974 |
| FAM198A | -1.38672 | -0.14393 | -3.61432 | 0.000624 | 0.002299 | -0.83363 |
| EMR3 | 1.301939 | -0.51959 | 3.608105 | 0.000636 | 0.002338 | -0.85201 |
| C3orf32 | 1.250451 | 0.111316 | 3.607613 | 0.000637 | 0.002339 | -0.85347 |
| LOC100130171 | 1.347245 | -0.10948 | 3.605503 | 0.000641 | 0.002353 | -0.8597 |
| OSM | 1.612225 | 0.249967 | 3.604529 | 0.000643 | 0.002359 | -0.86258 |
| ABCC13 | 1.55619 | 0.251652 | 3.600223 | 0.000652 | 0.002387 | -0.8753 |
| CAMK2N1 | 1.182254 | -0.14545 | 3.59688 | 0.000659 | 0.002409 | -0.88517 |
| LOC100505695 | 1.118199 | -0.23608 | 3.580604 | 0.000693 | 0.002524 | -0.93314 |
| CXCL3 | 1.155146 | 0.029854 | 3.574535 | 0.000707 | 0.002565 | -0.95099 |
| DUOXA2 | 1.123616 | -0.0421 | 3.570977 | 0.000715 | 0.00259 | -0.96144 |
| CPZ | 1.007825 | -0.44964 | 3.56482 | 0.000729 | 0.00263 | -0.97952 |
| TREM2 | 1.055352 | -0.2838 | 3.558144 | 0.000744 | 0.002675 | -0.9991 |
| ASPN | -1.23119 | -0.00846 | -3.5545 | 0.000752 | 0.002699 | -1.00977 |
| NRG3 | 1.024931 | 0.092364 | 3.553345 | 0.000755 | 0.002707 | -1.01316 |
| ODAM | 1.287082 | -0.0027 | 3.551897 | 0.000759 | 0.002718 | -1.0174 |
| UPP1 | 1.250753 | -0.15073 | 3.551827 | 0.000759 | 0.002718 | -1.0176 |
| MRVI1-AS1 | 1.009997 | -0.10286 | 3.550401 | 0.000762 | 0.002729 | -1.02178 |
| LGI1 | -1.41607 | 0.743879 | -3.54407 | 0.000777 | 0.002777 | -1.0403 |
| TNFRSF11B | 1.516301 | -0.22111 | 3.543469 | 0.000779 | 0.00278 | -1.04205 |
| FBXL13 | -1.13799 | -0.36363 | -3.53694 | 0.000795 | 0.002831 | -1.06114 |
| PABPC1L | -1.1625 | -0.3039 | -3.53607 | 0.000797 | 0.002837 | -1.06366 |
| DEFB103B | 1.107619 | -0.19586 | 3.533695 | 0.000803 | 0.002853 | -1.0706 |
| PXDN | 1.005659 | -0.11589 | 3.531071 | 0.00081 | 0.002873 | -1.07825 |
| CD160 | -1.32683 | -0.11586 | -3.52903 | 0.000815 | 0.002888 | -1.08421 |
| UGT2B15 | -1.65369 | 0.147058 | -3.49758 | 0.000898 | 0.003144 | -1.17563 |
| SCEL | -1.16963 | 0.070396 | -3.49553 | 0.000904 | 0.003162 | -1.18158 |
| CETN1 | 1.077294 | 0.073285 | 3.493311 | 0.00091 | 0.003179 | -1.188 |
| CHST5 | 1.580467 | 0.118133 | 3.492214 | 0.000913 | 0.003187 | -1.19118 |
| TCAM1P | 1.450849 | 0.299061 | 3.489744 | 0.00092 | 0.003207 | -1.19833 |
| HOXA7 | 1.312632 | -0.32079 | 3.487194 | 0.000928 | 0.003229 | -1.20571 |
| IRX2 | 2.641104 | -0.54267 | 3.483271 | 0.000939 | 0.003261 | -1.21705 |
| KDM5D | 1.841382 | -1.22176 | 3.481087 | 0.000945 | 0.003281 | -1.22337 |
| SCNN1B | -1.30006 | -0.00225 | -3.4787 | 0.000952 | 0.003301 | -1.23025 |
| UCA1 | 1.651278 | -0.19651 | 3.478048 | 0.000954 | 0.003306 | -1.23214 |
| PLA2G12B | 1.458753 | 0.427217 | 3.473311 | 0.000968 | 0.003348 | -1.24582 |
| DDX3Y | 2.178637 | -1.8647 | 3.469417 | 0.00098 | 0.003382 | -1.25705 |
| S100A9 | 1.878321 | -0.50503 | 3.468216 | 0.000984 | 0.003392 | -1.26051 |
| MMP3 | 1.953524 | 0.362401 | 3.46674 | 0.000988 | 0.003404 | -1.26477 |
| KCNJ16 | -1.86545 | 0.325597 | -3.46446 | 0.000995 | 0.003424 | -1.27133 |
| TRIM36 | 1.124648 | 0.201392 | 3.4503 | 0.00104 | 0.003555 | -1.31207 |
| GDAP1L1 | -1.25793 | 0.08162 | -3.4471 | 0.00105 | 0.003584 | -1.32127 |
| CNTN3 | -1.12854 | -0.00183 | -3.44706 | 0.00105 | 0.003584 | -1.32137 |
| OR8H1 | 1.144497 | 0.036238 | 3.437261 | 0.001082 | 0.003674 | -1.34948 |
| CYP3A7 | -1.1144 | -0.10701 | -3.43144 | 0.001102 | 0.003729 | -1.36616 |
| PI3 | 2.040052 | -0.4754 | 3.430896 | 0.001104 | 0.003733 | -1.36771 |
| MAB21L2 | 1.133008 | 0.107231 | 3.430175 | 0.001106 | 0.00374 | -1.36977 |
| HRASLS2 | -1.94527 | -0.59019 | -3.42497 | 0.001124 | 0.003794 | -1.38466 |
| RGS2 | 1.187439 | -0.16853 | 3.424086 | 0.001127 | 0.003801 | -1.38719 |
| TEKT4P2 | 1.346334 | 0.279188 | 3.42252 | 0.001132 | 0.003817 | -1.39166 |
| HMGCS2 | 1.079394 | -0.14315 | 3.416129 | 0.001155 | 0.00388 | -1.40992 |
| S100A7 | 1.058485 | -0.10439 | 3.404405 | 0.001197 | 0.004002 | -1.44334 |
| CHRDL2 | 1.806501 | -0.47991 | 3.403682 | 0.001199 | 0.00401 | -1.4454 |
| MUC12 | 1.357503 | 0.290823 | 3.402453 | 0.001204 | 0.004022 | -1.4489 |
| SLC5A9 | 1.388788 | 0.003743 | 3.399511 | 0.001215 | 0.004055 | -1.45727 |
| ALOX12P2 | -1.01179 | -0.05358 | -3.38898 | 0.001254 | 0.004167 | -1.48719 |
| CCDC144NL | -1.15298 | 0.225373 | -3.38637 | 0.001264 | 0.004197 | -1.49461 |
| C6orf58 | -2.54616 | -0.95054 | -3.37458 | 0.001311 | 0.004326 | -1.52801 |
| GLT1D1 | 1.15003 | -0.28691 | 3.364967 | 0.00135 | 0.004434 | -1.5552 |
| EDN3 | -1.98499 | -0.15679 | -3.35532 | 0.00139 | 0.004544 | -1.58242 |
| LRP8 | 1.311588 | -0.17426 | 3.352268 | 0.001403 | 0.004579 | -1.59103 |
| PITX2 | 1.767802 | 0.592006 | 3.350369 | 0.001411 | 0.0046 | -1.59638 |
| KLK12 | 1.940349 | 0.14457 | 3.347994 | 0.001421 | 0.00463 | -1.60307 |
| SPINK2 | -1.89352 | -0.79549 | -3.34723 | 0.001424 | 0.004639 | -1.60523 |
| DAZL | 3.501628 | -2.50446 | 3.345716 | 0.001431 | 0.004656 | -1.60948 |
| NEU3 | 1.008414 | 0.151185 | 3.344828 | 0.001434 | 0.004666 | -1.61198 |
| S100A8 | 2.03193 | -0.81658 | 3.340308 | 0.001454 | 0.004722 | -1.6247 |
| B3GNT6 | -1.57665 | -0.22051 | -3.33947 | 0.001458 | 0.004732 | -1.62707 |
| TRIM58 | -1.07525 | -0.06762 | -3.33922 | 0.001459 | 0.004734 | -1.62775 |
| ATP1A2 | -1.56858 | 0.259899 | -3.33696 | 0.001469 | 0.004764 | -1.6341 |
| DEFB4A | 1.90032 | 0.407271 | 3.319217 | 0.00155 | 0.004984 | -1.68386 |
| HOTAIRM1 | -1.07588 | 0.033041 | -3.31534 | 0.001568 | 0.005037 | -1.69471 |
| MLF1 | -1.0664 | -0.42881 | -3.31483 | 0.00157 | 0.005043 | -1.69614 |
| CYP2D6 | 1.255003 | 0.29286 | 3.31277 | 0.00158 | 0.005067 | -1.7019 |
| CRIP3 | 1.468841 | -0.29716 | 3.311679 | 0.001585 | 0.005081 | -1.70495 |
| CDX2 | 1.410601 | -0.08917 | 3.307356 | 0.001606 | 0.005137 | -1.71703 |
| C8orf22 | 1.002493 | 0.176887 | 3.306507 | 0.00161 | 0.005148 | -1.7194 |
| HBE1 | 1.090181 | -0.24426 | 3.304671 | 0.001619 | 0.005172 | -1.72452 |
| NETO2 | 1.331629 | -0.10054 | 3.287374 | 0.001705 | 0.0054 | -1.77271 |
| NEB | 1.31756 | 0.143813 | 3.282955 | 0.001728 | 0.005462 | -1.78499 |
| LOC440356 | 1.246936 | 0.213912 | 3.279854 | 0.001744 | 0.005507 | -1.7936 |
| DAZ2 | 2.685506 | -1.58674 | 3.267231 | 0.001811 | 0.005701 | -1.8286 |
| TEX11 | 1.03459 | 0.030669 | 3.263667 | 0.001831 | 0.005752 | -1.83847 |
| DEFA3 | 1.74291 | -0.16098 | 3.258436 | 0.00186 | 0.005827 | -1.85294 |
| GPX3 | -1.06147 | -0.1189 | -3.25603 | 0.001873 | 0.005861 | -1.85958 |
| CXCL17 | -1.14612 | -0.45466 | -3.25307 | 0.00189 | 0.005902 | -1.86777 |
| CXCL5 | 1.976807 | -0.05231 | 3.252585 | 0.001892 | 0.005908 | -1.8691 |
| CDSN | 1.065312 | 0.160718 | 3.247086 | 0.001923 | 0.005996 | -1.88427 |
| STOML3 | 1.050159 | 0.318517 | 3.245067 | 0.001935 | 0.006022 | -1.88984 |
| AIM1L | 1.088573 | 0.00139 | 3.242734 | 0.001949 | 0.006052 | -1.89627 |
| CKMT2 | -1.61146 | 0.018516 | -3.24007 | 0.001964 | 0.006094 | -1.9036 |
| HP | 1.248961 | 0.129661 | 3.238959 | 0.001971 | 0.006114 | -1.90666 |
| SFRP2 | -1.16164 | 0.176344 | -3.23332 | 0.002004 | 0.006204 | -1.92217 |
| APOBEC2 | -1.17566 | 0.049719 | -3.22889 | 0.00203 | 0.006274 | -1.93433 |
| LOC400891 | 1.174792 | 0.448801 | 3.228507 | 0.002033 | 0.006279 | -1.9354 |
| C14orf34 | -1.27237 | -0.21791 | -3.22797 | 0.002036 | 0.006286 | -1.93687 |
| C12orf56 | 1.168045 | 0.128267 | 3.227811 | 0.002037 | 0.006287 | -1.93731 |
| AQP9 | 1.843722 | -0.41212 | 3.226537 | 0.002045 | 0.006308 | -1.94081 |
| SEMG1 | 1.820955 | -0.13216 | 3.226478 | 0.002045 | 0.006308 | -1.94097 |
| CYR61 | 1.153399 | -0.07685 | 3.22585 | 0.002049 | 0.006316 | -1.94269 |
| ZYG11A | 1.004543 | 0.145052 | 3.225601 | 0.00205 | 0.006319 | -1.94338 |
| GIF | -3.17609 | 0.453129 | -3.22405 | 0.00206 | 0.006345 | -1.94762 |
| PLA2G2A | 1.749183 | 0.515619 | 3.21502 | 0.002116 | 0.006494 | -1.97238 |
| MGC24103 | -1.13648 | 0.107861 | -3.21084 | 0.002142 | 0.006554 | -1.98383 |
| LRRC17 | -1.29503 | -0.13872 | -3.2011 | 0.002204 | 0.006711 | -2.01045 |
| C1QTNF1 | 1.016901 | 0.046594 | 3.199715 | 0.002213 | 0.006732 | -2.01422 |
| C21orf37 | 1.160589 | 0.400459 | 3.196729 | 0.002233 | 0.006786 | -2.02237 |
| HPGD | -1.04121 | -0.30273 | -3.1951 | 0.002244 | 0.006814 | -2.02681 |
| CTTNBP2 | 1.052355 | 0.032264 | 3.18522 | 0.00231 | 0.006992 | -2.05372 |
| KLK11 | -1.30705 | -0.1444 | -3.18489 | 0.002312 | 0.006997 | -2.05463 |
| FCGR3A | 1.278051 | -0.52459 | 3.1844 | 0.002316 | 0.007001 | -2.05595 |
| APOA1 | 1.633171 | 0.535942 | 3.183392 | 0.002323 | 0.007018 | -2.0587 |
| FPR2 | 1.347905 | -0.19587 | 3.178813 | 0.002354 | 0.007104 | -2.07114 |
| EFNA3 | 1.043086 | 0.396128 | 3.173767 | 0.002389 | 0.007189 | -2.08484 |
| GPR158 | 1.136473 | -0.1168 | 3.168022 | 0.00243 | 0.007288 | -2.10042 |
| GJA8 | 1.066065 | -0.17619 | 3.167335 | 0.002435 | 0.007301 | -2.10228 |
| ABCA8 | -1.0604 | -0.04266 | -3.1641 | 0.002458 | 0.007359 | -2.11105 |
| NMUR2 | 1.52952 | 0.058209 | 3.16111 | 0.00248 | 0.007411 | -2.11914 |
| DKK1 | -1.75119 | -0.89073 | -3.16058 | 0.002483 | 0.00742 | -2.12057 |
| FAM155B | -1.12228 | -0.28661 | -3.15358 | 0.002535 | 0.007551 | -2.13949 |
| TCN1 | -1.5531 | -0.23863 | -3.14801 | 0.002577 | 0.007653 | -2.15454 |
| HPR | 1.373671 | 0.197336 | 3.139889 | 0.002638 | 0.007803 | -2.17643 |
| WASF3 | -1.07717 | -0.41211 | -3.13413 | 0.002683 | 0.007918 | -2.19194 |
| GPR133 | -1.01076 | 0.34704 | -3.12909 | 0.002723 | 0.008015 | -2.20547 |
| RBP2 | 2.860704 | -0.60751 | 3.127629 | 0.002734 | 0.008045 | -2.2094 |
| TXLNG2P | 1.765174 | -1.12774 | 3.127411 | 0.002736 | 0.008048 | -2.20999 |
| UTY | 1.343062 | -0.37314 | 3.125616 | 0.002751 | 0.008084 | -2.21481 |
| GPM6B | -1.18414 | -0.22441 | -3.12377 | 0.002765 | 0.008118 | -2.21977 |
| KIAA1324L | -1.61099 | -0.46934 | -3.12333 | 0.002769 | 0.008125 | -2.22095 |
| CCDC17 | -1.03454 | -0.242 | -3.11417 | 0.002844 | 0.00832 | -2.24549 |
| DLX4 | 1.048434 | -0.06381 | 3.10342 | 0.002934 | 0.008533 | -2.27424 |
| CRABP2 | 1.541312 | 0.248668 | 3.10177 | 0.002948 | 0.008564 | -2.27865 |
| AMPD1 | -1.54286 | -0.27544 | -3.09952 | 0.002967 | 0.008616 | -2.28466 |
| EREG | 1.084463 | -0.09424 | 3.099347 | 0.002969 | 0.008618 | -2.28512 |
| C8orf51 | 1.017341 | -0.01097 | 3.090512 | 0.003046 | 0.008817 | -2.30867 |
| RANBP17 | 1.153938 | -0.10212 | 3.084267 | 0.003101 | 0.008953 | -2.32529 |
| MDGA1 | -1.01387 | -0.55529 | -3.08261 | 0.003116 | 0.00899 | -2.32968 |
| CXCR2P1 | 1.061235 | -0.05188 | 3.082528 | 0.003117 | 0.008991 | -2.32991 |
| UGT2A3 | 1.092852 | 0.340348 | 3.080554 | 0.003134 | 0.009037 | -2.33516 |
| REG4 | 1.819441 | -0.27784 | 3.071163 | 0.003221 | 0.009249 | -2.36008 |
| ALPI | 1.150684 | 0.319042 | 3.067513 | 0.003255 | 0.009334 | -2.36975 |
| SCRG1 | -1.8279 | -0.75334 | -3.04989 | 0.003424 | 0.009741 | -2.41632 |
| KRT16P3 | 1.168255 | 0.194186 | 3.045437 | 0.003468 | 0.009853 | -2.42807 |
| FAM19A5 | -1.19253 | -0.19894 | -3.04251 | 0.003497 | 0.009919 | -2.43577 |
| WDR16 | 1.100049 | 0.465166 | 3.035762 | 0.003565 | 0.010083 | -2.45353 |
| ULBP2 | 1.115583 | -0.04844 | 3.032 | 0.003604 | 0.010168 | -2.46342 |
| ESX1 | 1.210393 | 0.504744 | 3.027322 | 0.003652 | 0.010275 | -2.4757 |
| DSG4 | -1.08902 | 0.434675 | -3.02609 | 0.003665 | 0.010304 | -2.47891 |
| CXCL2 | 1.4372 | -0.16375 | 3.02562 | 0.00367 | 0.010314 | -2.48016 |
| FAP | 1.27296 | -0.42467 | 3.01801 | 0.003751 | 0.010506 | -2.5001 |
| ARVCF | 1.130637 | 0.113358 | 3.016676 | 0.003765 | 0.010541 | -2.50359 |
| FAM176A | 1.139694 | -0.00056 | 3.013133 | 0.003803 | 0.010641 | -2.51286 |
| RFX6 | -1.34999 | -0.27449 | -3.00239 | 0.003922 | 0.010915 | -2.5409 |
| TTTY14 | 1.530471 | -0.25493 | 2.998703 | 0.003963 | 0.011014 | -2.55052 |
| ENPP5 | -1.16307 | -0.2879 | -2.99859 | 0.003964 | 0.011016 | -2.55081 |
| KLRC2 | -1.0118 | -0.11274 | -2.99141 | 0.004046 | 0.01119 | -2.56951 |
| RNF212 | -1.08411 | -0.10933 | -2.99137 | 0.004046 | 0.01119 | -2.56959 |
| CASP14L | 1.712796 | 0.63297 | 2.991223 | 0.004048 | 0.011193 | -2.56999 |
| LOC150622 | -1.14801 | 0.377654 | -2.97441 | 0.004246 | 0.011658 | -2.61362 |
| TNFRSF6B | 1.318918 | 0.246966 | 2.965521 | 0.004354 | 0.011915 | -2.63662 |
| LRRC3B | -1.05176 | 0.624225 | -2.95292 | 0.004511 | 0.01229 | -2.66914 |
| LOC728012 | -1.05668 | 0.322426 | -2.94754 | 0.00458 | 0.012451 | -2.68297 |
| REG3G | 2.115883 | -0.57522 | 2.940503 | 0.004671 | 0.012642 | -2.70107 |
| FPR1 | 1.418227 | -0.56724 | 2.931431 | 0.004792 | 0.012924 | -2.72434 |
| MIA | 1.23392 | -0.58758 | 2.930735 | 0.004801 | 0.012944 | -2.72612 |
| CLEC4E | 1.416104 | -0.61684 | 2.926652 | 0.004857 | 0.013066 | -2.73657 |
| ATP4A | -3.8518 | 1.336563 | -2.92329 | 0.004903 | 0.013174 | -2.74518 |
| LTF | -1.83765 | -0.63264 | -2.9082 | 0.005114 | 0.013646 | -2.78368 |
| MUC17 | 1.064071 | 0.108871 | 2.890935 | 0.005366 | 0.014191 | -2.82756 |
| OTOP3 | 1.464745 | 0.675505 | 2.888936 | 0.005396 | 0.014255 | -2.83263 |
| RNF186 | 1.152418 | -0.13099 | 2.881878 | 0.005503 | 0.014492 | -2.8505 |
| KIAA1257 | 1.024633 | 0.518115 | 2.879754 | 0.005535 | 0.014565 | -2.85587 |
| GPA33 | 1.257423 | 0.339224 | 2.878948 | 0.005548 | 0.014593 | -2.85791 |
| FBLL1 | -1.30548 | -0.41581 | -2.86977 | 0.005691 | 0.014906 | -2.88109 |
| ONECUT1 | -1.07583 | -0.03945 | -2.86937 | 0.005697 | 0.014916 | -2.88208 |
| FBP2 | -1.00945 | -0.11457 | -2.86555 | 0.005757 | 0.015039 | -2.89171 |
| KRT6B | 1.796913 | 0.042234 | 2.858587 | 0.005869 | 0.015278 | -2.90924 |
| ADORA2B | 1.051651 | -0.02494 | 2.849928 | 0.006011 | 0.015592 | -2.93099 |
| LILRA2 | 1.024743 | -0.21951 | 2.837848 | 0.006215 | 0.016065 | -2.96125 |
| ATP4B | -3.14171 | 1.076197 | -2.82899 | 0.006368 | 0.016401 | -2.98336 |
| PDZK1 | 1.309258 | 0.034298 | 2.790363 | 0.007077 | 0.017939 | -3.07923 |
| MTTP | 2.158632 | 0.4914 | 2.787117 | 0.00714 | 0.018066 | -3.08724 |
| TMEM213 | 1.008075 | 0.417717 | 2.786773 | 0.007147 | 0.018076 | -3.08809 |
| EGR3 | 1.156316 | -0.52391 | 2.785513 | 0.007171 | 0.018127 | -3.0912 |
| SPINK13 | -1.55649 | 0.097452 | -2.78119 | 0.007256 | 0.018304 | -3.10185 |
| IGFL2 | 1.41975 | 0.507072 | 2.779953 | 0.007281 | 0.018355 | -3.1049 |
| ANO3 | -1.18792 | 0.577503 | -2.76863 | 0.007508 | 0.018819 | -3.13273 |
| TACR1 | 1.042777 | 0.303146 | 2.765436 | 0.007573 | 0.018957 | -3.14056 |
| OXGR1 | 1.078597 | 0.46032 | 2.760715 | 0.00767 | 0.019159 | -3.15213 |
| NCRNA00185 | 2.647863 | -1.44927 | 2.76067 | 0.007671 | 0.019159 | -3.15224 |
| CHI3L1 | 1.415513 | -0.31297 | 2.756187 | 0.007765 | 0.019369 | -3.16321 |
| CST6 | 1.158192 | -0.08809 | 2.68846 | 0.009312 | 0.022592 | -3.32727 |
| CHIA | 1.291556 | 0.276699 | 2.688007 | 0.009323 | 0.022614 | -3.32835 |
| DUSP27 | 1.106012 | 0.38338 | 2.653916 | 0.010206 | 0.024408 | -3.40972 |
| MLIP | 1.002917 | 0.102217 | 2.634018 | 0.010755 | 0.025503 | -3.45683 |
| GREM1 | -1.72806 | 0.025264 | -2.61486 | 0.01131 | 0.026598 | -3.50194 |
| MSMB | -1.98252 | -0.69264 | -2.6109 | 0.011427 | 0.02682 | -3.51122 |
| BTNL3 | 2.001583 | -0.98749 | 2.604566 | 0.011618 | 0.027181 | -3.52606 |
| GUCY1B2 | 1.30527 | -0.07736 | 2.586788 | 0.012169 | 0.028275 | -3.56755 |
| NEURL3 | 1.254193 | -0.60105 | 2.563399 | 0.01293 | 0.029771 | -3.62178 |
| GPER | -1.03823 | -0.13224 | -2.5454 | 0.013545 | 0.03093 | -3.66324 |
| SLC25A41 | -1.0802 | 0.05018 | -2.52831 | 0.014153 | 0.032151 | -3.70242 |
| CXCR1 | 1.11079 | -0.4636 | 2.516787 | 0.014577 | 0.032971 | -3.7287 |
| FLJ42393 | -1.21676 | 0.606499 | -2.51012 | 0.014827 | 0.033451 | -3.74387 |
| APOB | 1.534722 | 0.652594 | 2.504342 | 0.015047 | 0.033864 | -3.75698 |
| DUOXA1 | -1.24873 | -0.30351 | -2.49181 | 0.015535 | 0.034765 | -3.78534 |
| ABCG2 | 1.033538 | 0.145711 | 2.474734 | 0.016222 | 0.036089 | -3.82381 |
| A4GNT | -1.53986 | 0.195896 | -2.47095 | 0.016378 | 0.03637 | -3.83231 |
| INSL5 | 1.158559 | 0.553053 | 2.463287 | 0.016698 | 0.036949 | -3.84947 |
| C3P1 | 1.226334 | 0.552431 | 2.452851 | 0.017143 | 0.037767 | -3.87278 |
| COL2A1 | -1.58637 | 1.179905 | -2.43774 | 0.017807 | 0.038975 | -3.9064 |
| VSNL1 | -1.11605 | -0.26088 | -2.42512 | 0.018378 | 0.040034 | -3.93433 |
| VCX | -1.01172 | 0.187669 | -2.42077 | 0.018578 | 0.040429 | -3.94394 |
| IL13RA2 | 1.081771 | 0.062566 | 2.409895 | 0.019089 | 0.041378 | -3.96789 |
| SLC7A9 | 1.400111 | 0.467959 | 2.385552 | 0.020278 | 0.043543 | -4.02118 |
| GC | -1.18302 | -0.14837 | -2.38306 | 0.020403 | 0.043761 | -4.02661 |
| ABCA12 | 1.019628 | -0.04806 | 2.377269 | 0.020697 | 0.044303 | -4.03921 |
| GKN2 | -1.36061 | -0.6671 | -2.37157 | 0.02099 | 0.044805 | -4.05158 |
| HOXC10 | 1.258138 | 0.376572 | 2.370494 | 0.021046 | 0.044887 | -4.05392 |
| CGA | -1.15273 | -0.12606 | -2.36964 | 0.02109 | 0.044947 | -4.05577 |
| CMTM2 | 1.311202 | -0.32224 | 2.36663 | 0.021247 | 0.045222 | -4.06229 |
| C8G | 1.054811 | 0.161295 | 2.366421 | 0.021258 | 0.04524 | -4.06274 |
