# Supplementary material for: Explore the active ingredients and potential mechanisms of JianPi QingRe HuaYu Methods in the treatment of gastric inflammation-cancer transformation by network pharmacology and experimental validation
Source: BMC Complement Med Ther. 2023 Nov 14;23:411. doi: 10.1186/s12906-023-04232-0 (PMC10644588; doi:10.1186/s12906-023-04232-0)
Supplement: Supplementary file 6 — Additional file 6: Table S6. The DEGs between EGC and chronic gastritis group in GSE55696 dataset. [file 12906_2023_4232_MOESM6_ESM.docx]

**Table S6. The DEGs between EGC and chronic gastritis group in GSE55696 dataset.**

| Genes | logFC | AveExpr | t | P.Value | adj.P.Val | B |
| --- | --- | --- | --- | --- | --- | --- |
| BCL2L2 | -1.19013 | 0.379622 | -12.3714 | 4.64E-15 | 8.52E-11 | 24.04153 |
| ALDOC | -3.45646 | 0.68141 | -11.6317 | 3.11E-14 | 2.85E-10 | 22.22735 |
| C15orf17 | -1.43282 | 0.544571 | -11.317 | 7.12E-14 | 3.46E-10 | 21.43268 |
| ZNF211 | -1.91588 | 0.605695 | -11.2956 | 7.53E-14 | 3.46E-10 | 21.37803 |
| STS | -2.67293 | 0.784333 | -11.1301 | 1.17E-13 | 4.30E-10 | 20.95393 |
| BCAR1 | -1.76265 | 0.406299 | -10.8811 | 2.29E-13 | 5.52E-10 | 20.30864 |
| FLNB | -2.12788 | 0.469319 | -10.8636 | 2.41E-13 | 5.52E-10 | 20.26302 |
| ZBTB48 | -1.20948 | 0.311589 | -10.5112 | 6.31E-13 | 1.26E-09 | 19.33403 |
| FOXJ2 | -1.46383 | 0.330561 | -10.4638 | 7.19E-13 | 1.26E-09 | 19.20776 |
| CYTH1 | -1.19764 | 0.500135 | -10.4356 | 7.78E-13 | 1.26E-09 | 19.13238 |
| SLC45A4 | -1.76786 | 0.482976 | -10.387 | 8.90E-13 | 1.26E-09 | 19.00233 |
| CLIP2 | -1.55515 | 0.431522 | -10.3512 | 9.83E-13 | 1.27E-09 | 18.90653 |
| ABCC5 | -2.67704 | 0.622826 | -10.3326 | 1.04E-12 | 1.27E-09 | 18.85648 |
| TSC2 | -1.11054 | 0.251011 | -10.2231 | 1.41E-12 | 1.61E-09 | 18.5616 |
| MBD6 | -1.17581 | 0.322787 | -10.038 | 2.36E-12 | 2.55E-09 | 18.05898 |
| SUN1 | -1.69381 | 0.529117 | -9.9772 | 2.81E-12 | 2.73E-09 | 17.89297 |
| PPFIBP1 | -1.32421 | 0.243407 | -9.86922 | 3.81E-12 | 3.33E-09 | 17.59673 |
| LOC100506328 | -3.99472 | 0.693909 | -9.8465 | 4.07E-12 | 3.39E-09 | 17.53418 |
| AGSK1 | -1.957 | 0.571125 | -9.78456 | 4.85E-12 | 3.87E-09 | 17.36333 |
| RIPK4 | -1.72621 | 0.336488 | -9.5793 | 8.74E-12 | 6.42E-09 | 16.79335 |
| CLASRP | -1.66193 | 0.654241 | -9.54976 | 9.52E-12 | 6.72E-09 | 16.71083 |
| SGSM3 | -1.81571 | 0.352298 | -9.52537 | 1.02E-11 | 6.94E-09 | 16.64264 |
| CTNNBL1 | -2.08905 | 0.360304 | -9.47932 | 1.17E-11 | 7.62E-09 | 16.51364 |
| KCNK10 | -2.17271 | 0.380169 | -9.46847 | 1.20E-11 | 7.62E-09 | 16.48319 |
| SIX1 | 4.349189 | -1.0937 | 9.433914 | 1.33E-11 | 8.00E-09 | 16.38614 |
| DOCK6 | -1.0779 | 0.334136 | -9.41893 | 1.39E-11 | 8.00E-09 | 16.34402 |
| SYTL4 | -2.00457 | 0.276714 | -9.41757 | 1.40E-11 | 8.00E-09 | 16.34018 |
| SLC39A11 | -1.76235 | 0.364253 | -9.38453 | 1.54E-11 | 8.30E-09 | 16.24718 |
| SLC9A1 | -2.38504 | 0.50866 | -9.38401 | 1.54E-11 | 8.30E-09 | 16.24572 |
| SCAMP4 | -1.33019 | 0.314818 | -9.29956 | 1.97E-11 | 9.59E-09 | 16.0073 |
| IFT140 | -1.62621 | 0.228526 | -9.29378 | 2.00E-11 | 9.59E-09 | 15.99093 |
| HOOK2 | -1.39398 | 0.305053 | -9.28152 | 2.07E-11 | 9.59E-09 | 15.95623 |
| ABLIM1 | -1.6335 | 0.236456 | -9.28119 | 2.08E-11 | 9.59E-09 | 15.95531 |
| SIRT7 | -1.22952 | 0.161937 | -9.27892 | 2.09E-11 | 9.59E-09 | 15.94889 |
| NPAS1 | -2.66553 | 0.754729 | -9.25623 | 2.23E-11 | 9.76E-09 | 15.88459 |
| ALDH3A2 | -2.06475 | 0.393793 | -9.22374 | 2.46E-11 | 1.04E-08 | 15.79243 |
| TAGLN2 | -1.82744 | 0.252313 | -9.21408 | 2.53E-11 | 1.04E-08 | 15.76499 |
| NR4A2 | 2.385386 | -0.38757 | 9.210179 | 2.55E-11 | 1.04E-08 | 15.75392 |
| KIAA0556 | -1.5402 | 0.405662 | -9.20197 | 2.62E-11 | 1.04E-08 | 15.73059 |
| ANO7 | -3.11004 | 0.850841 | -9.18052 | 2.79E-11 | 1.06E-08 | 15.66961 |
| LOC100129034 | -1.37339 | 0.276784 | -9.16563 | 2.91E-11 | 1.06E-08 | 15.62725 |
| TBC1D22A | -1.08913 | 0.136257 | -9.15421 | 3.01E-11 | 1.06E-08 | 15.59473 |
| KIAA0195 | -1.43804 | 0.316194 | -9.14796 | 3.07E-11 | 1.06E-08 | 15.57691 |
| PELI2 | -2.14524 | 0.548836 | -9.14713 | 3.07E-11 | 1.06E-08 | 15.57456 |
| CAPN8 | -2.36292 | 0.491258 | -9.13156 | 3.22E-11 | 1.09E-08 | 15.53017 |
| RGS1 | 3.070133 | 0.017484 | 9.122399 | 3.30E-11 | 1.10E-08 | 15.50405 |
| RALGPS1 | -1.31046 | 0.254092 | -9.10492 | 3.48E-11 | 1.14E-08 | 15.45419 |
| DGKD | -1.9026 | 0.362962 | -9.03695 | 4.25E-11 | 1.34E-08 | 15.25985 |
| KIAA1147 | -1.929 | 0.352005 | -9.01662 | 4.51E-11 | 1.40E-08 | 15.20159 |
| LTK | -2.6449 | 0.720692 | -8.99428 | 4.82E-11 | 1.47E-08 | 15.13754 |
| PLXNA2 | -1.77476 | 0.213042 | -8.98138 | 5.01E-11 | 1.51E-08 | 15.1005 |
| OTUD1 | -2.00725 | 0.550796 | -8.9662 | 5.24E-11 | 1.55E-08 | 15.05691 |
| AP1G2 | -1.03738 | 0.304273 | -8.9492 | 5.51E-11 | 1.59E-08 | 15.00807 |
| SREBF2 | -1.15603 | 0.066993 | -8.94732 | 5.54E-11 | 1.59E-08 | 15.00266 |
| MTMR3 | -1.11306 | 0.458094 | -8.93357 | 5.77E-11 | 1.59E-08 | 14.96312 |
| ULK3 | -1.07331 | 0.326027 | -8.93169 | 5.80E-11 | 1.59E-08 | 14.95769 |
| ESRP2 | -1.22497 | 0.296226 | -8.93072 | 5.82E-11 | 1.59E-08 | 14.9549 |
| KHNYN | -1.20599 | 0.319402 | -8.92498 | 5.92E-11 | 1.60E-08 | 14.9384 |
| TMEM106A | -1.34014 | 0.489095 | -8.90507 | 6.28E-11 | 1.67E-08 | 14.88106 |
| CDC42SE1 | -1.25884 | 0.335346 | -8.89929 | 6.39E-11 | 1.67E-08 | 14.86442 |
| OSBPL7 | -2.1209 | 0.413166 | -8.87221 | 6.92E-11 | 1.79E-08 | 14.78634 |
| AHNAK | -1.586 | 0.251244 | -8.82518 | 7.96E-11 | 2.03E-08 | 14.65051 |
| LOC388152 | -1.52307 | 0.448408 | -8.80548 | 8.44E-11 | 2.09E-08 | 14.59356 |
| UGDH | -1.95032 | 0.398907 | -8.80013 | 8.57E-11 | 2.10E-08 | 14.57806 |
| CHFR | -1.36544 | 0.154221 | -8.77179 | 9.33E-11 | 2.18E-08 | 14.49598 |
| CARNS1 | -4.21287 | 0.954635 | -8.7617 | 9.61E-11 | 2.18E-08 | 14.46676 |
| FOXA2 | -1.62796 | 0.398099 | -8.76095 | 9.64E-11 | 2.18E-08 | 14.46456 |
| TSPAN5 | -2.25846 | 0.413174 | -8.75896 | 9.69E-11 | 2.18E-08 | 14.4588 |
| ASCC2 | -1.29069 | 0.174251 | -8.75724 | 9.74E-11 | 2.18E-08 | 14.45381 |
| MICALL2 | -1.32821 | 0.342187 | -8.75034 | 9.95E-11 | 2.19E-08 | 14.43381 |
| PRDM16 | -2.30683 | 0.55433 | -8.74481 | 1.01E-10 | 2.19E-08 | 14.41777 |
| PKN1 | -1.28895 | 0.343285 | -8.74443 | 1.01E-10 | 2.19E-08 | 14.41666 |
| SCGB1D1 | -3.83816 | 1.41588 | -8.6689 | 1.27E-10 | 2.68E-08 | 14.19719 |
| ZSCAN4 | -3.6115 | 0.699377 | -8.63871 | 1.39E-10 | 2.90E-08 | 14.10927 |
| SEC16A | -1.09794 | 0.231249 | -8.62244 | 1.46E-10 | 3.01E-08 | 14.06181 |
| CYP4F8 | -1.55326 | 0.132746 | -8.59343 | 1.59E-10 | 3.17E-08 | 13.97716 |
| CCDC42B | -2.29673 | 0.508242 | -8.57461 | 1.68E-10 | 3.29E-08 | 13.9222 |
| FZD5 | -1.56429 | 0.290421 | -8.57101 | 1.70E-10 | 3.29E-08 | 13.91168 |
| LOC399900 | -1.85853 | 0.574756 | -8.53754 | 1.88E-10 | 3.52E-08 | 13.81379 |
| SGSM1 | -2.50923 | 0.608595 | -8.51796 | 2.00E-10 | 3.70E-08 | 13.75647 |
| ELF4 | -1.23142 | 0.017921 | -8.50101 | 2.10E-10 | 3.85E-08 | 13.70678 |
| NPW | -3.11067 | 0.781113 | -8.48532 | 2.20E-10 | 4.00E-08 | 13.66079 |
| RAB6B | -1.87127 | 0.188717 | -8.45448 | 2.42E-10 | 4.20E-08 | 13.57028 |
| FOSL2 | -1.56627 | 0.368415 | -8.45337 | 2.42E-10 | 4.20E-08 | 13.56701 |
| ZNF274 | -1.13897 | 0.177965 | -8.45003 | 2.45E-10 | 4.20E-08 | 13.55719 |
| IGSF9 | -1.96037 | 0.2265 | -8.44839 | 2.46E-10 | 4.20E-08 | 13.55237 |
| C10orf47 | -1.15381 | 0.086087 | -8.44674 | 2.47E-10 | 4.20E-08 | 13.54752 |
| ELOVL6 | -1.6898 | 0.121789 | -8.43916 | 2.53E-10 | 4.26E-08 | 13.52526 |
| CARD14 | 1.996622 | -0.49758 | 8.436263 | 2.55E-10 | 4.26E-08 | 13.51675 |
| ANKRD7 | 1.587526 | -0.22326 | 8.418165 | 2.70E-10 | 4.38E-08 | 13.46354 |
| PDXDC1 | -1.00515 | 0.056296 | -8.41812 | 2.70E-10 | 4.38E-08 | 13.46342 |
| ZNF134 | -1.80399 | 0.218661 | -8.41026 | 2.76E-10 | 4.41E-08 | 13.44028 |
| ARHGEF16 | -1.13834 | 0.181925 | -8.41024 | 2.76E-10 | 4.41E-08 | 13.44024 |
| SLC9A3R2 | -1.81528 | 0.501164 | -8.39841 | 2.86E-10 | 4.53E-08 | 13.40543 |
| TNKS1BP1 | -1.17394 | 0.277261 | -8.38955 | 2.94E-10 | 4.57E-08 | 13.37934 |
| CTNND1 | -1.12087 | 0.075453 | -8.36599 | 3.16E-10 | 4.83E-08 | 13.30991 |
| GOLGA6L9 | -1.8238 | 0.691261 | -8.36036 | 3.21E-10 | 4.86E-08 | 13.29333 |
| FHDC1 | -1.69939 | 0.071239 | -8.35842 | 3.23E-10 | 4.86E-08 | 13.28759 |
| KAT6B | -1.06279 | 0.189598 | -8.34012 | 3.42E-10 | 5.05E-08 | 13.23363 |
| B4GALNT3 | -2.1588 | 0.5185 | -8.33666 | 3.45E-10 | 5.07E-08 | 13.22343 |
| RENBP | 1.061365 | -0.33164 | 8.32625 | 3.56E-10 | 5.14E-08 | 13.1927 |
| OVOL2 | -1.17897 | 0.029359 | -8.30374 | 3.81E-10 | 5.38E-08 | 13.12623 |
| LPCAT4 | -1.26602 | -0.01923 | -8.29699 | 3.89E-10 | 5.44E-08 | 13.10626 |
| MAGIX | -1.04023 | 0.132309 | -8.29406 | 3.93E-10 | 5.44E-08 | 13.09761 |
| COX19 | -1.0511 | 0.232161 | -8.29303 | 3.94E-10 | 5.44E-08 | 13.09458 |
| FAM101A | -1.77936 | 0.239463 | -8.28518 | 4.04E-10 | 5.48E-08 | 13.07137 |
| RAB37 | -2.44653 | 0.681852 | -8.28346 | 4.06E-10 | 5.48E-08 | 13.06627 |
| ITPR3 | -1.1691 | 0.096921 | -8.28279 | 4.06E-10 | 5.48E-08 | 13.06428 |
| RASAL1 | -1.73884 | 0.326672 | -8.28062 | 4.09E-10 | 5.48E-08 | 13.05787 |
| EPDR1 | -2.51607 | 0.283759 | -8.258 | 4.38E-10 | 5.74E-08 | 12.99095 |
| CDK5RAP3 | -1.00392 | 0.392791 | -8.24147 | 4.61E-10 | 6.00E-08 | 12.942 |
| SLC44A2 | -1.58818 | 0.311452 | -8.23459 | 4.71E-10 | 6.08E-08 | 12.92163 |
| LOC727849 | -1.76743 | 0.509355 | -8.23249 | 4.74E-10 | 6.08E-08 | 12.91541 |
| TTC39A | -1.24211 | 0.090018 | -8.21711 | 4.96E-10 | 6.33E-08 | 12.86981 |
| MIA3 | -1.31824 | 0.214043 | -8.19735 | 5.27E-10 | 6.63E-08 | 12.81121 |
| CRIPAK | -1.31216 | 0.440647 | -8.19063 | 5.38E-10 | 6.72E-08 | 12.79129 |
| PER3 | -3.26353 | 0.654093 | -8.18355 | 5.50E-10 | 6.82E-08 | 12.77026 |
| NAGPA | -1.41066 | 0.334753 | -8.17422 | 5.66E-10 | 6.97E-08 | 12.74257 |
| PDZD3 | -2.83479 | 0.989681 | -8.16617 | 5.80E-10 | 7.02E-08 | 12.71865 |
| SLCO4A1 | -1.42683 | 0.1691 | -8.1651 | 5.82E-10 | 7.02E-08 | 12.71548 |
| FNIP2 | -1.01794 | 0.198828 | -8.15318 | 6.03E-10 | 7.14E-08 | 12.68006 |
| CCDC88B | -1.25352 | 0.396061 | -8.14773 | 6.13E-10 | 7.17E-08 | 12.66388 |
| KIAA0182 | -1.09829 | 0.210446 | -8.13651 | 6.35E-10 | 7.37E-08 | 12.63052 |
| MB21D2 | -1.86867 | 0.190124 | -8.11862 | 6.70E-10 | 7.69E-08 | 12.57727 |
| FMN1 | -1.90748 | 0.386699 | -8.09496 | 7.21E-10 | 8.17E-08 | 12.50683 |
| SARM1 | -1.31773 | 0.196588 | -8.09455 | 7.21E-10 | 8.17E-08 | 12.50561 |
| PCDHGA12 | -1.07285 | 0.310203 | -8.08802 | 7.36E-10 | 8.29E-08 | 12.48618 |
| BAAT | 2.244941 | 0.053777 | 8.075752 | 7.64E-10 | 8.55E-08 | 12.44962 |
| MEIS1 | -2.38891 | 0.409229 | -8.06661 | 7.86E-10 | 8.74E-08 | 12.42236 |
| MANSC1 | -1.49637 | 0.125872 | -8.04076 | 8.51E-10 | 9.24E-08 | 12.34527 |
| USH1C | -1.34936 | 0.128644 | -8.03671 | 8.61E-10 | 9.30E-08 | 12.33315 |
| ANG | -1.4145 | 0.180532 | -8.03282 | 8.72E-10 | 9.35E-08 | 12.32156 |
| NET1 | -1.26536 | 0.245404 | -8.02872 | 8.83E-10 | 9.40E-08 | 12.30931 |
| GLDN | -3.64038 | 0.751031 | -8.0274 | 8.86E-10 | 9.40E-08 | 12.30537 |
| CAPN13 | -2.11275 | 0.190631 | -8.02551 | 8.91E-10 | 9.40E-08 | 12.29972 |
| SIM2 | 2.590196 | -0.82861 | 8.023784 | 8.96E-10 | 9.40E-08 | 12.29458 |
| PRKCD | -1.5262 | 0.317239 | -8.02132 | 9.03E-10 | 9.41E-08 | 12.28721 |
| MAT2A | -1.09068 | 0.36616 | -8.00653 | 9.45E-10 | 9.74E-08 | 12.24303 |
| RHOBTB2 | -1.18398 | 0.170269 | -7.99415 | 9.81E-10 | 9.94E-08 | 12.20602 |
| C6orf141 | -1.5492 | 0.367129 | -7.9909 | 9.91E-10 | 9.94E-08 | 12.19632 |
| SLC27A3 | -1.49533 | 0.469099 | -7.98368 | 1.01E-09 | 1.01E-07 | 12.17474 |
| KLHL3 | -1.86265 | 0.519201 | -7.97239 | 1.05E-09 | 1.02E-07 | 12.14096 |
| SH3BGRL2 | -1.32716 | 0.153484 | -7.95101 | 1.12E-09 | 1.09E-07 | 12.07697 |
| MOGS | -1.02395 | 0.304725 | -7.94974 | 1.12E-09 | 1.09E-07 | 12.07318 |
| ERMAP | -1.25813 | 0.051984 | -7.93062 | 1.19E-09 | 1.13E-07 | 12.01589 |
| IL1A | 1.71503 | -0.36654 | 7.926648 | 1.21E-09 | 1.13E-07 | 12.00399 |
| RCAN3 | -1.09828 | 0.308566 | -7.92342 | 1.22E-09 | 1.14E-07 | 11.99432 |
| C6orf132 | -1.43309 | 0.22142 | -7.91206 | 1.26E-09 | 1.17E-07 | 11.96027 |
| LASP1 | -1.13693 | 0.091257 | -7.90933 | 1.27E-09 | 1.17E-07 | 11.95207 |
| FOXA3 | -1.57529 | 0.151673 | -7.89326 | 1.34E-09 | 1.23E-07 | 11.90388 |
| SPIRE2 | -1.41542 | 0.075 | -7.88772 | 1.36E-09 | 1.24E-07 | 11.88727 |
| ARHGAP21 | -1.06911 | 0.005439 | -7.88429 | 1.38E-09 | 1.24E-07 | 11.87697 |
| RNASE4 | -1.67568 | 0.339058 | -7.87027 | 1.44E-09 | 1.28E-07 | 11.83487 |
| LOC728978 | -3.39499 | 0.416174 | -7.86955 | 1.44E-09 | 1.28E-07 | 11.83272 |
| SLC7A8 | -2.54096 | 0.257601 | -7.86673 | 1.45E-09 | 1.28E-07 | 11.82424 |
| OSR2 | 2.88532 | -0.41538 | 7.851984 | 1.52E-09 | 1.33E-07 | 11.77996 |
| TMEM80 | -1.48066 | 0.368376 | -7.84435 | 1.56E-09 | 1.35E-07 | 11.75701 |
| CMTM4 | -1.4666 | 0.166948 | -7.84243 | 1.56E-09 | 1.35E-07 | 11.75126 |
| FLJ45340 | -1.16279 | 0.51893 | -7.83998 | 1.58E-09 | 1.35E-07 | 11.74388 |
| ADRB2 | -2.44565 | 0.221776 | -7.83216 | 1.62E-09 | 1.38E-07 | 11.72039 |
| TTLL1 | -1.06931 | 0.075654 | -7.82188 | 1.67E-09 | 1.42E-07 | 11.68945 |
| CBLB | -1.14713 | 0.246501 | -7.81655 | 1.70E-09 | 1.43E-07 | 11.67343 |
| PER2 | -1.29797 | 0.216763 | -7.80146 | 1.78E-09 | 1.49E-07 | 11.62802 |
| DBP | -2.28789 | 0.943609 | -7.79457 | 1.81E-09 | 1.51E-07 | 11.6073 |
| NANOG | -2.48452 | 0.378154 | -7.79148 | 1.83E-09 | 1.51E-07 | 11.59798 |
| DGCR8 | -1.17156 | 0.317244 | -7.78363 | 1.88E-09 | 1.54E-07 | 11.57435 |
| FAM83E | -1.79427 | 0.40247 | -7.76341 | 2.00E-09 | 1.64E-07 | 11.51344 |
| RDH13 | -1.04164 | 0.262882 | -7.7468 | 2.10E-09 | 1.71E-07 | 11.46338 |
| LINC00346 | 1.497382 | -0.53075 | 7.743497 | 2.12E-09 | 1.72E-07 | 11.45341 |
| TNK1 | -1.14479 | 0.207973 | -7.73443 | 2.18E-09 | 1.77E-07 | 11.42607 |
| OLFML2B | 1.024885 | -0.17382 | 7.730444 | 2.21E-09 | 1.77E-07 | 11.41404 |
| DOPEY2 | -1.60503 | -0.01151 | -7.71998 | 2.28E-09 | 1.81E-07 | 11.38247 |
| TTTY20 | 1.666749 | -0.24216 | 7.71639 | 2.31E-09 | 1.82E-07 | 11.37164 |
| ATG9B | 1.098188 | -0.39174 | 7.695621 | 2.46E-09 | 1.92E-07 | 11.30893 |
| PIK3R3 | 1.170091 | -0.28062 | 7.686722 | 2.53E-09 | 1.97E-07 | 11.28205 |
| TPSD1 | 1.687574 | -0.43302 | 7.685473 | 2.54E-09 | 1.97E-07 | 11.27827 |
| VILL | -1.76385 | 0.115847 | -7.68232 | 2.57E-09 | 1.98E-07 | 11.26874 |
| IFITM1 | 1.72788 | 0.137388 | 7.679008 | 2.59E-09 | 1.99E-07 | 11.25874 |
| APOE | 2.009905 | 0.169155 | 7.67659 | 2.61E-09 | 2.00E-07 | 11.25143 |
| LOC100287525 | -1.21493 | 0.183861 | -7.6692 | 2.67E-09 | 2.02E-07 | 11.22911 |
| ALAD | -1.2822 | 0.159948 | -7.66465 | 2.71E-09 | 2.03E-07 | 11.21534 |
| GALNT12 | -1.38468 | 0.26292 | -7.65874 | 2.76E-09 | 2.05E-07 | 11.19748 |
| SLC2A10 | -1.58399 | 0.356804 | -7.65567 | 2.79E-09 | 2.06E-07 | 11.18818 |
| IL5 | 1.281349 | -0.22203 | 7.649044 | 2.85E-09 | 2.09E-07 | 11.16815 |
| DEPTOR | -1.62041 | 0.310328 | -7.64361 | 2.90E-09 | 2.12E-07 | 11.1517 |
| BCAS1 | -1.94339 | 0.242005 | -7.64114 | 2.92E-09 | 2.12E-07 | 11.14425 |
| FLJ23867 | -1.62585 | 0.200567 | -7.63678 | 2.96E-09 | 2.14E-07 | 11.13105 |
| ACSF2 | -1.68788 | 0.249098 | -7.6285 | 3.03E-09 | 2.18E-07 | 11.106 |
| KIAA0664L3 | -1.54832 | 0.666064 | -7.62667 | 3.05E-09 | 2.19E-07 | 11.10046 |
| FER1L4 | -2.72328 | 0.865412 | -7.61989 | 3.12E-09 | 2.21E-07 | 11.07994 |
| PPFIBP2 | -1.03454 | 0.1448 | -7.61976 | 3.12E-09 | 2.21E-07 | 11.07953 |
| TMEM38A | -1.32374 | 0.112199 | -7.61892 | 3.13E-09 | 2.21E-07 | 11.07698 |
| YWHAH | -1.40343 | 0.225665 | -7.61599 | 3.15E-09 | 2.23E-07 | 11.06813 |
| ITPKA | -1.75776 | 0.135061 | -7.61314 | 3.18E-09 | 2.24E-07 | 11.05949 |
| KIAA1530 | -1.26604 | 0.52024 | -7.58579 | 3.46E-09 | 2.41E-07 | 10.97663 |
| CYP4F12 | -1.74714 | 0.230656 | -7.5826 | 3.50E-09 | 2.41E-07 | 10.96697 |
| CD59 | -1.25449 | 0.167439 | -7.57495 | 3.58E-09 | 2.46E-07 | 10.94379 |
| CASR | -3.39233 | 1.168127 | -7.57255 | 3.61E-09 | 2.47E-07 | 10.93649 |
| TLE4 | -1.3156 | 0.088163 | -7.57199 | 3.62E-09 | 2.47E-07 | 10.9348 |
| C18orf34 | 2.257349 | -0.23659 | 7.561797 | 3.73E-09 | 2.54E-07 | 10.90389 |
| GDF7 | 1.154118 | -0.27207 | 7.557845 | 3.78E-09 | 2.55E-07 | 10.89191 |
| GATA4 | -1.61406 | 0.175533 | -7.55492 | 3.81E-09 | 2.55E-07 | 10.88302 |
| SLCO2A1 | -1.66563 | 0.494085 | -7.54786 | 3.90E-09 | 2.59E-07 | 10.8616 |
| GALE | -1.52548 | 0.208762 | -7.54098 | 3.98E-09 | 2.64E-07 | 10.84075 |
| IL36G | 1.517213 | -0.24525 | 7.530595 | 4.11E-09 | 2.72E-07 | 10.80921 |
| UAP1 | -1.2019 | -0.07091 | -7.52435 | 4.19E-09 | 2.76E-07 | 10.79026 |
| HYLS1 | -1.00731 | 0.08216 | -7.52209 | 4.22E-09 | 2.77E-07 | 10.78338 |
| MCU | -1.2456 | -0.16374 | -7.50951 | 4.39E-09 | 2.85E-07 | 10.74519 |
| TJP2 | -1.02624 | 0.117764 | -7.5048 | 4.46E-09 | 2.85E-07 | 10.73087 |
| GALC | 1.269954 | -0.02002 | 7.503696 | 4.47E-09 | 2.85E-07 | 10.72752 |
| GPR97 | 1.199893 | -0.27886 | 7.501834 | 4.50E-09 | 2.85E-07 | 10.72186 |
| PRSS16 | -1.16195 | 0.007681 | -7.49988 | 4.53E-09 | 2.85E-07 | 10.71592 |
| CYP2C19 | -1.63301 | 0.300063 | -7.49543 | 4.59E-09 | 2.87E-07 | 10.7024 |
| SNAI1 | 1.192094 | -0.10729 | 7.494987 | 4.60E-09 | 2.87E-07 | 10.70105 |
| MAP7D2 | -2.23721 | 0.241909 | -7.49176 | 4.64E-09 | 2.89E-07 | 10.69124 |
| PCDHGA2 | -1.09616 | 0.348663 | -7.47928 | 4.83E-09 | 2.98E-07 | 10.65331 |
| MST1R | -1.05244 | 0.107845 | -7.47788 | 4.85E-09 | 2.99E-07 | 10.64905 |
| KIAA0513 | -1.12358 | 0.123834 | -7.47455 | 4.90E-09 | 3.01E-07 | 10.63894 |
| PRICKLE1 | -1.66549 | 0.36145 | -7.46396 | 5.06E-09 | 3.08E-07 | 10.60673 |
| AACS | -1.1359 | 0.039291 | -7.46302 | 5.08E-09 | 3.08E-07 | 10.60386 |
| SCGB1D2 | -3.03844 | 1.055868 | -7.46106 | 5.11E-09 | 3.08E-07 | 10.59788 |
| B4GALT4 | -1.15306 | 0.085374 | -7.44059 | 5.45E-09 | 3.27E-07 | 10.53561 |
| PARD3B | -1.07916 | 0.206922 | -7.43341 | 5.57E-09 | 3.32E-07 | 10.51376 |
| USP34 | -1.16626 | 0.200047 | -7.43124 | 5.61E-09 | 3.33E-07 | 10.50713 |
| MEIS2 | -1.80901 | 0.209222 | -7.4304 | 5.62E-09 | 3.33E-07 | 10.5046 |
| CYP2S1 | -1.91975 | 0.02888 | -7.42761 | 5.67E-09 | 3.34E-07 | 10.4961 |
| IFITM4P | 1.312727 | -0.04159 | 7.424381 | 5.73E-09 | 3.36E-07 | 10.48626 |
| RPH3AL | -1.06333 | 0.093097 | -7.41949 | 5.82E-09 | 3.39E-07 | 10.47137 |
| PTPLAD1 | -1.18995 | 0.136232 | -7.4161 | 5.88E-09 | 3.41E-07 | 10.46104 |
| SYTL2 | -1.865 | 0.175265 | -7.39646 | 6.25E-09 | 3.62E-07 | 10.4012 |
| GNAL | 1.254422 | -0.13487 | 7.392906 | 6.32E-09 | 3.64E-07 | 10.39037 |
| ERVK13-1 | -1.41858 | 0.350164 | -7.39288 | 6.32E-09 | 3.64E-07 | 10.39028 |
| BCAR3 | -1.29816 | 0.122626 | -7.38404 | 6.50E-09 | 3.70E-07 | 10.36335 |
| PTK2B | -1.03124 | 0.158182 | -7.38375 | 6.50E-09 | 3.70E-07 | 10.36247 |
| STXBP2 | -1.27211 | 0.167822 | -7.38069 | 6.57E-09 | 3.71E-07 | 10.35313 |
| GNB5 | 1.002986 | -0.08585 | 7.371655 | 6.76E-09 | 3.79E-07 | 10.32557 |
| USP54 | -1.54203 | 0.161515 | -7.3699 | 6.79E-09 | 3.80E-07 | 10.32021 |
| MAL | -3.85146 | -0.24234 | -7.36188 | 6.97E-09 | 3.85E-07 | 10.29577 |
| PKDREJ | -2.26668 | 0.125136 | -7.35809 | 7.05E-09 | 3.87E-07 | 10.28419 |
| TET3 | -1.23882 | 0.274094 | -7.35365 | 7.15E-09 | 3.88E-07 | 10.27064 |
| TMEM206 | 1.17867 | -0.12806 | 7.350181 | 7.22E-09 | 3.90E-07 | 10.26006 |
| SH3TC1 | -1.37872 | 0.204119 | -7.34823 | 7.27E-09 | 3.90E-07 | 10.25412 |
| RGNEF | -1.84573 | 0.763528 | -7.34649 | 7.31E-09 | 3.91E-07 | 10.24879 |
| CCDC89 | -2.32422 | 0.977547 | -7.33764 | 7.51E-09 | 3.98E-07 | 10.22179 |
| CLIC3 | -2.15547 | 0.301531 | -7.33042 | 7.69E-09 | 4.05E-07 | 10.19973 |
| SUCNR1 | -3.7483 | 1.552613 | -7.32806 | 7.74E-09 | 4.07E-07 | 10.19255 |
| AK5 | -4.10514 | 1.835182 | -7.32733 | 7.76E-09 | 4.07E-07 | 10.1903 |
| ZSWIM6 | -1.00093 | 0.284097 | -7.32563 | 7.80E-09 | 4.08E-07 | 10.18511 |
| CSRP2BP | -1.25387 | 0.28283 | -7.32158 | 7.90E-09 | 4.12E-07 | 10.17276 |
| THBD | -2.01072 | 0.445454 | -7.32046 | 7.93E-09 | 4.12E-07 | 10.16934 |
| FOXN3 | -1.05662 | 0.217094 | -7.32005 | 7.94E-09 | 4.12E-07 | 10.16808 |
| LIMK2 | -1.01705 | 0.242569 | -7.31887 | 7.97E-09 | 4.12E-07 | 10.16448 |
| PLLP | -1.87852 | 0.196174 | -7.31726 | 8.01E-09 | 4.13E-07 | 10.15955 |
| SPATA5 | -1.74004 | 0.289226 | -7.31402 | 8.09E-09 | 4.16E-07 | 10.14965 |
| EIF2C4 | -1.07068 | 0.38489 | -7.30216 | 8.40E-09 | 4.30E-07 | 10.11343 |
| LEF1 | 1.54152 | -0.32471 | 7.289759 | 8.73E-09 | 4.43E-07 | 10.07553 |
| FAM174B | -1.25595 | 0.328666 | -7.27951 | 9.02E-09 | 4.53E-07 | 10.04419 |
| PSD3 | -1.7854 | 0.291045 | -7.26842 | 9.33E-09 | 4.67E-07 | 10.0103 |
| ST6GALNAC2 | -1.20459 | 0.210441 | -7.26656 | 9.39E-09 | 4.67E-07 | 10.00459 |
| CLTB | -1.16599 | 0.092381 | -7.26439 | 9.45E-09 | 4.69E-07 | 9.997958 |
| MUC5AC | -2.79834 | 0.210682 | -7.24565 | 1.00E-08 | 4.92E-07 | 9.940641 |
| KCTD14 | -1.5811 | 0.035269 | -7.24259 | 1.01E-08 | 4.95E-07 | 9.931278 |
| HOXA10 | 3.673292 | -1.63569 | 7.242205 | 1.01E-08 | 4.95E-07 | 9.930084 |
| ARNTL2 | -1.22447 | 0.346771 | -7.23688 | 1.03E-08 | 5.00E-07 | 9.913799 |
| SH3RF1 | -1.17292 | 0.144776 | -7.23676 | 1.03E-08 | 5.00E-07 | 9.913406 |
| KLF2 | -1.37466 | 0.160769 | -7.21429 | 1.11E-08 | 5.34E-07 | 9.844632 |
| SLC3A2 | -1.04341 | 0.142668 | -7.21352 | 1.11E-08 | 5.34E-07 | 9.842273 |
| CCL3L3 | 2.106959 | -0.20074 | 7.211529 | 1.12E-08 | 5.36E-07 | 9.836167 |
| CTSE | -1.68586 | 0.038582 | -7.20239 | 1.15E-08 | 5.49E-07 | 9.80816 |
| C9orf125 | -1.09439 | 0.213298 | -7.19041 | 1.19E-08 | 5.60E-07 | 9.771476 |
| LOC643650 | -1.43127 | 0.550215 | -7.18928 | 1.20E-08 | 5.60E-07 | 9.768005 |
| FAM156A | -1.07495 | 0.27947 | -7.17631 | 1.25E-08 | 5.81E-07 | 9.72824 |
| PBX4 | -1.24605 | 0.476727 | -7.16842 | 1.28E-08 | 5.92E-07 | 9.704057 |
| C1orf51 | -2.91591 | 0.820764 | -7.16684 | 1.28E-08 | 5.94E-07 | 9.699231 |
| PLCXD1 | -1.86928 | 0.173225 | -7.16541 | 1.29E-08 | 5.95E-07 | 9.694823 |
| FCER1G | 1.187478 | -0.20536 | 7.158363 | 1.32E-08 | 6.07E-07 | 9.673227 |
| LGALS9C | -1.53904 | 0.258735 | -7.14929 | 1.36E-08 | 6.21E-07 | 9.645412 |
| MLPH | -1.39959 | 0.216802 | -7.14468 | 1.38E-08 | 6.28E-07 | 9.63125 |
| EMR2 | 2.249634 | -0.34565 | 7.144325 | 1.38E-08 | 6.28E-07 | 9.630169 |
| TMEM131 | -1.11332 | 0.413497 | -7.13342 | 1.43E-08 | 6.45E-07 | 9.59672 |
| KAZN | -1.11947 | 0.242409 | -7.11781 | 1.50E-08 | 6.69E-07 | 9.548802 |
| LINC00479 | -2.49101 | 0.317204 | -7.11458 | 1.51E-08 | 6.72E-07 | 9.538874 |
| OTX1 | 1.909223 | -0.73561 | 7.096015 | 1.60E-08 | 7.08E-07 | 9.481878 |
| ALDH3A1 | -2.43164 | 0.052827 | -7.09463 | 1.61E-08 | 7.08E-07 | 9.477639 |
| CAPN9 | -2.98707 | 0.3356 | -7.08929 | 1.64E-08 | 7.16E-07 | 9.46123 |
| GAST | -7.26847 | 0.210154 | -7.08815 | 1.64E-08 | 7.17E-07 | 9.457708 |
| ENTPD5 | -1.57308 | 0.17748 | -7.08237 | 1.68E-08 | 7.23E-07 | 9.439951 |
| TMCO3 | -1.11981 | 0.025225 | -7.08126 | 1.68E-08 | 7.24E-07 | 9.436546 |
| NR2F6 | -1.11656 | 0.197135 | -7.08075 | 1.68E-08 | 7.24E-07 | 9.434998 |
| MARCO | 1.871765 | -0.15749 | 7.078092 | 1.70E-08 | 7.26E-07 | 9.42682 |
| C1orf116 | -1.22077 | 0.063981 | -7.06856 | 1.75E-08 | 7.47E-07 | 9.39753 |
| NAPEPLD | -1.23067 | 0.071544 | -7.06528 | 1.77E-08 | 7.49E-07 | 9.38744 |
| MICALL1 | -1.57413 | 0.313362 | -7.06435 | 1.77E-08 | 7.50E-07 | 9.384598 |
| IL18BP | 1.000504 | -0.05185 | 7.062986 | 1.78E-08 | 7.50E-07 | 9.380396 |
| SLC16A14 | 1.514804 | -0.03243 | 7.062617 | 1.78E-08 | 7.50E-07 | 9.379262 |
| KIAA0319 | -2.77879 | 1.352989 | -7.05857 | 1.81E-08 | 7.58E-07 | 9.36682 |
| ATP2A3 | -1.52661 | 0.047552 | -7.05162 | 1.85E-08 | 7.73E-07 | 9.345447 |
| ADCK4 | -1.001 | 0.281942 | -7.04785 | 1.87E-08 | 7.79E-07 | 9.33388 |
| SAMHD1 | 1.177421 | -0.32504 | 7.045662 | 1.88E-08 | 7.80E-07 | 9.327138 |
| TBC1D3F | -1.30454 | 0.370602 | -7.04257 | 1.90E-08 | 7.83E-07 | 9.317624 |
| TSKU | -1.5098 | 0.121555 | -7.04253 | 1.90E-08 | 7.83E-07 | 9.317498 |
| FBLIM1 | -1.2812 | 0.090499 | -7.036 | 1.94E-08 | 7.98E-07 | 9.29744 |
| STBD1 | -1.71893 | 0.502643 | -7.03332 | 1.96E-08 | 8.03E-07 | 9.289188 |
| EGR2 | 2.104096 | -0.24408 | 7.030504 | 1.97E-08 | 8.06E-07 | 9.280523 |
| IL8 | 2.336044 | 0.45232 | 7.029008 | 1.98E-08 | 8.08E-07 | 9.275922 |
| TRIM16L | -1.23022 | 0.096875 | -7.0267 | 2.00E-08 | 8.12E-07 | 9.268835 |
| KAZALD1 | -1.31487 | 0.158438 | -7.02222 | 2.02E-08 | 8.17E-07 | 9.255037 |
| SPATA20 | -1.09464 | 0.254204 | -7.0222 | 2.03E-08 | 8.17E-07 | 9.254972 |
| RASEF | -1.58828 | 0.40386 | -7.02172 | 2.03E-08 | 8.17E-07 | 9.2535 |
| SH3RF2 | -1.03844 | -0.06049 | -7.0217 | 2.03E-08 | 8.17E-07 | 9.253451 |
| HOXA7 | 2.188983 | -0.05062 | 7.020897 | 2.03E-08 | 8.17E-07 | 9.250969 |
| LOC100505592 | 2.317093 | -0.22836 | 7.020621 | 2.04E-08 | 8.17E-07 | 9.250121 |
| EFNB2 | -1.26712 | 0.055466 | -7.02016 | 2.04E-08 | 8.17E-07 | 9.248692 |
| DYRK2 | -1.02684 | 0.19452 | -7.00589 | 2.13E-08 | 8.43E-07 | 9.204796 |
| KLF4 | -1.43905 | -0.06883 | -7.00423 | 2.14E-08 | 8.46E-07 | 9.199695 |
| PIGS | -1.31043 | 0.181174 | -6.99266 | 2.22E-08 | 8.73E-07 | 9.164087 |
| PDE4B | 1.361202 | 0.297706 | 6.991317 | 2.23E-08 | 8.75E-07 | 9.159939 |
| CRIP1 | -1.81641 | -0.1796 | -6.99047 | 2.24E-08 | 8.76E-07 | 9.157336 |
| ANKRD50 | 1.047196 | -0.42495 | 6.989283 | 2.25E-08 | 8.76E-07 | 9.153678 |
| CRYGD | -3.23054 | 1.375773 | -6.98858 | 2.25E-08 | 8.76E-07 | 9.15151 |
| CCL3 | 2.47042 | -0.00811 | 6.985475 | 2.27E-08 | 8.82E-07 | 9.141954 |
| HPCAL1 | -1.31635 | 0.224206 | -6.98327 | 2.29E-08 | 8.85E-07 | 9.135177 |
| C17orf110 | -1.81783 | 0.092871 | -6.97734 | 2.33E-08 | 8.98E-07 | 9.116898 |
| C8orf47 | -1.44939 | 0.173146 | -6.97185 | 2.37E-08 | 9.07E-07 | 9.100013 |
| UBL3 | -1.3264 | 0.1542 | -6.96907 | 2.39E-08 | 9.07E-07 | 9.091427 |
| GATA6 | -1.42945 | 0.400985 | -6.96325 | 2.44E-08 | 9.21E-07 | 9.073514 |
| C4orf34 | -1.40865 | 0.195651 | -6.96147 | 2.45E-08 | 9.24E-07 | 9.068032 |
| TTC7A | -1.2448 | 0.233666 | -6.94924 | 2.55E-08 | 9.51E-07 | 9.030365 |
| C9orf7 | -1.18969 | 0.1396 | -6.94924 | 2.55E-08 | 9.51E-07 | 9.030365 |
| RNASE2 | 2.039249 | -0.14175 | 6.93373 | 2.68E-08 | 9.87E-07 | 8.982555 |
| ASPHD2 | -1.56455 | 0.011895 | -6.92247 | 2.77E-08 | 1.02E-06 | 8.947853 |
| TFCP2L1 | -1.97624 | 0.398114 | -6.91904 | 2.80E-08 | 1.02E-06 | 8.937285 |
| TLCD2 | -1.46398 | 0.207448 | -6.91658 | 2.83E-08 | 1.03E-06 | 8.929692 |
| CIRBP | -1.22952 | 0.476792 | -6.91516 | 2.84E-08 | 1.03E-06 | 8.925318 |
| IL6 | 1.828013 | -0.06934 | 6.913366 | 2.86E-08 | 1.03E-06 | 8.919777 |
| SNX33 | -1.16219 | 0.077601 | -6.91251 | 2.86E-08 | 1.03E-06 | 8.917135 |
| STON2 | -1.56137 | 0.265279 | -6.91119 | 2.88E-08 | 1.04E-06 | 8.913067 |
| RPTN | -2.24802 | 1.179295 | -6.91042 | 2.88E-08 | 1.04E-06 | 8.910697 |
| NQO1 | -1.55543 | -0.03757 | -6.90879 | 2.90E-08 | 1.04E-06 | 8.905669 |
| GPNMB | 1.061842 | 0.121654 | 6.900707 | 2.97E-08 | 1.05E-06 | 8.88074 |
| C14orf132 | -1.74469 | 0.727794 | -6.90048 | 2.97E-08 | 1.05E-06 | 8.880031 |
| ABCC3 | -1.25664 | 0.076063 | -6.89618 | 3.02E-08 | 1.06E-06 | 8.86678 |
| FHAD1 | -2.07031 | 0.325063 | -6.89566 | 3.02E-08 | 1.06E-06 | 8.865179 |
| SYT4 | -3.71071 | 1.36945 | -6.88712 | 3.10E-08 | 1.09E-06 | 8.838843 |
| DNMBP | -1.15088 | -0.00244 | -6.88309 | 3.14E-08 | 1.10E-06 | 8.826404 |
| KCNJ6 | -2.58852 | 0.902147 | -6.88239 | 3.15E-08 | 1.10E-06 | 8.824245 |
| ATXN7L3B | -1.18708 | 0.152644 | -6.87569 | 3.22E-08 | 1.11E-06 | 8.803568 |
| PAN2 | -1.10313 | 0.294238 | -6.87376 | 3.24E-08 | 1.11E-06 | 8.797604 |
| OPHN1 | -1.30784 | 0.024785 | -6.87347 | 3.24E-08 | 1.11E-06 | 8.79671 |
| GABRG2 | -1.54604 | 0.594424 | -6.86328 | 3.35E-08 | 1.14E-06 | 8.765257 |
| SMPD3 | -1.58358 | -0.07396 | -6.86188 | 3.36E-08 | 1.14E-06 | 8.760939 |
| SENP7 | -1.23511 | 0.388449 | -6.86087 | 3.37E-08 | 1.14E-06 | 8.757838 |
| KLC4 | -1.02356 | 0.025926 | -6.85927 | 3.39E-08 | 1.15E-06 | 8.752883 |
| CXCR4 | 1.664354 | 0.246976 | 6.856638 | 3.42E-08 | 1.15E-06 | 8.744766 |
| CD300A | 1.559242 | -0.0283 | 6.853089 | 3.46E-08 | 1.16E-06 | 8.733811 |
| LINC00086 | -2.1152 | 0.348848 | -6.85091 | 3.48E-08 | 1.17E-06 | 8.727089 |
| ANKRD29 | -1.54337 | 0.227267 | -6.84588 | 3.54E-08 | 1.18E-06 | 8.71156 |
| FAM20A | -2.52152 | 0.488835 | -6.84525 | 3.54E-08 | 1.18E-06 | 8.709614 |
| CD300LF | 1.731967 | 0.07701 | 6.840173 | 3.60E-08 | 1.20E-06 | 8.693935 |
| FRY | -1.45881 | 0.539934 | -6.83915 | 3.61E-08 | 1.20E-06 | 8.690788 |
| PDPN | 2.008492 | -0.2949 | 6.837448 | 3.63E-08 | 1.21E-06 | 8.685523 |
| LOC653075 | -1.62744 | 0.581644 | -6.83211 | 3.69E-08 | 1.23E-06 | 8.669051 |
| SPATA6 | -1.50984 | 0.354106 | -6.8313 | 3.70E-08 | 1.23E-06 | 8.666531 |
| ARHGEF4 | -2.94711 | 0.866252 | -6.82111 | 3.82E-08 | 1.26E-06 | 8.635075 |
| HOXB13 | 3.544944 | -1.57386 | 6.803407 | 4.04E-08 | 1.32E-06 | 8.580377 |
| ECHDC2 | -1.09131 | 0.177091 | -6.80319 | 4.05E-08 | 1.32E-06 | 8.579709 |
| MYO18A | -1.01835 | 0.190688 | -6.79479 | 4.16E-08 | 1.35E-06 | 8.553746 |
| SLC5A5 | -2.27718 | -0.22018 | -6.7899 | 4.22E-08 | 1.37E-06 | 8.53863 |
| KLHL20 | -1.14311 | 0.176356 | -6.78831 | 4.24E-08 | 1.37E-06 | 8.533725 |
| GALNT6 | -1.77251 | 0.217033 | -6.78696 | 4.26E-08 | 1.37E-06 | 8.529538 |
| C12orf42 | 1.230241 | -0.01768 | 6.786712 | 4.26E-08 | 1.37E-06 | 8.528787 |
| TNFRSF10C | 1.585602 | -0.50968 | 6.78494 | 4.29E-08 | 1.38E-06 | 8.523309 |
| NR1D2 | -1.73897 | 0.499948 | -6.78018 | 4.35E-08 | 1.38E-06 | 8.508583 |
| SDS | 1.178963 | -0.27889 | 6.774528 | 4.43E-08 | 1.40E-06 | 8.491125 |
| PRRT2 | -1.75823 | 0.866872 | -6.77451 | 4.43E-08 | 1.40E-06 | 8.491072 |
| TBL1X | -1.06272 | 0.124468 | -6.77018 | 4.49E-08 | 1.42E-06 | 8.477684 |
| MRO | -2.17097 | 0.854476 | -6.76338 | 4.59E-08 | 1.45E-06 | 8.456655 |
| FAM110C | -1.20512 | 0.124473 | -6.76083 | 4.63E-08 | 1.45E-06 | 8.448773 |
| ODF3L1 | -2.16203 | -0.16361 | -6.75928 | 4.65E-08 | 1.45E-06 | 8.44399 |
| C21orf2 | -1.41825 | 0.413436 | -6.74479 | 4.87E-08 | 1.51E-06 | 8.399173 |
| GRIP2 | -1.59132 | 0.347529 | -6.74377 | 4.89E-08 | 1.52E-06 | 8.396015 |
| MAPK3 | -1.56699 | 0.061176 | -6.74151 | 4.92E-08 | 1.52E-06 | 8.389023 |
| EVPLL | -2.5051 | 0.863429 | -6.7385 | 4.97E-08 | 1.53E-06 | 8.379718 |
| NRG4 | -1.91908 | 0.216995 | -6.72924 | 5.12E-08 | 1.56E-06 | 8.351071 |
| KLB | -1.89341 | 0.214999 | -6.72384 | 5.20E-08 | 1.58E-06 | 8.334374 |
| LOC100505633 | -2.06205 | -0.03309 | -6.72063 | 5.26E-08 | 1.59E-06 | 8.324448 |
| SLC18A2 | 1.02666 | -0.0707 | 6.715736 | 5.34E-08 | 1.61E-06 | 8.309296 |
| LOC100505648 | -1.60579 | 0.509509 | -6.70981 | 5.44E-08 | 1.64E-06 | 8.290971 |
| SEMA3B | -1.2134 | 0.265092 | -6.70718 | 5.49E-08 | 1.65E-06 | 8.282816 |
| PIP4K2C | -1.35624 | 0.146711 | -6.70325 | 5.56E-08 | 1.66E-06 | 8.270643 |
| SIDT2 | -1.53801 | 0.228386 | -6.69518 | 5.70E-08 | 1.69E-06 | 8.245667 |
| EFHC1 | -1.13488 | 0.286357 | -6.69498 | 5.70E-08 | 1.69E-06 | 8.245061 |
| HOXA13 | 5.232488 | -2.06107 | 6.692439 | 5.75E-08 | 1.69E-06 | 8.237195 |
| GPRC5B | -1.66843 | -0.02724 | -6.69242 | 5.75E-08 | 1.69E-06 | 8.237147 |
| MCF2L | -1.12684 | 0.194687 | -6.68716 | 5.85E-08 | 1.71E-06 | 8.220854 |
| CCR1 | 1.262646 | 0.067185 | 6.68454 | 5.90E-08 | 1.72E-06 | 8.212743 |
| TMEM27 | -2.80317 | 0.98232 | -6.683 | 5.93E-08 | 1.72E-06 | 8.207981 |
| C1orf63 | -1.17273 | 0.328145 | -6.67521 | 6.07E-08 | 1.76E-06 | 8.183857 |
| GPD1L | -1.21327 | 0.00496 | -6.66055 | 6.36E-08 | 1.83E-06 | 8.138459 |
| SST | -3.4858 | 0.830199 | -6.65675 | 6.44E-08 | 1.85E-06 | 8.126708 |
| KLHDC1 | -1.02306 | 0.427271 | -6.65467 | 6.48E-08 | 1.86E-06 | 8.120251 |
| RAB27A | -1.75172 | 0.283349 | -6.65185 | 6.54E-08 | 1.87E-06 | 8.111518 |
| ATG16L1 | -1.09593 | 0.302667 | -6.65178 | 6.54E-08 | 1.87E-06 | 8.111294 |
| MPZL3 | -1.02226 | 0.172502 | -6.64534 | 6.68E-08 | 1.91E-06 | 8.091341 |
| FOSB | 3.75741 | -0.33705 | 6.643391 | 6.72E-08 | 1.91E-06 | 8.085317 |
| C19orf59 | 2.505403 | -0.36132 | 6.64138 | 6.76E-08 | 1.92E-06 | 8.079089 |
| TAX1BP3 | -1.23185 | 0.140358 | -6.63931 | 6.81E-08 | 1.93E-06 | 8.072689 |
| SSTR1 | -1.89292 | -0.24017 | -6.63292 | 6.95E-08 | 1.95E-06 | 8.05289 |
| MYZAP | -1.11469 | -0.00796 | -6.63181 | 6.97E-08 | 1.95E-06 | 8.049431 |
| C2 | 1.824892 | -0.3132 | 6.629135 | 7.03E-08 | 1.96E-06 | 8.041155 |
| ETNK1 | -1.55289 | 0.081368 | -6.62788 | 7.06E-08 | 1.97E-06 | 8.03726 |
| HOXA6 | 1.080109 | -0.11344 | 6.625797 | 7.11E-08 | 1.98E-06 | 8.030813 |
| CTSZ | 1.329724 | -0.49904 | 6.611892 | 7.43E-08 | 2.05E-06 | 7.987724 |
| PLA2G7 | 2.697015 | -0.49504 | 6.610319 | 7.46E-08 | 2.05E-06 | 7.982852 |
| ARL11 | 1.498765 | 0.243477 | 6.604044 | 7.61E-08 | 2.09E-06 | 7.963404 |
| GNA14 | 1.659817 | -0.28871 | 6.598608 | 7.75E-08 | 2.11E-06 | 7.946555 |
| SH3GLB2 | -1.0791 | 0.287261 | -6.59726 | 7.78E-08 | 2.12E-06 | 7.942365 |
| LOC100507520 | -2.31356 | 0.616648 | -6.59331 | 7.88E-08 | 2.14E-06 | 7.930139 |
| MSR1 | 1.761835 | -0.03261 | 6.591879 | 7.91E-08 | 2.14E-06 | 7.925697 |
| FCGR1B | 2.8108 | -0.27245 | 6.578216 | 8.27E-08 | 2.23E-06 | 7.883342 |
| DKFZP586I1420 | -1.06067 | 0.174101 | -6.57251 | 8.42E-08 | 2.27E-06 | 7.865641 |
| SOSTDC1 | -2.79615 | -0.00232 | -6.57061 | 8.47E-08 | 2.28E-06 | 7.859767 |
| MIRLET7BHG | -2.30365 | 0.839489 | -6.56533 | 8.61E-08 | 2.31E-06 | 7.843388 |
| SMAD7 | -1.15231 | 0.222348 | -6.56041 | 8.75E-08 | 2.34E-06 | 7.82814 |
| H1F0 | -1.48198 | 0.245018 | -6.55766 | 8.82E-08 | 2.35E-06 | 7.819596 |
| CXCL1 | 3.408123 | -0.7369 | 6.555043 | 8.90E-08 | 2.36E-06 | 7.811486 |
| C11orf9 | -1.06688 | 0.225156 | -6.5491 | 9.07E-08 | 2.39E-06 | 7.79307 |
| PTPRN2 | -1.23549 | 0.134582 | -6.54842 | 9.09E-08 | 2.40E-06 | 7.790959 |
| NR4A3 | 1.027223 | 0.032566 | 6.547552 | 9.11E-08 | 2.40E-06 | 7.788255 |
| AGAP8 | -1.07779 | 0.291593 | -6.54421 | 9.21E-08 | 2.42E-06 | 7.777873 |
| ARID3B | -1.02704 | 0.205658 | -6.53888 | 9.37E-08 | 2.44E-06 | 7.761354 |
| UNC5B | -1.53417 | 0.036539 | -6.53714 | 9.42E-08 | 2.45E-06 | 7.755961 |
| DPCR1 | -3.17835 | 0.054986 | -6.53651 | 9.44E-08 | 2.45E-06 | 7.753996 |
| ZDHHC11 | -2.55683 | 0.259685 | -6.53608 | 9.45E-08 | 2.45E-06 | 7.752668 |
| OSM | 3.023667 | 0.730805 | 6.525005 | 9.79E-08 | 2.54E-06 | 7.718313 |
| LOC100129917 | -1.30899 | 0.279526 | -6.51767 | 1.00E-07 | 2.58E-06 | 7.69557 |
| PSAPL1 | -3.99389 | -0.12373 | -6.50976 | 1.03E-07 | 2.63E-06 | 7.671006 |
| APOC2 | 2.631169 | -0.34493 | 6.506347 | 1.04E-07 | 2.65E-06 | 7.660425 |
| HIPK2 | -1.07507 | 0.198428 | -6.49985 | 1.06E-07 | 2.69E-06 | 7.640262 |
| SPOCK2 | 1.149479 | -0.05706 | 6.498905 | 1.06E-07 | 2.69E-06 | 7.63733 |
| C5orf25 | -1.24867 | 0.376444 | -6.4915 | 1.09E-07 | 2.75E-06 | 7.614338 |
| TMEM92 | -1.36406 | 0.308245 | -6.48211 | 1.12E-07 | 2.83E-06 | 7.585211 |
| MSX1 | 1.710863 | -1.21813 | 6.481554 | 1.12E-07 | 2.83E-06 | 7.583482 |
| UNC5CL | -1.4818 | 0.351705 | -6.47857 | 1.14E-07 | 2.85E-06 | 7.574234 |
| IL1R2 | -1.65282 | 0.149811 | -6.47763 | 1.14E-07 | 2.85E-06 | 7.571303 |
| GPR27 | -1.63561 | 0.265779 | -6.47685 | 1.14E-07 | 2.85E-06 | 7.568889 |
| MMEL1 | -1.57687 | 0.087502 | -6.47595 | 1.14E-07 | 2.85E-06 | 7.566081 |
| HAVCR2 | 1.65556 | -0.02404 | 6.473997 | 1.15E-07 | 2.87E-06 | 7.560024 |
| LOC100505702 | 1.998359 | -0.06821 | 6.470324 | 1.17E-07 | 2.88E-06 | 7.548621 |
| CD209 | 1.109338 | -0.14758 | 6.468494 | 1.17E-07 | 2.89E-06 | 7.542941 |
| LRP1 | -1.15687 | 0.189188 | -6.4683 | 1.17E-07 | 2.89E-06 | 7.542326 |
| LOC643201 | -2.1814 | 0.523123 | -6.4677 | 1.18E-07 | 2.89E-06 | 7.540479 |
| ZNF500 | -1.02853 | 0.26309 | -6.4672 | 1.18E-07 | 2.89E-06 | 7.53894 |
| HCG27 | -1.87041 | 0.536945 | -6.46303 | 1.19E-07 | 2.92E-06 | 7.525978 |
| C15orf27 | -2.61029 | 0.612478 | -6.46227 | 1.20E-07 | 2.93E-06 | 7.523616 |
| CCNL2 | -1.0723 | 0.28148 | -6.4587 | 1.21E-07 | 2.95E-06 | 7.512552 |
| HOXB7 | 1.306268 | -0.21161 | 6.448835 | 1.25E-07 | 3.02E-06 | 7.48191 |
| SEC14L5 | -2.63136 | 0.596085 | -6.44772 | 1.25E-07 | 3.03E-06 | 7.47845 |
| KDM4C | -1.25103 | 0.271284 | -6.44363 | 1.27E-07 | 3.06E-06 | 7.465735 |
| MID1IP1 | -1.26918 | -0.05475 | -6.44184 | 1.28E-07 | 3.07E-06 | 7.460205 |
| KLK6 | 2.65582 | -0.10761 | 6.438451 | 1.29E-07 | 3.10E-06 | 7.449666 |
| NOXA1 | -1.06499 | 0.175542 | -6.42976 | 1.33E-07 | 3.17E-06 | 7.422685 |
| GPR64 | -1.67753 | 0.430065 | -6.42524 | 1.35E-07 | 3.20E-06 | 7.408637 |
| FA2H | -1.31796 | -0.11589 | -6.4211 | 1.36E-07 | 3.24E-06 | 7.395799 |
| FJX1 | 1.429085 | -0.19863 | 6.420111 | 1.37E-07 | 3.25E-06 | 7.392713 |
| KHDC1 | -1.28889 | 0.155687 | -6.41839 | 1.37E-07 | 3.25E-06 | 7.38737 |
| LRRC56 | -1.02192 | 0.354749 | -6.41554 | 1.39E-07 | 3.27E-06 | 7.378516 |
| ABHD6 | -1.05388 | 0.160046 | -6.41425 | 1.39E-07 | 3.27E-06 | 7.374521 |
| C16orf79 | -1.14646 | 0.301037 | -6.41412 | 1.39E-07 | 3.27E-06 | 7.374099 |
| CCL4 | 1.718495 | 0.089134 | 6.413147 | 1.40E-07 | 3.28E-06 | 7.371084 |
| DAPK2 | -1.50889 | 0.033332 | -6.41289 | 1.40E-07 | 3.28E-06 | 7.370273 |
| PPP1R3E | -1.51356 | 0.503662 | -6.40789 | 1.42E-07 | 3.32E-06 | 7.354748 |
| CWH43 | -4.37494 | -0.63762 | -6.40628 | 1.43E-07 | 3.33E-06 | 7.349748 |
| ZNF285 | -1.98951 | 0.153332 | -6.40579 | 1.43E-07 | 3.33E-06 | 7.348219 |
| F2RL2 | 1.288012 | -0.12155 | 6.400686 | 1.45E-07 | 3.38E-06 | 7.332378 |
| BAIAP2 | -1.42595 | 0.184239 | -6.39973 | 1.46E-07 | 3.39E-06 | 7.329413 |
| HES2 | 1.505569 | -0.57712 | 6.398281 | 1.47E-07 | 3.40E-06 | 7.324909 |
| SRGAP1 | -1.207 | 0.223801 | -6.39261 | 1.49E-07 | 3.45E-06 | 7.307288 |
| UROS | 1.272465 | -0.27132 | 6.388971 | 1.51E-07 | 3.48E-06 | 7.295986 |
| TMPRSS6 | -1.67749 | 0.376548 | -6.38273 | 1.54E-07 | 3.52E-06 | 7.27659 |
| VSIG2 | -1.70153 | 0.085075 | -6.38177 | 1.55E-07 | 3.52E-06 | 7.273602 |
| CCR7 | 2.131882 | 0.354684 | 6.38013 | 1.55E-07 | 3.53E-06 | 7.26852 |
| SGK494 | -1.29869 | 0.34212 | -6.3668 | 1.62E-07 | 3.67E-06 | 7.227093 |
| FZD2 | 1.212631 | -0.03361 | 6.366157 | 1.62E-07 | 3.67E-06 | 7.225107 |
| RAB7B | -1.2395 | 0.154305 | -6.36222 | 1.64E-07 | 3.71E-06 | 7.212866 |
| KCNK17 | -2.30903 | 0.519625 | -6.35954 | 1.66E-07 | 3.73E-06 | 7.204549 |
| TEP1 | -1.05007 | 0.359985 | -6.35482 | 1.68E-07 | 3.79E-06 | 7.189878 |
| C5orf39 | 1.157809 | -0.03119 | 6.353149 | 1.69E-07 | 3.80E-06 | 7.184688 |
| MPP3 | -1.45523 | 0.286783 | -6.3524 | 1.70E-07 | 3.80E-06 | 7.182346 |
| CSTA | -2.27087 | 0.085982 | -6.34842 | 1.72E-07 | 3.84E-06 | 7.169986 |
| LOC100128822 | -1.07953 | 0.195141 | -6.3481 | 1.72E-07 | 3.84E-06 | 7.168987 |
| PLXNB2 | -1.16445 | 0.08552 | -6.34695 | 1.73E-07 | 3.85E-06 | 7.165418 |
| ATP9B | -1.27517 | 0.099863 | -6.34049 | 1.76E-07 | 3.91E-06 | 7.145337 |
| IL6R | -1.46005 | 0.532222 | -6.33966 | 1.77E-07 | 3.91E-06 | 7.142755 |
| ARHGAP29 | -1.41914 | 0.48786 | -6.33726 | 1.78E-07 | 3.92E-06 | 7.135309 |
| HSPB1 | -1.28708 | 0.241298 | -6.33706 | 1.78E-07 | 3.92E-06 | 7.134681 |
| EPS8L1 | -1.35328 | 0.309936 | -6.33681 | 1.78E-07 | 3.92E-06 | 7.133898 |
| ESYT3 | -1.69711 | 0.590219 | -6.33209 | 1.81E-07 | 3.97E-06 | 7.119246 |
| LOC729234 | -1.7173 | 0.61366 | -6.33098 | 1.82E-07 | 3.98E-06 | 7.115788 |
| TBC1D25 | -1.15111 | 0.275857 | -6.32237 | 1.87E-07 | 4.07E-06 | 7.089043 |
| EPN1 | -1.07023 | 0.039391 | -6.30594 | 1.97E-07 | 4.24E-06 | 7.037974 |
| PPP2R3A | -1.49884 | 0.373585 | -6.29825 | 2.02E-07 | 4.32E-06 | 7.01407 |
| LINC00087 | -1.82915 | 0.153155 | -6.28743 | 2.09E-07 | 4.43E-06 | 6.980433 |
| LOC100132363 | -2.11439 | 1.00249 | -6.2871 | 2.09E-07 | 4.43E-06 | 6.979407 |
| FLCN | -1.1653 | 0.248148 | -6.28585 | 2.10E-07 | 4.44E-06 | 6.975526 |
| FAM198B | 1.084907 | -0.10686 | 6.280197 | 2.14E-07 | 4.50E-06 | 6.957937 |
| HGF | 1.139366 | -0.01216 | 6.277971 | 2.15E-07 | 4.53E-06 | 6.951017 |
| KLHDC7B | 1.156165 | 0.131006 | 6.27558 | 2.17E-07 | 4.56E-06 | 6.943583 |
| TESC | -1.47581 | 0.077648 | -6.27458 | 2.17E-07 | 4.56E-06 | 6.940479 |
| RIT2 | 1.077122 | 0.101757 | 6.272678 | 2.19E-07 | 4.58E-06 | 6.934559 |
| MAGI3 | -1.39253 | 0.524355 | -6.27009 | 2.21E-07 | 4.61E-06 | 6.92652 |
| SEMA3F | -1.39838 | -0.02041 | -6.26044 | 2.28E-07 | 4.72E-06 | 6.896523 |
| KIAA1683 | -1.75158 | 0.603939 | -6.25961 | 2.28E-07 | 4.73E-06 | 6.893926 |
| LRRC25 | 1.595661 | -0.14775 | 6.257748 | 2.29E-07 | 4.74E-06 | 6.888141 |
| IGSF3 | -1.10848 | 0.240585 | -6.25752 | 2.30E-07 | 4.74E-06 | 6.887426 |
| RXFP4 | 1.80709 | -0.51216 | 6.257429 | 2.30E-07 | 4.74E-06 | 6.887149 |
| IGSF6 | 1.842709 | -0.09271 | 6.257075 | 2.30E-07 | 4.74E-06 | 6.886046 |
| SOX21 | -3.63271 | -0.24672 | -6.25413 | 2.32E-07 | 4.78E-06 | 6.876895 |
| MGC4294 | 1.165673 | -0.04426 | 6.249316 | 2.36E-07 | 4.84E-06 | 6.861923 |
| FCGR2A | 1.451502 | -0.04226 | 6.248757 | 2.36E-07 | 4.84E-06 | 6.860183 |
| HSD3B1 | 1.302189 | -0.09651 | 6.245886 | 2.38E-07 | 4.88E-06 | 6.851258 |
| CARTPT | -3.53962 | 1.264548 | -6.24446 | 2.39E-07 | 4.89E-06 | 6.846832 |
| NCKAP5 | -2.02011 | -0.21856 | -6.2431 | 2.40E-07 | 4.90E-06 | 6.842591 |
| RAPGEFL1 | -1.72608 | 0.017996 | -6.24155 | 2.42E-07 | 4.91E-06 | 6.837766 |
| ITGB8 | 1.319364 | -0.15624 | 6.240501 | 2.42E-07 | 4.91E-06 | 6.834511 |
| MGC50722 | -1.45083 | 0.288208 | -6.24038 | 2.43E-07 | 4.91E-06 | 6.834123 |
| UGCG | -1.03012 | 0.06211 | -6.23092 | 2.50E-07 | 5.06E-06 | 6.804731 |
| LILRA4 | 1.427991 | -0.38512 | 6.228086 | 2.52E-07 | 5.09E-06 | 6.795905 |
| ANKRD18B | -1.975 | 0.205747 | -6.22765 | 2.53E-07 | 5.09E-06 | 6.79456 |
| PPM1J | 1.352903 | -0.24194 | 6.226895 | 2.53E-07 | 5.10E-06 | 6.792201 |
| ADAP2 | 1.344444 | 0.027188 | 6.221957 | 2.57E-07 | 5.13E-06 | 6.776846 |
| HMGCR | -1.2976 | 0.140574 | -6.21766 | 2.61E-07 | 5.19E-06 | 6.763492 |
| HOXA9 | 3.028666 | -1.02011 | 6.21411 | 2.64E-07 | 5.24E-06 | 6.752443 |
| SOD2 | 1.253657 | 0.116078 | 6.210779 | 2.67E-07 | 5.28E-06 | 6.742083 |
| PDXDC2P | -1.07201 | 0.283609 | -6.20856 | 2.68E-07 | 5.30E-06 | 6.735196 |
| SNX24 | -1.00468 | -0.03472 | -6.19971 | 2.76E-07 | 5.41E-06 | 6.707654 |
| SENP3 | -1.13981 | 0.277498 | -6.19561 | 2.80E-07 | 5.47E-06 | 6.694918 |
| LOC202181 | -1.76898 | 0.479579 | -6.1919 | 2.83E-07 | 5.53E-06 | 6.68338 |
| PRPH | -1.74222 | 0.708732 | -6.19098 | 2.84E-07 | 5.54E-06 | 6.68052 |
| RAB5B | -1.02672 | -0.01817 | -6.18841 | 2.86E-07 | 5.58E-06 | 6.67251 |
| UST | -1.28695 | 0.098396 | -6.18771 | 2.87E-07 | 5.58E-06 | 6.670339 |
| STOX2 | -1.08489 | 0.116352 | -6.18698 | 2.88E-07 | 5.59E-06 | 6.668063 |
| TFAP2A | 2.647924 | -1.00915 | 6.175758 | 2.98E-07 | 5.74E-06 | 6.633164 |
| ARL4C | -1.18496 | 0.309852 | -6.17239 | 3.01E-07 | 5.79E-06 | 6.622704 |
| DOCK4 | 1.049176 | 0.004137 | 6.170592 | 3.03E-07 | 5.82E-06 | 6.617097 |
| SCIN | -1.7608 | -0.03835 | -6.1696 | 3.04E-07 | 5.83E-06 | 6.61401 |
| C5orf38 | 1.61891 | -0.35874 | 6.166485 | 3.07E-07 | 5.88E-06 | 6.604323 |
| SLC37A2 | 1.39366 | -0.10747 | 6.16517 | 3.08E-07 | 5.90E-06 | 6.600231 |
| EBI3 | 1.149906 | 0.048284 | 6.160703 | 3.13E-07 | 5.95E-06 | 6.58634 |
| SERPINE1 | 1.04598 | -0.37391 | 6.151424 | 3.22E-07 | 6.10E-06 | 6.557477 |
| LGALS2 | 2.034019 | 0.000968 | 6.133248 | 3.41E-07 | 6.40E-06 | 6.500938 |
| CALHM3 | 1.883981 | -0.51073 | 6.130744 | 3.44E-07 | 6.45E-06 | 6.49315 |
| LIF | 1.266497 | -0.4804 | 6.128682 | 3.47E-07 | 6.48E-06 | 6.486736 |
| CPVL | 1.59207 | -0.20423 | 6.127846 | 3.47E-07 | 6.49E-06 | 6.484135 |
| GJA3 | 1.128386 | 0.020821 | 6.120846 | 3.55E-07 | 6.62E-06 | 6.462362 |
| FMO5 | -1.24553 | -0.03419 | -6.11868 | 3.58E-07 | 6.66E-06 | 6.455632 |
| DISP1 | -1.46106 | 0.360885 | -6.11672 | 3.60E-07 | 6.69E-06 | 6.449536 |
| IFITM3 | 1.10101 | 0.027226 | 6.115054 | 3.62E-07 | 6.71E-06 | 6.444343 |
| ZNF767 | -1.15825 | 0.47854 | -6.1148 | 3.62E-07 | 6.71E-06 | 6.443569 |
| SETD5 | -1.08843 | 0.171676 | -6.11338 | 3.64E-07 | 6.73E-06 | 6.439138 |
| ZNF772 | -1.36069 | 0.040023 | -6.11132 | 3.66E-07 | 6.76E-06 | 6.432743 |
| SUN2 | -1.00603 | 0.283644 | -6.107 | 3.71E-07 | 6.81E-06 | 6.419293 |
| TYROBP | 1.61638 | 0.022784 | 6.105663 | 3.73E-07 | 6.84E-06 | 6.415131 |
| CDA | 2.124935 | -0.60262 | 6.104972 | 3.74E-07 | 6.84E-06 | 6.412984 |
| SPAG5-AS1 | -1.80848 | 0.373972 | -6.09451 | 3.86E-07 | 7.02E-06 | 6.380426 |
| OR10J5 | 1.092446 | -0.27933 | 6.092137 | 3.89E-07 | 7.06E-06 | 6.373056 |
| BCL2A1 | 1.409478 | 0.086459 | 6.092103 | 3.89E-07 | 7.06E-06 | 6.372949 |
| NFE2L2 | -1.07792 | 0.085142 | -6.09036 | 3.92E-07 | 7.09E-06 | 6.367526 |
| GLRX | 1.173279 | -0.13922 | 6.087235 | 3.96E-07 | 7.13E-06 | 6.357808 |
| CCDC19 | -1.32929 | 0.107438 | -6.08689 | 3.96E-07 | 7.13E-06 | 6.356743 |
| LOC100652730 | 2.861037 | -1.54209 | 6.086097 | 3.97E-07 | 7.14E-06 | 6.354267 |
| SYTL5 | -1.86845 | 0.135846 | -6.08532 | 3.98E-07 | 7.15E-06 | 6.351841 |
| RARRES1 | 2.499951 | -0.89578 | 6.083123 | 4.01E-07 | 7.19E-06 | 6.345015 |
| TMEM151A | -2.02411 | 0.50212 | -6.08122 | 4.03E-07 | 7.22E-06 | 6.339089 |
| AQP2 | 1.20427 | -0.4343 | 6.075517 | 4.11E-07 | 7.32E-06 | 6.321356 |
| LPCAT3 | -1.00959 | -0.00785 | -6.06645 | 4.23E-07 | 7.52E-06 | 6.293163 |
| LY86 | 1.302901 | 0.189341 | 6.066159 | 4.23E-07 | 7.52E-06 | 6.292245 |
| VLDLR | -1.48821 | 0.058037 | -6.06557 | 4.24E-07 | 7.52E-06 | 6.290411 |
| C1orf201 | -1.02602 | -0.01275 | -6.06386 | 4.26E-07 | 7.56E-06 | 6.285086 |
| CYTIP | -1.31588 | 0.260774 | -6.06131 | 4.30E-07 | 7.60E-06 | 6.277151 |
| ZFYVE28 | -1.31216 | 0.101688 | -6.05969 | 4.32E-07 | 7.63E-06 | 6.272136 |
| PTGS2 | 2.847207 | -0.00662 | 6.033362 | 4.70E-07 | 8.22E-06 | 6.190223 |
| FGL1 | 1.58258 | -0.36792 | 6.032426 | 4.71E-07 | 8.23E-06 | 6.187311 |
| ATP13A4 | -2.20923 | 0.508197 | -6.02888 | 4.77E-07 | 8.31E-06 | 6.176273 |
| RNASE3 | 1.468575 | -0.09722 | 6.025334 | 4.82E-07 | 8.39E-06 | 6.16525 |
| C17orf108 | -1.28251 | 0.373606 | -6.01821 | 4.93E-07 | 8.54E-06 | 6.143076 |
| MUC1 | -1.43115 | 0.15274 | -6.01725 | 4.95E-07 | 8.55E-06 | 6.140116 |
| MMP9 | 2.167775 | 0.167465 | 6.016103 | 4.96E-07 | 8.57E-06 | 6.136537 |
| CATSPER2 | -1.19736 | 0.440099 | -6.01392 | 5.00E-07 | 8.61E-06 | 6.12976 |
| PPP1R1C | -1.61668 | 0.004817 | -6.00846 | 5.09E-07 | 8.74E-06 | 6.112762 |
| HTRA4 | 1.886571 | -0.24455 | 6.007787 | 5.10E-07 | 8.74E-06 | 6.110668 |
| ZNF454 | -1.65955 | 0.761206 | -6.00544 | 5.14E-07 | 8.80E-06 | 6.103369 |
| CPAMD8 | -1.50721 | 0.517687 | -6.00493 | 5.15E-07 | 8.81E-06 | 6.101768 |
| HLA-DOA | 1.149399 | 0.023992 | 5.997803 | 5.26E-07 | 8.98E-06 | 6.079616 |
| CST2 | 1.06026 | -0.60243 | 5.990864 | 5.38E-07 | 9.14E-06 | 6.058032 |
| SERTAD2 | -1.02559 | 0.351788 | -5.98855 | 5.42E-07 | 9.19E-06 | 6.050836 |
| TST | -1.07563 | 0.000895 | -5.9884 | 5.42E-07 | 9.19E-06 | 6.050368 |
| LANCL3 | -1.68884 | -0.07972 | -5.98512 | 5.48E-07 | 9.26E-06 | 6.040152 |
| F2R | 1.285452 | -0.05237 | 5.98452 | 5.49E-07 | 9.27E-06 | 6.038299 |
| C17orf58 | -1.04474 | 0.213591 | -5.98295 | 5.52E-07 | 9.31E-06 | 6.033428 |
| CHAD | -2.00861 | 0.790614 | -5.9817 | 5.54E-07 | 9.33E-06 | 6.029518 |
| FSTL4 | -1.79468 | 0.241307 | -5.98091 | 5.56E-07 | 9.34E-06 | 6.027062 |
| CDH3 | 2.812978 | -1.11354 | 5.978681 | 5.60E-07 | 9.38E-06 | 6.020137 |
| SCARNA17 | -1.54083 | 0.640635 | -5.97771 | 5.61E-07 | 9.40E-06 | 6.017102 |
| PCDH7 | 1.144305 | 0.119244 | 5.975383 | 5.65E-07 | 9.46E-06 | 6.009878 |
| C4BPA | 1.738012 | -0.25643 | 5.972958 | 5.70E-07 | 9.52E-06 | 6.002335 |
| NRXN2 | 1.111018 | -0.23991 | 5.972429 | 5.71E-07 | 9.53E-06 | 6.000692 |
| IL10 | 1.247132 | -0.17881 | 5.972336 | 5.71E-07 | 9.53E-06 | 6.000402 |
| GUSBP1 | -1.09057 | 0.285294 | -5.97152 | 5.72E-07 | 9.54E-06 | 5.997862 |
| NANOS3 | 2.392882 | -0.52745 | 5.970721 | 5.74E-07 | 9.55E-06 | 5.995379 |
| C4orf38 | -1.36458 | 0.342479 | -5.96706 | 5.81E-07 | 9.63E-06 | 5.983999 |
| ZNF132 | -1.44462 | 0.26036 | -5.96379 | 5.87E-07 | 9.71E-06 | 5.973837 |
| GPRIN2 | -1.3609 | 0.252946 | -5.9562 | 6.01E-07 | 9.93E-06 | 5.950215 |
| CPA3 | 1.177269 | 0.114193 | 5.955678 | 6.02E-07 | 9.94E-06 | 5.948593 |
| CDH15 | 1.873321 | -0.38549 | 5.952387 | 6.09E-07 | 1.00E-05 | 5.938356 |
| SELENBP1 | -1.30119 | -0.08696 | -5.94778 | 6.18E-07 | 1.02E-05 | 5.924032 |
| WSCD2 | -2.61588 | 0.092063 | -5.94555 | 6.22E-07 | 1.02E-05 | 5.917109 |
| LOC100505576 | -1.46096 | 0.391724 | -5.94261 | 6.28E-07 | 1.03E-05 | 5.907965 |
| FOXD1 | 1.931914 | -0.18478 | 5.939548 | 6.34E-07 | 1.04E-05 | 5.898427 |
| ASAP3 | -1.09536 | 0.387917 | -5.93078 | 6.52E-07 | 1.07E-05 | 5.871161 |
| CYP2C9 | -1.95614 | 0.283839 | -5.93022 | 6.53E-07 | 1.07E-05 | 5.869428 |
| PAQR8 | -1.20041 | 0.039707 | -5.92527 | 6.64E-07 | 1.08E-05 | 5.854015 |
| SCNN1A | -1.62039 | 0.062667 | -5.92472 | 6.65E-07 | 1.08E-05 | 5.852322 |
| SNX10 | 1.627643 | 0.081331 | 5.921234 | 6.72E-07 | 1.09E-05 | 5.841476 |
| RHPN2 | -1.10188 | -0.03995 | -5.91813 | 6.79E-07 | 1.09E-05 | 5.83181 |
| FCN3 | 1.355485 | -0.10603 | 5.917459 | 6.80E-07 | 1.10E-05 | 5.829737 |
| FRMD4A | -2.18328 | 0.758635 | -5.91243 | 6.91E-07 | 1.11E-05 | 5.814096 |
| STAT6 | -1.24176 | 0.258588 | -5.91148 | 6.94E-07 | 1.11E-05 | 5.811147 |
| CCDC153 | -1.20303 | 0.200586 | -5.90923 | 6.99E-07 | 1.12E-05 | 5.804157 |
| SAMD4A | 1.217267 | 0.002202 | 5.907405 | 7.03E-07 | 1.12E-05 | 5.798473 |
| HPCA | -1.23341 | 0.081293 | -5.90452 | 7.09E-07 | 1.13E-05 | 5.789501 |
| HOTTIP | 1.178598 | -0.41565 | 5.900502 | 7.18E-07 | 1.14E-05 | 5.77701 |
| UPK1B | -4.38232 | -0.48406 | -5.89901 | 7.22E-07 | 1.15E-05 | 5.772361 |
| SLC1A3 | 1.204327 | 0.005763 | 5.897152 | 7.26E-07 | 1.15E-05 | 5.766593 |
| PPIF | -1.17892 | 0.37595 | -5.896 | 7.29E-07 | 1.16E-05 | 5.763002 |
| VNN1 | 3.142189 | -1.19379 | 5.893854 | 7.34E-07 | 1.16E-05 | 5.756338 |
| COMP | 1.459307 | -0.13908 | 5.892957 | 7.36E-07 | 1.17E-05 | 5.753549 |
| NKAIN2 | 1.384966 | -0.06032 | 5.891144 | 7.40E-07 | 1.17E-05 | 5.747912 |
| IL17RE | -1.16295 | 0.298711 | -5.89031 | 7.42E-07 | 1.17E-05 | 5.745328 |
| MIR22HG | -1.2343 | 0.180222 | -5.88939 | 7.44E-07 | 1.18E-05 | 5.742448 |
| C1orf38 | 1.198455 | 0.11271 | 5.882016 | 7.62E-07 | 1.20E-05 | 5.71953 |
| CYBB | 1.104457 | 0.245978 | 5.88115 | 7.64E-07 | 1.20E-05 | 5.716839 |
| C2orf65 | 1.474079 | 0.220708 | 5.880709 | 7.65E-07 | 1.20E-05 | 5.715469 |
| S100A8 | 3.884022 | -0.18425 | 5.879738 | 7.68E-07 | 1.20E-05 | 5.71245 |
| AXIN2 | -1.53263 | -0.00216 | -5.87253 | 7.85E-07 | 1.23E-05 | 5.690037 |
| CTHRC1 | 1.644232 | 0.102667 | 5.872045 | 7.87E-07 | 1.23E-05 | 5.688532 |
| BLVRB | -1.04954 | 0.10164 | -5.8653 | 8.04E-07 | 1.25E-05 | 5.667577 |
| DHH | -1.04904 | 0.028895 | -5.86379 | 8.08E-07 | 1.25E-05 | 5.662876 |
| C11orf67 | -1.22579 | 0.318243 | -5.86299 | 8.10E-07 | 1.26E-05 | 5.660368 |
| RBM14 | -1.02255 | 0.185058 | -5.86038 | 8.16E-07 | 1.27E-05 | 5.652279 |
| C19orf71 | -1.39476 | 0.344302 | -5.85846 | 8.22E-07 | 1.27E-05 | 5.646312 |
| GTF2IRD2 | -1.10211 | 0.144307 | -5.85807 | 8.23E-07 | 1.27E-05 | 5.645076 |
| KRT6A | 2.664701 | -0.05476 | 5.857493 | 8.24E-07 | 1.27E-05 | 5.643294 |
| SORL1 | -1.17626 | 0.411124 | -5.85519 | 8.30E-07 | 1.28E-05 | 5.636143 |
| FAIM3 | 1.174664 | 0.058128 | 5.851247 | 8.41E-07 | 1.29E-05 | 5.623878 |
| SH2D6 | -1.51231 | 0.551675 | -5.84971 | 8.45E-07 | 1.30E-05 | 5.619095 |
| TRIM47 | -1.02799 | 0.037327 | -5.84558 | 8.56E-07 | 1.31E-05 | 5.606254 |
| L3MBTL4 | -1.17365 | 0.236779 | -5.84178 | 8.66E-07 | 1.33E-05 | 5.594448 |
| PAX6 | -2.73818 | 0.513596 | -5.84108 | 8.68E-07 | 1.33E-05 | 5.592273 |
| IL2RA | 1.995648 | 7.35E-05 | 5.836177 | 8.82E-07 | 1.34E-05 | 5.577035 |
| ZNF81 | -1.06525 | 0.338781 | -5.83505 | 8.85E-07 | 1.35E-05 | 5.57353 |
| NR4A1 | 1.990732 | -0.42277 | 5.834897 | 8.86E-07 | 1.35E-05 | 5.573056 |
| FLJ43390 | -2.27673 | 1.369046 | -5.83088 | 8.97E-07 | 1.36E-05 | 5.560573 |
| LOC100288144 | -1.64739 | 0.674221 | -5.82935 | 9.02E-07 | 1.37E-05 | 5.555821 |
| SLC5A10 | -1.75399 | 0.751289 | -5.82446 | 9.16E-07 | 1.38E-05 | 5.540619 |
| ADIPOR2 | -1.02257 | -0.00187 | -5.82391 | 9.17E-07 | 1.38E-05 | 5.538897 |
| FZD8 | -2.29572 | 0.388315 | -5.82121 | 9.25E-07 | 1.39E-05 | 5.530531 |
| AMT | -1.20637 | -0.12542 | -5.8185 | 9.33E-07 | 1.40E-05 | 5.522086 |
| LOC100131053 | -1.02982 | 0.338232 | -5.81759 | 9.36E-07 | 1.41E-05 | 5.519278 |
| C21orf30 | -1.49952 | -0.03882 | -5.81527 | 9.43E-07 | 1.42E-05 | 5.512066 |
| MRAP2 | -1.40614 | -0.08345 | -5.81261 | 9.51E-07 | 1.42E-05 | 5.503789 |
| CNTD2 | 1.086673 | -0.57698 | 5.811877 | 9.53E-07 | 1.42E-05 | 5.501514 |
| SAA1 | 3.87635 | -0.62462 | 5.810271 | 9.58E-07 | 1.43E-05 | 5.496523 |
| CHD5 | 1.148931 | -0.1215 | 5.802897 | 9.81E-07 | 1.46E-05 | 5.473611 |
| TAGLN3 | -2.5402 | 0.729124 | -5.8015 | 9.85E-07 | 1.46E-05 | 5.469254 |
| ID4 | -2.05876 | 0.368451 | -5.80076 | 9.88E-07 | 1.46E-05 | 5.466968 |
| TRIM72 | 1.011156 | -0.41888 | 5.796295 | 1.00E-06 | 1.48E-05 | 5.453097 |
| TGFA | -1.50538 | 0.125522 | -5.79114 | 1.02E-06 | 1.50E-05 | 5.437075 |
| FCGR3A | 2.672842 | 0.035566 | 5.789647 | 1.02E-06 | 1.51E-05 | 5.432441 |
| APOC1 | 1.17184 | 0.272765 | 5.785171 | 1.04E-06 | 1.52E-05 | 5.418534 |
| PDZK1IP1 | 1.379296 | -0.10826 | 5.784269 | 1.04E-06 | 1.53E-05 | 5.41573 |
| XKR6 | -1.04387 | 0.200503 | -5.78132 | 1.05E-06 | 1.54E-05 | 5.406584 |
| C6orf204 | -1.43565 | 0.280086 | -5.77755 | 1.06E-06 | 1.55E-05 | 5.39486 |
| HOXA11-AS1 | 5.196074 | -2.33011 | 5.769672 | 1.09E-06 | 1.59E-05 | 5.370384 |
| CST1 | 1.509294 | -0.53647 | 5.769659 | 1.09E-06 | 1.59E-05 | 5.370344 |
| FOXC1 | 1.760143 | -0.4244 | 5.765188 | 1.11E-06 | 1.60E-05 | 5.356454 |
| RLBP1 | 1.128862 | -0.29884 | 5.759123 | 1.13E-06 | 1.63E-05 | 5.337615 |
| ZNF404 | -2.0352 | 0.450346 | -5.75395 | 1.15E-06 | 1.65E-05 | 5.321537 |
| S100A9 | 3.360355 | -0.01125 | 5.751586 | 1.16E-06 | 1.66E-05 | 5.314207 |
| HDHD1 | -1.28317 | 0.377666 | -5.74933 | 1.16E-06 | 1.67E-05 | 5.307202 |
| KRT14 | 2.110288 | -1.08766 | 5.748852 | 1.17E-06 | 1.67E-05 | 5.305713 |
| SPTSSB | -2.27247 | 0.124434 | -5.74473 | 1.18E-06 | 1.69E-05 | 5.292924 |
| METTL7A | -1.13701 | 0.276766 | -5.74182 | 1.19E-06 | 1.70E-05 | 5.283887 |
| FPR1 | 2.975579 | 0.058187 | 5.736889 | 1.21E-06 | 1.72E-05 | 5.268562 |
| LGMN | -1.22498 | 0.131948 | -5.73349 | 1.22E-06 | 1.74E-05 | 5.257999 |
| MICALCL | -1.14083 | 0.001063 | -5.7332 | 1.23E-06 | 1.74E-05 | 5.2571 |
| SYT12 | 1.467548 | -0.49831 | 5.730849 | 1.23E-06 | 1.75E-05 | 5.249805 |
| GPRC6A | 1.126812 | -0.27907 | 5.727241 | 1.25E-06 | 1.77E-05 | 5.238603 |
| ZNF671 | -1.28414 | 0.423061 | -5.72232 | 1.27E-06 | 1.79E-05 | 5.223307 |
| GDF15 | 1.781979 | -0.40044 | 5.720554 | 1.28E-06 | 1.80E-05 | 5.217838 |
| LINC00256A | 1.075712 | -0.13083 | 5.718819 | 1.28E-06 | 1.81E-05 | 5.212453 |
| CMBL | -1.31142 | 0.080666 | -5.71074 | 1.32E-06 | 1.85E-05 | 5.187359 |
| LOC100507153 | -1.63168 | 0.298189 | -5.70903 | 1.32E-06 | 1.85E-05 | 5.182068 |
| SIGLEC5 | 1.337219 | -0.10405 | 5.706654 | 1.33E-06 | 1.86E-05 | 5.174684 |
| LOC100129115 | -1.11711 | 0.100898 | -5.70647 | 1.33E-06 | 1.86E-05 | 5.174128 |
| ANO1 | -1.67896 | 0.505411 | -5.69651 | 1.38E-06 | 1.91E-05 | 5.143189 |
| HOXC10 | 2.789917 | 0.962575 | 5.691213 | 1.40E-06 | 1.94E-05 | 5.126754 |
| TNNC1 | 2.67217 | -0.36832 | 5.690704 | 1.40E-06 | 1.94E-05 | 5.125174 |
| HOXA11 | 1.787338 | 0.056716 | 5.686304 | 1.42E-06 | 1.97E-05 | 5.111519 |
| SUN3 | 1.122717 | -0.12487 | 5.686029 | 1.42E-06 | 1.97E-05 | 5.110665 |
| MAGI1 | -1.032 | 0.327856 | -5.6832 | 1.44E-06 | 1.98E-05 | 5.101887 |
| SYT8 | -1.94583 | 0.329026 | -5.6826 | 1.44E-06 | 1.99E-05 | 5.100036 |
| PPP1R32 | -1.15169 | 0.322118 | -5.68253 | 1.44E-06 | 1.99E-05 | 5.099804 |
| SLC44A4 | -1.12488 | 0.02767 | -5.68007 | 1.45E-06 | 2.00E-05 | 5.092158 |
| LOC100131564 | -1.50789 | 0.476536 | -5.67819 | 1.46E-06 | 2.01E-05 | 5.086346 |
| CDHR3 | -1.43239 | 0.269827 | -5.67635 | 1.47E-06 | 2.01E-05 | 5.08062 |
| DNAH1 | -1.14642 | 0.307612 | -5.67398 | 1.48E-06 | 2.03E-05 | 5.073269 |
| LOC202025 | -1.68611 | 0.430011 | -5.67115 | 1.49E-06 | 2.04E-05 | 5.064476 |
| SIGLEC10 | 1.99209 | -0.16969 | 5.6687 | 1.51E-06 | 2.06E-05 | 5.056888 |
| GPR84 | 1.396318 | -0.48782 | 5.668461 | 1.51E-06 | 2.06E-05 | 5.056146 |
| S100A12 | 3.54007 | -0.13527 | 5.662699 | 1.54E-06 | 2.09E-05 | 5.038265 |
| TEF | -1.4031 | 0.379858 | -5.66217 | 1.54E-06 | 2.09E-05 | 5.036628 |
| KIAA1199 | 1.47555 | 0.135818 | 5.661177 | 1.54E-06 | 2.10E-05 | 5.033543 |
| EMR1 | 1.916114 | -0.10656 | 5.660251 | 1.55E-06 | 2.10E-05 | 5.030671 |
| CAMK2B | -1.7765 | 0.257625 | -5.65996 | 1.55E-06 | 2.10E-05 | 5.029764 |
| LOC400756 | -1.56243 | 0.165719 | -5.65933 | 1.55E-06 | 2.10E-05 | 5.027827 |
| EFNA2 | 1.509577 | -0.53218 | 5.658263 | 1.56E-06 | 2.11E-05 | 5.024503 |
| TRNP1 | -1.1727 | -0.04314 | -5.65104 | 1.59E-06 | 2.15E-05 | 5.002087 |
| NBPF22P | 1.312478 | -0.17412 | 5.649566 | 1.60E-06 | 2.16E-05 | 4.997522 |
| EMR3 | 2.237455 | -0.19837 | 5.629415 | 1.71E-06 | 2.28E-05 | 4.935016 |
| SLC4A11 | 1.037411 | -0.13357 | 5.624303 | 1.74E-06 | 2.32E-05 | 4.919164 |
| TP73-AS1 | -1.60613 | 0.268921 | -5.61873 | 1.77E-06 | 2.35E-05 | 4.901877 |
| PTGDS | 2.157639 | 0.057317 | 5.616849 | 1.78E-06 | 2.36E-05 | 4.896051 |
| CHGB | -3.63492 | 1.261938 | -5.61663 | 1.78E-06 | 2.36E-05 | 4.895378 |
| CRYGC | -2.19713 | 0.926828 | -5.61197 | 1.80E-06 | 2.39E-05 | 4.88092 |
| ARSJ | -1.30215 | 0.081434 | -5.60617 | 1.84E-06 | 2.43E-05 | 4.862952 |
| FAM83A | 1.571664 | -0.35618 | 5.604767 | 1.85E-06 | 2.43E-05 | 4.858589 |
| GMPPB | -1.03914 | 0.210649 | -5.60462 | 1.85E-06 | 2.43E-05 | 4.858141 |
| LILRB2 | 1.720234 | 0.036121 | 5.593559 | 1.91E-06 | 2.51E-05 | 4.823845 |
| PIP | 1.853189 | -0.46822 | 5.590563 | 1.93E-06 | 2.53E-05 | 4.814559 |
| LOC100288432 | 1.925488 | -0.65425 | 5.586897 | 1.96E-06 | 2.55E-05 | 4.803196 |
| ADAM28 | -1.52081 | 0.337613 | -5.58576 | 1.96E-06 | 2.56E-05 | 4.799659 |
| C12orf36 | -2.20227 | -0.03393 | -5.58516 | 1.97E-06 | 2.56E-05 | 4.797805 |
| C19orf57 | -1.03688 | 0.031454 | -5.58351 | 1.98E-06 | 2.58E-05 | 4.792685 |
| LNX1 | -1.02286 | 0.09524 | -5.56971 | 2.07E-06 | 2.67E-05 | 4.74992 |
| GALNT5 | -1.20855 | -0.04831 | -5.5669 | 2.08E-06 | 2.69E-05 | 4.741231 |
| GABRA1 | -1.63774 | 0.549378 | -5.56536 | 2.09E-06 | 2.70E-05 | 4.736442 |
| PI16 | -1.53093 | 0.332513 | -5.56378 | 2.10E-06 | 2.71E-05 | 4.731554 |
| NR5A1 | 1.259548 | -0.15807 | 5.562255 | 2.11E-06 | 2.72E-05 | 4.726836 |
| SLC22A24 | 1.229671 | -0.20506 | 5.561072 | 2.12E-06 | 2.73E-05 | 4.723172 |
| HMP19 | -2.08893 | 0.73544 | -5.55948 | 2.13E-06 | 2.74E-05 | 4.718239 |
| KLK8 | 4.227217 | -0.34981 | 5.554307 | 2.17E-06 | 2.78E-05 | 4.702213 |
| BAHCC1 | -1.15766 | 0.274032 | -5.55386 | 2.17E-06 | 2.78E-05 | 4.700818 |
| RTN1 | -1.75431 | 0.690581 | -5.54328 | 2.25E-06 | 2.86E-05 | 4.66807 |
| LILRB3 | 1.600949 | 0.03846 | 5.542937 | 2.25E-06 | 2.86E-05 | 4.666996 |
| CA9 | -2.53362 | 0.178954 | -5.54009 | 2.27E-06 | 2.88E-05 | 4.658189 |
| MYO15A | -1.04498 | 0.265462 | -5.5382 | 2.28E-06 | 2.89E-05 | 4.65231 |
| WISP3 | 3.242418 | -0.56654 | 5.537082 | 2.29E-06 | 2.90E-05 | 4.648861 |
| DUSP5P | 1.009143 | 0.006214 | 5.528711 | 2.35E-06 | 2.96E-05 | 4.622941 |
| RASSF4 | 1.348346 | -0.51604 | 5.52859 | 2.35E-06 | 2.96E-05 | 4.622566 |
| CLEC4E | 3.046414 | 0.039779 | 5.524782 | 2.38E-06 | 2.99E-05 | 4.610777 |
| HOXB6 | 1.90926 | -0.50413 | 5.52337 | 2.39E-06 | 3.00E-05 | 4.606403 |
| CACNA2D2 | -1.6818 | 0.574938 | -5.52311 | 2.40E-06 | 3.00E-05 | 4.605587 |
| ZSCAN18 | -1.59761 | 0.535587 | -5.52243 | 2.40E-06 | 3.00E-05 | 4.603499 |
| LOC158402 | -1.01675 | 0.102622 | -5.52026 | 2.42E-06 | 3.02E-05 | 4.596764 |
| KCTD13 | -1.25039 | 0.397678 | -5.51696 | 2.44E-06 | 3.04E-05 | 4.586573 |
| C2orf70 | -1.16464 | 0.193129 | -5.51662 | 2.45E-06 | 3.04E-05 | 4.585497 |
| AKR1B1 | -1.37116 | -0.20567 | -5.51476 | 2.46E-06 | 3.06E-05 | 4.579753 |
| BHLHE40 | -1.11109 | 0.316606 | -5.5103 | 2.50E-06 | 3.09E-05 | 4.565944 |
| C1orf114 | 1.085987 | -0.12135 | 5.508725 | 2.51E-06 | 3.10E-05 | 4.561069 |
| QPRT | 1.509646 | -0.19318 | 5.503655 | 2.55E-06 | 3.15E-05 | 4.545377 |
| INHBA | 1.802211 | 0.07338 | 5.501533 | 2.57E-06 | 3.16E-05 | 4.538808 |
| LOC100506630 | -2.90983 | 1.080896 | -5.50015 | 2.58E-06 | 3.17E-05 | 4.534521 |
| CHST2 | 1.066439 | 0.122421 | 5.499935 | 2.58E-06 | 3.17E-05 | 4.533864 |
| CD300E | 1.224127 | -0.37538 | 5.492993 | 2.64E-06 | 3.23E-05 | 4.512381 |
| CYP2B7P1 | -1.25513 | 0.264809 | -5.49173 | 2.65E-06 | 3.24E-05 | 4.508483 |
| PIWIL2 | -1.57433 | 0.255551 | -5.48908 | 2.67E-06 | 3.27E-05 | 4.500285 |
| GAD1 | 1.345028 | 0.048019 | 5.488088 | 2.68E-06 | 3.27E-05 | 4.497205 |
| SYTL1 | -1.09219 | -0.03268 | -5.48038 | 2.75E-06 | 3.35E-05 | 4.473369 |
| GCKR | -1.36986 | 0.269187 | -5.47924 | 2.76E-06 | 3.36E-05 | 4.469842 |
| EDN1 | 1.569635 | -0.52175 | 5.471552 | 2.82E-06 | 3.42E-05 | 4.446047 |
| SIPA1L2 | 1.298746 | -0.15836 | 5.470918 | 2.83E-06 | 3.43E-05 | 4.444087 |
| FRAT1 | -1.13727 | 0.123926 | -5.45907 | 2.94E-06 | 3.55E-05 | 4.40746 |
| LOC100272228 | -2.29033 | 0.825697 | -5.45825 | 2.95E-06 | 3.55E-05 | 4.404922 |
| BAI2 | 1.028536 | 0.005844 | 5.454797 | 2.98E-06 | 3.58E-05 | 4.394232 |
| ITGB4 | -1.05298 | 0.099201 | -5.45451 | 2.98E-06 | 3.59E-05 | 4.393331 |
| CLDN18 | -1.31421 | -0.03104 | -5.45411 | 2.99E-06 | 3.59E-05 | 4.392095 |
| MTMR9LP | -1.08557 | 0.338665 | -5.45146 | 3.01E-06 | 3.61E-05 | 4.38393 |
| KRT17 | 3.890207 | -2.29439 | 5.449929 | 3.03E-06 | 3.62E-05 | 4.379181 |
| ZBED2 | 1.860887 | 0.074739 | 5.443633 | 3.09E-06 | 3.68E-05 | 4.359718 |
| LOC100507218 | -2.30284 | 0.455112 | -5.43946 | 3.13E-06 | 3.72E-05 | 4.346814 |
| TREM1 | 2.468775 | -0.23109 | 5.436608 | 3.16E-06 | 3.74E-05 | 4.338002 |
| AKR7A2P1 | -1.18677 | -0.1219 | -5.43638 | 3.16E-06 | 3.74E-05 | 4.337298 |
| HOXC8 | 1.341246 | 0.394661 | 5.432694 | 3.20E-06 | 3.77E-05 | 4.325906 |
| FCN1 | 1.989933 | 0.074953 | 5.430871 | 3.21E-06 | 3.79E-05 | 4.320273 |
| PBX1 | -1.00491 | 0.30158 | -5.42637 | 3.26E-06 | 3.83E-05 | 4.306353 |
| PLAC1 | 1.961012 | -0.28132 | 5.422515 | 3.30E-06 | 3.86E-05 | 4.294449 |
| WISP1 | 1.488307 | 0.163615 | 5.421457 | 3.31E-06 | 3.87E-05 | 4.291183 |
| DDX10 | 1.485128 | -0.31811 | 5.416327 | 3.37E-06 | 3.93E-05 | 4.275331 |
| SHISA6 | -2.66979 | 0.623709 | -5.41141 | 3.42E-06 | 3.98E-05 | 4.260152 |
| KBTBD8 | 1.082557 | -0.16612 | 5.398187 | 3.57E-06 | 4.12E-05 | 4.2193 |
| C5AR1 | 1.770495 | -0.46993 | 5.396272 | 3.59E-06 | 4.14E-05 | 4.213385 |
| HS3ST5 | -2.23378 | 0.699126 | -5.39586 | 3.59E-06 | 4.15E-05 | 4.212102 |
| LGALS12 | 1.042666 | -0.01806 | 5.38966 | 3.66E-06 | 4.22E-05 | 4.192968 |
| KIAA1652 | -1.74063 | 0.470626 | -5.38854 | 3.68E-06 | 4.23E-05 | 4.18952 |
| LILRB1 | 1.882555 | 0.183575 | 5.382883 | 3.74E-06 | 4.30E-05 | 4.172045 |
| SLC9A2 | -1.0375 | -0.20189 | -5.38202 | 3.75E-06 | 4.31E-05 | 4.16937 |
| WFDC2 | 1.593222 | -0.0627 | 5.381953 | 3.76E-06 | 4.31E-05 | 4.169176 |
| ASPG | -2.4644 | 0.173001 | -5.3789 | 3.79E-06 | 4.33E-05 | 4.159739 |
| ECEL1 | 1.140914 | -0.13328 | 5.37869 | 3.79E-06 | 4.33E-05 | 4.159101 |
| SIGLEC7 | 1.24134 | -0.04649 | 5.374666 | 3.84E-06 | 4.36E-05 | 4.146682 |
| OR1E1 | 1.367868 | -0.85134 | 5.370294 | 3.90E-06 | 4.42E-05 | 4.133188 |
| TREM2 | 1.724873 | -0.07452 | 5.368728 | 3.92E-06 | 4.43E-05 | 4.128355 |
| VAV3 | 2.115659 | -0.58744 | 5.368463 | 3.92E-06 | 4.43E-05 | 4.127536 |
| CRYGS | -1.29076 | 0.454297 | -5.36713 | 3.94E-06 | 4.45E-05 | 4.123417 |
| PTPRZ1 | -1.88504 | 0.76673 | -5.36169 | 4.01E-06 | 4.49E-05 | 4.106624 |
| ATOH1 | 1.126717 | -0.05385 | 5.360846 | 4.02E-06 | 4.50E-05 | 4.104031 |
| EGR3 | 2.047904 | -0.19875 | 5.359777 | 4.03E-06 | 4.51E-05 | 4.100734 |
| CHRM4 | 1.240734 | -0.14321 | 5.357495 | 4.06E-06 | 4.54E-05 | 4.093692 |
| TRPV1 | -1.07166 | 0.173504 | -5.35645 | 4.07E-06 | 4.54E-05 | 4.090477 |
| ZYG11A | 1.773048 | 0.387301 | 5.353273 | 4.11E-06 | 4.58E-05 | 4.080668 |
| IDO1 | 2.015926 | 0.46077 | 5.346199 | 4.21E-06 | 4.67E-05 | 4.058846 |
| FMO4 | -1.08851 | -0.1672 | -5.34042 | 4.29E-06 | 4.74E-05 | 4.04102 |
| LST1 | 1.517585 | 0.158263 | 5.336647 | 4.34E-06 | 4.79E-05 | 4.029388 |
| LOC100507094 | -1.05098 | 0.401384 | -5.33415 | 4.37E-06 | 4.82E-05 | 4.021679 |
| SCN8A | -1.63078 | 0.680071 | -5.33076 | 4.42E-06 | 4.86E-05 | 4.011247 |
| NNMT | 1.670409 | 0.098563 | 5.32761 | 4.46E-06 | 4.91E-05 | 4.001523 |
| CHRNA7 | -1.39561 | -0.01397 | -5.32623 | 4.48E-06 | 4.92E-05 | 3.997262 |
| NXF3 | 1.724177 | -0.28477 | 5.324996 | 4.50E-06 | 4.94E-05 | 3.993464 |
| CYP2C18 | -1.55883 | -0.09143 | -5.3235 | 4.52E-06 | 4.95E-05 | 3.988844 |
| ARHGDIB | 1.024925 | 0.036149 | 5.322448 | 4.54E-06 | 4.97E-05 | 3.985612 |
| C20orf112 | -1.13207 | -0.13043 | -5.3167 | 4.62E-06 | 5.05E-05 | 3.967888 |
| HOTAIR | 1.889987 | 0.322389 | 5.316179 | 4.63E-06 | 5.06E-05 | 3.966288 |
| CPM | 1.559531 | -0.2437 | 5.315069 | 4.65E-06 | 5.07E-05 | 3.962865 |
| KLHL5 | 1.127128 | 0.125613 | 5.312627 | 4.68E-06 | 5.09E-05 | 3.955341 |
| SLPI | -1.01581 | 0.044027 | -5.31215 | 4.69E-06 | 5.09E-05 | 3.953859 |
| BHMT | -2.27467 | 0.276625 | -5.30856 | 4.74E-06 | 5.15E-05 | 3.942804 |
| MEX3A | 1.425726 | -0.12399 | 5.307998 | 4.75E-06 | 5.15E-05 | 3.941076 |
| FAM105A | 1.201097 | -0.22716 | 5.304733 | 4.80E-06 | 5.19E-05 | 3.931016 |
| NKX6-2 | -4.4113 | -0.84549 | -5.30389 | 4.81E-06 | 5.20E-05 | 3.928427 |
| SCGB2A1 | -2.67136 | -0.20826 | -5.30167 | 4.85E-06 | 5.23E-05 | 3.921567 |
| NCF2 | 1.817991 | 0.271001 | 5.291553 | 5.01E-06 | 5.38E-05 | 3.890411 |
| CYP46A1 | 1.154065 | -0.41345 | 5.289873 | 5.03E-06 | 5.39E-05 | 3.885237 |
| BAIAP3 | -1.45987 | 0.396547 | -5.28911 | 5.04E-06 | 5.40E-05 | 3.882885 |
| KCNQ1OT1 | -1.50031 | 0.517308 | -5.28821 | 5.06E-06 | 5.41E-05 | 3.88011 |
| SSTR2 | -2.03045 | 0.637664 | -5.28557 | 5.10E-06 | 5.45E-05 | 3.871987 |
| TREML2 | 1.267367 | -0.15752 | 5.284939 | 5.11E-06 | 5.45E-05 | 3.870043 |
| MGC16075 | 1.079846 | 0.084303 | 5.283244 | 5.14E-06 | 5.47E-05 | 3.864824 |
| LOC100130331 | -1.02651 | 0.069107 | -5.28202 | 5.16E-06 | 5.49E-05 | 3.861039 |
| COX6B2 | 1.037935 | -0.41448 | 5.277184 | 5.24E-06 | 5.55E-05 | 3.846165 |
| PTPRO | 1.290862 | -0.039 | 5.273695 | 5.30E-06 | 5.61E-05 | 3.835424 |
| GATA1 | 1.081978 | -0.16619 | 5.27314 | 5.31E-06 | 5.61E-05 | 3.833713 |
| TMEM176A | 1.317214 | -0.44202 | 5.270145 | 5.36E-06 | 5.65E-05 | 3.824493 |
| LONRF2 | -1.89086 | 0.136387 | -5.26473 | 5.45E-06 | 5.74E-05 | 3.807823 |
| SMA4 | -1.19702 | 0.380812 | -5.26151 | 5.51E-06 | 5.78E-05 | 3.797928 |
| MYO15B | -1.12135 | 0.365674 | -5.26047 | 5.52E-06 | 5.79E-05 | 3.794703 |
| TMEM178 | -1.33567 | 0.40571 | -5.25894 | 5.55E-06 | 5.81E-05 | 3.789993 |
| SULT4A1 | 1.007857 | -0.16169 | 5.257732 | 5.57E-06 | 5.82E-05 | 3.786289 |
| CPS1-IT1 | 1.24621 | -0.55712 | 5.257332 | 5.58E-06 | 5.82E-05 | 3.785059 |
| BMS1P1 | -1.34844 | 0.209975 | -5.2521 | 5.67E-06 | 5.91E-05 | 3.768959 |
| CIDEC | -1.62025 | 0.006514 | -5.24873 | 5.73E-06 | 5.96E-05 | 3.758578 |
| CLEC4A | 1.31298 | 0.203556 | 5.244951 | 5.80E-06 | 6.01E-05 | 3.746968 |
| SLC35F3 | -1.59435 | 0.691455 | -5.24476 | 5.81E-06 | 6.01E-05 | 3.746373 |
| NKX6-3 | -1.72304 | -0.14023 | -5.24156 | 5.87E-06 | 6.07E-05 | 3.736538 |
| EPHX4 | 1.629471 | -0.16794 | 5.241009 | 5.88E-06 | 6.08E-05 | 3.734843 |
| RHBDL2 | -1.0277 | -0.0151 | -5.23816 | 5.93E-06 | 6.11E-05 | 3.726076 |
| AQP9 | 3.392721 | 0.113647 | 5.236029 | 5.97E-06 | 6.14E-05 | 3.719526 |
| ZNF471 | -1.24439 | 0.509275 | -5.23184 | 6.05E-06 | 6.21E-05 | 3.706657 |
| VSIG1 | -1.88876 | -0.08926 | -5.22888 | 6.11E-06 | 6.26E-05 | 3.697552 |
| EGR1 | 1.809673 | -0.35923 | 5.22843 | 6.12E-06 | 6.26E-05 | 3.696162 |
| EDN3 | -3.09659 | -0.28874 | -5.22427 | 6.20E-06 | 6.33E-05 | 3.683381 |
| EPHB1 | 1.907836 | -0.0613 | 5.213573 | 6.41E-06 | 6.51E-05 | 3.650493 |
| WFIKKN1 | -1.85335 | 0.353379 | -5.21307 | 6.42E-06 | 6.52E-05 | 3.648954 |
| SAA4 | 1.65556 | 0.254872 | 5.213002 | 6.42E-06 | 6.52E-05 | 3.648737 |
| COL17A1 | -1.63486 | -0.20864 | -5.21275 | 6.43E-06 | 6.52E-05 | 3.647949 |
| PI3 | 2.792654 | -0.38036 | 5.208816 | 6.51E-06 | 6.58E-05 | 3.635875 |
| AKR1B10 | -1.18493 | -0.22665 | -5.20767 | 6.53E-06 | 6.60E-05 | 3.632339 |
| NOS2 | 3.300667 | -0.50451 | 5.207016 | 6.55E-06 | 6.61E-05 | 3.630342 |
| NEUROD2 | 1.179478 | -0.54325 | 5.206434 | 6.56E-06 | 6.62E-05 | 3.628555 |
| CEACAM19 | -1.42738 | 0.565111 | -5.20559 | 6.58E-06 | 6.64E-05 | 3.625973 |
| LOC645249 | 1.308524 | -0.36985 | 5.203538 | 6.62E-06 | 6.67E-05 | 3.619657 |
| PLEK | 1.545694 | 0.176037 | 5.202295 | 6.64E-06 | 6.69E-05 | 3.615838 |
| RHOBTB3 | 1.675646 | -0.60619 | 5.199917 | 6.69E-06 | 6.73E-05 | 3.608534 |
| PITX2 | 1.645521 | 0.256513 | 5.196982 | 6.76E-06 | 6.78E-05 | 3.599517 |
| SH2B3 | 1.059723 | -0.16192 | 5.184415 | 7.03E-06 | 7.00E-05 | 3.560924 |
| NR1D1 | -1.99718 | 0.632595 | -5.18344 | 7.05E-06 | 7.01E-05 | 3.557935 |
| BNIPL | -1.80074 | 0.379402 | -5.17948 | 7.14E-06 | 7.07E-05 | 3.545787 |
| PILRB | -1.36613 | 0.39879 | -5.17279 | 7.30E-06 | 7.21E-05 | 3.525247 |
| FANK1 | -1.04476 | 0.223029 | -5.17184 | 7.32E-06 | 7.23E-05 | 3.522327 |
| C14orf34 | -2.14057 | -0.38528 | -5.17141 | 7.33E-06 | 7.23E-05 | 3.521009 |
| ATP1B3 | 1.72813 | -0.99939 | 5.168184 | 7.40E-06 | 7.30E-05 | 3.511102 |
| GDF2 | 1.045881 | -0.53309 | 5.165912 | 7.46E-06 | 7.34E-05 | 3.504129 |
| FAM134B | 1.276636 | -0.26267 | 5.164584 | 7.49E-06 | 7.36E-05 | 3.500054 |
| PKIB | -1.73496 | -0.0222 | -5.1636 | 7.51E-06 | 7.38E-05 | 3.497025 |
| ARHGAP24 | -1.44486 | 0.29078 | -5.16325 | 7.52E-06 | 7.39E-05 | 3.495969 |
| PON2 | 1.074981 | -0.26056 | 5.162215 | 7.54E-06 | 7.40E-05 | 3.492784 |
| C16orf3 | -1.06068 | -0.01611 | -5.15537 | 7.71E-06 | 7.53E-05 | 3.471772 |
| EEF1A2 | -2.4528 | 0.621067 | -5.15466 | 7.73E-06 | 7.55E-05 | 3.469609 |
| GPR128 | 3.723501 | -1.42512 | 5.149865 | 7.84E-06 | 7.64E-05 | 3.4549 |
| ZIK1 | -1.82228 | 0.626664 | -5.14617 | 7.94E-06 | 7.72E-05 | 3.44358 |
| MGAT3 | -1.20663 | 0.085856 | -5.14471 | 7.97E-06 | 7.75E-05 | 3.4391 |
| ME1 | -1.12027 | -0.04957 | -5.13953 | 8.11E-06 | 7.86E-05 | 3.423215 |
| KBTBD12 | -1.86847 | -0.20138 | -5.13686 | 8.17E-06 | 7.92E-05 | 3.415021 |
| RPS6KA6 | -1.67153 | -0.03398 | -5.13618 | 8.19E-06 | 7.92E-05 | 3.412935 |
| PBLD | -1.18503 | -0.01982 | -5.13472 | 8.23E-06 | 7.94E-05 | 3.408461 |
| NEAT1 | -1.65659 | 0.639665 | -5.13378 | 8.25E-06 | 7.96E-05 | 3.405592 |
| CRMP1 | -1.30571 | 0.55398 | -5.13196 | 8.30E-06 | 8.00E-05 | 3.400011 |
| ANTXR2 | 1.506301 | -0.60384 | 5.126283 | 8.45E-06 | 8.12E-05 | 3.382602 |
| TGM2 | 1.671477 | -0.23383 | 5.108264 | 8.95E-06 | 8.51E-05 | 3.327398 |
| TINAG | 1.635801 | -0.16574 | 5.108076 | 8.95E-06 | 8.51E-05 | 3.32682 |
| C14orf183 | -1.77587 | -0.11394 | -5.09697 | 9.27E-06 | 8.75E-05 | 3.292813 |
| CPT1B | -1.31329 | 0.265823 | -5.09574 | 9.31E-06 | 8.77E-05 | 3.289042 |
| CD1D | 1.036887 | 0.26285 | 5.095286 | 9.32E-06 | 8.78E-05 | 3.287659 |
| CHRNB3 | 1.271038 | -0.44547 | 5.092964 | 9.39E-06 | 8.83E-05 | 3.28055 |
| CEACAM4 | 1.590177 | -0.19318 | 5.087784 | 9.55E-06 | 8.94E-05 | 3.264696 |
| MTSS1 | -1.04684 | -0.13515 | -5.08538 | 9.62E-06 | 8.99E-05 | 3.257339 |
| HOXA2 | -1.66525 | 0.657989 | -5.0833 | 9.68E-06 | 9.03E-05 | 3.25097 |
| C5orf62 | 1.179937 | -0.39711 | 5.080793 | 9.76E-06 | 9.10E-05 | 3.243304 |
| RBM20 | -1.30717 | 0.660952 | -5.07279 | 1.00E-05 | 9.29E-05 | 3.218831 |
| CBR1 | -1.40908 | -0.05873 | -5.07236 | 1.00E-05 | 9.29E-05 | 3.217495 |
| FABP6 | 2.456163 | -0.92394 | 5.070313 | 1.01E-05 | 9.33E-05 | 3.211244 |
| C3orf14 | -1.24793 | 0.32475 | -5.06894 | 1.01E-05 | 9.36E-05 | 3.207052 |
| S100A3 | 1.544276 | -0.3069 | 5.068171 | 1.02E-05 | 9.38E-05 | 3.204694 |
| RASL11B | -1.10088 | 0.161059 | -5.06763 | 1.02E-05 | 9.39E-05 | 3.203033 |
| FBP2 | -1.87551 | -0.32992 | -5.06659 | 1.02E-05 | 9.40E-05 | 3.199859 |
| SCG3 | -2.12228 | 0.390133 | -5.06394 | 1.03E-05 | 9.46E-05 | 3.191747 |
| SVOPL | 1.512925 | -0.18621 | 5.063201 | 1.03E-05 | 9.47E-05 | 3.189495 |
| SEZ6L | -1.89608 | 0.888067 | -5.06248 | 1.03E-05 | 9.48E-05 | 3.187276 |
| LAPTM5 | 1.186933 | 0.153913 | 5.062352 | 1.03E-05 | 9.48E-05 | 3.186899 |
| CHKB | -1.01092 | 0.45187 | -5.05883 | 1.05E-05 | 9.56E-05 | 3.176124 |
| LOC100292909 | -2.04368 | 1.348895 | -5.05763 | 1.05E-05 | 9.58E-05 | 3.172463 |
| TULP1 | 1.072372 | -0.65821 | 5.056933 | 1.05E-05 | 9.60E-05 | 3.170332 |
| NEURL3 | 2.488835 | -0.1276 | 5.056704 | 1.05E-05 | 9.60E-05 | 3.16963 |
| C2orf89 | 1.957323 | -0.99585 | 5.056183 | 1.05E-05 | 9.61E-05 | 3.168037 |
| SLC46A3 | 1.799083 | -0.54047 | 5.055413 | 1.06E-05 | 9.63E-05 | 3.165685 |
| IFNE | -1.6018 | 0.318861 | -5.05362 | 1.06E-05 | 9.68E-05 | 3.160192 |
| SUSD4 | -1.67746 | 0.076301 | -5.05186 | 1.07E-05 | 9.70E-05 | 3.154831 |
| GCNT4 | -1.70721 | -0.1647 | -5.05185 | 1.07E-05 | 9.70E-05 | 3.154782 |
| TMEM196 | -1.7961 | 1.011534 | -5.04898 | 1.08E-05 | 9.78E-05 | 3.146033 |
| SPP1 | 2.340381 | 0.298792 | 5.047712 | 1.08E-05 | 9.82E-05 | 3.142148 |
| TSPAN15 | -1.4318 | 0.132523 | -5.04523 | 1.09E-05 | 9.86E-05 | 3.134561 |
| PID1 | -1.27856 | 0.076058 | -5.04503 | 1.09E-05 | 9.86E-05 | 3.133944 |
| NEU4 | 1.7205 | -0.95556 | 5.044241 | 1.10E-05 | 9.88E-05 | 3.131539 |
| SNAP25 | -1.83312 | 0.592833 | -5.04101 | 1.11E-05 | 9.95E-05 | 3.121667 |
| DRP2 | 1.00092 | -0.63073 | 5.038875 | 1.11E-05 | 0.0001 | 3.115144 |
| HK3 | 1.256262 | -0.1067 | 5.037468 | 1.12E-05 | 0.000101 | 3.110847 |
| CXCR1 | 2.294559 | 0.010538 | 5.034342 | 1.13E-05 | 0.000101 | 3.101298 |
| AKR7A3 | -1.07907 | -0.07421 | -5.03426 | 1.13E-05 | 0.000101 | 3.101046 |
| ZNF595 | -1.12606 | 0.027788 | -5.0338 | 1.13E-05 | 0.000101 | 3.099634 |
| C1orf81 | -1.32072 | 0.33569 | -5.0283 | 1.15E-05 | 0.000103 | 3.082853 |
| NIM1 | -1.50468 | 0.724476 | -5.02805 | 1.15E-05 | 0.000103 | 3.082074 |
| RGL3 | -1.26134 | 0.648423 | -5.02733 | 1.16E-05 | 0.000103 | 3.079878 |
| GOLM1 | -1.16515 | 0.353425 | -5.02244 | 1.17E-05 | 0.000104 | 3.064941 |
| LEPREL1 | -1.93629 | 0.008584 | -5.01891 | 1.19E-05 | 0.000105 | 3.054166 |
| NR6A1 | 1.113854 | -0.1587 | 5.014234 | 1.20E-05 | 0.000107 | 3.039905 |
| ASS1 | 1.194643 | -0.49544 | 5.012597 | 1.21E-05 | 0.000107 | 3.034908 |
| AIM2 | 2.374619 | 0.731013 | 5.007853 | 1.23E-05 | 0.000108 | 3.020432 |
| AADAT | -1.26391 | -0.02904 | -5.00783 | 1.23E-05 | 0.000108 | 3.020369 |
| FAM74A1 | 1.046163 | -0.27122 | 5.002248 | 1.25E-05 | 0.00011 | 3.003332 |
| LOC100508226 | -2.01047 | 0.589725 | -5.00148 | 1.25E-05 | 0.00011 | 3.000987 |
| CPE | -1.7509 | 0.533766 | -4.99981 | 1.26E-05 | 0.00011 | 2.995885 |
| THBS2 | 2.424074 | 0.055421 | 4.998311 | 1.27E-05 | 0.000111 | 2.991322 |
| OPN1SW | 1.222562 | -0.22808 | 4.998159 | 1.27E-05 | 0.000111 | 2.99086 |
| LHX2 | 1.247051 | -0.05283 | 4.996849 | 1.27E-05 | 0.000111 | 2.986863 |
| HCK | 1.260395 | 0.079571 | 4.995491 | 1.28E-05 | 0.000112 | 2.982721 |
| RDH16 | 1.105897 | -0.11829 | 4.994725 | 1.28E-05 | 0.000112 | 2.980386 |
| FLJ39653 | -1.13395 | 0.459938 | -4.99329 | 1.29E-05 | 0.000112 | 2.976009 |
| GREB1L | -1.34338 | 0.416788 | -4.99145 | 1.29E-05 | 0.000113 | 2.970388 |
| NS3BP | -1.33727 | 0.305422 | -4.99055 | 1.30E-05 | 0.000113 | 2.967665 |
| CSAG1 | 2.337284 | 0.184849 | 4.989728 | 1.30E-05 | 0.000113 | 2.965147 |
| SLC7A11 | -1.31949 | -0.09932 | -4.98806 | 1.31E-05 | 0.000113 | 2.960067 |
| DSC2 | -1.2939 | -0.04102 | -4.98284 | 1.33E-05 | 0.000115 | 2.944163 |
| LY96 | 1.435065 | 0.410051 | 4.980062 | 1.34E-05 | 0.000116 | 2.935681 |
| TMEM65 | 1.030523 | -0.14653 | 4.974536 | 1.36E-05 | 0.000117 | 2.918841 |
| TPRXL | 1.997877 | -0.31377 | 4.973463 | 1.37E-05 | 0.000118 | 2.915572 |
| PHOSPHO2-KLHL23 | 1.709091 | -0.55772 | 4.972112 | 1.38E-05 | 0.000118 | 2.911456 |
| G0S2 | 2.07828 | -0.07292 | 4.971804 | 1.38E-05 | 0.000118 | 2.910517 |
| LIN7A | 1.262719 | 0.262737 | 4.97108 | 1.38E-05 | 0.000118 | 2.908311 |
| GDPD1 | -1.03125 | 0.165958 | -4.96993 | 1.38E-05 | 0.000118 | 2.904796 |
| CIDECP | -1.28449 | -0.0539 | -4.96906 | 1.39E-05 | 0.000119 | 2.902163 |
| RNF182 | 1.137863 | -0.08929 | 4.964171 | 1.41E-05 | 0.00012 | 2.887263 |
| SLC16A2 | 1.129572 | -0.18337 | 4.959901 | 1.43E-05 | 0.000122 | 2.87426 |
| PRAME | 1.961495 | 0.297342 | 4.958947 | 1.43E-05 | 0.000122 | 2.871355 |
| SLC7A4 | -1.31305 | 0.003735 | -4.95549 | 1.45E-05 | 0.000123 | 2.860833 |
| TRIB3 | 1.126325 | 0.405491 | 4.953797 | 1.46E-05 | 0.000124 | 2.855673 |
| LOC100652876 | -1.22183 | 0.093654 | -4.95167 | 1.47E-05 | 0.000124 | 2.849203 |
| DUSP6 | 1.104327 | -0.23685 | 4.950001 | 1.47E-05 | 0.000125 | 2.844117 |
| UNC93A | 1.553734 | -0.461 | 4.949519 | 1.48E-05 | 0.000125 | 2.84265 |
| DUSP26 | -1.64003 | 0.560796 | -4.94866 | 1.48E-05 | 0.000125 | 2.84004 |
| AK8 | -1.1301 | 0.367679 | -4.9481 | 1.48E-05 | 0.000125 | 2.838324 |
| CYP26B1 | 1.141696 | -0.24222 | 4.946592 | 1.49E-05 | 0.000126 | 2.833741 |
| KCNJ2 | 1.312891 | -0.03837 | 4.946547 | 1.49E-05 | 0.000126 | 2.833605 |
| RGAG1 | 1.046164 | 0.082986 | 4.946191 | 1.49E-05 | 0.000126 | 2.832519 |
| C1QB | 1.525235 | -0.01956 | 4.945037 | 1.50E-05 | 0.000126 | 2.829008 |
| GLI2 | -1.34311 | 0.341948 | -4.94343 | 1.51E-05 | 0.000127 | 2.824106 |
| CLEC4D | 1.671827 | 0.285275 | 4.935779 | 1.54E-05 | 0.000129 | 2.800837 |
| PDE4C | -1.01739 | 0.247113 | -4.93389 | 1.55E-05 | 0.00013 | 2.795096 |
| ZNF223 | -1.03047 | 0.046307 | -4.93155 | 1.56E-05 | 0.00013 | 2.787983 |
| ASGR1 | 1.165848 | 0.10008 | 4.929165 | 1.57E-05 | 0.000131 | 2.780721 |
| TNFAIP8L2 | 1.23816 | 0.225864 | 4.927979 | 1.58E-05 | 0.000132 | 2.777112 |
| ARG2 | 1.139398 | -0.37307 | 4.923402 | 1.60E-05 | 0.000133 | 2.763195 |
| CSAG2 | 2.341848 | 0.527033 | 4.922209 | 1.61E-05 | 0.000134 | 2.759566 |
| SLC2A14 | 1.053744 | 0.36286 | 4.922102 | 1.61E-05 | 0.000134 | 2.759244 |
| HERC2P2 | -1.12407 | 0.336604 | -4.91877 | 1.63E-05 | 0.000135 | 2.749122 |
| DOK3 | 1.047449 | 0.212221 | 4.914106 | 1.65E-05 | 0.000136 | 2.734934 |
| CR1 | 1.041292 | -0.01205 | 4.91236 | 1.66E-05 | 0.000137 | 2.729628 |
| LOC100130428 | -1.64776 | 0.83497 | -4.90905 | 1.68E-05 | 0.000138 | 2.719566 |
| LRRC31 | 1.523526 | -0.58811 | 4.908286 | 1.68E-05 | 0.000138 | 2.717248 |
| IRS1 | -1.08864 | 0.0847 | -4.90304 | 1.71E-05 | 0.00014 | 2.701322 |
| CMTM2 | 2.975748 | 0.385813 | 4.900073 | 1.73E-05 | 0.000141 | 2.692298 |
| HLA-DPA1 | 1.370874 | 0.039937 | 4.898729 | 1.73E-05 | 0.000142 | 2.688217 |
| SERPINA1 | 1.737464 | -0.16552 | 4.898068 | 1.74E-05 | 0.000142 | 2.686209 |
| CALY | -1.52546 | 0.384992 | -4.89769 | 1.74E-05 | 0.000142 | 2.685061 |
| ADAMDEC1 | 2.156553 | -0.17619 | 4.893936 | 1.76E-05 | 0.000143 | 2.673658 |
| BTN1A1 | 1.275887 | -0.24853 | 4.891227 | 1.77E-05 | 0.000144 | 2.665435 |
| IL28B | 1.729585 | 0.478577 | 4.883969 | 1.81E-05 | 0.000147 | 2.643402 |
| CA11 | -1.17849 | 0.552098 | -4.88327 | 1.82E-05 | 0.000147 | 2.641267 |
| HOXB5 | 1.748765 | -0.49072 | 4.87924 | 1.84E-05 | 0.000149 | 2.629048 |
| P2RY6 | 1.339879 | 0.111435 | 4.877 | 1.85E-05 | 0.00015 | 2.622251 |
| PGLYRP1 | 1.3067 | -0.12423 | 4.867105 | 1.91E-05 | 0.000154 | 2.592235 |
| HS3ST2 | 1.747406 | 0.412391 | 4.862165 | 1.94E-05 | 0.000155 | 2.577254 |
| DEPDC7 | 1.628303 | -0.4652 | 4.861205 | 1.95E-05 | 0.000156 | 2.574343 |
| FAM49A | 1.208602 | 0.308224 | 4.856349 | 1.98E-05 | 0.000158 | 2.55962 |
| PLK2 | 1.02402 | -0.08831 | 4.852301 | 2.00E-05 | 0.000159 | 2.547351 |
| GAPT | 1.407614 | 0.269179 | 4.850189 | 2.02E-05 | 0.00016 | 2.540951 |
| FAM3D | 1.387308 | -0.41017 | 4.845791 | 2.05E-05 | 0.000162 | 2.527624 |
| GABRD | 1.003791 | -0.2894 | 4.843836 | 2.06E-05 | 0.000163 | 2.5217 |
| ADHFE1 | -2.40496 | 0.7295 | -4.84174 | 2.07E-05 | 0.000163 | 2.515339 |
| FNDC1 | -2.29018 | 1.135906 | -4.83535 | 2.11E-05 | 0.000166 | 2.495983 |
| CHI3L1 | 2.586563 | 0.103343 | 4.833837 | 2.12E-05 | 0.000167 | 2.491414 |
| FLRT3 | 1.059925 | -0.21081 | 4.830077 | 2.15E-05 | 0.000169 | 2.480031 |
| WNT11 | 1.047881 | -0.04101 | 4.829157 | 2.16E-05 | 0.000169 | 2.477245 |
| MAGEA11 | 1.311443 | 0.222209 | 4.826165 | 2.18E-05 | 0.00017 | 2.468188 |
| MIR7-3HG | -1.6875 | 0.567365 | -4.82562 | 2.18E-05 | 0.00017 | 2.466525 |
| FAM153B | -1.7823 | 0.582737 | -4.82371 | 2.19E-05 | 0.000171 | 2.460747 |
| PKP1 | 1.031658 | -0.26501 | 4.820289 | 2.22E-05 | 0.000173 | 2.450405 |
| ANKS4B | -1.19627 | -0.24249 | -4.81931 | 2.22E-05 | 0.000173 | 2.447452 |
| MMP28 | -1.07893 | -0.13226 | -4.81486 | 2.25E-05 | 0.000176 | 2.433989 |
| TMEM26 | 1.381682 | 0.212972 | 4.812196 | 2.27E-05 | 0.000177 | 2.425918 |
| LOC100272216 | -1.67472 | 0.566246 | -4.81204 | 2.27E-05 | 0.000177 | 2.425438 |
| ATG16L2 | -1.00151 | 0.421437 | -4.80736 | 2.31E-05 | 0.000179 | 2.41129 |
| SOX30 | 1.05075 | -0.37724 | 4.805245 | 2.32E-05 | 0.00018 | 2.404895 |
| OASL | -1.28813 | 0.02711 | -4.80464 | 2.33E-05 | 0.00018 | 2.403066 |
| SRGN | 1.370999 | 0.396466 | 4.801289 | 2.35E-05 | 0.000182 | 2.392936 |
| ISPD | -1.08769 | -0.02259 | -4.80009 | 2.36E-05 | 0.000182 | 2.38931 |
| LINC00261 | -1.38663 | 0.163175 | -4.79821 | 2.37E-05 | 0.000183 | 2.383623 |
| GABRB2 | 1.019632 | -0.4027 | 4.79602 | 2.39E-05 | 0.000184 | 2.377009 |
| MPPED2 | -1.1188 | 0.514923 | -4.79243 | 2.42E-05 | 0.000185 | 2.36616 |
| SVOP | -2.3527 | 0.238082 | -4.78966 | 2.44E-05 | 0.000187 | 2.357785 |
| STARD4 | -1.12877 | -0.00216 | -4.78574 | 2.47E-05 | 0.000189 | 2.345939 |
| SDR16C5 | -1.22686 | -0.15549 | -4.78466 | 2.48E-05 | 0.000189 | 2.342674 |
| ATP6V0D2 | 1.235294 | 0.177669 | 4.781754 | 2.50E-05 | 0.000191 | 2.333905 |
| COL22A1 | -1.51885 | 0.568985 | -4.78001 | 2.51E-05 | 0.000191 | 2.328647 |
| LOC100506310 | -1.33603 | 0.198434 | -4.77426 | 2.56E-05 | 0.000194 | 2.311271 |
| CLEC18B | -1.21409 | 0.083826 | -4.77102 | 2.59E-05 | 0.000196 | 2.301491 |
| MST1P9 | -2.29267 | 0.147044 | -4.76767 | 2.61E-05 | 0.000198 | 2.291369 |
| SAMSN1 | 1.032108 | 0.174079 | 4.761496 | 2.66E-05 | 0.000201 | 2.272751 |
| EFNA5 | -1.13721 | 0.006146 | -4.76117 | 2.67E-05 | 0.000201 | 2.27176 |
| LAIR1 | 1.040404 | 0.149206 | 4.760713 | 2.67E-05 | 0.000201 | 2.270386 |
| SEMA3A | 1.268741 | -0.03529 | 4.76001 | 2.68E-05 | 0.000201 | 2.268266 |
| PHYHD1 | -1.41614 | 0.103203 | -4.75891 | 2.69E-05 | 0.000202 | 2.264944 |
| MNDA | 2.217172 | 0.231619 | 4.756403 | 2.71E-05 | 0.000203 | 2.257387 |
| LILRA5 | 1.268827 | -0.2563 | 4.752618 | 2.74E-05 | 0.000205 | 2.245969 |
| CCNJL | 1.030211 | -0.16296 | 4.752458 | 2.74E-05 | 0.000205 | 2.245489 |
| SERPINB9 | 1.248188 | 0.154307 | 4.746964 | 2.79E-05 | 0.000208 | 2.22892 |
| C8orf68 | 1.047133 | -0.18008 | 4.746446 | 2.79E-05 | 0.000208 | 2.227359 |
| RNASE1 | -1.17374 | 0.112244 | -4.74587 | 2.80E-05 | 0.000208 | 2.225623 |
| PCDHB8 | 1.260955 | -0.12654 | 4.745488 | 2.80E-05 | 0.000208 | 2.224471 |
| ICAM1 | 1.316112 | 0.229479 | 4.742419 | 2.83E-05 | 0.00021 | 2.21522 |
| PTPRR | -1.57401 | 0.063765 | -4.73868 | 2.86E-05 | 0.000212 | 2.20394 |
| AHSG | 1.012888 | -0.35241 | 4.735792 | 2.89E-05 | 0.000214 | 2.195249 |
| HAMP | 1.171214 | -0.53307 | 4.732856 | 2.91E-05 | 0.000215 | 2.186404 |
| SCNN1B | -1.70643 | 0.089766 | -4.73276 | 2.91E-05 | 0.000215 | 2.186117 |
| MARVELD3 | 1.694527 | -0.74236 | 4.729214 | 2.95E-05 | 0.000217 | 2.175434 |
| C21orf90 | 2.570151 | -1.37049 | 4.726256 | 2.97E-05 | 0.000219 | 2.166524 |
| OSGIN1 | -1.04463 | -0.04307 | -4.7255 | 2.98E-05 | 0.000219 | 2.164257 |
| MPV17L | -1.04872 | 0.007584 | -4.71549 | 3.08E-05 | 0.000225 | 2.13411 |
| RETSAT | -1.35524 | 0.161615 | -4.7101 | 3.13E-05 | 0.000228 | 2.117888 |
| PCSK1 | -2.34474 | 0.769676 | -4.70304 | 3.20E-05 | 0.000232 | 2.09666 |
| C7orf54 | -1.5 | 0.564186 | -4.6979 | 3.25E-05 | 0.000235 | 2.081188 |
| RNASE6 | 1.246455 | 0.233956 | 4.696975 | 3.26E-05 | 0.000235 | 2.078413 |
| PXDNL | 1.332087 | 0.157235 | 4.693978 | 3.29E-05 | 0.000237 | 2.069402 |
| FLJ42393 | -1.74755 | 0.649614 | -4.68998 | 3.33E-05 | 0.000239 | 2.057375 |
| CAHM | -1.0163 | 0.718753 | -4.682 | 3.41E-05 | 0.000244 | 2.033396 |
| TFPI | 1.270035 | -0.37913 | 4.680763 | 3.43E-05 | 0.000245 | 2.029691 |
| RNU12 | 1.537788 | -0.61637 | 4.680501 | 3.43E-05 | 0.000245 | 2.028903 |
| DMRTA1 | -1.52501 | -0.20301 | -4.67961 | 3.44E-05 | 0.000246 | 2.026232 |
| CD55 | 1.542714 | -0.56192 | 4.676948 | 3.47E-05 | 0.000247 | 2.018229 |
| SH3TC2 | 1.064331 | -0.05249 | 4.671969 | 3.52E-05 | 0.00025 | 2.003278 |
| FLJ36840 | -1.49319 | 0.409736 | -4.66799 | 3.57E-05 | 0.000253 | 1.991341 |
| HSPA2 | -1.11249 | 0.216655 | -4.66789 | 3.57E-05 | 0.000253 | 1.991044 |
| PROKR2 | -1.08593 | 0.073891 | -4.66788 | 3.57E-05 | 0.000253 | 1.991005 |
| TM6SF2 | -1.81084 | -0.161 | -4.66769 | 3.57E-05 | 0.000253 | 1.990443 |
| ATP6V1C2 | 1.158762 | -0.07773 | 4.664954 | 3.60E-05 | 0.000255 | 1.98222 |
| HVCN1 | 1.100991 | 0.307957 | 4.664506 | 3.60E-05 | 0.000255 | 1.980875 |
| SAP30 | -1.29916 | 0.330624 | -4.65893 | 3.67E-05 | 0.000258 | 1.964133 |
| ZNF606 | -1.10314 | -0.15524 | -4.65617 | 3.70E-05 | 0.00026 | 1.95586 |
| GHRLOS2 | -1.20404 | -0.10478 | -4.65116 | 3.76E-05 | 0.000264 | 1.940842 |
| SEMA6A | 1.284859 | -0.15901 | 4.646969 | 3.81E-05 | 0.000267 | 1.928269 |
| PNPLA7 | -1.18815 | 0.448763 | -4.64559 | 3.82E-05 | 0.000267 | 1.924147 |
| PYDC1 | 1.103643 | -0.26656 | 4.642065 | 3.87E-05 | 0.00027 | 1.913567 |
| FLJ32255 | 1.292396 | -0.31032 | 4.640245 | 3.89E-05 | 0.000271 | 1.908112 |
| LOC100147773 | -1.04613 | 0.365494 | -4.63533 | 3.95E-05 | 0.000275 | 1.893388 |
| PAK3 | -1.40049 | 0.793024 | -4.63495 | 3.95E-05 | 0.000275 | 1.892253 |
| IL23A | 1.134154 | -0.01733 | 4.634157 | 3.96E-05 | 0.000275 | 1.889869 |
| LOC344887 | -1.48944 | 0.447869 | -4.63335 | 3.97E-05 | 0.000276 | 1.887463 |
| DOK5 | 1.325994 | -0.3691 | 4.626861 | 4.05E-05 | 0.00028 | 1.868017 |
| RGS2 | 1.630244 | -0.09453 | 4.626503 | 4.06E-05 | 0.00028 | 1.866945 |
| CUBN | 1.043478 | -0.24739 | 4.625166 | 4.07E-05 | 0.000281 | 1.862942 |
| NFE4 | 1.579987 | -0.18387 | 4.622388 | 4.11E-05 | 0.000283 | 1.854622 |
| NKX2-2 | -1.72999 | 0.465284 | -4.62173 | 4.12E-05 | 0.000284 | 1.852661 |
| VNN2 | 1.889166 | 0.357646 | 4.618737 | 4.16E-05 | 0.000286 | 1.843693 |
| XK | -1.05577 | -0.06245 | -4.61819 | 4.16E-05 | 0.000287 | 1.842065 |
| SPAM1 | 1.128348 | -0.17937 | 4.617037 | 4.18E-05 | 0.000287 | 1.838605 |
| CA1 | 3.532924 | 0.446721 | 4.616573 | 4.18E-05 | 0.000288 | 1.837218 |
| NEUROD1 | -2.23922 | 0.644658 | -4.61637 | 4.19E-05 | 0.000288 | 1.83662 |
| CRYGB | -1.44841 | 0.721571 | -4.61615 | 4.19E-05 | 0.000288 | 1.835936 |
| ZFHX4 | 1.357378 | 0.132932 | 4.614976 | 4.20E-05 | 0.000289 | 1.832437 |
| PADI3 | 1.415491 | -0.16216 | 4.614096 | 4.22E-05 | 0.000289 | 1.829803 |
| LY6G6F | 1.178081 | -0.49403 | 4.613453 | 4.22E-05 | 0.00029 | 1.827881 |
| BZRAP1 | -1.32959 | 0.468877 | -4.61254 | 4.24E-05 | 0.00029 | 1.825162 |
| LOC100507165 | 1.695837 | 0.119957 | 4.610902 | 4.26E-05 | 0.000291 | 1.820248 |
| CLDN14 | 1.550187 | -0.48699 | 4.610799 | 4.26E-05 | 0.000291 | 1.819939 |
| KRT6B | 2.912844 | 0.36208 | 4.608222 | 4.29E-05 | 0.000293 | 1.812231 |
| LOC100128180 | -1.28251 | 0.099799 | -4.6014 | 4.39E-05 | 0.000298 | 1.791823 |
| TNFAIP2 | 1.336299 | 0.084575 | 4.599699 | 4.41E-05 | 0.000299 | 1.786741 |
| VN1R1 | -1.14207 | 0.014712 | -4.59817 | 4.43E-05 | 0.000301 | 1.782177 |
| CD86 | 1.83728 | 0.015335 | 4.593353 | 4.50E-05 | 0.000304 | 1.767772 |
| KREMEN2 | 1.066283 | -0.0437 | 4.592588 | 4.51E-05 | 0.000305 | 1.765487 |
| STK32B | -1.41142 | 0.510849 | -4.59142 | 4.52E-05 | 0.000305 | 1.761986 |
| MYADML2 | 1.398019 | -0.2038 | 4.590861 | 4.53E-05 | 0.000306 | 1.760325 |
| OLFM4 | 4.545752 | -2.67995 | 4.588609 | 4.56E-05 | 0.000307 | 1.753596 |
| KCNF1 | 1.537174 | 0.123741 | 4.587983 | 4.57E-05 | 0.000307 | 1.751726 |
| CSF2RA | 1.132425 | 0.201964 | 4.586534 | 4.59E-05 | 0.000309 | 1.747397 |
| FUT9 | -1.39165 | 0.144762 | -4.58477 | 4.62E-05 | 0.00031 | 1.742115 |
| ZNF169 | -1.2305 | 0.029245 | -4.58409 | 4.63E-05 | 0.000311 | 1.740098 |
| LOC100128816 | -1.10833 | 0.12869 | -4.58044 | 4.68E-05 | 0.000314 | 1.729187 |
| LOC728392 | -1.36842 | 0.82018 | -4.56955 | 4.84E-05 | 0.000323 | 1.69669 |
| APOBEC3A | 2.069657 | 0.969278 | 4.564084 | 4.92E-05 | 0.000328 | 1.680378 |
| ZNF512B | -1.00674 | -0.00556 | -4.56386 | 4.93E-05 | 0.000328 | 1.679717 |
| LOC100128398 | -1.47142 | 0.768652 | -4.5629 | 4.94E-05 | 0.000328 | 1.676857 |
| CHRNG | 1.259043 | -0.1978 | 4.555832 | 5.05E-05 | 0.000334 | 1.655768 |
| SNORA70 | -1.05434 | 0.359671 | -4.55581 | 5.05E-05 | 0.000334 | 1.655709 |
| DSCAML1 | -1.58361 | 0.398955 | -4.55449 | 5.07E-05 | 0.000335 | 1.651756 |
| TFEC | 1.253536 | 0.537162 | 4.553568 | 5.09E-05 | 0.000335 | 1.649018 |
| CLDN3 | 2.349625 | -1.30116 | 4.552705 | 5.10E-05 | 0.000336 | 1.646444 |
| LOC283663 | 1.140252 | -0.32664 | 4.547168 | 5.19E-05 | 0.000341 | 1.62994 |
| TMEM176B | 1.185237 | -0.43585 | 4.545258 | 5.22E-05 | 0.000342 | 1.62425 |
| HORMAD1 | 1.211543 | 0.328859 | 4.541094 | 5.29E-05 | 0.000346 | 1.611845 |
| AMDHD1 | 1.164004 | -0.06873 | 4.540567 | 5.30E-05 | 0.000346 | 1.610274 |
| LYPD6B | -2.00222 | -0.05192 | -4.5405 | 5.30E-05 | 0.000346 | 1.610072 |
| ACCS | -1.22216 | 0.081059 | -4.53581 | 5.37E-05 | 0.00035 | 1.5961 |
| KLK10 | 2.536492 | -0.22417 | 4.535018 | 5.39E-05 | 0.000351 | 1.593749 |
| TMEM132A | 1.065534 | 0.072553 | 4.533678 | 5.41E-05 | 0.000352 | 1.589758 |
| PLXNC1 | 1.110467 | -0.07312 | 4.533565 | 5.41E-05 | 0.000352 | 1.589422 |
| PHEX | 1.162524 | -0.32599 | 4.532747 | 5.43E-05 | 0.000352 | 1.586985 |
| GTSF1 | 1.788518 | 0.752148 | 4.532557 | 5.43E-05 | 0.000352 | 1.586421 |
| ITM2A | 1.042238 | 0.087123 | 4.526221 | 5.54E-05 | 0.000358 | 1.567562 |
| C20orf194 | -1.0352 | 0.008134 | -4.52569 | 5.55E-05 | 0.000358 | 1.565984 |
| SFTPD | -1.1429 | 0.548626 | -4.52569 | 5.55E-05 | 0.000358 | 1.565977 |
| DPP4 | 1.956099 | -0.90451 | 4.520966 | 5.63E-05 | 0.000363 | 1.551924 |
| MS4A8B | -1.52293 | -0.1156 | -4.52081 | 5.63E-05 | 0.000363 | 1.551474 |
| CDKN2A | 1.674162 | -0.51578 | 4.51756 | 5.69E-05 | 0.000365 | 1.541794 |
| ISX | 2.828977 | -0.51326 | 4.516492 | 5.71E-05 | 0.000366 | 1.538616 |
| LYPD6 | -1.70626 | 0.01905 | -4.51441 | 5.74E-05 | 0.000368 | 1.532436 |
| IL17C | 1.26031 | -0.52897 | 4.513281 | 5.76E-05 | 0.000369 | 1.529068 |
| ADAMTS4 | 1.762249 | -0.11188 | 4.509401 | 5.83E-05 | 0.000373 | 1.517534 |
| VPREB3 | 1.256274 | -0.16961 | 4.506712 | 5.88E-05 | 0.000375 | 1.50954 |
| HMOX1 | 1.26394 | -0.37362 | 4.505105 | 5.91E-05 | 0.000377 | 1.504764 |
| NLRP12 | 1.310637 | -0.34198 | 4.503468 | 5.94E-05 | 0.000378 | 1.499901 |
| IGFBP5 | 1.149979 | -0.00851 | 4.502783 | 5.95E-05 | 0.000379 | 1.497865 |
| GUCA2B | -1.75614 | 0.337295 | -4.49976 | 6.01E-05 | 0.000381 | 1.488887 |
| IL22RA2 | 1.032196 | -0.20191 | 4.497353 | 6.05E-05 | 0.000383 | 1.481732 |
| ZNF347 | -1.2008 | 0.007759 | -4.49328 | 6.13E-05 | 0.000387 | 1.469649 |
| CLDN1 | 2.283231 | -0.94246 | 4.484081 | 6.31E-05 | 0.000396 | 1.442329 |
| VAX2 | 1.1122 | -0.04201 | 4.482684 | 6.33E-05 | 0.000398 | 1.438183 |
| CFP | 1.164901 | 0.333129 | 4.479828 | 6.39E-05 | 0.000401 | 1.429709 |
| FPR3 | 1.315276 | -0.05891 | 4.479764 | 6.39E-05 | 0.000401 | 1.429521 |
| FPR2 | 2.317099 | 0.150627 | 4.476506 | 6.46E-05 | 0.000404 | 1.419854 |
| MFAP2 | 1.293273 | 0.180061 | 4.474941 | 6.49E-05 | 0.000405 | 1.415212 |
| PIR | -1.02615 | 0.198307 | -4.47396 | 6.51E-05 | 0.000406 | 1.412289 |
| FOXD4 | 1.049557 | -0.51409 | 4.472004 | 6.55E-05 | 0.000408 | 1.406504 |
| WBP5 | -1.12319 | -0.06569 | -4.47165 | 6.55E-05 | 0.000408 | 1.40544 |
| ATP6V0A4 | 1.584111 | -0.10945 | 4.470666 | 6.57E-05 | 0.000409 | 1.402536 |
| ZNF415 | -1.26336 | 0.36868 | -4.46909 | 6.60E-05 | 0.000411 | 1.397861 |
| TTYH1 | 1.21578 | -0.43202 | 4.46853 | 6.62E-05 | 0.000411 | 1.396204 |
| IRX2 | 3.436919 | -0.57483 | 4.466485 | 6.66E-05 | 0.000414 | 1.390141 |
| CAMP | 2.058176 | 0.443333 | 4.461102 | 6.77E-05 | 0.000419 | 1.374188 |
| FAM110B | -1.16336 | 0.566513 | -4.46067 | 6.78E-05 | 0.000419 | 1.372909 |
| OR10A2 | 1.268276 | -0.64291 | 4.459809 | 6.80E-05 | 0.00042 | 1.370358 |
| POTED | 1.815819 | 0.376155 | 4.45874 | 6.82E-05 | 0.000421 | 1.36719 |
| CFC1 | 1.008143 | -0.00889 | 4.456169 | 6.87E-05 | 0.000424 | 1.359576 |
| KCNK16 | -1.40872 | 0.673618 | -4.45417 | 6.92E-05 | 0.000426 | 1.353669 |
| FCN2 | 1.194647 | 0.253894 | 4.453463 | 6.93E-05 | 0.000426 | 1.351562 |
| LILRA3 | 1.517965 | 0.26227 | 4.453398 | 6.93E-05 | 0.000426 | 1.351369 |
| DUOXA2 | 1.423599 | -0.03319 | 4.453304 | 6.93E-05 | 0.000426 | 1.351091 |
| LOC100506898 | -1.43003 | 0.566713 | -4.45088 | 6.99E-05 | 0.000429 | 1.343904 |
| LOC100507280 | -1.09841 | 0.158921 | -4.44908 | 7.02E-05 | 0.000431 | 1.33859 |
| MAGEL2 | 1.031026 | -0.50542 | 4.448943 | 7.03E-05 | 0.000431 | 1.338178 |
| LOC100170939 | -1.01625 | 0.322428 | -4.44335 | 7.15E-05 | 0.000436 | 1.321615 |
| H2AFY2 | -1.17493 | -0.18768 | -4.44313 | 7.15E-05 | 0.000436 | 1.320986 |
| KCNJ5 | -1.48832 | 0.621037 | -4.43827 | 7.26E-05 | 0.000442 | 1.306593 |
| CXXC4 | -1.3146 | 0.189062 | -4.43802 | 7.27E-05 | 0.000442 | 1.305838 |
| TM7SF4 | 1.138808 | -0.16301 | 4.437359 | 7.28E-05 | 0.000443 | 1.303896 |
| RGS9BP | 1.069244 | 0.099007 | 4.437199 | 7.29E-05 | 0.000443 | 1.303425 |
| SULT1C2 | -1.26807 | -0.17349 | -4.43659 | 7.30E-05 | 0.000443 | 1.30162 |
| SPATA8 | 1.051841 | -0.14428 | 4.433596 | 7.37E-05 | 0.000447 | 1.292769 |
| RAX | 1.243362 | -0.49925 | 4.43235 | 7.40E-05 | 0.000448 | 1.289084 |
| ZNF540 | -1.4007 | 0.667946 | -4.43164 | 7.41E-05 | 0.000449 | 1.286983 |
| RGAG4 | -1.19673 | 0.490484 | -4.43128 | 7.42E-05 | 0.000449 | 1.285933 |
| CSF2 | -1.6607 | 0.420606 | -4.4267 | 7.53E-05 | 0.000454 | 1.272392 |
| HCN3 | -1.04557 | 0.275735 | -4.42511 | 7.56E-05 | 0.000456 | 1.267674 |
| MSMO1 | -1.37014 | -0.02326 | -4.42503 | 7.56E-05 | 0.000456 | 1.267434 |
| CLDN10 | -2.79931 | -0.10028 | -4.42462 | 7.57E-05 | 0.000457 | 1.266227 |
| UBXN10 | -1.0945 | -0.14608 | -4.42313 | 7.61E-05 | 0.000459 | 1.261818 |
| LSS | -1.24119 | 0.091192 | -4.4224 | 7.63E-05 | 0.000459 | 1.259668 |
| AK7 | -1.30319 | 0.046082 | -4.41959 | 7.69E-05 | 0.000463 | 1.251377 |
| LYPD3 | 1.390123 | -0.31551 | 4.419544 | 7.69E-05 | 0.000463 | 1.251234 |
| FOSL1 | 1.681132 | -0.52061 | 4.41883 | 7.71E-05 | 0.000463 | 1.249124 |
| DPT | -1.68816 | 0.624317 | -4.41659 | 7.76E-05 | 0.000466 | 1.242509 |
| AIF1 | 1.173262 | 0.168367 | 4.412813 | 7.85E-05 | 0.00047 | 1.231353 |
| ELOVL7 | 1.435205 | -0.62574 | 4.411294 | 7.89E-05 | 0.000471 | 1.226869 |
| CLCA3P | 1.502324 | -0.1181 | 4.409933 | 7.92E-05 | 0.000473 | 1.222851 |
| ATPAF1-AS1 | -1.29658 | 0.351217 | -4.40978 | 7.93E-05 | 0.000473 | 1.222398 |
| LOC142937 | -1.56109 | 0.340101 | -4.40962 | 7.93E-05 | 0.000473 | 1.221913 |
| C11orf96 | 1.458444 | 0.025065 | 4.408754 | 7.95E-05 | 0.000474 | 1.219368 |
| ERO1LB | -1.4514 | 0.530153 | -4.40561 | 8.03E-05 | 0.000478 | 1.210088 |
| HOXA4 | -1.24113 | 0.45662 | -4.4005 | 8.16E-05 | 0.000485 | 1.195005 |
| POLR3G | 1.042503 | -0.2667 | 4.400319 | 8.16E-05 | 0.000485 | 1.194477 |
| ANXA9 | 1.532675 | -0.18657 | 4.396945 | 8.25E-05 | 0.000489 | 1.184523 |
| MYOM3 | 1.412132 | -0.35533 | 4.396499 | 8.26E-05 | 0.000489 | 1.183207 |
| KRT16 | 2.007978 | -0.74984 | 4.392924 | 8.35E-05 | 0.000494 | 1.172667 |
| FAP | 2.073054 | -0.18797 | 4.39169 | 8.38E-05 | 0.000495 | 1.169026 |
| PTPRN | -1.18702 | 0.506574 | -4.39129 | 8.39E-05 | 0.000496 | 1.167842 |
| ENTPD3 | -1.37299 | -0.16121 | -4.3827 | 8.61E-05 | 0.000507 | 1.142524 |
| LOC100130193 | -1.19247 | 0.57814 | -4.38265 | 8.62E-05 | 0.000507 | 1.142372 |
| TPSAB1 | 1.181613 | 0.156743 | 4.382624 | 8.62E-05 | 0.000507 | 1.142308 |
| ABCG1 | -1.07041 | 0.164871 | -4.37823 | 8.73E-05 | 0.000514 | 1.129351 |
| MAP6 | -1.10962 | 0.614453 | -4.37551 | 8.81E-05 | 0.000517 | 1.121354 |
| MSC | 1.138266 | 0.13429 | 4.37473 | 8.83E-05 | 0.000517 | 1.119054 |
| SAA2 | 1.282879 | 0.014222 | 4.370707 | 8.94E-05 | 0.000523 | 1.107211 |
| RIC3 | -1.09605 | 0.498645 | -4.36578 | 9.07E-05 | 0.000529 | 1.092698 |
| MCOLN3 | -1.02507 | 0.122422 | -4.36564 | 9.08E-05 | 0.000529 | 1.09229 |
| RASSF10 | -1.26453 | 0.067782 | -4.36543 | 9.08E-05 | 0.00053 | 1.09169 |
| LOC100506262 | -1.02 | -0.06573 | -4.36485 | 9.10E-05 | 0.00053 | 1.089988 |
| LIPC | 1.613743 | -0.06874 | 4.364789 | 9.10E-05 | 0.00053 | 1.089793 |
| LOC100652791 | -1.01457 | 0.379722 | -4.36439 | 9.11E-05 | 0.000531 | 1.088609 |
| OXGR1 | 1.131858 | 0.329612 | 4.358012 | 9.29E-05 | 0.000538 | 1.06986 |
| SLC29A1 | -1.01491 | 0.076759 | -4.35666 | 9.33E-05 | 0.00054 | 1.065889 |
| TFF3 | 2.142029 | -0.89493 | 4.354599 | 9.39E-05 | 0.000543 | 1.059824 |
| ACVR1C | -1.04547 | 0.117716 | -4.35107 | 9.49E-05 | 0.000547 | 1.04946 |
| RIMS3 | -1.24934 | 0.323365 | -4.34818 | 9.58E-05 | 0.000552 | 1.040966 |
| CA2 | -1.09074 | -0.20667 | -4.34515 | 9.67E-05 | 0.000556 | 1.03205 |
| C1QTNF1 | 1.360127 | 0.083775 | 4.341001 | 9.79E-05 | 0.000561 | 1.019863 |
| SPI1 | 1.321174 | -0.12885 | 4.337287 | 9.90E-05 | 0.000566 | 1.008956 |
| MAGEA1 | 2.082681 | 0.776178 | 4.336101 | 9.94E-05 | 0.000568 | 1.005475 |
| FAM151A | 1.201529 | -0.10436 | 4.334696 | 9.98E-05 | 0.00057 | 1.00135 |
| RDH12 | -2.01421 | 0.169773 | -4.33466 | 9.98E-05 | 0.00057 | 1.001245 |
| PSCA | -2.41237 | 0.001994 | -4.3335 | 0.0001 | 0.000572 | 0.997846 |
| SEMG1 | 2.148544 | -0.22439 | 4.333347 | 0.0001 | 0.000572 | 0.99739 |
| WNT5A | 1.612837 | -0.07325 | 4.330926 | 0.000101 | 0.000575 | 0.990282 |
| SLC23A3 | -1.22979 | 0.142021 | -4.32637 | 0.000102 | 0.000581 | 0.976914 |
| CHP2 | 5.098121 | -3.07132 | 4.321849 | 0.000104 | 0.000588 | 0.963653 |
| GBP5 | 1.352032 | 0.479447 | 4.321801 | 0.000104 | 0.000588 | 0.963514 |
| LOC84856 | 1.123655 | -0.08495 | 4.307829 | 0.000108 | 0.000609 | 0.922559 |
| MS4A7 | 1.226228 | 0.007578 | 4.304734 | 0.000109 | 0.000614 | 0.913492 |
| SYCE3 | 1.03207 | -0.20788 | 4.303091 | 0.00011 | 0.000616 | 0.908681 |
| IL11 | 1.145387 | 0.340868 | 4.30222 | 0.00011 | 0.000618 | 0.90613 |
| IGF1 | 1.020723 | 0.171797 | 4.300383 | 0.000111 | 0.000621 | 0.900752 |
| C8orf51 | 1.150994 | -0.07995 | 4.296594 | 0.000112 | 0.000626 | 0.88966 |
| PYGL | 1.085277 | 0.273544 | 4.296555 | 0.000112 | 0.000626 | 0.889547 |
| KCNMB2 | -1.94153 | 0.129287 | -4.29263 | 0.000113 | 0.000632 | 0.878052 |
| LOC100132356 | -1.21193 | 0.217164 | -4.29133 | 0.000114 | 0.000634 | 0.874257 |
| CNDP1 | 1.99809 | -0.54998 | 4.290284 | 0.000114 | 0.000636 | 0.871195 |
| CPNE8 | -1.14854 | 0.288838 | -4.28932 | 0.000115 | 0.000637 | 0.868382 |
| CCL20 | 2.36029 | -0.52381 | 4.284829 | 0.000116 | 0.000644 | 0.855242 |
| ZNF345 | -1.01007 | 0.16362 | -4.28298 | 0.000117 | 0.000646 | 0.849822 |
| GPR158 | 1.455193 | -0.10531 | 4.282756 | 0.000117 | 0.000646 | 0.849182 |
| FCGBP | -1.39848 | 0.039727 | -4.27978 | 0.000118 | 0.000651 | 0.84049 |
| SPG20 | -1.0644 | 0.035004 | -4.27723 | 0.000119 | 0.000656 | 0.833026 |
| ZNF300 | -1.11708 | 0.035576 | -4.27551 | 0.00012 | 0.000658 | 0.828005 |
| LDLRAD1 | -1.42308 | 0.150606 | -4.27396 | 0.00012 | 0.000661 | 0.823477 |
| CXorf48 | 1.291589 | -0.34941 | 4.268861 | 0.000122 | 0.000669 | 0.80858 |
| PEG3-AS1 | -1.26033 | 0.567955 | -4.26767 | 0.000122 | 0.000671 | 0.805099 |
| GRIA3 | -1.12599 | 0.408749 | -4.26475 | 0.000124 | 0.000675 | 0.796565 |
| PAX9 | 1.621559 | 0.063507 | 4.26125 | 0.000125 | 0.000681 | 0.786361 |
| GRAMD1B | -1.22706 | 0.210595 | -4.25902 | 0.000126 | 0.000684 | 0.779853 |
| KRT12 | -1.49925 | -0.02826 | -4.2523 | 0.000128 | 0.000695 | 0.760236 |
| FLJ43489 | -1.09312 | 0.300923 | -4.25048 | 0.000129 | 0.000699 | 0.754935 |
| HLA-DRA | 1.113818 | 0.014956 | 4.249693 | 0.000129 | 0.0007 | 0.752645 |
| KRT80 | 1.535815 | -0.43506 | 4.247638 | 0.00013 | 0.000704 | 0.746655 |
| NFE2 | 1.838063 | 0.430649 | 4.243415 | 0.000132 | 0.000712 | 0.734347 |
| BSND | 1.03089 | 0.018679 | 4.236319 | 0.000135 | 0.000724 | 0.713673 |
| IGHD | 1.159832 | -0.23067 | 4.235044 | 0.000135 | 0.000726 | 0.709959 |
| TIMD4 | 2.179733 | 0.729187 | 4.230731 | 0.000137 | 0.000735 | 0.697402 |
| SLAMF8 | 1.179157 | 0.189728 | 4.230458 | 0.000137 | 0.000735 | 0.696607 |
| KLK11 | -1.88992 | -0.13783 | -4.22892 | 0.000138 | 0.000738 | 0.692144 |
| C19orf18 | -1.06372 | -0.1477 | -4.22815 | 0.000138 | 0.00074 | 0.689882 |
| ARHGAP4 | 1.009205 | 0.449086 | 4.225947 | 0.000139 | 0.000744 | 0.683478 |
| RASL11A | 1.09434 | -0.25741 | 4.215388 | 0.000144 | 0.000764 | 0.652766 |
| ATF3 | 1.558812 | -0.09153 | 4.214498 | 0.000144 | 0.000765 | 0.65018 |
| FAM150A | 1.519079 | -0.34673 | 4.210145 | 0.000146 | 0.000773 | 0.637525 |
| SPRR1B | 1.736142 | 0.213791 | 4.207775 | 0.000147 | 0.000777 | 0.630639 |
| NEB | 1.461823 | 0.03386 | 4.201487 | 0.00015 | 0.000788 | 0.612375 |
| ACSM1 | -1.31664 | 0.225351 | -4.20076 | 0.00015 | 0.00079 | 0.610252 |
| EGFL6 | 1.269983 | 0.05842 | 4.200435 | 0.00015 | 0.00079 | 0.60932 |
| SLC38A4 | 1.280274 | 0.238686 | 4.197516 | 0.000152 | 0.000796 | 0.600848 |
| CD69 | 1.038333 | 0.408293 | 4.197438 | 0.000152 | 0.000796 | 0.60062 |
| DKFZP586B0319 | -1.43964 | 0.166207 | -4.19711 | 0.000152 | 0.000796 | 0.599654 |
| GABRA3 | 1.018947 | -0.28796 | 4.196944 | 0.000152 | 0.000796 | 0.599187 |
| CXCL2 | 1.870029 | -0.12854 | 4.196788 | 0.000152 | 0.000796 | 0.598734 |
| CHST4 | 1.382658 | 0.121631 | 4.196376 | 0.000152 | 0.000797 | 0.597537 |
| CRIP3 | 1.738187 | -0.34647 | 4.188776 | 0.000156 | 0.000812 | 0.575486 |
| FAM25A | 2.006367 | -0.19657 | 4.188148 | 0.000156 | 0.000813 | 0.573663 |
| SOX14 | 1.02592 | -0.0463 | 4.187311 | 0.000156 | 0.000814 | 0.571236 |
| DISP2 | -1.15495 | 0.305138 | -4.18498 | 0.000157 | 0.000819 | 0.564465 |
| ZNF818P | -1.00779 | 0.170772 | -4.18264 | 0.000159 | 0.000824 | 0.557693 |
| ADH6 | 1.692722 | -0.44656 | 4.18251 | 0.000159 | 0.000824 | 0.557314 |
| LILRA2 | 1.827612 | 0.084161 | 4.182392 | 0.000159 | 0.000824 | 0.556973 |
| SLC6A4 | -1.15494 | 0.498316 | -4.18073 | 0.000159 | 0.000826 | 0.552147 |
| CXorf61 | 3.666471 | -0.73174 | 4.180619 | 0.00016 | 0.000826 | 0.551833 |
| GPR65 | 1.075045 | 0.224221 | 4.179856 | 0.00016 | 0.000827 | 0.549622 |
| AQP1 | 1.907097 | -0.47897 | 4.173489 | 0.000163 | 0.000841 | 0.531171 |
| CXCL5 | 3.142476 | 0.268779 | 4.171768 | 0.000164 | 0.000844 | 0.526186 |
| LIPG | 1.519573 | -0.39045 | 4.169611 | 0.000165 | 0.000849 | 0.519941 |
| MMP12 | 2.52628 | -0.64233 | 4.16928 | 0.000165 | 0.000849 | 0.518981 |
| TRPA1 | 1.062656 | -0.19162 | 4.1682 | 0.000166 | 0.000851 | 0.515856 |
| CRIP2 | -1.05798 | 0.169923 | -4.16799 | 0.000166 | 0.000852 | 0.515237 |
| CHRDL2 | 2.745282 | -0.29089 | 4.167025 | 0.000166 | 0.000853 | 0.512453 |
| GPBAR1 | -1.21605 | 0.660827 | -4.165 | 0.000167 | 0.000858 | 0.506605 |
| CRHBP | 1.002074 | 0.091951 | 4.163511 | 0.000168 | 0.00086 | 0.502283 |
| C10orf128 | 1.025386 | 0.094777 | 4.155248 | 0.000172 | 0.000879 | 0.478376 |
| SPNS1 | -1.16643 | 0.246935 | -4.15249 | 0.000174 | 0.000885 | 0.4704 |
| C1QA | 1.147646 | 0.073966 | 4.150897 | 0.000175 | 0.000888 | 0.465794 |
| CDH16 | 2.391513 | -0.85914 | 4.147151 | 0.000177 | 0.000896 | 0.454968 |
| C8orf4 | 1.420804 | -0.36115 | 4.147016 | 0.000177 | 0.000896 | 0.454576 |
| HOXB8 | 1.551359 | 0.443904 | 4.145235 | 0.000178 | 0.0009 | 0.449432 |
| SERPINB4 | 1.686259 | 0.32104 | 4.139079 | 0.000181 | 0.000914 | 0.431647 |
| CCL26 | 2.193632 | -0.17442 | 4.138906 | 0.000181 | 0.000914 | 0.431147 |
| SPAG17 | -2.61541 | 1.253819 | -4.13857 | 0.000181 | 0.000915 | 0.430178 |
| ERP27 | 1.646372 | -0.07247 | 4.13793 | 0.000182 | 0.000917 | 0.428331 |
| COCH | 1.24267 | 0.045202 | 4.137156 | 0.000182 | 0.000918 | 0.426095 |
| ACTL8 | 1.9931 | 0.148026 | 4.135956 | 0.000183 | 0.000921 | 0.422631 |
| DLX4 | 1.256571 | -0.08457 | 4.134085 | 0.000184 | 0.000925 | 0.417228 |
| C4BPB | 1.307632 | -0.34886 | 4.132817 | 0.000184 | 0.000928 | 0.413569 |
| TNFRSF6B | 1.724069 | 0.275123 | 4.132028 | 0.000185 | 0.000929 | 0.411292 |
| ZBTB32 | 1.711628 | 0.225389 | 4.13094 | 0.000185 | 0.000931 | 0.408152 |
| FAM65B | 1.632168 | 0.375401 | 4.130914 | 0.000185 | 0.000931 | 0.408077 |
| ZNF528 | -1.44993 | 0.207934 | -4.12959 | 0.000186 | 0.000934 | 0.404269 |
| CYP4B1 | 1.238121 | -0.00017 | 4.126964 | 0.000188 | 0.000939 | 0.39668 |
| CD28 | 1.373499 | 0.311016 | 4.126878 | 0.000188 | 0.000939 | 0.396433 |
| DDX60 | -1.57973 | 0.209787 | -4.12606 | 0.000188 | 0.00094 | 0.394066 |
| MRVI1-AS1 | 1.293429 | -0.08849 | 4.125414 | 0.000188 | 0.000941 | 0.392211 |
| GPRIN3 | 1.357593 | -0.56735 | 4.124906 | 0.000189 | 0.000942 | 0.390744 |
| PLA2G4F | -1.01492 | 0.099052 | -4.12464 | 0.000189 | 0.000942 | 0.389989 |
| DAGLA | 1.376368 | -0.66723 | 4.122666 | 0.00019 | 0.000947 | 0.384286 |
| KIF1A | -1.17417 | 0.486687 | -4.11492 | 0.000195 | 0.000966 | 0.361945 |
| CPS1 | 3.841197 | -2.8186 | 4.11247 | 0.000196 | 0.000972 | 0.3549 |
| PRAP1 | 3.044162 | -2.06321 | 4.108724 | 0.000198 | 0.00098 | 0.34411 |
| PCSK1N | -1.08579 | 0.135663 | -4.10815 | 0.000199 | 0.000982 | 0.342444 |
| FAM84A | 1.474883 | -0.09443 | 4.107804 | 0.000199 | 0.000982 | 0.34146 |
| KCTD15 | -1.07846 | 0.121623 | -4.09803 | 0.000205 | 0.001008 | 0.313316 |
| KEL | 1.004395 | 0.399788 | 4.096319 | 0.000206 | 0.001012 | 0.308406 |
| LOC100506242 | -1.50974 | 0.854935 | -4.08475 | 0.000213 | 0.001039 | 0.275157 |
| SLC3A1 | 2.461061 | -0.68311 | 4.083444 | 0.000214 | 0.001043 | 0.271394 |
| IGF2BP1 | 1.325456 | 0.305555 | 4.080933 | 0.000216 | 0.001047 | 0.264183 |
| GABRP | 1.61445 | -0.13042 | 4.076213 | 0.000219 | 0.001059 | 0.250626 |
| GALNT8 | 1.859927 | -0.03899 | 4.076045 | 0.000219 | 0.001059 | 0.250144 |
| WNK4 | 1.288774 | -0.39675 | 4.073754 | 0.00022 | 0.001066 | 0.243569 |
| FAM3B | -1.13769 | -0.04275 | -4.07108 | 0.000222 | 0.001073 | 0.235901 |
| ORM2 | -1.91953 | -0.11628 | -4.07092 | 0.000222 | 0.001073 | 0.235436 |
| CA12 | 1.018095 | -0.11302 | 4.067949 | 0.000224 | 0.001081 | 0.22691 |
| C19orf6 | -1.35774 | 0.30735 | -4.0669 | 0.000225 | 0.001084 | 0.223892 |
| PSORS1C1 | -1.54467 | -0.08423 | -4.06637 | 0.000225 | 0.001085 | 0.222389 |
| C16orf89 | -2.3299 | 0.059673 | -4.06625 | 0.000225 | 0.001085 | 0.222025 |
| GUCA2A | 2.333488 | -0.40836 | 4.065109 | 0.000226 | 0.001087 | 0.218766 |
| CSF3R | 1.385604 | 0.170574 | 4.063736 | 0.000227 | 0.00109 | 0.214827 |
| AQPEP | 1.097861 | -0.40176 | 4.063648 | 0.000227 | 0.00109 | 0.214574 |
| P2RY2 | 1.457942 | -0.63494 | 4.063022 | 0.000227 | 0.001091 | 0.212781 |
| RPGR | -1.0048 | 0.366948 | -4.06161 | 0.000228 | 0.001095 | 0.20872 |
| SH2D1B | 1.198013 | -0.04083 | 4.057972 | 0.000231 | 0.001105 | 0.198304 |
| PAQR5 | 1.380108 | 0.049548 | 4.055551 | 0.000233 | 0.001113 | 0.191368 |
| GRM3 | -1.46101 | 0.852894 | -4.05229 | 0.000235 | 0.001122 | 0.182016 |
| DEFB1 | -1.7959 | 0.607091 | -4.05027 | 0.000236 | 0.001126 | 0.176242 |
| LOC100289058 | -1.11573 | 0.344184 | -4.04132 | 0.000243 | 0.001153 | 0.150611 |
| NOL4 | -1.75683 | 0.121945 | -4.0403 | 0.000243 | 0.001155 | 0.147698 |
| CXCR2P1 | 1.808019 | 0.213343 | 4.039829 | 0.000244 | 0.001156 | 0.146354 |
| LOC100505894 | 1.256037 | -0.09708 | 4.034534 | 0.000248 | 0.001174 | 0.131209 |
| NLRP14 | 1.227658 | -0.64181 | 4.034444 | 0.000248 | 0.001174 | 0.130952 |
| MYPN | -1.25103 | 0.131377 | -4.03239 | 0.000249 | 0.00118 | 0.125079 |
| LOC100507487 | -1.26742 | 0.612952 | -4.03011 | 0.000251 | 0.001186 | 0.118564 |
| POTEB | 1.777655 | 0.603526 | 4.028365 | 0.000252 | 0.00119 | 0.113575 |
| LTB | 1.373312 | 0.384788 | 4.028335 | 0.000252 | 0.00119 | 0.113489 |
| LOC400043 | -1.45257 | -0.06529 | -4.02754 | 0.000253 | 0.001193 | 0.11122 |
| OR7C2 | 1.687334 | -0.7946 | 4.02686 | 0.000253 | 0.001195 | 0.109274 |
| NLRP4 | 1.053518 | 0.14858 | 4.02598 | 0.000254 | 0.001197 | 0.10676 |
| HPGD | -1.3171 | -0.23081 | -4.02561 | 0.000254 | 0.001198 | 0.105714 |
| HOXC11 | 1.093982 | 0.193126 | 4.025096 | 0.000255 | 0.0012 | 0.104234 |
| GLT1D1 | 1.818199 | -0.07649 | 4.016808 | 0.000261 | 0.001227 | 0.080568 |
| DKFZP434I0714 | -1.0069 | 0.231658 | -4.01413 | 0.000263 | 0.001235 | 0.072939 |
| MIA | 1.83132 | -0.4464 | 4.013035 | 0.000264 | 0.001238 | 0.069801 |
| ZNF556 | 1.620525 | 0.589371 | 4.012263 | 0.000265 | 0.00124 | 0.0676 |
| SYCP2 | -1.4232 | 0.456269 | -4.01063 | 0.000266 | 0.001244 | 0.062952 |
| TLR7 | 1.108852 | 0.355497 | 4.009567 | 0.000267 | 0.001247 | 0.059909 |
| COL11A2 | -2.19594 | 0.571061 | -4.00699 | 0.000269 | 0.001255 | 0.052571 |
| LOC100506253 | 1.446238 | -0.50768 | 4.004738 | 0.000271 | 0.001261 | 0.046136 |
| DMBT1 | 3.569777 | -2.19151 | 4.00473 | 0.000271 | 0.001261 | 0.046115 |
| PRB1 | -1.95109 | 0.927546 | -4.00419 | 0.000271 | 0.001262 | 0.044576 |
| SLC38A1 | 1.273636 | -0.78276 | 4.003093 | 0.000272 | 0.001266 | 0.041447 |
| IGFL2 | 1.387201 | 0.272849 | 4.001209 | 0.000274 | 0.001272 | 0.03608 |
| LGALS7 | 1.243248 | 0.007405 | 3.99962 | 0.000275 | 0.001276 | 0.031551 |
| TEKT2 | -1.17327 | 0.586613 | -3.99097 | 0.000282 | 0.001304 | 0.00691 |
| CSTL1 | 1.012404 | -0.15217 | 3.990651 | 0.000282 | 0.001305 | 0.006005 |
| NCF1 | 1.282041 | 0.456443 | 3.988836 | 0.000284 | 0.001311 | 0.000839 |
| LOC440356 | 1.172676 | -0.00095 | 3.987162 | 0.000285 | 0.001315 | -0.00393 |
| STYK1 | -1.05649 | -0.04948 | -3.98363 | 0.000288 | 0.001327 | -0.01396 |
| IGSF5 | -1.15414 | 0.119773 | -3.98123 | 0.000291 | 0.001334 | -0.0208 |
| HIST1H2AA | 1.34241 | -0.85576 | 3.980535 | 0.000291 | 0.001337 | -0.02278 |
| EPHB6 | -1.31223 | 0.385265 | -3.9774 | 0.000294 | 0.001346 | -0.0317 |
| DEFB4A | 2.494132 | 0.446762 | 3.970788 | 0.0003 | 0.001369 | -0.05048 |
| FCRL3 | 1.140869 | 0.554746 | 3.964655 | 0.000305 | 0.001386 | -0.0679 |
| BASP1 | -1.2118 | 0.420474 | -3.96297 | 0.000307 | 0.001392 | -0.07269 |
| C16orf73 | 1.159985 | -0.02948 | 3.960491 | 0.000309 | 0.0014 | -0.07972 |
| SFRP5 | -1.85116 | 0.40592 | -3.96039 | 0.000309 | 0.0014 | -0.08 |
| ANXA2 | 1.153351 | -0.59616 | 3.95471 | 0.000314 | 0.001419 | -0.09612 |
| KL | -1.0013 | 0.389902 | -3.95435 | 0.000315 | 0.00142 | -0.09716 |
| AGT | 1.33423 | -0.14318 | 3.949503 | 0.000319 | 0.001437 | -0.11089 |
| C2orf54 | 1.794799 | -0.51885 | 3.944551 | 0.000324 | 0.001453 | -0.12492 |
| SLC6A20 | -1.29341 | -0.15095 | -3.93818 | 0.00033 | 0.001478 | -0.14296 |
| LGSN | 2.410685 | 0.783901 | 3.937729 | 0.000331 | 0.001479 | -0.14424 |
| KCNE4 | -1.44119 | 0.024972 | -3.93586 | 0.000333 | 0.001485 | -0.14955 |
| DEFB103B | 1.310623 | -0.21825 | 3.932289 | 0.000336 | 0.001499 | -0.15964 |
| EPHB2 | 1.509633 | -0.85722 | 3.929447 | 0.000339 | 0.00151 | -0.16768 |
| FLJ13744 | 1.54854 | 0.185135 | 3.920762 | 0.000348 | 0.001543 | -0.19223 |
| ANKRD60 | 1.06922 | -0.3005 | 3.918978 | 0.00035 | 0.001549 | -0.19727 |
| SLCO4C1 | -1.37503 | 0.671183 | -3.9097 | 0.000359 | 0.001588 | -0.22349 |
| SOX2 | -1.38433 | -0.09143 | -3.90784 | 0.000361 | 0.001594 | -0.22873 |
| KLK5 | 1.010104 | 0.066007 | 3.907636 | 0.000362 | 0.001595 | -0.2293 |
| AKR1C1 | -1.35079 | -0.1674 | -3.90737 | 0.000362 | 0.001596 | -0.23004 |
| LEMD1 | 1.139277 | -0.06921 | 3.906922 | 0.000362 | 0.001597 | -0.23132 |
| APOBEC1 | -1.27105 | -0.13545 | -3.904 | 0.000366 | 0.001608 | -0.23957 |
| LCN2 | 1.872939 | -0.05042 | 3.900976 | 0.000369 | 0.00162 | -0.24809 |
| CARD18 | 1.04811 | 0.334346 | 3.900252 | 0.00037 | 0.001623 | -0.25013 |
| SCGN | -1.5335 | 0.386746 | -3.89759 | 0.000373 | 0.001634 | -0.25762 |
| LEFTY1 | 3.695909 | -0.54927 | 3.892056 | 0.000379 | 0.001656 | -0.27323 |
| EYA2 | -1.4776 | -0.16105 | -3.89053 | 0.00038 | 0.001662 | -0.27753 |
| SHD | 1.618289 | -0.28605 | 3.888642 | 0.000383 | 0.00167 | -0.28284 |
| SPAG16 | -1.00566 | 0.447731 | -3.88614 | 0.000385 | 0.00168 | -0.28988 |
| CCL19 | 2.263809 | 0.738255 | 3.885465 | 0.000386 | 0.001682 | -0.29179 |
| CLCN4 | 1.045505 | -0.08452 | 3.885116 | 0.000387 | 0.001684 | -0.29277 |
| LOC100505474 | 1.090309 | -0.19945 | 3.881357 | 0.000391 | 0.0017 | -0.30335 |
| KCNJ15 | -1.80933 | 0.381413 | -3.87722 | 0.000396 | 0.001718 | -0.31498 |
| FKSG2 | 1.050625 | -0.7627 | 3.876558 | 0.000396 | 0.001721 | -0.31685 |
| ZNF334 | -1.56985 | 0.286388 | -3.86782 | 0.000407 | 0.001758 | -0.34143 |
| SULT1E1 | 2.239874 | -1.05401 | 3.865925 | 0.000409 | 0.001768 | -0.34674 |
| PLCXD3 | -1.87283 | 0.188218 | -3.86416 | 0.000411 | 0.001775 | -0.3517 |
| TRIM49 | -1.66155 | 0.242506 | -3.86326 | 0.000412 | 0.001778 | -0.35422 |
| CPZ | 1.28169 | -0.43104 | 3.859336 | 0.000417 | 0.001796 | -0.36524 |
| GCG | 3.88141 | -0.42537 | 3.858667 | 0.000418 | 0.001799 | -0.36712 |
| RBL1 | 1.168769 | 0.000236 | 3.857759 | 0.000419 | 0.001803 | -0.36967 |
| TIGIT | 1.125763 | 0.110316 | 3.852711 | 0.000425 | 0.001826 | -0.38383 |
| FP588 | -1.1238 | 0.066786 | -3.8522 | 0.000426 | 0.001827 | -0.38525 |
| KRT23 | 1.767041 | -0.10945 | 3.850383 | 0.000428 | 0.001836 | -0.39036 |
| PRKCB | 1.160452 | 0.374459 | 3.849998 | 0.000429 | 0.001838 | -0.39144 |
| CGNL1 | -1.09096 | 0.198136 | -3.84418 | 0.000436 | 0.001864 | -0.40775 |
| DACT2 | -1.9804 | 0.444984 | -3.84403 | 0.000436 | 0.001864 | -0.40818 |
| CXCL13 | 3.352173 | 0.97105 | 3.83901 | 0.000443 | 0.001886 | -0.42223 |
| PRSS21 | 1.295531 | 0.21915 | 3.833666 | 0.00045 | 0.001913 | -0.43719 |
| PXDN | 1.137041 | -0.16306 | 3.833326 | 0.00045 | 0.001914 | -0.43814 |
| MYO7B | 2.399798 | -1.86413 | 3.830664 | 0.000454 | 0.001928 | -0.44559 |
| HKDC1 | 2.116363 | -1.52628 | 3.827991 | 0.000458 | 0.001942 | -0.45307 |
| JPH1 | 1.040549 | -0.32307 | 3.827977 | 0.000458 | 0.001942 | -0.45311 |
| C2orf74 | -1.28627 | 0.515762 | -3.82794 | 0.000458 | 0.001942 | -0.45321 |
| TCAM1P | 1.265715 | 0.006091 | 3.827404 | 0.000458 | 0.001944 | -0.45471 |
| CPA6 | 1.107975 | 0.255936 | 3.823168 | 0.000464 | 0.001964 | -0.46655 |
| CHRNA2 | 1.034284 | -0.46325 | 3.822874 | 0.000464 | 0.001965 | -0.46737 |
| HLA-DRB5 | 1.163042 | -0.13265 | 3.821934 | 0.000466 | 0.001968 | -0.47 |
| MUC6 | -1.52959 | 0.391046 | -3.81915 | 0.00047 | 0.001982 | -0.47779 |
| FKBP10 | 1.354646 | -0.05198 | 3.816167 | 0.000474 | 0.001996 | -0.48612 |
| ABCG5 | 1.610097 | -0.5301 | 3.814423 | 0.000476 | 0.002005 | -0.49098 |
| NANOS1 | -1.11291 | 0.352287 | -3.81106 | 0.000481 | 0.002021 | -0.50038 |
| GPX3 | -1.25899 | 0.009838 | -3.80913 | 0.000484 | 0.00203 | -0.50577 |
| NR0B1 | -1.80054 | 0.978894 | -3.80692 | 0.000487 | 0.002041 | -0.51194 |
| ADAMTS8 | 1.080498 | -0.15945 | 3.805373 | 0.000489 | 0.002049 | -0.51624 |
| SEC31B | -1.21247 | 0.22698 | -3.80351 | 0.000492 | 0.002059 | -0.52145 |
| FAM155B | -1.20654 | -0.08713 | -3.80313 | 0.000492 | 0.00206 | -0.5225 |
| PADI2 | 1.32892 | 0.044106 | 3.796429 | 0.000502 | 0.002094 | -0.54118 |
| GPX8 | 1.107145 | 0.002056 | 3.795539 | 0.000503 | 0.002098 | -0.54366 |
| FAM135B | -1.01531 | -0.03681 | -3.79178 | 0.000509 | 0.002116 | -0.55412 |
| C3orf32 | 1.153442 | -0.10127 | 3.790304 | 0.000511 | 0.002123 | -0.55824 |
| FBXO2 | 1.227468 | 0.101478 | 3.788072 | 0.000514 | 0.002136 | -0.56446 |
| TLR10 | 1.313537 | 0.522792 | 3.785164 | 0.000519 | 0.002151 | -0.57255 |
| SCG5 | -1.61243 | 0.512485 | -3.78248 | 0.000523 | 0.002163 | -0.58003 |
| LOC595101 | -1.18965 | 0.327344 | -3.78232 | 0.000523 | 0.002163 | -0.58045 |
| LGR6 | 1.048883 | -0.37938 | 3.780378 | 0.000526 | 0.002174 | -0.58587 |
| TMCO5A | -1.25698 | 0.534724 | -3.77979 | 0.000527 | 0.002175 | -0.5875 |
| MAGEA4 | 2.134165 | 0.572141 | 3.776541 | 0.000532 | 0.002192 | -0.59653 |
| CXCL3 | 1.267722 | -0.0641 | 3.773716 | 0.000537 | 0.002207 | -0.60439 |
| LOC100505915 | -1.49107 | 0.259955 | -3.77349 | 0.000537 | 0.002208 | -0.60501 |
| GPR115 | 1.032235 | -0.193 | 3.773377 | 0.000537 | 0.002208 | -0.60533 |
| PTCHD1 | 1.134591 | 0.344535 | 3.76859 | 0.000545 | 0.002231 | -0.61863 |
| SHANK1 | 1.464431 | -0.72635 | 3.765006 | 0.00055 | 0.00225 | -0.62858 |
| LOC100505657 | -1.19587 | 0.303784 | -3.76498 | 0.00055 | 0.00225 | -0.62865 |
| FAM176A | 1.116349 | -0.16088 | 3.763737 | 0.000552 | 0.002256 | -0.6321 |
| KLK7 | 3.220605 | -1.06668 | 3.763062 | 0.000554 | 0.00226 | -0.63398 |
| FGR | 1.036236 | 0.323474 | 3.762715 | 0.000554 | 0.002261 | -0.63494 |
| UGT2B15 | -1.97032 | 0.368371 | -3.76196 | 0.000555 | 0.002265 | -0.63704 |
| LEAP2 | 1.108856 | 0.156758 | 3.752984 | 0.00057 | 0.002315 | -0.66193 |
| FCRL2 | 1.63851 | 0.38074 | 3.749628 | 0.000576 | 0.002332 | -0.67123 |
| FFAR1 | -1.00648 | 0.066276 | -3.74825 | 0.000578 | 0.00234 | -0.67504 |
| LGALS7B | 1.068401 | -0.00927 | 3.748111 | 0.000578 | 0.00234 | -0.67543 |
| CSPG4 | -1.22607 | 0.277006 | -3.74515 | 0.000583 | 0.002357 | -0.68363 |
| C4B | 1.248794 | 0.25214 | 3.744389 | 0.000585 | 0.00236 | -0.68574 |
| FLVCR2 | 1.67628 | -0.37689 | 3.742742 | 0.000587 | 0.002369 | -0.6903 |
| IL7R | 1.175329 | -0.2098 | 3.736806 | 0.000598 | 0.002406 | -0.70673 |
| TMEM130 | -1.52191 | 1.205279 | -3.73508 | 0.000601 | 0.002416 | -0.7115 |
| CHI3L2 | 2.001102 | 0.916045 | 3.733824 | 0.000603 | 0.002423 | -0.71498 |
| F12 | 1.196559 | -0.49084 | 3.733649 | 0.000603 | 0.002423 | -0.71546 |
| GPT | -1.02175 | -0.05264 | -3.72604 | 0.000617 | 0.002463 | -0.73649 |
| GNLY | 1.391448 | 0.166689 | 3.722688 | 0.000623 | 0.002483 | -0.74576 |
| GZMK | 1.306111 | 0.640851 | 3.722644 | 0.000623 | 0.002483 | -0.74588 |
| CLU | -1.19852 | 0.377496 | -3.71678 | 0.000634 | 0.002518 | -0.76207 |
| MIR155HG | 1.434363 | -0.1343 | 3.716268 | 0.000635 | 0.002521 | -0.76348 |
| HERC2P4 | -1.12561 | 0.395497 | -3.71116 | 0.000644 | 0.002552 | -0.77757 |
| FLJ33996 | -1.09316 | 0.203985 | -3.71087 | 0.000645 | 0.002554 | -0.77836 |
| MUC2 | 1.051762 | 0.049188 | 3.710523 | 0.000645 | 0.002555 | -0.77933 |
| FLJ45248 | 1.322301 | -0.55846 | 3.709304 | 0.000648 | 0.002563 | -0.78269 |
| C6orf123 | 1.021329 | -0.30025 | 3.708766 | 0.000649 | 0.002567 | -0.78417 |
| ISL2 | 1.06729 | 0.420213 | 3.706155 | 0.000654 | 0.002579 | -0.79137 |
| RALYL | -1.12228 | 0.482728 | -3.70572 | 0.000654 | 0.002582 | -0.79258 |
| TSKS | 1.029831 | 0.013072 | 3.705272 | 0.000655 | 0.002584 | -0.7938 |
| MILR1 | 1.238322 | 0.144409 | 3.703446 | 0.000659 | 0.002597 | -0.79884 |
| MAGEA12 | 2.452302 | 0.017684 | 3.703088 | 0.000659 | 0.002599 | -0.79982 |
| SPINK5 | -1.21808 | 0.437895 | -3.69722 | 0.000671 | 0.002637 | -0.81597 |
| KANK4 | -1.90168 | -0.02285 | -3.69695 | 0.000671 | 0.002637 | -0.81672 |
| B3GNT6 | -1.83287 | -0.0077 | -3.69499 | 0.000675 | 0.002649 | -0.82211 |
| KCNS3 | -1.38341 | 0.357407 | -3.69338 | 0.000678 | 0.002658 | -0.82655 |
| TNFRSF9 | 1.354965 | -0.04421 | 3.688486 | 0.000688 | 0.002688 | -0.84001 |
| SLC34A2 | 1.001008 | -0.51461 | 3.673239 | 0.000719 | 0.002785 | -0.8819 |
| HSD11B1 | 1.042245 | 0.043002 | 3.672272 | 0.000721 | 0.002792 | -0.88455 |
| DEFA3 | 2.34255 | -0.06984 | 3.670563 | 0.000725 | 0.002802 | -0.88924 |
| TPPP | -1.37516 | -0.32312 | -3.67025 | 0.000725 | 0.002803 | -0.8901 |
| APOA4 | 2.54613 | 0.034252 | 3.662696 | 0.000741 | 0.002855 | -0.91081 |
| GDPD3 | -1.20861 | -0.02665 | -3.66213 | 0.000743 | 0.00286 | -0.91238 |
| LOC100422737 | 1.105026 | -0.02277 | 3.660908 | 0.000745 | 0.002866 | -0.91571 |
| LOC643037 | 1.339709 | -0.50079 | 3.660534 | 0.000746 | 0.002868 | -0.91674 |
| ALDH1L2 | 1.013317 | 0.091107 | 3.655603 | 0.000757 | 0.002902 | -0.93024 |
| CYP3A7 | -1.43193 | -0.01803 | -3.65289 | 0.000763 | 0.00292 | -0.93766 |
| SLC5A9 | 1.587034 | -0.06751 | 3.651351 | 0.000766 | 0.002932 | -0.94188 |
| MFSD4 | -1.495 | 0.489599 | -3.64961 | 0.00077 | 0.002942 | -0.94664 |
| AHSA2 | -1.01546 | 0.440529 | -3.64814 | 0.000773 | 0.00295 | -0.95067 |
| HEPH | 1.889502 | -1.13345 | 3.648077 | 0.000773 | 0.00295 | -0.95084 |
| CD70 | 1.347124 | -0.08395 | 3.647094 | 0.000776 | 0.002957 | -0.95353 |
| C8orf48 | -1.87076 | 1.041207 | -3.64465 | 0.000781 | 0.002974 | -0.96021 |
| ESPNL | 1.493367 | 0.304182 | 3.643881 | 0.000783 | 0.00298 | -0.96231 |
| DSCR8 | 1.975379 | 0.778418 | 3.634336 | 0.000805 | 0.003048 | -0.98839 |
| KIAA0408 | -1.34451 | 0.528136 | -3.63197 | 0.00081 | 0.003066 | -0.99486 |
| RANBP17 | 1.307695 | -0.17839 | 3.629552 | 0.000816 | 0.003084 | -1.00145 |
| ZNF683 | -1.41336 | 0.572606 | -3.62674 | 0.000823 | 0.003106 | -1.00912 |
| TNNI3 | 1.115264 | 0.20061 | 3.62086 | 0.000837 | 0.003146 | -1.02515 |
| KRT6C | 1.53263 | 0.64909 | 3.620788 | 0.000837 | 0.003146 | -1.02535 |
| ZNF577 | -1.0539 | 0.22177 | -3.61236 | 0.000857 | 0.003215 | -1.0483 |
| CD22 | 1.293536 | 0.145455 | 3.611585 | 0.000859 | 0.003221 | -1.05042 |
| CLC | 1.601167 | -0.14931 | 3.603143 | 0.000881 | 0.003288 | -1.07339 |
| SLC27A6 | -1.21268 | 0.787519 | -3.60113 | 0.000886 | 0.003302 | -1.07885 |
| CYR61 | 1.762948 | 0.097235 | 3.600454 | 0.000887 | 0.003307 | -1.0807 |
| MAGEA9 | 1.821873 | 0.631615 | 3.599669 | 0.000889 | 0.003313 | -1.08283 |
| FMO3 | 1.137918 | -0.22205 | 3.59841 | 0.000893 | 0.003323 | -1.08625 |
| MLIP | 1.420271 | 0.171952 | 3.592744 | 0.000907 | 0.003366 | -1.10164 |
| LOC157860 | 1.715473 | -0.24828 | 3.589449 | 0.000916 | 0.003394 | -1.11059 |
| LOC100506523 | -1.09039 | 0.082678 | -3.58298 | 0.000933 | 0.003443 | -1.12814 |
| VIPR1 | -1.05746 | -0.10719 | -3.57561 | 0.000953 | 0.003502 | -1.14812 |
| CXCR2 | 1.72349 | 0.259664 | 3.572464 | 0.000962 | 0.003528 | -1.15663 |
| FGFBP2 | -1.11882 | 0.263779 | -3.56681 | 0.000977 | 0.003576 | -1.17194 |
| C7orf13 | -1.0403 | 0.332112 | -3.56461 | 0.000984 | 0.003594 | -1.17789 |
| HYAL1 | -1.06093 | -0.20654 | -3.56367 | 0.000986 | 0.003602 | -1.18043 |
| LRFN2 | -1.12245 | 0.103877 | -3.55782 | 0.001003 | 0.003649 | -1.19624 |
| LRRC3B | -1.37201 | 0.741916 | -3.5563 | 0.001007 | 0.003659 | -1.20033 |
| KLK12 | 1.527333 | -0.32607 | 3.554173 | 0.001013 | 0.003678 | -1.20609 |
| C11orf86 | -1.7323 | -0.18388 | -3.54555 | 0.001039 | 0.00375 | -1.22937 |
| S100B | 1.09328 | -0.12494 | 3.544906 | 0.001041 | 0.003756 | -1.2311 |
| C20orf85 | 1.021911 | -0.17949 | 3.544128 | 0.001043 | 0.003762 | -1.2332 |
| C21orf37 | 1.04312 | 0.177112 | 3.543304 | 0.001045 | 0.003769 | -1.23542 |
| CST6 | 1.653221 | 0.027036 | 3.532385 | 0.001079 | 0.003866 | -1.26484 |
| MMP3 | 2.120373 | 0.146666 | 3.529706 | 0.001087 | 0.003891 | -1.27205 |
| KRTDAP | -1.40453 | 0.950073 | -3.52815 | 0.001092 | 0.003904 | -1.27625 |
| LOC100507307 | 1.120034 | 0.09409 | 3.527223 | 0.001095 | 0.003912 | -1.27873 |
| MAGEA10 | 1.406876 | 0.559272 | 3.524529 | 0.001103 | 0.003938 | -1.28598 |
| HLA-DPB1 | 1.065592 | 0.136291 | 3.515752 | 0.001131 | 0.004015 | -1.30957 |
| GRIN2B | 1.065457 | -0.01205 | 3.513975 | 0.001137 | 0.004031 | -1.31434 |
| ZNF880 | -1.1404 | 0.334263 | -3.50938 | 0.001152 | 0.004075 | -1.32667 |
| SELP | 1.126757 | 0.007848 | 3.50848 | 0.001155 | 0.004085 | -1.32909 |
| HAS2 | 1.094622 | 0.152744 | 3.507472 | 0.001158 | 0.004092 | -1.33179 |
| EOMES | 1.413134 | 0.654348 | 3.505931 | 0.001163 | 0.004107 | -1.33593 |
| VGLL1 | 1.302653 | -0.13362 | 3.504361 | 0.001168 | 0.004124 | -1.34014 |
| ORM1 | -1.73233 | 0.028429 | -3.5023 | 0.001175 | 0.004143 | -1.34566 |
| FLJ38379 | -1.52194 | 0.843925 | -3.49868 | 0.001187 | 0.004179 | -1.35537 |
| CD72 | 1.240774 | 0.499019 | 3.498235 | 0.001189 | 0.004184 | -1.35656 |
| MUM1L1 | -2.06767 | 1.123619 | -3.49155 | 0.001212 | 0.00425 | -1.37446 |
| BTK | 1.123376 | 0.357544 | 3.490241 | 0.001216 | 0.004262 | -1.37796 |
| LOC389023 | -1.75489 | 0.518794 | -3.48899 | 0.001221 | 0.004271 | -1.38132 |
| COL8A1 | 1.539652 | 0.385127 | 3.48642 | 0.00123 | 0.004299 | -1.38818 |
| MST1P2 | -1.35675 | 0.606908 | -3.48383 | 0.001239 | 0.004327 | -1.39511 |
| KLHL1 | -1.49481 | 0.851954 | -3.48192 | 0.001245 | 0.004344 | -1.4002 |
| CDHR2 | -1.21784 | -0.07962 | -3.47934 | 0.001255 | 0.00437 | -1.40712 |
| SFTA1P | -1.0913 | 0.284592 | -3.47906 | 0.001255 | 0.004371 | -1.40784 |
| KCTD19 | -1.49916 | 0.795332 | -3.47862 | 0.001257 | 0.004375 | -1.40902 |
| CETP | 1.601684 | 0.175046 | 3.47785 | 0.00126 | 0.004382 | -1.41109 |
| GJB5 | 2.512039 | -0.42779 | 3.473791 | 0.001274 | 0.004425 | -1.42193 |
| UBD | 1.867648 | 0.134655 | 3.463942 | 0.001311 | 0.004526 | -1.4482 |
| HLA-DRB4 | 1.176597 | -0.09701 | 3.463616 | 0.001312 | 0.004528 | -1.44907 |
| HRASLS2 | -2.14286 | -0.27141 | -3.46279 | 0.001315 | 0.004535 | -1.45127 |
| COL6A5 | 1.039235 | 0.160015 | 3.460879 | 0.001322 | 0.004558 | -1.45636 |
| ULBP2 | 1.393516 | -0.04308 | 3.457503 | 0.001335 | 0.004597 | -1.46535 |
| SLC6A19 | 1.053705 | -0.40871 | 3.456016 | 0.00134 | 0.004611 | -1.46931 |
| P2RY8 | 1.09956 | 0.640838 | 3.455748 | 0.001341 | 0.004613 | -1.47002 |
| TSPYL5 | -1.06031 | 0.073953 | -3.45482 | 0.001345 | 0.004624 | -1.47249 |
| LOC100506165 | 2.251326 | -0.37477 | 3.454157 | 0.001347 | 0.00463 | -1.47426 |
| LY6D | 1.865397 | -0.06358 | 3.450786 | 0.00136 | 0.004667 | -1.48323 |
| PPP1R14C | -1.28339 | 0.088498 | -3.44878 | 0.001368 | 0.004685 | -1.48857 |
| LRRK2 | 1.165425 | 0.418399 | 3.447261 | 0.001374 | 0.004701 | -1.4926 |
| HSPA4L | 1.139271 | -0.75919 | 3.42543 | 0.001461 | 0.004943 | -1.55055 |
| NETO2 | 1.121768 | -0.37823 | 3.421419 | 0.001478 | 0.004989 | -1.56118 |
| CELF3 | -1.10679 | 0.31181 | -3.41997 | 0.001484 | 0.005004 | -1.56501 |
| DSG3 | 2.50269 | -0.18615 | 3.417621 | 0.001494 | 0.005032 | -1.57123 |
| FMOD | -1.17186 | 0.046451 | -3.41734 | 0.001495 | 0.005035 | -1.57197 |
| MUC12 | 1.558257 | 0.205794 | 3.415199 | 0.001504 | 0.005061 | -1.57764 |
| MAMDC2 | -1.27254 | 0.322334 | -3.40939 | 0.001529 | 0.005133 | -1.59299 |
| SCEL | -1.18357 | 0.329071 | -3.40737 | 0.001538 | 0.005156 | -1.59834 |
| KIF5C | -1.01693 | 0.361446 | -3.40444 | 0.00155 | 0.005188 | -1.60609 |
| PPY2 | 1.23753 | -0.30388 | 3.404009 | 0.001552 | 0.005193 | -1.60722 |
| DPEP1 | 1.47059 | -0.40406 | 3.398482 | 0.001576 | 0.005262 | -1.62181 |
| JAKMIP3 | -1.32135 | 0.74704 | -3.39814 | 0.001578 | 0.005266 | -1.62272 |
| FEZ1 | 1.030054 | -0.00175 | 3.39696 | 0.001583 | 0.005277 | -1.62583 |
| CD19 | 2.085318 | 1.154234 | 3.396349 | 0.001586 | 0.005284 | -1.62744 |
| MUCL1 | 1.150942 | 0.18716 | 3.382818 | 0.001647 | 0.00545 | -1.6631 |
| GABRR1 | 1.053368 | 0.018631 | 3.371703 | 0.0017 | 0.005598 | -1.69232 |
| ITLN1 | 3.104237 | -1.97186 | 3.371245 | 0.001702 | 0.005604 | -1.69353 |
| HOTAIRM1 | -1.1006 | 0.2714 | -3.36973 | 0.001709 | 0.005623 | -1.69752 |
| ANXA2P3 | 1.094193 | -0.70571 | 3.361576 | 0.001748 | 0.005734 | -1.71891 |
| TRAT1 | 1.061016 | 0.451702 | 3.361488 | 0.001749 | 0.005734 | -1.71915 |
| C2orf88 | 1.382151 | -0.44366 | 3.359972 | 0.001756 | 0.005752 | -1.72312 |
| AMZ1 | 1.006415 | 0.294399 | 3.359968 | 0.001756 | 0.005752 | -1.72313 |
| TRIM7 | -1.15321 | -0.15147 | -3.35907 | 0.001761 | 0.005764 | -1.72547 |
| CD180 | 1.358433 | 0.59625 | 3.347685 | 0.001818 | 0.005928 | -1.75531 |
| LRP8 | 1.246524 | -0.37125 | 3.334241 | 0.001887 | 0.006121 | -1.79047 |
| MAGEA8 | 1.066128 | 0.327417 | 3.328359 | 0.001918 | 0.006203 | -1.80582 |
| TLR8 | 1.201415 | 0.088353 | 3.328009 | 0.00192 | 0.006208 | -1.80673 |
| HLA-DQA2 | 1.121764 | -0.39932 | 3.32616 | 0.00193 | 0.006235 | -1.81156 |
| LOC100130811 | 1.072655 | 0.201476 | 3.320537 | 0.001961 | 0.006318 | -1.82622 |
| DUOX1 | -1.50479 | 0.440565 | -3.31808 | 0.001974 | 0.006343 | -1.83263 |
| PDGFRL | 1.053178 | -0.0508 | 3.318007 | 0.001974 | 0.006343 | -1.83281 |
| TCL1A | 2.22049 | 0.595721 | 3.300151 | 0.002075 | 0.006616 | -1.87925 |
| NFASC | -1.01931 | 0.400813 | -3.29786 | 0.002088 | 0.006648 | -1.88519 |
| SCRT2 | 1.021522 | -0.52326 | 3.297091 | 0.002093 | 0.006659 | -1.88719 |
| GRM4 | 1.234142 | 0.229433 | 3.2845 | 0.002167 | 0.006846 | -1.91984 |
| LPL | 1.117426 | 0.168256 | 3.282477 | 0.002179 | 0.006877 | -1.92508 |
| ATP8B4 | 1.007416 | 0.138865 | 3.281905 | 0.002183 | 0.006884 | -1.92656 |
| FLJ40606 | 1.065238 | -0.4135 | 3.277532 | 0.002209 | 0.006956 | -1.93788 |
| NKX3-2 | -1.62312 | 0.304046 | -3.2761 | 0.002218 | 0.006974 | -1.94157 |
| PRSS1 | 2.260069 | 0.005122 | 3.262971 | 0.0023 | 0.007185 | -1.9755 |
| CKMT2 | -1.78933 | 0.287265 | -3.26179 | 0.002307 | 0.007199 | -1.97854 |
| OCLM | 1.001293 | -0.19512 | 3.259777 | 0.00232 | 0.007226 | -1.98374 |
| C21orf88 | -1.10539 | 0.313246 | -3.25337 | 0.002362 | 0.007329 | -2.00026 |
| DSG4 | -1.37192 | 0.571297 | -3.25179 | 0.002372 | 0.007354 | -2.00432 |
| COL5A3 | 1.006447 | -0.10728 | 3.243815 | 0.002425 | 0.007481 | -2.02485 |
| CTAG1A | 3.066368 | 1.457206 | 3.242559 | 0.002433 | 0.007496 | -2.02808 |
| LOC145837 | -1.20208 | 0.094888 | -3.24097 | 0.002444 | 0.007522 | -2.03217 |
| CLECL1 | 1.423535 | 0.341351 | 3.237139 | 0.00247 | 0.007589 | -2.04201 |
| GHRL | 1.712442 | 0.154607 | 3.235797 | 0.002479 | 0.007611 | -2.04546 |
| TREH | -1.3922 | -0.04223 | -3.23354 | 0.002494 | 0.007652 | -2.05126 |
| FAM69C | -1.33155 | -0.34523 | -3.23104 | 0.002512 | 0.0077 | -2.05767 |
| LOC100289094 | -1.2513 | -0.39998 | -3.2286 | 0.002529 | 0.007741 | -2.06394 |
| XAGE1A | 1.844739 | 0.498047 | 3.22717 | 0.002538 | 0.007765 | -2.0676 |
| ALDOB | 2.048224 | -0.36768 | 3.220765 | 0.002584 | 0.007876 | -2.08402 |
| HBD | 1.673018 | 0.097318 | 3.218708 | 0.002598 | 0.007906 | -2.08928 |
| TBX3 | 1.143541 | -0.89195 | 3.218381 | 0.002601 | 0.00791 | -2.09012 |
| SSX8 | 1.248227 | -0.17538 | 3.216049 | 0.002617 | 0.007956 | -2.09609 |
| GATA5 | -1.42801 | -0.37596 | -3.2129 | 0.00264 | 0.008009 | -2.10415 |
| PPY | 1.116893 | 0.023427 | 3.208117 | 0.002675 | 0.0081 | -2.11638 |
| SERPINB7 | 1.247529 | -0.14363 | 3.205157 | 0.002697 | 0.008147 | -2.12394 |
| SIGLEC8 | 1.041891 | -0.08107 | 3.204911 | 0.002698 | 0.008151 | -2.12457 |
| PCDHB9 | 1.001264 | -0.21295 | 3.204152 | 0.002704 | 0.008166 | -2.12651 |
| STON1-GTF2A1L | 1.066259 | 0.069012 | 3.203561 | 0.002708 | 0.008177 | -2.12802 |
| ONECUT2 | 1.977304 | -1.26166 | 3.202339 | 0.002717 | 0.008199 | -2.13114 |
| SLC25A21 | 1.229254 | -0.5887 | 3.200941 | 0.002728 | 0.00822 | -2.13471 |
| FRMPD2 | -1.02776 | 0.371223 | -3.20045 | 0.002732 | 0.00823 | -2.13596 |
| MEP1A | 2.941526 | -1.03257 | 3.187572 | 0.00283 | 0.008478 | -2.16879 |
| VTCN1 | -1.24407 | 0.515639 | -3.1858 | 0.002843 | 0.008513 | -2.17331 |
| AFAP1-AS1 | 2.058804 | -1.80585 | 3.1841 | 0.002857 | 0.008539 | -2.17763 |
| KIAA0226L | 1.077043 | 0.019254 | 3.181347 | 0.002878 | 0.008587 | -2.18463 |
| CTNNA2 | -1.0205 | 0.352822 | -3.1748 | 0.00293 | 0.008702 | -2.20129 |
| LOC221442 | -1.03865 | 0.273118 | -3.17424 | 0.002934 | 0.008714 | -2.20269 |
| SLC39A12 | 1.023061 | 0.055964 | 3.172564 | 0.002948 | 0.008743 | -2.20696 |
| FAM70A | 1.073871 | 0.457147 | 3.165654 | 0.003004 | 0.008877 | -2.22449 |
| SERPINA4 | -1.24874 | -0.00951 | -3.16179 | 0.003036 | 0.008952 | -2.23429 |
| TRIM23 | 1.304003 | 0.008546 | 3.154369 | 0.003098 | 0.009109 | -2.25308 |
| GAL | 1.995109 | -0.41889 | 3.152374 | 0.003115 | 0.009141 | -2.25813 |
| EIF1AY | 1.8311 | -1.68301 | 3.14973 | 0.003137 | 0.009192 | -2.26482 |
| TKTL1 | 1.18124 | -0.17332 | 3.149495 | 0.003139 | 0.009195 | -2.26541 |
| TNFRSF11B | 1.555661 | -0.39695 | 3.144266 | 0.003184 | 0.009301 | -2.27863 |
| ABCA12 | 1.307348 | -0.02005 | 3.13996 | 0.003222 | 0.009393 | -2.2895 |
| ANGPTL4 | 1.137186 | -0.19709 | 3.139443 | 0.003226 | 0.009401 | -2.29081 |
| FADS6 | 1.063111 | -0.34258 | 3.135784 | 0.003258 | 0.009474 | -2.30004 |
| RGS18 | 1.202403 | 0.285112 | 3.132719 | 0.003285 | 0.009529 | -2.30777 |
| GKN2 | -1.69444 | -0.57665 | -3.12878 | 0.003321 | 0.009607 | -2.3177 |
| C3 | 1.396808 | 0.372773 | 3.127007 | 0.003337 | 0.009644 | -2.32216 |
| OR2A7 | -1.12144 | 0.108713 | -3.12629 | 0.003343 | 0.009662 | -2.32396 |
| APOA1 | 2.021938 | 0.501183 | 3.125906 | 0.003347 | 0.00967 | -2.32493 |
| TNFSF11 | 1.080167 | 0.018473 | 3.124194 | 0.003362 | 0.009704 | -2.32924 |
| MMP7 | 2.289926 | -0.11164 | 3.121492 | 0.003387 | 0.009773 | -2.33604 |
| TNFRSF8 | -1.07531 | 0.367134 | -3.11622 | 0.003436 | 0.00989 | -2.3493 |
| SSX4B | 1.30922 | 0.469865 | 3.116149 | 0.003436 | 0.00989 | -2.34947 |
| PCK1 | 1.657592 | -0.78812 | 3.115977 | 0.003438 | 0.009892 | -2.3499 |
| CTSG | -1.50276 | 0.158797 | -3.10886 | 0.003505 | 0.010039 | -2.36777 |
| CD79B | 1.29909 | 0.687093 | 3.107425 | 0.003518 | 0.010062 | -2.37137 |
| VIL1 | -1.39601 | -0.60884 | -3.10426 | 0.003549 | 0.010126 | -2.37931 |
| CXCL9 | 1.540287 | 0.412433 | 3.100377 | 0.003586 | 0.010206 | -2.38904 |
| EGR4 | 1.294796 | -0.43726 | 3.098903 | 0.0036 | 0.010242 | -2.39273 |
| SI | 3.302553 | -1.49663 | 3.095371 | 0.003635 | 0.010316 | -2.40158 |
| PASD1 | 1.044215 | 0.429478 | 3.089005 | 0.003698 | 0.01046 | -2.4175 |
| CXCL6 | 1.290691 | 0.284673 | 3.088754 | 0.0037 | 0.010466 | -2.41813 |
| TNFAIP6 | 1.322352 | 0.22119 | 3.08127 | 0.003775 | 0.010635 | -2.43682 |
| LOC254057 | 1.296469 | -0.12903 | 3.081141 | 0.003777 | 0.010637 | -2.43714 |
| LOC100240734 | -1.16303 | 0.203732 | -3.0726 | 0.003865 | 0.010849 | -2.45844 |
| LOC100506236 | 1.059572 | -0.51186 | 3.067737 | 0.003915 | 0.01097 | -2.47055 |
| XIST | -4.53294 | 4.603129 | -3.06737 | 0.003919 | 0.010979 | -2.47147 |
| PLA2G12B | 1.570951 | 0.28976 | 3.060989 | 0.003987 | 0.011124 | -2.48734 |
| DYNC2H1 | -1.24573 | 0.550534 | -3.05955 | 0.004002 | 0.011162 | -2.49092 |
| AOC3 | -1.04159 | 0.271368 | -3.0467 | 0.004143 | 0.011475 | -2.52282 |
| SHC3 | 1.018546 | 0.290093 | 3.046634 | 0.004143 | 0.011475 | -2.52298 |
| NPTXR | -1.20402 | -0.27244 | -3.04391 | 0.004174 | 0.01155 | -2.52972 |
| NOX1 | 1.390716 | 0.111271 | 3.042514 | 0.004189 | 0.011577 | -2.53319 |
| FLJ32756 | 1.111665 | -0.18757 | 3.041043 | 0.004206 | 0.011613 | -2.53683 |
| UCA1 | 1.775499 | -0.35394 | 3.037 | 0.004252 | 0.011706 | -2.54684 |
| LY6G6C | -1.12005 | 0.060174 | -3.02429 | 0.004398 | 0.012036 | -2.57824 |
| SORCS1 | -1.14993 | 0.559617 | -3.0145 | 0.004515 | 0.012303 | -2.60237 |
| HERC2P7 | -1.53912 | -0.20883 | -3.01321 | 0.00453 | 0.012338 | -2.60556 |
| SLCO1B3 | 1.644178 | 0.563302 | 3.012379 | 0.00454 | 0.01236 | -2.6076 |
| PON3 | 1.090183 | -0.24401 | 3.009777 | 0.004572 | 0.012426 | -2.61401 |
| CLDN7 | 1.844195 | -1.38883 | 2.998892 | 0.004706 | 0.012721 | -2.64076 |
| B3GALT5 | -1.18571 | -0.12848 | -2.99506 | 0.004754 | 0.012834 | -2.65016 |
| APOB | 1.596778 | 0.463881 | 2.985939 | 0.004871 | 0.013083 | -2.67252 |
| STAP1 | 1.404506 | 0.743415 | 2.978935 | 0.004962 | 0.013289 | -2.68965 |
| SH2D1A | 1.14835 | 0.210219 | 2.978867 | 0.004963 | 0.01329 | -2.68982 |
| JSRP1 | 1.204785 | 0.223531 | 2.964829 | 0.005151 | 0.013691 | -2.72409 |
| LOC728012 | -1.48318 | 0.382044 | -2.95579 | 0.005275 | 0.013976 | -2.7461 |
| IL13RA2 | 1.260984 | 0.014652 | 2.955248 | 0.005283 | 0.013989 | -2.74742 |
| KCNJ16 | -1.94777 | 0.713894 | -2.95421 | 0.005297 | 0.014015 | -2.74994 |
| CCND2 | 1.09093 | -0.581 | 2.952881 | 0.005316 | 0.014056 | -2.75318 |
| RFX6 | -1.48796 | -0.05237 | -2.95061 | 0.005348 | 0.014124 | -2.75869 |
| LOC100507056 | 1.45146 | -1.21067 | 2.948815 | 0.005373 | 0.014177 | -2.76306 |
| PEBP4 | -1.90229 | 0.941803 | -2.93718 | 0.00554 | 0.014551 | -2.7913 |
| C8G | 1.354222 | 0.173897 | 2.935288 | 0.005568 | 0.014601 | -2.79588 |
| ANO3 | -1.41893 | 0.772419 | -2.92532 | 0.005716 | 0.014923 | -2.82 |
| MST1 | -1.03587 | 0.490978 | -2.92496 | 0.005721 | 0.014932 | -2.82086 |
| EREG | 1.27426 | -0.12197 | 2.917347 | 0.005837 | 0.015201 | -2.83926 |
| TMEM213 | 1.200319 | 0.376202 | 2.916661 | 0.005847 | 0.015222 | -2.84091 |
| RGS13 | 1.35009 | 0.497187 | 2.915072 | 0.005872 | 0.015281 | -2.84475 |
| ALPK2 | 1.068435 | 0.689873 | 2.912591 | 0.00591 | 0.015362 | -2.85073 |
| DEFA6 | 2.813474 | 0.673419 | 2.911755 | 0.005923 | 0.015384 | -2.85274 |
| HPN | -1.60003 | -0.03861 | -2.91162 | 0.005925 | 0.015387 | -2.85306 |
| HBM | 1.209967 | 0.203471 | 2.902211 | 0.006073 | 0.015722 | -2.87572 |
| LRMP | -1.09975 | 0.835105 | -2.89569 | 0.006177 | 0.015952 | -2.8914 |
| CBLN1 | -1.02794 | 0.561477 | -2.89385 | 0.006207 | 0.016009 | -2.89581 |
| C4orf7 | 3.34657 | 2.224773 | 2.890292 | 0.006265 | 0.016121 | -2.90435 |
| LOC100506123 | -1.00993 | 0.422963 | -2.88381 | 0.006372 | 0.016346 | -2.91988 |
| COX7B2 | 1.27068 | 0.416429 | 2.87472 | 0.006524 | 0.016671 | -2.94164 |
| TNNC2 | 1.092666 | -0.06233 | 2.868248 | 0.006635 | 0.016904 | -2.9571 |
| APOA2 | 1.069828 | -0.23862 | 2.86741 | 0.00665 | 0.016937 | -2.9591 |
| KCNE2 | -1.86652 | 0.113209 | -2.86082 | 0.006765 | 0.017177 | -2.97482 |
| SLC18A1 | 1.054613 | -0.12366 | 2.845316 | 0.007042 | 0.017753 | -3.01169 |
| LOC100506252 | 1.081159 | -0.10587 | 2.839483 | 0.007149 | 0.017977 | -3.02553 |
| BEX1 | -1.42344 | 0.716777 | -2.83393 | 0.007253 | 0.018185 | -3.03869 |
| CYP2D6 | 1.170846 | 0.068753 | 2.832214 | 0.007285 | 0.018244 | -3.04276 |
| ABCC13 | 1.201013 | -0.13443 | 2.829632 | 0.007334 | 0.018338 | -3.04887 |
| KCNB1 | -1.06977 | 0.436701 | -2.82689 | 0.007386 | 0.01844 | -3.05535 |
| TEX11 | 1.109656 | -0.06094 | 2.82355 | 0.00745 | 0.01857 | -3.06325 |
| NAT2 | 1.397638 | -0.11866 | 2.822276 | 0.007474 | 0.018618 | -3.06626 |
| FXYD4 | 1.326875 | 0.088987 | 2.821447 | 0.00749 | 0.018645 | -3.06822 |
| ABHD12B | 1.182501 | 0.368354 | 2.818033 | 0.007556 | 0.018775 | -3.07628 |
| CCKBR | -2.01736 | -0.41015 | -2.80795 | 0.007755 | 0.019196 | -3.10004 |
| TAAR1 | -1.03089 | 0.890642 | -2.80751 | 0.007764 | 0.019212 | -3.10108 |
| FST | 1.052369 | -0.11523 | 2.807413 | 0.007766 | 0.019215 | -3.10131 |
| TFPI2 | 1.117762 | -0.0924 | 2.791248 | 0.008095 | 0.019911 | -3.13929 |
| FOLR3 | 1.140125 | 0.564758 | 2.786892 | 0.008186 | 0.020087 | -3.1495 |
| COLEC12 | -1.14185 | 0.402305 | -2.78282 | 0.008272 | 0.020251 | -3.15903 |
| GC | -1.59044 | -0.08308 | -2.78266 | 0.008276 | 0.020254 | -3.1594 |
| TDRD9 | -1.99067 | 1.468161 | -2.77701 | 0.008396 | 0.020525 | -3.17262 |
| RPS4Y2 | 4.134921 | -4.19054 | 2.776901 | 0.008399 | 0.020528 | -3.17287 |
| DDX3Y | 2.167828 | -2.33888 | 2.776418 | 0.008409 | 0.020547 | -3.174 |
| GYPC | 1.002549 | 0.400122 | 2.775163 | 0.008436 | 0.020597 | -3.17693 |
| C19orf69 | 1.740608 | 0.514764 | 2.771246 | 0.008521 | 0.020744 | -3.18608 |
| FLJ40712 | -1.66098 | 0.870346 | -2.76804 | 0.008591 | 0.0209 | -3.19355 |
| GALNT14 | 1.254638 | 0.552541 | 2.766067 | 0.008634 | 0.020989 | -3.19816 |
| CLEC12A | 1.008736 | 0.147439 | 2.762839 | 0.008706 | 0.021132 | -3.20568 |
| GKN1 | -1.31795 | -0.42193 | -2.76272 | 0.008708 | 0.021133 | -3.20595 |
| ANPEP | 2.672586 | -1.13974 | 2.76267 | 0.00871 | 0.021133 | -3.20607 |
| RIPPLY2 | -1.04475 | 0.501365 | -2.75917 | 0.008788 | 0.021286 | -3.21421 |
| S100A7 | 1.145521 | -0.19959 | 2.753388 | 0.008918 | 0.021559 | -3.22767 |
| MME | 1.149572 | -0.06375 | 2.7475 | 0.009053 | 0.021825 | -3.24134 |
| SLC16A9 | -1.11415 | -0.23591 | -2.74377 | 0.009139 | 0.022001 | -3.24998 |
| MS4A1 | 1.810886 | 0.916379 | 2.739251 | 0.009245 | 0.022203 | -3.26046 |
| FABP1 | 3.123816 | -2.71028 | 2.734385 | 0.00936 | 0.022412 | -3.27172 |
| ADH4 | 2.565332 | -0.87349 | 2.728273 | 0.009506 | 0.022706 | -3.28585 |
| VCY | 1.066155 | 0.000802 | 2.710977 | 0.009932 | 0.023552 | -3.32571 |
| KBTBD10 | 1.044201 | 0.137613 | 2.6992 | 0.010231 | 0.02413 | -3.35275 |
| PMCH | 1.018603 | 0.324051 | 2.683709 | 0.010639 | 0.024943 | -3.3882 |
| PCDHB16 | 1.037098 | 0.290788 | 2.680302 | 0.01073 | 0.025114 | -3.39598 |
| CCDC144NL | -1.0664 | 0.564935 | -2.67619 | 0.010841 | 0.025338 | -3.40536 |
| NCR3 | 1.000912 | 0.31076 | 2.672677 | 0.010937 | 0.025533 | -3.41336 |
| ANXA13 | 2.105487 | -1.59366 | 2.656084 | 0.011402 | 0.026432 | -3.45107 |
| FCRL5 | 1.194256 | 0.275258 | 2.655842 | 0.011409 | 0.026444 | -3.45162 |
| ADH1A | -1.02549 | -0.19341 | -2.65109 | 0.011545 | 0.026713 | -3.46239 |
| CCL25 | 1.975063 | 0.022894 | 2.647275 | 0.011656 | 0.026915 | -3.47102 |
| ELOVL3 | 1.021522 | -0.14544 | 2.641091 | 0.011837 | 0.027254 | -3.485 |
| LOC389831 | -1.1323 | 0.133647 | -2.63849 | 0.011914 | 0.027399 | -3.49089 |
| HLA-DPB2 | 1.155525 | 0.005948 | 2.637031 | 0.011957 | 0.027466 | -3.49417 |
| LOC100128098 | 1.588938 | 0.725708 | 2.635673 | 0.011998 | 0.027552 | -3.49723 |
| SLC25A41 | -1.408 | 0.183251 | -2.63141 | 0.012126 | 0.027787 | -3.50685 |
| DIRAS3 | -1.10746 | 0.628303 | -2.62428 | 0.012343 | 0.028195 | -3.5229 |
| ITLN2 | 1.714594 | -0.16021 | 2.622597 | 0.012395 | 0.028286 | -3.52668 |
| FABP2 | 1.829315 | -0.34704 | 2.619281 | 0.012497 | 0.028463 | -3.53413 |
| TMEM200A | 1.037548 | 0.072387 | 2.619188 | 0.0125 | 0.028463 | -3.53434 |
| RAB3B | -1.14399 | -0.16306 | -2.60707 | 0.012881 | 0.029146 | -3.5615 |
| PLA2G2A | 1.751241 | 0.282557 | 2.60437 | 0.012968 | 0.029299 | -3.56755 |
| CXCR5 | 1.46949 | 0.677762 | 2.60081 | 0.013082 | 0.029501 | -3.57551 |
| ENAM | -1.11255 | 0.764346 | -2.58401 | 0.013637 | 0.030559 | -3.61297 |
| GREM2 | -1.6413 | 0.552748 | -2.57665 | 0.013886 | 0.031027 | -3.62933 |
| ALPI | 1.070204 | 0.127902 | 2.575839 | 0.013914 | 0.031085 | -3.63113 |
| CILP | -1.114 | 0.359967 | -2.57527 | 0.013934 | 0.031122 | -3.6324 |
| PCDH20 | -1.22656 | 0.245168 | -2.57319 | 0.014005 | 0.031251 | -3.63702 |
| OSTalpha | 1.959942 | -1.2862 | 2.56271 | 0.01437 | 0.031926 | -3.66023 |
| RBP2 | 2.608594 | -1.20546 | 2.560111 | 0.014462 | 0.032088 | -3.66597 |
| CXCL17 | -1.01672 | -0.16709 | -2.55212 | 0.014748 | 0.03262 | -3.68361 |
| DAZL | 3.094293 | -3.46498 | 2.5424 | 0.015104 | 0.033225 | -3.70502 |
| DAZ2 | 2.413072 | -2.29975 | 2.534891 | 0.015383 | 0.033767 | -3.72152 |
| C17orf78 | 1.487826 | -0.04876 | 2.527499 | 0.015663 | 0.034287 | -3.73773 |
| DERL3 | -1.09399 | 0.709548 | -2.51676 | 0.016078 | 0.035032 | -3.76122 |
| ASPN | -1.00091 | 0.402344 | -2.51078 | 0.016313 | 0.03544 | -3.77426 |
| KRT7 | 1.486027 | -0.85237 | 2.502226 | 0.016655 | 0.036072 | -3.79289 |
| PNOC | 1.158747 | 0.527887 | 2.49744 | 0.016849 | 0.03642 | -3.80329 |
| LECT1 | 1.416085 | -0.14809 | 2.4951 | 0.016945 | 0.036592 | -3.80837 |
| C2orf40 | -1.54756 | 1.096389 | -2.48547 | 0.017344 | 0.037287 | -3.82923 |
| BLK | 1.116316 | 0.867549 | 2.484166 | 0.017399 | 0.037379 | -3.83206 |
| PDILT | 1.500778 | 0.925058 | 2.481256 | 0.017522 | 0.037594 | -3.83835 |
| CT45A5 | 1.269026 | 0.401813 | 2.477874 | 0.017665 | 0.037853 | -3.84565 |
| LRRC19 | 1.541347 | -0.44278 | 2.477103 | 0.017698 | 0.037915 | -3.84732 |
| PTPN20B | -1.45085 | -0.9114 | -2.47024 | 0.017993 | 0.038408 | -3.86213 |
| CRYGN | 1.076152 | 0.013354 | 2.465635 | 0.018194 | 0.038795 | -3.87204 |
| APOD | 1.09985 | -0.43311 | 2.461454 | 0.018377 | 0.039114 | -3.88103 |
| FOXL2 | 1.096132 | 0.377918 | 2.461004 | 0.018397 | 0.039144 | -3.88199 |
| REG3G | 2.074472 | -0.97114 | 2.457299 | 0.018562 | 0.03945 | -3.88995 |
| CR1L | 1.017288 | 0.587107 | 2.454822 | 0.018672 | 0.039623 | -3.89526 |
| TXLNG2P | 1.640256 | -1.44504 | 2.411709 | 0.020697 | 0.043112 | -3.98713 |
| UTY | 1.078516 | -0.64428 | 2.397614 | 0.021401 | 0.044351 | -4.01691 |
| INSL5 | 1.287201 | 0.457379 | 2.382002 | 0.022206 | 0.045689 | -4.04974 |
| LGI1 | -1.30313 | 1.157478 | -2.37502 | 0.022575 | 0.046344 | -4.06438 |
| KDM5D | 1.553459 | -1.65324 | 2.372264 | 0.022722 | 0.046599 | -4.07014 |
| NECAB2 | -1.05131 | 0.528026 | -2.36523 | 0.023101 | 0.047216 | -4.08484 |
| PF4 | 1.254351 | -0.13151 | 2.36148 | 0.023305 | 0.047567 | -4.09265 |
| INSL4 | -1.06706 | -0.03388 | -2.36143 | 0.023308 | 0.047567 | -4.09275 |
| KRT20 | -1.1681 | -0.48382 | -2.35442 | 0.023695 | 0.048125 | -4.10734 |
| APOBEC2 | -1.08902 | 0.363207 | -2.35157 | 0.023854 | 0.048371 | -4.11327 |
| MEP1B | 1.849473 | -1.01188 | 2.350796 | 0.023897 | 0.04842 | -4.11489 |
| HBB | 1.450417 | 0.038164 | 2.347272 | 0.024095 | 0.048735 | -4.1222 |
| LOC150622 | -1.06756 | 0.698968 | -2.34283 | 0.024347 | 0.049109 | -4.13142 |
| LYPD2 | 1.07861 | 0.201487 | 2.341574 | 0.024419 | 0.049205 | -4.13401 |
| B3GAT1 | -1.19924 | 0.381664 | -2.33467 | 0.024816 | 0.049885 | -4.14831 |
| CRLF1 | 1.009937 | 0.044316 | 2.333668 | 0.024874 | 0.049979 | -4.15037 |
