# Supplementary material for: Explore the active ingredients and potential mechanisms of JianPi QingRe HuaYu Methods in the treatment of gastric inflammation-cancer transformation by network pharmacology and experimental validation
Source: BMC Complement Med Ther. 2023 Nov 14;23:411. doi: 10.1186/s12906-023-04232-0 (PMC10644588; doi:10.1186/s12906-023-04232-0)
Supplement: Supplementary file 7 — Additional file 7: Table S7. Binding energy (kcal/mol) of quercetin target proteins. [file 12906_2023_4232_MOESM7_ESM.docx]

|  | **Target proteins** | | | |
| --- | --- | --- | --- | --- |
| **Ligand** | **IL6R** | **MYC** | **EGFR** | **HIF1A** |
| quercetin | -6.2 | -6.4 | -7.8 | -7.4 |

**Table S7. Binding energy (kcal/mol) of quercetin target proteins.**
