# Supplementary material for: Explore the active ingredients and potential mechanisms of JianPi QingRe HuaYu Methods in the treatment of gastric inflammation-cancer transformation by network pharmacology and experimental validation
Source: BMC Complement Med Ther. 2023 Nov 14;23:411. doi: 10.1186/s12906-023-04232-0 (PMC10644588; doi:10.1186/s12906-023-04232-0)
Supplement: Supplementary file 8 — Additional file 8: Table S8. Interaction between ligands and target proteins. [file 12906_2023_4232_MOESM8_ESM.docx]

|  | **Target protein** | | | |
| --- | --- | --- | --- | --- |
| **Force** | **IL6R** | **MYC** | **EGFR** | **HIF1A** |
| Van der Waals force | TRP A:96, GLN A:81, VAL A:80,  TYR A:47,  VAL A:79,  LYS A:53 | GLN A:11  SER A:15  GLU A:16  ASN B:24  GLU B:28  GLN B:29 | GLY A:796  PRO A:794  LEU A:792  THR A:854  GLU A:762  LEU A:788  MET A:766 | VAL B:422  GLN A:299  THR A:322  GLY A:298  VAL A:323  THR A:327  ASN A:326  SER A:330  SER B:442  SER B:424 |
| Conventional Hydrogen Bond | ARG A:46, ARG A:48 | - | THR A:790  GLN A:791  MET A:793 | TYR A:325  ARG B:440 |
| Pi-Alkyl | ARG A:46, ARG A:48 | LYS A:12  LEU A:19  LEU A:20  ARG A:23  LYS B:21  ALA B:25 | LYS A:745  LEU A:844  ALA A:743  VAL A:726  LEU A:718 | - |

**Table S8. Interaction between ligands and target proteins.**
