# Supplementary material for: Explore the active ingredients and potential mechanisms of JianPi QingRe HuaYu Methods in the treatment of gastric inflammation-cancer transformation by network pharmacology and experimental validation
Source: BMC Complement Med Ther. 2023 Nov 14;23:411. doi: 10.1186/s12906-023-04232-0 (PMC10644588; doi:10.1186/s12906-023-04232-0)
Supplement: Supplementary file 9 — Additional file 9: Table S9. The potential therapeutic targets for JQH and Quercetin in the treatments of gastric inflammation-cancer transformation. [file 12906_2023_4232_MOESM9_ESM.docx]

**Table S9. The potential therapeutic targets for JQH and Quercetin in the treatments of gastric inflammation-cancer transformation.**

| Targtets of JQH | Targets of Quercetin |
| --- | --- |
| POR | POR |
| TP53 | TP53 |
| ERBB2 | ERBB2 |
| EGFR | EGFR |
| AKT1 | AKT1 |
| IRF1 | IRF1 |
| IL1B | IL1B |
| MET | PTGS2 |
| PTGS2 | CCND1 |
| CCND1 | VEGFA |
| VEGFA | CHEK2 |
| CHEK2 | CASP8 |
| CASP8 | BCL2 |
| BCL2 | MYC |
| MYC | BAX |
| BAX | EGF |
| EGF | RB1 |
| RB1 | MMP9 |
| MMP9 | CDKN1A |
| CDKN1A | BIRC5 |
| BIRC5 | HIF1A |
| HIF1A | CASP3 |
| CASP3 | ERBB3 |
| ERBB3 | MMP2 |
| MMP2 | CXCL8 |
| CXCL8 | MAPK1 |
| MDM2 | BCL2L1 |
| CDK4 | GSTM1 |
| MAPK1 | GSTP1 |
| BCL2L1 | JUN |
| GSTM1 | RASSF1 |
| ESR1 | TOP2A |
| GSTP1 | RAF1 |
| JUN | CCNB1 |
| RASSF1 | CLDN4 |
| KDR | CASP9 |
| PGR | ODC1 |
| TIMP1 | PLAU |
| TOP2A | E2F1 |
| ABCC1 | SPP1 |
| PCNA | CYP1A1 |
| MAPK3 | PTGS1 |
| NOS2 | PPARG |
| RAF1 | ABCG2 |
| CCNB1 | NFE2L2 |
| CLDN4 | MMP3 |
| CASP9 | CTSD |
| ODC1 | IGF2 |
| PLAU | RELA |
| CCNA2 | MMP1 |
| CDK2 | AR |
| E2F1 | NQO1 |
| SPP1 | TOP1 |
| CYP1A1 | PARP1 |
| MAPK8 | CAV1 |
| PTGS1 | CDK1 |
| MAPK14 | IFNG |
| PPARG | HSPB1 |
| CYCS | ICAM1 |
| ABCG2 | MPO |
| NFE2L2 | CYP3A4 |
| MCL1 | PRSS1 |
| MMP3 | HMOX1 |
| CTSD | NCF1 |
| IGF2 | RXRA |
| IL4 | FOS |
| RELA | CCL2 |
| ESR2 | STAT1 |
| MMP1 | NFKBIA |
| AR | IGFBP3 |
| NQO1 | SERPINE1 |
| TOP1 | THBD |
| PARP1 | DPP4 |
| CAV1 | PRKCA |
| CDK1 | CYP1A2 |
| IFNG | IL2RA |
| HSPB1 | IL1A |
| BAD | ACP3 |
| ICAM1 | CHUK |
| MPO | SOD1 |
| CYP3A4 | E2F2 |
| PRSS1 | HSPA5 |
| HMOX1 | F3 |
| NCF1 | COL1A1 |
| RXRA | ALOX5 |
| FOS | CYP1B1 |
| FN1 | PTGER3 |
| CCL2 | HSF1 |
| GSK3B | NR1I2 |
| PLA2G4A | CXCL10 |
| PRKCD | RUNX2 |
| STAT1 | CXCL11 |
| CYP19A1 | AHR |
| NFKBIA | IL6R |
| IGFBP3 | NOS3 |
| SERPINE1 | ACHE |
| THBD | HK2 |
| CREB1 | VCAM1 |
| FASN | HSP90AB1 |
| DPP4 | GJA1 |
| PRKCA | SLC2A4 |
| CYP1A2 | PRKCB |
| IL2RA | SELE |
| IL1A | CD40LG |
| XIAP | PPARD |
| ACP3 | KCNH2 |
| CHUK | CXCL2 |
| CHEK1 | DUOX2 |
| SOD1 | PPARA |
| IKBKB | PLAT |
| E2F2 | ADRB2 |
| HSPA5 | GSTM2 |
| CAT | ELK1 |
| F3 | ACACA |
| EPHB2 | PON1 |
| COL1A1 | COL3A1 |
| ALOX5 | DCAF5 |
| CYP1B1 | INSRR |
| PTGER3 | IL10RA |
| HSF1 | EIF6 |
| NR1I2 | AKR1B1 |
| CXCL10 | NR1I3 |
| SLPI | PSMD3 |
| RUNX2 | MAOB |
| CXCL11 | GABRA1 |
| SREBF1 | SULT1E1 |
| AHR |  |
| IL6R |  |
| NOX5 |  |
| PTGES |  |
| NOS3 |  |
| NR3C1 |  |
| ACHE |  |
| HK2 |  |
| VCAM1 |  |
| HSP90AB1 |  |
| CASP7 |  |
| GJA1 |  |
| ADIPOR2 |  |
| SLC6A4 |  |
| SLC2A4 |  |
| EGLN1 |  |
| PRKCB |  |
| GSR |  |
| SELE |  |
| CD40LG |  |
| AKR1C1 |  |
| HTR3A |  |
| PPARD |  |
| RXRB  KCNH2  CXCL2  DUOX2  PPARA  PLAT  ADH1B  ADRA2A  ADRB2  UGT1A1  ADH1C  FOSL1  LDLR  APOB  GSTM2  CA2  NUF2  FOSL2  AKR1C3  ELK1  ACACA  OPRM1  PON1  CD163  COL3A1  TEP1  CHRM3  DCAF5  PPP3CA  MAP2  HSD3B2  HMGCR  HRH1  INSRR  SOAT2  SOAT1  IL10RA  PLB1  EIF6  FABP5  APP  RHO  HSD3B1  SLC6A3  LTA4H  AKR1B1  CHRM1  OLR1  NR1I3  ATP5F1B  ADRA1A  NR3C2  OPRD1  CES1  ADRB1  PSMD3  DPEP1  MAOA  CHRM4  MAOB  PKIA  NCOA1  GABRA1  SULT1E1  CHRNA2 |  |
